# Diagnostic accuracy of surveillance tests for hepatocellular carcinoma in cirrhosis: a systematic review and network meta-analysis

**DOI:** 10.1136/bmjgast-2025-002155

**Published:** 2026-07-17

**Authors:** Gabriel Rogers, Efthymia Derezea, Libby Sadler, Hanyu Wang, Matthew E Cramp, Stephen D Ryder, Kelsey Watt, Penny Whiting, Morwenna Rogers, John Bell, Felicity Oppe, Ken Stein, Nicky J Welton, Hayley E Jones

**Affiliations:** 1 Manchester Centre for Health Economics, University of Manchester, Manchester, UK; 2 Population Health Sciences, Bristol Medical School, University of Bristol, Bristol, UK; 3 UK Health Security Agency, London, UK; 4 Peninsula Medical School, University of Plymouth, Plymouth, UK; 5 Nottingham University Hospitals NHS Trust, Nottingham, UK; 6 Aneurin Bevan University Health Board, Newport, UK; 7 Medical School, University of Exeter, Exeter, UK; 8 Patient representative, NA, UK

**Keywords:** LIVER CIRRHOSIS, HEPATOCELLULAR CARCINOMA, META-ANALYSIS

## Abstract

**Objective:**

For surveillance for hepatocellular carcinoma (HCC) to be effective, tests—including imaging, serological biomarkers (conventional and genomic) and algorithms combining multiple tests—must identify early-stage tumours. We aimed to identify, appraise and synthesise studies reporting the accuracy of all such tests in people with cirrhosis.

**Design:**

Systematic review and network meta-analysis of diagnostic test accuracy (NMA-DTA) data.

**Data sources:**

MEDLINE and Embase (2005 to September 2025) and a published Cochrane review.

**Eligibility criteria:**

English-language, post-2005, one-gate or two-gate studies quantifying diagnostic accuracy of tests to detect HCC in populations wholly comprising people with cirrhosis, excluding those with pre-existing signs and symptoms of HCC.

**Data extraction and synthesis:**

Data extracted by one reviewer, checked by a second and made available in an open-access database. We assessed risk of bias using QUADAS-2. We synthesised data using Bayesian NMA-DTA, accounting for tumour stage and incorporating continuous tests across all possible thresholds.

**Results:**

We included 170 studies (62 643 participants). Of 115 index tests, 97 were amenable to NMA-DTA. Ultrasound appears no better than alpha-fetoprotein at detecting very-early-stage HCC (sensitivity 0.34 (95% CrI 0.21 to 0.55) vs 0.39 (95% CrI 0.28 to 0.47)), only becoming superior as stage advances. Least affected by stage are contrast-enhanced MRI (sensitivity 0.70 (95% CrI 0.50 to 0.84) very-early; 0.86 (95% CrI 0.73 to 0.94) early; 0.90 (95% CrI 0.67 to 0.98) advanced) and CT (0.68 (95% CrI 0.26 to 0.93) very-early; 0.77 (95% CrI 0.29 to 0.95) early; 0.92 (95% CrI 0.49 to 0.99) advanced). No genomic biomarkers show convincing improvements over combinations of conventional blood-markers. Most studies are at high risk of bias, but conclusions are robust when restricting to studies with favourable methodological characteristics.

**Conclusions:**

Using advanced synthesis methods, we found that tests have low sensitivity for detecting early-stage HCC. Given rising prevalence of cirrhosis and HCC, we need better tests and a stronger evidence-base to inform optimal surveillance strategies.

**PROSPERO registration number:**

CRD42022357163.

**WHAT IS ALREADY KNOWN ON THIS TOPIC**

- Guidelines agree that surveillance for hepatocellular carcinoma in people with cirrhosis should save lives, but there is disagreement about the best tool(s) for the job, with uncertainty encompassing longstanding options like alpha-fetoprotein assay and new approaches like multitest algorithms and genomic biomarkers. No previous evidence synthesis has compared all relevant options and robustly addressed the surveillance-critical objective of identifying early-stage tumours.

**WHAT THIS STUDY ADDS**

- This wide-ranging systematic review provides evidence on 115 different tests and combines evidence on 97 of these in a single, coherent quantitative synthesis (network meta-analysis). Stage-stratified analysis suggests that ultrasound—the backbone of most surveillance programmes—misses many of the most treatable cancers, though combining it with alpha-fetoprotein should improve sensitivity, while cross-sectional and/or contrast-enhanced imaging finds most early-stage tumours.

**HOW THIS STUDY MIGHT AFFECT RESEARCH, PRACTICE OR POLICY**

- Surveillance programmes for hepatocellular carcinoma commonly rely on tools that lack sensitivity to identify tumours that are amenable to the most effective treatments. Our findings will inform new decision-analytic evaluations of the benefits, harms and costs of surveillance for hepatocellular carcinoma in people with cirrhosis.

## Introduction

 Hepatocellular carcinoma (HCC) is relatively unusual among cancers in having a precursor condition that is identifiable in a substantial majority of cases—cirrhosis.^1^ As a result, people with cirrhosis represent an obvious target for periodic follow-up (‘surveillance’) for HCC. For surveillance to provide patient benefit, it must rely on diagnostic test(s) that can identify tumours at an earlier, more treatable stage than would be the case were people only to present when symptoms develop.

A wide array of diagnostic tests is available to identify HCC in people with cirrhosis, including imaging, serum biomarkers, genomic targets and algorithms comprising combinations of such tests. However, there is pervasive uncertainty about the best tool(s) for the job. While all authorities agree that ultrasound (US) forms the mainstay of HCC surveillance, other tests have varying degrees of support. The role of serum alpha-fetoprotein (AFP) is especially ambiguous. Both the American Association for the Study of Liver Diseases (AASLD)^2^ and the Japan Society of Hepatology (JSH)^3^ recommend routine AFP-testing alongside US, although they disagree about the threshold at which cases warrant further investigation (20 ng/mL in AASLD; 200 ng/mL in JSH). The European Association for the Study of the Liver (EASL)^4^ primarily recommends US, but says adding AFP is ‘a reasonable option’ while, in England and Wales, the National Institute for Health and Care Excellence sits on the fence, recommending US ‘with or without AFP’.^5^ EASL and AASLD recommend against other serum biomarkers, but JSH encourages the use of des-gamma-carboxy-prothrombin (DCP) and lens culinaris agglutinin-reactive fraction of fetoprotein (AFP-L3). A similar divide emerges regarding cross-sectional imaging: EASL and AASLD discourage using MRI or CT (though AASLD makes an exception for ‘select patients in whom US-based surveillance is suboptimal’), whereas JSH recommends that contrast-enhanced MRI or dynamic CT ‘may be performed’ in people with cirrhosis. No current guidelines rely on genomic biomarkers or multitest algorithms, though researchers envisage an important role for them.^6^

With so many options reported in a diffuse literature-base, it is challenging to take a coherent view of the relative merits of each possible approach. While multiple published systematic reviews cover some tests,^7^,^14^ none provides estimates across the range of tools that might be used for surveillance. There is therefore a need to collect available evidence for all relevant tests, assess its susceptibility to bias according to consistent standards and undertake unified, comprehensive quantitative synthesis. Network meta-analysis (NMA) is an approach to evidence synthesis that integrates data for multiple decision-options in joint analyses, strengthening inference about the performance of each option and making it easier to compare between them. NMA has become embedded in evidence-based decision-making comparing the effectiveness of multiple competing therapeutic interventions.^15^ However, it is less common for researchers to apply analogous techniques to assess the accuracy of diagnostic technologies.^16^

NMA of diagnostic test accuracy (NMA-DTA) enables us to estimate the pooled sensitivity and specificity of each test while accounting for correlations resulting from multiple studies reporting the accuracy of multiple tests. With extensions to the approach, we can also explore important subtopics, such as: How does the accuracy of continuous tests depend on the threshold used to indicate positivity? How does test accuracy vary according to aetiology of cirrhosis? How do the options compare in detecting HCC of different stages (particularly when it comes to the surveillance-critical objective of identifying early-stage tumours)?

To address these questions, we conducted a systematic review and evidence synthesis, as part of a wider project funded by the National Institute for Health and Care Research (NIHR134670).

## Methods

We published our protocol^17^ and registered it in PROSPERO. This report conforms to the Preferred Reporting Items for Systematic Reviews and Meta-Analyses extension for diagnostic test accuracy studies^18^ (online supplemental appendix S1). All data and analysis code appear in our GitHub repository: www.github.com/HCCsurv/NMA-DTA.

### Identifying evidence

#### Eligibility criteria

We included studies published from 2005 onwards (note online supplemental appendix S2) that provide sufficient data to estimate the sensitivity and specificity of ≥1 test of interest. The target condition is HCC of any stage. Eligible studies recruited adults (≥18 years) with cirrhosis of any aetiology and no known history of HCC at the time of the index test. We included both ‘one-gate’ studies (those with one set of eligibility criteria for all participants) and ‘two-gate’ studies (those with separate sampling schemes for HCC and HCC-free controls).^19^ We excluded conference abstracts and studies in languages other than English (online supplemental appendix S3 tabulates non-English studies that appeared potentially relevant). We excluded studies in which participants had signs or symptoms suggestive of HCC.

Tests of interest were: US (distinguishing between contrast-enhanced US (CEUS) and B-mode US); cross-sectional imaging (multiphase CT; MRI, distinguishing between contrast-enhanced and non-contrast); conventional biomarkers (AFP; AFP-L3; DCP); genomic biomarkers (any, provided the study reported a validation cohort and not only a discovery cohort); multicomponent algorithms incorporating one or more of these tests (any, provided the study assessed it as a binary classifier with a prespecified threshold).

Initial piloting showed that reference standards were often unclearly reported, with very few studies clearly using a reliable approach. Therefore, we accepted any reference standard investigators considered appropriate, including explant pathology following transplantation (where transplantation is unrelated to HCC), histology of resected or biopsied lesions and radiological follow-up. We then consider the appropriateness of the reference standard as part of assessing risk of bias (see below).

#### Information sources

We relied on an existing Cochrane review to identify the core of our evidence-base for AFP and US, as the review has similar eligibility criteria to ours, and a broad search strategy.^11^ We obtained full texts of all studies the authors included (n=346) as well as those they had excluded at full-text screening (n=219) and assessed them against our eligibility criteria. We carried out database searches to identify evidence on other index tests, and evidence on AFP and US published since the Cochrane review search (June 2020). We searched MEDLINE and Embase via Ovid, and the Cochrane Database of Systematic Reviews until 5 September 2025. Online supplemental appendix S4 gives full search strategies. We checked references of related systematic reviews.

#### Study selection

Two reviewers (from LS, HW, GR, MR and others (see acknowledgments)) independently screened titles and abstracts. We obtained full-text copies of studies that we could not confidently exclude and, alongside the included and excluded studies from the Cochrane review, either one or two reviewers (LS, HW, GR and others) assessed each for inclusion (see online supplemental appendix S2). We resolved disagreements through discussion.

### Extracting and appraising evidence

#### Data collection process

Data were extracted by one reviewer (LS or HW) using a bespoke database with facility to record qualitative and quantitative data, along with free-text notes, that we piloted on a small number of papers. A second reviewer (HW or GR) checked all data extraction, resolving disagreements through discussion. We extracted descriptive data for each included study and 2×2 data on test accuracy (number of true positives, false positives, true negatives and false negatives) at all reported thresholds. If studies did not explicitly report 2×2 data, we constructed these from reported measures of diagnostic accuracy where possible. We extracted accuracy data reported at the patient level if available; otherwise, we extracted any outcome level. We extracted 2×2 data separately for subgroups of interest (eg, by aetiology of cirrhosis), where reported. The full database is available in our open-source repository.

#### Risk of bias assessment

We used QUADAS-2,^20^ and QUADAS-C for comparative accuracy studies,^21^ to assess the risk of bias of included studies across four domains: patient selection, index test, reference standard and flow and timing. If ≥1 domain was rated ‘high’, we considered the study at high risk of bias. If all domains were rated ‘low’, we considered the study at low risk of bias. Otherwise, we rated the study as ‘unclear’ risk of bias. For the reference standard domain, we only rated studies as low risk of bias if they either used explant pathology following transplantation, histology of resected or biopsied lesions or radiological follow-up with ≥6 months’ follow-up after the index test for patients without HCC. For the flow and timing domain, we rated studies at low risk of bias if they had ≤3 months between index and reference tests.

### Statistical analysis

We performed meta-analyses using the Bayesian statistical software JAGS,^22^ via the R2jags package^23^ in R V.4.3.3.^24^ We assessed convergence using trace plots and Gelman-Rubin statistics.^25^

#### Connected synthesis network

Our analyses focus on a ‘connected synthesis network’ comprising all tests assessed in ≥1 comparative study (reporting on the accuracy of ≥2 tests). Analyses also include studies that evaluate only one index test if they report on the accuracy of a test already within the network. We treat combinations of tests as separate nodes—for example, AFP_·AND·_DCP (a ‘conjunctive’ combination of index tests counting results as positive if positive on both tests) or AFP_·OR·_DCP (a ‘disjunctive’ combination of index tests counting results as positive if positive on either test).

#### Network meta-analysis of diagnostic test accuracy

We fitted a newly proposed NMA-DTA model, that borrows elements of Nyaga *et al*’s analysis of variance (ANOVA) model for networks of tests^26^ and Jones *et al*’s model for (single-test) meta-analysis across multiple thresholds,^27^ allowing us to include both binary and continuous tests in a unified synthesis.^28^ Online supplemental appendix S5.1 provides full details. We give pooled estimates of sensitivity and specificity, and estimated ranks (where 1st=‘best’, etc), each with 95% credible intervals (CrIs). For imaging tests, we also plot data in receiver-operating-characteristic (ROC) space, with 95% credible ellipses and hierarchical summary ROC curves.^29^

#### Meta-analysis of each test separately

As sensitivity analyses, we show independent meta-analyses of each index test with data in ≥2 studies. For binary tests evaluated in ≥3 studies, we use the binomial bivariate random-effects model^30 31^; for tests evaluated in two studies, we fitted binomial univariate fixed-effect models. For continuous biomarkers, we use Jones *et al*’s multiple-thresholds model with log-transformed threshold.^27^

#### Variability in sensitivity by tumour stage

As the ability of each test to identify early-stage HCC is a critical determinant of surveillance’s utility, we undertook stage-stratified analysis of sensitivity. To make this possible, we mapped study-level detail on the size/stage of diagnosed HCCs onto approximately equivalent categories in Barcelona Clinic Liver Cancer (BCLC) staging system (online supplemental appendix S5.2 provides details). Only a minority of studies explicitly report sensitivity by stage (‘subgroup data’), so we also use information from studies that report ‘overall’ sensitivity and the proportion of participants with HCCs in each group. We fitted a meta-analysis model for multiple disease states that makes use of all available data by assuming that overall sensitivity is an average of stage-specific sensitivities, weighted according to these proportions.^32^ We report stage-stratified estimates of sensitivity for each test with subgroup data from ≥1 study and ≥4 studies providing some relevant data. For continuous tests, where sufficient information is available, we report stage-stratified estimates of sensitivity across the whole range of observed thresholds.

#### Exploration of heterogeneity

We reran the NMA-DTA restricted to one-gate studies, as two-gate (case-control) designs are known to overestimate accuracy.^19 33^ We also reran test-specific meta-analyses restricted to studies at low risk of bias in the ‘reference standard’ domain of QUADAS-2. We used meta-regression, for tests with relevant information reported in ≥4 studies, to explore whether sensitivity and/or specificity varies by study-level characteristics: (a) predominant aetiology of cirrhosis (alcohol-related liver disease, hepatitis B virus (HBV), hepatitis C virus (HCV), non-alcoholic fatty liver disease), (b) severity of cirrhosis (proportion of study participants with Child-Pugh score A), (c) average age of participants and (d) proportion male.

### Patient and public involvement

Two authors (JB and FO) are patients who, between them, have lived experience of cirrhosis, undergoing surveillance tests and receiving a diagnosis of HCC. We also discussed our plans for this review with a wider group of patients at a patient and public workshop at the project’s inception.

## Results

### Identification and appraisal of evidence

We included 170 unique studies (online supplemental appendix S6), comprising an aggregate of 62 643 participants. Online supplemental appendix S8 describes each included study in detail. Table 1 summarises key characteristics. A minority took place in surveillance programmes; most (67%) have a two-gate design, comparing people with HCC with controls identified elsewhere. In one-gate studies, mean prevalence of HCC is 16%, ranging from <1%^34^ to >70%.^35^ Low-prevalence studies tend to be conducted prospectively in surveillance programmes; high-prevalence studies are disproportionately those with explant pathology as a reference standard. The region of the world contributing most studies is North Africa and the Middle East, mostly owing to numerous small studies from Egypt (47 studies recruiting an aggregate of 5362 participants). However, a plurality of participants comes from the USA (22 703 across 25 studies). Almost 90% of evidence comes from studies in which the predominant aetiology of cirrhosis is viral, with HCV the most common cause. Reference standards are variable between and within studies.

**Table 1:** Characteristics of included studies

Characteristic	Number of studies (%)	Aggregate number of participants (%)
Study design		
One-gate	56 (32.9)	39 239 (63.7)
Two-gate	114 (67.1)	22 384 (36.3)
Method of recruitment		
Surveillance programme	42 (24.7)	28 143 (45.7)
Clinical cohort	128 (75.3)	33 480 (54.3)
Geographical setting		
Africa—N & Middle-East	55 (32.4)	6614 (10.7)
America—Latin	3 (1.8)	1091 (1.8)
America—N	25 (14.7)	22 703 (36.8)
Asia—E (exc. Japan)	44 (25.9)	20 249 (32.9)
Asia—Japan	4 (2.4)	571 (0.9)
Asia—S	7 (4.1)	2281 (3.7)
Australasia	4 (2.4)	735 (1.2)
Europe—C & E	3 (1.8)	213 (0.3)
Europe—Italy	10 (5.9)	2974 (4.8)
Europe—N & W (exc. Italy)	13 (7.6)	3730 (6.1)
Intercontinental	2 (1.2)	462 (0.7)
Predominant aetiology of cirrhosis		
Alcohol-related liver disease	11 (6.5)	1756 (2.8)
Hepatitis B	44 (25.9)	19 055 (30.9)
Hepatitis C	84 (49.4)	35 046 (56.9)
Non-alcoholic fatty liver disease	5 (2.9)	1276 (2.1)
Other/missing	26 (15.3)	4490 (7.3)
Reference standard establishing presence of HCC		
Explant	11 (6.5)	4224 (6.9)
Histology	13 (7.6)	1535 (2.5)
Histology or imaging	41 (24.1)	16 414 (26.6)
Imaging	87 (51.2)	34 896 (56.6)
Other	18 (10.6)	4554 (7.4)
Reference standard establishing absence of HCC		
Explant	11 (6.5)	4224 (6.9)
Histology	3 (1.8)	172 (0.3)
Histology or imaging	31 (18.2)	13 414 (21.8)
Imaging	63 (37.1)	31 078 (50.4)
Other/not reported	62 (36.5)	12 735 (20.7)

HCC, hepatocellular carcinoma.

We judge that the evidence-base is at high risk of bias throughout. Only two studies achieve an overall QUADAS-2 assessment of ‘low risk’^36 37^; one more is at ‘unclear’ risk,^38^ with the remaining 167 all having at least one characteristic that puts them at high risk of bias (online supplemental appendix S9). Studies are least susceptible to bias in the ‘index test’ domain. However, more than half the included studies are at high risk of bias in each of the other three domains. For ‘patient selection’, two-gate designs and/or non-consecutive samples predominate; for ‘reference standard’, differences in approach to HCC cases and HCC-free controls are very common (eg, histology may be available for people who underwent biopsy or resection, but not for people in whom treating clinicians do not suspect HCC); for ‘flow and timing’ investigators often allowed extended periods of time between index and reference tests.

**Figure 1:**
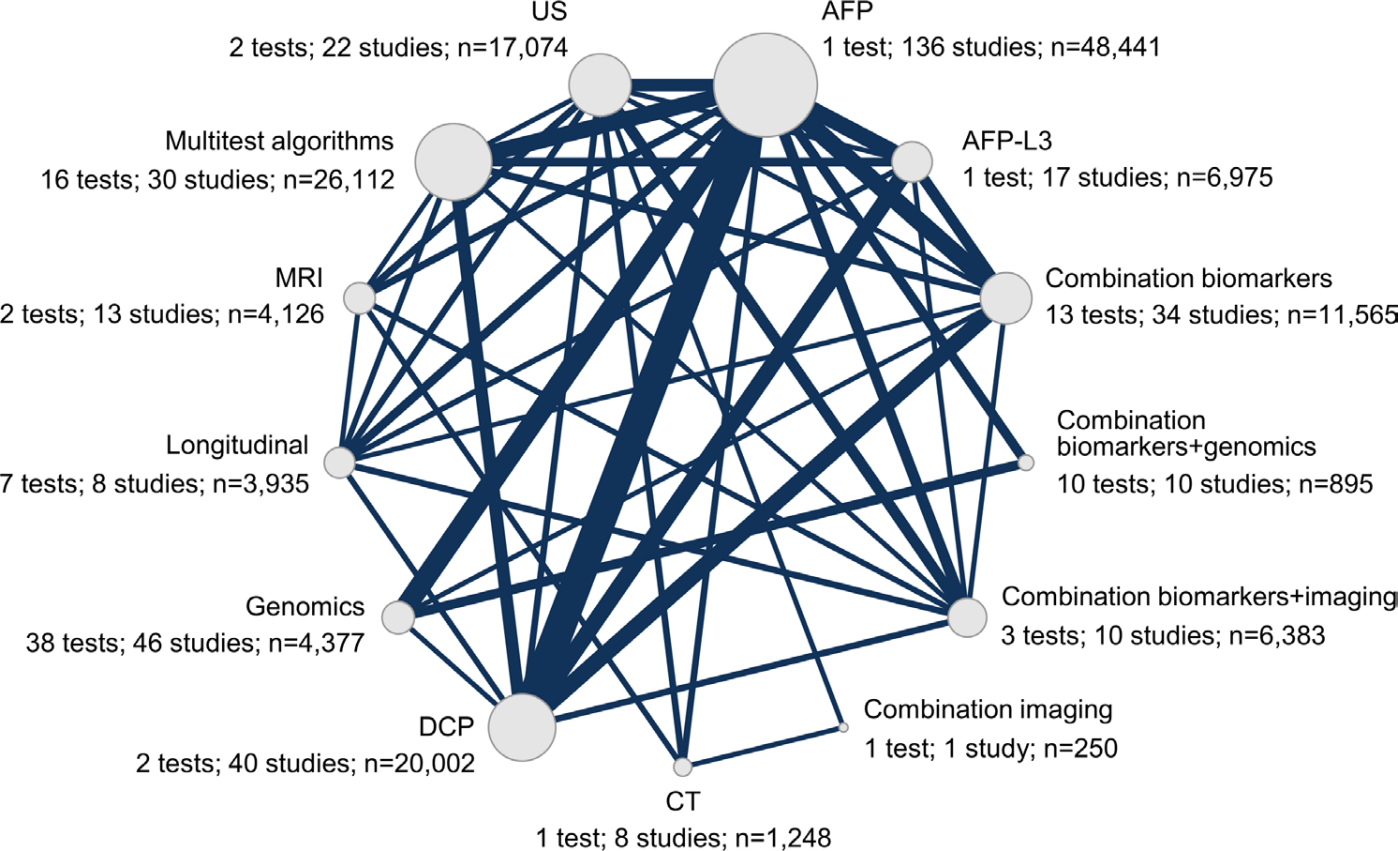
Connected synthesis network. Node size is proportional to number of participants; thickness of interconnecting lines is proportional to number of studies comparing the two nodes. ‘Biomarkers’ refers to conventional biomarkers (AFP, AFP-L3 and DCP). Note that all syntheses treat individual tests as discrete units of analysis; we show class-level groupings here for clarity of exposition only. AFP, alpha-fetoprotein; AFP-L3, lens culinaris agglutinin-reactive fraction of AFP; DCP, des-gamma-carboxy-prothrombin.

The included studies evaluate 115 index tests. Of these, 18 have no comparators in common with the core network (online supplemental appendix S10). This leaves 97 index tests, reported in 160 studies, to form the connected synthesis network. Figure 1 gives a summary of the network; for a test-by-test visualisation, see online supplemental appendix S11. The network is sparse, with the accuracy of 66/97 (68%) tests being informed by only one study. Four tests are continuous biomarkers evaluated across multiple thresholds: AFP (n=136 studies, ≤48 thresholds, most commonly 20 ng/mL), AFP-L3 (n=17, ≤2 thresholds, most commonly 10%) and DCP (distinguishing between mAU/mL (n=29, ≤7 thresholds, most commonly 40 mAU/mL) and ng/mL (n=11, ≤2 thresholds, most commonly 7.5 ng/mL), as conversion between the two is laboratory-specific). There are five imaging tests: US (n=20), dynamic contrast-enhanced MRI (n=11), multiphase CT (n=8), CEUS (n=2) and non-contrast MRI (n=2). Included genomic tests encompass 38 different molecular targets reported across many small studies (n=46). There are 16 multitest algorithms, the most common of which combines gender, age, AFP-L3, AFP and des-carboxy-prothrombin (GALAD; n=7). Combinations of ≥2 tests represent 27 unique nodes in our network; the most common are AFP_·OR·_US (n=8) and AFP_·OR·_DCP (mAU/mL) (n=7). Finally, seven tests measure longitudinal progression of a relevant tool (AFP n=7; GALAD n=1). Online supplemental appendix S11 gives more details on the quantity of data included for each test.

### Evidence synthesis

#### Network meta-analysis of diagnostic test accuracy

**Figure 2:**
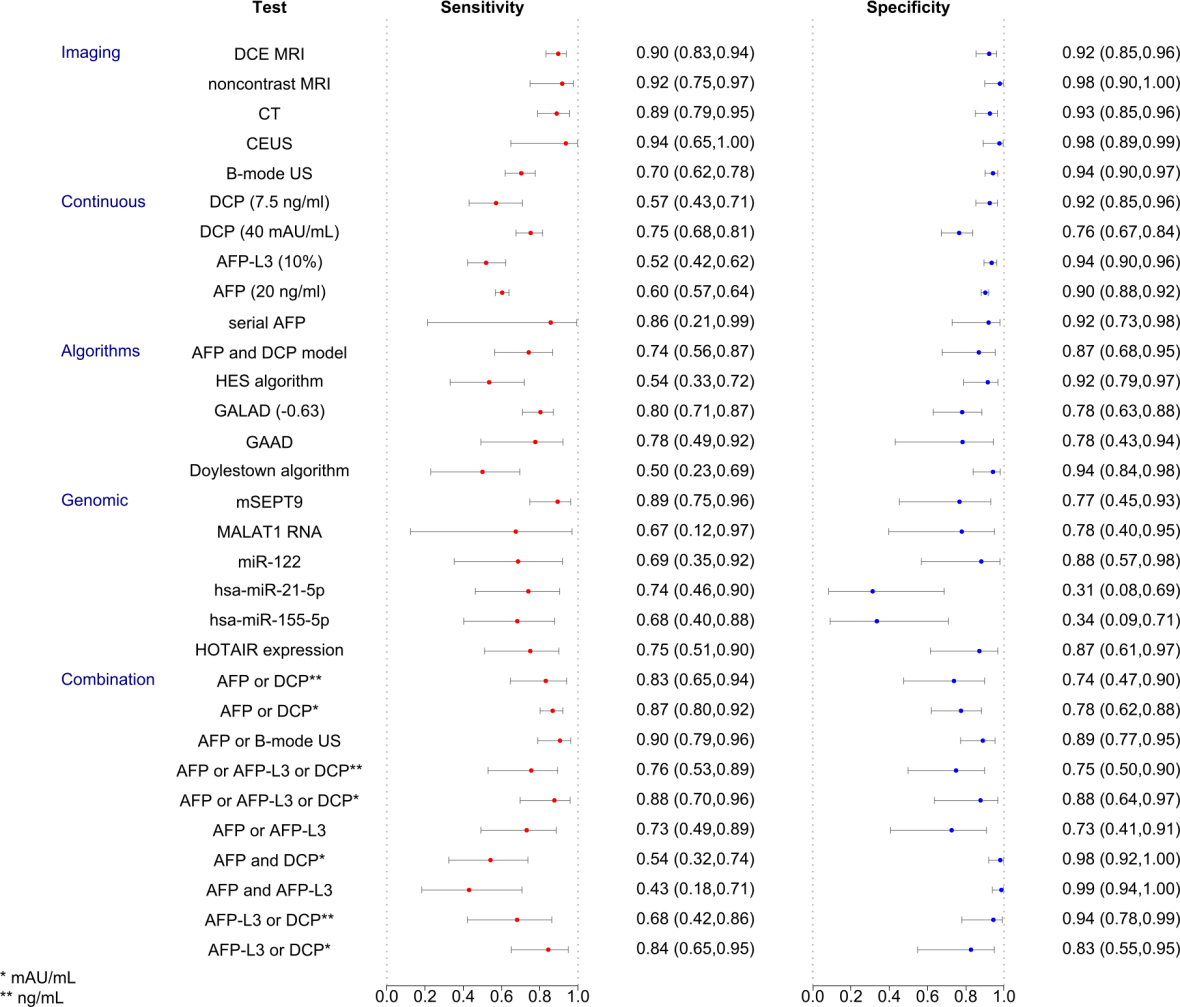
Pooled estimates of sensitivity and specificity for detecting HCC of any stage for each test reported in ≥2 studies. AFP, alpha-fetoprotein; AFP-L3, lens culinaris agglutinin-reactive fraction of AFP; CEUS, contrast-enhanced US; DCE MRI, dynamic contrast-enhanced MRI; DCP, des-gamma-carboxy-prothrombin; GAAD, gender–age–AFP–des-carboxy-prothrombin; GALAD, gender–age–AFP-L3–AFP–des-carboxy-prothrombin; HCC, hepatocellular carcinoma; HES, hepatocellular carcinoma early detection screening; HOTAIR, Hox transcript antisense intergenic RNA; MALAT1, metastasis-associated lung adenocarcinoma transcript 1; miR, microRNA; mSEPT9, methylated Septin9.

Figure 2 shows pooled estimates of sensitivity and specificity of all tests in the network evaluated in ≥2 studies (for tests evaluated in one study, see online supplemental appendix S12). Online supplemental appendix S13 shows rank statistics and plots. No option is clearly ‘best’. CEUS has the highest estimated sensitivity, but with considerable uncertainty (see the Discussion section). The other most sensitive tests are mostly cross-sectional and/or contrast-enhanced imaging. For specificity, AFP_·AND·_AFP-L3 ranks highest followed by AFP_·AND·_DCP (mAU/mL), non-contrast MRI and CEUS.

**Figure 3:**
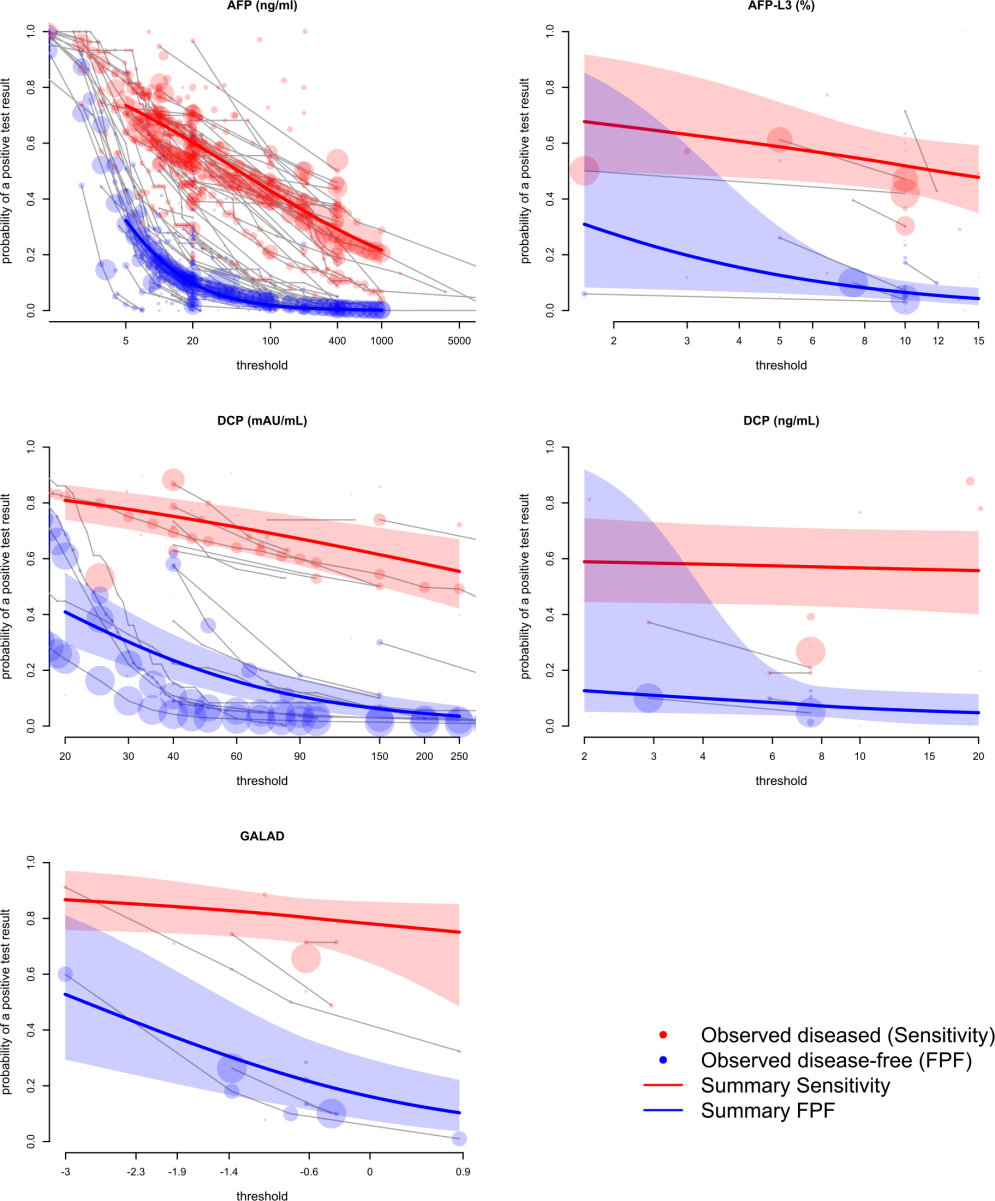
Sensitivity and false positive fraction (FPF=1−specificity) of continuous biomarkers across thresholds for detecting HCC of any stage. Shaded regions represent 95% CrIs; circles represent observed study-specific estimates (size proportional to number of participants); grey lines link estimates from the same study. AFP, alpha-fetoprotein; AFP-L3, lens culinaris agglutinin-reactive fraction of AFP; DCP, des-gamma-carboxy-prothrombin; FPF, false positive fraction; GALAD, gender–age–AFP-L3–AFP–des-carboxy-prothrombin; HCC, hepatocellular carcinoma.

Figure 3 shows findings for continuous biomarkers assessed across all reported thresholds. For all tests, we see heterogeneity in estimates at any given threshold. The NMA-DTA incorporating continuous measures enables us to estimate accuracy at any threshold: for example, AFP_20ng/mL_ sensitivity=0.60 (95% CrI 0.56 to 0.64), specificity=0.89 (95% CrI 0.86 to 0.91), whereas AFP_200ng/mL_ sensitivity=0.36 (95% CrI 0.30 to 0.41), specificity=0.99 (95% CrI 0.99 to 0.99).

For imaging tests, online supplemental figure S13.2 in online supplemental appendix S13 shows results in ROC space. Cross-sectional and/or contrast-enhanced techniques have similar sensitivity (summary estimates 0.89–0.94). US has lower sensitivity, though with substantial between-study heterogeneity. CEUS and non-contrast MRI have highest summary specificity (both 0.98) followed by US (0.94 (95% CrI 0.90 to 0.97)).

Six genomic targets appear in multiple studies, of which mSEPT9 (a circulating, cell-free DNA marker) has highest sensitivity (0.89 (95% CrI 0.75 to 0.96)), similar to the disjunctive combination of all three conventional biomarkers (AFP_·OR·_AFP-L3_·OR·_DCP (mAU/mL)—0.88 (95% CrI 0.70 to 0.96)).

#### Meta-analysis of each test separately

Online supplemental appendix S12 compares NMA-DTA results with estimates from sensitivity analyses in which we meta-analyse each test separately. Differences are more obvious for sensitivity than for specificity, particularly for multitest algorithms: GALAD, Doylestown and the HCC early detection screening algorithm all have sensitivity at least 0.15 higher in NMA-DTA than when we meta-analyse each in isolation.

#### Variability in sensitivity by tumour stage

Table 2 gives stage-stratified estimates of the sensitivity of tests for which we have sufficient data. Among continuous tests, there were sufficient data for a multiple-threshold analysis only for AFP. Online supplemental figure S13.3 in online supplemental appendix S13 shows pooled estimates of sensitivity for the three different stages across the whole range of observed thresholds. The model is able to distinguish clearly between advanced (BCLC_B/C/D_) and earlier stages (BCLC_0_ and BCLC_A_). We do not have enough empirical data to include AFP_·OR·_US in our stage-stratified analysis but, assuming independence, AFP_20ng/mL·OR·_US would have sensitivity of 0.60 (95% CrI 0.48 to 0.73) for very early HCC, 0.80 (95% CrI 0.66 to 0.89) for early HCC and 0.94 (95% CrI 0.87 to 0.98) for advanced HCC.

**Table 2:** Estimated sensitivity of tests to detect HCCs of different stages

	Very early stage (BCLC_0_)		Early stage (BCLC_A_)		Advanced stage (BCLC_B/C/D_)	
	Est.	95% CrI	Est.	95% CrI	Est.	95% CrI
AFP at threshold 20 ng/mL	0.39	(0.28 to 0.47)	0.46	(0.37 to 0.57)	0.72	(0.64 to 0.79)
AFP-L3 at threshold 10%	0.26	(0.09 to 0.52)	0.47	(0.16 to 0.83)	0.59	(0.35 to 0.78)
B-mode ultrasound	0.34	(0.21 to 0.55)	0.62	(0.40 to 0.80)	0.80	(0.55 to 0.94)
Contrast-enhanced MRI	0.70	(0.50 to 0.84)	0.86	(0.73 to 0.94)	0.90	(0.67 to 0.98)
Multiphase CT	0.68	(0.26 to 0.93)	0.77	(0.29 to 0.95)	0.92	(0.49 to 0.99)

Note that specificity has no relation to HCC stage, as no one in the relevant calculations has HCC. Specificity for each test is shown in figure 2*.*

AFP, alpha-fetoprotein; AFP-L3, lens culinaris agglutinin-reactive fraction of AFP; BCLC, Barcelona Clinic Liver Cancer; HCC, hepatocellular carcinoma.

#### Exploration of heterogeneity

Online supplemental appendix S14.1 gives results from sensitivity analyses restricted to one-gate studies and those at low risk of bias in the reference standard domain of QUADAS-2. Results appear similar, suggesting it is unlikely that there is a systematic relationship between methodological characteristics and accuracy estimates.

Meta-regression suggests that sensitivity of AFP is highest, but specificity lowest, in studies in which a plurality of participants had HCV-related cirrhosis (online supplemental appendix S14.2). There is no evidence of variation in test accuracy by predominant aetiology for the other tests. We found little evidence of accuracy varying by sex or average age of participants (online supplemental appendix S14.3). One exception is multiphase CT, where estimated sensitivity increases with age; however, relatively few studies inform this result. There is also weak evidence of lower sensitivity of AFP in studies with a higher proportion of participants with Child-Pugh score A. We found no other evidence of cirrhosis severity affecting accuracy (online supplemental appendix S14.3).

## Discussion

### Principal findings

Strikingly, we find that the most used tests for HCC surveillance lack sensitivity to identify tumours that are amenable to the most effective treatments. US, which forms the bedrock of surveillance programmes worldwide, is among the most specific tests, meaning it raises relatively few false alarms. However, we estimate that any individual scan will miss two-thirds of very-early-stage HCCs and two-fifths of early-stage HCCs. The periodic application of tests will result in better long-term sensitivity, though the window of opportunity is likely to be narrow, owing to unseen tumour progression.

Cross-sectional imaging has higher absolute sensitivity, but between-stage differences remain clear. Barriers to the use of multiphase CT and MRI as first-line surveillance tests include cost and exposure to radiation and/or contrast media; some authors propose that abbreviated protocols may reduce these problems.^9^ Because ‘abbreviated MRI’ has a variety of definitions, we simply categorise studies according to whether they have a contrast phase. Our analysis estimates that non-contrast MRI has comparable sensitivity to contrast-enhanced MRI for detecting HCC of any stage, and as-good-if-not-better specificity. However, owing to the small amount of published and included evidence, CrIs for non-contrast approaches are broad, and we cannot exclude important differences.

Eight studies explicitly provide evidence on a strategy combining AFP_20ng/mL·OR·_US, suggesting good overall accuracy (in stage-agnostic NMA-DTA, sensitivity=0.90 (95% CrI 0.79 to 0.96); specificity=0.89 (95% CrI 0.77 to 0.95)). It is reassuring that these estimates are consistent with combining estimates from the much fuller evidence-base describing each test in isolation: assuming independence, sensitivity=0.88 (1−(1−0.60 for AFP_20ng/mL_)×(1−0.70 for US)) and specificity=0.85 (0.90 for AFP_20ng/mL_×0.94 for US). Stage-specific analysis of AFP_20ng/mL_ and US individually implies that AFP_·OR·_US should detect around three-fifths of very-early-stage HCCs and four-fifths of early-stage HCCs. However, expected sensitivity could be somewhat lower if the two tests are not truly independent.

Our finding that the diagnostic accuracy of AFP for the detection of HCC varies according to aetiology of cirrhosis is not, in itself, novel,^39^ but explains some of the between-study heterogeneity we observe. Most obviously, as people with HCV are likely to have higher AFP, the test will be more sensitive and less specific, at any given threshold, than in people with other causes of liver disease. Exposure to direct-acting antiviral medicines may alter these dynamics, perhaps mandating a lower threshold in people with sustained virological response,^40^ though we found no evidence directly addressing this population.

### Strengths and limitations

This is by far the most extensive evidence synthesis of tests that might be used in surveillance for HCC, generating coherent pooled estimates of the accuracy of 97 different options. NMA-DTA exploits evidence showing how tests compare with each other, strengthening inference about continuous biomarkers by modelling all thresholds jointly, exploring heterogeneity using meta-regression and estimating the sensitivity of tests to detect HCCs at different stages.

The NMA-DTA model we fitted implicitly makes the assumption that the HCCs in each individual study have a greater or lesser level of detectability that influences all tests. This explains why the approach leads to some results that might appear counterintuitive at first glance. For example, we estimate sensitivity of non-contrast MRI at 0.92 (95% CrI 0.75 to 0.97), noticeably higher than either of the two studies that report it. This difference arises because, in the larger of the two studies,^41^ non-contrast MRI’s sensitivity (0.79) is impressive compared with the poor sensitivity of other tests (AFP_20ng/mL_=0.12; US=0.28). Because NMA-DTA takes this relationship into account, it estimates that, for averagely detectable HCCs (where, other evidence tells us, AFP and US will have much better sensitivity), non-contrast MRI will also perform better. If we look at the study in question, we see that it includes an unusually high proportion of early-stage HCCs (67% BCLC_0_; 31% BCLC_A_; only one more advanced case).^41^ This shows why such findings are ‘a feature, not a bug’ of our approach. A similar phenomenon is at play in our multiple-thresholds analysis of GALAD. In figure 3, it may appear that the fitted model for sensitivity is optimistic compared with the data. However, the model ‘knows’ that AFP_20ng/mL_ alone performed poorly in four of the seven studies evaluating GALAD, with sensitivity <0.30 (compared with our pooled estimate of 0.60),^3742^,^44^ and this has an important influence on the estimated sensitivity of GALAD. As above, early-stage HCCs are unusually prevalent in these studies. We do not configure the model to reflect that AFP is a component of GALAD, but it detects the correlation between the two.

We identify meaningful threats to the internal validity of almost all included evidence. QUADAS-2 effectively classifies all two-gate studies—which predominate here—as at high risk of bias in the ‘patient selection’ domain. It is also difficult to guarantee that such studies’ investigators assessed index test(s) without knowledge of the reference standard. However, when we restricted our analysis to studies at comparatively lower risk of bias, our syntheses produced closely comparable results to those performed in the full dataset.

We imposed a strict inclusion criterion that studies exclusively recruit people with cirrhosis. This has the advantage of limiting our evidence-base to a relatively homogeneous population. However, we recognise that surveillance may be indicated in some people without cirrhosis; readers should be cautious about generalising our results to such populations. Even in our constrained cirrhosis population, potentially important differences exist between study cohorts. We explored age, sex, aetiology and degree of underlying liver dysfunction in meta-regression, without finding any compelling evidence of associations with accuracy, other than where HCV predominates (see above). The way in which studies identify their cohorts may also have an influence. In particular, populations undergoing liver transplantation for indications other than HCC are conspicuously different from those recruited in surveillance programmes. On the one hand, explant pathology provides the most accurate possible reference standard against which to assess tests—indeed, it is the only way to ascribe true-negative status with confidence. On the other, people with end-stage decompensation are not directly representative of the surveillance population, and it is possible that spectrum effects compromise generalisability from one setting to another.

Ultimately, accurate diagnosis of HCC is only part of the mechanism by which surveillance may lead to improved patient outcomes. Because randomised trials of surveillance for HCC are unfeasible,^45^ no single study will ever conclusively evaluate impact on mortality and other important patient outcomes. In this context, robust estimates of diagnostic accuracy become a critical link in the chain of evidence through which we can assess the effectiveness of surveillance. This review is part of a wider project, including comprehensive decision-modelling, in which we use our test accuracy estimates, among other inputs, to simulate the benefits, harms and costs of an extensive range of surveillance strategies.

### Comparison with other published reviews

The Cochrane review on which we relied in identifying pre-2020 evidence for AFP and US^11^ provides pooled results that are comparable with ours, although specificity of AFP_20ng/mL_ is somewhat lower (0.84 (95% CI 0.82 to 0.86) compared with our 0.90 (95% CrI 0.88 to 0.92)). We note that the Cochrane review includes pre-2005 evidence, while we include studies that postdate their review. Possible reasons for specificity improving over time are increasingly accurate imaging-based reference standards (converting false positives into true positives) and the widespread use of effective antivirals for chronic HBV and HCV infections, making false-positive elevation of AFP less likely.

We are aware of one other NMA-DTA in this area, which finds that the ‘multitarget blood test’ has comparable sensitivity to AFP_·OR·_US, but better than either test in isolation.^12^ Although we include the single study reporting accuracy of this test, we are unable to include it in our connected synthesis network as, while the study assessed other tests of interest (AFP, GALAD), it only reports cirrhosis-specific results for the multitarget blood test.^46^

There are multiple meta-analyses synthesising data on the diagnostic accuracy of cross-sectional and/or contrast-enhanced imaging.^7^,^10^ However, none is limited to people with cirrhosis, and none adopts the eligibility criterion that participants should have no particular suspicion of HCC at baseline. We consider that the studies included in these reviews—which mostly describe delineation of lesions first discovered on US—may present a biased estimate of tools’ accuracy in the context of first-line surveillance.

## Conclusions

The optimal approach to surveillance for HCC in people with cirrhosis is a complex function of multiple factors, but the balance will always pivot on the ability of investigations to detect early-stage cancers. Commonly used tests may miss a significant proportion of these HCCs, although repeated use in surveillance will mitigate insensitivity to some degree. From the perspective of someone undergoing—or considering whether to undergo—surveillance, any sensitivity >0 represents a chance of finding a potentially lethal lesion that may be amenable to curative treatment. However, people’s preferences will vary when it comes to weighing this benefit against the chance of false alarms, as well as the inconvenience, expense and direct harms with which surveillance may also be associated. We identified a considerable volume of evidence, but it is thinly spread across a variety of possible diagnostic tools, and high risk of bias is overwhelmingly prevalent. Although our methods allow us to make the best of this unsatisfactory situation, we need more one-gate studies with consistent reference standards—allied to a convincing pathway delivering long-term benefits of early-stage detection—if we are to reach more confident conclusions.

## Supplementary material

10.1136/bmjgast-2025-002155 online supplemental file 1

## Data Availability

All data relevant to the study are included in the article or uploaded as supplementary information.
