# Supplementary material for: Diagnostic accuracy of surveillance tests for hepatocellular carcinoma in cirrhosis: a systematic review and network meta-analysis
Source: BMJ Open Gastroenterol. 2026 Jul 17;13(1):e002155. doi: 10.1136/bmjgast-2025-002155 (PMC13384173; doi:10.1136/bmjgast-2025-002155)
Supplement: online supplemental file 1 [file bmjgast-13-1-s001.pdf]

Gabriel Rogers, Efthymia Derezea, Libby Sadler, Hanyu Wang, Kelsey Watt, Stephen Ryder, Matthew Cramp, Penny Whiting, Morwenna Rogers, John Bell, Felicity Oppe, Nicky J Welton, Hayley E Jones

# Diagnostic accuracy of tests used in surveillance for hepatocellular carcinoma in people with cirrhosis: systematic review and network meta-analysis

*BMJ Open Gastroenterol 2026*

http://dx.doi.org/10.1136/bmjgast-2025-002155

## Supplementary material

| | | |
|---|---|---|
| Appendix S1 | PRISMA-DTA checklist | 2 |
| Appendix S2 | Deviations from review protocol | 5 |
| Appendix S3 | Potentially relevant non-English studies with English abstracts | 6 |
| Appendix S4 | Search strategies | 33 |
| Appendix S5 | Detailed methods for quantitative synthesis | 39 |
| Appendix S6 | PRISMA flow diagram | 43 |
| Appendix S7 | Excluded studies | 44 |
| Appendix S8 | Characteristics of included studies | 201 |
| Appendix S9 | Risk of bias assessment of included studies (QUADAS-2) | 255 |
| Appendix S10 | Tests not included in the common synthesis network | 260 |
| Appendix S11 | Characteristics of tests included in NMA-DTA analysis results | 261 |
| Appendix S12 | Network meta-analysis and meta-analysis of the accuracy of each test separately | 266 |
| Appendix S13 | Network meta-analysis of diagnostic test accuracy results | 274 |
| Appendix S14 | Exploration of heterogeneity | 278 |
| Appendix S15 | Forest plots of tests reported in 2 or more studies | 291 |
| Appendix S16 | Summary graphic | 310 |
| References for appendices | | 312 |

## Appendix S1 PRISMA-DTA checklist

| Section/topic | # | PRISMA-DTA Checklist Item | Reported on page # |
|---|---|---|---|
| **TITLE / ABSTRACT** | | | |
| Title | 1 | Identify the report as a systematic review (+/- meta-analysis) of diagnostic test accuracy (DTA) studies. | 1 |
| Abstract | A1 | Identify the report as a systematic review (+/– meta-analysis) of DTA studies | 1 |
| | A2 | Indicate the research question, including components such as participants, index test, and target conditions | 2 |
| | A3 | Include study characteristics used as criteria for eligibility | 2 |
| | A4 | List the key databases searched and the search dates | 2 |
| | A5 | Indicate the methods of assessing risk of bias and applicability | 2 |
| | AA1 | Indicate the methods for the data synthesis | 2 |
| | A6 | Indicate the number and type of included studies and the participants and relevant characteristics of the studies (including the reference standard) | 2 |
| | A7 | Include the results for the analysis of diagnostic accuracy, preferably indicating the number of studies and participants. Describe test accuracy including variability; if meta-analysis was done, include summary results and confidence intervals | 2 |
| | A9 | Provide a brief summary of the strengths and limitations of the evidence | 2 |
| | A10 | Provide a general interpretation of the results and the important implications | 2 |
| | A11 | Indicate the primary source of funding for the review | 2 |
| | A12 | Provide the registration number and the registry name | 2 |
| **INTRODUCTION** | | | |
| Rationale | 3 | Describe the rationale for the review in the context of what is already known. | 5 |
| Clinical role of index test | D1 | State the scientific and clinical background, including the intended use and clinical role of the index test, and if applicable, the rationale for minimally acceptable test accuracy (or minimum difference in accuracy for comparative design). | 5 |
| Objectives | 4 | Provide an explicit statement of question(s) being addressed in terms of participants, index test(s), and target condition(s). | 6 |
| **METHODS** | | | |
| Protocol and registration | 5 | Indicate if a review protocol exists, if and where it can be accessed (e.g., Web address), and, if available, provide registration information including registration number. | 7 |
| Eligibility criteria | 6 | Specify study characteristics (participants, setting, index test(s), reference standard(s), target condition(s), and study design) and report characteristics (e.g., years considered, language, publication status) used as criteria for eligibility, giving rationale. | 7 |

| Section/topic | # | PRISMA-DTA Checklist Item | Reported on page # |
|---|---|---|---|
| Information sources | 7 | Describe all information sources (e.g., databases with dates of coverage, contact with study authors to identify additional studies) in the search and date last searched. | 8 |
| Search | 8 | Present full search strategies for all electronic databases and other sources searched, including any limits used, such that they could be repeated. | 8, Appendix S4 |
| Study selection | 9 | State the process for selecting studies (i.e., screening, eligibility, included in systematic review, and, if applicable, included in the meta-analysis). | 8 |
| Data collection process | 10 | Describe method of data extraction from reports (e.g., piloted forms, independently, in duplicate) and any processes for obtaining and confirming data from investigators. | 8 |
| Definitions for data extraction | 11 | Provide definitions used in data extraction and classifications of target condition(s), index test(s), reference standard(s) and other characteristics (e.g. study design, clinical setting). | 8 |
| Risk of bias and applicability | 12 | Describe methods used for assessing risk of bias in individual studies and concerns regarding the applicability to the review question. | 8 |
| Diagnostic accuracy measures | 13 | State the principal diagnostic accuracy measure(s) reported (e.g. sensitivity, specificity) and state the unit of assessment (e.g. per-patient, per-lesion). | 8 |
| Synthesis of results | 14 | Describe methods of handling data, combining results of studies and describing variability between studies. This could include, but is not limited to: a) handling of multiple definitions of target condition. b) handling of multiple thresholds of test positivity, c) handling multiple index test readers, d) handling of indeterminate test results, e) grouping and comparing tests, f) handling of different reference standards | 9, Appendix S5 |
| Meta-analysis | D2 | Report the statistical methods used for meta-analyses, if performed. | 9 |
| Additional analyses | 16 | Describe methods of additional analyses (e.g., sensitivity or subgroup analyses, meta-regression), if done, indicating which were pre-specified. | 10, Appendix S5 |
| **RESULTS** | | | |
| Study selection | 17 | Provide numbers of studies screened, assessed for eligibility, included in the review (and included in meta-analysis, if applicable) with reasons for exclusions at each stage, ideally with a flow diagram. | Appendix S6 |
| Study characteristics | 18 | For each included study provide citations and present key characteristics including: a) participant characteristics (presentation, prior testing), b) clinical setting, c) study design, d) target condition definition, e) index test, f) reference standard, g) sample size, h) funding sources | 25, Appendix S8 |
| Risk of bias and applicability | 19 | Present evaluation of risk of bias and concerns regarding applicability for each study. | 11, Appendix S9 |
| Results of individual studies | 20 | For each analysis in each study (e.g. unique combination of index test, reference standard, and positivity threshold) report 2x2 data (TP, FP, FN, TN) with estimates of diagnostic accuracy and confidence intervals, ideally with a forest or receiver operator characteristic (ROC) plot. | Appendix S15, Appendix S10 |
| Synthesis of results | 21 | Describe test accuracy, including variability; if meta-analysis was done, include results and confidence intervals. | 12, Appendix S12, Appendix |

| Section/topic | # | PRISMA-DTA Checklist Item | Reported on page # |
|---|---|---|---|
| | | | S13, Appendix S15 |
| Additional analysis | 23 | Give results of additional analyses, if done (e.g., sensitivity or subgroup analyses, meta-regression; analysis of index test: failure rates, proportion of inconclusive results, adverse events). | 13, Appendix S14 |
| **DISCUSSION** | | | |
| Summary of evidence | 24 | Summarise the main findings including the strength of evidence. | 14 |
| Limitations | 25 | Discuss limitations from included studies (e.g. risk of bias and concerns regarding applicability) and from the review process (e.g. incomplete retrieval of identified research). | 15 |
| Conclusions | 26 | Provide a general interpretation of the results in the context of other evidence. Discuss implications for future research and clinical practice (e.g. the intended use and clinical role of the index test). | 16 |
| **FUNDING** | | | |
| Funding | 27 | For the systematic review, describe the sources of funding and other support and the role of the funders. | 18 |

*Adapted from:*
*McInnes MDF, Moher D, Thombs BD, McGrath TA, Bossuyt PM, The PRISMA-DTA Group (2018). Preferred Reporting Items for a Systematic Review and Meta-analysis of Diagnostic Test Accuracy Studies: The PRISMA-DTA Statement. JAMA. 2018 Jan 23;319(4):388-396. https://doi.org/10.1001/jama.2017.19163.*
*and:*
*Cohen JF, Deeks JJ, Hooft L, Salameh JP, Korevaar DA, Gatsonis C, Hopewell S, Hunt HA, Hyde CJ, Leeflang MM, Macaskill P. Preferred reporting items for journal and conference abstracts of systematic reviews and meta-analyses of diagnostic test accuracy studies (PRISMA-DTA for Abstracts): checklist, explanation, and elaboration. BMJ. 2021 Mar 15;372. https://doi.org/10.1136/bmj.n265*

## Appendix S2 Deviations from review protocol

Excluding studies published before 2005 was a post-hoc decision. We adopted this criterion to restrict the included evidence to that which is most relevant to present-day practice. In particular, we judge that imaging technology has progressed to a degree that renders older studies obsolescent – a bias that affects all index tests, as studies commonly use imaging as part of their reference standard, even for index tests that have not changed much over time. We chose 2005 as our cutoff, as a previous review gives good detail on the preexisting standard of care, derived from studies published before this date[1].

We were unable to perform full double assessment of full-text papers, as planned, as we found more potentially relevant studies than anticipated (almost 2,500). We double-screened full text of all studies identified from the Cochrane review of AFP and US, and all studies that we ultimately included also received full-text scrutiny from at least 2 independent researchers. So we are confident that there are no false-positive inclusions in our dataset. However, we acknowledge that only a proportion of excluded studies received double full-text assessment, so we cannot exclude the possibility that there are false-negative exclusions among them.

We planned to extract and analyze full cross-classified data for studies reporting multiple tests (e.g. 2×2×2 table for two index tests). We performed extraction as planned; however, we found that very few studies reported data in a format that would enable us to perform analysis, so we did not make a special case for the very few studies that provided data in this format.

In our protocol, we undertook to 'extract accuracy measures e.g. sensitivity or specificity to check these are consistent with extracted cross-classified test results'. In practice, our database did not make it possible to perform formal extraction of summary measures; however, we checked all 2×2 data against reported summary statistics and noted any discrepancies we observed.

## Appendix S3 Potentially relevant non-English studies with English abstracts

| Ref | Abstract |
|---|---|
| Bai, X. F. & Wang, P. Y. Value of combined determination of tumor markers based on two discriminative models in facilitating diagnosis of hepatic carcinoma. Medical Journal of Chinese People's Liberation Army 2012;37(11):849-52. | Objective To explore the value of determination of combined tumor markers based on artificial neural network (ANN) discrimination model in facilitating the diagnosis of hepatic carcinoma. Methods Serum samples were collected from three groups of subjects, including 50 cases of liver cancer, 40 cases of benign liver disease, and 50 normal controls. The levels of serum alpha fetoprotein (AFP), carbohydrate antigen 125 (CA125) and carcino-embryonic antigen (CEA) were determined by chemiluminescence immunoassay. The level of serum sialic acid (SA) was determined by spectrophotometry, the content of calcium in serum was measured by calcium assay kit (Azo-end method of arsenic HI). Based on the five tumor markers mentioned above as discrimination variables, Fisher discrimination and ANN were applied to set up the intelligent auxiliary diagnostic model. Results By applying the Fisher discrimination model established in present work, the diagnostic sensitivity of liver cancer was 46.1%, the specificity was 98.9%, the accurate rate was 79.3%, the positive predictive value was 95.8%, and the negative predictive value was 76.7% for the three groups. With the application of ANN discrimination model, the diagnostic sensitivity of liver cancer was raised to 96.0%, the specificity 98.9%, the accuracy 94.3%, the positive predictive value 98.0%, and the negative predictive value was 97.8%. Conclusion The diagnostic model based on ANN combined with 5 tumor markers is superior in diagnostic acuity to traditional Fisher discrimination analysis, thus more suitable for clinical data analysis. |
| Barletta, Emiddio & Tinessa, Vincenza. [Screening of hepatocellular carcinoma: role of the alpha-fetoprotein (AFP) and ultrasonography]. Lo screening del carcinoma epatocellulare. 2005;96(6):295-328. | Even if there are no definitive evidence that hepatocellular carcinoma (HCC) screening in high-risk groups improves survival, many physicians screen high-risk population by various strategies, alpha-fetoprotein (AFP) and liver ultrasonography are the most widely used tools. AFP sensitivity and specificity depend on the cut-off value chosen. In cirrhotic patients, using a cut-off level of 20 ng/mL, sensitivity is only around 60% and positive predictive value ranges from 9% to 50%, depending on HCC prevalence. Sensitivity and specificity are much higher (94.1% and 99.9%, respectively) in hepatitis B carriers, but positive predictive value is only 5%. The performance of ultrasonography as a screening tool varies widely depending on the experience of the examiner and the technology used. Recent studies generally indicate a 60% sensitivity or higher, a specificity greater than 90%, and a positive predictive value of 70%. Based on the estimated HCC doubling time, the recommended screening interval is 6 months, although a 1 year interval seems as effective. Currently, HCC screening with AFP only is not recommended except when ultrasonography is either not available or of poor quality. Ultrasonography seems more efficient as a screening tool. Pathology assessment of liver explants in living donor transplantation programs will provide more precise and reliable information regarding the value of AFP and ultrasonography as HCC screening tools. |
| Ben Hassine, L., Daghfous, M. H., Mami, A., Chammakhi-Jemli, C., Zouaoui, W., Saddoud, W., et al. [Imaging in the screening and diagnosis of hepatocellular carcinoma in cirrhosis liver in Tunisia. A series of 30 cases]. L'imagerie dans le depistage et le diagnostic du carcinome hepatocellulaire sur foie de cirrhose en Tunisie. A propos de 30 cas. 2007;85(5):421-26. | UNLABELLED: The aim of our study is to expose a practical screening and diagnosis strategy of HCC occuring in cirrhosis liver, which is nowadays a health public matter in Tunisia., METHODS: HCC routine screening in cirrhosis liver has been conducted every 6 months using a combination of abdominal sonography and serum alpha-foetoprotein levels. PCUS, CT scan or MRI have been performed each time a focal liver lesion was discovered in US, or in case of elevated serum alpha-foetoprotein levels. 30 cases of HCC were identified., RESULTS: US has shown a unique nodule in 46% of the cases, multiple nodules in 50% of them. No lesion has been discovered in 4% of the cases. Nodule size ranged from 8 to 140 mm. Heterogenous hyperechoic pattern was the most frequent one (41%). Hypervascular doppler feature was authentified in 20% of the cases. Serum alpha-foetoprotein levels had respectively a sensitivity and a specificity of 14% and 44% in lesions inferior to 3 cm, 62% and 80% in lesions superior to 3 cm. Typical arterial enhancement of HCC was depicted in 1 case for PCUS, in 76% for CT scan and 60% for MRI., CONCLUSION: Combination of abdominal sonography and serum alpha-foetoprotein levels practiced every 6 months represent a good compromise between cost and effectiveness. Second intention imaging is based on CT scan in Tunisia because of its availability compared to MRI. PCUS is becoming in the near future the best modality for lesion caracterization thanks to its safety and relative inexpensiveness. |
| Ben, Q., Ni, R., Xiao, M., Lu, C., Li, L. The value of HS-AFP in the early diagnosis and differential diagnosis of hepatocellular carcinoma. Chinese Journal of Clinical Oncology 2008;35(15):845-47. | Objective: To determine the value of hepatoma-specific alpha-fetoprotein (HS-AFP) in the early diagnosis and differential diagnosis of hepatocellular carcinoma (HCC). Method(s): HS-AFP and AFP were measured in 105 cases with HCC and 151 cases with benign liver diseases. There were 10 cases of benign liver diseases with positive HS-AFP that were followed up for 15 months. Total protein was separated with polyacrylamide gel electrophoresis (PAGE) in a discontinuous buffer system and HS-AFP was detected by Western blot. AFP was detected by chemiluminescence. Result(s): In the 105 cases of HCC, the positive rates of HS-AFP and AFP>200 mug/L were 60.0% and 50.5%, respectively (P>0.05). In the cases of benign liver diseases, the positive rate of HS-AFP was significantly lower (P<0.05). In the cases with AFP of 50mug/L to 400mug/L, the positive rate of HS-AFP was 77.1% in HCC and 13.6% in benign liver diseases. Of the 10 cases of benign liver diseases with positive HS-AFP, 3 of them developed HCC during the follow-up period. In the 11 cases with small HCC, the positive rate of HS-AFP (45.5%) was significantly higher than that of AFP (18.2%). Conclusion(s): HS-AFP was a better marker than AFP in the diagnosis of HCC. Monitoring HS-AFP in a high risk population was useful for early diagnosis of HCC. HS-AFP plays an important role in the differential diagnosis between HCC and benign liver diseases in patients with slight to moderate elevation of serum AFP. |

| Ref | Abstract |
|---|---|
| Best, J., Bilgi, H., Heider, D., Schotten, C., Manka, P., Bedreli, S., et al. The GALAD scoring algorithm based on AFP, AFP-L3, and DCP significantly improves detection of BCLC early stage hepatocellular carcinoma. Der GALAD-Score, ein AFP-, AFP-L3- und DCP-basierter Diagnosealgorithmus verbessert die Detektionsrate des hepatozellularen Karzinoms im BCLC-Fruhstadium signifikant. 2016;54(12):1296-3005. | Background: Hepatocellular carcinoma (HCC) is one of the leading causes of death in cirrhotic patients worldwide. The detection rate for early stage HCC remains low despite screening programs. Thus, the majority of HCC cases are detected at advanced tumor stages with limited treatment options. To facilitate earlier diagnosis, this study aims to validate the added benefit of the combination of AFP, the novel biomarkers AFP-L3, DCP, and an associated novel diagnostic algorithm called GALAD. Material and methods: Between 2007 and 2008 and from 2010 to 2012, 285 patients newly diagnosed with HCC and 402 control patients suffering from chronic liver disease were enrolled. AFP, AFP-L3, and DCP were measured using the microTASWako i30 automated immunoanalyzer. The diagnostic performance of biomarkers was measured as single parameters and in a logistic regression model. Furthermore, a diagnostic algorithm (GALAD) based on gender, age, and the biomarkers mentioned above was validated. Results: AFP, AFP-L3, and DCP showed comparable sensitivities and specifities for HCC detection. The combination of all biomarkers had the highest sensitivity with decreased specificity. In contrast, utilization of the biomarker-based GALAD score resulted in a superior specificity of 93.3 % and sensitivity of 85.6 %. In the scenario of BCLC 0/A stage HCC, the GALAD algorithm provided the highest overall AUROC with 0.9242, which was superior to any other marker combination. Conclusions: We could demonstrate in our cohort the superior detection of early stage HCC with the combined use of the respective biomarkers and in particular GALAD even in AFP-negative tumors. Copyright Â© Georg Thieme Verlag KG Stuttgart . New York. |
| Blondin, D., Erhardt, A., Crynen, K., Sagir, A., Scherer, A., Kropil, P., et al. [Diagnosis of focal liver lesions in cirrhotic patients: comparison of contrast-enhanced ultrasound using sulphur hexafluoride (SF6) microbubbles and MRI using Gd-EOB-DTPA]. Vergleich der kontrastverstarkten Sonografie und der MRT mit Gd-EOB-DTPA zur Diagnostik fokaler Leberlasionen bei Patienten mit Leberzirrhose. 2011;49(1):23-29. | AIM: The diagnostic accuracies of contrast-enhanced sonography and hepatobiliary contrast-enhanced MRI of the liver in evaluating focal liver lesions in patients with liver cirrhosis were compared., MATERIAL AND METHODS: In 33 patients (25 men, 8 women, mean age 63.2 +/- 11.2 years) MRI of the liver using Gd-EOB-DTPA (Primovist R, Bayer Schering Pharma, Berlin) was performed. Axial T(2)-weighted, unenhanced T(1)-weighted and enhanced T(1)-weighted scans during arterial, portal venous and late phases were acquired, followed by coronary T(1)-weighted and axial fat-suppressed T(1)-weighted scans 15 minutes post contrast application. In all patients within 4 weeks contrast-enhanced sonography using sulfur hexafluoride microbubbles (SonoVue R, Nycomed, Germany) was obtained., RESULTS: Cirrhosis of the liver was related to viral infection in 45.4% and to alcoholism in 39.4%. All hepatic lesions were confirmed by histologic examination. Sensitivity and specificity of MRI were 90.2% and 83.3%, compared to contrast-enhanced sonography with 92.7 % and 50 %, respectively. Positive and negative predictive values were 97.4% and 55.5 % for MRI and 90.5% and 50% for contrast-enhanced sonography, respectively., DISCUSSION: In this retrospective study MRI using Gd-EOB-DTPA as well as contrast-enhanced sonography using sulfur hexafluoride microbubbles gave excellent results in detecting HCC in patients suffering from liver cirrhosis. Although the specificity was higher for MRI, the accuracy showed no significant difference between these two imaging techniques. Copyright Â© Georg Thieme Verlag KG Stuttgart . New York. |
| Brudermanns, B. Hepatocellular carcinoma - Double contrast MRI at 3.0 T shows high sensitivity. RoFo Fortschritte auf dem Gebiet der Rontgenstrahlen und der Bildgebenden Verfahren 2009;181(1):8. | |
| Chen, H. B. & Gu, Y. M. The expression level of serum Golgi protein-73 in primary hepatocellular carcinoma and its clinical value in making early diagnosis. Journal of Interventional Radiology (China) 2014;23(6):528-31. | Objective: To investigate the expression level of serum Golgi protein-73 (GP73) in patients with primary hepatocellular carcinoma (HCC), or accompanied with Budd-Chiari syndrome (BCS), and to discuss its clinical value in making early diagnosis. Method(s): The peripheral serum samples were collected in 490 individuals, including patients with HCC (n = 65), cholangiocarcinoma (CC, n = 35), Budd-Chiari syndrome accompanied with HCC (n = 40), type B virus hepatitis (n = 60), cirrhosis after B hepatitis (n = 60), Budd-Chiari syndrome complicated by cirrhosis (n = 60), other malignant lesions (n = 50), benign hepatic lesions (n = 60) and healthy volunteers (n = 60). The expression levels of serum GP73 and AFP were determined by using Elisa and electrochemiluminescence methods separately. Result(s): The expression level of serum GP73 in the patients with HCC, CC or BCS plus HCC were significantly higher than that in the other individuals ($P < 0.05$). The area under the receiver operating characteristic curve (ROC), the sensitivity, the specificity and the diagnostic accuracy of GP73 and AFP for HCC were 0.868, 90.8%, 75.5%, 77.6% and 0.739, 60%, 83.2%, 80.2% respectively. The cut-off values of GP73 and AFP were 43.40 ng/ml and 20ng/ml respectively. The area under ROC and the sensitivity of GP73 for the diagnosis of HCC were strikingly higher than those of AFP ($P < 0.05$), while no significant differences in the specificity and diagnostic accuracy existed between GP73 and AFP ($P > 0.05$). The area under ROC, sensitivity, specificity and the accuracy of GP73 for the diagnosis of CC were 0.774, 88.6%, 72.7% and 73.9% respectively, and the optimal cut-off value was 45.40 ng/ml. Conclusion(s): In diagnosing HCC, the expression level of serum GP73 is superior to AFP. It is hopeful that serum GP73 may be proved to be an early serum tumor marker for HCC. |

| Ref | Abstract |
|---|---|
| Chen, L., Wang, F., Ju, L. L. Clinical application of serum miR-493-5p detection in patients with hepatocellular carcinoma. Chinese Journal of Cancer Prevention and Treatment 2019;26(5):306-10. | OBJECTIVE: MicroRNAs(miRNAs) play important roles in the occurrence and development of hepatocellular carcinoma (HCC).Previous studies have found that miR-493-5p is downregulated in HCC tissues,but its expression and significance in the peripheral blood circulation of HCC patients have not been reported.This study aimed to investigate the clinical application value of serum miR-493-5p as a biomarker for HCC. METHOD(S): Quantitative real time polymerase chain reaction(qRT-PCR) was used to assess the level of serum miR-493-5p in 52 HCC patients,which were enrolled from January 10,2014 to December 20,2016 in Third people's Hospital of Nantong City.ROC curve,survival curve and the relationship between miR-493-5p and clinical pathological parameters were analyzed.As controls,the serum levels of miR-493-5p were also measured in 45 patients with benign liver diseases and 40 healthy subjects.RESUITS The serum miR-493-5p levels in HCC patients (1.078+/-0.097) were lower than those in benign liver disease group (1.413+/-0.110) and healthy control group (1.613+/-0.139,F=5.745,P=0.004).The ROC curve showed that the area under the curve of serum miR-493-5p used for diagnosis of HCC was 0.676 and the 95% confidence interval was 0.570-0.770; the diagnostic sensitivity was 71.15%,and the specificity was 55.29%.The sensitivity and negative predictive value of combined detection of serum miR-493-5p and AFP for HCC diagnosis were superior to those of single detection.Serum miR-493-5p was associated with TNM stage in HCC patients(chi2=7.528,P=0.006),but not significantly correlated with other clinical parameters (all P>0.05).In addition,the level of serum miR-493-5p in postoperative HCC patients (1.815+/-0.176) was significantly higher than that of preoperative (1.274+/-0.151; t=2.330,P=0.025),and the serum miR-493-5p level in patients with recurrent or distant metastasis (1.092+/-0.205) was significantly lower than that of postoperative (1.815+/-0.176; t=2.557,P=0.015); there was no significant difference between the groups of recurrent or distant metastasis (1.092+/-0.205) and preoperative (1.274+/-0.151; t=0.713,P=0.486).Survival curve analysis showed that the overall survival rate of HCC patients with low serum miR-493-5p level was significantly lower than that of HCC patients with high serum miR-493-5p expression (chi2=9.906,P=0.002). CONCLUSION(S): Serum miR-493-5p level is significantly reduced in HCC patients,which can be used as an indicator for HCC diagnosis,disease assessment and prognosis.Copyright Â© 2019, Editorial Board of Chinese Journal of Cancer Prevention and Treatment. All right reserved. |
| Chen, Y., Lin, Y. Y., Hu, M. H. Clinical research of AFP variant with microcentrifugalcolumn method and crossed affinity immunoelectrophoresis autoradiography method. Zhonghua shi yan he lin chuang bing du xue za zhi = Zhonghua shiyan he linchuang bingduxue zazhi = Chinese journal of experimental and clinical virology 2008;22(5):379-81. | OBJECTIVE: To compare the clinical value of microcentrifugalcolumn method with crossed affinity immunoelectrophoresis autoradiography method for the measurement of alpha-fetoprotein variant (AFP-L3) in differentiation of benign and malignant liver. METHOD(S): Serum AFP-L3 variants in 102 primary hepatocellular carcinoma patients and 41 chronic and cirrhosis patients were separated by microcentrifugalcolumn method and crossed affinity immunoelectrophoresis autoradiography method and the clinical value of both method were compared. RESULT(S): In 102 primary hepatocellular carcinoma patients, the sensitivity of AFP-L3 was 79.4% and 91.2% respectively, in 41 chronic and cirrhosis patients. The specificity of AFP-L3 was 70.7% and 29.3% respectively. The diagnostic accuracy was 76.9% and 73.4% respectively. The area in the ROC curve was 0.791 and 0.758 respectively. In the 4 primary hepatocellular carcinoma patients with lower AFP-L3, the AFP-L3 was positive by useing microcentrifugalcolumn method, but none of AFP-L3 were found by useing crossed affinity immunoelectrophoresis autoradiography method. CONCLUSION(S): The micro centrifugalcolumn method is more simple and rapid, it may be more useful in differentiation of benign and malignant liver than traditional crossed affinity immunoelectrophoresis autoradiography method. |
| Chen, Z., Dong, M., Wu, X., Lin, Q., Li, X., Wen, J., Ma, X. The expression of soluble endoglin in patients with liver cirrhosis and hepatocellular carcinoma and its clinical significance. Chinese Journal of Clinical Oncology 2010;37(19):1097-1001. | Objective: To investigate the expression of soluble endoglin in patients with liver cirrhosis and hepatocellular carcinoma (HCC) and to determine its clinical significance. Method(s): We used Enzyme-Linked Immunosorbent Assay (ELISA) to measure the serum concentration of soluble endoglin in 87 HCC patients, 30 hepatic cirrhosis patients and 28 healthy controls. We also collected the clinical data of all of these patients and analyzed the relationship between soluble endoglin serum level and tumor stage, portal vein thrombus, and distant metastasis. Result(s): The serum concentration of soluble endoglin in patients with HCC and hepatic cirrhosis was significantly higher than that in patients with hepatic cirrhosis (P<0.001) and the control group (P<0.001). The serum concentration of soluble endoglin in the hepatic cirrhosis group was significantly higher than that in the control group (P=0.016). The serum concentration of soluble endoglin was highest in HCC patients with a tumor mass larger than 5 cm, distant metastasis, portal vein thrombus, an AFP level higher than 400 ng/mL and at a later clinical stage. ROC analysis showed that combined tes for endoglin and AFP levels has higher sensitivity than assessing either alone, and the AUC was increased. Conclusion(s): Soluble endoglin has the potential to be a novel complementary biomarker in the risk assessment for development of HCC in cirrhotic patients and can be used as an early diagnostic index. Soluble endoglin can be used as a marker for the recurrence, metastasis and prognosis of HCC. |
| Cheng, T. Clinical significance of AFP-L3,Hsp90alpha test in the diagnosis of hepatocellular carcinoma with low and medium concentration of AFP. Chinese Journal of | OBJECTIVE Alpha fetoprotein(AFP) has some limitations in the diagnosis of hepatocellular carcinoma(HCC) with low and medium levels of AFP.The aim of this study was to investigate the value of serum AFP-L3 and heat shock protein 90alpha(Hsp90alpha) in the diagnosis of HCC exhibiting low and medium levels of AFP (20-400 ng/mL).METHODS Totally 102 cases of HCC with low and medium levels of AFP,113 cases of benign liver diseases and 150 cases of healthy controls were collected.Levels of AFP-L3 and AFP were quantified by chemiluminescent immunoassay.Percentages of AFP-L3 in total AFP were calculated.The levels of Hsp90alpha were analyzed by enzyme-linked immunosorbent assay.AFP-L3(%) and the levels of Hsp90alpha |

| Ref | Abstract |
|---|---|
| Cancer Prevention and Treatment 2018;25(21):1511-14. | in each group were compared and the value of them in the diagnosis of HCC was evaluated using logistic regression and receiver operating characteristic curve.RESULTS AFP-L3(%) in patients with HCC (14.27%) were higher than those in patients with benign liver diseases (5.05%) and healthy controls (1.20%),H values were 85.03 and 155.79,all P<0.001.Levels of Hsp90alpha in HCC patients (145.19 ng/mL) were higher than those in benign liver diseases patients (81.31 ng/mL) and healthy controls (20.77 ng/mL),H values were 49.40 and 138.48,all P<0.001.AFP-L3(%) and levels of Hsp90alpha in benign liver diseases patients were higher than those in healthy controls,H values were 128.00 and 132.53,all P<0.001.The specificity (0.982) of AFP-L3(%) was higher than that of Hsp90a (0.673),P<0.001,the positive predictive value (0.973) of AFP-L3(%) was higher than that of Hsp90alpha (0.681),P<0.001,the diagnostic coincidence rate (0.851) of AFP-L3(%) was higher than that of Hsp90alpha(0.721),P=0.023,the area under curve(AUC) of AFP-L3(%) was 0.864,higher than that of Hsp90alpha(0.778),P=0.048.The sensitivity of 0 AFP-L3(%) is 0.706 lower than that of Hsp90alpha(0.775),P=0.009.The sensitivity(0.941),negative predictive value(0.944),accordance rate(0.921) and AUC(0.962) of the combined detection were higher than AFP-L3(%) or Hsp90alpha alone,all P<0.05.CONCLUSIONS AFP-L3(%) and Hsp90alpha can be used as diagnostic markers of HCC.The combined detection can improve the sensitivity,accordance rate and accuracy of diagnosis of HCC exhibiting low and medium levels of AFP.Copyright Â© 2018, Editorial Board of Chinese Journal of Cancer Prevention and Treatment. All right reserved. |
| Chrzanowska, Alicja, Mielczarek-Puta, Magdalena, Skwarek, Anna, Krawczyk, Marek. [Serum arginase activity in patients with liver cirrhosis and hepatocellular carcinoma]. Arginaza w surowicy krwi chorych z marskoscia i rakiem watrobowokomorkowym. 2007;60(5-6):215-18. | Preoperative and postoperative arginase activity was determined in blood serum of 25 patients with liver cirrhosis and 25 patients with hepatocellular carcinoma. The rise of serum arginase activity was observed in the majority of patients before the surgery and the decrease after tumor resection or liver transplantation. The preoperative values of serum arginase activity were similar in both groups of patients. A presence of additional, anionic arginase isoform (AII) was demonstrated in serum of studied patients, which was absent in healthy subjects. Thus, our results indicate that the arginase activity cannot differentiate liver cirrhosis and hepatocellular carcinoma. However, arginase isoform AII seems to be specific for studied liver diseases. |
| Cui, R. & Li, Y. Combined detection of alpha-fetoprotein and Golgi protein 73 for primary hepatic carcinoma diagnosis. Chinese Journal of Clinical Oncology 2015;42(4):222-25. | Objective: The diagnostic sensitivity of alpha-fetoprotein (AFP) in patients with primary liver cancer is poor and unsatisfactory in the clinical diagnosis of patients with primary liver cancer. Recent studies have demonstrated the upregulation of serum primary hepatic carcinoma (PHC) and Golgi glycoprotein 73 (GP73) in patients with PHC. This study aims to discuss the clinical significance of GP73 and AFP in the serum on the diagnosis of primary liver cancer. Method(s): The serum concentrations of GP73 and AFP were retrospectively analyzed in 46 hepatocellular carcinoma patients, 30 cases of patients with liver cirrhosis alone, 44 patients with other digestive tumors, and 40 healthy subjects as controls. Quantitative euzymelinked immunosorbent assay and electrochemiluminescence immunoassay were conducted to determine the GP73 and AFP levels in the serum, respectively. Result(s): The sensitivity of PHC diagnosis significantly increased with GP73 (73.9%) compared with AFP (56.5%), and the difference was statistically significant (P< 0.01). The sensitivity of AFP combined with GP73 significantly improved to 86.9% compared with the sensitivity of a single indicator (P<0.01). Conclusion(s): GP73 may be used as one of the PHC serum markers for diagnosis. The combined detection of GP73 and AFP offers great potential for future application in the diagnosis of PHC. |
| Dong, C., Chang, C. D., Zhao, D. D., Zhang, X. X., Guo, P. L., Dou, Y., Zhao, S. X. [Clinical value of plasma scaffold protein SEC16A in evaluating hepatitis B-related liver cirrhosis and hepatocellular carcinoma]. Zhonghua gan zang bing za zhi = Zhonghua ganzangbing zazhi = Chinese journal of hepatology 2023;31(6):621-26. | Objective: To investigate the clinical value of plasma scaffold protein SEC16A level and related models in the diagnosis of hepatitis B virus-related liver cirrhosis (HBV-LC) and hepatocellular carcinoma (HBV-HCC). Methods: Patients with HBV-LC and HBV-HCC and a healthy control group diagnosed by clinical, laboratory examination, imaging, and liver histopathology at the Third Hospital of Hebei Medical University between June 2017 and October 2021 were selected. Plasma SEC16A level was detected using an enzyme-linked immunosorbent assay (ELISA). Serum alpha-fetoprotein (AFP) was detected using an electrochemiluminescence instrument. SPSS 26.0 and MedCalc 15.0 statistical software were used to analyze the relationship between plasma SEC16A levels and the occurrence and development of liver cirrhosis and liver cancer. A sequential logistic regression model was used to analyze relevant factors. SEC16A was established through a joint diagnostic model. Receiver operating characteristic curve was used to evaluate the clinical efficacy of the model for liver cirrhosis and hepatocellular carcinoma diagnosis. Pearson correlation analysis was used to identify the influencing factors of novel diagnostic biomarkers. Results: A total of 60 cases of healthy controls, 60 cases of HBV-LC, and 52 cases of HBV-HCC were included. The average levels of plasma SEC16A were (7.41 +/- 1.66) ng/ml, (10.26 +/- 1.86) ng/ml, (12.79 +/- 1.49) ng /ml, respectively, with P < 0.001. The sensitivity and specificity of SEC16A in the diagnosis of liver cirrhosis and hepatocellular carcinoma were 69.44% and 71.05%, and 89.36% and 88.89%, respectively. SEC16A, age, and AFP were independent risk factors for the occurrence of HBV-LC and HCC. SAA diagnostic cut-off values, sensitivity, and specificity were 26.21 and 31.46, 77.78% and 81.58%, and 87.23% and 97.22%, respectively. The sensitivity and specificity for HBV-HCC early diagnosis were 80.95% and 97.22%, respectively. Pearson correlation analysis showed that AFP level was positively correlated with alanine aminotransferase (ALT), aspartate aminotransferase (AST), total bilirubin (TBil), and gamma-glutamyltransferase (GGT) with P < 0.01, while the serum SEC16A level was only slightly positively correlated with ALT and AST in the liver cirrhosis group (r = 0.268 and |

| Ref | Abstract |
|---|---|
| | 0.260, respectively, P < 0.05). Conclusion: Plasma SEC16A can be used as a diagnostic marker for hepatitis B-related liver cirrhosis and hepatocellular carcinoma. SEC16A, combined with age and the AFP diagnostic model with SAA, can significantly improve the rate of HBV-LC and HBV-HCC early diagnosis. Additionally, its application is helpful for the diagnosis and differential diagnosis of the progression of HBV-related diseases. |
| Fan, G. R., Xiong, J. H., Lin, H. Y., Li, L., Jiang, S. Y. Related clinical pathologic correlation analysis of glypican-3 expression in hepatocellular carcinoma. Cancer Research and Clinic 2013;25(10):685. | Objective: To investigate the clinical value of glypican-3 (GPC3) in the diaganosis of hepatocellular carcinoma (HCC), the contents of GPC3 in the serum and tissues of HCC patients were detected. Method(s): ELISA and immunohistochemical staining were applied to detect GPC3 expressing level in the serum and tissues in 79 cases with HCC, 35 cases with post-hepatitis cirrhosis and 30 normal liver specimens and the results were compared. The influential factor of GPC3 content in the patients with HCC was analyzed by logistic regression model. Result(s): The serum level of GPC3 in patients with HCC was (143.02+/-40.26) mug/L which was signifcantly higher than that in patients with post-hepatitis cirrhosis [(6.15+/-4.31) mug/L] and healthy controls [(4.47+/-3.22) mug/L] (all P < 0.01). The expression levels of GPC3 was signifcantly higher in post-hepatitis cirrhosis tumor-adjacent tissue and tumor-distant tissue. The expression levels of GPC3 was significantly positively correlated with tumor presence of distant mestasis (chi2 = 13.182, P < 0.0) and clinical stage (chi2 = 4.250, P < 0.05), and not correlated with sex, age, tumor size and the level of AFP in serum (P < 0.01). Conclusion(s): GPC3 is specific the diagnosis of HCC. The joint diagnosis of GPC3 and AFP will improve the sensitivity of HCC. Therefore, GPC3 could as a biomarker for evaluating HCC condition and prognsis. |
| Fan, R., Huang, W., Xiao, S. W., Luo, B., He, S. J., Luo, G. R. OY-TES-1 expression and serum immunoreactivity in hepatocellular carcinoma. World Chinese Journal of Digestology 2009;17(32):3307-12. | AIM: To invest igate OY-TES-1 expression and s e rum immunor eac t ivi ty in human hepatocellular carcinoma (HCC) and explore the possibility of using OY-TES-1 as a target for immunotherapy and auxiliary diagnosis of HCC. METHOD(S): The expression of OY-TES-1 mRNA and protein was detected by real-time PCR and immunohistochemistry, respectively. The presence of serum OY-TES-1 antibody in normal individuals and patients with HCC or hepatocirrhosis was determined by enzyme-linked immunosorbent assay (ELISA) using purified OYTES-1 as the antigen. The clinical significance of OY-TES-1 expression and serum immunoreactivity in HCC was then analyzed. RESULT(S): The positive rates of OY-TES-1 mRNA expression in HCC and tumor-adjacent tissue were 73.21% (41/56) and 64.86% (24/37), respectively, showing no significant difference (P > 0.05). The expression level of OY-TES-1 mRNA in HCC tissue was significantly higher than that in tumor-adjacent tissue (P < 0.05). The expression level of OY-TES-1 mRNA was correlated with histological grade of HCC. The positive rate of OY-TES-1 protein expression in HCC tissue was 40% (4/10). The positive rate of serum OY-TES-1 antibody was 20% in HCC patients, while no serum reactivity to OY-TES-1 was detected in 76 normal individuals and 17 patients with hepatocirrhosis. No correlation was found between the presence of OY-TES-1 antibody and clinical and pathological parameters in HCC. CONCLUSION(S): OY-TES-1 shows high-level expression and high serum immunoreactivity in HCC and is therefore a potential target for antigen-specific immunotherapy and auxiliary diagnosis of HCC. |
| Fan, Z. J., Liu, S., Zhang, L., Tian, Y. Q. Value of combined detection of serum vitronectin, alpha-1-B glycoprotein, antithrombin-III, and alpha fetoprotein for diagnosis of early hepatocellular carcinoma. World Chinese Journal of Digestology 2018;26(14):842-48. | AIM To evaluate the value of combined detection of serum vitronectin (VTN), alpha-1-B glycoprotein (A1BG), antithrombin-III (AT-III), and alpha fetoprotein (AFP) for early diagnosis of hepatocellular carcinoma (HCC). METHODS ELISA was used to detect the concentrations of serum VTN, A1BG, and AT-III in 160 patients with HCC, 70 patients with chronic hepatitis B (CHB), 70 patients with liver cirrhosis (LC), and 50 healthy controls (HC), and electrochemical luminescence was used to detect serum concentration of AFP. The changes of VTN, A1BG, AT-III, histidine-rich glycoprotein (HRG), and AFP in different groups of subjects were compared. The sensitivity and specificity of HRG, A1BG, AFP, and AT-III, alone or in different combinations, in the diagnosis of HCC were determined by receiver operating characteristic (ROC) curve analysis. Serum AFP, A1BG, and AT-III were then used as independent variables to establish a comprehensive prediction model for HCC. RESULTS The levels of serum AFP, VTN, A1BG, and AT-III were significantly different in the four groups (F = 1498.93, 51.68, 84.00, 115.34, P < 0.05). There was no significant difference in the level of VTN between the HCC group and other groups (F = 1.31), and its performance as a screening index was poor. The areas under the ROC curves (AUCs) of AFP, HRG, A1BG, and AT-III were 0.878, 0.579, 0.712, and 0.801, respectively. The AUC of VTN was the lowest, suggesting that it has no diagnostic value. The AUC of the comprehensive prediction model was 0.923, which was significantly higher than those of A1BG and AT-III (P < 0.05). The sensitivities of AFP, A1BG, and AT-III for the diagnosis of HCC were 70.00%, 64.37%, and 61.25%, respectively, the specificities were 91.05%, 74.74%, and 83.68%, respectively, and the AUCs were 0.878, 0.712 and 0.801, respectively. The sensitivity and specificity of the integrated prediction model were 85.00% and 88.42%, respectively. The AUC was 0.923, which was significantly different from the single diagnosis (P < 0.05). The predictive equation was P = 1/[1+exp (0.152-0.035 AFP-0.006 A1BG + 0.021 AT-III)], which had a good diagnostic performance. CONCLUSION Combined detection of serum AFP, A1BG, and AT-III can improve the early diagnosis of HCC.Copyright Â© The Author(s) 2018. Published by Baishideng Publishing Group Inc. All rights reserved. |
| Fang, Q. Q., Ni, R. Z., Xiao, M. B., Jiang, F. [Serum and tissue expressions of galectin-3 in hepatocellular carcinoma and the clinical significances]. | To study the expression of Galectin-3 in human hepatocellular carcinoma (HCC) tissues and the clinical value of serum Galectin-3 in the diagnosis of hepatocellular carcinoma. Immunohistochemistry method was used to detect the expression of Galectin-3 in the 46 pairs of HCC tissues and their para cancerous tissues. The relationship between expression levels of Galectin-3 and clinical parameters was analyzed. Serum Galectin-3 in different liver diseases were measured with ELISA. The sensitivity and specificity of galectin-3, alpha fetoprotein (AFP) and gamma-glutamyltranspeptidase II (GGT-II) for diagnosis of HCC were compared and the |

| Ref | Abstract |
|---|---|
| Zhonghua gan zang bing za zhi = Zhonghua ganzangbing zazhi = Chinese journal of hepatology 2011;19(7):527-31. | complementary diagnostic values of Galectin-3 and AFP and GGT-II for HCC were studied. (1) The positive rate of Galectin-3 in the tissue of HCC was 78.2%, dramatically higher than that in para cancerous tissues (15.2%) (P is less than 0.01). The expression levels were correlated with differentiation and with the high expression in poor differentiation tissues; (2) Based on ROC curve, the cut-off of serum Galectin-3 for HCC diagnosis was set as 0.62mug/L, the serum galectin-3 positive rate was 64.5% in HCC cases, which was apparently higher than that in liver cirrhosis, chronic hepatitis and healthy persons (P is less than 0.05); (3) Serum Galectin-3 was not correlated with AFP and GGT-II. Combined determination of the three markers had the complementary diagnostic value for HCC and might increase the diagnostic sensitivity to 94.7%. Galectin-3 is overexpressed in HCC tissues and is correlated with the tumor differentiation, suggesting that Galectin-3 may be associated with the carcinogenesis and development of HCC. Serum galectin-3 increases in the HCC cases and combined determination of serum Galectin-3, AFP and GGT-II can increase the diagnostic efficiency for HCC. Galectin-3 could be a novel serum tumor marker for HCC. |
| Gao, Dong-mei, Sun, Lu, Guo, Kun, Li, Yan, Liu, Yin-kun. [Applicability of the multiplex quantitative antibody array system for early diagnosis of hepatocellular carcinoma]. Zhonghua gan zang bing za zhi = Zhonghua ganzangbing zazhi = Chinese journal of hepatology 2012;20(10):785-88. | OBJECTIVE: To develop an early and accurate detection method for hepatocellular carcinoma (HCC) based on detection of tumor-associated serum markers using a multiplex quantitative antibody array., METHODS: The double-antibody sandwich principle was used to establish an antibody array composed of eight cancer-related serum markers, including alpha-fetoprotein (AFP), hepatocyte growth factor (HGF), insulin-like growth factor (IGF), interleukin-6 (IL-6), interleukin-8 (IL-8), interleukin-10 (IL-10), transforming growth factor-beta 1 (TGF-b1), and vascular endothelial growth factor (VEGF). Serum samples from 160 cases of clinically diagnosed HCC and from 58 cases of liver cirrhosis (LC; controls) were obtained to test the array. Sixty percent of the samples were randomly selected for use as the training set (HCC, n = 96; LC, n = 36), and the remaining 40% was used as the test set (HCC, n = 64; LC, n = 22). The SPSS statistical software was used to perform logistic regression analysis and to create a diagnostic model., RESULTS: When used with the training set, the model had sensitivity of 93.3%, specificity of 83.3%, and accuracy of 90.9%. When used with the test set, the model had sensitivity of 89.0%, specificity of 77.3%, and accuracy of 86.0%. The traditional serum AFP value (cut-off value of 20 ng/mL) showed 70.0% diagnostic sensitivity, 59.0% specificity, and 64.0% accuracy., CONCLUSION: The newly developed multiplex quantitative antibody detection system has high sensitivity and specificity. The diagnostic model with AFP and seven other cancer-related factors was superior to the traditional AFP only approach for early diagnosis of liver cancer, indicating its potential clinical value. |
| Gao, H., Li, X., Wang, J., Qiao, F., Gao, F. Clinical application of combined detection of serum Cys-C,5'-NT and AFP in hepatocellular carcinoma. Cancer Research and Clinic 2014;26(11):753-56. | Objective: To explore the clinical application of single or combined detection of serum Cys-C,5'-NT and AFP in hepatocellular carcinoma (HCC) in order to provide the platform for early diagnosis and prognosis evaluation. Method(s): The serum levels of Cys-C,5'-NT and AFP from 148 cases of HCC patients (HCC group), 135 cases of liver cirrhosis patients (LC group) and 155 cases of hepatitis (hepatitis group) and 100 healthy people (control group) were measured by latex-enhanced immunoturbidimetric assay (LEITD), peroxidase method and electrochemiluminescence immunoassay (ECLI), respectively. Then their differences had been compared. The sensitivity, specificity and Youden's index were calculated and the results were analyzed by receiver operating characteristic (ROC) curve. Result(s): The levels of serum Cys-C,5'-NT and AFP were significantly different in 4 groups. The differences were statistically significant (F = 12.35, 42.25, 58.12, P =0.000). The sensitivity of three indicators for diagnosing HCC was 100 %, which was higher than that of single or two combined detection (P < 0.05), and the specificity was 78.97 %. The area under ROC curves of combined detection of three indicators was 0.977, which was also higher than single or two combined detection (P < 0.05). Conclusion(s): The combined detection of serum Cys-C,5'-NT and AFP can greatly increase the sensitivity and accuracy for diagnosing HCC, which has an important clinical value for early diagnosis. Therefore it is worth popularizing and applying on clinic. |
| Gao, H., Zhao, Q., Wang, J., Gao, F., Qiao, F. Application of serum alpha-fetoprotein, alpha-L-fucosidase, 5'-nucleotidase and gamma-glutamyltransferase in early diagnosis of hepatocellular carcinoma. Cancer Research and Clinic 2016;28(8):519-27. | Objective: To investigate the application of alpha-fetoprotein (AFP), alpha-L-fucosidase(AFU), 5'-nucleotidase(5'-NT) and gamma-glutamyltransferase (GGT) in diagnosis of hepatocellular carcinoma(HCC), and to provide the basis for its early and rapid diagnosis. Method(s): The clinical data of 120 cases with initial diagnosis as hepatocellular carcinoma, 150 cases with liver cirrhosis, 200 cases with hepatitis were analyzed retrospectively from June 2013 to August 2014, meanwhile, 100 healthy people were selected as control group. The serum AFP, AFU, 5'-NT and GGT levels were measured by electrochemiluminescence immunoassay, rate method, peroxidase method and enzymatic colorimetric assay, respectively. The results were analyzed by receiver operating characteristic (ROC) curve, while the sensitivity, specificity, AUC were calculated at the same time. Result(s): The levels of serum AFP, AFU, 5'-NT and GGT were significantly different among HCC group, liver cirrhosis group, hepatitis group and control group (all P<0.05). When tested alone, the sensitivities of AFP, AFU, 5'-NT and GGT were 58.33 %(70/120), 82.50 %(99/120), 85.00 %(102/120), 78.33 %(94/120), the specificities were 88.33 %(106/120), 73.33 %(88/120), 88.33 %(106/120), 71.67 %(86/120), and AUC were 0.655, 0.702, 0.814, 0.754. When four indexes were performed with combined test, the sensitivity was 98.33 %(118/120), specificity was 96.67 %(116/120), AUC was 0.975, which were higher than the single detection (all P<0.05). Conclusion(s): The combined detection of serum AFP, AFU, 5'-NT and GGT can greatly increase the sensitivity and specificity for diagnosing HCC and will gain mutual complement advantages, which is of great clinical value for early and rapid diagnosis. |
| Gomez Rubio, M. & De Cuenca Moron, B. Tumoral markers in liver | |

| Ref | Abstract |
|---|---|
| cirrhosis and hepatocellular carcinoma [3]. Gastroenterologia y Hepatologia 2005;28(5):308. | |
| Guo, H. M., Li, J. S., Zhang, J. P., Li, D. Y. Significance of serum SCD25 level in diagnosis of hepatocellular carcinoma. World Chinese Journal of Digestology 2014;22(34):5344-49. | AIM: To explore the significance of serum sCD25 level in the diagnosis of hepatocellular carcinoma (HCC)., METHODS: A total of 160 subjects, including 40 patients with HCC, 40 patients with liver cirrhosis, 40 patients with chronic hepatitis B, and 40 healthy controls, were measured for serum sCD25 levels by enzyme-linked immuno sorbent assay (ELISA)., RESULTS: Serum sCD25 level in the HCC group (mean, 9641.23 pg/mL) was significantly higher than those in the cirrhosis group, chronic hepatitis B group and healthy control group (P < 0.05). Serum sCD25 level demonstrated a better sensitivity than alpha fetal protein (AFP) in the diagnosis of HCC., CONCLUSION: Serum sCD25 level is effective in the detection of early HCC, and it may serve as a novel predictive marker of HCC to distinguish advanced cirrhosis from early HCC.Copyright Â© 2014 Baishideng Publishing Group Inc. All rights reserved. |
| Haberman, Diego, Mela, Martin, Martinez, Adriana, Mancinelli, Adrian, Laguens, Ruben, Gruz, Fernando. [Accuracy of multislice computed tomography in the diagnosis of hepatocellular carcinoma in patients with cirrhosis evaluated for liver transplantation]. Precision de la tomografia multidetector en el diagnostico de hepatocarcinoma en pacientes con cirrosis evaluados para trasplante hepatico. 2011;41(3):190-98. | BACKGROUND: The presence of hepatocellular carcinoma (HCC) within Milan criteria provides additional points in the MELD (Model for end stage liver disease) system and benefits in the order of organ allocation. The imaging methods play a key role in this process and represent an essential tool in the diagnosis and staging of HCC., OBJECTIVES: 1) To assess the accuracy of dynamic multidetector computed tomography (MDCT) in the diagnosis of HCC in patients with cirrhosis who are listed for liver transplantation. 2) To evaluate the diagnostic performance of TCMD in relation to tumor size., MATERIAL AND METHODS: We retrospectively reviewed the reports of MDCT performed in our institution to 62 patients who were then transplanted The histological analysis of explants was considered as the reference method in the diagnosis of HCC. MDCT studies were performed with dynamic protocol in arterial, portal and late phases., RESULTS: Dynamic MDCT showed a sensitivity of 87.5% and correctly characterized 28 of 35 patients with pathology proved hepatocellular carcinoma. MDCT was negative in 25 of 30 patients without hepatocellular carcinoma in the explanted liver, with a specificity of 83.3%. Nodule by nodule evaluation revealed a sensitivity of 80.3% and a specificity of 72.2%., CONCLUSION: In our center MDCT presented high accuracy in the correct diagnosis of HCC, showing its reliability when requesting additional points for organ allocation. |
| He, N., Feng, G., Zhang, F. N., Hao, S., Li, R., Zhao, Z. Q., Tian, Y. W. [Expression and clinical significance of plasma methylated SEPT 9 gene in patients with primary liver cancer]. Zhonghua gan zang bing za zhi = Zhonghua ganzangbing zazhi = Chinese journal of hepatology 2023;31(3):265-70. | Objective: To investigate the expression and clinical significance of plasma methylated SEPT9 (mSEPT9) gene in patients with primary liver cancer. Methods: 393 cases who visited our hospital from May 2016 to October 2018 were selected. Among them, 75 cases were in the primary liver cancer (PLC) group, 50 cases were in the liver cirrhosis (LC) group, and 268 cases were in the healthy control group (HC). The three groups' positive rates of mSEPT9 expression in the peripheral plasma were detected by the polymerase chain reaction (PCR) fluorescent probe method. The correlational clinical features of liver cancer were analyzed. At the same time, the electrochemiluminescence detection method was used to compare the AFP positive rate. Statistical analysis was conducted using chi-square tests or continuity-corrected chi-square tests. Results: 367 cases actually had valid samples. There were 64, 42, and 64 cases in the liver cancer group, cirrhosis group, and healthy control group, respectively. Among them, 34 cases of liver cancer were verified from pathological tissues. The positive rate of plasma mSEPT9 was significantly higher in the liver cancer group than that in the liver cirrhosis and healthy control groups [76.6% (49/64), 35.7% (15/42), and 3.8% (10/261), respectively], and the differences were statistically significant (chi (2) = 176.017, P < 0.001). The sensitivity of plasma mSEPT9 detection (76.6%) was significantly better in liver cancer (76.6%) than that of AFP patients (54.7%), and the difference was statistically significant (chi (2) = 6.788, P < 0.01). Compared with the single detection, the sensitivity and specificity of plasma mSEPT9 combined with AFP were significantly improved (89.7% vs. 96.3%, respectively). Patients with liver cancer aged>=50 years, with clinical stage II or above, and those with pathological signs of moderate to low differentiation had higher levels of plasma mSEPT9 positive expression, and the differences were statistically significant (chi (2) = 6.41, 9.279, 6.332, P < 0.05). During the follow-up period, the survival time of liver cancer patients with positive plasma mSEPT9 expression was significantly shorter than that of those with negative expression (310 +/- 26 days vs. 487 +/- 59 days, respectively), with statistically significant differences (Log Rank P = 0.039). Conclusion: In China, the positive rate of plasma mSEPT9 detection in liver cancer patients is higher than that of AFP in relation to age, clinical stage, and degree of tissue differentiation; additionally, it has certain survival predictive values. As a result, detecting this gene has important clinical significance and potential clinical application value in the non-invasive diagnosis and prognosis assessment of patients with primary liver cancer. |
| Hirashima, N., Shimada, M., Urata, N. Surveillance of hepatocellular carcinoma using liver | To investigate predictive factors for hepatocellular carcinoma (HCC) occurrence and recurrence after sustained virologic response (SVR) by direct-acting antivirals (DAAs); 229 patients with hepatitis C were prospectively followed for > 1 year. Out of those, 207 had no history of HCC, while 22 had HCC treated by radiofrequency ablation or resection before DAA. Liver stiffness (LS) was measured using transient elastography and the assessments were performed at the initiation |

| Ref | Abstract |
|---|---|
| stiffness in patients with hepatitis C after sustained virologic response by direct-acting antivirals. Acta Hepatologica Japonica 2022;63(6):268-78. | of DAA 24 weeks (LS24) and 48 weeks (LS48) after that. HCC developed in 11 out of 207 and 12 out of 22 patients without and with history of HCC, respectively. Both patients with and without history of HCC had significantly higher alpha-fetoprotein levels at SVR24 and LS48. No HCC was recognized in patients with normalized LS at LS24. Surveillance of HCC in patients with normalized LS at LS24 might be performed at longer than 6 months.Copyright Â© 2022 The Japan Society of Hepatology. |
| Honglian, J., Caiyun, H., Lijun, S., Shilong, L., Sugui, H., Xiaorui, W. Diagnostic value of serum AFP-L3, GP73 and GGT combined detection in hepatocellular carcinoma. Cancer Research and Clinic 2014;26(7):465-67. | Objective: To explore the clinical value of AFP-L3, GP73 and GGT as biomarkers in diagnosis of hepatocellular carcinoma (HCC). Method(s): According to the pathological diagnosis, 141 patients were divided into two groups, HCC group were 74 cases, benign liver disease group were 67 cases. Use ELISA method tested the serum AFP-L3 and GP73 levels. The GGT level was detected by the automatic biochemical instrument of all the 141 patients. AFP-L3, GP73 and GGT concentration difference was compared between the two groups. ROC curve was used to determine the cut-off level to diagnose HCC. The value of single use AFP-L3, GP73, GGT and joint the three indexes to diagnose HCC were analyzed. Result(s): The average level of AFP-L3 in the patients with HCC was (113.58+/-63.62) mug/L, it was significantly higher than that in the patients with benign liver diseases [(23.19+/-34.54) mug/L] (P < 0.001). The area under the ROC curve of AFP-L3 level was 0.802. Taking AFP-L3 level >= 38.47 mug/L as diagnostic criteria, the sensitivity of AFP-L3 level in HCC diagnosis was 81.08 % and the specificity was 88.06 %. The average level of GP73 in the patients with HCC was (126.55+/-49.56) mug/L, it was significantly higher than that in the patients with benign liver diseases [(56.97 +/-26.48) mug/L] (P < 0.001). The area under the ROC curve of GP73 level was 0.811. Taking GP73 level >= 69.44 mug/L as diagnostic criteria, the sensitivity of GP73 level in HCC diagnosis was 75.68 % and the specificity was 91.04 %. The average level of GGT in the patients with HCC was (173.20+/-179.18) U/L, it was significantly higher than that in the patients with benign liver diseases [(90.77 +/-81.53) U/L] (P > 0.001). The area under the ROC curve of GGT level was 0.713. Taking GGT level>= 110.77 U/L as diagnostic criteria, the sensitivity of GGT level in HCC diagnosis was 74.32 % and the specificity was 77.61 %. Joint use AFP-L3, GP73 and GGT to diagnose HCC, the sensitivity was 83.78 %, specificity was 92.53 %. Conclusion(s): Combined detection of tumor markers AFP-L3, GP73 and GGT can improve the positive rate of HCC, which has good clinical application value. |
| Hou, Z. J., Wang, X. W., Zhang, L. H., Zhou, X. Y., Dai, D. L. Clinical significance of serum leptin, vascular endothelial growth factor and alpha-fetoprotein expression in hepatocelluar carcinoma. World Chinese Journal of Digestology 2006;14(33):3195-2000. | AIM: To explore the expression of serum leptin (Lep), vascular endothelial growth factor (VEGF) and alpha-fetoprotein (AFP) and their clinical significances in hepatocelluar carcinoma (HCC). METHOD(S): Enzyme-linked immunosorbent assay (ELISA) was used to detect serum Lep, VEGF and AFP levels in 146 patients with HCC, 31 patients with liver cirrhosis and 30 healthy controls. The correlations Lep, VEGF, and AFP expression with clinical indexes were analyzed. RESULT(S): The serum Lep level was correlated with body mass index (BMI) in HCC patients (r = 0.64, P < 0.01), and significantly lower than that in cirrhotic patients and healthy controls (19 mug/L vs 35, 27 mug/L, P < 0.05). The serum Lep level in HCC patients was significantly decreased with upgrade of TNM stages, metastasis and recurrence (P < 0.05). In comparison with that in cirrhotic patients and healthy controls, the serum VEGF level in HCC patients was significantly increased (398 ng/L vs 179, 167 ng/L, P < 0.01). There was no correlation between VEGF and AFP expression. The sensitivity and specificity of VEGF were 71.2% and 80.6%, and those of AFP were 73.3% and 83.9%, respectively, in the diagnosis of HCC. However, the sensitivity was increased to 91.8%, while the accuracy reached 90.4% after the combined detection of VEGF and AFP was used. There was a relationship beween the level of serum VEGF and tumor size (<3 cm: 237 +/- 96 ng/L, >3 cm: 398 +/- 124 ng/L, P < 0.01). As compared with that of TNM I, II stage, the serum VEGF of TNM III, IV stage was obviously higher (346 +/- 131, 401 +/- 152 ng/L vs 228 +/- 89, 259 +/- 102 ng/L, P < 0.01), and it also showed a significant elevation in the patients with metastasis and recurrent HCC (P < 0.01). CONCLUSION(S): Nutrition status and prognosis of patients with HCC can be judged by serum Lep level, and the combined detection of serum VEGF and AFP may provide a significant marker for HCC diagnosis and prognosis. |
| Hu, Y., Hua, D., Cheng, Z. H., Wu, Y. Y., Xie, Q. G., Wang, Q. Y., Yu, J. Clinical value of methylation of plasma adenomatous polyposis coli gene in the molecular diagnosis of hepatocellular carcinoma. Tumor 2011;31(10):924-29. | Objective: To establish a method of methylation-sensitive restriction enzymes-quantitative PCR (MSRE-qPCR) for methylation analysis of adenomatous polyposis coli (APC ) gene, and to further assess the clinical value of plasma methylation detection by using this method in diagnosis of hepatocellular carcinoma (HCC). Method(s): Hha I was used to digest genomic DNA, and the digestion efficiency was evaluated by using qPCR technique. Then the optimized MSRE-qPCR method was established. The methylation levels of APC in 45 liver tissues (20 surgically resected HCC specimens and the matched non-cancerous tissues, as well as 5 normal liver tissues) were detected by MSRE-qPCR, and then further validated by using bisulfite sequencing PCR (BSP). The results of MSRE-qPCR were compared with those of methylation-specific PCR (MSP) assay. MSRE-qPCR method was used to detect the APC methylation status of plasma samples from 72 cases of H CC, 37 cases of benign liver diseases and 41 healthy volunteers. Result(s): The established MSRE-qPCR method could detect as low as 1% methylated target sites in given DNA samples. The results of MSRE-qPCR and MSP showed that APC gene was hypermethylated in HCC tissues. The result of MSRE-qPCR was verified by BSP, and it was comparable with that of MSP (Kappa=0.955, P<.000 1). Methylation level of plasma APC in patients with H CC was significant higher than those in patients with benign liver diseases and the healthy volunteers (P<.000 1).Combined analysis of plasma APC methylation and serum alpha-fetoprotein (AFP) revealed an increased diagnostic efficacy for HCC. Conclusion(s): MSRE-qPCR is a method for quantitative analysis of APC methylation level. Methylation analysis of plasma APC is a valuable method for the noninvasive diagnosis of HCC. CopyrightÂ© 2011 by TUMOR. |

| Ref | Abstract |
|---|---|
| Inoue, Takako. [Clinical Significance of Novel Serum Biomarkers in the Management of Liver Diseases]. Rinsho byori. The Japanese journal of clinical pathology 2017;65(1):83-91. | Serum biomarkers are playing an increasingly important role in the management of liver diseases. In this article, we provide an overview of the biomarkers that are currently used for the primary diagnosis of liver fibrosis, hepatocellular carcinoma (HCC), and occult hepatitis B virus (HBV) infection. Because liver fibrosis is a significant risk factor for hepatocarcinogenesis, monitoring its progression is important for predicting and preventing HCC. In 2015, the novel liver fibrosis glycobiomarker Wisteriaflori- bunda agglutinin-positive Mac-2 binding protein (WFA'-M2BP, M2BP glycan isomer [M2BPGil) became available in Japan. The HISCL M2BPGi assay is fully automated, rapid (17 minutes), and requires only a small sample volume (10 muL). Serum tumor markers are noninvasive and valuable for the management of patients with a high risk of de- veloping HCC. Although a-fetoprotein (AFP) has been commonly used, its insufficient sensitivity and specificity for early-stage HCC make it unsuitable for managing patients at high risk of developing HCC. However, a combination of AFP and protein induced by vitamin K absence factor II (PIVKA-II), or Lens culinaris agglutinin-reactive fraction of AFP (AFP-L3) and PIVKA-II are currently being evaluated for HCC pre- diction. Recently, a highly sensitive chemiluminescent enzyme immunoassay (CLEIA) for HBsAg detection by Lumipulse HBsAg-HQ has been reported as the latest clinical application. Although the sensitivity of this assay ( >=5 mIU/mL) is 10-fold higher than that of the conventional assay, it is still lower than that of the HBV-DNA assay. HBsAg-HQ will be applied for detecting occult HBV infection and HBV reactivation. [Review]. |
| Ishida, Haku & Matsuo, Shuji. [Evaluation of diagnostic performance of alpha-fetoprotein (AFP) and des-gamma-carboxy prothrombin (DCP) for HCV related hepatocellular carcinoma developed after long-term follow up]. Rinsho byori. The Japanese journal of clinical pathology 2010;58(11):1065-72. | PURPOSE: This study evaluated the diagnostic performance of two tumor markers, alpha-fetoprotein (AFP) and des-gamma-carboxy prothrombin (DCP), a combination of these tests and other common tests for patients developing hepatocellular carcinoma (HCC) during long-term follow-up for HCV-related liver disease., METHODS: We reviewed the medical records of 144 patients who developed HCC after at least one-year follow up for HCV-related liver disease in Yamaguchi University Hospital or Tenri Hospital and demographic, clinical and laboratory data were collected retrospectively. To evaluate diagnostic test performance, we defined the oldest data collected from each patient at least one year before the diagnosis of HCC as negative control data., RESULTS: Sensitivity and specificity were 0.52, 0.61 for AFP with a cut-off of 20 ng/ml compared to 0.47 and 1.00 for DCP with a cut-off of 40 mAU/ml, respectively. Combination tests for AFP at 100 ng/ml and DCP at 40 mAU/ml yielded the best sensitivity at 0.84 with a specificity of 0.50. Multivariate logistic regression model using AFP, AST, LD, hemoglobin, prothrombin time and male ratio as variables showed significantly better diagnostic performance, i.e. sensitivity 0.85, specificity 0.74, than single test or combination test using AFP and DCP alone., CONCLUSIONS: AFP and DCP alone were not sufficient for detection of HCC developing during long-term follow-up of HCV-related cirrhosis. Logistic regression including AFP and commonly used laboratory tests showed superior diagnostic accuracy compared to that of tumor marker tests alone. |
| Jia, K. D. & Shi, S. X. Values of reactive oxygen toxic species of finger blood dot in the diagnosis of hepatocellular carcinoma. World Chinese Journal of Digestology 2006;14(24):2406-10. | AIM: To observe the morphology and inclusion body of reactive oxygen toxic species (ROTS) of finger blood dot in hepatocellular carcinoma (HCC) and liver cirrhosis (LC) by multimedia microscopy and explore its value in the diagnosis of HCC. METHOD(S): The finger blood dots were prepared from 28 patients with HCC, 43 patients with LC, 38 patients with other carcinomas. The blood dots were scanned and the morphology and inclusion body of ROTS were analyzed by high-vivid color pathology image analysis system (HPIAS-1000) between different groups of patients. RESULT(S): The enlarged ROTS was detected in 20 of 28 patients with HCC and 16 of 43 patients with LC. Fifteen patients with HCC were found to have pricks, something like nails, or transparent body inside the enlarged ROTS, but only 8 patients with LC and no patients with other carcinomas showed ROTS morphological changes above. Regarding the pricks or transparent body as positive mark, the positive rate of HCC was 53.6%, significantly higher than that of LC (18.6%) (P = 0.0038). Further study indicated that the levels of serum sialic acid and alpha-fetoprotein (AFP) in ROTS-positive patients with HCC were not significant higher than those of ROTS-negative ones. The ages of patients, the levels of alanine aminotransferase (ALT) and Child-Pugh grades for liver function were not significantly different between ROTS-positive and negative patients, either. CONCLUSION(S): ROTS of finger blood dots in HCC patient shows morphological changes of pricks and transparent body by multimedia microscopy, which can be used to differentiate most HCC from LC patients. |
| Jiang, H. J., Xu, K., Bai, R. J., Liu, B. L., Wang, D. Quantitative study of hepatic dynamic flow in hepatocellular carcinoma by multi-slice CT perfusion. Chinese Journal of Medical Imaging Technology 2007;23(5):711-14. | Objective To study the changes of perfusion parameters in hepatocellular carcinoma (HCC) by multi-slice computed tomography (MSCT) perfusion technique. Method(s): Perfusion CT images were obtained in 30 patients with HCC and 30 control subjects. A single-slice dynamic CT was performed after an intravenous bolus injection of 50 ml of contrast material and continued for 50 seconds at a speed of 4.0 ml/s. The parameters were calculated by deconvolution method, and perfusion maps and quantitative parameters were created. Result(s): For HCC, the decreased parameters such as HBF, HBV, HPP and the increased parameters such as HAP, HAI in HCC were differentiated from the apparently normal liver adjacent to cancer and the normal liver region (P<0.05). All the parameters between the normal liver adjacent to cancer and normal liver region have no differences (P>0.05). Conclusion(s): Perfusion CT can provide quantitative information about arterial and portal blood flow of HCC. |
| Kang, C., Xu, R., Xu, F. The value of dynamic combined detection of serum tumor biomarkers in diagnosing primary hepatic carcinoma. Cancer Research and | Objective: To investigate the value of combined detection of serum tumor biomarkers including alpha-fetoprotein (AFP), tumor specific growth factor (TSGF), golgi protein 73 (GP73) and osteopontin (OPN) in diagnosis of primary hepatic carcinoma. Method(s): AFP, TSGF and GP73 levels were measured by electro-chemiluminescence immunoassay, and OPN levels by enzyme-linked immunosorbent assay (ELISA) in 122 cases of primary hepatic carcinoma, 50 cases of liver benign lesions and 50 cases of healthy control. The biological parameters and the levels of AFP, TSGF, GP73 and OPN were studied. Result(s): The serum levels of AFP, TSGF, GP73 and OPN |

| Ref | Abstract |
|---|---|
| Clinic 2014;26(8):531-38. | in primary hepatic carcinoma were higher than those in the liver benign disease group and the normal control group (all P < 0.01). Sex and age of the patients at diagnosis showed no significant association with the levels of the four serum tumor biomarkers in the primary hepatic carcinoma groups (all P > 0.05). But the tumor size, amount, tumor stage, metastasis and recurrence showed significant association with the levels of those in the primary hepatic carcinoma groups (all P < 0.05). The sensitivity, specificity and veracity of serum AFP, TSGF, GP73 and OPN as individual diagnostic marker was only 57.38 %, 68.85 %, 70.49 % and 69.67 %, respectively. The sensitivity of combined detection of AFP and TSGF was 80.33 %, and that of combined detection of AFP, TSGF and GP73 was 85.25 %. While, the sensitivity of the four serum tumor markers in combination was 98.36 %, the accuracy was 95.65 %. The sensitivity and accuracy of combined detection with the four serum tumor markers were significandy higher than those of the individual markers and other combination detection methods (all P < 0.05). Conclusion(s): Serum markers of AFP, TSGF, GP73 and OPN can serve as a means for diagnosis of primary hepatic carcinoma. Combined detection of the four serum tumor biomarkers can improve the sensitivity, accuracy and the negative predictive value, which is benefit to early diagnosis and interference. |
| Kim, Kyung-Ah, Lee, June Sung, Jung, Eun Sook, Kim, Jong Yeon, Bae, Won Ki, Kim, Nam-Hoon. [Usefulness of serum alpha-fetoprotein (AFP) as a marker for hepatocellular carcinoma (HCC) in hepatitis C virus related cirrhosis: analysis of the factors influencing AFP elevation without HCC development]. The Korean journal of gastroenterology = Taehan Sohwagi Hakhoe chi 2006;48(5):321-6. | BACKGROUND/AIMS: Serum alpha-fetoprotein (AFP) is frequently used for the diagnosis of hepatocellular carcinoma (HCC). Most available data concerning AFP came from studies of patients with chronic hepatitis B or mixed etiologies. Studies concerning the diagnostic value of AFP for HCV-related liver cirrhosis (LC) are limited. We evaluated the factors influencing AFP elevation in the absence of HCC and analyzed the diagnostic value of serum AFP in HCC surveillance of HCV-related LC patients., METHODS: We enrolled 55 patients of HCV-related LC with HCC and 62 patients without HCC as a case-control study were analyzed. The sensitivity and specificity were calculated and the clinical and biochemical factors influencing serum AFP levels., RESULTS: The sensitivities and specificities of serum AFP for the detection of HCC in HCV-related LC were 72.7% and 59.7% for AFP>or=20 ng/mL, and 47.3% and 92.5% for AFP>or=100 ng/mL, respectively. Elevated serum AST was independently associated with elevated serum AFP level in HCV-related LC. In cases of ASTor=100 ng/mL for the diagnosis of HCC was 100%. However, in case of AST>2 ULN, the specificity was 85.0% for AFP>or=100 ng/mL and 95.0% for AFP>or=200 ng/mL., CONCLUSIONS: Serum AST levels influence serum AFP level in HCV-related LC. In cases of AST2 ULN. |
| Kim, Myong Jin, Bae, Kang Woo, Seo, Pyoung Ju, Jeong, In Kook, Kim, Jung Hyuk, Lee, Bo Han, et al. [Optimal cut-off value of PIVKA-II for diagnosis of hepatocellular carcinoma--using ROC curve]. The Korean journal of hepatology 2006;12(3):404-11. | BACKGROUND/AIMS: Protein induced by vitamin K absence or antagonist-II (PIVKA-II), also known as des-carboxyprothrombin (DCP), can be used as an alternative tool to alpha-fetoprotein (AFP) for surveillance of hepatocellular carcinoma (HCC). The aims of the present study were to compare PIVKA-II levels between the patients with HCC and patients with non-HCC chronic liver disease, to evaluate the correlation of PIVKA-II and AFP in HCC patients, and finally to estimate the optimal cut-off value for PIVKA-II for the diagnosis of HCC with using the receiver operating characteristic (ROC) curve., METHODS: A total of 227 consecutive patients with HCC (n=42) or chronic liver disease (n=185) were enrolled in this study. HCC was diagnosed histologically or by imaging such as computed tomography, magnetic resonance imaging or angiography. The serum PIVKA-II and AFP levels were measured by electrochemiluminoimmunoassay with using the Haicatch PIVKA-II kit and by immunoradiometric assay, respectively., RESULTS: The PIVKA-II level in the HCC patients was significantly higher than the non-HCC chronic liver disease patients (903.0+/-1156.7 vs. 111.7+/-211.0 mAU/ mL, respectively, P<0.01). PIVKA-II and AFP showed a statistical correlation in HCC patients (r=0.46, P<0.01). The sensitivity and specificity of PIVKA-II for the diagnosis of HCC were 66.7% and 74.1%, respectively, and when tasted together with AFP, the sensitivity was increased by 85.7%. For the ROC curve of PIVKA-II in HCC patients, the specificity of a 250 mAU/mL level of PIVKA-II was 95%., CONCLUSIONS: PIVKA-II was as useful surveillance tool for differentiating HCC from chronic liver disease, and a PIVKA-II value of 250 mAU/ mL was proposed as a significant cut-off value for diagnosis of hepatocellular carcinoma. |
| Ko, Young Sun, Bae, Joo Hwan, Sinn, Dong Hyun, Gwak, Geum Youn, Kang, Wonseok, Paik, Yong Han, et al. [The Clinical Significance of Serum Alpha-fetoprotein in Diagnosing Hepatocellular Carcinoma in a Health Screening Population]. The Korean journal of gastroenterology = Taehan Sohwagi Hakhoe chi 2017;69(4):232-38. | BACKGROUND/AIMS: Serum alpha-fetoprotein (AFP) measurement is commonly included in a health check-up program in Korea. However, its benefits remain uncertain. We analyzed whether AFP measurement should be included in a general health check-up program to screen for hepatocellular carcinoma (HCC)., METHODS: A total of 36,552 adults aged 18 years or older-who participated in a routine health examination including AFP determination between January 2009 and December 2009 at the Health Promotion Center, Samsung Medical Center, South Korea-were analyzed. High risk of HCC was defined as positivity for hepatitis B surface antigen, anti-hepatitis C virus antibody or having liver cirrhosis., RESULTS: AFP level >10 ng/mL was observed in 27 participants (0.1%) and primary liver cancer was diagnosed in 9 patients (6 HCC and 3 cholangiocarcinoma). Among 1,619 participants with high risk factors of HCC, AFP level >10 ng/mL was observed in 16 participants, of which, 4 diagnoses were made. Sensitivity, specificity, positive predictive value, and negative predictive value of AFP for HCC was 0.66, 0.99, 0.25 and 0.99, respectively, for high risk participants. Among 34,933 participants without risk factors for HCC, 11 patients (<0.1%) showed elevated AFP levels above 10 ng/mL, and no case was diagnosed with primary liver cancer during a median follow-up period of 36 months (range: 0-48 months)., CONCLUSIONS: AFP elevation was rare in participants without risk factors for HCC, and was unable to screen for HCC in this population. We discourage routine AFP measurements for asymptomatic adults without risk factors of HCC. |

| Ref | Abstract |
|---|---|
| Li, Bao-ding, Zhao, Qing-chuan, Zhu, Yang-ting, Zhang, Fu-qin. [Significance of glypican-3 mRNA expression in hepatocellular carcinoma tissues and peripheral blood cells]. Zhonghua wai ke za zhi [Chinese journal of surgery] 2006;44(7):458-62. | OBJECTIVE: To investigate the expressions of glypican-3 (GPC3) mRNA in hepatocellular carcinoma (HCC) tissues and peripheral blood cells (PBCs), and to determine the values of GPC3 mRNA in the diagnosis of HCC and HCC micrometastasis., METHODS: Using semi-quantitative and nested reverse transcription polymerase chain reactions (RT-PCR), we detected the expressions of AFP and GPC3 genes in the tissues of 41 HCC, 41 paracancer and 52 non-HCC liver samples (41 far from HCC tissues and 11 normal liver tissues), and in the PBCs of 67 specimens from subjects., RESULTS: The semi-quantitative RT-PCR displayed GPC3 mRNA was expressed in all samples of tissues and PBCs, and the relative intensities of its expressions in HCC, paracancer, non-HCC liver tissues were 78.9 +/- 35.5, 30.6 +/- 21.6, 23.8 +/- 15.5 respectively. The AFP mRNA expression values were 61.2 +/- 32.6, 31.5 +/- 23.6, and 21.2 +/- 15.9 respectively. The expression of each gene in HCC differed significantly from those in other two kinds of tissue samples (P < 0.01). The expressions of GPC3 mRNA and AFP mRNA, accounting for 80.5% and 63.4% in all the HCC tissues, were higher than their respective peak values in the tissues of non-HCC liver (+1.96s), but the expressions of at least one of the two genes was elevated in 92.7% of all the HCC tissues. There was a significant difference between combined detection of two genes and single AFP mRNA detection in HCC tissues (P < 0.01). Clinicopathologically, AFP mRNA was related with the grade of HCC and serum AFP, while GPC3 mRNA was related with not only the grade of HCC but also the invasion of HCC. The relative intensities of GPC3 mRNA expressions in PBCs of 67 specimens was 15.9 +/- 9.0, and GPC3 mRNA expressed in three kinds of tissue samples were all stronger than its counterparts in PBCs (P < 0.01). The GPC3 mRNA expression values in PBCs of the HCC group and the non-HCC group were respectively 16.1 +/- 8.3, 15.6 +/- 10.2, there was no significant difference between the two groups. Of the HCC metastasis group and the HCC non-metastasis group, the respective GPC3 mRNA expression values in PBCs were 16.0 +/- 9.0 and 16.3 +/- 7.7, there was also no significant difference between the two groups. The nested RT-PCR showed that the positive rates of AFP mRNA expressions in PBCs from the HCC group and the non-HCC group were 56.1% and 23.1%, and the difference between the two groups was significant (P = 0.011). The positive rates of AFP mRNA expressions in PBCs from the HCC metastasis group and the HCC non-metastasis group were 80.9% and 30.0%, and there was also a significant difference between the two groups (P = 0.002)., CONCLUSIONS: Although GPC3 mRNA is expressed broadly, it still may serve as a potential tissue biomarker in the diagnosis of HCC. Detecting the expression of the two genes in the tissues will improve the screening and diagnosis of HCC. GPC3 is prevalently transcribed in the PBCs, but we have not found any relationship between the GPC3 expression in PBCs and the metastasis or recurrence of hepatocellular carcinoma, thus we can not identify HCC micrometastasis with GPC3 mRNA. |
| Li, D., Zhang, J. Z., Zheng, Y. H., Ji, X. L., You, H., Shu, Q. M., Fan, L. N. Using protein chip technology to screen for tissue proteomic profiles and tumor markers in hepatocellular carcinoma. World Chinese Journal of Digestology 2007;15(22):2424-30. | AIM: To use the surface-enhanced laser desorption and ionization time-of-flight mass spectrometry (SELDI-TOF-MS) technique to screen for tissue biomarkers in patients with hepatocellular carcinoma (HCC), but with different clinicopathological features. METHOD(S): Proteomic spectra were examined and analyzed by mass spectroscopy in 44 cases, including 26 specimens of HCC tissue that had been pathologically confirmed in patients aged 34-68 years, and 18 specimens of liver cirrhosis tissue in patients aged 38-70 years. The spectra obtained were analyzed using the biomarker wizard system, and the biomarkers were defined by searching www.ExPasy.org. RESULT(S): A total of 16 distinguished proteomic biomarkers, 7 up-regulated and 9 down-regulated, were detected from screening HCC tissue, in contrast with liver cirrhosis tissue. There were significant differences in the protein peaks of different molecular masses of 4.7, 7.2 and 9.8 kDa between HCC and liver cirrhosis tissues. Eleven distinguished proteomic biomarkers were screened when comparing cases of moderately and highly differentiated HCC tissue. All proteins were confirmed by searches of www.ExPasy.org. CONCLUSION(S): The SELDI-TOF-MS technique offers a unique platform for proteomic detection in HCC. It is also a non-invasive method for studying proteomic changes in the development and progression of HCC. |
| Li, Jie. [Significance of arginase-1, glypican-3, hepatocyte paraffin antigen 1 and alpha-fetoprotein in diagnosis and differential diagnosis of liver tumors]. Zhonghua bing li xue za zhi = Chinese journal of pathology 2014;43(4):246-50. | OBJECTIVE: To study the expression of arginase-1 (Arg-1), glypican-3 (GPC3), hepatocyte paraffin antigen 1 (HepPar-1) and alpha-fetoprotein (AFP) in hepatocellular carcinoma (HCC), benign liver lesions (BLL) and metastatic carcinoma (MC), and their applications in diagnosis and differential diagnosis., METHODS: Immunohistochemical study (EnVision method) for Arg-1, GPC3, HepPar-1 and AFP was carried out in three groups of liver lesions, including 85 cases of HCC, 35 cases of BLL and 19 cases of MC. The relationship between expression of Arg-1, GPC3, HepPar-1 and AFP and clinicopathologic features in HCC was also analyzed., RESULTS: The positive expression rate of Arg-1 was 90.6% (79/85) in HCC and 100% (35/35) in BLL. Arg-1 expression was observed in 1 of the 19 cases of MC studied. The positive expression rate of GPC3 was 82.4% (70/85) in HCC, 5.3% (1/19) in MC and 0 (0/35) in BLL. The positive expression rate of AFP was 47.1% (40/85) in HCC and 0 in BLL or MC. The positive expression rate of HepPar-1 was 72.9% (62/85) in HCC, 100% (35/35) in BLL and 2/19 in MC. Arg-1 has a higher sensitivity in highlighting hepatocellular lesions than AFP and HepPar-1 (P=0.000 versus P=0.002). The specificity of GPC3 expression in HCC was 98.1%., CONCLUSIONS: Arg-1 is a sensitive hepatocellular marker in delineation of liver lesions.GPC3 is a relatively specific marker in diagnosis of HCC. |
| Li, P., Zhai, Y., Liu, H., Lv, F. D., Li, N. Diagnostic value of serum AFP alone or in combination with glypican 3, VEGF or | AIM: To evaluate the diagnostic value of serum alpha-fetoprotein (AFP) alone or in combination with glypican 3 (GPC3), vascular endothelial growth factor (VEGF) or insulin-like growth factor-II (IGF-II) for patients with primary hepatocellular carcinoma (HCC). METHOD(S): Serum AFP, GPC3, VEGF and IGF-II were measured by enzyme-linked immunosorbent assay in 66 patients with histopathologically proven HCC and 16 cirrhotic patients. The cut-off level of AFP for diagnosis of HCC was 400 mug/L, while those of GPC3, VEGF and IGF-II were determined using |

| Ref | Abstract |
|---|---|
| IGF-II for patients with primary hepatocellular carcinoma. World Chinese Journal of Digestology 2010;18(25):2702-06. | ROC curve analysis. RESULT(S): The sensitivity of serum GPC3 for diagnosis of HCC was highest, followed by serum IGF-II , AFP and VEGF. The specificity of serum AFP for diagnosis of HCC was highest, followed by serum GPC3, VEGF and IGF-II. AFP in combination with GPC3 was most useful for diagnosis of HCC, especially early HCC. In AFP-negative HCC patients, the positive rate of GPC3 was 51.1%. In patients with early HCC (BCLC stage A), the positive rate of GPC3 was 50.0%. CONCLUSION(S): Detection of serum AFP (>=400 mug/L) alone has its imitations in the diagnosis of HCC. AFP in combination with GPC3 is most useful for diagnosis of HCC, especially early HCC. |
| Li, X., Li, Y., Li, X., Jiang, L. N., Zhu, L., Lu, F. M. [A preliminary discussion on carnosine dipeptidase 1 as a potential novel biomarker for the diagnostic and prognostic evaluation of hepatocellular carcinoma]. Zhonghua gan zang bing za zhi = Zhonghua ganzangbing zazhi = Chinese journal of hepatology 2023;31(6):627-33. | Objective: To explore carnosine dipeptidase 1 (CNDP1) potential value as a diagnostic and prognostic evaluator of hepatocellular carcinoma (HCC). Methods: A gene chip and GO analysis were used to screen the candidate marker molecule CNDP1 for HCC diagnosis. 125 cases of HCC cancer tissues, 85 cases of paracancerous tissues, 125 cases of liver cirrhosis tissues, 32 cases of relatively normal liver tissue at the extreme end of hepatic hemangioma, 66 cases from serum samples of HCC, and 82 cases of non-HCC were collected. Real-time fluorescent quantitative PCR, immunohistochemistry, western blot, and enzyme-linked immunosorbent assay were used to detect the differences in mRNA and protein expression levels of CNDP1 in HCC tissue and serum. Receiver operating characteristic (ROC) curves and Kaplan-Meier survival were used to analyze and evaluate the value of CNDP1 in the diagnosis and prognosis of HCC patients. Results: The expression level of CNDP1 was significantly reduced in HCC cancer tissues. The levels of CNDP1 were significantly lower in the cancer tissues and serum of HCC patients than those in liver cirrhosis patients and normal controls. ROC curve analysis showed that the area under the curve of serum CNDP1 in the diagnosis of HCC patients was 0.753 2 (95% CI 0.676-0.830 5), and the sensitivity and specificity were 78.79% and 62.5%, respectively. The combined detection of serum CNDP1 and serum alpha-fetoprotein (AFP) significantly improved the diagnostic accuracy (AUC = 0.820 6, 95% CI 0.753 5-0.887 8). The diagnostic sensitivity and specificity of serum CNDP1 for AFP-negative HCC patients were 73.68% and 68.75% (AUC = 0.793 1, 95% CI 0.708 8-0.877 4), respectively. In addition, the level of serum CNDP1 distinguished small liver cancer (tumor diameter < 3 cm) (AUC = 0.757 1, 95% CI 0.637 4-0.876 8). Kaplan-Meier survival analysis showed that CNDP1 was associated with a poor prognosis in HCC patients. Conclusion: CNDP1 may be a potential biomarker for the diagnostic and prognostic evaluation of HCC, and it has certain complementarity with serum AFP. |
| Liang, R., Luo, X. L., Ge, L. Y., Yuan, W. P., Yue, H. F., Jia, B. C. The diagnostic value of serum Golgi protein-73 in hepatocellular carcinoma. Tumor 2012;32(2):115-18. | Objective: To investigate the diagnostic value of serum Golgi protein-73 (GP73) in hepatocellular carcinoma (HCC). Method(s): The peripheral serum samples from 504 individuals were collected, including 144 HCC patients, 50 cirrhotic patients, 100 hepatitis B patients, 84 hepatitis B virus carriers, 50 patients with other malignancies, 26 patients with benign liver tumor, and 50 healthy volunteers. The expression levels of serum GP73 and alpha-fetal protein (AFP) were detected by double antibody sandwich ELISA and electrochemiluminescence assay, respectvely. Result(s): The expression level of serum GP73 from the HCC patients was significantly higher than those from the other patient groups (P <0.05). When the cut-off value was determined as 64 ng/mL by the receiver operating characteristic (ROC) curve, the sensitivity and specificity of GP73 for the diagnosis of HCC were both higher than those of AFP (83.3% vs 72.2%; 88.3% vs 76.7%; both P <0.05). The sensitivity of GP73 in combination with serum AFP for the diagnosis of HCC was 94.4%. Conclusion(s): The sensitivity and specificity of serum GP73 for the diagnosis of HCC are higher than those of serum AFP. The rate of HCC diagnosis is increased by combined examination of serum GP73 and AFP. Copyright Â© 2012 by TUMOR. |
| Liu, Hong-Dao, Ni, Run-Zhou, Xiao, Ming-Bing, Li, Li-Ren, Hua, Guo-Ping. [Clinical significance of serum GPDA-F determined by immunoelectrophoresis in diagnosis of hepatocellular carcinoma]. Ai zheng = Aizheng = Chinese journal of cancer 2006;25(2):247-49. | BACKGROUND & OBJECTIVE: Serum fast band of glycylproline dipeptidyl aminopeptidase isoenzyme (GPDA-F) is useful to the diagnosis of hepatocellular carcinoma, especially for the cases without expression of alpha-fetoprotein (AFP). Polyacrylamide electrophoresis for detection of GPDA-F is relatively complicated and has limitation in its clinical use. This study was to establish a simple and easy method of immunoelectrophoresis to detect serum GPDA-F, and evaluate clinical value of GPDA-F in the diagnosis of hepatocellular carcinoma., METHODS: Serum GPDA-F was purified to raise polyclonal GPDA-F antibody, and immunoelectrophoresis was established for the detection of serum GPDA-F. Serum GPDA-F in 99 specimens of hepatocellular carcinoma and 115 specimens of benign liver diseases (36 cases of liver cirrhosis, 23 cases of acute hepatitis, 38 cases of chronic hepatitis, and 18 cases of benign liver space-occupying lesions) was simultaneously detected by both polyacrylamide electrophoresis and immunoelectrophoresis. The clinical value of serum GPDA-F detected by immunoelectrophoresis was compared with that by polyacrylamide electrophoresis for the diagnosis of hepatocellular carcinoma., RESULTS: If the cut-off was set at 71 u/L, the diagnostic sensitivity, specificity, and accuracy of immunoelectrophoresis in detecting GPDA-F in hepatocellular carcinoma were 83.8%, 85.2%, and 84.6%, respectively; while those of polyacrylamide electrophoresis were 81.8%, 77.3%, and 79.4%, respectively., CONCLUSIONS: Serum GPDA-F detected by immunoelectrophoresis is useful to the diagnosis of hepatocellular carcinoma. Compared with polyacrylamide electrophoresis, this method is cheap, time-saving, and easy. |
| Liu, X., Lina, M., Xia, L., Tang, Y., Liu, S. Diagnostic value of liver stiffness measurement combined with serum high-sensitivity C-reactive protein detection in HBV-related | Objective: The aim of this study was to explore the diagnostic value of liver stiffness measurement combined with serum high-sensitivity C-reactive protein detection in HBV-related cirrhosis patients complicated with primary liver cancer. Method(s): A total of 156 previously untreated chronic hepatitis B-related cirrhosis patients and 50 healthy subjects were included in this study. The 156 patients were divided into two groups: those with primary liver cancer (67 cases) and without liver cancer (89 cases). The 50 healthy subjects were considered as normal control group. Liver stiffness measurement (LSM) was conducted and serum high-sensitivity C-reactive protein (CRP) level was assayed in all the 156 patients and 50 normal individuals, and their measurement values |

| Ref | Abstract |
| --- | --- |
| cirrhosis patients complicated with primary liver cancer. Chinese Journal of Oncology 2015;37(2):119-22. | were statistically compared and analyzed. Result(s): The ISM value was (39. 72 +/- 29.05) kPa in the liver cancer patients, significantly higher than the (27. 81 +/- 18. 46) kPa in the cirrhosis alone patients and (4. 25 +/- 0. 74) kPa in the healthy controls (P<0.01 for both). Serum hs-CRP levels in the liver cancer patients was 5.81mg/L, significantly higher than 1.78 mg/L in the cirrhosis alone patients and 0. 38mg/L in healthy controls, (P < 0.01 for both). The higher the grade of LSM values was, the positive rate of CRP was higher in the cirrhosis patients complicated with primary liver cancer. In patients with LSM values >27. 6 kPa, the serum CRP positive rate was 64.2% in patients with primary liver cancer, significantly higher than the 38. 0% in patients with cirrhosis alone (P<0.01). In the 67 HBV-related cirrhosis patients complicated primary liver cancer, the LSM value and serum hs-CRP level in AFP-positive patients were (48. 95 +/-28. 59) kPa and 4. 91 mg/L, respectively, higher than those in the AFP-negative patients (28. 64 +/-26. 83) kPa and 4. 16 mg/L, but with a nonsignificant difference (P >= 0. 05). Conclusion(s): Liver stiffness measurement combined with serum high-sensitivity C-reactive protein detection may have potential diagnostic implications as a marker of primary liver cancer occurrence in patients with HBV-related cirrhosis. |
| Lu, F., Chen, H., Gao, M. Clinical value of des-gamma-carboxy prothrombin in the diagnosis of hepatocellular carcinoma. Chinese Journal of Clinical Oncology 2009;36(7):361-64. | Objective: To study the clinical value of the serum levels of des-gamma-carboxy prothrombin (DCP) in the diagnosis of hepatocellular carcinoma. Method(s): A total of 172 patients were enrolled in this study, including 25 normal controls, 20 cases of chronic hepatitis, 51 cases of liver cirrhosis and 76 cases of HCC. Serum DCP levels were determined by ELISA and AFP levels were determined by ECLIA. The diagnostic value of DCP and AFP in distinguishing HCC from chronic nonmalignant diseases and the relationship of serum level of DCP and AFP with the clinicopathologic factors of hepatocellular carcinoma were analyzed. Result(s): The mean serum concentrations of DCP in normal controls, patients with chronic hepatitis, patients with liver cirrhosis and patients with HCC were 17.72+/-9.59, 26.12+/-12.64, 37.45+/-18.26, and 806.71+/-639.79 (mAU/ml), respectively. The mean serum concentrations of AFP were 7.93+/-5.42, 14.59+/-11.91, 16.29+/-14.10, and 547.47+/- 544.98 (ng/ml) in the above four groups, respectively. Both DCP and AFP levels in HCC patients were significantly higher than those in the other three groups. DCP levels showed an increasing trend in normal controls, patients with chronic hepatitis, patients with liver cirrhosis and patients with HCC. The sensitivity of DCP was higher than AFP in diagnosing HCC (78.95% vs 73.68%). The positive rate was 89.47% when serum DCP was used in combination with AFP, significantly higher than those obtained through each factor independently. In 76 patients with HCC, the DCP concentration with large HCC tumors (>5cm), multi-tumors and PVI-positive tumors was significantly higher than that in small tumors and PVI-negative ones (P<0.05). The AFP levels in patients with positive HBSAg were significantly higher than those in patients with negative HBSAg. Conclusion(s): DCP has a higher diagnostic value than AFP in differentiating HCC from nonmalignant chronic liver disease. Serum DCP levels were correlated with clinicopathologic factors including tumor size and tumor PVI. AFP and DCP can complement each other in the diagnosis of HCC. |
| Lv, F., Gao, Y. F., Rao, J. G., Zhang, W., Zou, G. Z. Clinical characteristics of patients with hepatitis B virus related liver cirrhosis and primary liver cancer. World Chinese Journal of Digestology 2015;23(17):2798-8004. | AIM: To investigate the clinical characteristics of hepatitis B virus (HBV) related liver cirrhosis patients with primary liver cancer (PLC), in order to provide evidence for the prevention and early diagnosis of PLC. METHOD(S): A total of 187 patients with HBV related liver cirrhosis and PLC and 318 patients with HBV related liver cirrhosis only were included in this study. Serological markers of HBV (HBV-M) were detected using enzyme linked immunosorbent assay (ELISA). HBV DNA was detected by quantitative polymerase chain reaction (PCR), and liver function was detected using an automatic biochemistry analyzer. The difference in clinical features between the two groups of patients was analyzed statistically. RESULT(S): In the PLC group, there were 156 men and 31 women, the proportion of male patients was higher than that of female patients (5.03:1), and the percentage of patients more than 40 years old was higher (87.83%). The main serological pattern in the PLC group was hepatitis B surface antigen (HBsAg), hepatitis B e antibody (HBeAb) and hepatitis B core antibody (HBcAb) positivity. HBV DNA positive rate in the PLC group was significantly lower than that in the HBV related liver cirrhosis alone group (75.31% vs 85.85%, P < 0.05). HBV DNA level in the PLC group was also significantly lower than that in the HBV related liver cirrhosis alone group (4.73 log10IU/mL +/- 1.26 log10IU/ mL vs 5.47 log10IU/mL +/- 1.35 log10IU/mL, P < 0.05). The difference in the hepatitis B e antigen (HBeAg) conversion rates between <40, 40-60 and >60 years old groups had no statistical significance (P > 0.05). The differences in alanine aminotransferase (ALT), total bile acid (TBA), total bilirubin (TB), globulin (GLB), gamma-glutamyl transpeptidase (GGT), alkaline phosphatase (ALP) and alpha-fetoprotein (AFP) levels were statistically significant between the two groups (P < 0.05). CONCLUSION(S): HBV related liver cirrhosis patients who are male, more than 40 years old, HBeAb positive or have a low level of HBV DNA should be paid more attention for early screening of primary liver cancer. The combined detection of GGT, ALP and AFP could facilitate the early diagnosis of PLC.Copyright Â© 2015 Baishideng Publishing Group Inc. All rights reserved. |
| Ma, Q. Q., Huang, J. T., Tong, H. B. Glypican-3, a novel tumor marker in diagnosis of human hepatocellular carcinoma. Chinese Journal of Cancer Prevention and Treatment 2014;21(2):127-32. | OBJECTIVE: To explore the clinical value of combined detection of serum human GlyPican-3 and alpha-fetoprotein (AFP) in the diagnosis of hepatocellular carcinoma. METHOD(S): The peripheral serum samples from 218 individuals were collected, including 54 HCC patients, 36 cirrhotic patients, 40 hepatitis patients, 18 patients with benign liver tumor, 40 patients with other malignancies, and 30 healthy volunteers. GlyPican-3 levels in the serum were detected by double antibody sandwich ELISA, and AFP levels in serum were detected by the electrochemiluminescence immunoassay. Those results were shown with median level. Accuracy of the diagnosis was evaluated by the area under the receiver operating characteristic curve (ROC-AUC). RESULT(S): The expression level of serum GlyPican-3 from the HCC patients was significantly higher than those from the other patient groups (all, P<0.05). AUC was 0.883 (95% |

| Ref | Abstract |
|---|---|
| | CI: 0.833-0.923) for GlyPican-3, 0.802 (95% CI: 0.743-0.853) for AFP, 0.945(95% CI: 0.905-0.971) for GlyPican-3+AFP, respectively. AUC was the highest for GlyPican-3+AFP, significantly greater than both AFP and GlyPican-3 (Z=5.944, P=0.007 and Z=3.409, P=0.007). When the cut-off value was determined as 60 rhog/mL, the sensitivity and specificity of GlyPican-3 for the diagnosis of HCC were 85.18% (46/54) and 73.78% (121/164). The sensitivity of GlyPican-3 in combination with serum AFP for the diagnosis of HCC was 96.29%(52/54). The expression level of serum GlyPican-3 was markedly increased with the increase of clinical stages (H=4.571, P=0.025). CONCLUSION(S): The expression level of serum GlyPican-3 has a high diagnostic value for HCC patients. The rate of HCC diagnosis is increased by combined examination of serum GlyPican-3 and AFP. The expression level of serum GlyPican-3 in HCC is related to the clinical staging. |
| Malov, S. I., Malov, I. V., Dvornichenko, V. V., Rasulov, R. I., Kuvshinov, A. G., Marche, P. N., et al. [Application of alpha-fetoprotein and osteopontin combination for early diagnosis of hepatocellular carcinoma associated with hepatitis C.]. Klinicheskaia laboratornaia diagnostika 2019;64(10):607-12. | Liver cirrhosis in the outcome of hepatitis C is the leading cause of hepatocellular carcinoma (HCC) in the world. Early diagnosis and timely treatment of HCC are important for reducing mortality and increasing life expectancy of patients with hepatocellular carcinoma. To assess the risk of HCC, the definition of alpha-fetoprotein (AFP) in the blood is most widely used, but low sensitivity limits its diagnostic value. In 2012, a new HCC biomarker - osteopontin (OPN), which is a secreted phosphoprotein that has a high affinity for integrins was proposed. The level of acute renal failure begins to rise in the early stages of malignancy, before the period of HCC detection by imaging methods, and has significantly better sensitivity than AFP. The purpose of this study is to evaluate the diagnostic efficacy of the combined determination of alpha-fetoprotein and osteopontin in prospective monitoring of patients with chronic hepatitis C in the advanced phase of liver fibrosis. Monitoring of 588 patients with hepatitis C was carried out from February 2013 to February 2019. HCC was detected in 55 of them (2.6% per year). The combination of 2 biomarkers showed better diagnostic efficacy than alpha-fetoprotein and osteopontin separately: AUC 0.85 (95% CI 0.80-0.90) versus AUC 0.63 (95% CI 0.57-0, 70) and AUC 0.82 (95% CI 0.77-0.88), respectively. This combination showed a sensitivity of 85.5% and made it possible to diagnose HCC with a prognostic level of a positive result of 72.3% at 19,4+/-0,8 weeks before the diagnosis was confirmed by instrumental imaging methods (ultrasound, MRI, CT). In the combined variant, ARF made the greatest contribution to the increase in diagnostic efficacy (AUC). At an early and very early stage of HCC development, isolated HCC elevations were found in only 5.4% of patients. Conclusion : the combined use of alphafetoprotein and osteopontin as a diagnostic panel can be recommended for monitoring patients with liver cirrhosis in the outcome of hepatitis C and predicting HCC at an early stage of development. |
| Malov, S. I., Yushchuk, N. D., Malov, I. V., Dvornichenko, V. V., Rasulov, R. I., Marche, P. N., et al. A study of serum miRNA-122 in hepatitis C and associated hepatocellular carcinoma. Vestnik Rossiiskoi Akademii Meditsinskikh Nauk 2019;74(6):388-95. | BACKGROUND: The discovery of a cluster of short non-coding RNAs called microRNAs (miRNAs) has become an important event in molecular biology. One of its representatives, miR-122 plays a large role in regulating the expression of genes involved in carbohydrate, lipid metabolism, and iron metabolism in the body. In experimental studies it was shown that in addition to regulatory functions, miR-122 is involved in the pathogenesis of hepatitis C, providing the life cycle of the virus in the cell. The shift of emphasis in the study of miR-122 from basic research into clinical medicine seems to be a promising area of personalized medicine. AIMS: to determine the clinical significance of miR-122 in acute and chronic hepatitis C and associated hepatocellular carcinoma. MATERIALS AND METHODS: A total of 407 people were examined, including 17 patients with acute hepatitis C (AHC), 158 patients with chronic hepatitis C (CHC) and 62 patients with HCC associated with hepatitis C. Comparison groups consisted of 84 healthy individuals and 62 patients with clinically pronounced cirrhosis of a non-infectious aetiology. In each cohort, the relative miR-122 level was determined in the blood of patients. The analysis was performed in PCR using the Qubit microRNA Assay Kit -100 for the quantitative determination of microRNAs (Thermo Fisher Scientific, USA). Relative miR-122 expression values were calculated by the formula 2 -DELTADELTACT using U6 snRNA as a reference RNA. RESULT(S): The highest miR-122 level in serum was found in patients with AHC at the height of the icteric period. The level of miR-122 showed a direct correlation with the activity of hepatic transaminases in patients with AHC (r = 0.72) and CHC (r = 0.44). An analysis of miR-122 level relative to the degree of liver fibrosis in patients with chronic hepatitis C showed that, as liver fibrosis progresses, the level of miR-122 expression decreases. The decrease in miR-122 expression in patients with severe fibrosis was universal and did not depend on the aetiology of the disease. The development of HCC in the presence of chronic hepatitis C was accompanied by a decrease in the level of miR-122 by 10 times on average compared to patients with chronic hepatitis C. CONCLUSION(S): The determination of the expression level of miR-122 in serum can be used in laboratory monitoring of the management of patients with HC as an indicator of the severity of liver damage in AHC and the rate of formation of liver fibrosis in CHC. Evaluation of possibility of using miR-122 as a predictor of the development of HCC in the outcome of HC requires additional studies of the specificity and sensitivity of the test and comparison of the obtained data with the results of using generally accepted protein tumor markers.Copyright Â© The article is licensed by CC BY-NC-ND 4.0 International Licensee https://creativecommons.org/licenses/by-nc-nd/4.0/ |
| Mao, Y. L., Yang, H. Y., Xu, H. F., Sang, X. T., Lu, X., Yang, Z. Y., et al. Significance of Golgi glycoprotein 73, a new tumor marker in diagnosis of hepatocellular | Objective: To evaluate the sensitivity and specificity of Golgi glycoprotein 73 (GP73) for the diagnosis of hepatitis B related hepatocelluar carcinoma (HCC). Method(s): Western blotting was used to detect the serum GP73level in 25 patients being HBV carrier, 24 HCC patients, 12 patients with non-liver disease, and 99 healthy controls. Serum alpha-fetoprotein (AFP) was detected by electrochemiluminescence reaction. The levels of sensitivity and specificity of serum GP73 in diagnosing HCC were compared with those of AFP. The serum GP73 levels of some HCC patients during the perioprative period were compared. Result(s): The serum GP73 level of the HCC patients, all HBV positive, was (40.36 +/- 64.43) relative units, significantly higher than |

| Ref | Abstract |
|---|---|
| carcinoma: A primary study. National Medical Journal of China 2008;88(14):948-51. | those of the HBV carriers, non-liver patients, and healthy controls [(7.82 +/- 10.72), (4.48 +/- 5.70), and (2.59 +/- 5.12) relative units respectively, all P < 0.01]. There was no difference of GP73 levels between the healthy controls and the patients of non liver diseases (P = 0.2925). The sensitivity of GP73 for the diagnosis of HCC was 76.9%, significantly higher than that of AFP (48.6%). The specificity for the diagnosis of HCC of GP73 was 92.9%. Findings in a few HCC patients showed that the GP73 level remained not remarkably lowered within a week after surgical resection; but became lower 1.5 - 2 years after surgery. There was no raise of GP73 in the patients with non-malignant liver lesions. The GP73 levels of 4 of the 6 intra-hepatic cholangiocarcinoma patients were between those of the HCC patients and HBV carriers. Conclusion(s): Serum GP73 has higher sensitivity and specificity in diagnosis of hepatitis B-related HCC than AFP, and it can become a new effective HCC tumor marker. |
| Ogawa, K., Suzuki, K., Nakai, M., Sho, T., Suda, G., Morikawa, K. Evaluation of clinical utility of PIVKA-II using a chemiluminescent immunoassay. Acta Hepatologica Japonica 2019;60(11):397-404. | PIVKA-II is a tumor marker highly specific for hepatocellular carcinoma. We investigated the utility of Architect PIVKA-II, a chemiluminescent immunoassay, in 168 patients with liver disease (chronic hepatitis, n = 29; liver cirrhosis, n = 28; and hepatocellular carcinoma by stage: stage 1, n = 29; stage 2, n = 29; stage 3, n = 26; and stage 4, n = 27). Architect PIVKA-II was measured in preserved serum and compared with Lumipulse PIVKA-II and alpha fetoprotein (AFP) values measured that had been measured during patient evaluation. Both methods indicated increasing PIVKA-II levels with each higher stage of hepatocellular carcinoma. The diagnostic accuracy when combined with AFP was equivalent. Architect PIVKA-II has a diagnostic accuracy comparable to conventional tests in cases of hepatocellular carcinoma and should be useful in clinical practice.Copyright Â© 2019 The Japan Society of Hepatology. |
| Ouedraogo, W., Tran-Van Nhieu, J., Baranes, L., Lin, S. J., Decaens, T., Laurent, A., et al. [Evaluation of noninvasive diagnostic criteria for hepatocellular carcinoma on pretransplant MRI (2010): correlation between MR imaging features and histological features on liver specimen]. Evaluation des criteres diagnostiques non invasifs du carcinome hepatocellulaire sur IRM pre-greffe hepatique (2010) : correlations IRM - anatomopathologiques sur explants hepatiques. 2011;92(7-8):688-700. | PURPOSE: To validate the 2010 diagnostic criteria from the American Association for the Study of Liver Diseases (AASLD) for hepatocellular carcinoma (HCC) on MRI using the surgical liver specimen as a gold standard., PATIENTS AND METHODS: A total of 21 liver transplant recipients were retrospectively included. Each underwent surgery because of HCC between January 2007 and January 2008. Pre-transplant MRI was performed on a 1.5 Tesla MR unit. The T1W and T2W signal and kinetic contrast enhancement were correlated for each lesion with the surgical specimen. Lesion diameters between MRI and specimen were compared (Spearman). A multivariate model was created (R statistics software package) to predict the presence and grade of tumor differentiation (WHO, Edmonson Steiner)., RESULTS: A total of 71 nodules were detected at histology, including 54 HCC (mean size: 25.3mm) compared to 68 on MRI. There was moderate agreement (r=0.58, P<0.001) between the maximum lesion diameters measured on MRI and at histology. Wash-out on MRI provided an accuracy of 75 % for the detection of HCC (sensitivity=75 %, specificity=76 %). Adding T2W hyperintensity to the AASLD criteria increased the sensitivity of MRI from 70.3 % to 77.7 % for the diagnosis of HCC and from 67.6 % to 79 % for nodules less than 20mm in diameter, without affecting specificity. On multivariate analysis, wash out as a single variable was significantly associated with a diagnosis of HCC (P<0.01, odds ratio 12.0, CI 95 % [2.6-55.5]). T1W hyperintensity (P=0.04, odds ratio 5.4) and loss of signal on opposed-phase images (P=0.02, odds ratio 9.2) were predictive of good differentiation., CONCLUSION: On MRI, the AASLD criteria or presence of wash out within a liver nodule in patients with underlying chronic hepatocellular disease are suggestive of tumoral transformation. The addition of T2W hyperintensity to the AASLD criteria increases the detection of HCC, especially nodules smaller than 20mm. Copyright Â© 2011 Elsevier Masson SAS and Editions francaises de radiologie. All rights reserved. |
| Pei, M. M. & Lu, M. Clinical significance of serum miR-888-5p in patients with primary hepatocellular carcinoma. World Chinese Journal of Digestology 2020;28(7):247-53. | Background: MiR-888-5p is highly expressed in hepatocellular carcinoma (HCC) tissues and cell lines, and it can promote tumor invasion and metastasis and is related to disease stage and poor prognosis. However, the clinical value of serum miR-888-5p levels in the diagnosis and prognosis of HCC remains unclear. Aim(s): To detect serum miR-888-5p in patients with HCC and investigate its diagnostic value and correlation with clinical characteristics of HCC. Method(s): A total of 197 subjects were enrolled, consisting of 68 HCC patients, 46 chronic hepatitis B (CHB) patients, 43 liver cirrhosis (LC) patients, and 40 healthy volunteers. Serum miR-888-5p levels were measured by quantitative real-time polymerase chain reaction (qRT-PCR). The diagnostic value of miR-888-5p and its correlation with clinical features were assessed. Result(s): Compared with CHB patients, LC patients, and healthy controls, serum miR-888-5p increased significantly in HCC patients (P < 0.05). Receiver operating characteristic curve (ROC) analysis demonstrated that the area under the ROC curve (AUC) of miR-888-5p combined with alpha-fetoprotein (AFP) in discriminating HCC patients from healthy controls was 0.907 (sensitivity: 91.18%; specificity: 72.50%), higher than that of either AFP (AUC = 0.819; sensitivity: 73.53%; specificity: 97.50%) or miR-888-5p alone (AUC = 0.737; sensitivity: 79.41%; specificity: 62.50%). Serum miR-888-5p maintained its diagnostic efficiency in AFP negative HCC patients with an AUC of 0.793 (sensitivity: 90.90%; specificity: 62.50%). Serum miR-888-5p levels were significantly associated with pulmonary metastasis (P=0.01) of HCC. Conclusion(s): Serum miR-888-5p has high diagnostic value for HCC, and combined detection of serum miR-888-5p and AFP could improve the diagnostic efficiency. MiR-888-5p also has good diagnostic value for AFP negative HCC, and it is closely related to lung metastasis of HCC. MiR-888- 5p is expected to become a new serum marker for early diagnosis and prognosis evaluation of HCC.Copyright Â© 2020 The Author(s). |
| Qiu, L. W., Wu, W., Sai, W. L., Yang, J. L., Zhang, H. J., Gu, X., et al. Diagnostic value of expression of micro RNA-183 family | AIM: To investigate the expression of microRNA- 183 family members in hepatocellular carcinoma (HCC) and to analyze their diagnostic value. METHOD(S): The expression of miR-183 family members (miR-96, miR-182 and miR-183) was examined by real-time reverse transcriptionquantitative polymerase chain reaction (RTqPCR) in HCC and matched tumor-adjacent and tumor-distant tissues, and in 80 serum samples from patients with HCC, cirrhosis or chronic hepatitis B, and health controls. RESULT(S): The levels of miR-96 and miR-182 |

| Ref | Abstract |
|---|---|
| members in hepatocellular carcinoma. World Chinese Journal of Digestology 2013;21(16):1487-92. | expression in tissues and sera of HCC patients were significant higher than those in any of other groups (all P < 0.001). The level of miR-183 in the liver was significantly lower in HCC than in matched tumor-adjacent and tumor-distant tissue (both P < 0.001), although there was no significant difference in serum miR-183 between each group. Liver expression of miR-183 family members was positively associated with their serum levels (rmiR-96 = 0.815, rmiR-182 = 0.806, rmiR-183 = 0.851) in HCC. The areas under the receiver operating characteristic curve of serum miR-96 and miR-182 were 0.927 and 0.940. The relative level of serum miR-183 was significantly higher in patients with extrahepatic metastasis than in those without extrahepatic metastasis (P < 0.019). CONCLUSION(S): Expression of miR-96 and miR-182 is up-regulated and that of miR-183 down-regulated in HCC. Circulating miR-96 and miR-182 are useful biomarkers for HCC diagnosis. Down-regulation of miR-183 is associated with HCC extrahepatic metastasis. Â© 2013 Baishideng. All rights reserved. |
| Song, W., Wu, J., Wu, L. Signifi cance of serum lncRNA-PVT1 expression in diagnosis and prognosis of hepatocellular carcinoma. Journal of Practical Oncology 2020;35(4):317-21. | Objective: To investigate the expression and the significance of long non-coding RNA (lncRNA) PVT1 in the diagnosis and prognosis of hepatocellular carcinoma (HCC) patients. Method(s): Serum expression of lncRNA-PVT1 in 94 HCC patients with hepatitis B cirrhosis and 52 hepatitis B cirrhosis patients were detected by qRT-PCR. The relationship between lncRNA-PVT1 expression and clinicopathological characteristics of HCC patients was analyzed. The prognostic factors in HCC patients were analyzed by the Cox regression method. Receiver operating characteristic (ROC) curve was used to analyze the area under curve (AUC) of alpha-fetoprotein (AFP) and/or lncRNA-PVT1 and compared the sensitivity of lncRNA- PVT1 in the early diagnosis of HCC. Result(s): The serum expression of lncRNA-PVT1 in HCC patients with hepatitis B cirrhosis was significantly higher than that in hepatitis B cirrhosis patients [(4.34+/-0.25) vs (3.06+/-0.23), P=0.001]. The expression level of serum lncRNA-PVT1 in HCC patients was closely correlated with AFP level, TNM stage, BCLC stage and venous invasion (all P<0.05), but not related to age, sex, tumor size and tumor capsule (all P>0.05). Multivariate regression analysis demonstrated that TNM stage, vascular invasion and lncRNA-PVT1 were independent prognostic factors of HCC patients (all P<0.05). ROC AUC of lncRNA-PVT1 combined with AFP was 0.940 while AFP alone and lncRNA-PVT1 alone was 0.824 and 0.916, respectively. Conclusion(s): The expression of serum lncRNA-PVT1 in HCC patients is up-regulated and is closely related with the occurrence and progression of HCC, which is an effective biomarker for early diagnosis and prognosis monitoring in HCC patients.Copyright Â© 2020, The Second Affiliated Hospital, College of Medicine, Zhejiang University.. All right reserved. |
| Suarez-Munoz, Miguel Angel, Leiva-Vera, Maria Carmen, Santoyo-Santoyo, Julio, Fernandez-Aguilar, Jose Luis, Perez-Daga, Jose Antonio, Sanchez-Perez, Belinda, et al. [Detection of neoplastic lesions in cirrhotic patients waiting for liver transplantation]. Deteccion de lesiones neoplasicas en pacientes cirroticos candidatos a trasplante hepatico. 2006;80(3):157-61. | INTRODUCTION: Hepatocellular carcinoma is a frequent complication of cirrhosis. Liver transplantation is a valid therapeutic option for this disease providing that certain morphologic selection criteria (Milan criteria) are fulfilled., OBJECTIVE: To evaluate the accuracy of pretransplantation imaging examinations in the detection and characterization of neoplastic lesions in cirrhotic candidates for liver transplantation., PATIENTS AND METHOD: We performed a retrospective study of 250 cirrhotic patients who underwent liver transplantation. The preoperative radiological diagnosis was compared with the definitive pathological diagnosis, allowing the diagnostic sensitivity of the different techniques, as well as the degree of agreement between pre- and postoperative tumoral staging, to be identified., RESULTS: Analysis of 250 specimens from total hepatectomy identified 58 patients with hepatic tumors, with a total of 136 nodules. Fifty-three patients had hepatocarcinoma, nine of which were found incidentally. There were six radiological false positive diagnoses. Sixty-two percent of patients with hepatocarcinoma had multiple lesions. The most sensitive technique for the diagnosis of tumors smaller than 1 cm was magnetic resonance imaging. Agreement between pre- and postoperative staging was found in only 63.6% of cases; consequently, 43% of the patients who exceeded the Milan criteria (20% stage T3 and 23% stage T4a) underwent transplantation., CONCLUSIONS: Currently used imaging techniques lead to a substantial proportion of incorrect stagings in terms of the size and number of lesions in cirrhotic patients. |
| Sun, Z. H., Feng, Y. L., Li, L. H., Yang, Y. Q. Plasma cell-free DNA level and integrity as biomarkers to diagnose hepatocellular carcinoma. Medical Journal of Chinese People's Liberation Army 2016;41(12):1016-19. | Objective To detect the circulating cell-free DNA (cf-DNA) level and integrity in plasma of the patients with hepatocellular carcinoma (HCC), HBV and healthy controls by real time PCR (RT-PCR), evaluate their diagnostic value in hepatocellular carcinoma. Methods Venous blood samples from 52 HCC patients, 58 HBV patients and 60 healthy controls were collected. The levels of glyceraldehyde-3-phosphate dehydrogenase (GAPDH), beta-actin400 and beta-actin167 in plasma cf-DNA and the integrity of cf-DNA (reflected by beta-actin400/beta-actin167) were determined by RT-PCR. The differences in different groups and between two groups were analyzed with SPSS software. Results The beta-actin167 levels in plasma cf-DNA and the integrity of cf-DNA were significantly lower in healthy controls than in HCC patients (Z=-4.328, P=0.000; Z=-3.885, P=0.001) and HBV patients (Z=-2.473, P=0.005; Z=-3.295, P=0.013), and the integrity of cf-DNA was obviously lower in HBV patients than in HCC patients (Z=-4.836, P=0.002). Conclusion The integrity of circulating cf-DNA beta-actin167 and beta-actin400 detected by RT-PCR may be auxiliary biomarker in diagnosis of hepatocellular carcinoma.Copyright Â© 2016, People's Military Medical Press. All rights reserved. |
| Tian, Z. B., Liu, H., Sun, G. R., Kong, X. J., Zhang, C. P. Application of surface enhanced laser desorption ionization time-of-flight mass spectrometry technology in the | AIM: To explore tumor markers for the diagnosis of hepatocellular carcinoma (HCC) through detecting the serum protein spectrum differently expressed between hepatitis B virus (HBV) carriers and HCC patients. METHOD(S): We detected the serum protein spectrum in 27 HCC patients, 27 HBV carriers and 25 healthy controls using surface enhanced laser desorption ionization time-of-flight mass spectrometry (SELDI-TOF-MS) technique, and the diagnosis model was established through analyzing the detected data by biomarker patterns software (BPS) 5.0. RESULT(S): The protein peaks, which could discriminate HBV carriers from HCC patients and healthy individuals, as well as healthy individuals from HCC patients, were detected. A diagnosis |

| Ref | Abstract |
|---|---|
| diagnosis of hepatocellular carcinoma. World Chinese Journal of Digestology 2006;14(25):2499-5003. | model based on the detected data was established with the specificity of 93%, 96%, 84%, and sensitivity of 85%, 96%, 89%, respectively. In addition, the 8141-Da protein in HCC patients had a higher expression than that in HBV carriers (P < 10-5); the expression of 3448-Da protein was higher both in HCC patients and HBV carriers than that in healthy controls (P < 10-5), but it had no significant difference between HCC patients and HBV carriers (P > 0.05), indicating that 3448-Da protein might be a potential marker for HBV infection; 7771-Da protein was differently expressed between the three groups of patients. CONCLUSION(S): With a high specificity and sensitivity, the detection of serum protein spectrum can be performed easily and quickly by SELDI-TOF-MS technique, which provides a serological way for the diagnosis of HCC. |
| Tong, H. B., Ting, Y. J., Luo, L. M. Value of alpha fetoprotein, Dickkopf1 glycoprotein and alpha-L - Fucosidase in early diagnosis of liver cancer. World Chinese Journal of Digestology 2014;22(24):3670-74. | Aim: To investigate the value of alpha fetoprotein (AFP), Dickkopf1 glycoprotein (DKK1) and alpha-L-fucosidase (AFU) in the early diagnosis of hepatocellular carcinoma (HCC)., Methods: One hundred and three patients with primary HCC, 60 patients with liver cirrhosis, 127 patients with chronic hepatitis B, and 236 healthy people were included in the study. AFP, DKK1 and AFU were detected and compared among the four groups. The value of these markers in the diagnosis of primary HCC was evaluated., Results: Serum levels and abnormal rates of AFP, DKK1 and AFU were significantly higher in the HCC group than in the other groups (P < 0.05), and in the liver cirrhosis group than in the chronic hepatitis B group and control group (P < 0.05). Combined detection of AFP, DKK1 and AFU in the diagnosis of HCC had a sensitivity of 89.3%, a Youden index of 0.789, and a negative predictive value of 97.2%. The diagnostic accuracy of combined detection was significantly improved compared to the detection of any single index., Conclusion: Combined detection of AFP, AFU and DKK1 has a high sensitivity in the diagnosis of primary HCC and can reduce the rate of missed diagnosis.Copyright Â© 2014 Baishideng Publishing Group Inc. All rights reserved. |
| Tong, L., Gao, Z., Huang, C., Feng, H., Sun, X., Ji, J., et al. Role of GALAD serological model in the clinical diagnosis of primary hepatocellular carcinoma. Chinese Journal of Laboratory Medicine 2019;42(12):1037-41. | Objective: To explore the value of GALAD model, including gender, age, AFP, AFP-L3 and DCP in diagnosis of primary hepatocellular carcinoma and prediction of microvascular invasion (MVI). Method(s): Using retrospective study method, 5 919 patients with primary hepatocellular carcinoma (HCC) who received radical operation from January 2015 to December 2018 in Eastern Hepatobiliary Surgery Hospital were enrolled into study group. At the same time, 1 745 patients with benign liver diseases (BLDs) were enrolled into control group. The concentration of DCP was detected by Lumipulse G1200 automatic immune analyzer, and the concentration of AFP was detected by Cobas e601 automatic immune analyzer. AFP-L3 was detected by affinity adsorption centrifugation. The non-parametric Mann Whitney test was used to compare the difference between two groups. The chi square test was used to compare the rates. The diagnostic value of single serological marker and GALAD model for primary hepatocellular carcinoma was analyzed. The predictive effect of GALAD model on MVI of primary hepatocellular carcinoma was evaluated. Result(s): Compared with single serum marker, the diagnostic value of GALAD model is higher. When the cutoff value is -0.33, the diagnostic sensitivity, specificity and accuracy reach to 91.9% (5 440/5 919), 86.8% (1 515/1 745) and 90.7% (6 955/7 664), respectively. The area under the curve can reach 0.960 [95%CI (0.955-0.964)]. Compared with no MVI (MO) group, the value of GALAD model in MVI low-risk group (M1), MVI high-risk group (M2) and MVI (M1+2) were significantly higher (Z values were-12.517, -22.883, -21.655, P<0.05), Galad model predicts MVI (M2) in high risk group,AUC was 0.717 [95%CI (0.701-0.733)] (M0 ratio M2). Conclusion(s): GALAD model has better diagnostic performance in primary hepatocellular carcinoma and has certain predictive value for microvascular invasion.Copyright Â© 2019 by the Chinese Medical Association. |
| Wang, H. & Mu, X. T. Comparative study of dynamic MRI and MSCT in hepatocellular carcinoma with cirrhosis. Chinese Journal of Medical Imaging Technology 2007;23(7):1046-48. | Objective: To compare the sensitivity of dynamic MRI and multislice spiral CT(MSCT) for detection of hepatocellular carcinoma (HCC) by using explantation correlation in patients with cirrhosis. Method(s): Seventy-three cirrhosis patients without a known history of HCC who underwent MR and MSCT and subsequent liver transplantation within 90 days were examined. MSCT and MR with FLASH/T1WI, TSE/T2WI MR images were obtained. MR and MSCT images were obtained in the hepatic arterial, portal venous, and equilibrium phases. MRI interpretations were compared with MSCT. Result(s): Twelve of seventy-three patients had 20 HCC. MRI depicted 12 of 20 HCC, for a sensitivity of 60%. MRI depicted 6 of 7 (85.7%) lesions larger than 2 cm, 6 of 13 (46.2%) lesions equal or smaller than 2 cm. MSCT depicted 11 of 20 HCC, for a sensitivity of 55%. MSCT depicted 7 of 7 (100%) lesions larger than 2 cm, 4 of 13 (30.8%) lesions equal or smaller than 2 cm. The difference of sensitivity between MRI and MSCT was not statistically significant. Conclusion(s): The difference of sensitivity between MR and MSCT was not statistically significant, but they are all insensitive for small (<=2 cm) HCC. |
| Wang, H., Dong, J., Bao, J. F., Wang, C. B. [Analysis of the diagnostic efficiency of combining multiple laboratory hematological indicators in alpha-fetoprotein-negative hepatocellular carcinoma]. Zhonghua yi xue za zhi 2022;102(17):1303-10. | Objective: To establish a diagnostic model for alpha-fetoprotein-negative hepatocellular carcinoma (AFP-NHCC) by combining multiple laboratory hematological indicators and explore its clinical diagnostic efficiency. Methods: A total of 124 inpatients, including 110 males and 14 females, aged 57 (51, 66) years, who were first diagnosed with AFP-NHCC in the PLA General Hospital were included from December 2011 to June 2017. Meanwhile, 331 cases of non-HCC were enrolled as the control group, including 279 males and 52 females, aged 58 (51, 63) years old, with 47 cases of hepatitis B virus (HBV) infection, 40 cases of liver cirrhosis, 64 cases of hepatic hemangioma or cysts, 7 cases of liver nodules, 8 cases of fatty liver, 146 cases of non-liver disease and 19 health controls. Subjects in the AFP-NHCC group and the control group were divided into a training group and a validation group. A total of 196 subjects were involved in the training group, including 103 AFP-NHCC patients and 93 non-HCC patients (19 healthy controls, 25 patients with HBV infection, 22 patients with liver cirrhosis, 23 patients with hepatic hemangioma or cyst, and 4 patients with liver nodules). The differences in laboratory parameters were analyzed, and a diagnostic model of AFP-NHCC under different AFP levels was established. |

| Ref | Abstract |
|---|---|
| | Likewise, 259 subjects, including 113 patients with liver disease, were involved in the validation group to verify the diagnostic efficiency of the model for AFP-NHCC. The receiver operating characteristic (ROC) curve was used to analyze the sensitivity and specificity of different models, and the area under the curve (AUC) was calculated to evaluate the diagnostic performance of different models. Results: In the training group, the indicators of AFP-NHCC diagnostic model included platelet (PLT), prothrombin activity (PTA), serum albumin (ALB), prothrombin time (PT) and carbohydrate antigen 19-9 (CA19-9), and the AUC of the model was 0.848 (95%CI: 0.786-0.911) when AFP<=5 mug/L. Similarly, the indicators of AFP-NHCC diagnostic model included PLT, PTA, ALB, PT and hematocrit (HCT), and the AUC of the model was 0.839 (95%CI: 0.780-0.897) when AFP<=10 mug/L. When AFP<=20 mug/L, the indicators of AFP-NHCC diagnostic model contained PLT, PTA, ALB, PT, HCT and AFP, and the AUC of the model was 0.866 (95%CI: 0.815-0.917). The AUC values of these three models were higher than those of AFP and CA19-9 alone for the diagnosis of AFP-NHCC [0.634 (95%CI: 0.560-0.709), 0.691 (95%CI:0.620-0.761), all P<0.05]. The indicators screened by these three models were combined to establish the final diagnostic model, and the AUC of the model was 0.873 (95%CI: 0.824-0.923), with the sensitivity of 78.6% (81/103) and the specificity of 81.7% (76/93). In the validation group, the predictive AUC of the final model in liver disease patients was 0.892 (95%CI: 0.832-0.951), with the sensitivity of 100% (21/21) and the specificity of 71.7% (66/92), while in the total validation population, the predictive AUC was 0.931 (95%CI: 0.890-0.972), with the sensitivity of 100.0% (21/21) and the specificity of 75.6% (180/238). Conclusion: The final diagnostic model includes PLT, PTA, ALB, PT, HCT, CA19-9 and AFP, which has higher sensitivity and specificity, and has good diagnostic efficiency for the clinical diagnosis of AFP-NHCC. |
| Wang, L., Pan, L., Yao, M., Zheng, W., Fang, M., Qiu, L., Dong, Z. Clinical values of Wnt3a as a novel biomarker in diagnosis and prognosis of hepatocellular carcinoma. National Medical Journal of China 2016;96(44):3554-58. | Objective: To explore Wnt3a expression in HCC tissues and serum,and to discuss its clinical diagnostic and prognostic value. Method(s): The Wnt3a expressions were detected in a total of 186 patients (HCC, liver cirrhosis and chronic Hepatitis) and 40 controls by Elisa, comparing with AFP to evaluate its clinical diagnosis value. Wnt3a expressions in 80 HCC and surrounding tissues were analyzed by IHC,to explore its prognostic value. Result(s): Wnt3a with brown staining was mainly distributed in cytosol and of hepatocyte membrane. The higher expression (3-6 scores) was 71.3% in HCC, 13.8% in surrounding tissues, associated with poorly-differentiated grade, liver cirrhosis, HBV infection, higher TNM stage (P <0. 05) and 5-year survival rate (P <0. 001).identified as independent predictive factors for poor HCC outcome and closely related with lower five-year survival rate. Serum average Wnt3a levels were significantly higher (P <0. 001) in the HCC group than those in any other groups of benign liver diseases, with about 4. 0, 9. 2 and 26. 7 times higher than that in the liver cirrhosis, chronic hepatitis and normal control group. Wnt3a expression in HCC were closely related to AFP concentration, liver cirrhosis HBV infection, poor differentiation, TNM stagingand extra-hepatic metastasis (P < 0. 05). The sensitivity, specificity, accuracy, positive predictive value and negative predictive values were 92. 5, 94. 3, 93. 2, 96. 1 and 89. 3% at 800 ng/L as cutoff value for Wnt3a. Combining Wnt3a and AFP test, the total sensitivity could rise to 96. 3%. The area under ROC curve in Wnt3a (0.994) was higher than in AFP (0.710). Conclusions Wnt3a as a critical signal molecule in the Wnt pathway is a new specific marker for HCC diagnosis and prognosis. |
| Wang, N. Y., Zhao, W., Zhang, D., Zhang, Y. C. Clinical application of avidin-biotin ELISA to detect serum hepatoma-specific gamma-glutamyltransferase in patients with primary hepatic cancer. Zhonghua zhong liu za zhi [Chinese journal of oncology] 2009;31(2):114-17. | OBJECTIVE: To detect serum hepatoma-specific datura stramonium lectin-tightly binding gamma-glutamyl transferase (DSA-GGT) in patients with primary hepatic cancer (PHC) by avidin-biotin ELISA method which was established in our laboratory, and carry on a study of its clinical application. METHOD(S): To detect serum DSA-GGT in 45 healthy control subjects, 58 PHC patients and 203 non-PHC patients (including 36 patients with other tumors and 167 patients with benign liver diseases) with the method was established; meanwhile, AFP was detected by ELISA method. RESULT(S): 38 individuals were DSA-GGT positive in 58 PHC patients, the sensitivity was 65.5%. 18 individuals were DSA-GGT positive in 203 patients without PHC, the specificity was 91.1%. The sensitivity and specificity of AFP in diagnosis of PHC patients was 69.0% and 90.6%, respectively. The sensitivity and specificity of combination of DSA-GGT and AFP was 93.1% and 85.7%, respectively. The average intra-CV and inter-CV of DSA-GGT ELISA was 8.9% and 11.5%, respectively. CONCLUSION(S): The sensitivity and specificity of DSA-GGT ELISA method established in our lab is similar with that of AFP assay and the accuracy is good. Combination of DSA-GGT and AFP may improve the diagnostic sensitivity. The method should be potentially as a new way to improve diagnosis of PHC. |
| Wang, T., Wang, F. M., Gao, Y. T., Zhu, Z. Y., Guo, H. S., Yang, B., et al. Significance of GPC3 expression in liver biopsy specimens for differential diagnosis of liver diseases. World Chinese Journal of Digestology 2011;19(7):693-99. | AIM: To analyze the expression of glypican 3 (GPC3) in different liver diseases and to explore its significance in early diagnosis of hepatocel-lular carcinoma (HCC). METHOD(S): The expression of GPC3 and alpha-fetoprotein (AFP) was detected by immunohis-tochemistry in 126 liver needle biopsy specimens from 13 patients with very early stage HCC, 44 patients with early stage HCC, 16 patients with dysplastic nodules, 29 patients with liver cirrhosis, and 24 patients with hepatitis, and in 57 respected HCC specimens from patients with intermediate and advanced HCC. Twenty-eight respected normal liver tissue specimens were used as controls. RESULT(S): The expression of GPC3 in HCC differed significantly from those in other liver diseases (all P < 0.01). Detection of GPC3 expression showed a higher diagnostic sensitivity (80.7%) and specificity (99.4%) than that of AFP (37.7% and 93.6%, respectively). GPC3 was expressed in 92.3% of very early stage HCC and in 72.7% of early stage of HCC. In contrast, AFP only appeared in 38.5% of very early stage HCC and in 34.1% of early stage HCC. The positive rates of GPC3 expression in very early and early stage HCC were significantly higher than those in dysplastic nodules (both P < 0.01). GPC3 and AFP expression was not detected in liver cirrhosis, hepatitis or normal liver tissue. In patients with small HCC, the positive rate of GPC3 expression was 78.6%, and combined detection of GPC3 and AFP had a sensitivity of 85.7%. In patients with small HCC who had an AFP of <=20 |

| Ref | Abstract |
|---|---|
| | mug/L, the positive rate of GPC3 expression was 70%. The recurrence rate after radical surgery in GPC3-positive patients was higher than that of GPC3-negative ones (P < 0.05). CONCLUSION(S): GPC3 is highly expressed in HCC. Detection of GPC3 expression has a high sensitivity and specificity for diagnosis of very early and early stage HCC and therefore represents a potential diagnostic parameter for early HCC. Immunohistochemistry detection of GPC3 in liver needle biopsy specimens is an effective ancillary tool for early diagnosis of HCC. GPC3 expression is an independent prognostic factor for HCC recurrence after surgery. |
| Wang, W. & Wang, J. H. Detection of serum markers of primary hepatocellular carcinoma by ELISA using synthetic polypeptide antigen. Chinese Journal of Biologicals 2009;22(4):399-401. | Objective: To develop an ELISA method for detection of the serum markers of primary hepatocellular carcinoma (HCC). Method(s): Five kinds of up-regulated cell proteins, i.e. URG4, URG7, URG11, S15a and Suil, by HBx induction were served as serum markers of HCC in this paper. According to the sequences of above-mentioned proteins, polypeptides L4A/L4B, L7B, L11-1/L11-3/L11-4, L12A/L12B and Sui1A/Sui1B were synthesized and used as antigens for detection of the corresponding antibodies in sera by indirect ELISA. The developed ELISA method was evaluated for sensitivity, specificity and precision and used for detection of 161 samples from patients with HCC, liver cirthosis and hepatitis B and 39 from healthy blood donors. Result(s): The sensitivity, specificity, inter- and intra-variation coefficients of developed ELISA method were 93. 8%, 97. 9%, 3. 8% - 5. 6% and 7. 4% - 10. 3% respectively. Of the serum samples from patients with HCC, liver cirthosis and hepatitis B, 90. 4%, 92. 6% and 61. 7% were positive for not less than 1 marker, and 78. 6%, 70. 6% and 48. 3% for not less than 2 markers respectively. However, of the serum samples from healthy blood donors, 12. 2% were positive for not less than 1 marker and 6. 3% for not less than 2 markers. Conclusion(s): The developed ELISA may be used as an assistant diagnostic method of AFP and MRI for the screening of high risk population of HCC and the early diagnosis of small HCC. |
| Wang, W., Wang, J. H., Miao, X. H. Detection of serum markers of hepatocellular carcinoma by ELISA. Chinese Journal of Biologicals 2010;23(3):307-09. | Objective To further evaluate and optimize the developed ELISA for detection of serum markers of hepatocellular carcinoma (HCC). Methods The serum samples from 407 patients with liver diseases and 95 healthy blood donors were detected by ELISA developed by the authors, and'the results were analyzed. Results Of the 324 serum samples from patients with HCC, cirrhosis and hepatitis B, 77.6%, 76.1% and 52.5% were positive for at least 2 serum markers respectively. The positive rates of at least 3 serum markers in the patients with HCC were 39.9% and 310% higher than those with cirrhosis and hepatitis B respectively. Of healthy blood donors and the patients with drug-caused hepatitis, alcohol-caused hepatitis and other cancers, 87.5% - 100% were positive for not more than 1 serum marker. URG7 was the antibody positive marker which appeared at first, while URGI1 and S15a antibodies appeared in prominent proportions in the sera of patients with liver diseases at late stage of progress and those with HCC. Conclusion Five serum markers were positively related to the risk of HCC. The developed ELISA may be used for the screening of high-risk population of HCC and the early diagnosis of small HCC, which is an accessory diagnostic method of AFP and MRI. |
| Wang, Yuan-Yuan, Zhou, Chen-Jie, Li, Jing, Zhou, Ling, Li, Ming-Song. [Value of detection of serum glypican-3 level in diagnosis and therapeutic effect evaluation of primary hepatocellular carcinoma]. Nan fang yi ke da xue xue bao = Journal of Southern Medical University 2017;37(8):1060-65. | OBJECTIVE: To explore the clinical value of detecting serum glypican-3 in the diagnosis and therapeutic effect evaluation of primary hepatocellular carcinoma (PHC)., METHODS: Using sandwich ELISA, we detected serum glypican-3 levels in 60 patients with PHC, 60 with metastatic liver cancer, 50 with liver cirrhosis, 50 with chronic viral hepatitis, 20 with hepatic cyst, 20 with fatty liver, 20 with hepatic hemangioma and 20 with drug-induced hepatitis as well as in 40 healthy subjects (control). We also analyzed the changes in serum levels of glypican-3 and alpha fetoprotein (AFP) in PHC patients after treatment., RESULTS: PHC patients had significantly higher serum levels of glypican-3 than patients with other liver diseases and the control subjects (P<0.05). The levels of serum glypican-3 were significantly higher in patients with metastatic liver cancer, liver cirrhosis and viral hepatitis than in those with other benign liver diseases and the control subjects (P<0.05). Glypican-3 level was not associated with AFP level or liver function in PHC patients, in whom the positivity rates for glypican-3 and AFP were 65% and 56.7%, respectively. The detection rate of PHC increased to 85% by a combined detection of AFP and glypican-3. In the 23 PHC patients who responded positively to treatments, serum glypican-3 level showed a steady decline compared with that in 15 patients before treatment, while serum AFP level showed a similar decrease only in 10 patients., CONCLUSION: Combined detection of glypican-3 and AFP is expected to improve the early diagnosis rate of PHC. The different thresholds of serum glypican-3 may play a role in the differential diagnosis of PHC and other various liver diseases. Glypican-3 may serve as a better marker than AFP with a high specificity and sensitivity for evaluating the therapeutic effect in PHC patients. |
| Wen, B., Mi, J. J., Hou, T. H., Guo, X. H., Bai, C. A. Expression of serum bone morphogenetic protein 2 in patients with primary hepatic carcinoma. World Chinese Journal of Digestology 2010;18(4):397-99. | AIM: To investigate the diagnostic value of serum bone morphogenetic protein 2 (BMP2) in patients with primary hepatic carcinoma (PHC) by measuring serum BMP2 levels in patients with chronic liver disease. METHOD(S): Seventy serum samples, including 20 from patients with chronic hepatitis, 20 from those with hepatic cirrhosis and 30 from those with PHC, were detected in the study. Seventeen serum samples from normal donors were used as normal controls. The serum level of BMP2 was measured by enzyme-linked immunosorbent assay (ELISA). The content of serum alphafetoprotein (AFP) was determined by radioimmunoassay. RESULT(S): The level of serum BMP2 in PHC patients was significantly lower than those in chronic hepatitis patients, hepatic cirrhosis patients and normal controls (all P < 0.05). The sensitivity, specificity and accuracy of serum BMP2 measurement in diagnosis of PHC were 90%, 100% and 96.55%, respectively. The sensitivity of serum BMP2 measurement in diagnosis of PHC was higher than that of serum AFP measurement (X2 = 22.78, P = 0.001). CONCLUSION(S): Serum BMP2 level has appreciable value in the diagnosis of PHC. |

| Ref | Abstract |
|---|---|
| Wu, F. & Wang, C. X. Expression and clinical significance of LncRNA ZFAS1 in serum exosomes of patients with hepatocellular carcinoma. Chinese Journal of Cancer Prevention and Treatment 2019;26(12):849-54. | OBJECTIVE: Long-chain noncoding ribonucleic acid(LncRNA) zinc finger-protein antisense chain 1(ZFAS1) plays an important role in hepatocellular carcinoma(HCC). The purpose of this study was to investigate the expression of LncRNA ZFAS1 in serum exosomes of patients with HCC and to analyze its clinical significance. METHOD(S): A total of 84 patients with HCC in the Oncology Department of the Second People's Hospital of Dongying from 2012-04-01 to 2014-01-31 were selected as the study subjects(HCC group), a total of 50 patients with chronic hepatitis and cirrhosis were selected as benign liver disease group, and another 50 healthy subjects taken physical examination in the same period were selected as the control group. qRT-PCR was used to detect the expression level of LncRNA ZFAS1 in the serum exosomes of the subjects, and investigate its relationships with clinicopathological features of hepatocellular carcinoma and prognosis of patients. RESULT(S): The expression of LncRNA ZFAS1 in serum exosomes of HCC group, benign liver disease group and control group was (3. 62+/-0.84), (1. 56+/-0.41) and (1. 02+/-0.29) respectively, and there were significant differences between groups, F=325. 56, P<0.001. Multiple comparison RESULTS: showed that HCC group was higher than benign liver disease group(t=16. 212, P<0.001) and healthy control group(t=21. 123, P<0.001). Benign liver disease group was higher than healthy control group, t=7. 603, P<0.001. The expression of LncRNA ZFAS1 in serum exosomes was correlated with tumor stage(chi2=6. 980, P=0.008), Child-pugh grade(chi2=4. 061, P=0.044), AFP level(chi2=4. 842, P=0.028) and lymphatic metastasis(chi2=5. 010, P=0.025) in HCC patients. The progression-free survival(PFS) of low expression group of LncRNA ZFAS1 was 60.71%, which was higher than that of high expression group(37. 50%), chi2=4. 061, P=0.044. OS of low expression group of LncRNA ZFAS1 was 64. 29%, which was higher than that of high expression group(39. 29%), chi2=4. 677, P=0.031. Multivariate analysis showed that high expression of LncRNA ZFAS1(HR=2. 687, 95%CI:2. 224-3. 246, P=0.002) and tumor stage(HR=1. 578, 95%CI:1. 061-2. 346, P=0.046) were independent risk factors for prognosis of HCC patients. The area under curve(AUC) of serum exosome LncRNA ZFAS1 for diagnosis of hepatocellular carcinoma was 0.920(95%CI:0.876-0.965), Yoden index was 0.798, truncation value was 1. 783, sensitivity was 90.5%, specificity was 89. 0%. CONCLUSION(S): LncRNA ZFAS1 in serum exosomes of HCC patients is highly expressed, and related to the prognosis of HCC patients. Detection of the serum exosome LncRNA ZFAS1 expression level has certain reference value in the diagnosis of hepatocellular carcinoma, and it can be used as an indicator for diagnosis and prognosis of HCC patients.Copyright Â© 2019, Editorial Board of Chinese Journal of Cancer Prevention and Treatment. All right reserved. |
| Wu, L. Q., Wang, X. J., Zhang, B., Lu, Y. Expression of cancer-testis antigen SSX-2 and SSX-5 in tissues and peripheral blood of patients with hepatocellular carcinoma. World Chinese Journal of Digestology 2005;13(14):1667-72. | AIM: To investigate the expression of SSX-2 and SSX-5 mRNA in the tissues and peripheral blood of patients with hepatocellular carcinoma (HCC) and their relations with kinds of cilinical indexes, and to evaluate the possibility of using them as the targets for specific immunotherapy of HCC as well as the markers to in adjuvant diagnosis and prognosis and recurrence detection of HCC. METHOD(S): One-step reverse transcription polymerase chain reaction (RT-PCR) wasused to detect the expression of SSX-2 and SSX-5 mRNA in the cancer tissues, corresponding cancer-adjacent tissues and peripheral blood of 26 patiernts with HCC, as well as in12 cirrositic tissues and 10 normal tissuesOne sample, selected from each gene with positive PCR outcomes, was sequenced to testify the reliability. The relation between SSX-2, SSX-5 expression and kinds of clinical indexes, such as alpha-fetoprotein (AFP), HBsAg, diameter of the tumor, TNM staging, was analysed. RESULT(S): The positive rates of SSX-2 and SSX-5 mRNA expression were 34.6%(9/26) and 46.2%(12/26) in HCC tissues respectively, while none of the two genes was detected in the corresponding cancer-adjacent tissues. At least one gene was expressed in 65.4%(17/26) of HCC tissues and the co-expression was found in 15.4%(4/26). In the peripheral blood of the 26 HCC patients, the positive-expression rates of SSX-2 and SSX-5 mRNA were 19.2% (5/26) and 23.1%(6/26) respectively. The positive rates of single expression and co-expression were 34.6%(9/26) and 7.7%(2/26) respectively. In the 12 patients with hepatic cirrhosis and 10 nomal controls, none of SSX-2 and SSX-5 mRNA was detectable in liver tissues and peripheral blood. After sequencing, the RT-PCR products were confirmed to be the target cDNA. No relationship was found between the expression of the two genes and the clinical indicators such as age, sex, tumor size, serum AFP level and infection of hepatitis B virus (P>0.05). However, in some patients with normal serum AFP (<20 ng/L), specific expression of SSX-2 and/or SSX-5 genes were/was observed in the peripheral blood. CONCLUSION(S): SSX-2 and SSX-5 mRNA are expressed with a high frequency and specificity in HCC, and they can be also co-expressed. They can be used as the targets for specific immunotherapy of HCC. No relationship exists between the expression of SSX-2 and SSX-5 and clinical indicators, and the expression of the two genes may be used as the markers in diagnosis and prognosis prediction of HCC. |
| Xu, A. F., Wang, M. C., Sui, D. M., Yuan, Y. H., Chen, G. Subject diagnostic value of detecting a1pha-fetoprotein variants with a new microspincolumn method in hepatocellular carcinoma. Zhonghua shi yan he lin chuang bing du xue za zhi = Zhonghua shiyan he | OBJECTIVE: To evaluate the usefulness of new microspincolumn method for the measurement of a1pha-fetoprotein variant AFP-L3 in differentiation of benign and malignant liver disease and the warming for liver cancer. METHOD(S): AFP-L3 was isolated by using microspincolumn coupled with lens culinaris agglutinin (LCA), AFP and AFP-L3 were determined with chemiluminescent immunoassay, the proportion of AFP-L3 levels AFP-L3(%) were calculated, and the relationship between the elevated AFP-L3(%) levels and benign and malignant liver disease was analyzed. RESULT(S): The levels of AFP-L3(%) in serum of patients with hepatocellular carcinoma was significantly higher than those in the patients with other liver diseases (P < 0.001). Taking AFP-L3(%) >or= 10% as the diagnostic criteria, the sensitivity for diagnosis of liver cancer was 90.9%. CONCLUSION(S): Detection of AFP-L3 seemed to be of clinical value in diagnosis and differential diagnosis of hepatocellular carcinoma; it may be especially important for identifying patients with hepatocellular carcinoma whose a1pha-fetoprotein level is low. |

| Ref | Abstract |
|---|---|
| linchuang bingduxue zazhi = Chinese journal of experimental and clinical virology 2007;21(1):67-69. | |
| Xu, F., Li, X. L., Wang, Y. M. Study on diagnostic significance of GPC3 in the patients with primary hepatocellular carcinoma. Journal of Dalian Medical University 2013;35(4):381-83. | Objective: To evaluate the diagnostic value of GPC3 for patients with primary hepatocellular carcinoma, especially when AFP was negative or swelling was smaller. Method(s): Serum GPC3 and AFP were measured by ELISA and the electrochemical luminescence method in 120 patients with primary hepatocellular carcinoma, 32 patients with hepatic cirrhosis. The results were divided into groups according to the size of swelling(<3 cm,3 ~ 5 cm, 5 ~ 10 cm > 10 cm). Result(s): (1) The value of GPC3 in hepatocellular carcinoma group was (22.014 +/- 36.930) ng/mL, and the value of GPC3 in hepatic cirrhosis was (8.590 +/- 8.171) ng/mL. The difference was significant between two groups(P < 0.01). (2) The positive rates of GPC3 and AFP were 86.67% and 71.43% in swelling size <3 cm group, and the difference was not significant(P > 0.05). The positive rates of GPC3 in the other groups(swelling size 3 ~ 5 cm, 5 ~ 10 cm, > 10 cm) were lower than AFP, and the difference was not significant(P > 0.05). (3) The positive rate of GPC3 in case of negative AFP was 75%. Conclusion(s): As a new tumor marker in primary hepatocellular carcinoma, GPC3 perhaps has higher diagnosis value in case of negative AFP and smaller swelling size. GPC3 in combination with AFP can improve the early stage detection of HCC. |
| Xu, P. J., Yan, F. H., Wang, J. H., Lin, J., Ji, Y., Chen, C. Z., Shen, J. Z. The value of breath-hold diffusion-weighted imaging in small hepatocellular carcinoma lesion (<=3 cm) detection. National Medical Journal of China 2009;89(9):592-96. | Objective: To evaluate the added value of single breath-hold diffusion-weighted imaging (DWI) in detection of small HCC lesions (<=3 cm) in patients with chronic liver disease, by comparing the detection sensitivity of DWI/conventional dynamic contrast enhancement (DCE) MRI and that of conventional DCE MRI alone. Method(s): A total of 54 patients with chronic liver diseases underwent abdominal MRI at 1.5T, including T1-weighted (T 1WI), T2-weighted (T2 WI), and 2D conventional DCE. For each patienl study, axial DWI was performed with a single-shot echo-planar imaging (EPI) sequence using modified sensitivity encoding (mSENSE) with b-value of 500 seconds/mm2. A total of 20-24 slices were obtained during a 15-17-second breath-hold. Two observers independently interpreted the combined DWI/conventional DCE MRI images and the conventional DCE MRI images in random order. For all small HCC lesions and micro-hepatocellular carcinoma (smaller than 10 mm MHCC), the diagnostic performance using each imaging techniques were evaluated by ROC analysis. Sensitivity and positive predictive values were also calculated. Result(s): The mean areas under the ROC curve (Az) of combined DWI/conventional DCE MKI images (0.945, 0.86) were, statistically higher than those of conventional DCE MRI alone (0.86, 0.64) for all small HCC lesions and MHCC (micro hepatocellular carcinoma) lesions (P < 0.01). The lesion detection sensitivities using the combined technique of both observers were significantly higher than those using the conventional DCE MRI alone for all small lesions and for MHCC (P < 0.01). For MHCC, the sensitivities using the combined technique and the conventional DCE MRI alone were 95.8%-96.0% and 62.5%-68.0%, respectively. The positive predictive values for MHCC using the combined imaging technique (95.8%-96.0%) were higher than those using the conventional DCE MRI alone (94.1%-94.4%)(P < 0.05). For all small HCC lesions, the positive predictive values using the combined imaging technique and the conventional DCE MRI alone were 98.6%-98.7% and 95.5%-95.7%, respectively and there was no statistical difference. Conclusion(s): Combined use of Breath-hold DWI with conventional DCE MRI helped to provide higher sensitivities than conventional DCE MKI alone in the detection of small HCC lesions in patients with chronic liver disease. Furthermore, DWI could provide additional valuable information that benefits the differential diagnosis. |
| Xu, Wen-fang, Fei, Ying-ming, Zhou, Jian-kang, Shen, Hua-jing, Chen, Xue-fang, Lv, Qiu-qiong. [Significance of serum golgi protein 73 (GP73), alpha-fetoprotein (AFP) and lectin-reactive alpha-fetoprotein (AFP-L3) expresssion in primary hepatic carcinoma]. Zhonghua shi yan he lin chuang bing du xue za zhi = Zhonghua shiyan he linchuang bingduxue zazhi = Chinese journal of experimental and clinical virology 2011;25(4):286-88. | OBJECTIVE: To explore the alone and joint diagnostic value of serum golgi protein 73 (GP73), alpha-fetoprotein (AFP) and the percentage of lectin-reactive aipha-fetoprotein (AFP-L3) of primary hepatic carcinoma (PHC), and provide a novel method for diagnosis for PHC and screening for high-risk population., METHODS: ELISA was used to detect the serum level of GP73, AFP and AFP-L3% in 81 cases of PHC,176 cases chronic hepatitis and liver cirrhosis, 30 cases other tumber cancer and 40 cases of health people., RESULTS: The sensitivity of GP73, AFP and AFP-L3% in PHC is 77.78%, 62.69% and 51.85%, and the specificity is 84.55%, 86.99% and 96.34%, respectively. Joint detection could increase the sensitivity up to 88.89%., CONCLUSION: GP73 was a high sensitivity mark for dignosis of PHC, while AFP-L3% was a high specificity mark for dignosis of PHC. The joint detection could improve PHC diagnostic performance. |
| Xu, X., Xiao, X., Huang, C., Gao, Z., Ji, J., Fang, M. Establishment of lectin-ELISA for sialylated fetuin-A and its | Objective To establish a lectin enzyme-linked immunosorbent assay (lectin-ELISA) for the dection of sialylated fetuin-A and to explore the clinical diagnostic value of sialylated fetuin-A in hepatocellular carcinoma (HCC). Methods From January 2017 to December 2020, 300 HCC patients and 160 disease controls, including 36 liver cirrhosis subgroups and 124 chronic hepatitis B subgroups, were collected from Shanghai Eastern Hepatobiliary Surgery Hospital. At the same time, 100 healthy subjects were collected as healthy controls. Lectin-ELISA method for detecting |

| Ref | Abstract |
|---|---|
| diagnostic value in primary hepatocellular carcinoma. Chinese Journal of Laboratory Medicine 2022;45(4):366-72. | sialylated fetuin A was established based on the principle that Sambucus nigra lectin (SNA) can recognize the structure of alpha-2, 6-linked sialic acid residues. Differences between groups were compared using t-test or analysis of variance. Logistic regression method was used to establish the multi-index joint detection model, and receiver operating characteristic curve (ROC) was used to evaluate the efficacy of single index and joint detection model in the diagnosis of HCC. Results A lectin-ELISA method for the detection of serum Sia-fetuin A was established. The linear regression coefficient of the system was 0.978 5, and the precision evaluation and interference experiments were in line with the clinical detection requirements. Using this method to detect serum Sia-fetuin A levels in each group, the levels of HCC group, disease control group and healthy control group were 1.362+/-0.310, 1.199+/-0.370, 1.086+/-0.420, respectively, and the three groups decreased in turn. The areas under the curve of Sia-fetuin A, alpha-fetoprotein, and their combined detection models for differential diagnosis of HCC were 0.790, 0.809, and 0.860, respectively. The diagnostic model had a sensitivity of 79.3% (238/300) and a specificity of 95.0% (247/260). Among the 300 patients in the HCC group, 138 (46%) patients were negative for serum AFP (<20 mug/L), and their serum Sia-fetuin A level was 1.364+/-0.305. Combining the disease control group and the healthy control group into the non-Cancer group, the serum Sia-fetuin A level was 1.146+/-0.381. The serum level of Sia-fetuin A in AFP-negative HCC patients was higher than that in non-HCC group (t=6.134, P<0.001). The areas under the curve of Sia-fetuin A and the combined diagnostic model for the diagnosis of AFP-negative HCC were 0.776 and 0.919, respectively. The combined diagnostic model had a sensitivity of 93.4% (129/138) and a specificity of 77.3% (201/260). Conclusion Serum Sia-fetuin A and combined determination model can provide a new auxiliary diagnostic index for AFP-negative HCC.Copyright Â© 2022 Chinese Medical Journals Publishing House Co.Ltd. All rights reserved. |
| Yan, C. Y., Fu, Z. J., Liu, G. S., Li, J. Value of heat shock protein 90 combined with alpha-fetoprotein in early diagnosis of hepatocellular carcinoma. World Chinese Journal of Digestology 2021;29(21):1215-21. | BACKGROUND Hepatocellular carcinoma (HCC) is the sixth most common cancer in the world, and early diagnosis mainly relies on serological examination. With the deepening of molecular research of heat shock protein 90 (HSP90) in hepatocellular carcinoma, this study innovatively explored the value of HSP90 combined with alpha-fetoprotein (AFP) in early diagnosis of HCC. AIM To evaluate the diagnostic value of HSP90 combined with AFP in HCC. METHODS A total of 383 subjects, including patients with primary liver cancer, those with posthepatitic cirrhosis (types B and C), and healthy subjects, were selected from August 2017 to July 2020. The serum concentration of AFP was measured by electrochemical method, the serum concentration of HSP90 was measured by ELLISA, and the results were processed and analyzed using statistical software. RESULTS When AFP or HSP90 alone was used for the diagnosis of HCC, the sensitivity of HSP90 was higher than that of AFP, while the specificity of AFP was higher than that of HSP90. The sensitivity and specificity of AFP combined with HSP90 in the diagnosis of HCC were 87.8% and 91.2%, respectively. CONCLUSION AFP combined with HSP90 can significantly improve the diagnostic value of either of them alone in HCC.Copyright Â© The Author(s) 2021. Published by Baishideng Publishing Group Inc. All rights reserved. |
| Yangmei, S. Z., Chen, G., Dang, Y. W., Chen, S. Clinical significance of elevated serum DcR3 in patients with hepatocellular carcinoma. World Chinese Journal of Digestology 2009;17(20):2042-47. | AIM: To investigate the diagnostic value and clinical significance of elevated serum decoy receptor 3 (DcR3) in patients with hepatocellular carcinoma (HCC). METHOD(S): Serum DcR3 levels were measured using enzyme-linked immunosorbent assay (ELISA) in 67 patients with HCC, 8 patients with liver cirrhosis, 17 patients with cholecystitis and 28 healthy individuals. Serum alpha fetoprotein (AFP) levels were measured by chemiluminescence. The expression of DcR3 in HCC tissue was evaluated by immunohistochemistry (IHC). RESULT(S): The levels of serum DcR3 in both HCC and liver cirrhosis patients were significantly higher than that in healthy individuals (P < 0.01). The level of serum DcR3 in HCC patients was correlated with the development of cirrhosis, capsular infiltration and metastasis (P < 0.05). There was a positive correlation between the levels of DcR3 in the serum and HCC tissue (r = 0.395, P < 0.01). The positive rate of DcR3 in the serum was significantly higher than that in HCC tissue (P < 0.05). The sensitivities of single detection of AFP or DcR3 were 82% and 3 76%, respectively. In contrast, the sensitivity of combined detection of AFP and DcR3 rose to 93%. CONCLUSION(S): Elevated serum DcR3 may play an important role in the pathogenesis, development, invasion and metastasis of HCC. It may be of great significance for the screening, diagnosis and prognosis of HCC to simultaneously monitor the levels of DcR3 and AFP in the serum of high-risk populations and HCC patients. |
| Ye, F., Song, Y., Yu, X. D., Zhang, H. M., Ouyang, H. Hepatobiliary phase imaging with Gd-EOB-DTPA for detection and characterization of hepatocellular carcinoma in patients with chronic liver disease. Chinese Journal of Medical Imaging Technology 2015;31(4):571-75. | Objective: To evaluate hepatobiliary phase (HBP) imaging with Gd-EOB-DTPA for detection and characterization of hepatocellular carcinoma (HCC) in patients with chronic liver disease. Method(s): Totally 99 patients with chronic liver disease who underwent Gd-EOB-DTPA MR examinations were analyzed retrospectively, 89 patients were clinically or pathologically diagnosed as HCC, and 10 patients were clinically diagnosed as normal to serve as a control. The ability of HBP and DWI in detection of HCC were compared by two senior radiologists. Another 3 junior and medium grade radiologists independently reviewed each examination with and without HBP. Lesion detection, confidence scores and area under the ROC curve (AUC) were compared. Statistical comparisons of the rated results were made by using Wilcoxon test or McNemar's Chi-square test. Result(s): A total of 130 nodules were diagnosed as HCC (n=111) and benign (n=19), 100 HCCs were proved by pathology and 11 HCCs were clinically diagnosed. HBP had a higher detection rate for HCCs than DWI (99.10% [110/111] vs 90.99% [101/111], P=0.012). With the inclusion of the HBP, lesions detection was improved (93.00% [17.67/19] vs 70.16% [1.33/19], P=0.016) for HCCs<=1 cm in size and mean confidence scores significantly increased (P=0.001). For lesions<=1 cm in size, the diagnostic performance improved with the addition of the HBP (AUC 0.949 [5%CI 0.815~0.995] vs 0.744 [5%CI 0.566~0.877], P=0.0023). Conclusion(s): HBP with Gd-EOB-DTPA has a superior sensitivity than DWI. It may improve the detection rate and the |

| Ref | Abstract |
|---|---|
| | diagnosis confidence of HCCs<=1 cm in patients with chronic liver disease.Copyright Â© 2015 by the Press of Chinese Journal of Medical Imaging and Technology. |
| Zhan, X., Fang, M., Xiao, X., Ji, J. The value of core fucosylated alpha 2 macroglobulin in the diagnosis of hepatocellular carcinoma. Chinese Journal of Laboratory Medicine 2019;42(3):193-97. | Objective: To investigate the core fucosylated alpha 2-macroglobulin(LCA-alpha2M) level in patients with hepatocellular carcinoma (HCC), liver cirrhosis (LC) and chronic hepatitis B (CHB), and explore its diagnostic value in HCC. Method(s): A total of 193 HCC patients,104 LC patients and 71 HC patients in Shanghai Eastern Hepatobiliary Surgery Hospital and 45 CHB patients in Changzheng Hospital from January 2013 to December 2016 were included for retrospective study. The method for detecting LCA-alpha2M was set up, and then the levels of serum alpha2M and LCA-alpha2M in each group were detected. The diagnostic value of LCA-alpha2M for HCC was evaluated by receiver operating characteristic (ROC) analysis. Result(s): The level of LCA-alpha2M/alpha2M x100(LCA-alpha2M%) was significantly higher in HCC patients[31.25(26.61-35.42)] than that in LC patients [26.00(22.30-30.64)], CHB patients[26.23(23.86-31.86)] and healthy controls[20.29(17.35-22.60)] (H values were 5.626, 3.388 and 10.942, respectively, P<0.05). The area under the receiver operating characteristic curve (AUC) of LCA-alpha2M% for identifying HCC was 0.768 (0.725-0.808). Combined alpha-fetoprotein(AFP) and LCA-alpha2M%, the area under the ROC curve was 0.890(0.856-0.919). For AFP negative HCC patients, the sensitivity of LCA-alpha2M% was 77.42%(24/31). Conclusion(s): LCA-alpha2M% has some values in assistant diagnosis of HCC, and could improve the detection of AFP negative HCC patients.Copyright Â© 2019 by the Chinese Medical Association. |
| Zhang, H., Chen, X., Yang, B., Zhang, M. Research on the serum level of microRNA-224 in hepatocellular carcinoma patients and its clinical diagnostic significance. Chinese Journal of Clinical Oncology 2014;41(9):576-79. | Objective: To investigate the serum level of miR-224 in hepatocellular carcinoma (HCC) patients and its clinical diagnostic significance. Method(s): The serum level of miR-224 was detected by real-time quantitative PCR. This study included 42 cases of patients with HCC, 36 patients with liver cirrhosis (LC), 55 patients with chronic hepatitis B (CHB), and 40 healthy persons (NC). The relative expressions of miR-224 were calculated. The receiver operating characteristic (ROC) curves were analyzed to determine the sensitivity and specificity of miR-224 expression levels in HCC diagnosis. Result(s): Result shows that the relative miR-224 expression was higher in the serum of HCC patients than that in the CHB, LC, and NC groups. The difference was statistically significant (P<0.05 or P<0.01). The relative miR-224 expression in the serum of HCC patients and alpha fetoprotein (AFP) were positively correlated (P<0.05). By comparison, the tumor size, TNM stage, tumor differentiation, and lymph node metastasis were not correlated with mirR-224 expression (P>0.05). ROC analysis shows that the best critical value of the relative expression levels of miR-224 was 3.47, with sensitivity of 82.2%, specificity of 92.8%, and area under the curve of 0.935. Conclusion(s): The serum level of miR-224 in HCC patients has high specificity, and miR-224 has great potential to become a new serological marker for the diagnosis of HCC. |
| Zhang, Hong, Huang, Dong-feng, Wei, Qun, Hua, Ting-yan, Li, Feng. [Expression and its clinicopathological features of tumor rejective antigen 1 in human tissues of hepatocellular carcinoma and liver cirrhosis]. Zhonghua gan zang bing za zhi = Zhonghua ganzangbing zazhi = Chinese journal of hepatology 2010;18(9):661-65. | OBJECTIVE: To investigate the expression of tumor rejective antigen 1 in hepatocellular carcinoma (HCC) and liver cirrhosis(LC) tissues, and the relationship between clinicopathological feature and HCC., METHODS: The expressions of TRA1 mRNA and its protein were detected by reverse transcription polymerase chain reaction (RT-PCR) and Western blot respectively. Immunohistochemical staining was used to further examine the expression of TRA1 protein in LC, HCC and control tissues. The relationship between clinicopathological feature and HCC was analyzed. Data of RT-PCR and Western blot were analyzed by One-way ANOVA; results of immunohistochemical staining were analyzed by Fisher's exact test and correlation analysis using Spearman rank correlation., RESULTS: RT-PCR data showed that the expression of TRA1 mRNA was higher in HCC and LC tissues than that in the normal liver tissues (F values were 20.821 and 12.311 respectively, P is less than 0.05). The expression of TRA1 protein in HCC and LC tissues was signiflcantly higher than that in control by Western blot (F values were 21.231 and 20.125 respectively, P < 0.05). The immunohistochemical data showed the expression of TRA1 protein was gradually increased in HCC group than that in the LC group and control group, and the positive expression rate of TRA1 was 57.14%, 78.95% and 93.75% respectively. The expression of TRA1 protein was negatively correlated with HCC differentiation (r = -0.4655, P = 0.0073) and positively correlated with HCC TNM staging (r = 0.5157, P = 0.0025)., CONCLUSION: The over-expression of TRA1 in hepatocirrhosis and HCC is correlated with the formation and development of HCC. It may be a prognostic marker for the diagnosis of HCC and be associated with the degree of differentiation and HBV infection. It can be used as a marker for prognostic prediction of HCC. |
| Zhang, J. W., Guan, L. Y., E, C. Y., Yang, J. H., Xuan, W., Meng, Z. H. [The value of serum abnormal prothrombin in clinical application of hepatocellular carcinoma]. Zhonghua wai ke za zhi [Chinese journal of surgery] 2020;58(10):776-81. | Objective: To examine the value of serum protein induced by vitamin K absence or antagonist-II (PIVKA-II) detection in the early diagnosis and surveillance of hepatocellular carcinoma (HCC). Methods: The clinical data of 215 patients with HCC admitted to Department of Hepatobiliary-Pancreatic Surgery of China-Japan Union Hospital of Jilin University from October 2017 to May 2018 were analyzed retrospectively. There were 172 males and 43 females, aged of (59.0+/-9.3) years old (range 34 to 86 years old). In addition, there were 85 non HCC patients were enrolled in the control group, 42 males and 43 females, aged (54.2+/-11.3) years old (range 22 to 80 years old). The blood sample of 3 ml was drawn from the elbow vein at 6:00 am on the next day of admission, and then was kept in low temperature away from light, and sent for PIVKA-II detection on the same day. The positive value of AFP was >=20 mug/L and PIVKA-II was >=32 AU/L. The data were analyzed statistically by chi(2) test, t test or rank sum test. The correlation between AFP, PIVKA-II and tumor maximum diameter was analyzed by linear regression. Results: The sensitivity of PIVKA-II detection only for the diagnosis of HCC in all stages was significantly higher than AFP or equivalent to AFP, the overall sensitivity of PIVKA-II and AFP was 85.1% and 52.1%, respectively. But the specificity of PIVKA-II was lower than that of AFP, they were 78.8% and 96.5%, respectively. In particularly, in the earlier stage of HCC (Ia) , the sensitivity of PIVAK-II to HCC was 64.5%, while the AFP was only 26.3%. Combined detection of PIVKA-II and AFP |

| Ref | Abstract |
|---|---|
| | significantly improved the diagnostic rate of HCC to 88.4%, and the specificity to 76.5%. Moreover, there was a positive correlation between PIVKA-II level and the maximum tumor diameter (r(2)=0.587, P<0.05), but there was no correlation between the AFP level and the maximum tumor diameter (r(2)=0.296, P>0.05). The positive rate of PIVKA-II in the diagnosis of HCC with vascular invasion was also significantly higher than that of AFP (P<0.01) . Conclusions: PIVKA-II can be used as a serological marker for HCC screening and diagnosis. In particular, PIVKA-II detection was significantly sensitive than AFP in the earlier stage of HCC. Combined detection of PIVKA-II and AFP can effectively improve the diagnostic rate of HCC in all stages. The significant elevation of PIVKA-II is also helpful to determine the tumor aggressiveness, vascular invasion and prognosis of HCC patients. |
| Zhang, S. Y., Cao, N., Chen, M. K., Zhang, L. D., Bai, Y. Q. Diagnostic value of plasma HLA-F adjacent transcript 10 mRNA expression in patients with hepatocellular carcinoma or colon cancer. World Chinese Journal of Digestology 2015;23(36):5775-83. | AIM: To detect plasma levels of human leukocyte antigen (HLA)-F adjacent transcript 10 (FAT10) mRNA in patients with hepatocellular carcinoma (HCC) or colon cancer (CC), and to assess its diagnostic value. METHOD(S): Blood samples were collected from 70 patients with HCC, 60 patients with cirrhosis, 64 patients with CC, and 64 healthy volunteers. The expression levels of plasma FAT10 mRNA and -fetoprotein (AFP) protein were measured by RT-PCR and ELISA, respectively. RESULT(S): The positive rates of FAT10 mRNA in plasma of patients with HCC, those with cirrhosis and healthy controls were 72.85%, 28.3% and 12.5%, respectively, and there were iiP < 0.05). High expression levels of FAT10 mRNA in HCC were related to TNM stage and lymph node metastasis (P < 0.05). The positive rate of 2TPii CC patients than in healthy controls (P < 0.05). The up-regulation of FAT10 mRNA in CC was closely related to Duke's stage and distance metastasis. FAT10 mRNA had a sensitivity of 66.6% for diagnosis of AFP-negative HCC. Furthermore, the FAT10 mRNA sensitivity of 68.8% and specificity of 79.8% in distinguishing small HCC patients from non-HCC individuals (cirrhosis patients and healthy controls). Combined AFP and FAT10 mRNA detection could improve the sensitivity to 84.4%. CONCLUSION(S): FAT10 mRNA is a promising serological marker for HCC and CC. Plasma FAT10 mRNA and AFP protein could be combined to improve the diagnosis of HCC.Copyright Â© 2015 Baishideng Publishing Group Inc. All rights reserved. |
| Zhang, Y., Yang, B., Wang, Y., Gao, Y., Bai, T., Bai, Y. Methylation profile of tumor suppressor genes in the cell-free DNA of plasma in hepatocellular carcinoma. Chinese Journal of Clinical Oncology 2013;40(23):1436-40. | Objective: This study aimed to detect the special methylation profile in peripheral blood for hepatocellular carcinoma (HCC). Method(s): The methylation status of 12 tumor suppressor genes (TSGs) in the plasma of 55 HCCs and 54 chronic liver diseases (CLDs) was tested by methylation-specific PCR (MSP). Result(s): In HCC, the methylation frequencies were 78.18% in APC, 63.64% in cyclin D2, 58.18% in TFPI2, 49.09% in DKK3, 49.09% in GSTP1, 47.27% in p16, 40.00% in Sigma 14-3-3, 18.18% in SFRP2, 16.36% in ppENK, 9.09% in DKK2, 7.27% in NPTX2, and 5.45% in LHX1. In CLD, the methylation frequencies were 27.78% in APC, 22.22% in cyclin D2, 7.41% in TFPI2, 3.70% in DKK3, 16.67% in GSTP1, 37.04% in p16, 37.04% in Sigma 14-3-3, 11.11% in SFRP2, 20.37% in ppENK, 7.41% in DKK2, 7.41% in NPTX2, and 9.26% in LHX1. The methylation frequencies of APC, cyclin D2, TFPI2, DKK3, and GSTP1 were higher in HCC than in CLD (P<0.01). The methylation index (MI) of the five-gene methylation profile was statistically higher in HCC (median, 0.6; IQR, 0.4-0.8) than CLD (median, 0.2; IQR, 0-0.2) (P<0.01). In HCC, MI was statistically related to the patient's age. Older patients with HCC had a higher MI. No significant correlation was observed between MI and other clinicopathological data. Moreover, MI was not related to the disease free survival and the overall survival in HCC. Conclusion(s): This five-gene methylation profile may be a promising biomarker for the assistant diagnosis of HCC. |
| Zhao, H. J., Li, S. M., Zhang, H. J., Guo, Z. J., Fan, H. Y., Yang, G. R., Wang, L. Diagnostic and prognostic value of RASSF1A and APC gene methylation in plasma of liver cancer patients. Chinese Journal of Cancer Prevention and Treatment 2013;20(1):53-57. | OBJECTIVE: To explore the correlation of the suppressor gene RASSF1A and APC promoter region methylation with the diagnosis and prognosis in plasma of liver cancer patients. MRTHODS: 102 cases liver cancer plasma, 80 cases cirrhosis plasma and 100 cases healthy people plasma were recruited and the DNA were extracted, methylation specific PCR was used for detection of RASSF1A and APC gene methylation, all patients were followed up, and analyzed the correlation of methylation status with the diagnosis and prognosis. RESULT(S): Positive ratio of RASSF1A gene methylation in plasma of liver cancer and cirrhosis was 51.96% (53/102) versus 15.00% (12/80), the difference was statistically significant, chi2=26.677, P=0.000. Positive ratio of APC gene methylation in plasma of liver cancer and cirrhosis was 47.06% (48/102) versus 22.50% (18/80), the difference was statistically significant (chi2=11.700, P=0.001). There were 44(43.14%) AFP positive patients in the 102 patients with liver cancer. In 58 cases of AFP negative patients, the positive ratio of promoter region in RASSF1A and APC gene methylation was 53.44% (31/58) versus 46.55% (27/58). The survival rate of RASSF1A gene's methylation status was significantly different between the patients with positive and negative (P=0.011). But there was no significant difference in survival rate of APC gene's methylation status between patients with positive and negative (P=0.175). Multivariate analysis showed that AFP, clinical stage, treatment, with or without portal vein tumor embolus were independent prognosis factors, both of RASSF1A and APC gene are methylation status were independent prognosis factors (P=0.019). CONCLUSION(S): RASSF1A and APC gene promoter region methylation are helpful for diagnosis of liver cancer, especially in AFP negative liver cancer patients. RASSF1A and APC gene promoter region methylation can be used as an independent prognostic factor for the evaluation of survival prognosis of liver cancer. |
| Zhao, Q., Liu, Y. Y., Lei, X., Lei, F. F., Li, G., Li, F., et al. PIVKA-II as a screening marker for hepatocellular carcinoma in patients with hepatitis B virus | Hepatitis B virus (HBV) infection is highly prevalent in China. Hepatocellular carcinoma (HCC) is the sixth most common cancer in the world, and HBV infection is the most common predisposing factor for HCC. China has 53.3% of HCC cases worldwide and 80% of HCC cases are related to HBV-associated hepatitis. HCC grows silently with mild or no symptoms until advanced. Due to the absence of effective treatment for advanced stage hepatic cancer, screening for high-risk population for early detection is of particular importance. Alpha fetoprotein (AFP) is the most widely used biomarker for HCC surveillance, but has a sensitivity of only 60%, and it is easy to be |

| Ref | Abstract |
|---|---|
| infection. World Chinese Journal of Digestology 2017;25(9):803-09. | effected by other factors. Studies have suggested that the level of prothrombin induced by vitamin K absence- II (PIVKA-II) in serum (40 mAU/mL) could be used as a predictive biomarker of HCC. PIVKA-II proved to be superior to AFP for early detection of HCC with regard to higher sensitivity and specificity. Furthermore, PIVKA-II can be used to di s c r iminat e between different histopathological grades of HCC. The combined detection of PIVKA- II and AFP in HCC is important for early diagnosis, therapeutic efficiency and prognosis evaluation, especially in AFP-negative HCC.Copyright Â© The Author(s) 2017. |
| Zhao, Xiu-Ying, Li, Ning, Ding, Hui-Guo. [Detection and evaluation of serum GP73, a resident Golgi glycoprotein, as a marker in diagnosis of hepatocellular carcinoma]. Zhonghua zhong liu za zhi [Chinese journal of oncology] 2010;32(12):943-45. | OBJECTIVE: The aim of this study was to determine if Golgi protein-73 (GP73) is up-regulated in hepatocellular carcinoma (HCC), and to explore the possibility of using GP73 in diagnosis and treatment of HCC., METHODS: Serum GP73 was detected by a quantitative ELISA assay. A total of 372 serum samples were included, among them 43 from healthy donors (Normal), 110 from either chronic hepatitis or cirrhosis (CH/LC), and 219 from HCC patients. The levels of GP73 were compared among the 3 groups. The received operating curve (ROC), sensitivity and specificity of GP73 for HCC patients were calculated., RESULTS: The average level of GP73 expression in normal, CH/LC and HCC groups were (22.1 +/- 8.5) ng/ml, (81.4 +/- 57.2) ng/ml and (271.5 +/- 202.3) ng/ml, respectively. Serum GP73 levels were significantly higher in patients with HCC compared to those with CH/LC (P < 0.001). The GP73 area under ROC was 0.857. Put 100 ng/ml as the optimal cut-off point, GP73 had a sensitivity of 76.7% and a specifically of 73.2%. GP73 level had a significantly higher sensitivity than AFP (32.0%) in diagnosis of early HCC (P < 0.001). Moreover, GP73 level was elevated in the serum (72.5%, 108/149) of individuals with HCC who had serum AFP level less than 400 ng/ml. Following-up study of 4 HCC patients with low level AFP indicated that GP73 was associated with treatment and prognosis of HCC., CONCLUSION: Higher level of GP73 can be found in the serum of patients with HCC than those without. GP73 is better than AFP for the diagnosis of early HCC and in evaluating treatment result in patients with normal AFP. Further studies may help to validate both the role and mechanism of GP73 in diagnosis of HCC. |
| Zhao, Y., Li, Y., Hu, B., He, X., Huang, J., Zhan, M. Clinical application of serum Golgi protein 73 in the diagnosis and progress of hepatocellular carcinoma. National Medical Journal of China 2014;94(5):390-92. | Objective: To explore the clinical application of serum GP73 in the diagnosis and progress of hepatocellular carcinoma (HCC). Method(s): Enzyme-linked immunosorbent assay was employed to quantitatively detect serum GP73 in 59 HCC patients, 23 cases of hepatitis B virus (HBV)-related cirrhosis and 33 normal controls. The relationship between GP73 and diagnosis as well as progress of HCC was examined. Result(s): Significant differences existed when serum GP73 was categorized by Child-Pugh class A and B (P<0.01). There was no correlation between GP73 levels and other parameters including age, gender, BCLC staging, HBV infection, tumor size, extrahepatic metastasis and tumor numbers. The serum GP73 with a mean level of [127(147) mug/L, M(QR)] in HCC patients was significantly higher than that in normal controls or those with HBV-liver cirrhosis (P<0.01). Based on the ROC curve analysis, the cut-off value was 99 mug/L with 64.4% sensitivity and 96.4% specificity. The sensitivity of diagnosing HCC with GP73 plus AFP improved (P=0.025), but not in specificity. Serum GP73 and AFP did not change greatly in terms of response assessment in mRECIST from baseline to progressive disease (P=0.959, P=0.788). No correlation existed between baseline concentration of GP73 with AFP and time to progression (r=0.119, P=0.608; r=0.142, P=0.540). Conclusion(s): GP73 may become a potential serum marker for diagnosing HCC. And a combination of AFP yields better outcomes. The exact relationship between mRECIST and GP73 as well as AFP shall be proved by large-scale clinical trials. Copyright Â© 2014 by the Chinese Medical Association. |
| Zhao, Y., Zhang, L., Li, H., Cui, C. Clinical significance of serum GP73, AFP, and CA199 test in the diagnosis of hepatic cancer. Chinese Journal of Clinical Oncology 2013;40(1):29. | Objective: To investigate the clinical value of serum Glogi glycoprotein 73 (GP73) and its combination to alpha-fetoprotein (AFP) and CA199 for the diagnosis of primary hepatic cancer (PHC). Method(s): Sera from 110 patients (50 hepatic cancer, 20 hepatitis, 20 liver cirrhosis, and 20 malignant tumors of the digestive system except hepatic cancer) and 20 healthy controls were collected. Serum GP73 was analyzed by enzyme-linked immunosorbent assay. The levels of AFP and CA199 were quantified by electrochemiluminescence immunoassays. Result(s): The median serum GP73 concentration of PHC (104.4 mug/L) was higher than that of hepatitis (22.7 mug/L), liver cirrhosis (53.8 mug/L), malignant tumors of the digestive system except hepatic cancer (41.4 mug/L), and healthy controls (19.3 mug/L) significantly (P<0.05). The sensitivity, specificity, and accuracy to diagnose PHC by GP73 alone were 72.0%, 95.0% and 86.2%, respectively, and were higher than that of AFP or CA199 alone. GP73's sensitivity was highest (92.0%) for the parallel test of GP73, AFP, and CA199; its specificity was highest (100.0%) for the series-wound test of GP73, AFP, and CA199; and its accuracy was the highest (87.7%) for the parallel test of GP73 and AFP. The area under the ROC curve of GP73 was 0.824, which is higher than that of AFP or CA199 alone. The serum GP73 level was not significantly correlated with that of AFP or CA199. Conclusion(s): GP73 is a new effective marker in the diagnosis of PHC, and the combined examination of GP73, AFP, and CA199 can improve the diagnosis value of PHC. |
| Zheng, R. Q., Huang, D. M., Zhang, B., Su, Z. Z., Kudo, M. Comparison of ultrasonography, CT and MRI in the diagnosis of liver cirrhosis. World Chinese Journal of Digestology 2005;13(8):993-96. | AIM: To compare ultrasound (US), CT and MRI in the diagnosis of liver cirrhosis, and to select the best predictive signs for the diagnosis of liver cirrhosis by each technique. METHOD(S): Imaging findings on US, CT and MRI in 142 patients with hepatocellular carcinoma (<3 cm in diameter) were blindly reviewed by three doctors using a severity scoring system. Imaging findings included irregular or nodular liver surface, blunt liver edge, liver parenchyma abnormalities, liver morphological changes and portal hypertension. Results were compared with pathological results on non-tumor resected specimens. RESULT(S): Blunt liver edge, liver parenchyma abnormalities, and liver morphological changes were the best predictive signs for US diagnosis of liver cirrhosis. Irregular or nodular liver surface, liver parenchyma abnormalities and portal hypertension were the best predictive signs for MRI. Irregular or nodular liver surface, blunt liver edge, liver parenchyma abnormalities and portal hypertension were the best predictive signs for CT. The diagnostic |

| Ref | Abstract |
|---|---|
| | accuracies of liver cirrhosis by MRI, CT and US were 70.3%, 67.0%, 64.0%, the sensitivities were 86.7%, 84.3%, 52.4%, and the specificities were 53.9%, 52.9%, 73.5%, respectively. MRI and CT were slightly superior to US in the diagnosis of liver cirrhosis; however, there was no significant difference regarding the diagnostic accuracy among them. CONCLUSION(S): US, CT and MRI have different predictive signs, different imaging feature and diagnostic superiority. These imaging techniques all play important roles in the evaluation of liver cirrhosis. |
| Zhou, C. F. . World Chinese Journal of Digestology 2017;25(35):3161-66. | AIM To assess the clinical value of combined detection of four common tumor markers in benign and malignant hepatobiliary system diseases. METHODS Two hundred and seventy patients with benign and malignant hepatobiliary system diseases (hepatolithiasis, liver abscess, choledocholithiasis, cholecystitis, primary hepatocellular carcinoma, and cholangiocarcinoma) treated at our hospital from February 2015 to February 2017 were included. The levels of a-fetoprotein (AFP), carcino-embryonic antigen (CEA), carbohydrate antigen19-9 (CA19-9), and cancer antigen 125 (CA125) were detected in all patients by enzyme linked immunosorbent assay, and their value in disease diagnosis and curative effect monitoring was assessed. RESULTS There was no significant difference in serum levels of tumor markers between the normal control group and the benign liver disease group (P > 0.05). The levels of tumor markers in the malignant hepatobiliary disease group were significantly higher than those in the benign liver disease group (P < 0.05). In malignant liver diseases, the sensitivity, specificity, and accuracy of AFP were the highest among all single detections; compared with single detection, the sensitivity of combined detection was increased significantly (P < 0.05), but the specificity and accuracy were decreased (P > 0.05). In malignant biliary diseases, the sensitivity, specificity, and accuracy of CA19-9 were the highest among all single detections; compared with single detection, the sensitivity of combined detection was increased significantly (P < 0.05), but the specificity and accuracy were decreased (P > 0.05). Compared with the values before treatment, the levels of AFP, CEA, CA19-9, and CA125 in patients with malignant hepatobiliary diseases significantly decreased after treatment (P < 0.05). CONCLUSION Compared with single detection, the combined detection of tumor markers can significantly improve the value of diagnosis and curative effect monitoring in benign and malignant diseases of the hepatobiliary system.Copyright Â© The Author(s) 2017. Published by Baishideng Publishing Group Inc. All rights reserved. |
| Zhou, Q. & Zhang, Q. Differential diagnosis of primary hepatic carcinoma by detecting serum alpha-fetoprotein, tumor-associated substances and carcinoembryonic antigen. World Chinese Journal of Digestology 2010;18(18):1936-39. | AIM: To investigate the value of combined detection of serum alpha-fetoprotein (AFP), tumor-associated substances (TSGF) and carcinoembryonic antigen (CEA) in the diagnosis of primary hepatic carcinoma (PHC). METHOD(S): Serum samples from 100 patients with primary hepatic carcinoma (PHC group), 100 patients with benign liver disease (benign liver disease group) and 100 healthy individuals (control group) were used to detect the contents of AFP, TSGF and CEA by electrochemical luminescence immunoassay and biochemical colorimetric assay. RESULT(S): In the PHC group, the positive rate of combined detection of AFP, TSGF and CEA was 95%, significantly higher than those of detection of AFP (70%), TSGF (61%) or CEA (57%) alone (all P < 0.05). The sensitivity, specificity, accuracy, positive predictive value and negative predictive value of the combined detection method were significantly higher than those of detection of a single tumor marker (all P < 0.05). After treatment, serum levels of AFP, TSGF and CEA significantly declined in PHC patients when compared with pre-treatment values (all P < 0.01). CONCLUSION(S): Combined detection of AFP, TSGF and CEA can not only improve the diagnosis of PHC, especially in AFP-negative patients, but also help make a differential diagnosis between benign and malignant liver tumors. |
| Zhou, W., Guo, X., Cao, Z. G., Zhao, X. T. Detection of glypican 3 in serum and its clinical significance on the diagnosis of hepatocellular carcinoma. Tumor 2007;27(9):679-82. | Objective: To evaluate the clinical significance of detection of serum glypican 3 (GPC3) on the diagnosis of hepatocellular carcinoma. Method(s): Mouse anti-GPC3 (25-358 aa) monoclonal antibody and rabbit anti-GPC3 (379-393 as) antibody were used to establish the sandwich ELISA method for detecting serum GPC3 level in 364 HCC patients, 96 patients with chronic hepatitis, and 106 healthy subjects. Serum alpha-fetoprotein (AFP) level was detected by radioimmunoassay. Result(s): The mean concentration of serum GPC3 was 86.96 +/- 42.2 ng/mL, 11.7 +/- 12.23 ng/mL, and 6.04 +/- 9.21 ng/mL in HCC patients, chronic hepatitis patients, and healthy subjects, respectively. The difference between HCC patients and chronic hepatitis patients and healthy subjects was highly significant (P <0.001). But the difference between chronic hepatitis patients and healthy subjects. was not significant (P > 0.05). When the cutoff value was set at 30 ng/mL, the sensitivity and specificity of GPC3 in the diagnosis of hepatocellular carcinoma was 40% and 93%, respectively. Meanwhile, the positive rate of AFP was 49% and the positive rate of GPC3 was 40% in 151 HCC serum samples. The detection rate of HCC increased to 72% by combined detection of AFP with GPC3. Conclusion(s): Serum GPC3 can be used as a marker for the early diagnosis of HCC in clinic. |
| Zhou, X. L., Mi, Y. Q., Xu, L., Liu, Y. G., Zhong, Y. Clinical value of protein induced by vitamin K absence or antagonist-II in diagnosis of hepatocellular carcinoma. World Chinese Journal of Digestology 2018;26(7):425-33. | AIM To evaluate the clinical value of protein induced by vitamin K absence or antagonist-II (PIVKA-II) and alpha-fetoprotein (AFP) in the diagnosis of hepatocellular carcinoma (HCC). METHODS A total of 285 patients with HCC, 60 patients with liver cirrhosis, 60 patients with chronic viral hepatitis (38 with chronic hepatitis B and 22 with chronic hepatitis C), 30 patients with other chronic hepatopathies (14 with drug hepatitis, 8 with alcoholic hepatitis, and 8 with non-alcoholic steatohepatitis), 25 patients with gastric cancer or colorectal cancer, and 40 healthy controls were enrolled at the Tianjin Second People's Hospital from April 2016 to April 2017. Their serum samples were collected and serum levels of PIVKA-II andAFP were measured by electrochemiluminescence immunoassay (ELICA). The area under the ROC curve (AUC), sensitivity, and specificity of PIVKA-II and AFP, alone or in combination, in the diagnosis of HCC were analyzed. The correlation between PIVKA-II and clinical characteristics (such as tumor size and BCLC stage) was also analyzed. RESULTS There were significant differences in serum levels of AFP and PIVKA-II between the HCC group and the other groups (P < 0.01). Serum levels of |

| Ref | Abstract |
|---|---|
| | both PIVKA- II and AFP in the HCC group were significantly higher than those in the other groups (P < 0.01). For the diagnosis of HCC, PIVKA-II had a higher sensitivity (84.2% vs 70.9%, P < 0.001) and specificity (81.4% vs 65.6%, P < 0.001) than AFP. When combining the serum levels of AFP and PIVKA-II, the sensitivity for the diagnosis of HCC was higher than PIVKA-II alone (89.5% vs 84.2%), but there was no statistical significance (P > 0.05). PIVKA-II in differentiating HCC cases from controls was also better than AFP (AUC: 0.865 vs 0.768, P < 0.001). In the HCC group, serum levels of PIVKA- II were associated with sex, tumor number, and microvascular invasion, but had no association with age, viral infection, tumor diameter, portal vein tumor thrombus, or Child-Pugh classification. Serum levels of PIVKA-II increased with increased BCLC stage (H = 48.70, P < 0.001). CONCLUSION Serum PIVKA-II is more efficient than AFP in the diagnosis of HCC. In the HCC group, PIVKA-II serum levels are associated with sex, tumor number, and microvascular invasion.Copyright Â© The Author(s) 2018. Published by Baishideng Publishing Group Inc. All rights reserved. |

# Appendix S4 Search strategies

## S4.1. Ovid MEDLINE

| # | String | N |
|---|---|---|
| 1 | exp Liver Cirrhosis/ | 98723 |
| 2 | exp Hepatitis/ or Hepatitis B/ or Hepatitis C/ | 179330 |
| 3 | Liver Diseases, Alcoholic/ | 6011 |
| 4 | exp Fatty Liver/ | 42365 |
| 5 | Hemochromatosis/ or h?emochromatos*.ti,ab. | 10581 |
| 6 | Hepatolenticular Degeneration/ or ((wilson* adj (disease or syndrome)) or Wilsonian).ti,ab. | 8447 |
| 7 | Liver Cirrhosis, Biliary/ or primary biliary.ti,ab. | 11782 |
| 8 | cirrho*.ti,ab. | 110133 |
| 9 | hepatiti*.ti,ab. | 234027 |
| 10 | (chronic adj3 liver).ti,ab. | 36451 |
| 11 | (HBV or HCV).ti,ab. | 99542 |
| 12 | ((fatty adj liver) or NAFLD).ti,ab. | 38350 |
| 13 | (steatohepat* or NASH).ti,ab. | 16945 |
| 14 | (benign adj3 liver).ti,ab. | 2079 |
| 15 | or/1-14 | 447023 |
| 16 | Carcinoma, Hepatocellular/ | 99238 |
| 17 | hepatocarcinoma.ti,ab. | 4176 |
| 18 | hepatoma.ti,ab. | 27763 |
| 19 | ((liver or hepat*) adj (carcinom* or cancer* or neoplasm* or malign* or tumo*)).ti,ab. | 145736 |
| 20 | HCC.ti,ab. | 69071 |
| 21 | or/16-20 | 187617 |
| 22 | diagnosis/ or early diagnosis/ or "early detection of cancer"/ | 81491 |
| 23 | cancer screening/ | 34630 |
| 24 | diagnos*.ti,ab. | 2840619 |
| 25 | screen*.ti,ab. | 895790 |
| 26 | surveil*.ti,ab. | 215357 |
| 27 | "Sensitivity and Specificity"/ | 365619 |
| 28 | "ROC Curve"/ | 69366 |
| 29 | (sensitiv* or insensitiv*).ti,ab. | 1612086 |
| 30 | (specific* or unspecific*).ti,ab. | 3565021 |
| 31 | (marker* or biomarker*).ti,ab. | 1154732 |
| 32 | reference standard.ab. | 17486 |
| 33 | predictive value.ti,ab. | 107788 |
| 34 | false positive*.ti,ab. | 65612 |
| 35 | false negative*.ti,ab. | 37091 |
| 36 | true positive*.ti,ab. | 9413 |
| 37 | true negative*.ti,ab. | 3899 |
| 38 | ((receiver adj operating) or ROC).ti,ab. or "ROC Curve"/ | 160040 |
| 39 | ((area adj under adj the adj4 curve) or AUC or c-statistic or harrell* c).ti,ab. | 160398 |
| 40 | Carcinoma, Hepatocellular/bl, di, dg or Liver Neoplasms/bl, di, dg | 41832 |

| # | String | N |
|---|---|---|
| 41 | [any 3 of 22-40][1] | 593268 |
| 42 | 15 and 21 and 41 | 5771 |
| 43 | ((surveil* or detect* or differentiat* or discriminat* or distinguish*) adj2 (hepatocellular or hepatoma* or HCC)).ti,ab. | 6806 |
| 44 | 42 or 43 | 10876 |
| 45 | alpha-Fetoproteins/ | 16638 |
| 46 | AFP.ti,ab. | 15696 |
| 47 | f?etoprotein*.ti,ab. | 20323 |
| 48 | or/45-47 | 31340 |
| 49 | Ultrasonography/mt | 24535 |
| 50 | ultraso?n*.ti,ab. | 425939 |
| 51 | sonogra*.ti,ab. | 59781 |
| 52 | echogra*.ti,ab. | 10450 |
| 53 | or/49-52 | 469855 |
| 54 | (Magnetic resonance imaging or MRI).ti,ab. or Magnetic resonance imaging/ | 637311 |
| 55 | ((Compute* adj tomography) or CT).ti,ab. or Tomography, X-Ray Computed/ | 765627 |
| 56 | 54 or 55 | 1270027 |
| 57 | (liquid adj biopsy).ti,ab. | 4401 |
| 58 | (circulating adj tumo?r).ti,ab. | 12369 |
| 59 | (cell adj free).ti,ab. | 45029 |
| 60 | (cfDNA* or (cf adj DNA) or ctDNA* or (ct adj DNA) or microRNA or (micro adj RNA*) or mRNA* or miRNA* or mi-RNA*).ti,ab. | 671837 |
| 61 | exosom*.ti,ab. | 20838 |
| 62 | Liquid Biopsy/ or Neoplastic Cells, Circulating/ or Biomarkers, Tumor/ | 183083 |
| 63 | or/57-62 | 887529 |
| 64 | ((carboxy adj prothrombin) or carboxyprothrombin or DCP or PIVKA*).ti,ab. | 5142 |
| 65 | ((((predict* or diagnos* or detect*) and (tool* or model* or score* or algorithm*)) or (GALAD or Doylestown or multitarget HCC)) and validat*).ti,ab. | 174497 |
| 66 | 48 or 53 or 56 or 63 or 64 or 65 | 2686479 |
| 67 | 44 and 66 | 6984 |
| 68 | exp ULTRASONOGRAPHY/ | 477584 |
| 69 | (ultrason* or ultrasound* or echograph* or echotomograph* or doppler* or B-mode or B-scan or grey*scale).mp. | 630037 |
| 70 | 68 or 69 | 745965 |
| 71 | exp alpha-Fetoproteins/ | 16638 |
| 72 | ((alpha or alfa) and (fetoprotein* or foetoprotein or fetalprotein)).mp. | 26333 |
| 73 | 71 or 72 | 26333 |
| 74 | exp "Diagnostic Techniques and Procedures"/ | 7730261 |
| 75 | diagnos*.mp. | 5683885 |
| 76 | 74 or 75 | 10514797 |
| 77 | exp Carcinoma, Hepatocellular/ | 99238 |
| 78 | exp Liver Neoplasms/ | 185892 |
| 79 | (((liver or hepato*) and (carcinom* or cancer* or neoplasm* or malign* or tumo*)) or HCC).mp. | 383638 |
| 80 | 77 or 78 or 79 | 383648 |
| 81 | exp Liver Diseases/ | 608267 |
| 82 | (liver or hepat* or cirrhosis or fibrosis).mp. | 1716457 |
| 83 | 81 or 82 | 1736574 |

[1] To operationalise this, we enumerate all possible 3-way combinations of lines and join them with OR operators; we omit the resulting 3 pages of text from this summary for brevity.

| # | String | N |
|---|---|---|
| 84 | 70 or 73 | 769143 |
| 85 | 76 and 80 and 83 and 84 | 24332 |
| 86 | limit 85 to (humans and ("all adult (19 plus years)" or "young adult (19 to 24 years)" or "adult (19 to 44 years)" or "young adult and adult (19-24 and 19-44)" or "middle age (45 to 64 years)" or "middle aged (45 plus years)" or "all aged (65 and over)" or "aged (80 and over)")) | 13902 |
| 87 | (44 and (48 or 53)) not 86 | 2089 |
| 88 | 44 and (56 or 63 or 64 or 65) | 5359 |
| 89 | 87 or 88 | 6279 |

## S4.2. EMBASE via Ovid

| # | String | N |
|---|---|---|
| 1 | receiver operating characteristic/ | 175095 |
| 2 | exp Liver Cirrhosis/ | 177921 |
| 3 | exp Hepatitis/ or Hepatitis B/ or Hepatitis C/ | 336830 |
| 4 | exp alcohol liver disease/ | 29406 |
| 5 | exp Fatty Liver/ | 99177 |
| 6 | Hemochromatosis/ or h?emochromatos*.ti,ab. | 14422 |
| 7 | Wilson disease/ or ((wilson* adj (disease or syndrome)) or Wilsonian).ti,ab. | 12221 |
| 8 | biliary cirrhosis/ or primary biliary.ti,ab. | 15874 |
| 9 | cirrho*.ti,ab. | 168427 |
| 10 | hepatiti*.ti,ab. | 321955 |
| 11 | (chronic adj3 liver).ti,ab. | 56488 |
| 12 | (HBV or HCV).ti,ab. | 161454 |
| 13 | ((fatty adj liver) or NAFLD).ti,ab. | 60594 |
| 14 | (steatohepat* or NASH).ti,ab. | 31425 |
| 15 | (benign adj3 liver).ti,ab. | 2890 |
| 16 | or/2-15 | 675913 |
| 17 | exp liver cell carcinoma/ | 182729 |
| 18 | hepatocarcinoma.ti,ab. | 5643 |
| 19 | hepatoma.ti,ab. | 31920 |
| 20 | ((liver or hepat*) adj (carcinom* or cancer* or neoplasm* or malign* or tumo*)).ti,ab. | 205363 |
| 21 | HCC.ti,ab. | 107649 |
| 22 | or/17-21 | 275395 |
| 23 | cancer diagnosis/ | 158698 |
| 24 | cancer screening/ | 90025 |
| 25 | diagnos*.ti,ab. | 4093334 |
| 26 | screen*.ti,ab. | 1247053 |
| 27 | surveil*.ti,ab. | 294460 |
| 28 | "Sensitivity and Specificity"/ | 440725 |
| 29 | receiver operating characteristic/ | 175095 |
| 30 | (sensitiv* or insensitiv*).ti,ab. | 2006998 |
| 31 | (specific* or unspecific*).ti,ab. | 4474471 |
| 32 | (marker* or biomarker*).ti,ab. | 1656034 |
| 33 | reference standard.ab. | 23990 |
| 34 | predictive value.ti,ab. | 163494 |
| 35 | false positive*.ti,ab. | 88458 |

| # | String | N |
|---|---|---|
| 36 | false negative*.ti,ab. | 52116 |
| 37 | true positive*.ti,ab. | 13951 |
| 38 | true negative*.ti,ab. | 6154 |
| 39 | ((receiver adj operating) or ROC).ti,ab. or receiver operating characteristic/ | 246119 |
| 40 | ((area adj under adj the adj4 curve) or AUC or c-statistic or harrell* c).ti,ab. | 245134 |
| 41 | liver cell carcinoma/di or liver tumor/di | 26650 |
| 42 | [any 3 of 23-41][2] | 899845 |
| 43 | 16 and 22 and 42 | 10554 |
| 44 | ((surveil* or detect* or differentiat* or discriminat* or distinguish*) adj2 (hepatocellular or hepatoma* or HCC)).ti,ab. | 10018 |
| 45 | 43 or 44 | 17730 |
| 46 | alpha fetoprotein/ | 39912 |
| 47 | AFP.ti,ab. | 25439 |
| 48 | f?etoprotein*.ti,ab. | 26193 |
| 49 | or/46-48 | 54717 |
| 50 | echography/ | 360592 |
| 51 | ultraso?n*.ti,ab. | 619757 |
| 52 | sonogra*.ti,ab. | 83750 |
| 53 | echogra*.ti,ab. | 13826 |
| 54 | or/50-53 | 813671 |
| 55 | (Magnetic resonance imaging or MRI).ti,ab. or nuclear magnetic resonance imaging/ | 1070883 |
| 56 | ((Compute* adj tomography) or CT).ti,ab. or x-ray computed tomography/ | 934379 |
| 57 | 55 or 56 | 1832401 |
| 58 | (liquid adj biopsy).ti,ab. | 7627 |
| 59 | (circulating adj tumo?r).ti,ab. | 21512 |
| 60 | (cell adj free).ti,ab. | 52312 |
| 61 | (cfDNA* or (cf adj DNA) or ctDNA* or (ct adj DNA) or microRNA or (micro adj RNA*) or mRNA* or miRNA* or mi-RNA*).ti,ab. | 848376 |
| 62 | exosom*.ti,ab. | 29487 |
| 63 | Liquid Biopsy/ or tumor marker/ | 102335 |
| 64 | or/58-63 | 1008317 |
| 65 | ((carboxy adj prothrombin) or carboxyprothrombin or DCP or PIVKA*).ti,ab. | 7167 |
| 66 | ((((predict* or diagnos* or detect*) and (tool* or model* or score* or algorithm*)) or (GALAD or Doylestown or multitarget HCC)) and validat*).ti,ab. | 268291 |
| 67 | 49 or 54 or 57 or 64 or 65 or 66 | 3708821 |
| 68 | 45 and 67 | 11379 |
| 69 | exp echography/ | 922236 |
| 70 | (ultrason* or ultrasound* or echograph* or echotomograph* or doppler* or B-mode or B-scan or grey*scale).mp. | 1050807 |
| 71 | 69 or 70 | 1375335 |
| 72 | exp alpha fetoprotein/ | 39912 |
| 73 | ((alpha or alfa) and (fetoprotein* or foetoprotein or fetalprotein)).mp. | 45039 |
| 74 | 72 or 73 | 45039 |
| 75 | exp diagnostic test/ | 1069941 |
| 76 | diagnos*.mp. | 7172071 |
| 77 | 75 or 76 | 7704686 |
| 78 | exp liver cell carcinoma/ | 182729 |

[2] To operationalise this, we enumerate all possible 3-way combinations of lines and join them with OR operators; we omit the resulting 3 pages of text from this summary for brevity.

| # | String | N |
|---|---|---|
| 79 | exp liver tumor/ | 323040 |
| 80 | (((liver or hepato*) and (carcinom* or cancer* or neoplasm* or malign* or tumo*)) or HCC).mp. | 622958 |
| 81 | 78 or 79 or 80 | 630065 |
| 82 | exp liver disease/ | 1120307 |
| 83 | (liver or hepat* or cirrhosis or fibrosis).mp. | 2377797 |
| 84 | 82 or 83 | 2405100 |
| 85 | 71 or 74 | 1411969 |
| 86 | 77 and 81 and 84 and 85 | 48317 |
| 87 | limit 86 to (human and (adult <18 to 64 years> or aged <65+ years>)) | 27991 |
| 88 | limit 87 to dd=19700101-20200601 | 11543 |
| 89 | (45 and (49 or 54)) not 88 | 6093 |
| 90 | 45 and (57 or 64 or 65 or 66) | 8007 |
| 91 | 89 or 90 | 10752 |

## S4.3. Cochrane Database of Systematic Reviews, Issue 8 of 12 August 2022

| # | String | N |
|---|---|---|
| 1 | MeSH descriptor: [Liver Cirrhosis] explode all trees | 3189 |
| 2 | MeSH descriptor: [Hepatitis, Alcoholic] explode all trees | 155 |
| 3 | MeSH descriptor: [Hepatitis B] explode all trees | 2909 |
| 4 | MeSH descriptor: [Hepatitis C] explode all trees | 3416 |
| 5 | MeSH descriptor: [Liver Diseases, Alcoholic] explode all trees | 503 |
| 6 | MeSH descriptor: [Hemochromatosis] explode all trees | 48 |
| 7 | MeSH descriptor: [Hepatolenticular Degeneration] explode all trees | 37 |
| 8 | cirrho*:ti,ab | 10373 |
| 9 | hepatiti*:ti,ab | 19320 |
| 10 | (chronic NEXT/3 liver):ti,ab | 2282 |
| 11 | (HBV or HCV):ti,ab | 10550 |
| 12 | ((fatty NEXT liver) or NAFLD):ti,ab | 3465 |
| 13 | (steatohepat* or NASH):ti,ab | 1833 |
| 14 | (benign NEXT/3 liver):ti,ab | 48 |
| 15 | h?emochromatos*:ti,ab | 168 |
| 16 | ((wilson* NEXT (disease or syndrome)) or Wilsonian):ti,ab | 129 |
| 17 | "primary biliary":ti,ab | 899 |
| 18 | #1 or #2 or #3 or #4 or #5 or #6 or #7 or #8 or #9 or #10 or #11 or #12 or #13 or #14 or #15 or #16 or #17 | 34017 |
| 19 | MeSH descriptor: [Carcinoma, Hepatocellular] explode all trees | 2003 |
| 20 | hepatocarcinoma:ti,ab | 72 |
| 21 | hepatoma:ti,ab | 128 |
| 22 | ((liver or hepat*) NEXT (carcinom* or cancer* or neoplasm* or malign* or tumo*)):ti,ab | 6664 |
| 23 | HCC:ti,ab | 3738 |
| 24 | #19 or #20 or #21 or #22 or #23 | 7374 |
| 25 | MeSH descriptor: [Diagnosis] explode all trees | 353620 |
| 26 | MeSH descriptor: [Early Detection of Cancer] explode all trees | 1479 |
| 27 | diagnos*:ti,ab | 169539 |
| 28 | screen*:ti,ab | 82378 |
| 29 | surveil*:ti,ab | 8303 |

| | | |
|---|---|---|
| 30 | MeSH descriptor: [Sensitivity and Specificity] explode all trees | 16186 |
| 31 | MeSH descriptor: [ROC Curve] explode all trees | 1209 |
| 32 | (sensitiv* or insensitiv*):ti,ab | 71520 |
| 33 | (specific* or unspecific*):ti,ab | 139085 |
| 34 | (marker* or biomarker*):ti,ab | 80267 |
| 35 | reference standard:ab | 8611 |
| 36 | predictive value:ti,ab | 9063 |
| 37 | false positive*:ti,ab | 145163 |
| 38 | false negative*:ti,ab | 90030 |
| 39 | true positive*:ti,ab | 145156 |
| 40 | true negative*:ti,ab | 90030 |
| 41 | ((receiver NEXT operating) or ROC):ti,ab | 5730 |
| 42 | ((area NEXT under NEXT the NEXT/4 curve) or AUC or c-statistic or harrell* c):ti,ab | 31447 |
| 43 | #25 or #26 or #27 or #28 or #29 or #30 or #31 or #32 or #33 or #34 or #35 or #36 or #37 or #38 or #39 or #40 or #41 or #42 | 819364 |
| 44 | ((surveil* or detect* or differentiat* or discriminat* or distinguish*) NEXT/2 (hepatocellular or hepatoma* or HCC)):ti,ab | 99 |
| 45 | #18 and #24 and #43 | 1373 |
| 46 | #44 or #45 | 1420 |

# Appendix S5 Detailed methods for quantitative synthesis

For brevity, in what follows we refer to:

- Tests that produce a dichotomous result, or tests reported at only a single diagnostic threshold in each study, as ‘binary’ tests.
- Tests that produce a continuous result, with some studies reporting accuracy at more than one explicit threshold value as ‘continuous’ tests.

## S5.1. NMA of diagnostic test accuracy, accounting for thresholds for continuous tests

We fitted a new NMA-DTA model that borrows ideas from the arm-based NMA model for binary tests proposed by Nyaga *et al*[2] and the model for (single-test) meta-analysis of continuous tests across all thresholds by Jones *et al*.[3]

For binary tests, we assumed independent binomial likelihoods for the number of true and false positives. Between-studies, an analysis of variance (ANOVA) model structure was assumed for sensitivity and false positive fraction (FPF, defined as 1−specificity) on the log-odds scale, with study-level random effects allowing for correlation across the accuracy of ≥2 tests evaluated in the same study, and additional study-by-test interactions encapsulating the accuracy of each individual test.

For continuous tests, independent multinomial likelihoods were assumed for the full contingency table of counts within the HCC and HCC-free populations. Between-studies, we assumed a similar model structure as that described by Jones *et al.* (2019), but with the addition of additive study-level random effects, and the assumption that the underlying continuous test results follow a standardised and centred (around the most commonly reported threshold) logistic distribution. For the underlying test results, a log transformation was adopted – preliminary analyses using a more flexible (Box–Cox) transformation showed minimal differences from a simple log transformation, so we preferred the latter for parsimony and computational ease.

The study-level random effects in the HCC and HCC-free populations are shared across both binary and continuous tests reported on in the same study. A bivariate normal distribution was assumed for these, with means equal to zero. As many of the binary tests in the network were

only evaluated in one study, providing no information to estimate between-study variance components for these tests, we also included a hierarchical model for these variance components across the binary tests[4]. For more details and a full mathematical description of the model, see Derezea *et al.* (2026)[5].

We also ranked estimates of sensitivity and specificity across tests in the network. Ranks are presented with 95% CrIs. As rank statistics are known to be sensitive to imprecise data[6], we only estimated ranks for tests appearing in ≥2 studies. For continuous tests reported across more than one threshold, we only evaluated rank statistics for the most commonly reported threshold.

## S5.2. Stage-stratified meta-analysis of sensitivity

### S5.2.1. Methods

For each test, we used a model that combines stage-specific subgroup data (e.g. sensitivity for $BCLC_0$) with data incorporating multiple or all stages (e.g. sensitivity for $BCLC_{0A}$ or sensitivity for any HCC, in studies that report proportion of people with HCCs of each stage). We report stage-stratified estimates of sensitivity for each test with subgroup data from ≥1 study and ≥4 studies providing some relevant data. For full details and a mathematical description of the model, see Derezea *et al.* (2026b)[7].

### S5.2.2. Mapping stage categories

As different studies reported HCC size and/or stage information in different ways, to allow exploration of test sensitivity by stage, we first mapped this information to approximate Barcelona Clinic Liver Cancer (BCLC) staging system categories. We aimed to categorise studies or subgroups as (approximately) equivalent to 3 BCLC stages:

- BCLC 0: (very early stage) single nodule <=2cm
- BCLC A: (early stage) single, or up to 3 nodules all <= 3cm
- BCLC B/C/D: (intermediate/advanced/terminal stage) single or multiple nodules all >3cm

The following list shows the subgroups that fall within the 3 main BCLC categories described above, as well a fourth overlapping category between BCLC 0 and BCLC A.

### Table S5.1 Mapping for HCC size / stage categories

**1. BCLC 0 (very early stage): single nodule <=2 cm**

- HCC <1cm
- HCC <2.0cm
- HCC 1-2cm
- HCC less than or equal to 2.0cm
- Single nodule <2cm
- Very early HCC (BCLC 0)

**2.Overlapping BCLC 0 / BCLC A (i.e. everyone meets criteria for A; some unknown proportion could also be classified as 0)**

- Early HCC (BCLC 0/A)
- Early HCC (0.6-2.5cm)
- Early HCC (Milan [1 tumour <5 cm or 2–3 tumours each <3 cm])
- Early HCC (single tumour <3cm)
- Early HCC (staging system not reported)
- HCC <3cm
- HCC <5cm
- HCC less than or equal to 3cm
- HCC less than or equal to 5cm
- Nodules less than or equal to 3cm and less than or equal to 3 nodules
- Single nodule
- Single nodule <3cm
- Single nodule <3cm or no. of nodules less than or equal to 2 and the sum of maximum diameters less than or equal to 3cm
- Single nodule less than or equal to 5cm
- TNM stage I
- TNM stage I or II

**3. BCLC A (early stage): single, or up to 3 nodules all <= 3cm (i.e. not including people who meet criteria for BCLC 0)**

- Early HCC (BCLC A)
- HCC 2.1-3.0cm
- HCC 2-4cm
- HCC 2-5cm
- HCC 3.1-5.0cm
- Single lesion >2 cm or 2-3 lesions each <3 cm
- Single nodule more than or equal to 3cm
- TNM stage II

**4. BCLC B/C/D (intermediate/advanced/terminal, all grouped together)**

- Advanced HCC (BCLC C)
- Advanced HCC (stages III and IV; staging system not reported – presumably TNM)
- End-stage HCC (BCLC D)
- Intermediate HCC (BCLC B)
- Late HCC (BCLC B/C)
- Late HCC (BCLC B-D)
- Late HCC (beyond Milan criteria)
- Metastatic HCC
- Multiple nodules
- Multiple nodules or diffuse
- Progressed HCC (staging system not reported)
- TNM stage III
- TNM stage III or IV
- TNM stage IV

## S5.3. Meta-regression

Meta-regression was used to explore between-study heterogeneity for each test separately. For binary tests, we included study characteristics as covariates acting on the logit transformed sensitivity and specificity in the bivariate model. This formulation assumes a linear relationship between the study characteristic (e.g. mean participant age) and the logit transformed sensitivity and specificity. For example, the regression coefficient for mean participant age represents the estimated change in logit sensitivity and specificity for each one year increase in average age.

For continuous tests, we explored evidence for the study level covariates acting on the location parameters of the Jones model[3].

## Appendix S6 PRISMA flow diagram

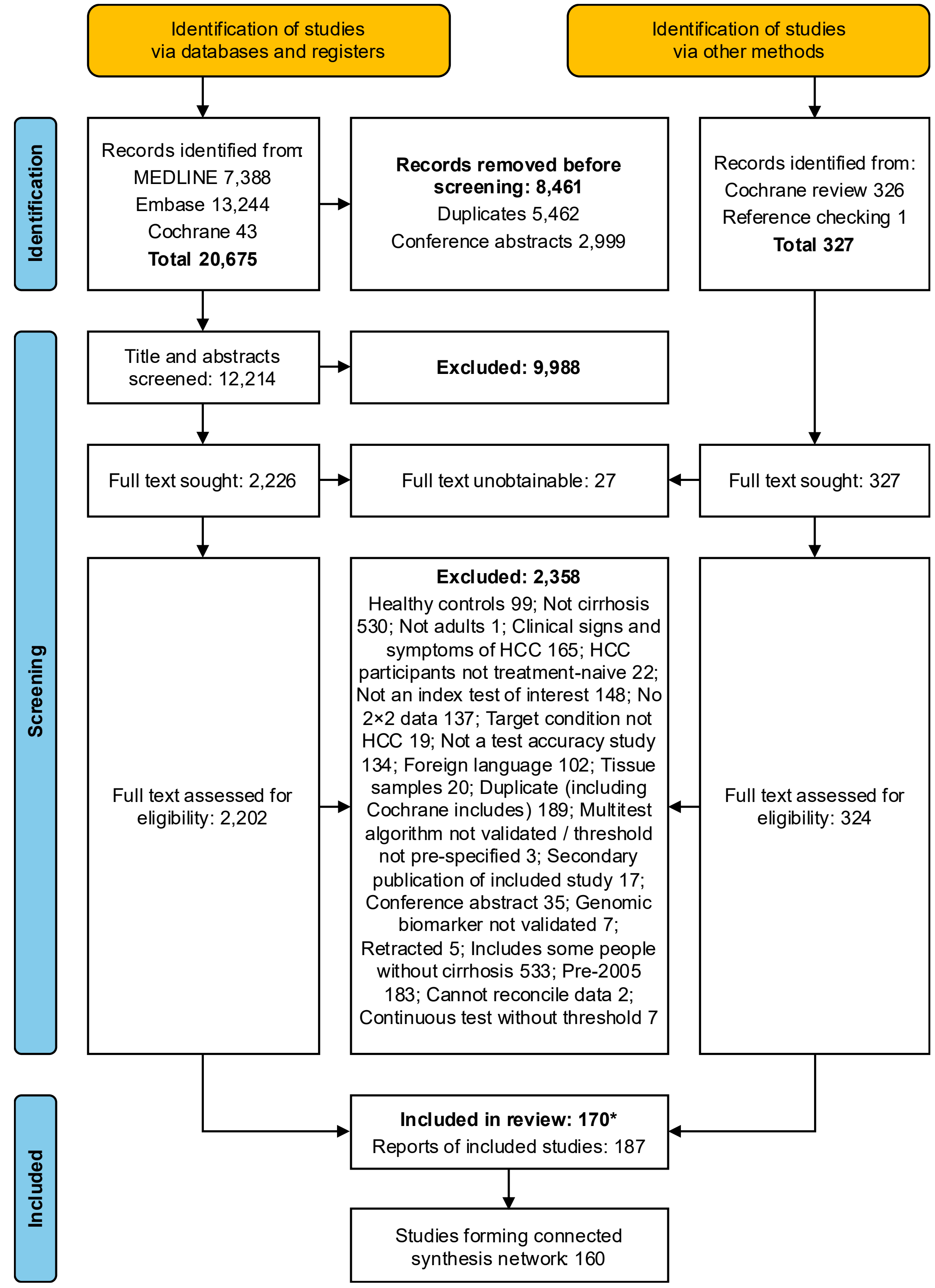


* *Total included studies is 2 greater than full-text papers assessed minus exclusions because 2 papers[8,9] report 2 separate cohorts that we count independently as they have different designs*

**Figure S6.1 Flow diagram documenting the number of studies at each stage of the review and reasons for exclusion**

## Appendix S7 Excluded studies

| Reference | Reason for exclusion |
|---|---|
| Abbas, E., Shaker, O., El Aziz, G. A., Ramadan, H. Epidermal growth factor gene polymorphism 61A/G in patients with chronic liver disease for early detection of hepatocellular carcinoma: A pilot study. European Journal of Gastroenterology & Hepatology 2012;():n. pag.. | Does not meet full eligibility criteria: includes people without cirrhosis |
| Abd Alkhalik Basha, M. & Abd El Aziz El Sammak, D. Diagnostic efficacy of the Liver Imaging-Reporting and Data System (LI-RADS) with CT imaging in categorising small nodules (10-20 mm) detected in the cirrhotic liver at screening ultrasound. Clinical radiology 2017;72(10):901.e1-11. | Exclude on population: clinical signs and symptoms of HCC |
| Abd El Gawad, Iman A, Mossallam, Ghada I, Radwan, Noha H, Elzawahry, Heba M. Comparing prothrombin induced by vitamin K absence-II (PIVKA-II) with the oncofetal proteins glypican-3, Alpha feto protein and carcinoembryonic antigen in diagnosing hepatocellular carcinoma among Egyptian patients. Journal of the Egyptian National Cancer Institute 2014;26(2):79-85. | Does not meet full eligibility criteria: includes people without cirrhosis |
| Abd El Gawad, Iman A., Mossallam, Ghada I., Radwan, Noha H., Elzawahry, Heba M. Comparing prothrombin induced by vitamin K absence-II (PIVKA-II) with the oncofetal proteins glypican-3, Alpha feto protein and carcinoembryonic antigen in diagnosing hepatocellular carcinoma among Egyptian patients. Journal of the Egyptian National Cancer Institute 2014;26(2):79-85. | Duplicate (including Cochrane includes) |
| Abd El Gwad, Asmaa, Matboli, Marwa, El-Tawdi, Ahmed, Habib, Eman K., Shehata, Hanan, Ibrahim, Doaa. Role of exosomal competing endogenous RNA in patients with hepatocellular carcinoma. Journal of cellular biochemistry 2018;119(10):8600-10. | Exclude on population: not cirrhosis |
| Abd El-Baky, R. M., Hetta, H. F., Koneru, G., Ammar, M., Shafik, E. A., Mohareb, D. A., et al. Impact of interleukin IL-6 rs-1474347 and IL-10 rs-1800896 genetic polymorphisms on the susceptibility of HCV-infected Egyptian patients to hepatocellular carcinoma. Immunologic Research 2020;68(3):118-25. | Does not meet full eligibility criteria: includes people without cirrhosis |
| Abdalla, Moemen Ak. Promising Candidate Urinary MicroRNA Biomarkers for the Early Detection of Hepatocellular Carcinoma among High-Risk Hepatitis C Virus Egyptian Patients. Journal of Cancer 2012;3():19-31. | Exclude on population: not cirrhosis |
| Abdalla, Moemen Ak. Promising Urinary Protein Biomarkers for the Early Detection of Hepatocellular Carcinoma among High-Risk Hepatitis C Virus Egyptian Patients. Journal of Cancer 2012;3():390-403. | Exclude on population: not cirrhosis |
| Abdel Allah, H. M. M., Zahran, W. E., El-Masry, S. A., El-Bendary, M. Association of MTHFR and TYMS gene polymorphisms with the susceptibility to HCC in Egyptian HCV cirrhotic patients. Clinical and Experimental Medicine 2022;22(2):257-67. | Exclude on study design: not a 1- or 2-gate test accuracy study |
| Abdel Ghafar, Muhammad Tarek, Elkhouly, Reham A., Elnaggar, Mohammed H., Mabrouk, Mohamed M., Darwish, Sara A., Younis, Reham L. Utility of serum neuropilin-1 and angiopoietin-2 as markers of hepatocellular carcinoma. Journal of investigative medicine : the official publication of the American Federation for Clinical Research 2021;69(6):1222-29. | Does not meet full eligibility criteria: includes people without cirrhosis |
| Abdelaziz, A. O., Nabil, M. M., Omran, D. A., Abdelmaksoud, A. H., Asem, N., Shousha, H. I., Elbaz, T. M. Hepatocellular Carcinoma Multidisciplinary Clinic-Cairo University (HMC-CU) score: A new simple score for diagnosis of HCC. Arab Journal of Gastroenterology 2020;21(2):102-05. | Unvalidated model / threshold not pre-specified |
| Abdelaziz, A. O., Shousha, H. I., Said, E. M., Soliman, Z. A., Shehata, A. A., Nabil, M. M., et al. Evaluation of liver steatosis, measured by controlled attenuation parameter, in patients with hepatitis C-induced advanced liver fibrosis and hepatocellular carcinoma. European Journal of Gastroenterology and Hepatology 2018;30(11):1384-88. | Exclude on index test |
| Abdelgawad, Iman Attia, Mossallam, Ghada Ibrahim, Radwan, Noha Hassan, Elzawahry, Heba Mohammed. Can Glypican3 be diagnostic for early hepatocellular carcinoma among Egyptian patients? Asian Pacific journal of cancer prevention : APJCP 2013;14(12):7345-49. | Exclude on population: healthy controls |

| Reference | Reason for exclusion |
| --- | --- |
| Abdelgawad, Iman Attia. Clinical utility of simple non-invasive liver fibrosis indices for predicting hepatocellular carcinoma (HCC) among Egyptian patients. Journal of clinical pathology 2015;68(2):154-60. | Exclude on population: healthy controls |
| Abdelgawad, Mohamed Essameldin, Darwish, Hossam, Nabawy, Mai M. Development of novel score based on Angiogenic panel for accurate diagnosis of hepatocellular carcinoma among hepatitis C virus high-risk patients. Infection, genetics and evolution : journal of molecular epidemiology and evolutionary genetics in infectious diseases 2020;85():104572. | Duplicate (including Cochrane includes) |
| Abdelghany, Amani M, Rezk, Nasser Sadek, Osman, Mona Mostafa, Hamid, Amira I, Al-Breedy, Ashraf Mohammad. Using Lamin B1 mRNA for the early diagnosis of hepatocellular carcinoma: a cross-sectional diagnostic accuracy study. F1000Research 2018;7(101594320):1339. | Exclude on population: not cirrhosis |
| Abdel-Hamid N.M., Wahid A.M., Anbar N.H. Estimation of des-gamma-carboxy prothrombin with alpha fetoprotein elevates the diagnostic performance of hepatocellular carcinoma among upper Egyptian hepatitis C patients. Research Journal of Pharmaceutical, Biological and Chemical Sciences 2014;5(1):821-31. | Exclude on population: not cirrhosis |
| Abdel-Hamid, N. M., Wahid, A. M., Anbar, N. H. Estimation of des-gamma-carboxy prothrombin with alpha fetoprotein elevates the diagnostic performance of hepatocellular carcinoma among upper Egyptian hepatitis C patients. Research Journal of Pharmaceutical, Biological and Chemical Sciences 2014;5(1):821-31. | Exclude on population: not cirrhosis |
| Abdelkhalek, Zeinab Sayed, Abdalla, Mohamed Shehata, Fathy, Mona Mohamed, Elbaz, Tamer Mahmoud, Abdelaziz, Ashraf Omar, Nabeel, Mohamed Mahmoud, et al. Role of circulating microRNA-21 and microRNA-215 in the diagnosis of hepatitis C related hepatocellular carcinoma. Journal of infection in developing countries 2021;15(7):997-1003. | Exclude on population: not cirrhosis |
| Abdel-Razik, Ahmed, Elhelaly, Rania, Elzehery, Rasha, El-Diasty, Amany, Abed, Sally, Elhammady, Dina. Could serotonin be a potential marker for hepatocellular carcinoma? A prospective single-center observational study. European journal of gastroenterology & hepatology 2016;28(5):599-605. | Duplicate (including Cochrane includes) |
| Abdelsameea, Eman, Nada, Ali, Omar, Nabil, Saleh, Saleh M., Naguib, Mary, El-Ezawy, Hosam El-Din M., Bakry, Lamiaa. Urine Neutrophil Gelatinase-Associated Lipocalin a Possible Diagnostic Marker for Egyptian Hepatocellular Carcinoma Patients. Asian Pacific journal of cancer prevention : APJCP 2020;21(8):2259-64. | Does not meet full eligibility criteria: includes people without cirrhosis |
| Abelev, G. I., Tsvetkov, V. S., Biriulina, T. I., El'gort, D. A. [Evaluation of the use of highly sensitive methods of determining alpha-fetoprotein for the diagnosis of hepatocellular cancer and teratoblastoma]. Otsenka primeneniia vysokochuvstvitel'nykh metodov opredeleniia al'fa-fetoproteina dlia diagnostiki gepatotselliuliarnogo raka i teratoblastom. 1971;71(4):75-81. | Does not meet full eligibility criteria: pre-2005 |
| Aboelfotoh, Aly O, Foda, Enas M, Elghandour, Ahmed M, Teama, Nahla M, Abouzein, Reham A. Talin-1; other than a potential marker for hepatocellular carcinoma diagnosis. Arab journal of gastroenterology : the official publication of the Pan-Arab Association of Gastroenterology 2020;21(2):80-84. | Does not meet full eligibility criteria: includes people without cirrhosis |
| Aboelfotoh, Aly O., Foda, Enas M., Elghandour, Ahmed M., Teama, Nahla M., Abouzein, Reham A. Talin-1; other than a potential marker for hepatocellular carcinoma diagnosis. Arab journal of gastroenterology : the official publication of the Pan-Arab Association of Gastroenterology 2020;21(2):80-84. | Duplicate (including Cochrane includes) |
| Abouzied, Mekky Mohammed, Eltahir, Heba M., Fawzy, Michael Atef, Abdel-Hamid, Nabil Mohie, Gerges, Amany Saber, El-Ibiari, Hesham Mohmoud. Estimation of leucine aminopeptidase and 5-nucleotidase increases alpha-fetoprotein sensitivity in human hepatocellular carcinoma cases. Asian Pacific journal of cancer prevention : APJCP 2015;16(3):959-63. | Exclude on population: healthy controls |
| Abu El Makarem, Mona A, Abdel-Aleem, Ashraf, Ali, Ahmed, Saber, Rafet, Shatat, Mohammed, Rahem, Doaa Abdel. Diagnostic significance of plasma osteopontin in hepatitis C virus-related hepatocellular carcinoma. Annals of hepatology 2011;10(3):296-305. | Does not meet full eligibility criteria: includes people without cirrhosis |
| Abu El Makarem, Mona A., Abdel-Aleem, Ashraf, Ali, Ahmed, Saber, Rafet, Shatat, Mohammed, Rahem, Doaa Abdel. Diagnostic significance of plasma | Duplicate (including Cochrane includes) |

| Reference | Reason for exclusion |
|---|---|
| osteopontin in hepatitis C virus-related hepatocellular carcinoma. Annals of hepatology 2011;10(3):296-305. | |
| Addley, H. C., Griffin, N., Shaw, A. S., Mannelli, L., Parker, R. A., Aitken, S., et al. Accuracy of hepatocellular carcinoma detection on multidetector CT in a transplant liver population with explant liver correlation. Clinical radiology 2011;66(4):349-56. | Exclude on population: clinical signs and symptoms of HCC |
| Ahmed Mohamed, Amal, El-Toukhy, Naglaa, Abdel-Hady Alkhalegy, Ayman, Boraii, Sherif. Osteopontin as A Tumor Marker for Hepatocellular Carcinoma: Mohamed AA et al . Osteopontin & Hepatocellular Carcinoma. Journal of Gastroenterology and Hepatology Research 2016;5(4):2140-0446. | Exclude on population: healthy controls |
| Ahmed, M. A. H. & Ellakwa, D. E. S. Role of serum osteopontin level as a diagnostic biomarker for early hepatocellular carcinoma. International Journal of Cancer Research 2014;10(1):37-45. | Exclude on population: not cirrhosis |
| Ahmed, N. N. A., El Gaafary, S. M., Elia, R. Z. Role of abbreviated MRI protocol for screening of HCC in HCV related cirrhotic patients prior to direct-acting antiviral treatment. Egyptian Journal of Radiology and Nuclear Medicine 2020;51(1):102. | Exclude on target condition: not HCC |
| Ahmed, R., Salama, H., Fouad, A., Sabry, D., AbdAlah, E. S. Detection of aberrant p16INK4A methylation in sera of patients with HCV-related liver diseases: An Egyptian study. Case Reports and Clinical Practice Review 2010;16(9):410-15. | Does not meet full eligibility criteria: includes people without cirrhosis |
| Ahn, D. G., Kim, H. J., Kang, H., Lee, H. W., Bae, S. H., Lee, J. H., Paik, Y. H. Feasibility of alpha-fetoprotein as a diagnostic tool for hepatocellular carcinoma in Korea. Korean Journal of Internal Medicine 2016;31(1):46-53. | Duplicate (including Cochrane includes) |
| Ahn, Dae Geon, Kim, Hyung Joon, Kang, Hyun, Lee, Hyun Woong, Bae, Si Hyun, Lee, Joon Hyoek, Paik, Yong Han. Feasibility of alpha-fetoprotein as a diagnostic tool for hepatocellular carcinoma in Korea. The Korean journal of internal medicine 2016;31(1):46-53. | Does not meet full eligibility criteria: includes people without cirrhosis |
| Ahn, Yeong Hee, Shin, Park Min, Oh, Na Ree, Park, Gun Wook, Kim, Hoguen. A lectin-coupled, targeted proteomic mass spectrometry (MRM MS) platform for identification of multiple liver cancer biomarkers in human plasma. Journal of proteomics 2012;75(17):5507-15. | Exclude on index test |
| Akai, Hiroyuki, Kiryu, Shigeru, Matsuda, Izuru, Satou, Jirou, Takao, Hidemasa, Tajima, Taku, et al. Detection of hepatocellular carcinoma by Gd-EOB-DTPA-enhanced liver MRI: comparison with triple phase 64 detector row helical CT. European journal of radiology 2011;80(2):310-15. | Exclude on population: clinical signs and symptoms of HCC |
| Akhtar, S., Hussain, M., Ali, S., Maqsood, S., Akram, S. Comparison of positive predictive value of multiphasic dynamic contrast enhanced MRI with dynamic contrast enhanced CT for the detection of hepatocellular Carcinoma. Pakistan Journal of Medical and Health Sciences 2020;14(3):562-64. | Exclude on population: clinical signs and symptoms of HCC |
| Akuta, N., Kawamura, Y., Arase, Y., Saitoh, S., Fujiyama, S., Sezaki, H., et al. TERT Promoter Mutation in Serum Cell-Free DNA Is a Diagnostic Marker of Primary Hepatocellular Carcinoma in Patients with Nonalcoholic Fatty Liver Disease. Oncology (Switzerland) 2021;99(2):114-23. | Exclude on population: not cirrhosis |
| Al Haddad, M., El-Mezayen, H. A., El-Kassas, M., Metwally, F. Clinical Utility of Cytokeratins for Accurate Diagnosis of Hepatocellular Carcinoma Among Hepatitis C Virus High-Risk Patients. Asian Pacific journal of cancer prevention : APJCP 2024;25(4):1325 “ 1332. | Continuous test without threshold |
| Aleem, Eiman, Elshayeb, Ayman, Elhabachi, Nihal, Mansour, Amal Refaat, Gowily, Ahmed. Serum IGFBP-3 is a more effective predictor than IGF-1 and IGF-2 for the development of hepatocellular carcinoma in patients with chronic HCV infection. Oncology letters 2012;3(3):704-12. | Exclude on index test |
| Alexander, M G, Purves, L R, Kirsch, R E, Bass, N M, Gitlin, N, Terblanche, J. alpha-Fetoprotein in liver disease. South African medical journal = Suid-Afrikaanse tydskrif vir geneeskunde 1978;53(12):433-6. | Does not meet full eligibility criteria: includes people without cirrhosis |
| Ali, Lamia H, Higazi, Aliaa M, Moness, Hend M, Farag, Naglaa M, Saad, Zienab M, Moukareb, Hamdy A, et al. Clinical significances and diagnostic utilities of both miR-215 and squamous cell carcinoma antigen-IgM versus alpha-fetoprotein in Egyptian patients with hepatitis C virus-induced hepatocellular | Does not meet full eligibility criteria: includes people without cirrhosis |

| Reference | Reason for exclusion |
|---|---|
| carcinoma. Clinical and experimental gastroenterology 2019;12(101532800):51-66. | |
| Ali, Lamia H., Higazi, Aliaa M., Moness, Hend M., Farag, Naglaa M., Saad, Zienab M., Moukareb, Hamdy A., et al. Clinical significances and diagnostic utilities of both miR-215 and squamous cell carcinoma antigen-IgM versus alpha-fetoprotein in Egyptian patients with hepatitis C virus-induced hepatocellular carcinoma. Clinical and experimental gastroenterology 2019;12():51-66. | Duplicate (including Cochrane includes) |
| Ali, M. A., Matboli, M., El-Khazragy, N., Saber, O., El-Nakeep, S., Abdelzaher, H. M., Shafei, A. E. S. Investigating miRNA-661 and ATG4-B mRNA expression as potential biomarkers for hepatocellular carcinoma. Biomarkers in Medicine 2018;12(3):345-56. | Does not meet full eligibility criteria: includes people without cirrhosis |
| Ali, Omaima Mohamed, El Amin, Hussein Ahmed, Sharkawy, Yousry Lotfy, Mohamed Ali, Adnan Ahmed, Kholef, Emad Farah Mohammed. Golgi Protein 73 versus Alpha-Fetoprotein as a New Biomarker in Early Diagnosis of Hepatocellular Carcinoma. International journal of general medicine 2020;13():193-200. | Exclude on population: not cirrhosis |
| Allam, A., Gabr, S., Ajarem, J. Bcl-2 and p53 expression in hepatic tissues of Egyptian patients with Chronic Hepatitis C. Journal of the Pakistan Medical Association 2015;65(11):1186-92. | Exclude on study design: not a 1- or 2-gate test accuracy study |
| Allam, Maha M., Abd El Moneim, Elhamy, Hassouna, Mouna M., El Jaky, Ashraf. Levels of chemokine receptors expressed on peripheral blood T lymphocytes of Egyptian patients with hepatocellular carcinoma. The Egyptian journal of immunology 2009;16(1):17-25. | Exclude on population: not cirrhosis |
| Almani, SA, Memon, AS, Ghori, RA, Memon, J. Alpha-fetoprotein as a diagnostic tool in differentiating hepatocellular carcinoma from benign hepatic disorders. Journal of Liaquat University of Medical and Health Sciences 2004;3(1):7-12. | Exclude on population: not cirrhosis |
| Alpert, E, Hershberg, R, Schur, P H. -fetoprotein in human hepatoma: improved detection in serum, and quantitative studies using a new sensitive technique. Gastroenterology 1971;61(2):137-43. | Exclude on population: not cirrhosis |
| Alsebaey, Ayman. Talin-1 and Non-invasive Fibrosis Models in the Assessment of Patients with Hepatocellular Carcinoma. Asian Pacific journal of cancer prevention : APJCP 2016;17(8):4077-82. | Does not meet full eligibility criteria: includes people without cirrhosis |
| Alsebaey, Ayman. Talin-1 and Non-invasive Fibrosis Models in the Assessment of Patients with Hepatocellular Carcinoma. Asian Pacific journal of cancer prevention : APJCP 2016;17(8):4077-82. | Does not meet full eligibility criteria: includes people without cirrhosis |
| Alunni-Fabbroni, Marianna, Weber, Sabine, Ocal, Osman, Seidensticker, Max, Mayerle, Julia, Malfertheiner, Peter. Circulating Cell-Free DNA Combined to Magnetic Resonance Imaging for Early Detection of HCC in Patients with Liver Cirrhosis. Cancers 2021;13(3):n. pag.. | Genomic biomarker not validated |
| Alzamzamy, Ahmed, Elsayed, Huda, Abd Elraouf, Mona, Eltoukhy, Hanan, Megahed, Tarek. Serum vascular endothelial growth factor as a tumor marker for hepatocellular carcinoma in hepatitis C virus-related cirrhotic patients. World journal of gastrointestinal oncology 2021;13(6):600-11. | Exclude on population: not cirrhosis |
| Al-Zoubi, Sara & Wassouf, Ahmad. Measuring levels of osteopontin as potential biomarker for hepatocellular carcinoma in Syrian patients. Gastroenterology and hepatology from bed to bench 2017;10(2):97-101. | Does not meet full eligibility criteria: includes people without cirrhosis |
| Al-Zoubi, Sara & Wassouf, Ahmad. Measuring levels of osteopontin as potential biomarker for hepatocellular carcinoma in Syrian patients. Gastroenterology and hepatology from bed to bench 2017;10(2):97-101. | Does not meet full eligibility criteria: includes people without cirrhosis |
| Amhimmid Badr, Sana, Waheeb Fahmi, Maryan, Mahmoud Nomir, Manal. Calcium channel alpha2delta1 subunit as a novel biomarker for diagnosis of hepatocellular carcinoma. Cancer biology & medicine 2018;15(1):52-60. | Exclude on population: healthy controls |
| Amr, Khalda S., Elmawgoud Atia, Hanan Abd, Elazeem Elbnhawy, Rehab Abd. Early diagnostic evaluation of miR-122 and miR-224 as biomarkers for hepatocellular carcinoma. Genes & diseases 2017;4(4):215-21. | Exclude on population: not cirrhosis |

| Reference | Reason for exclusion |
|---|---|
| Amuro, Y, Nakaoka, H, Shimomura, S, Fujikura, M, Yamamoto, T, Tamura, S, Hada, T. Serum pseudouridine as a biochemical marker in patients with hepatocellular carcinoma. Clinica chimica acta; international journal of clinical chemistry 1988;178(2):151-8. | Does not meet full eligibility criteria: includes people without cirrhosis |
| Amuro, Y., Nakaoka, H., Shimomura, S., Fujikura, M., Yamamoto, T., Tamura, S., Hada, T. Serum pseudouridine as a biochemical marker in patients with hepatocellular carcinoma. Clinica chimica acta; international journal of clinical chemistry 1988;178(2):151-58. | Does not meet full eligibility criteria: includes people without cirrhosis |
| An, Chansik, Kim, Do Young, Choi, Jin-Young, Han, Kwang Hyub, Roh, Yun Ho. Noncontrast magnetic resonance imaging versus ultrasonography for hepatocellular carcinoma surveillance (MIRACLE-HCC): study protocol for a prospective randomized trial. BMC cancer 2018;18(1):915. | Exclude on study design: not a 1- or 2-gate test accuracy study |
| Andreana, Lorenzo, Isgro, Graziella, Pleguezuelo, Maria, Germani, Giacomo. Surveillance and diagnosis of hepatocellular carcinoma in patients with cirrhosis. World journal of hepatology 2009;1(1):48-61. | Exclude on study design: not a 1- or 2-gate test accuracy study |
| Ang, Irene L., Poon, Terence C. W., Lai, Paul B. S., Chan, Anthony T. C., Ngai, Sai-Ming, Hui, Alex Y., Johnson, Philip J. Study of serum haptoglobin and its glycoforms in the diagnosis of hepatocellular carcinoma: a glycoproteomic approach. Journal of proteome research 2006;5(10):2691-7000. | Exclude on population: not cirrhosis |
| Anonymous. Poster Liver. Journal of gastroenterology and hepatology 2015;30 Suppl 4(a6j, 8607909):323-407. | Exclude on study design: not a 1- or 2-gate test accuracy study |
| Anzidei, M., Di Martino, M., Sacconi, B., Saba, L., Boni, F., Zaccagna, F., et al. Evaluation of image quality, radiation dose and diagnostic performance of dual-energy CT datasets in patients with hepatocellular carcinoma. Clinical radiology 2015;70(9):966-73. | Exclude on population: clinical signs and symptoms of HCC |
| Ao, Junjie, Chiba, Tetsuhiro, Kanzaki, Hiroaki, Kanayama, Kengo, Shibata, Shuhei, Kurosugi, Akane, et al. Serum Angiopoietin 2 acts as a diagnostic and prognostic biomarker in hepatocellular carcinoma. Journal of Cancer 2021;12(9):2694-7001. | Exclude on population: not cirrhosis |
| Aoyagi, Y., Suzuki, Y., Igarashi, K., Saitoh, A., Oguro, M., Yokota, T., et al. The usefulness of simultaneous determinations of glucosaminylation and fucosylation indices of alpha-fetoprotein in the differential diagnosis of neoplastic diseases of the liver. Cancer 1991;67(9):2390-94. | Exclude on population: not cirrhosis |
| Aoyagi, Y., Suzuki, Y., Isemura, M., Nomoto, M., Sekine, C., Igarashi, K. The fucosylation index of alpha-fetoprotein and its usefulness in the early diagnosis of hepatocellular carcinoma. Cancer 1988;61(4):769-74. | Does not meet full eligibility criteria: includes people without cirrhosis |
| Arita, Junichi, Takahashi, Michiro, Hata, Shojiro, Shindoh, Junichi, Beck, Yoshifumi, Sugawara, Yasuhiko, Hasegawa, Kiyoshi. Usefulness of contrast-enhanced intraoperative ultrasound using Sonazoid in patients with hepatocellular carcinoma. Annals of surgery 2011;254(6):992-99. | Exclude on study design: not a 1- or 2-gate test accuracy study |
| Arrieta, O., Cacho, B., Morales-Espinosa, D., Ruelas-Villavicencio, A., Flores-Estrada, D. The progressive elevation of alpha fetoprotein for the diagnosis of hepatocellular carcinoma in patients with liver cirrhosis. BMC Cancer 2007;7():28. | Duplicate (including Cochrane includes) |
| Arrigoni, A, Andriulli, A, Gindro, T, Piantino, P, Capussotti, L. Pattern analysis of serum alpha-fetoprotein in the early diagnosis of hepatocellular carcinoma in liver cirrhosis. The International journal of biological markers 1988;3(3):172-6. | Does not meet full eligibility criteria: pre-2005 |
| Arrigoni, A., Andriulli, A., Gindro, T., Piantino, P., Capussotti, L. Pattern analysis of serum alpha-fetoprotein in the early diagnosis of hepatocellular carcinoma in liver cirrhosis. The International journal of biological markers 1988;3(3):172-76. | Duplicate (including Cochrane includes) |
| Asazawa, Hitomi, Kamada, Yoshihiro, Takeda, Yuri, Takamatsu, Shinji, Shinzaki, Shinichiro, Kim, Youkoku, et al. Serum fucosylated haptoglobin in chronic liver diseases as a potential biomarker of hepatocellular carcinoma development. Clinical chemistry and laboratory medicine 2015;53(1):95-102. | Exclude on population: HCC participants not treatment-naive |
| Asghar, Sidra, Waqar, Walifa, Umar, Muhammad. Tumor educated platelets, a promising source for early detection of hepatocellular carcinoma: Liquid biopsy an alternative approach to tissue biopsy. Clinics and research in hepatology and gastroenterology 2020;44(6):836-44. | Exclude on population: not cirrhosis |

| Reference | Reason for exclusion |
|---|---|
| Astrom, Eva, Stal, Per, Zenlander, Robin, Edenvik, Pia, Alexandersson, Catharina, Haglund, Mats, Ryden, Ingvar. Reverse lectin ELISA for detecting fucosylated forms of alpha1-acid glycoprotein associated with hepatocellular carcinoma. PloS one 2017;12(3):e0173897. | No 2x2 data |
| Atiq, Omair, Tiro, Jasmin, Yopp, Adam C., Muffler, Adam, Marrero, Jorge A., Parikh, Neehar D., et al. An assessment of benefits and harms of hepatocellular carcinoma surveillance in patients with cirrhosis. Hepatology (Baltimore, Md.) 2017;65(4):1196-2005. | Duplicate (including Cochrane includes) |
| Atta, Mohamed Magdi El-Sadek Ali, Atta, Hazem Mahmoud, Gad, Magdy Abdel-Mawgoud, Rashed, Laila Ahmad, Said, Ebada M., Hassanien, Sharaf El-Sayed Ali. Clinical significance of vascular endothelial growth factor in hepatitis C related hepatocellular carcinoma in Egyptian patients. Journal of hepatocellular carcinoma 2016;3():19-24. | Exclude on population: not cirrhosis |
| Attallah, A M, Albannan, M S, El-Deen, M S, Farid, K, Khedr, F M, Attallah, K A. Diagnostic role of collagen-III and matrix metalloproteinase-1 for early detection of hepatocellular carcinoma. British journal of biomedical science 2020;77(2):58-63. | Does not meet full eligibility criteria: includes people without cirrhosis |
| Attallah, A. M., Albannan, M. S., El-Deen, M. S., Farid, K., Khedr, F. M., Attallah, K. A. Diagnostic role of collagen-III and matrix metalloproteinase-1 for early detection of hepatocellular carcinoma. British journal of biomedical science 2020;77(2):58-63. | Duplicate (including Cochrane includes) |
| Attallah, A. M., El-Far, M., Abdel Malak, C. A., Zahran, F., Farid, K., Omran, M. M., Zagloul, H. Evaluation of cytokeratin-1 in the diagnosis of hepatocellular carcinoma. Clinica Chimica Acta 2011;412(23-24):2310-15. | Duplicate (including Cochrane includes) |
| Attallah, A. M., El-Far, M., Abdelrazek, M. A., Omran, M. M., Attallah, A. A., Elkhouly, A. A., Elkenawy, H. M. Combined use of nuclear phosphoprotein c-Myc and cellular phosphoprotein p53 for hepatocellular carcinoma detection in high-risk chronic hepatitis C patients. British Journal of Biomedical Science 2017;74(4):170-75. | Exclude on population: not cirrhosis |
| Attallah, A. M., Omran, M. M., Attallah, A. A., Abdallah, S. O., Farid, K., Darwish, H., El-Dosoky, I. HCC-ART score, a simple, highly sensitive and specific test for early diagnosis of hepatocellular carcinoma: a large-scale, multicentre study. British journal of cancer 2013;109(6):1657-65. | Duplicate (including Cochrane includes) |
| Attallah, Abdelfattah M, El-Far, Mohamed, Abdel Malak, Camelia A, Zahran, Faten, Farid, Khaled, Omran, Mohamed M, Zagloul, Hayat. Evaluation of cytokeratin-1 in the diagnosis of hepatocellular carcinoma. Clinica chimica acta; international journal of clinical chemistry 2011;412(23-24):2310-5. | Does not meet full eligibility criteria: includes people without cirrhosis |
| Attallah, Abdelfattah M, Omran, Mohamed M, Attallah, Ahmed A, Abdelrazek, Mohamed A, Farid, Khaled. Simplified HCC-ART score for highly sensitive detection of small-sized and early-stage hepatocellular carcinoma in the widely used Okuda, CLIP, and BCLC staging systems. International journal of clinical oncology 2017;22(2):332-39. | Does not meet full eligibility criteria: includes people without cirrhosis |
| Attallah, Abdelfattah M., El-Far, Mohamed Abd El-Hafez, Omran, Mohamed Mostafa, Saeed, Aya Mohamed, Elbendary, Mohamed Sayed, Attallah, Kareem Abdelfattah. Comparison between glypican-3 and alpha-fetoprotein in discrimination of hepatocellular carcinoma from cirrhotic patients. Journal of Bioscience and Applied Research 2018;4(4):459-68. | Does not meet full eligibility criteria: includes people without cirrhosis |
| Attallah, Abdelfattah M., El-Far, Mohamed, Abdel Malak, Camelia A., Farid, Khaled, Omran, Mohamed M., Yahya, Raida S., et al. A simple diagnostic index comprising epithelial membrane antigen and fibronectin for hepatocellular carcinoma. Annals of hepatology 2015;14(6):869-80. | Exclude on index test |
| Attallah, Abdelfattah M., El-Far, Mohamed, Malak, Camelia A. Abdel, Omran, Mohamed M., Shiha, Gamal E., Farid, Khaled, et al. HCC-DETECT: a combination of nuclear, cytoplasmic, and oncofetal proteins as biomarkers for hepatocellular carcinoma. Tumour biology : the journal of the International Society for Oncodevelopmental Biology and Medicine 2015;36(10):7667-74. | Does not meet full eligibility criteria: includes people without cirrhosis |
| Attallah, Abdelfattah M., El-Far, Mohamed, Omran, Mohamed M., Abdelrazek, Mohamed A., Attallah, Ahmed A., Saeed, Aya M. GPC-HCC model: a combination of glybican-3 with other routine parameters improves the diagnostic efficacy in hepatocellular carcinoma. Tumour biology : the journal of | Does not meet full eligibility criteria: includes people without cirrhosis |

| Reference | Reason for exclusion |
|---|---|
| the International Society for Oncodevelopmental Biology and Medicine 2016;37(9):12571-77. | |
| Attallah, Abdelfattah M., Omran, Mohamed M., Attallah, Ahmed A., Abdelrazek, Mohamed A., Farid, Khaled. Simplified HCC-ART score for highly sensitive detection of small-sized and early-stage hepatocellular carcinoma in the widely used Okuda, CLIP, and BCLC staging systems. International journal of clinical oncology 2017;22(2):332-39. | Duplicate (including Cochrane includes) |
| Attallah, K. A. & Farid, K. Revolutionizing early HCC detection: groundbreaking validation of the HCC-check index for small tumors in Egyptian patients using CLIP and BCLC staging systems. Medical Oncology 2025;42(7):274. | Does not meet full eligibility criteria: includes people without cirrhosis |
| Attallah, K. A., Albannan, M. S., Farid, K., Rizk, S. M. HCC-Check: A Novel Diagnostic Tool for Early Detection of Hepatocellular Carcinoma Based on Cytokeratin-1 and Epithelial Membrane Antigen: A Cross-Sectional Study. Technology in Cancer Research and Treatment 2024;23():n. pag.. | Does not meet full eligibility criteria: includes people without cirrhosis |
| Attia, M. S., Youssef, A. O., Khan, Ziya A. Alpha fetoprotein assessment by using a nano optical sensor thin film binuclear Pt-2-aminobenzimidazole-Bipyridine for early diagnosis of liver cancer. Talanta 2018;186():36-43. | Exclude on population: not cirrhosis |
| Aube, Christophe, Oberti, Frederic, Lonjon, Julie, Pageaux, Georges, Seror, Olivier, N'Kontchou, Gisele, et al. EASL and AASLD recommendations for the diagnosis of HCC to the test of daily practice. Liver international : official journal of the International Association for the Study of the Liver 2017;37(10):1515-25. | Exclude on population: clinical signs and symptoms of HCC |
| Augustin, Salvador. Diagnostic and prognostic markers in liver cirrhosis. Disease markers 2011;31(3):119-20. | Exclude on study design: not a 1- or 2-gate test accuracy study |
| Awad, A. E., Ebrahim, M. A., Eissa, L. A. Dickkopf-1 and amphiregulin as novel biomarkers and potential therapeutic targets in hepatocellular carcinoma. International Journal of Hematology-Oncology and Stem Cell Research 2019;13(3):153-63. | Exclude on index test |
| Aydin, Yucel, Koksal, Ali Riza, Thevenot, Paul, Chava, Srinivas, Heidari, Zahra, Lin, Dong, et al. Experimental Validation of Novel Glypican 3 Exosomes for the Detection of Hepatocellular Carcinoma in Liver Cirrhosis. Journal of hepatocellular carcinoma 2021;8():1579-96. | No 2x2 data |
| Bachtiar, Indra, Kheng, Valentine, Wibowo, Gunawan A., Gani, Rino A., Hasan, Irsan, Sanityoso, Andri, et al. Alpha-1-acid glycoprotein as potential biomarker for alpha-fetoprotein-low hepatocellular carcinoma. BMC research notes 2010;3():319. | Exclude on population: not cirrhosis |
| Bachtiar, Indra, Santoso, Julian Mulya, Atmanegara, Benny, Gani, Rino Alvani, Hasan, Irsan, Lesmana, Laurentius Adrianto, et al. Combination of alpha-1-acid glycoprotein and alpha-fetoprotein as an improved diagnostic tool for hepatocellular carcinoma. Clinica chimica acta; international journal of clinical chemistry 2009;399(1-2):97-101. | Exclude on population: not cirrhosis |
| Bachtiar, Indra, Santoso, Julian Mulya, Atmanegara, Benny, Gani, Rino Alvani, Hasan, Irsan, Lesmana, Laurentius Adrianto, et al. Combination of alpha-1-acid glycoprotein and alpha-fetoprotein as an improved diagnostic tool for hepatocellular carcinoma. Clinica chimica acta; international journal of clinical chemistry 2009;399(1-2):97-101. | Duplicate (including Cochrane includes) |
| Badr E.A.E., Korah T.E., Ghani A.A., El-Sayed S. Role of serum glypican-3 in the diagnosis and differentiation of small hepatocellular carcinoma from hepatitis-C virus cirrhosis. Alexandria Journal of Medicine 2014;50(3):221-26. | Does not meet full eligibility criteria: includes people without cirrhosis |
| Badr, E. A. E., Korah, T. E., Ghani, A. A., El-Sayed, S. Role of serum glypican-3 in the diagnosis and differentiation of small hepatocellular carcinoma from hepatitis-C virus cirrhosis. Alexandria Journal of Medicine 2014;50(3):221-26. | Duplicate (including Cochrane includes) |
| Badr, S. A., Fahmi, M. W., Nomir, M. M. Calcium channel alpha2delta1 subunit as a novel biomarker for diagnosis of hepatocellular carcinoma. Cancer Biology and Medicine 2018;15(1):52-60. | Exclude on population: healthy controls |
| Bae, J. S., Lee, D. H., Lee, S. M., Suh, K. S., Lee, K. W., Yi, N. J., et al. Performance of LI-RADS Version 2018 on CT for Determining Eligibility for Liver Transplant According to Milan Criteria in Patients at High Risk for Hepatocellular Carcinoma. American Journal of Roentgenology 2022;219(1):86-96. | Does not meet full eligibility criteria: includes people without cirrhosis |

| Reference | Reason for exclusion |
|---|---|
| Bae, J. S., Lee, H. H., Kim, H., Song, I. C., Lee, J. Y. Deep Learning-aided 1H-MR Spectroscopy for Differentiating between Patients with and without Hepatocellular Carcinoma. Magnetic resonance in medical sciences : MRMS : an official journal of Japan Society of Magnetic Resonance in Medicine 2025;():n. pag.. | Genomic biomarker not validated |
| Bae, So Young, Choi, Moon Seok, Gwak, Geum-Youn, Paik, Yong Han, Lee, Joon Hyoek, Koh, Kwang Cheol, Paik, Seung Woon. Comparison of usefulness of clinical diagnostic criteria for hepatocellular carcinoma in a hepatitis B endemic area. Clinical and molecular hepatology 2012;18(2):185-94. | Exclude on index test |
| Baek, C. K., Choi, J. Y., Kim, K. A., Park, M. S., Lim, J. S., Chung, Y. E., Kim, M. J. Hepatocellular carcinoma in patients with chronic liver disease: A comparison of gadoxetic acid-enhanced MRI and multiphasic MDCT. Clinical Radiology ;():n. pag.. | Exclude on population: clinical signs and symptoms of HCC |
| Baek, K. A., Kim, S. S., Shin, H. C., Hwang, J. A., Choi, S. Y., Lee, W. H., et al. Gadoxetic acid-enhanced MRI for diagnosis of hepatocellular carcinoma in patients with chronic liver disease: can hypointensity on the late portal venous phase be used as an alternative to washout? Abdominal Radiology 2020;45(9):2705-16. | Exclude on population: clinical signs and symptoms of HCC |
| Bai, X. F. & Wang, P. Y. Value of combined determination of tumor markers based on two discriminative models in facilitating diagnosis of hepatic carcinoma. Medical Journal of Chinese People's Liberation Army 2012;37(11):849-52. | Foreign language |
| Bai, X., Jia, J. A., Fang, M., Chen, S., Liang, X., Zhu, S., et al. Deep sequencing of HBV pre-S region reveals high heterogeneity of HBV genotypes and associations of word pattern frequencies with HCC. PLoS Genetics 2018;14(2):e1007206. | Exclude on population: not cirrhosis |
| Bai, Yu, Shen, Yunzhi, Yuan, Qiang, Lv, Chengyu. Evaluation of Relationship between Occurrence of Liver Cancer and Methylation of Fragile Histidine Triad (FHIT) and P16 Genes. Medical science monitor : international medical journal of experimental and clinical research 2019;25():1301-06. | Exclude on study design: not a 1- or 2-gate test accuracy study |
| Baig, Jawed Altaf, Alam, Junaid Mahmood, Mahmood, Syed Riaz, Baig, Mohammad, Shaheen, Rabia, Sultana, Ishrat. Hepatocellular carcinoma (HCC) and diagnostic significance of A-fetoprotein (AFP). Journal of Ayub Medical College, Abbottabad : JAMC 2009;21(1):72-75. | Exclude on population: not cirrhosis |
| Bakr, N. M. & Awad, A. Association of genetic variants in the interleukin-18 gene promoter with risk of hepatocellular carcinoma and metastasis in patients with hepatitis C virus infection. IUBMB Life 2018;70(2):165-74. | Exclude on population: not cirrhosis |
| Balkan, A. & Gulsen, M. T. Serum MicroRNA-26, MicroRNA-122 and MicroRNA-192 expressions in hepatocellular carcinoma. Acta Medica Mediterranea 2017;33(1):165-72. | Exclude on population: not cirrhosis |
| Baniasadi, Hamid, Gowda, G. A. Nagana, Gu, Haiwei, Zeng, Ao, Zhuang, Shui, Skill, Nicholas, Maluccio, Mary. Targeted metabolic profiling of hepatocellular carcinoma and hepatitis C using LC-MS/MS. Electrophoresis 2013;34(19):2910-17. | Exclude on index test |
| Bannaga, A., Metzger, J., Voigtlander, T., Pejchinovski, M., Frantzi, M., Book, T., et al. Pathophysiological implications of urinary peptides in hepatocellular carcinoma. Cancers 2021;13(15):3786. | Exclude on index test |
| Bannaga, Ayman S., Alvarez, Rocio, Zhou, Lisa, Petchey, Michael, Noufaily, Angela, Hitchins, Megan P. Role of methylated septin 9 as an adjunct diagnostic and prognostic biomarker in hepatocellular carcinoma. HPB : the official journal of the International Hepato Pancreato Biliary Association 2021;23(10):1595-6006. | Exclude on population: not cirrhosis |
| Barletta, Emiddio & Tinessa, Vincenza. [Screening of hepatocellular carcinoma: role of the alpha-fetoprotein (AFP) and ultrasonography]. Lo screening del carcinoma epatocellulare. 2005;96(6):295-328. | Foreign language |
| Barsoum, Ihab & Elgohary, Marwan N. Lipocalin-2: A novel diagnostic marker for hepatocellular carcinoma. Cancer biomarkers : section A of Disease markers 2020;28(4):523-28. | Does not meet full eligibility criteria: includes people without cirrhosis |

| Reference | Reason for exclusion |
|---|---|
| Bartolozzi, C., Donati, F., Cioni, D., Crocetti, L. MnDPDP-enhanced MRI vs dual-phase spiral CT in the detection of hepatocellular carcinoma in cirrhosis. European radiology 2000;10(11):1697-7002. | Exclude on population: clinical signs and symptoms of HCC |
| Bartolozzi, Carlo, Battaglia, Valentina, Bargellini, Irene, Bozzi, Elena, Campani, Daniela, Pollina, Luca Emanuele. Contrast-enhanced magnetic resonance imaging of 102 nodules in cirrhosis: correlation with histological findings on explanted livers. Abdominal imaging 2013;38(2):290-96. | Exclude on population: clinical signs and symptoms of HCC |
| Bashir, Aya O., El-Mesery, Mohamed E., Anwer, Rokiah. Thymoquinone potentiates miR-16 and miR-375 expressions in hepatocellular carcinoma. Life sciences 2020;254():117794. | Exclude on population: healthy controls |
| Basile, U., Miele, L., Napodano, C., Ciasca, G., Gulli, F., Pocino, K., et al. The diagnostic performance of PIVKA-II in metabolic and viral hepatocellular carcinoma: A pilot study. European Review for Medical and Pharmacological Sciences 2021;24(24):12675-85. | Exclude on population: healthy controls |
| Batbaatar, B., Gurbadam, U., Tuvshinsaikhan, O., Narmandakh, N. E., Khatanbaatar, G., Radnaabazar, M., et al. Evaluation of glypican-3 in patients with hepatocellular carcinoma. Molecular and Clinical Oncology 2025;22(1):1. | Exclude on index test |
| Bayjanov, A. K., Ismoilov, U. Yu, Khikmatullaeva, A. S., Nasirova Kh, P., Anvarov, J. A. The use of biomarkers GP-73 and AFP in the early diagnosis of hepatocarcinoma associated with liver cirrhosis of HCV etiology. Jurnal Infektologii 2025;17(2):89 “ 95. | Foreign language |
| Bedair, H. M., El-Banna, E. A., Ahmed, E. A., Elhelbawy, M. G., Abdelfattah, A., Khalaf, F. A., Abdel-Samiee, M. Evaluation of Circular RNA SMARCA5 as a Novel Biomarker for Hepatocellular Carcinoma. Asian Pacific journal of cancer prevention : APJCP 2024;25(4):1411 “ 1417. | Does not meet full eligibility criteria: includes people without cirrhosis |
| Bell, H. Alpha-fetoprotein and carcinoembryonic antigen in patients with primary liver carcinoma, metastatic liver disease, and alcoholic liver disease. Scandinavian journal of gastroenterology 1982;17(7):897-903. | Exclude on population: not cirrhosis |
| Ben Hassine, L., Daghfous, M. H., Mami, A., Chammakhi-Jemli, C., Zouaoui, W., Saddoud, W., et al. [Imaging in the screening and diagnosis of hepatocellular carcinoma in cirrhosis liver in Tunisia. A series of 30 cases]. L'imagerie dans le depistage et le diagnostic du carcinome hepatocellulaire sur foie de cirrhose en Tunisie. A propos de 30 cas. 2007;85(5):421-26. | Foreign language |
| Ben, Q., Ni, R., Xiao, M., Lu, C., Li, L. The value of HS-AFP in the early diagnosis and differential diagnosis of hepatocellular carcinoma. Chinese Journal of Clinical Oncology 2008;35(15):845-47. | Foreign language |
| Beneduce, L, Castaldi, F, Marino, M, Tono, N, Gatta, A, Pontisso, P. Improvement of liver cancer detection with simultaneous assessment of circulating levels of free alpha-fetoprotein (AFP) and AFP-IgM complexes. The International journal of biological markers 2004;19(2):155-9. | Does not meet full eligibility criteria: pre-2005 |
| Beneduce, L, Pesce, G, Gallotta, A, Zampieri, F, Biasiolo, A, Tono, N, et al. Tumour-specific induction of immune complexes: DCP-IgM in hepatocellular carcinoma. European journal of clinical investigation 2008;38(8):571-7. | Does not meet full eligibility criteria: includes people without cirrhosis |
| Beneduce, L., Castaldi, F., Marino, M., Tono, N., Gatta, A., Pontisso, P. Improvement of liver cancer detection with simultaneous assessment of circulating levels of free alpha-fetoprotein (AFP) and AFP-IgM complexes. The International journal of biological markers 2004;19(2):155-59. | Does not meet full eligibility criteria: includes people without cirrhosis |
| Beneduce, L., Pesce, G., Gallotta, A., Zampieri, F., Biasiolo, A., Tono, N., et al. Tumour-specific induction of immune complexes: DCP-IgM in hepatocellular carcinoma. European journal of clinical investigation 2008;38(8):571-77. | Duplicate (including Cochrane includes) |
| Beneduce, Luca, Castaldi, Francesco, Marino, Maria, Quarta, Santina, Ruvoletto, Mariagrazia, Benvegnu, Luisa, et al. Squamous cell carcinoma antigen-immunoglobulin M complexes as novel biomarkers for hepatocellular carcinoma. Cancer 2005;103(12):2558-65. | Does not meet full eligibility criteria: includes people without cirrhosis |
| Benhammou, Jihane N., Rich, Nicole E., Cholankeril, George, Zhang, Peng, Zeng, Weihua, Rao, Shuyun, Tayob, Nabihah. DETECT: Development of Technologies for Early HCC Detection. Gastroenterology 2022;163(1):21-27. | Exclude on study design: not a 1- or 2-gate test accuracy study |
| Bennett, Genevieve L, Krinsky, Glenn A, Abitbol, Roxanne J, Kim, Sue Y, Theise, Neil D. Sonographic detection of hepatocellular carcinoma and | Does not meet full eligibility criteria: pre-2005 |

| Reference | Reason for exclusion |
|---|---|
| dysplastic nodules in cirrhosis: correlation of pretransplantation sonography and liver explant pathology in 200 patients. AJR. American journal of roentgenology 2002;179(1):75-80. | |
| Berhane, Sarah, Toyoda, Hidenori, Tada, Toshifumi, Kumada, Takashi, Kagebayashi, Chiaki, Satomura, Shinji, et al. Role of the GALAD and BALAD-2 Serologic Models in Diagnosis of Hepatocellular Carcinoma and Prediction of Survival in Patients. Clinical gastroenterology and hepatology : the official clinical practice journal of the American Gastroenterological Association 2016;14(6):875-86.ee6. | Exclude on population: not cirrhosis |
| Bertino, G., Neri, S., Bruno, C. M., Ardiri, A. M., Calvagno, G. S., Malaguarnera, M., et al. Diagnostic and prognostic value of alpha-fetoprotein, des-gamma-carboxy prothrombin and squamous cell carcinoma antigen immunoglobulin M complexes in hepatocellular carcinoma. Minerva medica 2011;102(5):363-71. | Exclude on study design: not a 1- or 2-gate test accuracy study |
| Bertino, Gaetano, Ardiri, Annalisa Maria, Calvagno, Giuseppe Stefano, Bertino, Nicoletta. Prognostic and diagnostic value of des-gamma-carboxy prothrombin in liver cancer. Drug news & perspectives 2010;23(8):498-508. | Exclude on study design: not a 1- or 2-gate test accuracy study |
| Bertino, Gaetano, Ardiri, Annalisa, Malaguarnera, Michele, Malaguarnera, Giulia, Bertino, Nicoletta. Hepatocellualar carcinoma serum markers. Seminars in oncology 2012;39(4):410-33. | Exclude on study design: not a 1- or 2-gate test accuracy study |
| Besa, Cecilia, Kakite, Suguru, Cooper, Nancy, Facciuto, Marcelo. Comparison of gadoxetic acid and gadopentetate dimeglumine-enhanced MRI for HCC detection: prospective crossover study at 3 T. Acta radiologica open 2015;4(2):2047981614561285. | Exclude on population: clinical signs and symptoms of HCC |
| Besa, Cecilia, Lewis, Sara, Pandharipande, Pari V., Chhatwal, Jagpreet, Kamath, Amita, Cooper, Nancy, et al. Hepatocellular carcinoma detection: diagnostic performance of a simulated abbreviated MRI protocol combining diffusion-weighted and T1-weighted imaging at the delayed phase post gadoxetic acid. Abdominal radiology (New York) 2017;42(1):179-90. | Does not meet full eligibility criteria: includes people without cirrhosis |
| Best, J, Bilgi, H, Heider, D, Schotten, C, Manka, P, Bedreli, S, et al. The GALAD scoring algorithm based on AFP, AFP-L3, and DCP significantly improves detection of BCLC early stage hepatocellular carcinoma. Der GALAD-Score, ein AFP-, AFP-L3- und DCP-basierter Diagnosealgorithmus verbessert die Detektionsrate des hepatozellularen Karzinoms im BCLC-Fruhstadium signifikant. 2016;54(12):1296-3005. | Exclude on population: not cirrhosis |
| Best, J., Bechmann, L. P., Sowa, J. P., Sydor, S., Dechene, A., Pflanz, K., et al. GALAD Score Detects Early Hepatocellular Carcinoma in an International Cohort of Patients With Nonalcoholic Steatohepatitis. Clinical Gastroenterology and Hepatology 2020;18(3):728-35.ee4. | Duplicate (including Cochrane includes) |
| Best, J., Bilgi, H., Heider, D., Schotten, C., Manka, P., Bedreli, S., et al. The GALAD scoring algorithm based on AFP, AFP-L3, and DCP significantly improves detection of BCLC early stage hepatocellular carcinoma. Der GALAD-Score, ein AFP-, AFP-L3- und DCP-basierter Diagnosealgorithmus verbessert die Detektionsrate des hepatozellularen Karzinoms im BCLC-Fruhstadium signifikant. 2016;54(12):1296-3005. | Foreign language |
| Best, Jan, Bechmann, Lars P, Sowa, Jan-Peter, Sydor, Svenja, Dechene, Alexander, Pflanz, Kristina, et al. GALAD Score Detects Early Hepatocellular Carcinoma in an International Cohort of Patients With Nonalcoholic Steatohepatitis. Clinical gastroenterology and hepatology : the official clinical practice journal of the American Gastroenterological Association 2020;18(3):728-35.ee4. | Does not meet full eligibility criteria: includes people without cirrhosis |
| Bharali, Dipu, Banerjee, Basu D., Bharadwaj, Mausumi, Husain, Syed A. Expression Analysis of MicroRNA-21 and MicroRNA-122 in Hepatocellular Carcinoma. Journal of clinical and experimental hepatology 2019;9(3):294-301. | Exclude on population: healthy controls |
| Bharali, Dipu, Banerjee, Basu Dev, Bharadwaj, Mausumi, Husain, Syed Akhtar. Expression analysis of apolipoproteins AI & AIV in hepatocellular carcinoma: A protein-based hepatocellular carcinoma-associated study. The Indian journal of medical research 2018;147(4):361-68. | Exclude on population: not cirrhosis |
| Bharali, Dipu, Jebur, Hakim B., Baishya, Debabrat, Kumar, Suresh, Sarma, Manash P., Masroor, Mirza, et al. Expression Analysis of Serum microRNA-34a | Exclude on population: not cirrhosis |

| Reference | Reason for exclusion |
|---|---|
| and microRNA-183 in Hepatocellular Carcinoma. Asian Pacific journal of cancer prevention : APJCP 2018;19(9):2561-68. | |
| Bhartia, Bobby, Ward, Janice, Guthrie, J. Ashley. Hepatocellular carcinoma in cirrhotic livers: double-contrast thin-section MR imaging with pathologic correlation of explanted tissue. AJR. American journal of roentgenology 2003;180(3):577-84. | Does not meet full eligibility criteria: pre-2005 |
| Bhat, M., Goldstein, S., Tchervenkov, J., Ali, S., Neville, A., Kalyanasundaram, S., et al. P53 inducible gene-3: Potential serum marker for hepatocellular carcinoma beyond Milan criteria. Journal of Gastroenterology and Hepatology Research 2015;4(1):1434-38. | Exclude on study design: not a 1- or 2-gate test accuracy study |
| Bhattacharya, Sourav, Steele, Robert, Shrivastava, Shubham, Chakraborty, Sounak, Di Bisceglie, Adrian M. Serum miR-30e and miR-223 as Novel Noninvasive Biomarkers for Hepatocellular Carcinoma. The American journal of pathology 2016;186(2):242-47. | Exclude on population: not cirrhosis |
| Bianco, Cristiana, Jamialahmadi, Oveis, Pelusi, Serena, Baselli, Guido, Dongiovanni, Paola, Zanoni, Irene, et al. Non-invasive stratification of hepatocellular carcinoma risk in non-alcoholic fatty liver using polygenic risk scores. Journal of hepatology 2021;74(4):775-82. | Exclude on index test |
| Bihrer, Verena, Waidmann, Oliver, Friedrich-Rust, Mireen, Forestier, Nicole, Susser, Simone, Haupenthal, Jorg, et al. Serum microRNA-21 as marker for necroinflammation in hepatitis C patients with and without hepatocellular carcinoma. PloS one 2011;6(10):e26971. | Exclude on population: not cirrhosis |
| Bischof, Danielle A. Surveillance for hepatocellular carcinoma with ultrasound and AFP is associated with improvements in tumour detection, receipt of curative therapy and overall survival in patients with cirrhosis. Evidence-based medicine 2014;19(6):225-26. | Exclude on study design: not a 1- or 2-gate test accuracy study |
| Biselli, M., Conti, F., Gramenzi, A., Frigerio, M., Cucchetti, A., Fatti, G., et al. A new approach to the use of alpha-fetoprotein as surveillance test for hepatocellular carcinoma in patients with cirrhosis. British Journal of Cancer 2015;112(1):69-76. | Duplicate (including Cochrane includes) |
| Bizollon, T., Rode, A., Bancel, B., Gueripel, V., Ducerf, C., Baulieux, J. Diagnostic value and tolerance of Lipiodol-computed tomography for the detection of small hepatocellular carcinoma: correlation with pathologic examination of explanted livers. Journal of hepatology 1998;28(3):491-96. | Exclude on population: clinical signs and symptoms of HCC |
| Blondin, D., Erhardt, A., Crynen, K., Sagir, A., Scherer, A., Kropil, P., et al. [Diagnosis of focal liver lesions in cirrhotic patients: comparison of contrast-enhanced ultrasound using sulphur hexafluoride (SF6) microbubbles and MRI using Gd-EOB-DTPA]. Vergleich der kontrastverstarkten Sonografie und der MRT mit Gd-EOB-DTPA zur Diagnostik fokaler Leberlasionen bei Patienten mit Leberzirrhose. 2011;49(1):23-29. | Foreign language |
| Bohling, N. Contrast enhanced ultrasound (CEUS) in the diagnosis of malignant liver lesions in patients with cirrhosis and transjugular intrahepatic portosystemic shunt (TIPS). Zeitschrift fur Gastroenterologie 2019;57(9):e286. | Exclude on population: clinical signs and symptoms of HCC |
| Bolondi, L, Sofia, S, Siringo, S, Gaiani, S, Casali, A, Zironi, G, et al. Surveillance programme of cirrhotic patients for early diagnosis and treatment of hepatocellular carcinoma: a cost effectiveness analysis. Gut 2001;48(2):251-9. | Does not meet full eligibility criteria: pre-2005 |
| Bon, C, Brillard, B, Gelineau, M C, Mailliavin, A, Trepo, C. [Decarboxyprothrombin: importance in the diagnosis of hepatocellular carcinoma]. La decarboxyprothrombine: interet dans le diagnostic du carcinome hepatocellulaire. 1998;56(2):175-81. | Does not meet full eligibility criteria: pre-2005 |
| Bon, C., Brillard, B., Gelineau, M. C., Mailliavin, A., Trepo, C. [Decarboxyprothrombin: importance in the diagnosis of hepatocellular carcinoma]. La decarboxyprothrombine: interet dans le diagnostic du carcinome hepatocellulaire. 1998;56(2):175-81. | Does not meet full eligibility criteria: pre-2005 |
| Bon, C., Brillard, B., Gelineau, M. C., Mailliavin, A., Trepo, C. Decarboxyprothrombin : Diagnostic value in hepatocellular carcinoma. Annales de Biologie Clinique 1998;56(2):175-81. | Does not meet full eligibility criteria: pre-2005 |
| Born, M., Layer, G., Kreft, B., Schwarz, N. [MRI, CT and CT arterial portography in the diagnosis of malignant liver tumors in liver cirrhosis]. MRT, | Does not meet full eligibility criteria: pre-2005 |

| Reference | Reason for exclusion |
|---|---|
| CT und CTAP in der Diagnostik maligner Lebertumoren bei Leberzirrhose. 1998;168(6):567-72. | |
| Born, M., Layer, G., Kreft, B., Schwarz, N. Diagnostics of malignant liver tumours by MRI, CT and CTAP in cirrhosis patients. RoFo Fortschritte auf dem Gebiete der Rontgenstrahlen und der Neuen Bildgebenden Verfahren 1998;168(6):567-72. | Does not meet full eligibility criteria: pre-2005 |
| Borscheri, N. & Roessner, A. Canalicular immunostaining of neprilysin (CD10) as a diagnostic marker for hepatocellular carcinomas. The American journal of surgical pathology 2001;25(10):1297-3003. | Exclude on population: tissue samples |
| Boryczka, Grzegorz & Hartleb, Marek. Detection of hepatocellular carcinoma by tissue resonance interaction method (TRIM). Przeglad gastroenterologiczny 2018;13(1):40-46. | Exclude on index test |
| Bota, S., Mandorfer, M., Ferlitsch, A., Sieghart, W. High alpha-fetoprotein levels and presence of clinically significant portal hypertension represent risk factors for hepatocellular carcinoma development. Wiener Klinische Wochenschrift 2014;126(17-18):568. | Exclude on study design: not a 1- or 2-gate test accuracy study |
| Bowers, Jeremiah, Hughes, Emma, Skill, Nicholas, Maluccio, Mary. Detection of hepatocellular carcinoma in hepatitis C patients: biomarker discovery by LC-MS. Journal of chromatography. B, Analytical technologies in the biomedical and life sciences 2014;966():154-62. | Exclude on index test |
| Brancatelli, Giuseppe, Baron, Richard L., Peterson, Mark S. Helical CT screening for hepatocellular carcinoma in patients with cirrhosis: frequency and causes of false-positive interpretation. AJR. American journal of roentgenology 2003;180(4):1007-14. | Does not meet full eligibility criteria: pre-2005 |
| Bruce, M. G., Bruden, D., McMahon, B. J., Christensen, C., Homan, C., Sullivan, D., et al. Clinical significance of elevated alpha-fetoprotein in Alaskan Native patients with chronic hepatitis C. Journal of viral hepatitis 2008;15(3):179-87. | Exclude on population: not cirrhosis |
| Brudermanns, B. Hepatocellular carcinoma - Double contrast MRI at 3.0 T shows high sensitivity. RoFo Fortschritte auf dem Gebiet der Rontgenstrahlen und der Bildgebenden Verfahren 2009;181(1):8. | Foreign language |
| Brunello, F, Marcarino, C, Pasquero, P, Gastaldi, P, Gonella, S, Martini, S, Calcamuggi, G. The des-gamma-carboxyprothrombin for the diagnosis of hepatocellular carcinoma. The Italian journal of gastroenterology 1993;25(1):9-12. | Does not meet full eligibility criteria: pre-2005 |
| Brunello, F., Marcarino, C., Pasquero, P., Gastaldi, P., Gonella, S., Martini, S., Calcamuggi, G. The des-gamma-carboxyprothrombin for the diagnosis of hepatocellular carcinoma. The Italian journal of gastroenterology 1993;25(1):9-12. | Duplicate (including Cochrane includes) |
| Bruno, S., Silini, E., Crosignani, A., Borzio, F., Leandro, G., Bono, F., et al. Hepatitis C virus genotypes and risk of hepatocellular carcinoma in cirrhosis: a prospective study. Hepatology (Baltimore, Md.) 1997;25(3):754-58. | Exclude on study design: not a 1- or 2-gate test accuracy study |
| Brunsing, Ryan L., Chen, Dennis H., Schlein, Alexandra, Wolfson, Tanya, Gamst, Anthony, Mamidipalli, Adrija, et al. Gadoxetate-enhanced Abbreviated MRI for Hepatocellular Carcinoma Surveillance: Preliminary Experience. Radiology. Imaging cancer 2019;1(2):e190010. | Does not meet full eligibility criteria: includes people without cirrhosis |
| Bu, H., Luo, W., Tao, W., Dong, C., Wang, M., Ye, X., et al. A Large-Scale Retrospective Study of Serum Des-Gamma-Carboxy Prothrombin as a Diagnostic Marker of HCC: Effect of Liver Function on Specificity. Journal of Clinical Laboratory Analysis 2025;39(10):e70025. | Does not meet full eligibility criteria: includes people without cirrhosis |
| Buffet, C, Prades, P, Hagege, H, Cauquil, P, Ink, O. [Is the focal echographic lesion of the liver in patients with cirrhosis always correlated with hepatocellular carcinoma? 217 cases]. La lesion echographique focalisee du foie chez le cirrhotique correspond-t-elle toujours a un carcinome hepatocellulaire? 217 observations. 1988;17(32):1629-32. | Does not meet full eligibility criteria: pre-2005 |
| Bui Huu, Hoang, Ha Thuc, Nhuong, Thi Le, Hoa Pham, Thi Thanh, Thuy Do, Luong Bac, An, Tiribelli, Claudio, et al. Characterization of SCCA-IgM as a biomarker of liver disease in an Asian cohort of patients. Scandinavian journal of clinical and laboratory investigation 2018;78(3):204-10. | No 2x2 data |

| Reference | Reason for exclusion |
|---|---|
| Burditt, L. J., Johnson, M. M., Johnson, P. J. Detection of hepatocellular carcinoma-specific alpha-fetoprotein by isoelectric focusing. Cancer 1994;74(1):25-29. | Secondary publication of included study |
| Burrel, M., Llovet, J. M., Ayuso, C., Iglesias, C., Sala, M., Miquel, R., et al. MRI angiography is superior to helical CT for detection of HCC prior to liver transplantation: An explant correlation. Hepatology 2003;38(4):1034-42. | Exclude on population: HCC participants not treatment-naive |
| Cabel, L., Proudhon, C., Buecher, B., Pierga, J. Y. Circulating tumor DNA detection in hepatocellular carcinoma. Annals of oncology : official journal of the European Society for Medical Oncology 2018;29(5):1094-96. | Exclude on study design: not a 1- or 2-gate test accuracy study |
| Cabiati, Manuela, Gaggini, Melania, Cesare, Maria Michela, Caselli, Chiara, De Simone, Paolo, Filipponi, Franco, et al. Osteopontin in hepatocellular carcinoma: A possible biomarker for diagnosis and follow-up. Cytokine 2017;99():59-65. | Exclude on study design: not a 1- or 2-gate test accuracy study |
| Cabrera, Roniel, Fitian, Asem I, Ararat, Miguel, Xu, Yiling, Brusko, Todd, Wasserfall, Clive, et al. Serum levels of soluble CD25 as a marker for hepatocellular carcinoma. Oncology letters 2012;4(4):840-46. | Does not meet full eligibility criteria: includes people without cirrhosis |
| Cabrera, Roniel, Fitian, Asem I., Ararat, Miguel, Xu, Yiling, Brusko, Todd, Wasserfall, Clive, et al. Serum levels of soluble CD25 as a marker for hepatocellular carcinoma. Oncology letters 2012;4(4):840-46. | Does not meet full eligibility criteria: includes people without cirrhosis |
| Cai, Jiabin, Chen, Lei, Zhang, Zhou, Zhang, Xinyu, Lu, Xingyu, Liu, Weiwei, et al. Genome-wide mapping of 5-hydroxymethylcytosines in circulating cell-free DNA as a non-invasive approach for early detection of hepatocellular carcinoma. Gut 2019;68(12):2195-2005. | Exclude on population: not cirrhosis |
| Cai, L., Rao, X. H., Su, Q., Qin, J. S., Cai, L. Q., Hong, H., et al. Diagnostic value of joint detection of GP73 and AFP-L3 in primary hepatic carcinoma with low concentration of AFP. Journal of International Translational Medicine 2015;3(1):28-32. | Exclude on population: not cirrhosis |
| Cai, Mu-Yan, Tong, Zhu-Ting, Zheng, Fang, Liao, Yi-Ji, Wang, Yi, Rao, Hui-Lan, et al. EZH2 protein: a promising immunomarker for the detection of hepatocellular carcinomas in liver needle biopsies. Gut 2011;60(7):967-76. | Exclude on index test |
| Cai, Xin, Tang, Dongling, Chen, Juanjuan, Li, Huan. Evaluation of Serum FGL1 as Diagnostic Markers for HBV-Related Hepatocellular Carcinoma. Laboratory medicine 2023;54(3):270-81. | Does not meet full eligibility criteria: includes people without cirrhosis |
| Cai, Z., Zhang, J., He, Y., Xia, L., Dong, X., Chen, G., et al. Liquid biopsy by combining 5-hydroxymethylcytosine signatures of plasma cell-free DNA and protein biomarkers for diagnosis and prognosis of hepatocellular carcinoma. ESMO open 2021;6(1):100021. | Does not meet full eligibility criteria: includes people without cirrhosis |
| Camaggi, Carlo M., Zavatto, Elena, Gramantieri, Laura, Camaggi, Valeria, Strocchi, Elena, Righini, Roberto, et al. Serum albumin-bound proteomic signature for early detection and staging of hepatocarcinoma: sample variability and data classification. Clinical chemistry and laboratory medicine 2010;48(9):1319-26. | Exclude on index test |
| Campani, Claudia, Imbeaud, Sandrine, Couchy, Gabrielle, Ziol, Marianne, Hirsch, Theo Z., Rebouissou, Sandra, et al. Circulating tumour DNA in patients with hepatocellular carcinoma across tumour stages and treatments. Gut 2024;73(11):1870â€“1882. | Exclude on population: not cirrhosis |
| Cao, Bangrong, Yang, Lei, Rong, Weiqi, Feng, Lin, Han, Naijun, Zhang, Kaitai, et al. Latent transforming growth factor-beta binding protein-1 in circulating plasma as a novel biomarker for early detection of hepatocellular carcinoma. International journal of clinical and experimental pathology 2015;8(12):16046-54. | Exclude on population: not cirrhosis |
| Cao, Chunyu. Clinical significance of serum miR-768-3p in HBV-related hepatocellular carcinoma and its potential mechanism. Clinical and experimental medicine 2020;20(4):569-76. | Exclude on population: not cirrhosis |
| Cao, L. L., Han, Y., Wang, Y., Pei, L., Yue, Z., Qin, L., et al. Metabolic Profiling Identified a Novel Biomarker Panel for Metabolic Syndrome-Positive Hepatocellular Cancer. Frontiers in Endocrinology 2022;12():816748. | Exclude on population: not cirrhosis |

| Reference | Reason for exclusion |
|---|---|
| Cao, Lin-Lin, Han, Yi, Pei, Lin, Yue, Zhi-Hong, Liu, Bo-Yu, Cui, Jing-Wen, Jia, Mei. A Serum Metabolite Classifier for the Early Detection of Type 2 Diabetes Mellitus-Positive Hepatocellular Cancer. Metabolites 2022;12(7):n. pag.. | Exclude on population: not cirrhosis |
| Cao, Lin-Lin, Han, Yi, Wang, Yuanxiao, Pei, Lin, Yue, Zhihong, Qin, Li, et al. Metabolic Profiling Identified a Novel Biomarker Panel for Metabolic Syndrome-Positive Hepatocellular Cancer. Frontiers in endocrinology 2021;12():816748. | Exclude on population: not cirrhosis |
| Cao, S., Zhong, F., Chen, X., Ke, S., Zhong, X., Li, T., et al. The combination of serum lncRNA PTTG3P and mRNA PTTG1 serves as a diagnostic and prognostic marker for hepatocellular carcinoma. Molecular Medicine Reports 2025;31(2):44. | Does not meet full eligibility criteria: includes people without cirrhosis |
| Cao, Xinyi, Cao, Zhao, Shao, Yuyin, Liu, Chao, Yan, Guoquan, Meng, Xinmin, et al. Analysis of Serum Paraoxonase 1 Using Mass Spectrometry and Lectin Immunoassay in Patients With Alpha-Fetoprotein Negative Hepatocellular Carcinoma. Frontiers in oncology 2021;11():651421. | Exclude on index test |
| Capurro, Mariana, Wanless, Ian R, Sherman, Morris, Deboer, Gerrit, Shi, Wen, Miyoshi, Eiji. Glypican-3: a novel serum and histochemical marker for hepatocellular carcinoma. Gastroenterology 2003;125(1):89-97. | Does not meet full eligibility criteria: includes people without cirrhosis |
| Capurro, Mariana, Wanless, Ian R., Sherman, Morris, Deboer, Gerrit, Shi, Wen, Miyoshi, Eiji. Glypican-3: a novel serum and histochemical marker for hepatocellular carcinoma. Gastroenterology 2003;125(1):89-97. | Does not meet full eligibility criteria: includes people without cirrhosis |
| Carr, Brian I., Bag, Harika Gozukara, Ince, Volkan, Akbulut, Sami, Ersan, Veysel, Usta, Sertac, et al. A Combination of Blood Lymphocytes and AST Levels Distinguishes Patients with Small Hepatocellular Carcinomas from Non-cancer Patients. Journal of gastrointestinal cancer 2021;52(4):1211-16. | Exclude on index test |
| Carvalho-Gomes, A., Valcheva Valcheva, T. V., Sahuco, I., Vidal, E., Martinez-Arenas, L., Vinaixa, C., et al. External validation of models to predict hepatocellular carcinoma in Hepatitis C Virus cured F3-F4 patients. United European Gastroenterology Journal 2024;12(7):901 “ 910. | No 2x2 data |
| Casaril, M., Capra, F., Marchiori, L., Gabrielli, G. B., Nicoli, N., Corso, F., Baracchino, F. Serum copper and ceruloplasmin in early and in advanced hepatocellular carcinoma: diagnostic and prognostic relevance. Tumori 1989;75(5):498-502. | Exclude on index test |
| Castaldo, G. & Salvatore, F. Serum type-2 macro-creatine kinase isoenzyme is not a useful marker of severe liver diseases or neoplasia. Clinical biochemistry 1990;23(6):523-27. | Exclude on index test |
| Castaldo, G., Calcagno, G., Sibillo, R., Cuomo, R., Nardone, G., Castellano, L., et al. Quantitative analysis of aldolase A mRNA in liver discriminates between hepatocellular carcinoma and cirrhosis. Clinical chemistry 2000;46(7):901-06. | No 2x2 data |
| Castaldo, G., Oriani, G., Lofrano, M. M., Cimino, L., Topa, M., Budillon, G., Salvatore, F. Differential diagnosis between hepatocellular carcinoma and cirrhosis through a discriminant function based on results for serum analytes. Clinical chemistry 1996;42(8 Pt 1):1263-69. | Does not meet full eligibility criteria: pre-2005 |
| Caturelli, E., Pompili, M., Bartolucci, F., Siena, D. A., Sperandeo, M., Andriulli, A. Hemangioma-like lesions in chronic liver disease: diagnostic evaluation in patients. Radiology 2001;220(2):337-42. | Does not meet full eligibility criteria: pre-2005 |
| Caviglia, G. P., Fariselli, P., D'Ambrosio, R., Colombatto, P., Degasperi, E., Ricco, G., et al. Development and Validation of a PIVKA-II-Based Model for HCC Risk Stratification in Patients With HCV-Related Cirrhosis Successfully Treated With DAA. Alimentary Pharmacology and Therapeutics 2025;61(3):538 “ 549. | Exclude on study design: not a 1- or 2-gate test accuracy study |
| Caviglia, Gian P., Abate, Maria L., Gaia, Silvia, Petrini, Elisa, Bosco, Caterina, Olivero, Antonella, et al. Risk of hepatocellular carcinoma in HBV cirrhotic patients assessed by the combination of miR-122, AFP and PIVKA-II. Panminerva medica 2017;59(4):283-89. | Duplicate (including Cochrane includes) |
| Caviglia, Gian Paolo, Abate, Maria Lorena, Petrini, Elisa, Gaia, Silvia, Rizzetto, Mario. Highly sensitive alpha-fetoprotein, Lens culinaris agglutinin-reactive fraction of alpha-fetoprotein and des-gamma-carboxyprothrombin for hepatocellular carcinoma detection. Hepatology research : the official journal of the Japan Society of Hepatology 2016;46(3):E130-5. | Exclude on population: not cirrhosis |

| Reference | Reason for exclusion |
|---|---|
| Caviglia, Gian Paolo, Abate, Maria Lorena, Petrini, Elisa, Gaia, Silvia, Rizzetto, Mario. Highly sensitive alpha-fetoprotein, Lens culinaris agglutinin-reactive fraction of alpha-fetoprotein and des-gamma-carboxyprothrombin for hepatocellular carcinoma detection. Hepatology research : the official journal of the Japan Society of Hepatology 2016;46(3):E130-35. | Duplicate (including Cochrane includes) |
| Caviglia, Gian Paolo, Armandi, Angelo, Rosso, Chiara, Gaia, Silvia, Aneli, Serena, Rolle, Emanuela, et al. Biomarkers of Oncogenesis, Adipose Tissue Dysfunction and Systemic Inflammation for the Detection of Hepatocellular Carcinoma in Patients with Nonalcoholic Fatty Liver Disease. Cancers 2021;13(10):n. pag.. | Does not meet full eligibility criteria: includes people without cirrhosis |
| Caviglia, Gian Paolo, Nicolosi, Aurora, Abate, Maria Lorena, Carucci, Patrizia, Rosso, Chiara, Rolle, Emanuela, et al. Liver Cancer-Specific Isoform of Serine Protease Inhibitor Kazal for the Detection of Hepatocellular Carcinoma: Results from a Pilot Study in Patients with Dysmetabolic Liver Disease. Current oncology (Toronto, Ont.) 2022;29(8):5457-65. | Continuous test without threshold |
| Caviglia, Gian Paolo, Ribaldone, Davide Giuseppe, Abate, Maria Lorena, Ciancio, Alessia, Pellicano, Rinaldo, Smedile, Antonina. Performance of protein induced by vitamin K absence or antagonist-II assessed by chemiluminescence enzyme immunoassay for hepatocellular carcinoma detection: a meta-analysis. Scandinavian journal of gastroenterology 2018;53(6):734-40. | Exclude on study design: not a 1- or 2-gate test accuracy study |
| Cavus, B., Akyuz, F., Iliaz, R., Akyuz, U., Duranyildiz, D., Serilmez, M., et al. Assessment of prognostic and diagnostic value of some biomarkers in hepatocellular carcinoma. Experimental oncology 2020;42(3):208-14. | Exclude on population: healthy controls |
| Cedrone, A, Covino, M, Caturelli, E, Pompili, M, Lorenzelli, G, Villani, M R, et al. Utility of alpha-fetoprotein (AFP) in the screening of patients with virus-related chronic liver disease: does different viral etiology influence AFP levels in HCC? A study in 350 western patients. Hepato-gastroenterology 2000;47(36):1654-8. | Does not meet full eligibility criteria: pre-2005 |
| Cha, D. I., Jang, K. M., Kim, S. H., Kang, T. W. Liver Imaging Reporting and Data System on CT and gadoxetic acid-enhanced MRI with diffusion-weighted imaging. European Radiology 2017;27(10):4394-4005. | Exclude on population: clinical signs and symptoms of HCC |
| Chalasani, N, Horlander, J C Sr, Said, A, Hoen, H, Kopecky, K K, Stockberger, S M Jr, et al. Screening for hepatocellular carcinoma in patients with advanced cirrhosis. The American journal of gastroenterology 1999;94(10):2988-93. | Does not meet full eligibility criteria: pre-2005 |
| Chalasani, N. P., Camardo, M., Porter, K., Keilar Grevstad, E., Roberts, L. R., Kisiel, J., et al. MULTI-TARGET HEPATOCELLULAR CARCINOMA BLOOD TEST PERFORMANCE ACROSS A RANGE OF SPECIFICITY CUT-OFF VALUES. Hepatology 2023;77(5):E142. | CONFERENCE ABSTRACT |
| Chalasani, N. P., Porter, K., Book, A. J., Xiong, K. M., Ramasubramanian, T. S., Kielar-Grevstad, E., et al. THE MULTI-TARGET HEPATOCELLULAR CARCINOMA BLOOD TEST PROVIDES HIGH SENSITIVITY FOR DETECTING EARLY-STAGE HEPATOCELLULAR CARCINOMA ACROSS IMPORTANT PATIENT SUBGROUPS. Gastroenterology 2022;162(7 Supplement):S-1130. | CONFERENCE ABSTRACT |
| Chalasani, N., Horlander, J. C., Sr., Said, A., Hoen, H., Kopecky, K. K., Stockberger, S. M., Jr., et al. Screening for hepatocellular carcinoma in patients with advanced cirrhosis. The American journal of gastroenterology 1999;94(10):2988-93. | Does not meet full eligibility criteria: pre-2005 |
| Chalasani, Naga P., Ramasubramanian, Tiruvidaimarudur S., Bhattacharya, Abhik, Olson, Marilyn C., Edwards V, David K., Roberts, Lewis R., et al. A Novel Blood-Based Panel of Methylated DNA and Protein Markers for Detection of Early-Stage Hepatocellular Carcinoma. Clinical gastroenterology and hepatology : the official clinical practice journal of the American Gastroenterological Association 2021;19(12):2597-605.ee4. | Does not meet full eligibility criteria: includes people without cirrhosis |
| Chan H.L.-Y., Wong G.L.H., Lo A.O.S. Revisit of alpha-fetoprotein as a tumor marker for hepatocellular carcinoma in chronic hepatitis B patients receiving entecavir. Hepatology 2013;58(4 SUPPL. 1):1256A. | Exclude on population: not cirrhosis |
| Chan Kyo, Kim & Jae Hoon, Lim. Detection of hepatocellular carcinomas and dysplastic nodules in cirrhotic liver: Accuracy of ultrasonography in transplant patients. Journal of Ultrasound in Medicine 2001;20(2):99-104. | Does not meet full eligibility criteria: pre-2005 |

| Reference | Reason for exclusion |
|---|---|
| Chan, H. L. Y., Hu, Y., Malinowsky, K., Madin, K., Kroeniger, K., Hou, J. Prospective appraisal of clinical diagnostic algorithms for hepatocellular carcinoma surveillance in Chinese patients with chronic hepatitis B infection. Scientific reports 2024;14(1):28996. | Does not meet full eligibility criteria: includes people without cirrhosis |
| Chan, Henry L. Y., Vogel, Arndt, Berg, Thomas, De Toni, Enrico N., Kudo, Masatoshi, Trojan, Jorg, et al. Performance evaluation of the Elecsys PIVKA-II and Elecsys AFP assays for hepatocellular carcinoma diagnosis. JGH open : an open access journal of gastroenterology and hepatology 2022;6(5):292-300. | Does not meet full eligibility criteria: includes people without cirrhosis |
| Chan, K. C. Allen, Lai, Paul B. S., Mok, Tony S. K., Chan, Henry L. Y., Ding, Chunming, Yeung, S. W. Quantitative analysis of circulating methylated DNA as a biomarker for hepatocellular carcinoma. Clinical chemistry 2008;54(9):1528-36. | Does not meet full eligibility criteria: includes people without cirrhosis |
| Chan, Michael Vinchill, McDonald, Stephen J., Ong, Yang-Yi, Mastrocostas, Katerina, Ho, Edwin, Huo, Ya Ruth, et al. HCC screening: assessment of an abbreviated non-contrast MRI protocol. European radiology experimental 2019;3(1):49. | Exclude on population: not cirrhosis |
| Chan, Rebecca W. Y., Wong, John, Chan, Henry L. Y., Mok, Tony S. K., Lo, Wyatt Y. W., Lee, Vincent, et al. Aberrant concentrations of liver-derived plasma albumin mRNA in liver pathologies. Clinical chemistry 2010;56(1):82-89. | Exclude on target condition: not HCC |
| Chan, Stephen L, Mo, Frankie, Johnson, Philip J, Siu, Deyond Y W, Chan, Michael H M, Lau, Wan Y, et al. Performance of serum alpha-fetoprotein levels in the diagnosis of hepatocellular carcinoma in patients with a hepatic mass. HPB : the official journal of the International Hepato Pancreato Biliary Association 2014;16(4):366-72. | Exclude on population: clinical signs and symptoms of HCC |
| Chan, Y. S., Cho, C. C. M., Yuen, T. Y., Hung, H. Y., Wong, P. K. M., Sou, M. M. Y., et al. Contrast-Enhanced Ultrasound with Perfluorobutane for Hepatocellular Cancer Surveillance: Our Initial Local Experience. Hong Kong Journal of Radiology 2024;27(1):e16 “ e27. | Does not meet full eligibility criteria: includes people without cirrhosis |
| Chang, Jong-In, Sinn, Dong Hyun, Jeong, Woo Kyoung, Hwang, Jeong Ah, Won, Ho Young, Kim, Kyunga, et al. Imaging features of hepatobiliary MRI and the risk of hepatocellular carcinoma development. Scandinavian journal of gastroenterology 2022;57(12):1470-77. | Exclude on study design: not a 1- or 2-gate test accuracy study |
| Chang, Te-Sheng, Wu, Yu-Chih, Tung, Shui-Yi, Wei, Kuo-Liang, Hsieh, Yung-Yu, Huang, Hao-Chun, et al. Alpha-Fetoprotein Measurement Benefits Hepatocellular Carcinoma Surveillance in Patients with Cirrhosis. The American journal of gastroenterology 2015;110(6):836-45. | Duplicate (including Cochrane includes) |
| Chang, Te-Sheng, Wu, Yu-Chih, Tung, Shui-Yi, Wei, Kuo-Liang, Hsieh, Yung-Yu, Huang, Hao-Chun, et al. Corrigendum: Alpha-Fetoprotein Measurement Benefits Hepatocellular Carcinoma Surveillance in Patients With Cirrhosis. The American journal of gastroenterology 2016;111(11):1668. | Exclude on study design: not a 1- or 2-gate test accuracy study |
| Chang, W Y. Complements as new diagnostic tools of hepatocellular carcinoma in cirrhotic patients. Cancer 1988;62(2):227-32. | Does not meet full eligibility criteria: pre-2005 |
| Chang, W. Y. Complements as new diagnostic tools of hepatocellular carcinoma in cirrhotic patients. Cancer 1988;62(2):227-32. | Does not meet full eligibility criteria: pre-2005 |
| Chang, W. Y. Serum B protein in hepatocellular carcinoma and liver cirrhosis. Gaoxiong yi xue ke xue za zhi = The Kaohsiung journal of medical sciences 1990;6(11):599-605. | Does not meet full eligibility criteria: pre-2005 |
| Chang, Y. C., Ho, C. L., Chen, H. H. W., Chang, T. T., Lai, W. W., Dai, Y. C., Lee, W. Y. Molecular diagnosis of primary liver cancer by microsatellite DNA analysis in the serum. British Journal of Cancer 2002;87(12):1449-53. | Exclude on population: not cirrhosis |
| Chao, Y. LncRNA-D16366 is a potential biomarker for diagnosis and prognosis of hepatocellular carcinoma. Medical Science Monitor 2019;25():6581-86. | Exclude on population: not cirrhosis |
| Chapman, R. W. & Bassendine, M. F. Serum ferritin and binding of serum ferritin to concanavalin A as a tumor marker in patients with primary liver cell cancer and chronic liver disease. Digestive Diseases and Sciences 1982;27(2):111-16. | Does not meet full eligibility criteria: pre-2005 |

| Reference | Reason for exclusion |
|---|---|
| Chaturvedi, Abhishek, Bhargava, Puneet, Kolokythas, Orpheus, Mitsumori, Lee M. Computer-assisted evaluation of contrast kinetics for detection of hepatocellular carcinoma on magnetic resonance imaging. Current problems in diagnostic radiology 2015;44(1):8-14. | Exclude on population: clinical signs and symptoms of HCC |
| Chayvialle, J A, Brissot, P, Pelletier, M J, Hita de Nercy, Y, Lambert, R. alpha-Fetoprotein screening in patients with idiopathic hemochromatosis and liver cirrhosis. Digestion 1977;16(1-2):118-27. | Does not meet full eligibility criteria: pre-2005 |
| Chen, C. J., Lu, S. N., You, S. L., Wu, M. H., Wang, L. Y., Lee, L. T., et al. [Community-based hepatocellular carcinoma screening in seven townships in Taiwan]. Journal of the Formosan Medical Association = Taiwan yi zhi 1995;94 Suppl 2():S94-102. | Does not meet full eligibility criteria: pre-2005 |
| Chen, D S. Serum alphafetoprotein in hepatocellular carcinoma. Cancer 1977;40(2):779-83. | Does not meet full eligibility criteria: includes people without cirrhosis |
| Chen, Dion, Jain, Surbhi, Su, Ying-Hsu. Building Classification Models with Combined Biomarker Tests: Application to Early Detection of Liver Cancer. Journal of statistical science and application 2017;5(3):91-103. | Does not meet full eligibility criteria: includes people without cirrhosis |
| Chen, Guangji, Xie, Dihuo, Zhang, Ping. Circular RNA hsa_circ_0000437 may be used as a new indicator for the diagnosis and prognosis of hepatocellular carcinoma. Bioengineered 2022;13(6):14118-24. | Exclude on population: not cirrhosis |
| Chen, H. B. & Gu, Y. M. The expression level of serum Golgi protein-73 in primary hepatocellular carcinoma and its clinical value in making early diagnosis. Journal of Interventional Radiology (China) 2014;23(6):528-31. | Foreign language |
| Chen, Hongda, Zhang, Yue, Li, Siwen, Li, Ni, Chen, Yuhan, Zhang, Bei, et al. Direct comparison of five serum biomarkers in early diagnosis of hepatocellular carcinoma. Cancer management and research 2018;10():1947-58. | Duplicate (including Cochrane includes) |
| Chen, Hongda, Zhang, Yue, Li, Siwen, Li, Ni, Chen, Yuhan, Zhang, Bei, et al. Direct comparison of five serum biomarkers in early diagnosis of hepatocellular carcinoma. Cancer management and research 2018;10(101512700):1947-58. | Does not meet full eligibility criteria: includes people without cirrhosis |
| Chen, Hui, Sun, Ling-yu, Zheng, Hong-qun, Zhang, Qi-fan. Total serum DNA and DNA integrity: diagnostic value in patients with hepatitis B virus-related hepatocellular carcinoma. Pathology 2012;44(4):318-24. | Exclude on population: not cirrhosis |
| Chen, J., Wu, M., Gong, J., Liu, Z., He, G., Zhu, H., Li, Z. Influence of Alanine Transaminase Levels on Alpha-Fetoprotein for Predicting Hepatocellular Carcinoma in Patients with Hepatitis B Infection. BioMed Research International 2020;2020():2043715. | Exclude on population: not cirrhosis |
| Chen, J-G, Parkin, D M, Chen, Q-G, Lu, J-H, Shen, Q-J, Zhang, B-C. Screening for liver cancer: results of a randomised controlled trial in Qidong, China. Journal of medical screening 2003;10(4):204-9. | Exclude on population: not cirrhosis |
| Chen, Juanjuan, Tang, Dongling, Li, Huan. Expression changes of serum LINC00941 and LINC00514 in HBV infection-related liver diseases and their potential application values. Journal of clinical laboratory analysis 2022;36(1):e24143. | Does not meet full eligibility criteria: includes people without cirrhosis |
| Chen, Juanjuan, Tang, Dongling, Xu, Chu, Niu, Zhili, Li, Huan, Li, Yan. Evaluation of Serum GDF15, AFP, and PIVKA-II as Diagnostic Markers for HBV-Associated Hepatocellular Carcinoma. Laboratory medicine 2021;52(4):381-89. | Does not meet full eligibility criteria: includes people without cirrhosis |
| Chen, Ken, Zhang, Hong, Zhang, Li-Na, Ju, Shao-Qing, Qi, Jing, Huang, Dong-Feng, et al. Value of circulating cell-free DNA in diagnosis of hepatocelluar carcinoma. World journal of gastroenterology 2013;19(20):3143-49. | Exclude on population: healthy controls |
| Chen, L., Wang, F., Ju, L. L. Clinical application of serum miR-493-5p detection in patients with hepatocellular carcinoma. Chinese Journal of Cancer Prevention and Treatment 2019;26(5):306-10. | Foreign language |
| Chen, La-Mei, Xiang, Lin, Sun, Wei-Juan, Zhai, Yu-Jia, Gao, Shuai, Fan, Yu-Chen. Diagnostic Value of the Hypomethylation of the WISP1 Promoter in Patients with Hepatocellular Carcinoma Associated with Hepatitis B Virus. The Tohoku journal of experimental medicine 2020;252(4):297-307. | Exclude on population: not cirrhosis |

| Reference | Reason for exclusion |
|---|---|
| Chen, Lei, Abou-Alfa, Ghassan K., Zheng, Bo, Liu, Jing-Feng, Bai, Jian, Du, Lu-Tao, et al. Genome-scale profiling of circulating cell-free DNA signatures for early detection of hepatocellular carcinoma in cirrhotic patients. Cell research 2021;31(5):589-92. | Does not meet full eligibility criteria: includes people without cirrhosis |
| Chen, Lei, Ho, David W. Y., Lee, Nikki P. Y., Sun, Stella, Lam, Brian, Wong, Kwong-Fai, et al. Enhanced detection of early hepatocellular carcinoma by serum SELDI-TOF proteomic signature combined with alpha-fetoprotein marker. Annals of surgical oncology 2010;17(9):2518-25. | Does not meet full eligibility criteria: includes people without cirrhosis |
| Chen, Lin, Chu, Feihu, Cao, Yali, Shao, Jianguo. Serum miR-182 and miR-331-3p as diagnostic and prognostic markers in patients with hepatocellular carcinoma. Tumour biology : the journal of the International Society for Oncodevelopmental Biology and Medicine 2015;36(10):7439-47. | Duplicate (including Cochrane includes) |
| Chen, Lin, Chu, Feihu, Cao, Yali, Shao, Jianguo. Serum miR-182 and miR-331-3p as diagnostic and prognostic markers in patients with hepatocellular carcinoma. Tumour biology : the journal of the International Society for Oncodevelopmental Biology and Medicine 2015;36(10):7439-47. | Exclude on population: not cirrhosis |
| Chen, Min, Li, Guohua, Yan, Jian, Lu, Xiuzhi, Cui, Jianwei, Ni, Zhengxian, et al. Reevaluation of glypican-3 as a serological marker for hepatocellular carcinoma. Clinica chimica acta; international journal of clinical chemistry 2013;423():105-11. | Exclude on index test |
| Chen, Ming, Zheng, Tenghao, Han, Shuangyin, Zhang, Lida, Bai, Yangqiu, Fang, Xinhui, Ding, Song-Ze. A preliminary study of plasma cyclase-associated protein 2 as a novel biomarker for early stage and alpha-fetoprotein negative hepatocellular carcinoma patients. Clinics and research in hepatology and gastroenterology 2015;39(2):215-21. | Does not meet full eligibility criteria: includes people without cirrhosis |
| Chen, Ningxin, Motosugi, Utaroh, Morisaka, Hiroyuki, Ichikawa, Shintaro, Sano, Katsuhiro, Ichikawa, Tomoaki, et al. Added Value of a Gadoxetic Acid-enhanced Hepatocyte-phase Image to the LI-RADS System for Diagnosing Hepatocellular Carcinoma. Magnetic resonance in medical sciences : MRMS : an official journal of Japan Society of Magnetic Resonance in Medicine 2016;15(1):49-59. | Exclude on population: clinical signs and symptoms of HCC |
| Chen, Q. F., Luo, X. M., Zhao, M. P. Dynamic monitoring of serum alpha-fetoprotein and its correlation with early hepatocellular carcinoma in patients with chronic hepatitis B. Biomedical Research (India) 2017;28(18):8100-04. | Exclude on population: not cirrhosis |
| Chen, S., Fu, Z., Wen, S., Yang, X., Yu, C., Zhou, W., Lin, Y. Expression and Diagnostic Value of miR-497 and miR-1246 in Hepatocellular Carcinoma. Frontiers in Genetics 2021;12():666306. | Exclude on population: healthy controls |
| Chen, Shanshan, Chen, Hao, Gao, Shanshan, Qiu, Shili, Zhou, Hu, Yu, Mingxia. Differential expression of plasma microRNA-125b in hepatitis B virus-related liver diseases and diagnostic potential for hepatitis B virus-induced hepatocellular carcinoma. Hepatology research : the official journal of the Japan Society of Hepatology 2017;47(4):312-20. | Does not meet full eligibility criteria: includes people without cirrhosis |
| Chen, Shipeng, Zhang, Zihan, Wang, Ying, Fang, Meng, Zhou, Jun, Li, Ya, et al. Using Quasispecies Patterns of Hepatitis B Virus to Predict Hepatocellular Carcinoma With Deep Sequencing and Machine Learning. The Journal of infectious diseases 2021;223(11):1887-96. | Exclude on population: not cirrhosis |
| Chen, Shuying, Mao, Yinqi, Chen, Wei, Liu, Chenbin, Wu, Han, Zhang, Jingjun, et al. Serum exosomal miR-34a as a potential biomarker for the diagnosis and prognostic of hepatocellular carcinoma. Journal of Cancer 2022;13(5):1410-17. | Exclude on population: healthy controls |
| Chen, Siyuan, Li, Junhong, Tan, Xiaodan, Xu, Qi, Mo, Yuncong, Qin, Hongyan, et al. Clinical role of combining alpha-fetoprotein and lens culinaris agglutinin-reactive fraction of alpha-fetoprotein for hepatocellular carcinoma: Evidence from literature and an original study. Journal of clinical laboratory analysis 2020;34(7):e23262. | Exclude on population: healthy controls |
| Chen, X. & Mohammed, A. F. Assessment of the Clinical Value of Platelet-to-Lymphocyte Ratio in Patients with Hepatocellular Carcinoma. Clinical and Applied Thrombosis/Hemostasis 2024;30():n. pag.. | Exclude on index test |
| Chen, X. Clinical significance of mir-195 in hepatocellular carcinoma and its biological function in tumor progression. OncoTargets and Therapy 2019;12():527-34. | Exclude on population: not cirrhosis |

| Reference | Reason for exclusion |
|---|---|
| Chen, X., Fu, S., Chen, F., Chen, H. Identification of tumor-associated antigens in human hepatocellular carcinoma by autoantibodies. Oncology Reports 2008;20(4):979-85. | Genomic biomarker not validated |
| Chen, Y., Lin, Y. Y., Hu, M. H. Clinical research of AFP variant with microcentrifugalcolumn method and crossed affinity immunoelectrophoresis autoradiography method. Zhonghua shi yan he lin chuang bing du xue za zhi = Zhonghua shiyan he linchuang bingduxue zazhi = Chinese journal of experimental and clinical virology 2008;22(5):379-81. | Foreign language |
| Chen, Yi, Chen, Jin, Liu, Yizhao, Li, Shiliang. Plasma miR-15b-5p, miR-338-5p, and miR-764 as Biomarkers for Hepatocellular Carcinoma. Medical science monitor : international medical journal of experimental and clinical research 2015;21():1864-71. | Does not meet full eligibility criteria: includes people without cirrhosis |
| Chen, Yifan, Barefoot, Megan E., Varghese, Rency S., Wang, Kuijun, Di Poto, Cristina. Integrative Analysis to Identify Race-Associated Metabolite Biomarkers for Hepatocellular Carcinoma. Annual International Conference of the IEEE Engineering in Medicine and Biology Society. IEEE Engineering in Medicine and Biology Society. Annual International Conference 2020;2020():5300-03. | Exclude on study design: not a 1- or 2-gate test accuracy study |
| Chen, Yueming, Dong, Xueyan, Yu, Daojun. Serum miR-96 is a promising biomarker for hepatocellular carcinoma in patients with chronic hepatitis B virus infection. International journal of clinical and experimental medicine 2015;8(10):18462-68. | Exclude on population: not cirrhosis |
| Chen, Yuxin, Wu, Yanping, Zhang, Xiao, Zeng, Hong, Liu, Ya, Wu, Qi, et al. Angiopoietin-2 (Ang-2) is a useful serum tumor marker for liver cancer in the Chinese population. Clinica chimica acta; international journal of clinical chemistry 2018;478():18-27. | Exclude on population: not cirrhosis |
| Chen, Z., Dong, M., Wu, X., Lin, Q., Li, X., Wen, J., Ma, X. The expression of soluble endoglin in patients with liver cirrhosis and hepatocellular carcinoma and its clinical significance. Chinese Journal of Clinical Oncology 2010;37(19):1097-1001. | Foreign language |
| Cheng J., Gholami P., Kappus M. Evaluation of diagnostic performance of alpha-fetoprotein (AFP), AFP-l3 and des-gamma-carboxy prothrombin (DCP) for HCV and non-HCV related hepatocellular carcinoma (HCC) after long-term follow-up. American Journal of Gastroenterology 2012;107(SUPPL. 1):S145-46. | CONFERENCE ABSTRACT |
| Cheng, Chi-Tung, Cai, Jinzheng, Teng, Wei, Zheng, Youjing, Huang, Yu-Ting, Wang, Yu-Chao, et al. A flexible three-dimensional heterophase computed tomography hepatocellular carcinoma detection algorithm for generalizable and practical screening. Hepatology communications 2022;6(10):2901-13. | Does not meet full eligibility criteria: includes people without cirrhosis |
| Cheng, Chi-Tung, Cai, Jinzheng, Teng, Wei, Zheng, Youjing, Huang, Yu-Ting, Wang, Yu-Chao, et al. A flexible three-dimensional heterophase computed tomography hepatocellular carcinoma detection algorithm for generalizable and practical screening. Hepatology communications 2022;():n. pag.. | Exclude on population: not cirrhosis |
| Cheng, Kai, Shi, Jie, Liu, Zixin, Jia, Yin, Qin, Qin, Zhang, Hui, et al. A panel of five plasma proteins for the early diagnosis of hepatitis B virus-related hepatocellular carcinoma in individuals at risk. EBioMedicine 2020;52():102638. | Does not meet full eligibility criteria: includes people without cirrhosis |
| Cheng, Ken-Sheng, Tang, Hui-Ling, Chou, Fu-Tsan, Chou, Jen-Wei, Hsu, Chang-Hu, Yu, Cheng-Ju, Kao, Shung-Te. Cytokine evaluation in liver cirrhosis and hepatocellular carcinoma. Hepato-gastroenterology 2009;56(93):1105-10. | No 2x2 data |
| Cheng, Ningtao & Lou, Bin. Discovering the digital biomarker of hepatocellular carcinoma in serum with SERS-based biosensors and intelligence vision. Colloids and surfaces. B, Biointerfaces 2023;226():113315. | Exclude on population: not cirrhosis |
| Cheng, T. Clinical significance of AFP-L3,Hsp90alpha test in the diagnosis of hepatocellular carcinoma with low and medium concentration of AFP. Chinese Journal of Cancer Prevention and Treatment 2018;25(21):1511-14. | Foreign language |
| Chernyak, Victoria, van der Pol, Christian B., Vernuccio, Federica. The Global Reading Room: Imaging Detection of Hepatocellular Carcinoma. AJR. American journal of roentgenology 2022;218(5):922-23. | Exclude on study design: not a 1- or 2-gate test accuracy study |
| Chignard, Nicolas, Shang, Sufen, Wang, Hong, Marrero, Jorge, Brechot, Christian, Hanash, Samir. Cleavage of endoplasmic reticulum proteins in | Does not meet full eligibility criteria: includes people without cirrhosis |

| Reference | Reason for exclusion |
|---|---|
| hepatocellular carcinoma: Detection of generated fragments in patient sera. Gastroenterology 2006;130(7):2010-22. | |
| Chimparlee, Nitinan, Chuaypen, Natthaya, Khlaiphuengsin, Apichaya, Pinjaroen, Nutcha, Payungporn, Sunchai, Poovorawan, Yong. Diagnostic and Prognostic Roles of Serum Osteopontin and Osteopontin Promoter Polymorphisms in Hepatitis B-related Hepatocellular Carcinoma. Asian Pacific journal of cancer prevention : APJCP 2015;16(16):7211-17. | Does not meet full eligibility criteria: includes people without cirrhosis |
| Chimparlee, Nitinan, Chuaypen, Natthaya, Khlaiphuengsin, Apichaya, Pinjaroen, Nutcha, Payungporn, Sunchai, Poovorawan, Yong. Diagnostic and Prognostic Roles of Serum Osteopontin and Osteopontin Promoter Polymorphisms in Hepatitis B-related Hepatocellular Carcinoma. Asian Pacific journal of cancer prevention : APJCP 2015;16(16):7211-7. | Does not meet full eligibility criteria: includes people without cirrhosis |
| Chio, L. F. Changes in serum alpha 1 antitrypsin, alpha1 acid glycoprotein and beta 2 glycoprotein I in patients with malignant hepatocellular carcinoma. Cancer 1979;43(2):596-604. | Does not meet full eligibility criteria: includes people without cirrhosis |
| Cho, Eun Ju, Leem, Sangseob, Kim, Sun Ah, Yang, Jinho, Lee, Yun Bin, Kim, Soon Sun, et al. Circulating Microbiota-Based Metagenomic Signature for Detection of Hepatocellular Carcinoma. Scientific reports 2019;9(1):7536. | Exclude on population: healthy controls |
| Cho, Hyo Jung, Eun, Jung Woo, Baek, Geum Ok, Seo, Chul Won, Ahn, Hye Ri, Kim, Soon Sun, Cho, Sung Won. Serum Exosomal MicroRNA, miR-10b-5p, as a Potential Diagnostic Biomarker for Early-Stage Hepatocellular Carcinoma. Journal of clinical medicine 2020;9(1):n. pag.. | Does not meet full eligibility criteria: includes people without cirrhosis |
| Cho, Hyo Jung, Kim, Bohyun, Lee, Jung-Dong, Kang, Dae Ryong, Kim, Jai Keun, Lee, Jei Hee, et al. Development of Risk Prediction Model for Hepatocellular Carcinoma Progression of Indeterminate Nodules in Hepatitis B Virus-Related Cirrhotic Liver. The American journal of gastroenterology 2017;112(3):460-70. | Exclude on population: clinical signs and symptoms of HCC |
| Cho, S. Y., Yang, J. J., You, E., Kim, B. H., Shim, J., Lee, H. J., et al. Mean platelet volume/platelet count ratio in hepatocellular carcinoma. Platelets 2013;24(5):375-77. | Exclude on index test |
| Choi, D., Kim, S., Lim, J., Lee, W., Jang, H., Lee, S. Preoperative detection of hepatocellular carcinoma: ferumoxides-enhanced mr imaging versus combined helical CT during arterial portography and CT hepatic arteriography. AJR. American journal of roentgenology 2001;176(2):475-82. | Exclude on population: clinical signs and symptoms of HCC |
| Choi, J. Y., Choi, S. H., Byun, J. H., Lee, S. J., Kim, S. Y., Won, H. J. Liver Imaging Reporting and Data System version 2018 for diagnosing hepatocellular carcinoma in alcoholic liver cirrhosis and virus-related cirrhosis. European Journal of Radiology 2023;168():111139. | Exclude on population: clinical signs and symptoms of HCC |
| Choi, J., Kim, G. A., Han, S., Lee, W., Chun, S. Longitudinal Assessment of Three Serum Biomarkers to Detect Very Early-Stage Hepatocellular Carcinoma. Hepatology 2019;69(5):1983-94. | Duplicate (including Cochrane includes) |
| Choi, J., Lee, S. Y., Lim, Y. S., Lee, W. Longitudinal assessment of alphafetoprotein lectin-reactive alphafetoprotein and des-gamma-carboxy prothrombin for the early detection of hepatocellular carcinoma. Gut 2018;67(Supplement 2):A102. | Exclude on population: not cirrhosis |
| Choi, Ji Young, Choi, Sang Hyun, Byun, Jae Ho, Lee, So Jung, Kim, So Yeon, Won, Hyung Jin. Liver Imaging Reporting and Data System version 2018 for diagnosing hepatocellular carcinoma in alcoholic liver cirrhosis and virus-related cirrhosis. European journal of radiology 2023;168():111139. | Exclude on population: clinical signs and symptoms of HCC |
| Choi, Jong Young, Jung, Seung Won, Kim, Hee Yeon, Kim, Myungshin, Kim, Yonggoo, Kim, Dong Goo. Diagnostic value of AFP-L3 and PIVKA-II in hepatocellular carcinoma according to total-AFP. World journal of gastroenterology 2013;19(3):339-46. | Duplicate (including Cochrane includes) |
| Choi, Jonggi & Tayob, Nabihah. Detecting Early Hepatocellular Carcinoma in Patients With Chronic Hepatitis B Using Longitudinal alpha-Fetoprotein Screening. Clinical gastroenterology and hepatology : the official clinical practice journal of the American Gastroenterological Association 2023;21(6):1590-97. | Does not meet full eligibility criteria: includes people without cirrhosis |

| Reference | Reason for exclusion |
|---|---|
| Choi, Jonggi, Kim, Gi-Ae, Han, Seungbong, Lee, Woochang, Chun, Sail. Longitudinal Assessment of Three Serum Biomarkers to Detect Very Early-Stage Hepatocellular Carcinoma. Hepatology (Baltimore, Md.) 2019;69(5):1983-94. | Does not meet full eligibility criteria: includes people without cirrhosis |
| Choi, Sang Hyun, Byun, Jae Ho, Kim, So Yeon, Lee, So Jung, Won, Hyung Jin, Shin, Yong Moon. Liver Imaging Reporting and Data System v2014 With Gadoxetate Disodium-Enhanced Magnetic Resonance Imaging: Validation of LI-RADS Category 4 and 5 Criteria. Investigative radiology 2016;51(8):483-90. | Exclude on population: clinical signs and symptoms of HCC |
| Chong, N., Schoenberger, H., Yekkaluri, S., Fetzer, D. T., Rich, N. E., Yokoo, T. LIMITED ULTRASOUND VISUALIZATION IS ASSOCIATED WITH LOWER SENSITIVITY AND SPECIFICITY DURING HEPATOCELLULAR CARCINOMA SURVEILLANCE. Gastroenterology 2022;162(7 Supplement):S-1164. | CONFERENCE ABSTRACT |
| Chotipanich, Chanisa, Kunawudhi, Anchisa, Promteangtrong, Chetsadaporn, Tungsuppawattanakit, Puntira, Sricharunrat, Thaniya. Diagnosis of Hepatocellular Carcinoma Using C11 Choline PET/CT: Comparison with F18 FDG, ContrastEnhanced MRI and MDCT. Asian Pacific journal of cancer prevention : APJCP 2016;17(7):3569-73. | Exclude on population: clinical signs and symptoms of HCC |
| Chounta, Athina, Ellinas, Christofer, Tzanetakou, Vassiliki, Pliarhopoulou, Fani, Mplani, Virginia, Oikonomou, Angelos, Leventogiannis, Kostantinos. Serum soluble urokinase plasminogen activator receptor as a screening test for the early diagnosis of hepatocellular carcinoma. Liver international : official journal of the International Association for the Study of the Liver 2015;35(2):601-07. | No 2x2 data |
| Chrzanowska, Alicja, Grabon, Wojciech, Mielczarek-Puta, Magdalena. Significance of arginase determination in body fluids of patients with hepatocellular carcinoma and liver cirrhosis before and after surgical treatment. Clinical biochemistry 2014;47(12):1056-59. | No 2x2 data |
| Chrzanowska, Alicja, Mielczarek-Puta, Magdalena, Skwarek, Anna, Krawczyk, Marek. [Serum arginase activity in patients with liver cirrhosis and hepatocellular carcinoma]. Arginaza w surowicy krwi chorych z marskoscia i rakiem watrobowokomorkowym. 2007;60(5-6):215-18. | Foreign language |
| Chu, Hyung Jun, Heo, Jeong, Seo, Soo Boon, Kim, Gwang Ha, Kang, Dae Hwan, Song, Geun Am, Cho, Mong. Detection of aberrant p16INK4A methylation in sera of patients with liver cirrhosis and hepatocellular carcinoma. Journal of Korean medical science 2004;19(1):83-86. | Does not meet full eligibility criteria: includes people without cirrhosis |
| Chuaypen, Natthaya, Chittmittraprap, Salyavit, Pinjaroen, Nutcha, Sirichindakul, Boonchoo, Poovorawan, Yong, Tanaka, Yasuhito. Serum Wisteria floribunda agglutinin-positive Mac-2 binding protein level as a diagnostic marker of hepatitis B virus-related hepatocellular carcinoma. Hepatology research : the official journal of the Japan Society of Hepatology 2018;48(11):872-81. | Duplicate (including Cochrane includes) |
| Chuaypen, Natthaya, Chittmittraprap, Salyavit, Pinjaroen, Nutcha, Sirichindakul, Boonchoo, Poovorawan, Yong, Tanaka, Yasuhito. Serum Wisteria floribunda agglutinin-positive Mac-2 binding protein level as a diagnostic marker of hepatitis B virus-related hepatocellular carcinoma. Hepatology research : the official journal of the Japan Society of Hepatology 2018;48(11):872-81. | Does not meet full eligibility criteria: includes people without cirrhosis |
| Chuaypen, Natthaya, Sriphoosanaphan, Supachaya, Vorasittha, Athaya, Pinjaroen, Nutcha, Chaiyarit, Sakdithep, Thongboonkerd, Visith, Tangkijvanich, Pisit. Targeted Proteins Reveal Cathepsin D as a Novel Biomarker in Differentiating Hepatocellular Carcinoma from Cirrhosis and Other Liver Cancers. Asian Pacific journal of cancer prevention : APJCP 2022;23(6):2017-25. | Does not meet full eligibility criteria: includes people without cirrhosis |
| Chun, S., Rhie, S. Y., Ki, C. S., Kim, J. E. Evaluation of alpha-fetoprotein as a screening marker for hepatocellular carcinoma in hepatitis prevalent areas. Annals of Hepatology 2015;14(6):881-87. | Exclude on population: not cirrhosis |
| Chung, J. W., Jang, E. S., Kim, J., Jeong, S. H., Kim, N., Lee, D. H., Lee, K. H. Development of a nomogram for screening of hepatitis B virusassociated hepatocellular carcinoma. Oncotarget 2017;8(63):106499-51010. | Exclude on population: not cirrhosis |
| Chung, Jung Wha, Kim, Beom Hee, Lee, Chung Seop, Kim, Gi Hyun, Sohn, Hyung Rae, Min, Bo Young, et al. Optimizing Surveillance Performance of | Exclude on population: not cirrhosis |

| Reference | Reason for exclusion |
|---|---|
| Alpha-Fetoprotein by Selection of Proper Target Population in Chronic Hepatitis B. PloS one 2016;11(12):e0168189. | |
| Chung, Tae-Wook, Kim, Jeong-Ran, Suh, Jeong-Il, Lee, Young-Choon, Chang, Young-Chae, Chung, Tai Ho. Correlation between plasma levels of matrix metalloproteinase (MMP)-9 /MMP-2 ratio and alpha-fetoproteins in chronic hepatitis carrying hepatitis B virus. Journal of gastroenterology and hepatology 2004;19(5):565-71. | Exclude on study design: not a 1- or 2-gate test accuracy study |
| Cillo, Umberto, Navaglia, Filippo, Vitale, Alessandro, Molari, Alfiero, Basso, Daniela, Bassanello, Marco, et al. Clinical significance of alpha-fetoprotein mRNA in blood of patients with hepatocellular carcinoma. Clinica chimica acta; international journal of clinical chemistry 2004;347(1-2):129-38. | Does not meet full eligibility criteria: includes people without cirrhosis |
| Cimentepe, M., Ozturk, G., Balli, H. T., Komur, S., Doran, F., Binokay, H. The investigation of micrornas as potential biomarkers in serum and tissue for hepatitis B virus related hepatocellular carcinoma. Acta Medica Mediterranea 2021;37(2):1335-40. | Exclude on population: not cirrhosis |
| Clemente, Caterina, Elba, Silvana, Buongiorno, Gianpiero, Berloco, Pasquale, Guerra, Vito. Serum retinol and risk of hepatocellular carcinoma in patients with child-Pugh class A cirrhosis. Cancer letters 2002;178(2):123-29. | Exclude on index test |
| Clemente, Caterina, Elba, Silvana, Buongiorno, Gianpiero, Guerra, Vito, D'Attoma, Benedetta, Orlando, Antonella. Manganese superoxide dismutase activity and incidence of hepatocellular carcinoma in patients with Child-Pugh class A liver cirrhosis: a 7-year follow-up study. Liver international : official journal of the International Association for the Study of the Liver 2007;27(6):791-97. | Exclude on index test |
| Colagrande, S., Fargnoli, R., Dal Pozzo, F., Bindi, A., Rega, L. Value of hepatic arterial phase CT versus lipiodol ultrafluid CT in the detection of hepatocellular carcinoma. Journal of computer assisted tomography 2000;24(6):878-83. | Exclude on population: clinical signs and symptoms of HCC |
| Compagnon, Philippe, Grandadam, Stephane, Lorho, Richard, Turlin, Bruno, Camus, Christophe, Jianrong, Yang, et al. Liver transplantation for hepatocellular carcinoma without preoperative tumor biopsy. Transplantation 2008;86(8):1068-76. | Exclude on population: clinical signs and symptoms of HCC |
| Comunale, Mary Ann, Rodemich-Betesh, Lucy, Hafner, Julie, Wang, Mengjun, Norton, Pamela, Di Bisceglie, Adrian M., Block, Timothy. Linkage specific fucosylation of alpha-1-antitrypsin in liver cirrhosis and cancer patients: implications for a biomarker of hepatocellular carcinoma. PloS one 2010;5(8):e12419. | Exclude on index test |
| Comunale, Mary Ann, Wang, Mengjun, Rodemich-Betesh, Lucy, Hafner, Julie, Lamontagne, Anne, Klein, Andrew, et al. Novel changes in glycosylation of serum Apo-J in patients with hepatocellular carcinoma. Cancer epidemiology, biomarkers & prevention : a publication of the American Association for Cancer Research, cosponsored by the American Society of Preventive Oncology 2011;20(6):1222-29. | Exclude on index test |
| Cong, Min, Ou, Xiaojuan, Huang, Jian, Long, Jiang, Li, Tong, Liu, Xueen, et al. A Predictive Model Using N-Glycan Biosignatures for Clinical Diagnosis of Early Hepatocellular Carcinoma Related to Hepatitis B Virus. Omics : a journal of integrative biology 2020;24(7):415-23. | No 2x2 data |
| Correale, M., Giannuzzi, V., Iacovazzi, P. A., Valenza, M. A., Lanzillotta, S., Abbate, I., et al. Serum 90K/MAC-2BP glycoprotein levels in hepatocellular carcinoma and cirrhosis. Anticancer research 1999;19(4C):3469-72. | Does not meet full eligibility criteria: pre-2005 |
| Cortis, Kelvin, Liotta, Rosa, Miraglia, Roberto, Caruso, Settimo, Tuzzolino, Fabio. Incorporating the hepatobiliary phase of gadobenate dimeglumine-enhanced MRI in the diagnosis of hepatocellular carcinoma: increasing the sensitivity without compromising specificity. Acta radiologica (Stockholm, Sweden : 1987) 2016;57(8):923-31. | Exclude on population: clinical signs and symptoms of HCC |
| Cotsoglou, C. Diagnostic approaches for small hepatocellular carcinomas. Annals of Gastroenterology 2013;26(1):89-90. | Exclude on study design: not a 1- or 2-gate test accuracy study |
| Cotte, A. K., Cottet, V., Aires, V., Mouillot, T., Rizk, M., Vinault, S., et al. Phospholipid profiles and hepatocellular carcinoma risk and prognosis in cirrhotic patients. Oncotarget 2019;10(22):2161-72. | Exclude on index test |

| Reference | Reason for exclusion |
|---|---|
| Cottone, M, Marceno, M P, Maringhini, A, Rinaldi, F, Russo, G, Sciarrino, E, Turri, M. Ultrasound in the diagnosis of hepatocellular carcinoma associated with cirrhosis. Radiology 1983;147(2):517-9. | Exclude on population: clinical signs and symptoms of HCC |
| Cottone, M, Turri, M, Caltagirone, M, Maringhini, A, Sciarrino, E, Virdone, R, et al. Early detection of hepatocellular carcinoma associated with cirrhosis by ultrasound and alfafetoprotein: a prospective study. Hepato-gastroenterology 1988;35(3):101-3. | Does not meet full eligibility criteria: pre-2005 |
| Cottone, M., Turri, M., Caltagirone, M., Maringhini, A., Sciarrino, E., Virdone, R., et al. Early detection of hepatocellular carcinoma associated with cirrhosis by ultrasound and alfafetoprotein: a prospective study. Hepato-gastroenterology 1988;35(3):101-03. | Does not meet full eligibility criteria: pre-2005 |
| Court, Colin M., Hou, Shuang, Winograd, Paul, Segel, Nicholas H., Li, Qingyu Wilda, Zhu, Yazhen, et al. A novel multimarker assay for the phenotypic profiling of circulating tumor cells in hepatocellular carcinoma. Liver transplantation : official publication of the American Association for the Study of Liver Diseases and the International Liver Transplantation Society 2018;24(7):946-60. | Exclude on index test |
| Cui, Jiefeng, Kang, Xiaonan, Dai, Zhi, Huang, Cheng, Zhou, Haijun, Guo, Kun, et al. Prediction of chronic hepatitis B, liver cirrhosis and hepatocellular carcinoma by SELDI-based serum decision tree classification. Journal of cancer research and clinical oncology 2007;133(11):825-34. | Exclude on population: healthy controls |
| Cui, R, He, J, Zhang, F, Wang, B, Ding, H, Shen, H, Li, Y. Diagnostic value of protein induced by vitamin K absence (PIVKAII) and hepatoma-specific band of serum gamma-glutamyl transferase (GGTII) as hepatocellular carcinoma markers complementary to alpha-fetoprotein. British journal of cancer 2003;88(12):1878-82. | Does not meet full eligibility criteria: includes people without cirrhosis |
| Cui, R. & Li, Y. Combined detection of alpha-fetoprotein and Golgi protein 73 for primary hepatic carcinoma diagnosis. Chinese Journal of Clinical Oncology 2015;42(4):222-25. | Foreign language |
| Cui, R., He, J., Zhang, F., Wang, B., Ding, H., Shen, H., Li, Y. Diagnostic value of protein induced by vitamin K absence (PIVKAII) and hepatoma-specific band of serum gamma-glutamyl transferase (GGTII) as hepatocellular carcinoma markers complementary to alpha-fetoprotein. British journal of cancer 2003;88(12):1878-82. | Does not meet full eligibility criteria: includes people without cirrhosis |
| Cui, Rutao, Wang, Baoen, Ding, Huiguo, Shen, Hong, Li, Yimei. Usefulness of determining a protein induced by vitamin K absence in detection of hepatocellular carcinoma. Chinese medical journal 2002;115(1):42-45. | Does not meet full eligibility criteria: pre-2005 |
| Cui, Rutao, Wang, Baoen, Ding, Huiguo, Shen, Hong, Li, Yimei. Usefulness of determining a protein induced by vitamin K absence in detection of hepatocellular carcinoma. Chinese medical journal 2002;115(1):42-5. | Does not meet full eligibility criteria: pre-2005 |
| Cui, Zhuqingqing, Yu, Xin, Guo, Limin, Wei, Yuhua, Zheng, Shengmin, Li, Wenxia, et al. Combined analysis of serum alpha-fetoprotein and MAGE-A3-specific cytotoxic T lymphocytes in peripheral blood for diagnosis of hepatocellular carcinoma. Disease markers 2013;35(6):915-23. | Does not meet full eligibility criteria: includes people without cirrhosis |
| da Costa, Andre Nogueira, Plymoth, Amelie, Santos-Silva, Daniela, Ortiz-Cuaran, Sandra, Camey, Suzy, Guilloreau, Paule, et al. Osteopontin and latent-TGF beta binding-protein 2 as potential diagnostic markers for HBV-related hepatocellular carcinoma. International journal of cancer 2015;136(1):172-81. | Duplicate (including Cochrane includes) |
| da Costa, Andre Nogueira, Plymoth, Amelie, Santos-Silva, Daniela, Ortiz-Cuaran, Sandra, Camey, Suzy, Guilloreau, Paule, et al. Osteopontin and latent-TGF beta binding-protein 2 as potential diagnostic markers for HBV-related hepatocellular carcinoma. International journal of cancer 2015;136(1):172-81. | Exclude on population: not cirrhosis |
| da Silva, Priscila Henriques, Gomes, Matheus Menezes, de Matos, Carla Adriana Loureiro, de Souza E Silva, Ivonete Sandra, Gonzalez, Adriano Miziara, Torres, Ulysses S., Salazar, Gloria Maria Martinez. HCC Detection on Surveillance US: Comparing Focused Liver Protocol Using US LI-RADS Technical Guidelines to a General Complete Abdominal US Protocol. Journal of ultrasound in medicine : official journal of the American Institute of Ultrasound in Medicine 2021;40(11):2487-95. | No 2x2 data |

| Reference | Reason for exclusion |
|---|---|
| Daher, D., Gopal, P., Coignet, M. V., Xiao, V., Kurtzman, K. N. Performance of a Multi-cancer Early Detection Test for Detection of Hepatocellular Carcinoma in Patients With Cirrhosis. Clinical Gastroenterology and Hepatology 2025;23(2):369 “ 370. | CONFERENCE ABSTRACT |
| Dai, L., Ren, P., Liu, M., Imai, H., Tan, E. M. Using immunomic approach to enhance tumor-associated autoantibody detection in diagnosis of hepatocellular carcinoma. Clinical Immunology 2014;152(1-2):127-39. | Exclude on index test |
| Dai, Liping, Ren, Pengfei, Liu, Mei, Imai, Haruhiko, Tan, Eng M. Using immunomic approach to enhance tumor-associated autoantibody detection in diagnosis of hepatocellular carcinoma. Clinical immunology (Orlando, Fla.) 2014;152(1-2):127-39. | No 2x2 data |
| Dai, M. & Li, L. Clinical value of miRNA-122 in the diagnosis and prognosis of various types of cancer. Oncology Letters 2019;17(4):3919-29. | Exclude on population: clinical signs and symptoms of HCC |
| Dai, Ying, Chen, Min Hua, Fan, Zhi Hui, Yan, Kun, Yin, Shan Shan. Diagnosis of small hepatic nodules detected by surveillance ultrasound in patients with cirrhosis: Comparison between contrast-enhanced ultrasound and contrast-enhanced helical computed tomography. Hepatology research : the official journal of the Japan Society of Hepatology 2008;38(3):281-90. | Exclude on population: clinical signs and symptoms of HCC |
| Daif, Ahmed, Al-Azzawi, Mahmood A., Sakr, Moustafa A., Ismail, Hisham A. Noninvasive identification of molecular biomarkers of hepatocellular carcinoma in HCV-Egyptian patients. Journal of the Egyptian National Cancer Institute 2023;35(1):11. | Exclude on population: not cirrhosis |
| Dalal, K., Dalal, B., Bhatia, S., Shukla, A. Analysis of serum Haptoglobin using glycoproteomics and lectin immunoassay in liver diseases in Hepatitis B virus infection. Clinica chimica acta; international journal of clinical chemistry 2019;495():309-17. | No 2x2 data |
| Dat, Vo Hoang Xuan, Nhung, Bui Thi Huyen, Chau, Nguyen Ngoc Bao, Cuong, Pham Hung, Hieu, Vo Duc, Linh, Nguyen Thi Minh. Identification of potential microRNA groups for the diagnosis of hepatocellular carcinoma (HCC) using microarray datasets and bioinformatics tools. Heliyon 2022;8(2):e08987. | Exclude on population: tissue samples |
| Dawood, Ashraf A., Saleh, Amany A., Elbahr, Osama, Gohar, Suzy Fawzy. Inverse relationship between the level of miRNA 148a-3p and both TGF-beta1 and FIB-4 in hepatocellular carcinoma. Biochemistry and biophysics reports 2021;27():101082. | Does not meet full eligibility criteria: includes people without cirrhosis |
| Dawood, Reham M., Salum, Ghada M., El-Meguid, Mai Abd, Elsayed, Ahmed, Yosry, Ayman, Abdelaziz, Ashraf, et al. Development of a gene signature for predicting cirrhosis risk score of chronic liver disease associated with HCV infection in Egyptians. Microbial pathogenesis 2021;153():104805. | Exclude on population: not cirrhosis |
| De Mattia, Elena, Cecchin, Erika, Polesel, Jerry, Bignucolo, Alessia, Roncato, Rossana, Lupo, Francesco, et al. Genetic biomarkers for hepatocellular cancer risk in a caucasian population. World journal of gastroenterology 2017;23(36):6674-84. | Exclude on population: not cirrhosis |
| De Palma, M. & Coukos, G. TIE2-expressing monocytes: A novel cellular biomarker for hepatocellular carcinoma? Hepatology 2013;57(4):1294-96. | Exclude on study design: not a 1- or 2-gate test accuracy study |
| Debnath, P., Dalal, K., Dalal, B., Athalye, S., Chandnani, S., Jain, S., et al. Characterization and detection of Circulatory Tumor Cells (CTCs) using imaging flow cytometry in liver cirrhosis and Hepatocellular carcinoma patients. Journal of Clinical and Experimental Hepatology 2022;12(Supplement 2):S106-07. | CONFERENCE ABSTRACT |
| Debongnie, J. C., Pauls, C., Fievez, M. Prospective evaluation of the diagnostic accuracy of liver ultrasonography. Gut 1981;22(2):130-35. | No 2x2 data |
| Degasperi, Elisabetta, Perbellini, Riccardo, D'Ambrosio, Roberta, Uceda Renteria, Sara Colonia, Ceriotti, Ferruccio, Perego, Alberto, et al. Prothrombin induced by vitamin K absence or antagonist-II and alpha foetoprotein to predict development of hepatocellular carcinoma in Caucasian patients with hepatitis C-related cirrhosis treated with direct-acting antiviral agents. Alimentary pharmacology & therapeutics 2022;55(3):350-59. | Exclude on study design: not a 1- or 2-gate test accuracy study |

| Reference | Reason for exclusion |
| --- | --- |
| Demangeat, J. L., Manil, L., Demangeat, C., Rico, E., Staedel-Flaig, C., Duclos, B., et al. Is anti-alphafetoprotein immunoscintigraphy a promising approach for the diagnosis of hepatoma? Implications of a quantitative study in 41 patients. European Journal of Nuclear Medicine 1988;14(12):612-20. | Exclude on population: not cirrhosis |
| Demerdash, Hala M., Hussien, Hend M., Hassouna, Ehab. Detection of MicroRNA in Hepatic Cirrhosis and Hepatocellular Carcinoma in Hepatitis C Genotype-4 in Egyptian Patients. BioMed research international 2017;2017():1806069. | Exclude on population: not cirrhosis |
| Demirtas, C. O., Akin, S., Yilmaz Karadag, D., Yilmaz, T., Ciftci, U., Huseynov, J., et al. Enhancing Hepatocellular Carcinoma Surveillance: Comparative Evaluation of AFP, AFP-L3, DCP and Composite Models in a Biobank-Based Case-Control Study. Cancers 2025;17(14):2390. | Does not meet full eligibility criteria: includes people without cirrhosis |
| Deng, Huan, Fan, Xiude, Wang, Xiaoyun, Zeng, Lu, Zhang, Kun, Zhang, Xiaoge, et al. Serum pentraxin 3 as a biomarker of hepatocellular carcinoma in chronic hepatitis B virus infection. Scientific reports 2020;10(1):20276. | Does not meet full eligibility criteria: includes people without cirrhosis |
| Dengler, Mirko, Staufer, Katharina, Huber, Heidemarie, Stauber, Rudolf, Bantel, Heike, Weiss, Karl Heinz, et al. Soluble Axl is an accurate biomarker of cirrhosis and hepatocellular carcinoma development: results from a large scale multicenter analysis. Oncotarget 2017;8(28):46234-48. | Exclude on population: healthy controls |
| Dhayat, Sameer A., Husing, Anna, Senninger, Norbert, Schmidt, Hartmut H., Haier, Jorg, Wolters, Heiner. Circulating microRNA-200 Family as Diagnostic Marker in Hepatocellular Carcinoma. PloS one 2015;10(10):e0140066. | No 2x2 data |
| Di Martino, Michele, Marin, Daniele, Guerrisi, Antonino, Baski, Mahbubeh, Galati, Francesca, Rossi, Massimo, et al. Intraindividual comparison of gadoxetate disodium-enhanced MR imaging and 64-section multidetector CT in the Detection of hepatocellular carcinoma in patients with cirrhosis. Radiology 2010;256(3):806-16. | Exclude on population: clinical signs and symptoms of HCC |
| Di Poto, C., Ferrarini, A., Zhao, Y., Varghese, R. S., Tu, C., Zuo, Y., et al. Metabolomic characterization of hepatocellular carcinoma in patients with liver cirrhosis for biomarker discovery. Cancer Epidemiology Biomarkers and Prevention 2017;26(5):675-83. | No 2x2 data |
| Di Poto, Cristina, He, Shisi, Varghese, Rency S., Zhao, Yi, Ferrarini, Alessia, Su, Shan, et al. Identification of race-associated metabolite biomarkers for hepatocellular carcinoma in patients with liver cirrhosis and hepatitis C virus infection. PloS one 2018;13(3):e0192748. | No 2x2 data |
| Di Santo, R., Verdelli, F., Niccolini, B., Varca, S., Gaudio, A. D., Di Giacinto, F., et al. Exploring novel circulating biomarkers for liver cancer through extracellular vesicle characterization with infrared spectroscopy and plasmonics. Analytica Chimica Acta 2024;1319():342959. | No 2x2 data |
| DiCioccio, R. A. & Barlow, J. J. Evaluation of serum alpha-L-fucosidase as a marker for primary liver cancer. IRCS Medical Science 1985;13(9):849-50. | Does not meet full eligibility criteria: pre-2005 |
| Dick, D., Regenstein, F., Blazek, J. Liver transplantation for hepatocellular carcinoma: one center's experience, 1987-1994. Journal of transplant coordination : official publication of the North American Transplant Coordinators Organization (NATCO) 1996;6(3):145-47. | Does not meet full eligibility criteria: pre-2005 |
| Dietrich, C. F., Teufel, A., Sirlin, C. B. Surveillance of hepatocellular carcinoma by medical imaging. Quantitative Imaging in Medicine and Surgery 2019;9(11):1904-10. | Exclude on study design: not a 1- or 2-gate test accuracy study |
| Ding, Yezhou, Liu, Kehui, Xu, Yumin, Zhao, Qingqing, Lou, Shike, Xiang, Xiaogang, et al. Combination of inflammatory score/liver function and AFP improves the diagnostic accuracy of HBV-related hepatocellular carcinoma. Cancer medicine 2020;9(9):3057-69. | Duplicate (including Cochrane includes) |
| Ding, Yezhou, Liu, Kehui, Xu, Yumin, Zhao, Qingqing, Lou, Shike, Xiang, Xiaogang, et al. Combination of inflammatory score/liver function and AFP improves the diagnostic accuracy of HBV-related hepatocellular carcinoma. Cancer medicine 2020;9(9):3057-69. | Exclude on population: not cirrhosis |
| Dodd, G D 3rd, Miller, W J, Baron, R L, Skolnick, M L. Detection of malignant tumors in end-stage cirrhotic livers: efficacy of sonography as a screening technique. AJR. American journal of roentgenology 1992;159(4):727-33. | Does not meet full eligibility criteria: pre-2005 |

| Reference | Reason for exclusion |
|---|---|
| Dong, C., Chang, C. D., Zhao, D. D., Zhang, X. X., Guo, P. L., Dou, Y., Zhao, S. X. [Clinical value of plasma scaffold protein SEC16A in evaluating hepatitis B-related liver cirrhosis and hepatocellular carcinoma]. Zhonghua gan zang bing za zhi = Zhonghua ganzangbing zazhi = Chinese journal of hepatology 2023;31(6):621-26. | Foreign language |
| Dong, Min, Chen, Zhan-Hong, Li, Xing, Li, Xiao-Yun, Wen, Jing-Yun, Lin, Qu, et al. Serum Golgi protein 73 is a prognostic rather than diagnostic marker in hepatocellular carcinoma. Oncology letters 2017;14(5):6277-84. | Does not meet full eligibility criteria: includes people without cirrhosis |
| Dong, Xueyan, He, Hui, Zhang, Weiying, Yu, Daojun, Wang, Xianjun. Combination of serum RASSF1A methylation and AFP is a promising non-invasive biomarker for HCC patient with chronic HBV infection. Diagnostic pathology 2015;10():133. | Duplicate (including Cochrane includes) |
| Dong, Xueyan, He, Hui, Zhang, Weiying, Yu, Daojun, Wang, Xianjun. Combination of serum RASSF1A methylation and AFP is a promising non-invasive biomarker for HCC patient with chronic HBV infection. Diagnostic pathology 2015;10(101251558):133. | Exclude on population: not cirrhosis |
| Dong, Xueyan, Hou, Qiang, Chen, Yueming. Diagnostic Value of the Methylation of Multiple Gene Promoters in Serum in Hepatitis B Virus-Related Hepatocellular Carcinoma. Disease markers 2017;2017():2929381. | Does not meet full eligibility criteria: includes people without cirrhosis |
| Dong, Z. Z., Yao, D. F., Yao, M., Qiu, L. W., Zong, L., Wu, W., et al. Clinical impact of plasma TGF-beta1 and circulating TGF-beta1 mRNA in diagnosis of hepatocellular carcinoma. Hepatobiliary and Pancreatic Diseases International 2008;7(3):288-95. | Does not meet full eligibility criteria: includes people without cirrhosis |
| Dong, Zhi-Zhen, Yao, Deng-Fu, Yao, Deng-Bing, Wu, Xin-Hua, Wu, Wei, Qiu, Li-Wei, et al. Expression and alteration of insulin-like growth factor II-messenger RNA in hepatoma tissues and peripheral blood of patients with hepatocellular carcinoma. World journal of gastroenterology 2005;11(30):4655-60. | Does not meet full eligibility criteria: includes people without cirrhosis |
| Dong, Zhi-Zhen, Yao, Deng-Fu, Yao, Min, Qiu, Li-Wei, Zong, Lei, Wu, Wei, et al. Clinical impact of plasma TGF-beta1 and circulating TGF-beta1 mRNA in diagnosis of hepatocellular carcinoma. Hepatobiliary & pancreatic diseases international : HBPD INT 2008;7(3):288-95. | Does not meet full eligibility criteria: includes people without cirrhosis |
| D'Onofrio, M., Faccioli, N., Zamboni, G., Malago, R., Caffarri, S., Fattovich, G. Focal liver lesions in cirrhosis: value of contrast-enhanced ultrasonography compared with Doppler ultrasound and alpha-fetoprotein levels. La Radiologia medica 2008;113(7):978-91. | Exclude on population: clinical signs and symptoms of HCC |
| dos Santos, D. B., Fernandez, G. J., Silva, L. T., Silva, G. F., Lima, E. O., Galvani, A. F., Pereira, G. L. lncRNAs as Biomarkers of Hepatocellular Carcinoma Risk and Liver Damage in Advanced Chronic Hepatitis C. Current Issues in Molecular Biology 2025;47(5):348. | Does not meet full eligibility criteria: includes people without cirrhosis |
| Dou, C. Y., Fan, Y. C., Cao, C. J., Yang, Y. Sera DNA Methylation of CDH1, DNMT3b and ESR1 Promoters as Biomarker for the Early Diagnosis of Hepatitis B Virus-Related Hepatocellular Carcinoma. Digestive Diseases and Sciences 2016;61(4):1130-38. | Does not meet full eligibility criteria: includes people without cirrhosis |
| Doyle, Deirdre J., O'Malley, Martin E., Jang, Hyun-Jung. Value of the unenhanced phase for detection of hepatocellular carcinomas 3 cm or less when performing multiphase computed tomography in patients with cirrhosis. Journal of computer assisted tomography 2007;31(1):86-92. | Exclude on index test |
| Du, Lin, Wang, Manli, Li, Hui, Li, Na. Identification of CCL20 and LCN2 as Efficient Serological Tools for Detection of Hepatocellular Carcinoma. Disease markers 2022;2022():7758735. | Does not meet full eligibility criteria: includes people without cirrhosis |
| Du, Y., Shi, C., Gao, F., Zhang, M., Wang, L., Zhang, Z., Ming, Y. Value of serum Aldo-keto reductase family 1 member B10(AKR1B10)in diagnosis of hepatocellular carcinoma. Journal of Clinical Hepatology 2025;41(4):684 “ 689. | Foreign language |
| Du, Zhenhua, Liu, Xinyi, Wei, Xiaojun, Luo, Hongbo, Li, Peiyao, Shi, Mengting, et al. Quantitative proteomics identifies a plasma multi-protein model for detection of hepatocellular carcinoma. Scientific reports 2020;10(1):15552. | Exclude on population: healthy controls |
| Duclos, B., Demangeat, J. L., Manil, L., Chamouard, P., Baumann, R., Demangeat, C., et al. Immunoscintigraphic study of hepatocellular carcinoma | Does not meet full eligibility criteria: pre-2005 |

| Reference | Reason for exclusion |
|---|---|
| with monoclonal antibody anti-AFP. Gastroenterologie Clinique et Biologique 1989;13(11):910-15. | |
| Dumas, O., Roget, L., Coppere, H., David, A., Richard, P., Barthelemy, C., Veyret, C. Value of ultrasound-guided fine-needle aspiration biopsy: Report of 206 patients with hepatic lesions and comparison with ultrasound imaging among different clinical presentations. Gastroenterologie Clinique et Biologique 1990;14(1):67-73. | Does not meet full eligibility criteria: pre-2005 |
| Dunn, Colin, Lin, Bo, Rich, Nicole E., Patel, Madhukar S., Gopal, Purva. Correlation of LI-RADS 3 or 4 Observations with Histopathologic Diagnosis in Patients with Cirrhosis. Clinical gastroenterology and hepatology : the official clinical practice journal of the American Gastroenterological Association 2022;():n. pag.. | Exclude on population: clinical signs and symptoms of HCC |
| Durazo, Francisco A, Blatt, Lawrence M, Corey, William G, Lin, Jiing-Huey, Han, Steven, Saab, Sammy, Busuttil, Ronald W. Des-gamma-carboxyprothrombin, alpha-fetoprotein and AFP-L3 in patients with chronic hepatitis, cirrhosis and hepatocellular carcinoma. Journal of gastroenterology and hepatology 2008;23(10):1541-8. | Does not meet full eligibility criteria: includes people without cirrhosis |
| Durazo, Francisco A., Blatt, Lawrence M., Corey, William G., Lin, Jiing-Huey, Han, Steven, Saab, Sammy, Busuttil, Ronald W. Des-gamma-carboxyprothrombin, alpha-fetoprotein and AFP-L3 in patients with chronic hepatitis, cirrhosis and hepatocellular carcinoma. Journal of gastroenterology and hepatology 2008;23(10):1541-48. | Duplicate (including Cochrane includes) |
| Edis, C, Kahler, C, Klotz, W, Herold, M, Feichtinger, H, Konigsreiner, A, et al. A comparison between alpha-fetoprotein and p53 antibodies in the diagnosis of hepatocellular carcinoma. Transplantation proceedings 1998;30(3):780-1. | Does not meet full eligibility criteria: pre-2005 |
| Edoo, Muhammad Ibrahim Alhadi, Chutturghoon, Vikram Kumar, Wusu-Ansah, Gyabaah Kwabena, Zhu, Hai, Zhen, Tao Yang, Xie, Hai Yang. Serum Biomarkers AFP, CEA and CA19-9 Combined Detection for Early Diagnosis of Hepatocellular Carcinoma. Iranian journal of public health 2019;48(2):314-22. | Exclude on target condition: not HCC |
| Edoo, Muhammad Ibrahim Alhadi, Chutturghoon, Vikram Kumar, Wusu-Ansah, Gyabaah Kwabena, Zhu, Hai, Zhen, Tao Yang, Xie, Hai Yang. Serum Biomarkers AFP, CEA and CA19-9 Combined Detection for Early Diagnosis of Hepatocellular Carcinoma. Iranian journal of public health 2019;48(2):314-22. | Does not meet full eligibility criteria: includes people without cirrhosis |
| Effat Saied, Narmin, Elmazny, Gehad Mohsen, El-Helaly, Rania Mostafa, Farag, Raghda El-Sayed, Abd El-Wahab, Khaled, Abo Hashim, Ekbal. Utility of comet assay of DNA damage in the detection of malignant transformation of chronic liver cirrhosis. Scandinavian journal of clinical and laboratory investigation 2023;83(3):145-51. | Does not meet full eligibility criteria: includes people without cirrhosis |
| Egusa, K. & Ide, T. Serum alpha-fetoprotein level in the cases of hepatocellular carcinomas and chronic liver disorders - Association with liver function tests and tumor marker tests on which reference values were determined. IRYO - Japanese Journal of National Medical Services 1985;39(5):430-08. | Does not meet full eligibility criteria: pre-2005 |
| Eilard, M. S. Combination of biomarkers for the detection of hepatocellular carcinoma. Annals of Translational Medicine 2020;8(20):1283. | Exclude on study design: not a 1- or 2-gate test accuracy study |
| Eisa, N. H., Ebrahim, M. A., Ragab, M., Eissa, L. A. Galectin-3 and matrix metalloproteinase-9: Perspective in management of hepatocellular carcinoma. Journal of Oncology Pharmacy Practice 2015;21(5):323-30. | No 2x2 data |
| Eissa, Laila A, Eisa, Nada H, Ebrahim, Mohamed A, Ragab, Maha. Nitric Oxide is a Potential Diagnostic Marker for Hepatocellular Carcinoma. Scientia pharmaceutica 2013;81(3):763-75. | Exclude on population: healthy controls |
| Eissa, Laila A., Eisa, Nada H., Ebrahim, Mohamed A., Ragab, Maha. Nitric Oxide is a Potential Diagnostic Marker for Hepatocellular Carcinoma. Scientia pharmaceutica 2013;81(3):763-75. | Duplicate (including Cochrane includes) |
| Eissa, Mohamed, Awad, Selmy, Barakat, Somaya, Saleh, Ahmed. Serum Golgi protein 73 as a sensitive biomarker for early detection of hepatocellular carcinoma among Egyptian patients with hepatitis C virus-related cirrhosis. Medical journal, Armed Forces India 2021;77(3):331-36. | Exclude on population: not cirrhosis |
| Eissa, S., Swellam, M., El Ahmady, O., Hamdy, H., Youssef, M. Clinical significance of circulating p53 autoantibodies in hepatocellular carcinoma and | Does not meet full eligibility criteria: pre-2005 |

| Reference | Reason for exclusion |
|---|---|
| chronic HCV patients in relation to AFP and ferritin levels. Cancer Molecular Biology 2002;9(1):1773-83. | |
| Ekmen, N. & Akalin, C. Predictive value of protein induced by absence of vitamin K absence or antagonist II, alpha-fetoprotein and gamma-glutamyltransferase/aspartate aminotransferase ratio for the diagnosis of hepatocellular carcinoma in transplantation candidates. European Journal of Gastroenterology and Hepatology 2021;32(2):294-99. | Exclude on population: HCC participants not treatment-naive |
| El Azm, Abdel Raouf Abou, Yousef, Mohamed, Salah, Raafat, Mayah, Wael, Tawfeek, Salwa, Ghorabah, Hussien. Serum anti-P53 antibodies and alpha-fetoprotein in patients with non-B non-C hepatocellular carcinoma. SpringerPlus 2013;2(1):69. | Does not meet full eligibility criteria: includes people without cirrhosis |
| El Gedawy, G., Obada, M., Kelani, A., El-Said, H. Circulating MiRNA-21 and programed cell death (PDCD) 4 gene expression in hepatocellular carcinoma (HCC) in Egyptian patients. Egyptian Journal of Medical Human Genetics 2017;18(2):137-45. | Exclude on population: not cirrhosis |
| El Hawary, A. T., Nabil, F., El Hendawy, R., Mahrous, H. K. A., AbdelHamid, G. A. Serum microRNA-16 as a potential biomarker for HCV-induced hepato-cellular carcinoma in Egyptian patients. The Egyptian journal of immunology 2024;31(2):102 “ 111. | Exclude on index test |
| El Mahdy, Hussein Ahmed, Abdelhamid, Ismail Abdelshafy, Amen, Ahmed Ibrahim, Abdelsameea, Eman. MicroRNA-215 as a Diagnostic Marker in Egyptian Patients with Hepatocellular Carcinoma. Asian Pacific journal of cancer prevention : APJCP 2019;20(9):2723-31. | Does not meet full eligibility criteria: includes people without cirrhosis |
| El Moety A.A.A. Evaluation of nitric oxide as a novel diagnostic marker for hepatocellular carcinoma. Alexandria Journal of Medicine 2011;47(1):31-35. | Exclude on population: not cirrhosis |
| El Moety, A. A. A. Evaluation of nitric oxide as a novel diagnostic marker for hepatocellular carcinoma. Alexandria Journal of Medicine 2011;47(1):31-35. | Duplicate (including Cochrane includes) |
| El Shafie, Mona A., Fawzy, Amal M., Al Monem, E. A., Abbass, Sherif, Zakaria, Doaa M. Golgi Protein 73 (GP73) as a novel serum marker for early detection of hepatocellular carcinoma in egyptian patients. Life Sci. J 2012;9(2):823-30. | Exclude on population: healthy controls |
| El Shazly, Y. M., El Moghny, M. A., Al Swaff, R., Hussein, H. A., Rasmy, H. S. Assessment of serum retinol binding protein 4 in patients with hepatocellular carcinoma. Journal of Clinical and Diagnostic Research 2018;12(10):OC27-31. | Exclude on index test |
| El-Abd, Nevine E., Fawzy, Nahla A., El-Sheikh, Suzan M. Circulating miRNA-122, miRNA-199a, and miRNA-16 as Biomarkers for Early Detection of Hepatocellular Carcinoma in Egyptian Patients with Chronic Hepatitis C Virus Infection. Molecular diagnosis & therapy 2015;19(4):213-20. | Duplicate (including Cochrane includes) |
| El-Abd, Nevine, Fawzy, Amal, Elbaz, Tamer. Evaluation of annexin A2 and as potential biomarkers for hepatocellular carcinoma. Tumour biology : the journal of the International Society for Oncodevelopmental Biology and Medicine 2016;37(1):211-16. | Duplicate (including Cochrane includes) |
| El-Abd, Nevine, Fawzy, Amal, Elbaz, Tamer. Evaluation of annexin A2 and as potential biomarkers for hepatocellular carcinoma. Tumour biology : the journal of the International Society for Oncodevelopmental Biology and Medicine 2016;37(1):211-6. | Exclude on population: healthy controls |
| El-Ahwany, Eman G. E., Mourad, Lobna, Zoheiry, Mona M. K., Abu-Taleb, Hoda, Hassan, Marwa, Atta, Raafat, Hassanien, Moataz. MicroRNA-122a as a non-invasive biomarker for HCV genotype 4-related hepatocellular carcinoma in Egyptian patients. Archives of medical science : AMS 2019;15(6):1454-61. | Exclude on population: healthy controls |
| El-Araby, R. E., Khalifa, M. A., Zoheiry, M. M., Zahran, M. Y., Rady, M. I., Ibrahim, R. A., El-Talkawy, M. D. The interaction between microRNA-152 and DNA methyltransferase-1 as an epigenetic prognostic biomarker in HCV-induced liver cirrhosis and HCC patients. Cancer Gene Therapy 2020;27(6):486-97. | Retracted |
| El-Attar, H. A., Kandil, M. H., El-Kerm, Y. M. Comparison of serum survivin and alpha fetoprotein in Egyptian patients with hepatocellular carcinoma associated with hepatitis C viral infection. Asian Pacific journal of cancer prevention : APJCP 2010;11(4):897-903. | Exclude on population: not cirrhosis |

| Reference | Reason for exclusion |
|---|---|
| El-Bendary, M., Nour, D., Arafa, M. Methylation of tumour suppressor genes RUNX3, RASSF1A and E-Cadherin in HCV-related liver cirrhosis and hepatocellular carcinoma. British Journal of Biomedical Science 2020;77(1):35-40. | Does not meet full eligibility criteria: includes people without cirrhosis |
| El-Daly, Usama M., Saber, Magdy M., Abdellateif, Mona S., Nassar, Hanan R., Namour, Alfred E., Ismail, Yahia M. The Possible Role of Adipokines in HCV Associated Hepatocellular Carcinoma. Asian Pacific journal of cancer prevention : APJCP 2020;21(3):599-609. | Exclude on population: not cirrhosis |
| El-Derany, Marwa O. Polymorphisms in Interleukin 13 Signaling and Interacting Genes Predict Advanced Fibrosis and Hepatocellular Carcinoma Development in Non-Alcoholic Steatohepatitis. Biology 2020;9(4):n. pag.. | Exclude on population: not cirrhosis |
| Elemeery, Moustafa Nouh, Badr, Ahmed Noah, Mohamed, Marwa Anwar. Validation of a serum microRNA panel as biomarkers for early diagnosis of hepatocellular carcinoma post-hepatitis C infection in Egyptian patients. World journal of gastroenterology 2017;23(21):3864-75. | Does not meet full eligibility criteria: includes people without cirrhosis |
| Elemeery, Moustafa Nouh, Mohamed, Marwa Anwar, Madkour, Marwa Ahmed, Shamseya, Mohammed Mohammed, Issa, Noha Mahmoud, Badr, Ahmed Noah, Ghareeb, Doaa Ahmed. MicroRNA signature in patients with hepatocellular carcinoma associated with type 2 diabetes. World journal of gastroenterology 2019;25(42):6322-41. | Genomic biomarker not validated |
| El-Fadle, A. A., Al Husseini, N. F., Al-Kholy, A. F., Al-Said, O., Al-Toukhy, N. Tolemerase reverse transcriptase gene expression as a tumor marker for hepatocellular carcinoma. American Journal of Biochemistry and Biotechnology 2011;7(2):55-62. | Does not meet full eligibility criteria: includes people without cirrhosis |
| Elfert, Ashraf Y., Salem, Amel, Abdelhamid, Amr M., Salama, Ahmad, Sourour, Doaa A., Shaker, Olfat. Implication of miR-122, miR-483, and miR-335 Expression Levels as Potential Signatures in HCV-Related Hepatocellular Carcinoma (HCC) in Egyptian Patients. Frontiers in molecular biosciences 2022;9():864839. | Exclude on population: not cirrhosis |
| El-Folly, Runia Fouad & El-Kabarity, Rania Hamdy. Assessment of the role of interleukin-6 in diagnosis of hepatocellular carcinoma. The Egyptian journal of immunology 2010;17(2):11-22. | Does not meet full eligibility criteria: includes people without cirrhosis |
| El-Garem, Hassan, Ammer, Ayman, Shehab, Hany, Shaker, Olfat, Anwer, Mohammed, El-Akel, Wafaa. Circulating microRNA, miR-122 and miR-221 signature in Egyptian patients with chronic hepatitis C related hepatocellular carcinoma. World journal of hepatology 2014;6(11):818-24. | Exclude on population: not cirrhosis |
| El-Gebaly, Fatma, Abou-Saif, Sabry, Elkadeem, Mahmoud, Helmy, Amal, Abd-Elsalam, Sherief, Yousef, Mohamed, et al. Study of Serum Soluble Programmed Death Ligand 1 as a Prognostic Factor in Hepatocellular Carcinoma in Egyptian Patients. Current cancer drug targets 2019;19(11):896-905. | Exclude on index test |
| Elghoroury, E. A., Abdelghaffar, E. E., Wahby, A. A., Nasr, S. A., Hussein, M. A., Nazim, W. S., et al. Evaluation of Homocysteine and Micro RNA as Diagnostic Markers for Hepatocellular Carcinoma in Virus Hepatitis C Egyptian Patients. Biomedical and Pharmacology Journal 2020;13(3):1145-55. | No 2x2 data |
| Elghoroury, Eman A., Abdelghaffar, Esmat E., Awadallah, Eman, Kamel, Solaf A., Kandil, Dina, Hassan, Eman M., et al. Detection of exosomal miR-18a and miR-222 levels in Egyptian patients with hepatic cirrhosis and hepatocellular carcinoma. International journal of immunopathology and pharmacology 2022;36():3946320221097832. | Exclude on population: healthy controls |
| El-Hamouly, Moamena S., Azzam, Ayman A., Ghanem, Samar E., El-Bassal, Fathia I., Shebl, Nashwa. Circulating microRNA-301 as a promising diagnostic biomarker of hepatitis C virus-related hepatocellular carcinoma. Molecular biology reports 2019;46(6):5759-65. | Does not meet full eligibility criteria: includes people without cirrhosis |
| el-Houseini, Motawa E, Mohammed, Mohammed S, Elshemey, Wael M, Hussein, Tarek D, Desouky, Omar S. Enhanced detection of hepatocellular carcinoma. Cancer control : journal of the Moffitt Cancer Center 2005;12(4):248-53. | Does not meet full eligibility criteria: includes people without cirrhosis |

| Reference | Reason for exclusion |
|---|---|
| el-Houseini, Motawa E., Mohammed, Mohammed S., Elshemey, Wael M., Hussein, Tarek D., Desouky, Omar S. Enhanced detection of hepatocellular carcinoma. Cancer control : journal of the Moffitt Cancer Center 2005;12(4):248-53. | Does not meet full eligibility criteria: includes people without cirrhosis |
| Elias, J. Evaluation of CA 125 as a serum marker of hepatocellular carcinoma. International journal of cancer 1990;46(5):805-07. | No 2x2 data |
| Elkammah, M., Gowily, A., Okda, T. Serum soluble Toll-like receptor 4 and the risk of hepatocellular carcinoma in hepatitis C virus patients. Wspolczesna Onkologia 2021;24(4):216-20. | Exclude on population: not cirrhosis |
| El-Maraghy, Shohda A., Adel, Ola, Zayed, Naglaa, Yosry, Ayman, El-Nahaas, Saeed M. Circulatory miRNA-484, 524, 615 and 628 expression profiling in HCV mediated HCC among Egyptian patients; implications for diagnosis and staging of hepatic cirrhosis and fibrosis. Journal of advanced research 2020;22():57-66. | Does not meet full eligibility criteria: includes people without cirrhosis |
| El-Masry, M. I. Study of role of melanoma-associated antigen D1 (MAGE-D1) in hepatocellular carcinoma. Journal of Investigative Medicine 2025;73(1):35 " 44. | Does not meet full eligibility criteria: includes people without cirrhosis |
| El-Mazny, Ahmed & Sayed, Mohamed. Human telomerase reverse transcriptase messenger RNA (TERT mRNA) as a tumour marker for early detection of hepatocellular carcinoma. Arab journal of gastroenterology : the official publication of the Pan-Arab Association of Gastroenterology 2014;15(2):68-71. | Exclude on population: healthy controls |
| El-Mezayen, Hatem A., El -Kassas, Mohamed, El-Taweel, Fathy M., Metwally, Fateheya M., Ghonaim, Noha A. Diagnostic Performance of Circulating Tumor Cells for Predicting of Hepatocellular Carcinoma in Hepatitis C Virus-High Risk Patients: Role of Liquid Biopsy. Asian Pacific journal of cancer prevention : APJCP 2022;23(7):2541-49. | Continuous test without threshold |
| El-Naidany, Sherin Sobhy, Zid, Ebrahim, Reda, Fatma M., Nada, Ali. Clinical Significance of MiR-130b and MiR-125b as Biomarkers in Hepatocellular Carcinoma. Asian Pacific journal of cancer prevention : APJCP 2022;23(8):2687-93. | Does not meet full eligibility criteria: includes people without cirrhosis |
| El-Saeed, G. K., El-Bassuoni, M. A., El-Saeed, H. H., Gaweesh, I. A., Soliman, M. A. Molecular detection of human telomerase mRNA (hTERT-mRNA) in Egyptian patients with hepatocellular carcinoma. Hepatitis Monthly 2009;9(2):114-21. | Exclude on population: not cirrhosis |
| El-Serag H.B., White D., Tayob N., Alsarraj A., Mori Y., Yamada H. Phase 3 biomarker study for HCC surveillance using AFP, AFP-l3 and DCP: A prospective collection with retrospective blinded evaluation. Hepatology 2017;66(Supplement 1):754A. | CONFERENCE ABSTRACT |
| El-Serag, H. B., Jin, Q., Tayob, N., Salem, E., Luster, M., Alsarraj, A., et al. HES V2.0 outperforms GALAD for detection of HCC: A phase 3 biomarker study in the United States. Hepatology 2024;():n. pag.. | Duplicate (including Cochrane includes) |
| El-Serag, H., Kanwal, F., Ning, J., Powell, H., Khaderi, S., Singal, A. G., et al. Serum biomarker signature is predictive of the risk of hepatocellular cancer in patients with cirrhosis. Gut 2024;73(6):1000 " 1007. | No 2x2 data |
| Elsewify, Wael Abd Elgwad, Hassan, Elham Ahmed, Mekky, Mohamed A., Abd El-Rehim, Abeer Sharaf El-Din, Sayed, Zain El-Abdeen Ahmed, Abdel Malek, Mohamed Omar, ElMelegy, Tarek T. H. Usefulness of Circulating Methylated p16 as a Noninvasive Molecular Biomarker for Hepatitis C-Related Hepatocellular Carcinoma with Normal Serum Alpha-Fetoprotein Levels. International journal of general medicine 2020;13():147-55. | Exclude on population: not cirrhosis |
| El-Shabrawi, A., Bacher, H., Cerwenka, H., Werkgartner, G., El-Shabrawi, Y., Hofler, G. Is the information yielded by detection of circulating HCC cells in peripheral blood of clinical relevance? European Surgery - Acta Chirurgica Austriaca 2002;34(6):359-61. | Exclude on population: clinical signs and symptoms of HCC |
| El-Shal, Amal S., Zidan, Haidy E., Rashad, Nearmeen M. Angiopoietin-like protein 3 and 4 expression 4 and their serum levels in hepatocellular carcinoma. Cytokine 2017;96():75-86. | Does not meet full eligibility criteria: includes people without cirrhosis |
| El-Shayeb, A. F., El-Habachi, N. M., Mansour, A. R. Serum midkine is a more sensitive predictor for hepatocellular carcinoma than Dickkopf-1 and alpha-L- | Duplicate (including Cochrane includes) |

| Reference | Reason for exclusion |
|---|---|
| fucosidase in cirrhotic HCV patients. Medicine (United States) 2021;100(17):E25112. | |
| El-Shazly, S. F., Eid, M. A., El-Sourogy, H. A., Attia, G. F. Evaluation of serum DNA integrity as a screening and prognostic tool in patients with hepatitis C virus-related hepatocellular carcinoma. International Journal of Biological Markers 2010;25(2):79-86. | Exclude on population: not cirrhosis |
| El-Shenawy, R., Moustafa, R. I., Helmy, N. M., El-Abd, Y. S., Tabll, A. A., Elesnawy, Y. K. Peripheral immune signatures associated with the risk of hepatocarcinogenesis in cirrhotic Egyptian HCV patients before and after treatment with direct-acting antivirals. Virology Journal 2024;21(1):293. | Meets all eligibility criteria but data are untrustworthy |
| El-Shenawy, Soha Z., El-Sabawy, Maha M., Abd El-Razik, Eman. Detection of serum KL-6 as a tumor marker in hepatocellular carcinoma. Life Sci. J 2012;9(1):667-73. | Does not meet full eligibility criteria: includes people without cirrhosis |
| El-Sherif, Wafaa T, Makhlouf, Nahed A, El-Gendi, Salwa S, Hassan, Howayda I. Evaluation of transforming growth factor alpha and vascular endothelial growth factor in diagnosis of hepatocellular carcinoma. The Egyptian journal of immunology 2012;19(2):53-65. | Exclude on population: healthy controls |
| Eltaher, S M, El-Gil, R, Fouad, N, Mitwali, R. Evaluation of serum levels and significance of soluble CD40 ligand in screening patients with hepatitis C virus-related hepatocellular carcinoma. Eastern Mediterranean health journal = La revue de sante de la Mediterranee orientale = al-Majallah al-sihhiyah li-sharq al-mutawassit 2016;22(8):603-10. | Exclude on population: healthy controls |
| Eltaher, S. M., El-Gil, R., Fouad, N., Mitwali, R. Evaluation of serum levels and significance of soluble CD40 ligand in screening patients with hepatitis C virus-related hepatocellular carcinoma. Eastern Mediterranean health journal = La revue de sante de la Mediterranee orientale = al-Majallah al-sihhiyah li-sharq al-mutawassit 2016;22(8):603-10. | Exclude on population: healthy controls |
| El-Tawdi, A. H. F., Matboli, M., Shehata, H. H., Tash, F., El-Khazragy, N., Azazy, A. E. S. M. Evaluation of Circulatory RNA-Based Biomarker Panel in Hepatocellular Carcinoma. Molecular Diagnosis and Therapy 2016;20(3):265-77. | Exclude on population: not cirrhosis |
| El-Tayeh, Sarmad F, Hussein, Tarek D, El-Houseini, Motawa E, Amer, Mahmoud A, El-Sherbini, Mamdooh. Serological biomarkers of hepatocellular carcinoma in Egyptian patients. Disease markers 2012;32(4):255-63. | Exclude on population: not cirrhosis |
| El-Tayeh, Sarmad F., Hussein, Tarek D., El-Houseini, Motawa E., Amer, Mahmoud A., El-Sherbini, Mamdooh. Serological biomarkers of hepatocellular carcinoma in Egyptian patients. Disease markers 2012;32(4):255-63. | Duplicate (including Cochrane includes) |
| ElZefzafy, Wafaa Mohammed, Hussien, Magda, Mohmmed, Zakia Abu Zahab. The diagnostic value of golgi protien-73 and DICKKOPF-1 in hepatocellular carcinoma. Journal of immunoassay & immunochemistry 2021;42(2):174-87. | Exclude on population: healthy controls |
| El-Zefzafy, Wafaa, Eltokhy, Hanan, Mohamed, Nagwa Abd El-Ghaffar. Significance of Serum Cytokeratin-18 in Prediction of Hepatocellular Carcinoma in Chronic Hepatitis C Infected Egyptian Patients. Open access Macedonian journal of medical sciences 2015;3(1):117-23. | Exclude on population: not cirrhosis |
| El-Zefzafy, Wafaa, Eltokhy, Hanan, Mohamed, Nagwa Abd El-Ghaffar. Significance of Serum Cytokeratin-18 in Prediction of Hepatocellular Carcinoma in Chronic Hepatitis C Infected Egyptian Patients. Open access Macedonian journal of medical sciences 2015;3(1):117-23. | Duplicate (including Cochrane includes) |
| Elzeneini, A. M. & Abdelmohsen, M. A. Multi-phasic magnetic resonance imaging of hemodynamic interchanges in hepatocarcinogenesis. Egyptian Journal of Radiology and Nuclear Medicine 2023;54(1):181. | No 2x2 data |
| Endo, Y., Tsuchida, Y., Miyazaki, J. [Analysis of lectin-affinity of alpha fetoprotein-diagnostic approach]. Gan to kagaku ryoho. Cancer & chemotherapy 1983;10(2 Pt 2):636-41. | Does not meet full eligibility criteria: pre-2005 |
| Enjoji, M., Nakamuta, M., Arimura, E., Morizono, S., Kuniyoshi, M., Fukushima, M., Kotoh, K. Clinical significance of urinary N1,N12-diacetylspermine levels in patients with hepatocellular carcinoma. The International journal of biological markers 2004;19(4):322-27. | No 2x2 data |

| Reference | Reason for exclusion |
|---|---|
| Enooku, Kenichiro, Nakagawa, Hayato, Soroida, Yoko, Ohkawa, Ryunosuke, Kageyama, Yuko, Uranbileg, Baasanjav, et al. Increased serum mitochondrial creatine kinase activity as a risk for hepatocarcinogenesis in chronic hepatitis C patients. International journal of cancer 2014;135(4):871-79. | Exclude on population: not cirrhosis |
| Erdal, H., Utku, O. G., Karatay, E., Celik, B., Elbeg, S. Combination of DKK1 and AFP improves diagnostic accuracy of hepatocellular carcinoma compared with either marker alone. Turkish Journal of Gastroenterology 2016;27(4):375-81. | Duplicate (including Cochrane includes) |
| Erdal, Harun, Gul Utku, Ozlem, Karatay, Eylem, Celik, Bulent, Elbeg, Sehri. Combination of DKK1 and AFP improves diagnostic accuracy of hepatocellular carcinoma compared with either marker alone. The Turkish journal of gastroenterology : the official journal of Turkish Society of Gastroenterology 2016;27(4):375-81. | Does not meet full eligibility criteria: includes people without cirrhosis |
| Ertle, Judith M, Heider, Dominik, Wichert, Marc, Keller, Benedikt, Kueper, Robert, Hilgard, Philip, Gerken, Guido. A combination of alpha-fetoprotein and des-gamma-carboxy prothrombin is superior in detection of hepatocellular carcinoma. Digestion 2013;87(2):121-31. | Does not meet full eligibility criteria: includes people without cirrhosis |
| Ertle, Judith M., Heider, Dominik, Wichert, Marc, Keller, Benedikt, Kueper, Robert, Hilgard, Philip, Gerken, Guido. A combination of alpha-fetoprotein and des-gamma-carboxy prothrombin is superior in detection of hepatocellular carcinoma. Digestion 2013;87(2):121-31. | Duplicate (including Cochrane includes) |
| Ette, Akpakip I, Ndububa, Dennis A, Adekanle, Olusegun. Utility of serum des-gamma-carboxyprothrombin in the diagnosis of hepatocellular carcinoma among Nigerians, a case-control study. BMC gastroenterology 2015;15(100968547):113. | Exclude on population: not cirrhosis |
| Ette, Akpakip I., Ndububa, Dennis A., Adekanle, Olusegun. Utility of serum des-gamma-carboxyprothrombin in the diagnosis of hepatocellular carcinoma among Nigerians, a case-control study. BMC gastroenterology 2015;15():113. | Duplicate (including Cochrane includes) |
| Eun, Jung Woo, Jang, Jeong Won, Yang, Hee Doo, Kim, Jooyoung, Kim, Sang Yean, Na, Min Jeong, et al. Serum Proteins, HMMR, NXPH4, PITX1 and THBS4; A Panel of Biomarkers for Early Diagnosis of Hepatocellular Carcinoma. Journal of clinical medicine 2022;11(8):n. pag.. | Does not meet full eligibility criteria: includes people without cirrhosis |
| Ezzat, W. M., Amr, K. S., Elhosary, Y. A., Hegazy, A. E., Fahim, H. H., Eltaweel, N. H. Detection of DNA methylated microRNAs in hepatocellular carcinoma. Gene 2019;702():153-57. | Does not meet full eligibility criteria: includes people without cirrhosis |
| Ezzat, Wafaa M., Amr, Khalda Said, Raouf, Haiam Abdel, Elhosary, Yasser A., Hegazy, Abdelfattah E., Fahim, Hoda H. Relationship Between Serum microRNA155 and Telomerase Expression in Hepatocellular Carcinoma. Archives of medical research 2016;47(5):349-55. | Exclude on population: healthy controls |
| Ezzikouri, Sayeh, Kimura, Kiminori, Sunagozaka, Hajime, Kaneko, Shuichi, Inoue, Kazuaki, Nishimura, Tomohiro, et al. Serum DHCR24 Auto-antibody as a new Biomarker for Progression of Hepatitis C. EBioMedicine 2015;2(6):604-12. | Does not meet full eligibility criteria: includes people without cirrhosis |
| Ezzikouri, Sayeh, Kimura, Kiminori, Sunagozaka, Hajime, Kaneko, Shuichi, Inoue, Kazuaki, Nishimura, Tomohiro, et al. Serum DHCR24 Auto-antibody as a new Biomarker for Progression of Hepatitis C. EBioMedicine 2015;2(6):604-12. | Duplicate (including Cochrane includes) |
| Fabris, C, Basso, D A, Leandro, G, Meggiato, T, Elba, S, Panozzo, M P, et al. Serum CA 19-9 and alpha-fetoprotein levels in primary hepatocellular carcinoma and liver cirrhosis. Cancer 1991;68(8):1795-8. | Does not meet full eligibility criteria: pre-2005 |
| Fabris, C., Basso, D. A., Leandro, G., Meggiato, T., Elba, S., Panozzo, M. P., et al. Serum CA 19-9 and alpha-fetoprotein levels in primary hepatocellular carcinoma and liver cirrhosis. Cancer 1991;68(8):1795-98. | Does not meet full eligibility criteria: pre-2005 |
| Fahim, Amin, Qureshi, Aneela, Farsi Kazi, Salman Ahmed, Kazi, Navaid. Expression of miR-20a: A serum biomarker in the diagnostic approach for hepatocellular carcinoma. JPMA. The Journal of the Pakistan Medical Association 2019;69(1):29-33. | Exclude on population: not cirrhosis |
| Fan, G. R., Xiong, J. H., Lin, H. Y., Li, L., Jiang, S. Y. Related clinical pathologic correlation analysis of glypican-3 expression in hepatocellular carcinoma. Cancer Research and Clinic 2013;25(10):685. | Foreign language |

| Reference | Reason for exclusion |
|---|---|
| Fan, R., Huang, W., Xiao, S. W., Luo, B., He, S. J., Luo, G. R. OY-TES-1 expression and serum immunoreactivity in hepatocellular carcinoma. World Chinese Journal of Digestology 2009;17(32):3307-12. | Foreign language |
| Fan, Rong, Chen, Lei, Zhao, Siru, Yang, Hao, Li, Zhengmao, Qian, Yunsong, et al. Novel, high accuracy models for hepatocellular carcinoma prediction based on longitudinal data and cell-free DNA signatures. Journal of hepatology 2023;79(4):933-44. | Exclude on study design: not a 1- or 2-gate test accuracy study |
| Fan, Yu-Chen, Zhang, Yuan-Yuan, Sun, Yan-Yan, Wang, Na, Xiao, Xiao-Yan. Altered expression of A20 gene in peripheral blood mononuclear cells is associated with the progression of chronic hepatitis B virus infection. Oncotarget 2016;7(42):68821-32. | No 2x2 data |
| Fan, Z. J., Liu, S., Zhang, L., Tian, Y. Q. Value of combined detection of serum vitronectin, alpha-1-B glycoprotein, antithrombin-III, and alpha fetoprotein for diagnosis of early hepatocellular carcinoma. World Chinese Journal of Digestology 2018;26(14):842-48. | Foreign language |
| Fang, Meng, Zhao, Yun-Peng, Zhou, Fei-Guo, Lu, Lun-Gen, Qi, Peng, Wang, Hao, et al. N-glycan based models improve diagnostic efficacies in hepatitis B virus-related hepatocellular carcinoma. International journal of cancer 2010;127(1):148-59. | Exclude on population: not cirrhosis |
| Fang, Meng, Zhao, Yun-Peng, Zhou, Fei-Guo, Lu, Lun-Gen, Qi, Peng, Wang, Hao, et al. N-glycan based models improve diagnostic efficacies in hepatitis B virus-related hepatocellular carcinoma. International journal of cancer 2010;127(1):148-59. | Duplicate (including Cochrane includes) |
| Fang, Q. Q., Ni, R. Z., Xiao, M. B., Jiang, F. [Serum and tissue expressions of galectin-3 in hepatocellular carcinoma and the clinical significances]. Zhonghua gan zang bing za zhi = Zhonghua ganzangbing zazhi = Chinese journal of hepatology 2011;19(7):527-31. | Foreign language |
| Fang, Qingqing, Chen, Wei, Jian, Yourong, Li, Yu, Lian, Wei, Wan, Hongyu, et al. Serum Expression Level of MicroRNA-122 and Its Significance in Patients with Hepatitis B Virus Infection. Journal of healthcare engineering 2022;2022():8430276. | No 2x2 data |
| Fang, Yujia, Yan, Dong, Wang, Lixin, Zhang, Jie. Circulating microRNAs (miR-16, miR-22, miR-122) expression and early diagnosis of hepatocellular carcinoma. Journal of clinical laboratory analysis 2022;36(7):e24541. | Exclude on population: not cirrhosis |
| Fang, Zhong-Liao, Sabin, Caroline A., Dong, Bai-Qing, Ge, Lian-Ying, Wei, Shao-Chao, Chen, Qin-Yan, et al. HBV A1762T, G1764A mutations are a valuable biomarker for identifying a subset of male HBsAg carriers at extremely high risk of hepatocellular carcinoma: a prospective study. The American journal of gastroenterology 2008;103(9):2254-62. | Exclude on population: not cirrhosis |
| Fang-Xin, Z. Role of determination of multiple tumor markers in the diagnosis of liver cancer in patient with negative or low serum AFP. Chinese Journal of Clinical Oncology 1994;21(1):50-52. | Does not meet full eligibility criteria: pre-2005 |
| Fani, A., Fani, I., Eshrati, B., Samadian, P., Fani, P., Gorishi, Y. Screening for hepatocellular carcinoma in chronic carriers of hepatitis B and C in Markazi province, Iran. Hepatitis Monthly 2007;7(3):149-52. | Exclude on population: not cirrhosis |
| Fani, A., Fani, I., Eshratie, B., Samadian, P., Fani, P. Screening for hepatocellular carcinoma in hepatitis B and C chronic carriers in Iran. Indian Journal of Gastroenterology 2007;26(6):297-98. | Exclude on population: not cirrhosis |
| Farag, R. M. M. A., AlAyobi, D., Alsaleh, K. A., Kwon, H. J., A, E. L-Ansary. Influence of Glypican-3 as anewly diagnostic biomarker in earlydetection of hepatocellular carcinoma among Saudi patients. Biomedical and Pharmacology Journal 2018;11(4):1789-96. | Does not meet full eligibility criteria: includes people without cirrhosis |
| Farag, Randa Mohamed Ahmed, Al Ayobi, Dujana, Alsaleh, Khalid A., Kwon, Hye-Joo, El-Ansary, Afaf. Studying the Impact of Golgi Protein 73 Serving as a Candidate Biomarker in Early Diagnosis for Hepatocellular Carcinoma among Saudi Patients. Asian Pacific journal of cancer prevention : APJCP 2019;20(1):215-20. | Exclude on population: healthy controls |
| Farinati, F., Annoni, G., Donato, M. F., Nardelli, P., Bertozzo, A., De Maria, N., et al. Diagnostic and prognostic value of the determination of the | Does not meet full eligibility criteria: pre-2005 |

| Reference | Reason for exclusion |
|---|---|
| aminopropeptide of type III procollagen in patients with primary liver cancer. Journal of gastroenterology and hepatology 1990;5(6):633-38. | |
| Fathy.Elmougy, F. A., Mohamed, R. A., Hassan, M. M., Elsheikh, S. M., Marzban, R. N., Ahmed, F. E. M. Study of serum microRNA19a and microRNA223 as potential biomarkers for early diagnosis of hepatitis C virus-related hepatocellular carcinoma. Gene Reports 2019;15():100398. | Exclude on population: not cirrhosis |
| Fawzy Montaser, Mohamed & Amin Sakr, Mohamed. Alpha-L-fucosidase as a tumour marker of hepatocellular carcinoma. Arab journal of gastroenterology : the official publication of the Pan-Arab Association of Gastroenterology 2012;13(1):9-13. | Exclude on population: not cirrhosis |
| Fawzy Montaser, Mohamed & Amin Sakr, Mohamed. Alpha-L-fucosidase as a tumour marker of hepatocellular carcinoma. Arab journal of gastroenterology : the official publication of the Pan-Arab Association of Gastroenterology 2012;13(1):9-13. | Duplicate (including Cochrane includes) |
| Fawzy, A., Fawzy, N., El-Sayed Zaher, A., Gomaa, A. I., Hashim, M., Abdallah, A. R., Moawad, M. Expression of SSX-1 and SSX-5 genes in the peripheral blood of patients with hepatocellular carcinoma. Egyptian Journal of Medical Human Genetics 2014;15(1):45-51. | Does not meet full eligibility criteria: includes people without cirrhosis |
| Fei, X., Xueqin, Z., Xiaofen, M., Jian, L. Comparison of the diagnostic performance in the hepatocellular carcinoma with cirrhosis between the 2017 and 2018 versions of LI-RADS based on Gd-EOB-DTPA enhanced MRI. Chinese Journal of Radiology (China) 2022;56(3):286-92. | Exclude on population: clinical signs and symptoms of HCC |
| Felgendreff, Philipp, Raschzok, Nathanael, Kunze, Kerstin, Leder, Annekatrin, Lippert, Steffen, Klunk, Sergej, et al. Tissue-based miRNA mapping in alcoholic liver cirrhosis: different profiles in cirrhosis with or without hepatocellular carcinoma. Biomarkers : biochemical indicators of exposure, response, and susceptibility to chemicals 2020;25(1):62-68. | Exclude on population: tissue samples |
| Feng, Honglei, Li, Bole, Li, Ze, Wei, Qian. PIVKA-II serves as a potential biomarker that complements AFP for the diagnosis of hepatocellular carcinoma. BMC cancer 2021;21(1):401. | Exclude on population: not cirrhosis |
| Feng, Ju-Tao, Liu, Yin-Kun, Song, Hai-Yan, Dai, Zhi, Qin, Lun-Xiu, Almofti, Mohamad Radwan, et al. Heat-shock protein 27: a potential biomarker for hepatocellular carcinoma identified by serum proteome analysis. Proteomics 2005;5(17):4581-88. | Exclude on population: not cirrhosis |
| Feng, L. H., Wei, L., Hu, B., Li, Q., Yin, Q., Su, T., et al. Evaluation of serum ESPL1 as a biomarker for early diagnosis of HBV-related hepatocellular carcinoma. Frontiers in Oncology 2025;15():1574317. | Does not meet full eligibility criteria: includes people without cirrhosis |
| Feng, Xiaobin, Song, Peipei, Bie, Ping, Jiang, Peng, Ma, Kuansheng, Li, Xiaowu, et al. Des-gamma-Carboxyprothrombin Plasma Level in Diagnosis of Hepatocellular Carcinoma in a Chinese Population Undergoing Surgery. Medical science monitor : international medical journal of experimental and clinical research 2016;22():1663-72. | Exclude on population: not cirrhosis |
| Feng, Xiaobin, Song, Peipei, Bie, Ping, Jiang, Peng, Ma, Kuansheng, Li, Xiaowu, et al. Des-gamma-Carboxyprothrombin Plasma Level in Diagnosis of Hepatocellular Carcinoma in a Chinese Population Undergoing Surgery. Medical science monitor : international medical journal of experimental and clinical research 2016;22(dxw, 9609063):1663-72. | Exclude on population: not cirrhosis |
| Fernandes-Ferreira, Rafael, Tenani, Graciele Domitila, Pinhel, Marcela Augusta de Souza, Abrantes, Ana Margarida Coelho, Botelho, Maria Filomena Rabaca Roque, Silva, Rita de Cassia Martins Alves da, Souza, Doroteia Rossi Silva. GENES EXPRESSION AND SERUM BIOMARKERS FOR DIAGNOSIS OF HEPATOCELLULAR CARCINOMA, CIRRHOSIS AND HEPATITIS C. Arquivos de gastroenterologia 2022;59(3):394-401. | Does not meet full eligibility criteria: includes people without cirrhosis |
| Ferrin, Gustavo, Ranchal, Isidora, Llamoza, Camilo, Rodriguez-Peralvarez, Manuel L., Romero-Ruiz, Antonio, Aguilar-Melero, Patricia, et al. Identification of candidate biomarkers for hepatocellular carcinoma in plasma of HCV-infected cirrhotic patients by 2-D DIGE. Liver international : official journal of the International Association for the Study of the Liver 2014;34(3):438-46. | Exclude on index test |
| Ferrin, Gustavo, Rodriguez-Peralvarez, Manuel, Aguilar-Melero, Patricia, Ranchal, Isidora, Llamoza, Camilo, Linares, Clara I., et al. Plasma protein | Exclude on index test |

| Reference | Reason for exclusion |
|---|---|
| biomarkers of hepatocellular carcinoma in HCV-infected alcoholic patients with cirrhosis. PloS one 2015;10(3):e0118527. | |
| Fiduzi, Federico I. F., Willemssen, Francois E. J. A., de Braak, Celine van, de Lussanet de la Sabloniere, Quido G., Ijzermans, Jan N. M., Bos, Daniel, de Man, Robert A. Evaluation of Hepatocellular Carcinoma Surveillance with Contrast-enhanced MRI in a High-Risk Western European Cohort. Current problems in diagnostic radiology 2024;53(6):709â€“716. | Does not meet full eligibility criteria: includes people without cirrhosis |
| Fimmel, C. J. Golgi protein 73 as a biomarker of hepatocellular cancer: Development of a quantitative serum assay and expression studies in hepatic and extrahepatic malignancies. Hepatology 2009;49(5):1421-23. | Exclude on study design: not a 1- or 2-gate test accuracy study |
| Foda, Z. H., Annapragada, A. V., Boyapati, K., Bruhm, D. C., Vulpescu, N. A., Medina, J. E., et al. Detecting Liver Cancer Using Cell-Free DNA Fragmentomes. Cancer Discovery 2023;13(3):616 “ 631. | Does not meet full eligibility criteria: includes people without cirrhosis |
| Foda, Zachariah H., Annapragada, Akshaya V., Boyapati, Kavya, Bruhm, Daniel C., Vulpescu, Nicholas A., Medina, Jamie E., et al. Detecting Liver Cancer Using Cell-Free DNA Fragmentomes. Cancer discovery 2023;13(3):616-31. | No 2x2 data |
| Fornari, Francesca, Ferracin, Manuela, Trere, Davide, Milazzo, Maddalena, Marinelli, Sara, Galassi, Marzia, et al. Circulating microRNAs, miR-939, miR-595, miR-519d and miR-494, Identify Cirrhotic Patients with HCC. PloS one 2015;10(10):e0141448. | No 2x2 data |
| Forner, A. Non-invasive diagnostic criteria for hepatocellular carcinoma: The value of contrast washout at imaging and the death of alpha-fetoprotein. Liver International 2011;31(10):1419-21. | Exclude on study design: not a 1- or 2-gate test accuracy study |
| Fouad, S. A., Elsaaid, N. H., Mohamed, N. A. Diagnostic value of serum level of soluble tumor necrosis factor receptor IIalpha in Egyptian patients with chronic hepatitis C virus infection and hepatocellular carcinoma. Hepatitis Monthly 2014;14(9):n. pag.. | Exclude on index test |
| Fouad, Shawky Abdelhamid, Mohamed, Nagwa Abdel Ghaffar, Fawzy, Mary Wadie. Plasma Osteopontin Level in Chronic Liver Disease and Hepatocellular Carcinoma. Hepatitis monthly 2015;15(9):e30753. | Exclude on population: not cirrhosis |
| Fouad, Yasser M., Mohamed, Hala I., Kamal, Enas M. Clinical significance and diagnostic value of serum dickkopf-1 in patients with hepatocellular carcinoma. Scandinavian journal of gastroenterology 2016;51(9):1133-37. | Exclude on population: not cirrhosis |
| Fowler, Kathryn J., Karimova, E. Jane, Arauz, Anthony R., Saad, Nael E., Brunt, Elizabeth M., Chapman, William C. Validation of organ procurement and transplant network (OPTN)/united network for organ sharing (UNOS) criteria for imaging diagnosis of hepatocellular carcinoma. Transplantation 2013;95(12):1506-11. | Exclude on population: not cirrhosis |
| Fracanzani, A. L., Burdick, L., Borzio, M., Roncalli, M., Bonelli, N., Borzio, F., et al. Contrast-enhanced Doppler ultrasonography in the diagnosis of hepatocellular carcinoma and premalignant lesions in patients with cirrhosis. Hepatology (Baltimore, Md.) 2001;34(6):1109-12. | Exclude on population: clinical signs and symptoms of HCC |
| Frey, Rahel Susanne & Boldanova, Tujana. Ultrasound surveillance for hepatocellular carcinoma: real-life performance in a hepatology outpatient clinic. Swiss medical weekly 2015;145():w14200. | Exclude on study design: not a 1- or 2-gate test accuracy study |
| Frundt, Thorben, Krause, Linda, Hussey, Elaine, Steinbach, Bettina, Kohler, Daniel, von Felden, Johann, et al. Diagnostic and Prognostic Value of miR-16, miR-146a, miR-192 and miR-221 in Exosomes of Hepatocellular Carcinoma and Liver Cirrhosis Patients. Cancers 2021;13(10):n. pag.. | Exclude on population: HCC participants not treatment-naive |
| Fu, Liyun, Yao, Ting, Chen, Qingqing, Mo, Xiaoyan, Hu, Yaoren. Screening differential circular RNA expression profiles reveals hsa_circ_0004018 is associated with hepatocellular carcinoma. Oncotarget 2017;8(35):58405-16. | Exclude on population: tissue samples |
| Fu, S., Boers, R. G., Boers, J. B., van der Meeren, P. E., Helmijr, J., de Weerd, V., et al. Genome-Wide Methylation Sequencing to Identify DNA Methylation Markers for Early-stage Hepatocellular Carcinoma in Liver and Blood. Journal of Experimental and Clinical Cancer Research 2025;44(1):144. | No 2x2 data |
| Fu, Yan, Xu, Xiao, Huang, Dongsheng, Cui, Dawei, Liu, Lisheng, Liu, Junwei, et al. Plasma Heat Shock Protein 90alpha as a Biomarker for the Diagnosis of | Exclude on population: not cirrhosis |

| Reference | Reason for exclusion |
|---|---|
| Liver Cancer: An Official, Large-scale, and Multicenter Clinical Trial. EBioMedicine 2017;24():56-63. | |
| Fujii, T, Horie, Y, Ikuta, Y, Nishimuki, E, Murawaki, Y, Suou, T. Clinical evaluation of a monoclonal antibody to serum KM01 for the diagnosis of hepatocellular carcinoma. Clinica chimica acta; international journal of clinical chemistry 1995;236(1):71-9. | Does not meet full eligibility criteria: pre-2005 |
| Fujii, T., Horie, Y., Ikuta, Y., Nishimuki, E., Murawaki, Y., Suou, T. Clinical evaluation of a monoclonal antibody to serum KM01 for the diagnosis of hepatocellular carcinoma. Clinica chimica acta; international journal of clinical chemistry 1995;236(1):71-79. | Does not meet full eligibility criteria: pre-2005 |
| Fujiwara, N., Marsh, T. L., Marquez, C. A., Raman, I., Li, Q. Z., Parikh, N. D., et al. PROGNOSTIC LIVER SECRETOME SIGNATURE AND ALPHA-FETOPROTEIN (PLSEC-AFP) PREDICTS LONG-TERM HEPATOCELLULAR CARCINOMA RISK IN PATIENTS WITH CIRRHOSIS: A NATIONWIDE PHASE 3 BIOMARKER STUDY IN THE U.S. Gastroenterology 2022;162(7 Supplement):S-1140. | CONFERENCE ABSTRACT |
| Fujiyama, S., Izuno, K., Gohshi, K., Shibata, J. Clinical usefulness of des-gamma-carboxy prothrombin assay in early diagnosis of hepatocellular carcinoma. Digestive diseases and sciences 1991;36(12):1787-92. | Does not meet full eligibility criteria: includes people without cirrhosis |
| Fujiyama, S., Izuno, K., Yamasaki, K., Sato, T. Determination of optimum cutoff levels of plasma des-gamma-carboxy prothrombin and serum alpha-fetoprotein for the diagnosis of hepatocellular carcinoma using receiver operating characteristic curves. Tumour biology : the journal of the International Society for Oncodevelopmental Biology and Medicine 1992;13(5-6):316-23. | Does not meet full eligibility criteria: pre-2005 |
| Fujiyama, S., Morishita, T., Hashiguchi, O. Plasma abnormal prothrombin (des-gamma-carboxy prothrombin) as a marker of hepatocellular carcinoma. Cancer 1988;61(8):1621-28. | Does not meet full eligibility criteria: includes people without cirrhosis |
| Fujiyama, S., Morishita, T., Sagara, K., Sato, T., Motohara, K. Clinical evaluation of plasma abnormal prothrombin (PIVKA-II) in patients with hepatocellular carcinoma. Hepato-gastroenterology 1986;33(5):201-05. | Does not meet full eligibility criteria: pre-2005 |
| Fujiyama, S., Tsude, K., Sakai, M. 5'-Nucleotide phosphodiesterase isozyme-V in hepatocellular carcinoma and other liver diseases. Hepato-gastroenterology 1990;37(5):469-73. | Does not meet full eligibility criteria: pre-2005 |
| Fujiyama, S., Tsude, K., Sakai, M. 5'Nucleotide phosphodiesterase isozyme-V in hepatocellular carcinoma and other liver diseases. Hepato-Gastroenterology 1990;37(5):469-73. | Does not meet full eligibility criteria: pre-2005 |
| Fye, Haddy K. S., Wright-Drakesmith, Cynthia, Kramer, Holger B., Camey, Suzi, Nogueira da Costa, Andre, Jeng, Adam, et al. Protein profiling in hepatocellular carcinoma by label-free quantitative proteomics in two west African populations. PloS one 2013;8(7):e68381. | Does not meet full eligibility criteria: includes people without cirrhosis |
| Gaber, Dalia A., Shaker, Olfat, Younis, Alaa Tarek. LncRNA HULC and miR-122 Expression Pattern in HCC-Related HCV Egyptian Patients. Genes 2022;13(9):n. pag.. | Exclude on population: not cirrhosis |
| Gad, Amal, Tanaka, Eiji, Matsumoto, Akihiro, Wahab, Moushira Abd-el, Serwah, Abd el-Hamid, Attia, Fawzy, et al. Assessment of KL-6 as a tumor marker in patients with hepatocellular carcinoma. World journal of gastroenterology 2005;11(42):6607-12. | Exclude on population: not cirrhosis |
| Gad, Amal, Tanaka, Eiji, Matsumoto, Akihiro, Wahab, Moushira Abd-el, Serwah, Abd el-Hamid, Attia, Fawzy, et al. Assessment of KL-6 as a tumor marker in patients with hepatocellular carcinoma. World journal of gastroenterology 2005;11(42):6607-12. | Exclude on population: not cirrhosis |
| Gamal, M., Moheyeldin, K., Wagdy, M., Aposhady, N. Assessment of serum Mac-2 binding protein glycosylation isomer as a potential marker for hepatocellular carcinoma in cirrhotic hepatitis C patients. Clinical and Experimental Hepatology 2024;10(2):90 “ 97. | Exclude on index test |
| Gambarin-Gelwan, M, Wolf, D C, Shapiro, R, Schwartz, M E. Sensitivity of commonly available screening tests in detecting hepatocellular carcinoma in cirrhotic patients undergoing liver transplantation. The American journal of gastroenterology 2000;95(6):1535-8. | Does not meet full eligibility criteria: pre-2005 |

| Reference | Reason for exclusion |
|---|---|
| Gambarin-Gelwan, M., Wolf, D. C., Shapiro, R., Schwartz, M. E. Sensitivity of commonly available screening tests in detecting hepatocellular carcinoma in cirrhotic patients undergoing liver transplantation. The American journal of gastroenterology 2000;95(6):1535-38. | Does not meet full eligibility criteria: pre-2005 |
| Gandolfi, L., Solmi, L., Bertoni, F., Muratori, R. Small hepatocellular carcinoma: an Italian experience. Hepato-gastroenterology 1987;34(3):100-02. | Does not meet full eligibility criteria: pre-2005 |
| Gao, De-Yong, Ling, Yun, Lou, Xiao-Li, Wang, Ying-Ying. GTSF1 gene may serve as a novel potential diagnostic biomarker for liver cancer. Oncology letters 2018;15(3):3133-40. | Exclude on population: not cirrhosis |
| Gao, Dong-mei, Sun, Lu, Guo, Kun, Li, Yan, Liu, Yin-kun. [Applicability of the multiplex quantitative antibody array system for early diagnosis of hepatocellular carcinoma]. Zhonghua gan zang bing za zhi = Zhonghua ganzangbing zazhi = Chinese journal of hepatology 2012;20(10):785-88. | Foreign language |
| Gao, Guosheng, Dong, Feibo, Xu, Xiaozhen, Hu, Airong. Diagnostic value of serum Golgi protein 73 for HBV-related primary hepatic carcinoma. International journal of clinical and experimental pathology 2015;8(9):11379-85. | Exclude on population: not cirrhosis |
| Gao, H., Li, X., Wang, J., Qiao, F., Gao, F. Clinical application of combined detection of serum Cys-C,5'-NT and AFP in hepatocellular carcinoma. Cancer Research and Clinic 2014;26(11):753-56. | Foreign language |
| Gao, H., Zhao, Q., Wang, J., Gao, F., Qiao, F. Application of serum alpha-fetoprotein, alpha-L-fucosidase, 5'-nucleotidase and gamma-glutamyltransferase in early diagnosis of hepatocellular carcinoma. Cancer Research and Clinic 2016;28(8):519-27. | Foreign language |
| Gao, Rong, Cheng, Jianhua, Fan, Chunlei, Shi, Xiaofeng, Cao, Yuan, Sun, Bo, et al. Serum Metabolomics to Identify the Liver Disease-Specific Biomarkers for the Progression of Hepatitis to Hepatocellular Carcinoma. Scientific reports 2015;5():18175. | Exclude on index test |
| Gao, Shoubao, Xu, Xiaohong, Wang, Yue, Zhang, Wei. Diagnostic utility of plasma lncRNA small nucleolar RNA host gene 1 in patients with hepatocellular carcinoma. Molecular medicine reports 2018;18(3):3305-13. | Exclude on population: not cirrhosis |
| Garcia Gullon, C., Rendon Unceta, P., Martin Herrera, L., Soria de la Cruz, M. J., Macias, M. A. Usefulness of ultrasonography in the early diagnosis of hepatocarcinoma in patients with liver cirrhosis. Revista espanola de enfermedades digestivas : organo oficial de la Sociedad Espanola de Patologia Digestiva 1995;87(11):798-801. | Does not meet full eligibility criteria: pre-2005 |
| Garretti, L, Faucigliettі, P, Regge, D, Bonino, F, Brunetto, M. [Evaluation of the usefulness of ultrasonics in the diagnosis of cancerous cirrhosis. Comparison with CT]. Valutazione dell'utilita dell'ecotomografia nella diagnosi della cancro-cirrosi. Confronto con la TC. 1988;76(3):187-92. | Does not meet full eligibility criteria: pre-2005 |
| Gatselis, Nikolaos K., Tornai, Tamas, Shums, Zakera, Zachou, Kalliopi, Saitis, Asterios, Gabeta, Stella, et al. Golgi protein-73: A biomarker for assessing cirrhosis and prognosis of liver disease patients. World journal of gastroenterology 2020;26(34):5130-45. | No 2x2 data |
| Gatto, A., De Gaetano, A. M., Giuga, M., Ciresa, M., Siciliani, L., Miele, L., et al. Differentiating hepatocellular carcinoma from dysplastic nodules at gadobenate dimeglumine-enhanced hepatobiliary-phase magnetic resonance imaging. Abdominal imaging 2013;38(4):736-44. | Exclude on population: clinical signs and symptoms of HCC |
| Gavilan, Juan Carlos, Ojeda, Guillermo, Arnedo, Rocio. Predictive factors of risk of hepatocellular carcinoma in chronic hepatitis C. European journal of internal medicine 2013;24(8):846-51. | Exclude on population: not cirrhosis |
| Ge, Tianxiang, Shen, Qiujin, Wang, Ning, Zhang, Yurong, Ge, Zhouhong, Chu, Wei, et al. Diagnostic values of alpha-fetoprotein, dickkopf-1, and osteopontin for hepatocellular carcinoma. Medical oncology (Northwood, London, England) 2015;32(3):59. | Duplicate (including Cochrane includes) |
| Ge, Tianxiang, Shen, Qiujin, Wang, Ning, Zhang, Yurong, Ge, Zhouhong, Chu, Wei, et al. Diagnostic values of alpha-fetoprotein, dickkopf-1, and osteopontin for hepatocellular carcinoma. Medical oncology (Northwood, London, England) 2015;32(3):59. | Exclude on population: not cirrhosis |

| Reference | Reason for exclusion |
|---|---|
| Gentile, Ivan, Buonomo, Antonio Riccardo, Scotto, Riccardo, Zappulo, Emanuela, Carriero, Canio, Piccirillo, Mauro, et al. Diagnostic Accuracy of PIVKA-II, Alpha-Fetoprotein and a Combination of both in Diagnosis of Hepatocellular Carcinoma in Patients Affected by Chronic HCV Infection. In vivo (Athens, Greece) 2017;31(4):695-700. | Does not meet full eligibility criteria: includes people without cirrhosis |
| Gentile, Ivan, Buonomo, Antonio Riccardo, Scotto, Riccardo, Zappulo, Emanuela, Carriero, Canio, Piccirillo, Mauro, et al. Diagnostic Accuracy of PIVKA-II, Alpha-Fetoprotein and a Combination of both in Diagnosis of Hepatocellular Carcinoma in Patients Affected by Chronic HCV Infection. In vivo (Athens, Greece) 2017;31(4):695-700. | Does not meet full eligibility criteria: includes people without cirrhosis |
| Gharib, Amal F., Eed, Emad M., Khalifa, Amany S., Raafat, Nermin, Shehab-Eldeen, Somaia, Alwakeel, Hany R., Darwiesh, Ehab. Value of Serum miRNA-96-5p and miRNA-99a-5p as Diagnostic Biomarkers for Hepatocellular Carcinoma. International journal of general medicine 2022;15():2427-36. | Does not meet full eligibility criteria: includes people without cirrhosis |
| Ghiuchici, Ana Maria, Danila, Mirela, Popescu, Alina, Sirli, Roxana, Moga, Tudor, Topan, Madalina, Bende, Felix. Contrast-enhanced ultrasound algorithm (ACR CEUS LI-RADSv 2017)- a valuable tool for the noninvasive diagnosis of hepatocellular carcinoma in patients with chronic liver disease. Medical ultrasonography 2021;23(4):383-89. | Exclude on population: clinical signs and symptoms of HCC |
| Ghosh, Adarsh, Yadav, Richa, Shalimar. Respiratory triggered diffusion-weighted imaging with a single diffusion sensitising gradient to reduce image acquisition time - A feasibility study in the workup of hepatocellular carcinoma. European journal of radiology 2021;141():109807. | Exclude on population: clinical signs and symptoms of HCC |
| Ghosh, Amit, Ghosh, Alip, Datta, Somenath, Dasgupta, Debanjali, Das, Soumyajit, Ray, Sukanta, et al. Hepatic miR-126 is a potential plasma biomarker for detection of hepatitis B virus infected hepatocellular carcinoma. International journal of cancer 2016;138(11):2732-44. | Does not meet full eligibility criteria: includes people without cirrhosis |
| Ghosh, Suchandrima, Bhowmik, Sayantani, Majumdar, Swagata, Goswami, Avijit, Chakraborty, Joyeeta, Gupta, Subash, et al. The exosome encapsulated microRNAs as circulating diagnostic marker for hepatocellular carcinoma with low alpha-fetoprotein. International journal of cancer 2020;147(10):2934-47. | Does not meet full eligibility criteria: includes people without cirrhosis |
| Giangregorio, F., Comparato, G., Marinone, M. G., Di Stasi, M., Sbolli, G., Aragona, G., Tansini, P. Imaging detection of new HCCs in cirrhotic patients treated with different techniques: Comparison of conventional US, spiral CT, and 3-dimensional contrast-enhanced US with the Navigator technique (Nav 3D CEUS)(). Journal of ultrasound 2009;12(1):12-21. | Exclude on population: HCC participants not treatment-naive |
| Giangregorio, Francesco. Contrast-Enhanced Ultrasound (CEUS) for Echographic Detection of Hepato Cellular Carcinoma in Cirrhotic Patients Previously Treated with Multiple Techniques: Comparison of Conventional US, Spiral CT and 3-Dimensional CEUS with Navigator Technique (3DNav CEUS). Cancers 2011;3(2):1763-76. | Exclude on population: HCC participants not treatment-naive |
| Giannelli, Gianluigi, Fransvea, Emilia, Trerotoli, Paolo, Beaugrand, Michel, Marinosci, Felice, Lupo, Luigi, et al. Clinical validation of combined serological biomarkers for improved hepatocellular carcinoma diagnosis in 961 patients. Clinica chimica acta; international journal of clinical chemistry 2007;383(1-2):147-52. | Does not meet full eligibility criteria: includes people without cirrhosis |
| Giannelli, Gianluigi, Fransvea, Emilia, Trerotoli, Paolo, Beaugrand, Michel, Marinosci, Felice, Lupo, Luigi, et al. Clinical validation of combined serological biomarkers for improved hepatocellular carcinoma diagnosis in 961 patients. Clinica chimica acta; international journal of clinical chemistry 2007;383(1-2):147-52. | Does not meet full eligibility criteria: includes people without cirrhosis |
| Giannelli, Gianluigi, Marinosci, Felice, Trerotoli, Paolo, Volpe, Anna, Quaranta, Michele, Dentico, Pietro. SCCA antigen combined with alpha-fetoprotein as serologic markers of HCC. International journal of cancer 2005;117(3):506-09. | Duplicate (including Cochrane includes) |
| Giannini, E. G. Accuracy of alpha-Fetoprotein Measurement in Detection of Hepatocellular Carcinoma-1 More Nail in the Coffin. Clinical Gastroenterology and Hepatology 2014;12(12):2138-39. | Exclude on study design: not a 1- or 2-gate test accuracy study |
| Giannini, Edoardo G. & Erroi, Virginia. Effectiveness of alpha-fetoprotein for hepatocellular carcinoma surveillance: the return of the living-dead? Expert review of gastroenterology & hepatology 2012;6(4):441-44. | Secondary publication of included study |

| Reference | Reason for exclusion |
|---|---|
| Giannoulis, E. & Arvanitakis, C. Diagnostic value of serum ferritin in primary hepatocellular carcinoma. Digestion 1984;30(4):236-41. | Exclude on population: not cirrhosis |
| Giardina, M. G., Matarazzo, M., Morante, R., Lucariello, A., Varriale, A., Guardasole, V. Serum alpha-L-fucosidase activity and early detection of hepatocellular carcinoma: a prospective study of patients with cirrhosis. Cancer 1998;83(12):2468-74. | No 2x2 data |
| Giardina, M. G., Matarazzo, M., Varriale, A., Morante, R., Napoli, A. Serum alpha-L-fucosidase. A useful marker in the diagnosis of hepatocellular carcinoma. Cancer 1992;70(5):1044-48. | Does not meet full eligibility criteria: includes people without cirrhosis |
| Gibriel, A. A., Al-Anany, A. M., Al-Arab, M. A. E. Investigating circulatory microRNA expression profiles in Egyptian patients infected with hepatitis C virus mediated hepatic disorders. Meta Gene 2020;26():100792. | Does not meet full eligibility criteria: includes people without cirrhosis |
| Gibriel, Abdullah Ahmed, Ismail, Manal Fouad, Sleem, Hameis, Zayed, Naglaa, Yosry, Ayman, El-Nahaas, Saeed M. Diagnosis and staging of HCV associated fibrosis, cirrhosis and hepatocellular carcinoma with target identification for miR-650, 552-3p, 676-3p, 512-5p and 147b. Cancer biomarkers : section A of Disease markers 2022;34(3):413-30. | Genomic biomarker not validated |
| Gil-Gomez, Antonio, Rojas, Angela, Liu, Chang-Hai, Gallego-Duran, Rocio, Munoz-Hernandez, Rocio, Fassina, Giorgio, et al. Combination of squamous cell carcinoma antigen immunocomplex and alpha-fetoprotein in mid- and long-term prediction of hepatocellular carcinoma among cirrhotic patients. World journal of gastroenterology 2021;27(48):8343-56. | Exclude on study design: not a 1- or 2-gate test accuracy study |
| Giorgio, Antonio, De Luca, Massimo, Gatti, Pietro, Matteucci, Paolo. Can contrast-enhanced ultrasound with perfluorobutane add value in detection of hepatocellular carcinoma in cirrhosis during surveillance? Quantitative imaging in medicine and surgery 2019;9(8):1466-69. | Exclude on study design: not a 1- or 2-gate test accuracy study |
| Giorgio, Antonio, Montesarchio, Luca, Gatti, Piero, Amendola, Ferdinando, Matteucci, Paolo, Santoro, Bruno, et al. Contrast-Enhanced Ultrasound: a Simple and Effective Tool in Defining a Rapid Diagnostic Work-up for Small Nodules Detected in Cirrhotic Patients during Surveillance. Journal of gastrointestinal and liver diseases : JGLD 2016;25(2):205-11. | Exclude on population: clinical signs and symptoms of HCC |
| Girardet, Raphael, Dubois, Margaux, Manasseh, Gibran, Jreige, Mario, Du Pasquier, Celine, Canniff, Emma, et al. The combination of non-contrast abbreviated MRI and alpha foetoprotein has high performance for hepatocellular carcinoma screening. European radiology 2023;33(10):6929-38. | Does not meet full eligibility criteria: includes people without cirrhosis |
| Giray, Burcu Gurer, Emekdas, Gurol, Tezcan, Seda, Ulger, Mahmut, Serin, Mehmet Sami, Sezgin, Orhan, Altintas, Engin. Profiles of serum microRNAs; miR-125b-5p and miR223-3p serve as novel biomarkers for HBV-positive hepatocellular carcinoma. Molecular biology reports 2014;41(7):4513-19. | No 2x2 data |
| Golam, R. M., Khalil, M. A. F., Shaker, O. G., Ahmed, T. I., Abd Elguaad, M. K., Hassan, E. A., et al. The clinical significance of long non-coding RNAs MALAT1 and CASC2 in the diagnosis of HCV-related hepatocellular carcinoma. PLoS ONE 2024;19(5 May):e0303314. | Exclude on population: not cirrhosis |
| Goldman, Radoslav, Ressom, Habtom W., Abdel-Hamid, Mohamed, Goldman, Lenka, Wang, Antai, Varghese, Rency S., et al. Candidate markers for the detection of hepatocellular carcinoma in low-molecular weight fraction of serum. Carcinogenesis 2007;28(10):2149-53. | Exclude on population: not cirrhosis |
| Goldman, Radoslav, Ressom, Habtom W., Varghese, Rency S., Goldman, Lenka, Bascug, Gregory, Loffredo, Christopher A., et al. Detection of hepatocellular carcinoma using glycomic analysis. Clinical cancer research : an official journal of the American Association for Cancer Research 2009;15(5):1808-13. | Exclude on index test |
| Golfieri, Rita, Renzulli, Matteo, Lucidi, Vincenzo, Corcioni, Beniamino, Trevisani, Franco. Contribution of the hepatobiliary phase of Gd-EOB-DTPA-enhanced MRI to Dynamic MRI in the detection of hypovascular small (<= 2 cm) HCC in cirrhosis. European radiology 2011;21(6):1233-42. | Exclude on population: clinical signs and symptoms of HCC |
| Gomez Rubio, M. & De Cuenca Moron, B. Tumoral markers in liver cirrhosis and hepatocellular carcinoma [3]. Gastroenterologia y Hepatologia 2005;28(5):308. | Foreign language |

| Reference | Reason for exclusion |
|---|---|
| Gong, Jiao, Jie, Yusheng, Xiao, Cuicui, Zhou, Wenying, Li, Xinhua, Chen, Yaqiong, et al. Increased Expression of Fibulin-1 Is Associated With Hepatocellular Carcinoma Progression by Regulating the Notch Signaling Pathway. Frontiers in cell and developmental biology 2020;8():478. | Does not meet full eligibility criteria: includes people without cirrhosis |
| Gong, L., Zhu, X., Zhang, W., Ni, H., Yin, W. Plasma lncrnas acting as fingerprints in predicting hepatocellular carcinoma from hbv positive chronic hepatitis. International Journal of Clinical and Experimental Medicine 2018;11(11):12088-96. | Exclude on population: not cirrhosis |
| Gopal, P., Yopp, A. C., Waljee, A. K., Chiang, J., Nehra, M., Kandunoori, P. Factors that affect accuracy of alpha-fetoprotein test in detection of hepatocellular carcinoma in patients with cirrhosis. Clinical Gastroenterology and Hepatology 2014;12(5):870-77. | Duplicate (including Cochrane includes) |
| Goshima, S., Kanematsu, M., Kondo, H., Watanabe, H., Noda, Y., Fujita, H. Computer-aided assessment of hepatic contour abnormalities as an imaging biomarker for the prediction of hepatocellular carcinoma development in patients with chronic hepatitis C. European Journal of Radiology 2015;84(5):811-15. | Exclude on index test |
| Gouas, D. A., Villar, S., Ortiz-Cuaran, S., Legros, P., Ferro, G., Kirk, G. D., et al. TP53 R249S mutation, genetic variations in HBX and risk of hepatocellular carcinoma in The Gambia. Carcinogenesis 2012;33(6):1219-24. | Does not meet full eligibility criteria: includes people without cirrhosis |
| Goyal, R. A Symbolic Regression Approach to Hepatocellular Carcinoma Diagnosis Using Hypermethylated CpG Islands in Circulating Cell-Free DNA. medRxiv 2022;():n. pag.. | Exclude on population: not cirrhosis |
| Grammatikos, Georgios, Schoell, Niklas, Ferreiros, Nerea, Bon, Dimitra, Herrmann, Eva, Farnik, Harald, et al. Serum sphingolipidomic analyses reveal an upregulation of C16-ceramide and sphingosine-1-phosphate in hepatocellular carcinoma. Oncotarget 2016;7(14):18095-10505. | No 2x2 data |
| Granito, A., Galassi, M., Piscaglia, F., Romanini, L., Lucidi, V., Renzulli, M., et al. Impact of gadoxetic acid (Gd-EOB-DTPA)-enhanced magnetic resonance on the non-invasive diagnosis of small hepatocellular carcinoma: a prospective study. Alimentary pharmacology & therapeutics 2013;37(3):355-63. | Exclude on population: clinical signs and symptoms of HCC |
| Gray, Joe, Chattopadhyay, Dipankar, Beale, Gary S., Patman, Gillian L., Miele, Luca, King, Barry P., et al. A proteomic strategy to identify novel serum biomarkers for liver cirrhosis and hepatocellular cancer in individuals with fatty liver disease. BMC cancer 2009;9():271. | Exclude on index test |
| Grazi, G L, Mazziotti, A, Legnani, C, Jovine, E, Miniero, R, Gallucci, A, Palareti, G. The role of tumor markers in the diagnosis of hepatocellular carcinoma, with special reference to the des-gamma-carboxy prothrombin. Liver transplantation and surgery : official publication of the American Association for the Study of Liver Diseases and the International Liver Transplantation Society 1995;1(4):249-55. | Exclude on population: not cirrhosis |
| Grazi, G. L., Mazziotti, A., Legnani, C., Jovine, E., Miniero, R., Gallucci, A., Palareti, G. The des-gamma-carboxy prothrombin in the diagnosis of hepatocellular carcinoma. Comparison with other tumor markers. Chirurgia 1994;7(10):726-32. | Does not meet full eligibility criteria: pre-2005 |
| Grazi, G. L., Mazziotti, A., Legnani, C., Jovine, E., Miniero, R., Gallucci, A., Palareti, G. The role of tumor markers in the diagnosis of hepatocellular carcinoma, with special reference to the des-gamma-carboxy prothrombin. Liver transplantation and surgery : official publication of the American Association for the Study of Liver Diseases and the International Liver Transplantation Society 1995;1(4):249-55. | Does not meet full eligibility criteria: includes people without cirrhosis |
| Gruden, Gabriella, Carucci, Patrizia, Lolli, Valentina, Cosso, Loretta, Dellavalle, Erika, Rolle, Emanuela, et al. Serum heat shock protein 27 levels in patients with hepatocellular carcinoma. Cell stress & chaperones 2013;18(2):235-41. | Does not meet full eligibility criteria: includes people without cirrhosis |
| Guan, M. C., Ouyang, W., Liu, S. Y., Sun, L. Y., Chen, W. Y., Tong, X. M., Zhu, H. Alpha-fetoprotein, protein induced by vitamin K absence or antagonist-II, lens culinaris agglutinin-reactive fraction of alpha-fetoprotein alone and in combination for early detection of hepatocellular carcinoma from nonalcoholic fatty liver disease: A multicenter analysis. Hepatobiliary and Pancreatic Diseases International 2022;():n. pag.. | Exclude on population: not cirrhosis |

| Reference | Reason for exclusion |
|---|---|
| Guan, Ming-Cheng, Ouyang, Wei, Liu, Si-Yu, Sun, Li-Yang, Chen, Wei-Yue, Tong, Xiang-Min, Zhu, Hong. Alpha-fetoprotein, protein induced by vitamin K absence or antagonist-II, lens culinaris agglutinin-reactive fraction of alpha-fetoprotein alone and in combination for early detection of hepatocellular carcinoma from nonalcoholic fatty liver disease: A multicenter analysis. Hepatobiliary & pancreatic diseases international : HBPD INT 2022;21(6):559-68. | Duplicate (including Cochrane includes) |
| Guan, W., Zhang, C., Miao, T., Dong, C., Li, L., Yuan, X., et al. The Potential of the lncRNAs ADAMTSL4-AS1, AC067931 and SOCS2-AS1 in Peripheral Blood Mononuclear Cells as Novel Diagnostic Biomarkers for Hepatitis B Virus-Associated Hepatocellular Carcinoma. Journal of Hepatocellular Carcinoma 2024;11():1221 " 1233. | Does not meet full eligibility criteria: includes people without cirrhosis |
| Guan, Wenqian, Gao, Zhiyuan, Huang, Chenjun, Fang, Meng, Feng, Huijuan, Chen, Shipeng, et al. The diagnostic value of serum DSA-TRF in hepatocellular carcinoma. Glycoconjugate journal 2020;37(2):231-40. | Does not meet full eligibility criteria: includes people without cirrhosis |
| Guarneri, V., Loggi, E., Ramacieri, G., Serra, C., Vukotic, R., Vitale, G., et al. Diagnostic Performance of PIVKA-II in Italian Patients with Hepatocellular Carcinoma. Cancers 2025;17(2):167. | Does not meet full eligibility criteria: includes people without cirrhosis |
| Guiu, Boris, Loffroy, Romaric, Ben Salem, Douraied, Lepage, Come, Guiu, Severine, Aho, Serge, et al. Combined SPIO-gadolinium magnetic resonance imaging in cirrhotic patients: negative predictive value and role in screening for hepatocellular carcinoma. Abdominal imaging 2008;33(5):520-28. | Exclude on population: clinical signs and symptoms of HCC |
| Gulzar, N., Pari, A., Naeem, S., Yousaf, S. Prevalence of hepatocellular carcinoma in hepatitis C patients. Pakistan Journal of Medical and Health Sciences 2018;12(1):334-36. | Exclude on population: not cirrhosis |
| Gumilas, N. S. A., Widodo, I., Ratnasari, N. Potential relative quantities of miR-122 and miR-150 to differentiate hepatocellular carcinoma from liver cirrhosis. Non-coding RNA Research 2022;7(1):34-39. | Does not meet full eligibility criteria: includes people without cirrhosis |
| Gumilas, Nur Signa Aini, Harini, Ika Murti, Ernawati, Dwi Arini, Indriani, Vitasari, Novrial, Dody. Potential of Apolipoprotein A1 (ApoA1) for Detecting Liver Cirrhosis and Hepatocellular Carcinoma. Asian Pacific journal of cancer prevention : APJCP 2022;23(6):2001-08. | Exclude on index test |
| Guo, De-Zhen, Huang, Ao, Wang, Ying-Chao, Zhou, Shuang, Wang, Hui, Xing, Xiang-Lei, et al. Early detection and prognosis evaluation for hepatocellular carcinoma by circulating tumour DNA methylation: A multicentre cohort study. Clinical and translational medicine 2024;14(5):e1652. | Does not meet full eligibility criteria: includes people without cirrhosis |
| Guo, H. M., Li, J. S., Zhang, J. P., Li, D. Y. Significance of serum SCD25 level in diagnosis of hepatocellular carcinoma. World Chinese Journal of Digestology 2014;22(34):5344-49. | Foreign language |
| Guo, Jiao, Jing, Rui, Zhong, Jian-Hong, Dong, Xin, Li, Yun-Xi, Liu, Yin-Kun, Huang, Tian-Ren. Identification of CD14 as a potential biomarker of hepatocellular carcinoma using iTRAQ quantitative proteomics. Oncotarget 2017;8(37):62011-28. | Does not meet full eligibility criteria: includes people without cirrhosis |
| Guo, L., Hao, X., Chen, L., Qian, Y., Liu, X., Fan, X., et al. Early warning of hepatocellular carcinoma in cirrhotic patients by three-phase CT-based deep learning radiomics model: a retrospective, multicentre, cohort study. eClinicalMedicine 2024;74():102718. | Exclude on index test |
| Guo, L., Liang, C., Yu, T., Wang, G., Li, N., Sun, H., Gao, F. 3 T MRI of hepatocellular carcinomas in patients with cirrhosis: does T2-weighted imaging provide added value? Clinical radiology 2012;67(4):319-28. | Exclude on population: clinical signs and symptoms of HCC |
| Guo, Ping, Zheng, Hailing, Li, Yihan, Li, Yuntong, Xiao, Yue, Zheng, Jin, et al. Hepatocellular carcinoma detection via targeted enzymatic methyl sequencing of plasma cell-free DNA. Clinical epigenetics 2023;15(1):2. | Exclude on population: not cirrhosis |
| Guo, Wei, Sun, Yun-Fan, Shen, Min-Na, Ma, Xiao-Lu, Wu, Jiong, Zhang, Chun-Yan, et al. Circulating Tumor Cells with Stem-Like Phenotypes for Diagnosis, Prognosis, and Therapeutic Response Evaluation in Hepatocellular Carcinoma. Clinical cancer research : an official journal of the American Association for Cancer Research 2018;24(9):2203-13. | Exclude on population: not cirrhosis |

| Reference | Reason for exclusion |
|---|---|
| Guo, Wenbo, Lu, Jilin, Yan, Linlin, Sun, Debin, Gong, Longlong. Molecular Alterations of Circulating Cell-Free DNA in the Pathological Progression of Hepatocellular Carcinoma. Journal of oncology 2021;2021():3637436. | Exclude on population: not cirrhosis |
| Guo, Xin, Lv, Xiaohui, Lv, Xing, Ma, Yueyun, Chen, Lin. Circulating miR-21 serves as a serum biomarker for hepatocellular carcinoma and correlated with distant metastasis. Oncotarget 2017;8(27):44050-58. | Does not meet full eligibility criteria: includes people without cirrhosis |
| Guo, Xuli, Xiong, Hailin, Dong, Shaoting. Identification and Validation of a Novel Immune Infiltration-Based Diagnostic Score for Early Detection of Hepatocellular Carcinoma by Machine-Learning Strategies. Gastroenterology research and practice 2022;2022():5403423. | Exclude on population: not cirrhosis |
| Guo, Yantong, Zhao, Jingming, Bi, Jingtao, Wu, Quan, Wang, Xin. Heterogeneous nuclear ribonucleoprotein K (hnRNP K) is a tissue biomarker for detection of early hepatocellular carcinoma in patients with cirrhosis. Journal of hematology & oncology 2012;5():37. | Exclude on population: clinical signs and symptoms of HCC |
| Guo, Z., Mo, H., Yuan, Y. Diagnostic and Prognostic Value of Combined Detection of Serum Protein Induced by Vitamin K Absence or Antagonist-II, Alpha-Fetoprotein, and Spliced Hepatitis B Virus in Hepatitis B Virus-Induced Hepatocellular Carcinoma. Cancer Biotherapy and Radiopharmaceuticals 2025;():n. pag.. | Does not meet full eligibility criteria: includes people without cirrhosis |
| Gupta, P., Soundararajan, R., Subramanian, P., Talasila, P., Singh, S., Gulati, A., et al. Liver Observations in Chronic Liver Disease on Noncontrast Abbreviated Magnetic Resonance Imaging MRI (AMRI): Proposal of Modified Liver Imaging Reporting and Data System (AMRI-LI-RADS) Categorization. ghted imaging; Journal of Clinical and Experimental Hepatology 2025;15(6):102605. | Does not meet full eligibility criteria: includes people without cirrhosis |
| Gutierrez Reyes, Cristian D., Huang, Yifan, Atashi, Mojgan, Zhang, Jie, Zhu, Jianhui, Liu, Suyu, et al. PRM-MS Quantitative Analysis of Isomeric N-Glycopeptides Derived from Human Serum Haptoglobin of Patients with Cirrhosis and Hepatocellular Carcinoma. Metabolites 2021;11(8):n. pag.. | No 2x2 data |
| Haberman, Diego, Mela, Martin, Martinez, Adriana, Mancinelli, Adrian, Laguens, Ruben, Gruz, Fernando. [Accuracy of multislice computed tomography in the diagnosis of hepatocellular carcinoma in patients with cirrhosis evaluated for liver transplantation]. Precision de la tomografia multidetector en el diagnostico de hepatocarcinoma en pacientes con cirrosis evaluados para trasplante hepatico. 2011;41(3):190-98. | Foreign language |
| Habieb, Alaa, Matboli, Marwa, El-Tayeb, Hanaa. Potential role of lncRNA-TSIX, miR-548-a-3p, and SOGA1 mRNA in the diagnosis of hepatocellular carcinoma. Molecular biology reports 2019;46(4):4581-90. | Does not meet full eligibility criteria: includes people without cirrhosis |
| Hadi, Hana, Wan Shuaib, Wan Muhammad Azfar, Raja Ali, Raja Affendi. Utility of PIVKA-II and AFP in Differentiating Hepatocellular Carcinoma from Non-Malignant High-Risk Patients. Medicina (Kaunas, Lithuania) 2022;58(8):n. pag.. | Does not meet full eligibility criteria: includes people without cirrhosis |
| Hagag, Neven A., Ali, Yasser B. M., Elsharawy, Ahmed A. Clinical Impact of Circulated miR-1291 in Plasma of Patients with Liver Cirrhosis (LC) and Hepatocellular Carcinoma (HCC): Implication on Glypican-3 Expression. Journal of gastrointestinal cancer 2020;51(1):234-41. | Does not meet full eligibility criteria: includes people without cirrhosis |
| Hallager, S., Ladelund, S., Kjaer, M., Madsen, L. G., Belard, E., Laursen, A. L., et al. Hepatocellular carcinoma in patients with chronic hepatitis C and cirrhosis in Denmark: A nationwide cohort study. Journal of Viral Hepatitis 2018;25(1):47-55. | Duplicate (including Cochrane includes) |
| Hamad, R. S., Al Abdulsalam, N. K., Elrefaiy, M. A. Interplay between TGF-b1 and miRNA-122 biomarkers in hepatocellular carcinoma progression in patients with chronic hepatitis C. Tropical biomedicine 2022;39(4):559-68. | Does not meet full eligibility criteria: includes people without cirrhosis |
| Hammad, Lamiaa N., Abdelraouf, Sahar M., Hassanein, Fetouh S., Mohamed, Walid A. Circulating IL-6, IL-17 and vitamin D in hepatocellular carcinoma: potential biomarkers for a more favorable prognosis? Journal of immunotoxicology 2013;10(4):380-86. | Does not meet full eligibility criteria: includes people without cirrhosis |
| Hammad, Reham, Aglan, Reda Badr, Mohammed, Shaymaa A., Awad, Eman Abu-Elnasr, Elsaid, Marwa A., Bedair, Hanan M., et al. Cytotoxic T Cell Expression of Leukocyte-Associated Immunoglobulin-Like Receptor-1 (LAIR-1) | Does not meet full eligibility criteria: includes people without cirrhosis |

| Reference | Reason for exclusion |
|---|---|
| in Viral Hepatitis C-Mediated Hepatocellular Carcinoma. International journal of molecular sciences 2022;23(20):n. pag.. | |
| Han, Chao, Gao, Lanzhu, Bai, Han. Identification of a role for serum aldo-keto reductase family 1 member B10 in early detection of hepatocellular carcinoma. Oncology letters 2018;16(6):7123-30. | Duplicate (including Cochrane includes) |
| Han, Chao, Gao, Lanzhu, Bai, Han. Identification of a role for serum aldo-keto reductase family 1 member B10 in early detection of hepatocellular carcinoma. Oncology letters 2018;16(6):7123-30. | Exclude on population: healthy controls |
| Han, J., Li, J., Liu, W., Liang, J., Huang, Z., Wang, S., et al. Identification of miR-148a in plasma as a potential noninvasive biomarker for hepatocellular carcinoma. Gut 2018;67(Supplement 2):A101-02. | CONFERENCE ABSTRACT |
| Han, Jun, Han, Min-Lu, Xing, Hao, Li, Zhen-Li, Yuan, Dao-Yi, Wu, Han, et al. Tissue and serum metabolomic phenotyping for diagnosis and prognosis of hepatocellular carcinoma. International journal of cancer 2020;146(6):1741-53. | Does not meet full eligibility criteria: includes people without cirrhosis |
| Han, Jun, Qin, Wen-Xing, Li, Zhen-Li, Xu, Ai-Jing, Xing, Hao, Wu, Han, et al. Tissue and serum metabolite profiling reveals potential biomarkers of human hepatocellular carcinoma. Clinica chimica acta; international journal of clinical chemistry 2019;488():68-75. | Does not meet full eligibility criteria: includes people without cirrhosis |
| Han, Juqiang, Li, Jiarui, Qian, Yun, Liu, Wenpeng, Liang, Jiguang, Huang, Zhigang, Wang, Shuai. Identification of plasma miR-148a as a noninvasive biomarker for hepatocellular carcinoma. Clinics and research in hepatology and gastroenterology 2019;43(5):585-93. | Does not meet full eligibility criteria: includes people without cirrhosis |
| Han, Ke-Qi, Han, Hui, He, Xue-Qun, Wang, Lei, Guo, Xiao-Dong, Zhang, Xue-Ming, et al. Chemokine CXCL1 may serve as a potential molecular target for hepatocellular carcinoma. Cancer medicine 2016;5(10):2861-71. | No 2x2 data |
| Han, Li-Yan, Fan, Yu-Chen, Mu, Nan-Nan, Gao, Shuai, Li, Feng, Ji, Xiang-Fen, Dou, Cheng-Yun. Aberrant DNA methylation of G-protein-coupled bile acid receptor Gpbar1 (TGR5) is a potential biomarker for hepatitis B Virus associated hepatocellular carcinoma. International journal of medical sciences 2014;11(2):164-71. | Exclude on population: not cirrhosis |
| Han, Li-Yan, Fan, Yu-Chen, Mu, Nan-Nan, Gao, Shuai, Li, Feng, Ji, Xiang-Fen, Dou, Cheng-Yun. Aberrant DNA methylation of G-protein-coupled bile acid receptor Gpbar1 (TGR5) is a potential biomarker for hepatitis B Virus associated hepatocellular carcinoma. International journal of medical sciences 2014;11(2):164-71. | Duplicate (including Cochrane includes) |
| Han, Li-Yan, Sun, Wei-Juan, Zhao, Ze-Hua, Gao, Shuai. Decreased GPX3 mRNA level in peripheral blood mononuclear cells is associated with HBV-related hepatocellular carcinoma. Transactions of the Royal Society of Tropical Medicine and Hygiene 2023;117(10):727-32. | Exclude on population: not cirrhosis |
| Han, Li-Yan, Sun, Wei-Juan, Zhao, Ze-Hua, Gao, Shuai. Decreased GPX3 mRNA level in peripheral blood mononuclear cells is associated with HBV-related hepatocellular carcinoma. Transactions of the Royal Society of Tropical Medicine and Hygiene 2023;117(10):727â€“732. | Exclude on population: not cirrhosis |
| Han, Li-Yan, Yang, Jie-Ru, Zhao, Ze-Hua, Gao, Shuai, Fan, Yu-Chen. RIPK3 mRNA level acts as a diagnostic biomarker in hepatitis B virus-associated hepatocellular carcinoma. Pathology, research and practice 2020;216(10):153147. | Does not meet full eligibility criteria: includes people without cirrhosis |
| Han, Seunghee, Choi, Joon-Il, Park, Michael Yong, Choi, Moon Hyung, Rha, Sung Eun. The Diagnostic Performance of Liver MRI without Intravenous Contrast for Detecting Hepatocellular Carcinoma: A Case-Controlled Feasibility Study. Korean journal of radiology 2018;19(4):568-77. | Does not meet full eligibility criteria: includes people without cirrhosis |
| Han, Yawei, Jiang, Wenna, Wang, Yu, Zhao, Meng, Li, Yueguo. Serum long non-coding RNA SCARNA10 serves as a potential diagnostic biomarker for hepatocellular carcinoma. BMC cancer 2022;22(1):431. | Exclude on population: not cirrhosis |
| Han, Yueting, Zhang, Youqin, Cui, Lin, Li, Ze, Feng, Honglei, Zhang, Ying, Sun, Da. Plasma heat shock protein 90alpha as a biomarker for the diagnosis of liver cancer: in patients with different clinicopathologic characteristics. World journal of surgical oncology 2021;19(1):228. | Exclude on population: clinical signs and symptoms of HCC |

| Reference | Reason for exclusion |
|---|---|
| Hanafy, Amr Shaaban & Mohamed, Mohamed Sorour. Ascitic Calprotectin as an early predictor of hepatocellular carcinoma in patients with cirrhotic ascites. Journal of cancer research and clinical oncology 2020;146(12):3207-14. | Exclude on index test |
| Hanaoka, Takuya, Sato, Shuichi, Tobita, Hiroshi, Miyake, Tatsuya, Ishihara, Shunji, Akagi, Shuji, Amano, Yuji. Clinical significance of the highly sensitive fucosylated fraction of alpha-fetoprotein in patients with chronic liver disease. Journal of gastroenterology and hepatology 2011;26(4):739-44. | Exclude on population: not cirrhosis |
| Hanna, Robert F., Ward, Thomas J., Chow, Daniel S., Lagana, Stephen M., Moreira, Roger K., Emond, Jean C., Weintraub, Joshua L. An evaluation of the sensitivity of MRI at detecting hepatocellular carcinoma in cirrhotic patients utilizing an explant reference standard. Clinical imaging 2014;38(5):693-97. | Exclude on population: HCC participants not treatment-naive |
| Hanss, M., Bonvoisin, C., Patouillard, B., Martin, T., Martin, D., Audigier, J. C., Descos, L. Increased plasma levels of urokinase type plasminogen activator during hepatocellular carcinoma. Fibrinolysis 1994;8(4):255-60. | No 2x2 data |
| Hao, X. & Xin, R. Decreased serum exosomal miR-320a expression is an unfavorable prognostic factor in patients with hepatocellular carcinoma. Journal of International Medical Research 2020;48(4):n. pag.. | Exclude on population: not cirrhosis |
| Hardie, Andrew D. & Kizziah, Michael K. Can the patient with cirrhosis be imaged for hepatocellular carcinoma without gadolinium?: Comparison of combined T2-weighted, T2*-weighted, and diffusion-weighted MRI with gadolinium-enhanced MRI using liver explantation standard. Journal of computer assisted tomography 2011;35(6):711-15. | Exclude on population: clinical signs and symptoms of HCC |
| Hardie, Andrew D. The use of T2*-weighted multi-echo GRE imaging as a novel method to diagnose hepatocellular carcinoma compared with gadolinium-enhanced MRI: a feasibility study. Magnetic resonance imaging 2010;28(2):281-85. | Exclude on population: not cirrhosis |
| Hashad, Doaa I. & Elyamany, Amany S. Mitochondrial DNA Copy Number in Egyptian Patients with Hepatitis C Virus-Related Hepatocellular Carcinoma. Genetic testing and molecular biomarkers 2015;19(11):604-09. | Exclude on population: HCC participants not treatment-naive |
| Hashem, Somaya, ElHefnawi, Mahmoud, Habashy, Shahira, El-Adawy, Mohamed, Esmat, Gamal, Elakel, Wafaa, et al. Machine Learning Prediction Models for Diagnosing Hepatocellular Carcinoma with HCV-related Chronic Liver Disease. Computer methods and programs in biomedicine 2020;196():105551. | Exclude on population: not cirrhosis |
| Hassouna, Mona M., Naguib, Mary, Radwan, Enas M., Abdel-Samiee, Mohamed, Estaphan, Suzanne. DNA Methyltransferases as Potential Biomarkers for HCV Related Hepatocellular Carcinoma. Asian Pacific journal of cancer prevention : APJCP 2020;21(11):3357-63. | Does not meet full eligibility criteria: includes people without cirrhosis |
| Hayasaka, A., Suzuki, N., Fujimoto, N., Iwama, S., Fukuyama, E., Kanda, Y. Elevated plasma levels of matrix metalloproteinase-9 (92-kd type IV collagenase/gelatinase B) in hepatocellular carcinoma. Hepatology (Baltimore, Md.) 1996;24(5):1058-62. | Exclude on index test |
| Hayashi, E., Motomura, Y., Shirakawa, H., Yoshikawa, T., Oba, N., Nishinakagawa, S., et al. Detection of glypican-3-specific CTLs in chronic hepatitis and liver cirrhosis. Oncology Reports 2009;22(1):149-54. | Exclude on index test |
| He, D., Zhang, X., Zhu, X., Maharjan, N., Wang, Y., Luo, P., Liang, C. Identify and Validate the Transcriptomic, Functional Network, and Predictive Validity of FBXL19-AS1 in Hepatocellular Carcinoma. Frontiers in Oncology 2020;10():609601. | Does not meet full eligibility criteria: includes people without cirrhosis |
| He, H., Wu, Y., Jia, Z., Pan, Y., Zhang, Y., Su, K., et al. A stratified precision screening strategy for enhancing hepatitis B- and C-associated liver cancer detection: a prospective study. Scientific reports 2025;15(1):11396. | Exclude on population: not cirrhosis |
| He, Jian. Serum TGF-beta1: A Potential Biomarker for Early Detection of Hepatocellular Carcinoma. EBioMedicine 2016;12():4-5. | Exclude on study design: not a 1- or 2-gate test accuracy study |
| He, Kun, Hu, Zemin, Ruan, Jiahou, Ma, Qianhong, Zhong, Feng, Cheng, Xinsheng, Sun, Shibo. MicroRNA301 is a potential diagnostic biomarker for hepatocellular cancer. International journal of clinical and experimental pathology 2015;8(5):5603-08. | Exclude on population: not cirrhosis |

| Reference | Reason for exclusion |
|---|---|
| He, N., Feng, G., Zhang, C., Wu, F., Zhang, T. Plasma levels of methylated septin 9 are capable of detecting hepatocellular carcinoma and hepatic cirrhosis. Molecular Medicine Reports 2020;22(4):2705-14. | No 2x2 data |
| He, N., Feng, G., Zhang, F. N., Hao, S., Li, R., Zhao, Z. Q., Tian, Y. W. [Expression and clinical significance of plasma methylated SEPT 9 gene in patients with primary liver cancer]. Zhonghua gan zang bing za zhi = Zhonghua ganzangbing zazhi = Chinese journal of hepatology 2023;31(3):265-70. | Foreign language |
| He, X., Li, Y., Han, D., Zhou, Y., Zhang, J., Xu, Q., et al. Gadoxetic acid disodium (Gd-EOB-DTPA) contrast-enhanced abbreviated magnetic resonance imaging (MRI) for hepatocellular carcinoma surveillance in at-risk patients: a multi-center study in China. ghted imaging; Quantitative Imaging in Medicine and Surgery 2024;14(12):8520 “ 8537. | Does not meet full eligibility criteria: includes people without cirrhosis |
| He, X., Wang, Y., Zhang, W., Li, H., Luo, R., Zhou, Y., et al. Screening differential expression of serum proteins in AFP-negative HBV-related hepatocellular carcinoma using iTRAQ -MALDI-MS/MS. Neoplasma 2014;61(1):17-26. | Exclude on population: not cirrhosis |
| He, Xiao-Fan, Wen, Zhi-Bin, Liu, Min-Juan, Zhang, Hui, Li, Qun. Levels of plasma des-gamma-carboxy protein C and prothrombin in patients with liver diseases. World journal of gastroenterology 2004;10(20):3073-75. | Exclude on index test |
| He, Xiaomin, Hong, Yu, Wang, Xiaomei, Zhang, Xiaohong, Long, Jiang, Li, Hai, et al. Identification and clinical significance of an elevated level of serum aminoacylase-1 autoantibody in patients with hepatitis B virus-related liver cirrhosis. Molecular medicine reports 2016;14(5):4255-62. | Exclude on target condition: not HCC |
| Hecht, Elizabeth M., Holland, Agnes E., Israel, Gary M., Hahn, Winnie Y., Kim, Danny C., West, A. Brian, et al. Hepatocellular carcinoma in the cirrhotic liver: gadolinium-enhanced 3D T1-weighted MR imaging as a stand-alone sequence for diagnosis. Radiology 2006;239(2):438-47. | No 2x2 data |
| Hegazy, E. A. A. A., Eissa, S. A., Abde, A. E., Sorour, H., Fouad, H. H., Abde, D. Evaluation of serum protein markers in diagnosis of hepatocellular carcinoma and carcinogenesis risk assessment in chronic liver disease patients. Asian Pacific Journal of Tropical Disease 2017;7(9):564-68. | Does not meet full eligibility criteria: includes people without cirrhosis |
| Helaly, Ghada F. Influence of hepatitis C virus infection on circulating levels of sICAM-1 and VEGF in patients with hepatitis C and hepatocellular carcinoma (HCC) and their role in enhancing detection of HCC. The Egyptian journal of immunology 2006;13(1):27-38. | Exclude on population: not cirrhosis |
| Helmberger, Thomas K., Laubenberger, Jorg, Rummeny, Ernst, Jung, Gregor, Sievers, Klaus, Dohring, Wilfried, Meurer, Karoline. MRI characteristics in focal hepatic disease before and after administration of MnDPDP: discriminant analysis as a diagnostic tool. European radiology 2002;12(1):62-70. | Exclude on population: clinical signs and symptoms of HCC |
| Hemken, Philip M., Sokoll, Lori J., Yang, Xiaoqing, Dai, Jianliang, Elliott, Debra, Gawel, Susan H., et al. Validation of a novel model for the early detection of hepatocellular carcinoma. Clinical proteomics 2019;16():2. | Secondary publication of included study |
| Henderson, J. M., Campbell, J. D., Olson, R. Role of computed tomography in screening for hepatocellular carcinoma in patients with cirrhosis. Gastrointestinal radiology 1988;13(2):129-34. | Does not meet full eligibility criteria: pre-2005 |
| Heo, S., Kim, S. Y., Lee, S. J., Lee, S. S., Byun, J. H., Won, H. J., et al. LI-RADS Ultrasound Surveillance Version 2024: Comparison With Version 2017 for Hepatocellular Carcinoma Detection and Risk Factors for Visualization Score C. American Journal of Roentgenology 2025;224(4):e2432433. | Secondary publication of included study |
| Hernandez-Meza, G., Violi, N. V., Said, D., Novogrodsky, E., Villavisanis, D., Maron, S. Z., et al. MRI is the most commonly used imaging modality for HCC screening at a tertiary care transplant center. Abdominal Radiology 2021;46(11):5142-51. | Exclude on study design: not a 1- or 2-gate test accuracy study |
| Higashi, Mikito, Yoshimura, Takeshi, Usui, Noriyoshi, Kano, Yuichiro, Deguchi, Akihiro, Tanabe, Kazuhiro, et al. A Potential Serum N-glycan Biomarker for Hepatitis C Virus-Related Early-Stage Hepatocellular Carcinoma with Liver Cirrhosis. International journal of molecular sciences 2020;21(23):n. pag.. | No 2x2 data |
| Hippo, Yoshitaka, Watanabe, Kiyotaka, Watanabe, Akira, Midorikawa, Yutaka, Yamamoto, Shogo, Ihara, Sigeo, et al. Identification of soluble NH2-terminal | Does not meet full eligibility criteria: includes people without cirrhosis |

| Reference | Reason for exclusion |
|---|---|
| fragment of glypican-3 as a serological marker for early-stage hepatocellular carcinoma. Cancer research 2004;64(7):2418-23. | |
| Hirakawa, Masakazu, Yoshimitsu, Kengo, Irie, Hiroyuki, Tajima, Tsuyoshi, Nishie, Akihiro, Asayama, Yoshiki, et al. Performance of radiological methods in diagnosing hepatocellular carcinoma preoperatively in a recipient of living related liver transplantation: comparison with step section histopathology. Japanese journal of radiology 2011;29(2):129-37. | Exclude on population: clinical signs and symptoms of HCC |
| Hirashima, N., Shimada, M., Urata, N. Surveillance of hepatocellular carcinoma using liver stiffness in patients with hepatitis C after sustained virologic response by direct-acting antivirals. Acta Hepatologica Japonica 2022;63(6):268-78. | Foreign language |
| Hlady, Ryan A., Zhao, Xia, Pan, Xiaoyu, Yang, Ju Dong, Ahmed, Fowsiyo, Antwi, Samuel O., et al. Genome-wide discovery and validation of diagnostic DNA methylation-based biomarkers for hepatocellular cancer detection in circulating cell free DNA. Theranostics 2019;9(24):7239-50. | No 2x2 data |
| Ho, C. H., Lee, S. D., Chang, H. T., Wu, J. C., Tsai, Y. T. Application of des-gamma-carboxy prothrombin as a complementary tumor marker with alpha-fetoprotein in the diagnosis of hepatocellular carcinoma. Scandinavian journal of gastroenterology 1989;24(1):47-52. | Does not meet full eligibility criteria: pre-2005 |
| Ho, S., Cheng, P., Yuen, J., Chan, A., Leung, N., Yeo, W., et al. Isoelectric focusing of alphafetoprotein in patients with hepatocellular carcinoma--frequency of specific banding patterns at non-diagnostic serum levels. British journal of cancer 1996;73(8):985-88. | Exclude on population: clinical signs and symptoms of HCC |
| Hong, Y., Long, J., Chen, S., Liu, Q., Zhang, B., He, X., et al. An Analysis of Immunoreactive Signatures in Early Stage Hepatocellular Carcinoma. EBioMedicine 2015;2(5):438-46. | Does not meet full eligibility criteria: includes people without cirrhosis |
| Honglian, J., Caiyun, H., Lijun, S., Shilong, L., Sugui, H., Xiaorui, W. Diagnostic value of serum AFP-L3, GP73 and GGT combined detection in hepatocellular carcinoma. Cancer Research and Clinic 2014;26(7):465-67. | Foreign language |
| Hou, J., Berg, T., Vogel, A., Piratvisuth, T., Trojan, J., De Toni, E. N., et al. Comparative evaluation of multimarker algorithms for early-stage HCC detection in multicenter prospective studies. JHEP Reports 2025;7(2):101263. | Exclude on population: not cirrhosis |
| Hou, Si-Cong, Xiao, Ming-Bing, Ni, Run-Zhou, Ni, Wen-Kai, Jiang, Feng, Li, Xiao-Yan, Lu, Cui-Hua. Serum GP73 is complementary to AFP and GGT-II for the diagnosis of hepatocellular carcinoma. Oncology letters 2013;6(4):1152-58. | Exclude on population: healthy controls |
| Hou, Z. J., Wang, X. W., Zhang, L. H., Zhou, X. Y., Dai, D. L. Clinical significance of serum leptin, vascular endothelial growth factor and alpha-fetoprotein expression in hepatocelluar carcinoma. World Chinese Journal of Digestology 2006;14(33):3195-2000. | Foreign language |
| Howell, Jessica, Atkinson, Stephen R., Pinato, David J., Knapp, Susanne, Ward, Caroline, Minisini, Rosalba, et al. Identification of mutations in circulating cell-free tumour DNA as a biomarker in hepatocellular carcinoma. European journal of cancer (Oxford, England : 1990) 2019;116():56-66. | Exclude on study design: not a 1- or 2-gate test accuracy study |
| Hribek, P., Habartova, L., Kubickova, K., Klasova, J., Setnicka, V. The potential of spectroscopy in the diagnosis of hepatocellular carcinoma - a pilot study. Gastroenterologie a Hepatologie 2021;75(5):404-09. | Exclude on index test |
| Hsieh, S. Y., He, J. R., Yu, M. C., Lee, W. C., Chen, T. C., Lo, S. J., et al. Secreted ERBB3 isoforms are serum markers for early hepatoma in patients with chronic hepatitis and cirrhosis. Journal of Proteome Research 2011;10(10):4715-24. | No 2x2 data |
| Hsu, P. I., Chow, N. H., Lai, K. H., Yang, H. B., Chan, S. H., Lin, X. I. Z., et al. Implications of serum basic fibroblast growth factor levels in chronic liver diseases and hepatocellular carcinoma. Anticancer Research 1997;17(4 A):2803-09. | Does not meet full eligibility criteria: pre-2005 |
| Hu, J., Wang, N., Yang, Y., Ma, L., Han, R., Zhang, W., et al. Diagnostic value of alpha-fetoprotein combined with neutrophil-to-lymphocyte ratio for hepatocellular carcinoma. BMC Gastroenterology 2018;18(1):186. | Duplicate (including Cochrane includes) |
| Hu, Jian, Wang, Nianyue, Yang, Yongfeng, Ma, Li, Han, Ruilin, Zhang, Wei, et al. Diagnostic value of alpha-fetoprotein combined with neutrophil-to- | Exclude on population: not cirrhosis |

| Reference | Reason for exclusion |
|---|---|
| lymphocyte ratio for hepatocellular carcinoma. BMC gastroenterology 2018;18(1):186. | |
| Hu, Jin-song, Wu, De-wu, Liang, Shuo. GP73, a resident Golgi glycoprotein, is sensibility and specificity for hepatocellular carcinoma of diagnosis in a hepatitis B-endemic Asian population. Medical oncology (Northwood, London, England) 2010;27(2):339-45. | Exclude on population: not cirrhosis |
| Hu, Na, Fan, Xiao-Peng, Fan, Yu-Chen, Chen, Long-Yan, Qiao, Chen-Yang, Han, Li-Yan. Hypomethylated Ubiquitin-Conjugating Enzyme2 Q1 (UBE2Q1) Gene Promoter in the Serum Is a Promising Biomarker for Hepatitis B Virus-Associated Hepatocellular Carcinoma. The Tohoku journal of experimental medicine 2017;242(2):93-100. | Does not meet full eligibility criteria: includes people without cirrhosis |
| Hu, X., Huang, F., Yao, J., Lv, J., Mai, J., Li, N. Cross-sectional study on the diagnostic significance of plasma exosomal miRNAs in HBV-related hepatocellular carcinoma. Journal of Translational Medicine 2024;22(1):1006. | Exclude on population: not cirrhosis |
| Hu, Y., Hua, D., Cheng, Z. H., Wu, Y. Y., Xie, Q. G., Wang, Q. Y., Yu, J. Clinical value of methylation of plasma adenomatous polyposis coli gene in the molecular diagnosis of hepatocellular carcinoma. Tumor 2011;31(10):924-29. | Foreign language |
| Hu, Z., Chen, H., Chen, S., Huang, Z., Qin, S., Zhong, J., Qin, X. The value of neutrophil to lymphocyte ratio and gamma-glutamyl transpeptidase to platelet ratio in patients with hepatocellular carcinoma. Medicine 2019;98(9):e14749. | Duplicate (including Cochrane includes) |
| Hu, Zuojian, Chen, Huaping, Chen, Siyuan, Huang, Zhili, Qin, Shanzi, Zhong, Jianing, Qin, Xue. The value of neutrophil to lymphocyte ratio and gamma-glutamyl transpeptidase to platelet ratio in patients with hepatocellular carcinoma. Medicine 2019;98(9):e14749. | Does not meet full eligibility criteria: includes people without cirrhosis |
| Huang, Ao, Zhang, Xin, Zhou, Shao-Lai, Cao, Ya, Huang, Xiao-Wu, Fan, Jia, Yang, Xin-Rong. Plasma Circulating Cell-free DNA Integrity as a Promising Biomarker for Diagnosis and Surveillance in Patients with Hepatocellular Carcinoma. Journal of Cancer 2016;7(13):1798-8003. | Exclude on population: not cirrhosis |
| Huang, Chenjun, Fang, Meng, Feng, Huijuan, Liu, Lijuan, Li, Ya, Xu, Xuewen, et al. N-glycan fingerprint predicts alpha-fetoprotein negative hepatocellular carcinoma: A large-scale multicenter study. International journal of cancer 2021;149(3):717-27. | Exclude on population: healthy controls |
| Huang, Chenjun, Fang, Meng, Xiao, Xiao, Wang, Hong, Gao, Zhiyuan, Ji, Jun, et al. Validation of the GALAD model for early diagnosis and monitoring of hepatocellular carcinoma in Chinese multicenter study. Liver international : official journal of the International Association for the Study of the Liver 2022;42(1):210-23. | Exclude on population: not cirrhosis |
| Huang, Daniel Q., Fowler, Kathryn J., Liau, Joy, Cunha, Guilherme M., Louie, Ashley L., An, Julie Y., et al. Comparative efficacy of an optimal exam between ultrasound versus abbreviated MRI for HCC screening in NAFLD cirrhosis: A prospective study. Alimentary pharmacology & therapeutics 2022;55(7):820-27. | Exclude on study design: not a 1- or 2-gate test accuracy study |
| Huang, F., Bai, J., Hu, L., Luo, C. Serum DLAT Is a Potential Diagnostic Marker in AFP-Negative HCC. Biological and Pharmaceutical Bulletin 2024;47(12):2127 “ 2137. | Retracted |
| Huang, F., Jiang, H., Shen, M., Zhang, C., Chen, Y., Pan, B., et al. Plasma metabolomic profiling for hepatocellular carcinoma diagnosis and microvascular invasion prediction. International Journal of Cancer 2025;():n. pag.. | Does not meet full eligibility criteria: includes people without cirrhosis |
| Huang, Gengming, Krocker, Joseph D., Kirk, Jason L., Merwat, Shehzad N., Ju, Hyunsu, Soloway, Roger D., et al. Evaluation of INK4A promoter methylation using pyrosequencing and circulating cell-free DNA from patients with hepatocellular carcinoma. Clinical chemistry and laboratory medicine 2014;52(6):899-909. | Exclude on population: not cirrhosis |
| Huang, H., Sun, X., Zhang, Q., Liu, C., Cao, X., Zhang, D., Wang, G. Combined serum IFN-gamma and IL-22 levels as predictive biomarkers for hepatocellular carcinoma risk: A clinical investigation. Biomedical Reports 2025;23(3):149. | No 2x2 data |
| Huang, Jinlan, Zheng, Yansong, Xiao, Xialin, Liu, Can, Lin, Jinpiao, Zheng, Sijia, Yang, Bin. A Circulating Long Noncoding RNA Panel Serves as a Diagnostic Marker for Hepatocellular Carcinoma. Disease markers 2020;2020():5417598. | Exclude on population: healthy controls |

| Reference | Reason for exclusion |
|---|---|
| Huang, Li, Mo, Zhuning, Hu, Zuojian, Zhang, Linyan, Qin, Shanzi, Qin, Xue. Diagnostic value of fibrinogen to prealbumin ratio and gamma-glutamyl transpeptidase to platelet ratio in the progression of AFP-negative hepatocellular carcinoma. Cancer cell international 2020;20():77. | Exclude on index test |
| Huang, Shujing, Jiang, Feifei, Wang, Ying, Yu, Yanhua, Ren, Siqian, Wang, Xiaowei, Yin, Peng. Diagnostic performance of tumor markers AFP and PIVKA-II in Chinese hepatocellular carcinoma patients. Tumour biology : the journal of the International Society for Oncodevelopmental Biology and Medicine 2017;39(6):1010428317705763. | Exclude on population: not cirrhosis |
| Huang, W., Li, T., Yang, W., Chai, X., Chen, K., Wei, L., et al. Analysis of DNA methylation in plasma for monitoring hepatocarcinogenesis. Genetic Testing and Molecular Biomarkers 2015;19(6):295-302. | Does not meet full eligibility criteria: includes people without cirrhosis |
| Huang, Wei-Jian, He, Wei-Ying, Li, Jian-Di, He, Rong-Quan, Huang, Zhi-Guang, Zhou, Xian-Guo, et al. Clinical significance and molecular mechanism of angiotensin-converting enzyme 2 in hepatocellular carcinoma tissues. Bioengineered 2021;12(1):4054-69. | Exclude on population: not cirrhosis |
| Huang, X. H., Sun, L. H., Lu, D. D., Sun, Y., Ma, L. J., Zhang, X. R., Huang, J. Codon 249 mutation in exon 7 of p53 gene in plasma DNA: Maybe a new early diagnostic marker of hepatocellular carcinoma in Qidong risk area, China. World Journal of Gastroenterology 2003;9(4):692-95. | Does not meet full eligibility criteria: includes people without cirrhosis |
| Huang, Xuejing, Sun, Liyuan, Wen, Sha, Deng, Deli, Wan, Fengjie, He, Xiao, et al. RNA sequencing of plasma exosomes revealed novel functional long noncoding RNAs in hepatocellular carcinoma. Cancer science 2020;111(9):3338-49. | Does not meet full eligibility criteria: includes people without cirrhosis |
| Huang, Y. C., Huang, C. F., Chang, K. C., Hung, S. F., Wang, J. H., Hung, C. H., et al. Community-based screening for hepatocellular carcinoma in elderly residents in a hepatitis B- and C-endemic area. Journal of Gastroenterology and Hepatology (Australia) 2011;26(1):129-34. | No 2x2 data |
| Huang, Ya-Hui, Liang, Kung-Hao, Chien, Rong-Nan, Hu, Tsung-Hui, Lin, Kwang-Huei, Hsu, Chao-Wei, et al. A Circulating MicroRNA Signature Capable of Assessing the Risk of Hepatocellular Carcinoma in Cirrhotic Patients. Scientific reports 2017;7(1):523. | Exclude on study design: not a 1- or 2-gate test accuracy study |
| Huang, Yi, Zhang, Songgao, Zheng, Qingzhu, Li, Yadong, Yu, Lili, Wu, Qingwei, et al. Development of up-converting phosphor technology-based lateral flow assay for quantitative detection of serum PIVKA-II: Inception of a near-patient PIVKA-II detection tool. Clinica chimica acta; international journal of clinical chemistry 2019;488():202-08. | Exclude on population: clinical signs and symptoms of HCC |
| Huang, Yifan, Zhou, Shiyue, Zhu, Jianhui, Lubman, David M. LC-MS/MS isomeric profiling of permethylated N-glycans derived from serum haptoglobin of hepatocellular carcinoma (HCC) and cirrhotic patients. Electrophoresis 2017;38(17):2160-67. | Exclude on index test |
| Huang, Zhao-Hui, Hu, Yu, Hua, Dong, Wu, Yu-Yu, Song, Ming-Xu. Quantitative analysis of multiple methylated genes in plasma for the diagnosis and prognosis of hepatocellular carcinoma. Experimental and molecular pathology 2011;91(3):702-07. | Does not meet full eligibility criteria: includes people without cirrhosis |
| Huang, Zhaohui, Hua, Dong, Hu, Yu, Cheng, Zhihong, Zhou, Xike, Xie, Qigen, et al. Quantitation of plasma circulating DNA using quantitative PCR for the detection of hepatocellular carcinoma. Pathology oncology research : POR 2012;18(2):271-76. | Does not meet full eligibility criteria: includes people without cirrhosis |
| Huber, Adrian Thomas, Schuster, Frederik, Ebner, Lukas, Butikofer, Yanik, Ott, Daniel, Leidolt, Lars, et al. Hepatocellular Carcinoma Screening With Computed Tomography Using the Arterial Enhancement Fraction With Radiologic-Pathologic Correlation. Investigative radiology 2016;51(1):25-32. | Exclude on population: HCC participants not treatment-naive |
| Huber, K., Kirchheimer, J. C., Ermler, D., Bell, C. Determination of plasma urokinase-type plasminogen activator antigen in patients with primary liver cancer: characterization as tumor-associated antigen and comparison with alpha-fetoprotein. Cancer research 1992;52(7):1717-20. | Does not meet full eligibility criteria: pre-2005 |
| Hui, Anselm Wang-Hei, Lau, Hon-Wai, Cao, Cyanne Ye, Zhou, Jun-Wei, Lai, Paul Bo-San. Downregulation of PLZF in human hepatocellular carcinoma and its clinical significance. Oncology reports 2015;33(1):397-402. | Exclude on population: tissue samples |

| Reference | Reason for exclusion |
|---|---|
| Hung, Chao-Hung, Hu, Tsung-Hui, Lu, Sheng-Nan, Kuo, Fang-Ying, Chen, Chien-Hung, Wang, Jing-Houng, et al. Circulating microRNAs as biomarkers for diagnosis of early hepatocellular carcinoma associated with hepatitis B virus. International journal of cancer 2016;138(3):714-20. | Does not meet full eligibility criteria: includes people without cirrhosis |
| Huo, Li, Guo, Jinxia, Dang, Yonghong, Lv, Jinqiao, Zheng, Youjing, Li, Fang, Xie, Qingguo. Kinetic analysis of dynamic (11)C-acetate PET/CT imaging as a potential method for differentiation of hepatocellular carcinoma and benign liver lesions. Theranostics 2015;5(4):371-77. | Exclude on population: clinical signs and symptoms of HCC |
| Huo, Teh-Ia, Hsia, Cheng-Yuan, Chu, Chi-Jen, Huang, Yi-Hsiang, Lui, Wing-Yiu, Wu, Jaw-Ching, et al. The predictive ability of serum alpha-fetoprotein for hepatocellular carcinoma is linked with the characteristics of the target population at surveillance. Journal of surgical oncology 2007;95(8):645-51. | Does not meet full eligibility criteria: includes people without cirrhosis |
| Huo, Teh-Ia, Hsia, Cheng-Yuan, Chu, Chi-Jen, Huang, Yi-Hsiang, Lui, Wing-Yiu, Wu, Jaw-Ching, et al. The predictive ability of serum alpha-fetoprotein for hepatocellular carcinoma is linked with the characteristics of the target population at surveillance. Journal of surgical oncology 2007;95(8):645-51. | Exclude on population: HCC participants not treatment-naive |
| Hussain, Hero K., Syed, Ibrahim, Nghiem, Hanh V., Johnson, Timothy D., Carlos, Ruth C., Weadock, William J. T2-weighted MR imaging in the assessment of cirrhotic liver. Radiology 2004;230(3):637-44. | Does not meet full eligibility criteria: pre-2005 |
| Hussein, M. M., Ibrahim, A. A., Abdella, H. M., Montasser, I. F. Evaluation of serum squamous cell carcinoma antigen as a novel biomarker for diagnosis of hepatocellular carcinoma in Egyptian patients. Indian journal of cancer 2008;45(4):167-72. | Exclude on population: not cirrhosis |
| Hwang, J. S., Lee, S., Kim, G., Jeong, H., Kwon, K., Jung, E., et al. A serum exosomal microRNA-based artificial intelligence diagnostic model for highly accurate detection of hepatocellular carcinoma. Cancer communications (London, England) 2025;():n. pag.. | Exclude on population: not adults |
| Hwang, Jiyoung, Kim, Young Kon, Kim, Jong Man, Lee, Won Jae, Choi, Dongil. Pretransplant diagnosis of hepatocellular carcinoma by gadoxetic acid-enhanced and diffusion-weighted magnetic resonance imaging. Liver transplantation : official publication of the American Association for the Study of Liver Diseases and the International Liver Transplantation Society 2014;20(12):1436-46. | Does not meet full eligibility criteria: includes people without cirrhosis |
| Hwang, Sung Ho, Yu, Jeong-Sik, Kim, Ki Whang, Kim, Joo Hee. Small hypervascular enhancing lesions on arterial phase images of multiphase dynamic computed tomography in cirrhotic liver: fate and implications. Journal of computer assisted tomography 2008;32(1):39-45. | Exclude on population: clinical signs and symptoms of HCC |
| Hyodo, I., Jinno, K., Tanimizu, M., Hosokawa, Y., Nishikawa, Y., Akiyama, M., Mandai, K. Detection of circulating intercellular adhesion molecule-1 in hepatocellular carcinoma. International journal of cancer 1993;55(5):775-79. | No 2x2 data |
| Iacovazzi, P. A., Trisolini, A., Barletta, D., Elba, S., Manghisi, O. G. Serum 90K/MAC-2BP glycoprotein in patients with liver cirrhosis and hepatocellular carcinoma: a comparison with alpha-fetoprotein. Clinical chemistry and laboratory medicine 2001;39(10):961-65. | Exclude on population: HCC participants not treatment-naive |
| Iannaccone, Riccardo, Laghi, Andrea, Catalano, Carlo, Rossi, Plinio, Mangiapane, Filippo, Murakami, Takamichi, et al. Hepatocellular carcinoma: role of unenhanced and delayed phase multi-detector row helical CT in patients with cirrhosis. Radiology 2005;234(2):460-67. | Exclude on population: clinical signs and symptoms of HCC |
| Iavarone, M., Lampertico, P., Ronchi, G., Del Ninno, E., Zanella, A. A prospective study of blood alpha-fetoprotein messenger RNA as a predictor of hepatocellular carcinoma in patients with cirrhosis. Journal of viral hepatitis 2003;10(6):423-26. | Exclude on study design: not a 1- or 2-gate test accuracy study |
| Ibrahim A.M., Hashem M.E., Mostafa E.F., Refaey M.M., Hamed E.F., Ibrahim I. Annexin A2 versus Afp as an efficient diagnostic serum marker for hepatocellular carcinoma. Journal of Gastroenterology and Hepatology Research 2013;2(9):780-85. | Exclude on population: healthy controls |
| Ibrahim, A. M., Hashem, M. E., Mostafa, E. F., Refaey, M. M., Hamed, E. F., Ibrahim, I. Annexin A2 versus Afp as an efficient diagnostic serum marker for hepatocellular carcinoma. Journal of Gastroenterology and Hepatology Research 2013;2(9):780-85. | Duplicate (including Cochrane includes) |

| Reference | Reason for exclusion |
|---|---|
| Ibrahim, Gehan H. & Mahmoud, Moushira A. Evaluation of circulating Transforming growth factor-beta1, Glypican-3 and Golgi protein-73 mRNAs expression as predictive markers for hepatocellular carcinoma in Egyptian patients. Molecular biology reports 2013;40(12):7069-75. | Exclude on population: not cirrhosis |
| Ichikawa, Shintaro, Motosugi, Utaroh, Shimizu, Tatsuya, Kromrey, Marie Luise, Aikawa, Yoshihito, Tamada, Daiki. Diagnostic performance and image quality of low-tube voltage and low-contrast medium dose protocol with hybrid iterative reconstruction for hepatic dynamic CT. The British journal of radiology 2021;94(1128):20210601. | Exclude on population: not cirrhosis |
| Ichikawa, Tomoaki & Sano, Katsuhiro. Diagnosis of Pathologically Early HCC with EOB-MRI: Experiences and Current Consensus. Liver cancer 2014;3(2):97-107. | Exclude on population: clinical signs and symptoms of HCC |
| Idriss, Naglaa K., Fakhry, Michel, Imam, Hala M., Abd-Elmoez, Fatema A., Abdelewahab, Hossam, Abdel-Wahid, Lobna, et al. Analysis of Lamin B1, Vimentin and Anti-Ku86 as Prospective Biomarkers of Hepatocellular Carcinoma in Patients with Hepatitis C Virus Infection. Cellular physiology and biochemistry : international journal of experimental cellular physiology, biochemistry, and pharmacology 2019;52(3):595-605. | Does not meet full eligibility criteria: includes people without cirrhosis |
| Iizuka, Norio, Oka, Masaaki, Sakaida, Isao, Moribe, Toyoki, Miura, Toshiaki, Kimura, Naoki, et al. Efficient detection of hepatocellular carcinoma by a hybrid blood test of epigenetic and classical protein markers. Clinica chimica acta; international journal of clinical chemistry 2011;412(1-2):152-8. | Does not meet full eligibility criteria: includes people without cirrhosis |
| Iizuka, Norio, Oka, Masaaki, Sakaida, Isao, Moribe, Toyoki, Miura, Toshiaki, Kimura, Naoki, et al. Efficient detection of hepatocellular carcinoma by a hybrid blood test of epigenetic and classical protein markers. Clinica chimica acta; international journal of clinical chemistry 2011;412(1-2):152-58. | Duplicate (including Cochrane includes) |
| Iizuka, Norio, Sakaida, Isao, Moribe, Toyoki, Fujita, Nozomi, Miura, Toshiaki, Stark, Markus, et al. Elevated levels of circulating cell-free DNA in the blood of patients with hepatitis C virus-associated hepatocellular carcinoma. Anticancer research 2006;26(6C):4713-19. | Exclude on population: not cirrhosis |
| Ijuin, Sho, Oda, Kohei, Mawatari, Seiichi, Taniyama, Ohki, Toyodome, Ai, Sakae, Haruka, et al. Serine palmitoyltransferase long chain subunit 3 is associated with hepatocellular carcinoma in patients with NAFLD. Molecular and clinical oncology 2022;16(2):55. | Exclude on population: not cirrhosis |
| Ikoma, Jiro, Kaito, Masahiko, Ishihara, Tomoaki, Nakagawa, Naoki, Kamei, Akira, Fujita, Naoki, et al. Early diagnosis of hepatocellular carcinoma using a sensitive assay for serum des-gamma-carboxy prothrombin: a prospective study. Hepato-gastroenterology 2002;49(43):235-38. | Does not meet full eligibility criteria: pre-2005 |
| Ilikhan, S. U., Bilici, M., Sahin, H., Akca, A. S. D., Can, M., Oz, I. I., et al. Assessment of the correlation between serum prolidase and alpha-fetoprotein levels in patients with hepatocellular carcinoma. World Journal of Gastroenterology 2016;22(22):6999-7007. | Exclude on population: healthy controls |
| Imai, Y., Taira, J. I., Okada, M., Ando, M., Sano, T., Miyata, Y., et al. The close linkage between the elasticity modulus measured by real-time mapping shear wave elastography and the presence of hepatocellular carcinoma in patients with a sustained virological response to interferon for chronic hepatitis C. Journal of Medical Ultrasonics 2015;42(3):341-47. | Exclude on population: not cirrhosis |
| Inoue, T., Funaki, N., Hatahara, T., Igarashi, S., Kaneko, E. [Assay of serum collagen markers in chronic liver diseases and liver cancer]. Nihon Shokakibyo Gakkai zasshi = The Japanese journal of gastro-enterology 1990;87(7):1506-13. | Does not meet full eligibility criteria: pre-2005 |
| Inoue, Takako. [Clinical Significance of Novel Serum Biomarkers in the Management of Liver Diseases]. Rinsho byori. The Japanese journal of clinical pathology 2017;65(1):83-91. | Foreign language |
| Ishida, Haku & Matsuo, Shuji. [Evaluation of diagnostic performance of alpha-fetoprotein (AFP) and des-gamma-carboxy prothrombin (DCP) for HCV related hepatocellular carcinoma developed after long-term follow up]. Rinsho byori. The Japanese journal of clinical pathology 2010;58(11):1065-72. | Foreign language |

| Reference | Reason for exclusion |
| --- | --- |
| Ishii, M, Gama, H, Chida, N, Ueno, Y, Shinzawa, H, Takagi, T, et al. Simultaneous measurements of serum alpha-fetoprotein and protein induced by vitamin K absence for detecting hepatocellular carcinoma. South Tohoku District Study Group. The American journal of gastroenterology 2000;95(4):1036-40. | Does not meet full eligibility criteria: includes people without cirrhosis |
| Ishii, M., Gama, H., Chida, N., Ueno, Y., Shinzawa, H., Takagi, T., et al. Simultaneous measurements of serum alpha-fetoprotein and protein induced by vitamin K absence for detecting hepatocellular carcinoma. American Journal of Gastroenterology 2000;95(4):1036-40. | Does not meet full eligibility criteria: includes people without cirrhosis |
| Ishizuka, H., Nakayama, T., Matsuoka, S., Gotoh, I., Ogawa, M., Suzuki, K., et al. Prediction of the development of hepato-cellular-carcinoma in patients with liver cirrhosis by the serial determinations of serum alpha-L-fucosidase activity. Internal medicine (Tokyo, Japan) 1999;38(12):927-31. | Exclude on index test |
| Ismail, Manar M, Morsi, Heba K, Abdulateef, Nahla A B, Noaman, Maissa K. Evaluation of prothrombin induced by vitamin K absence, macrophage migration inhibitory factor and Golgi protein-73 versus alpha fetoprotein for hepatocellular carcinoma diagnosis and surveillance. Scandinavian journal of clinical and laboratory investigation 2017;77(3):175-83. | Exclude on population: not cirrhosis |
| Ismail, Manar M., Morsi, Heba K., Abdulateef, Nahla A. B., Noaman, Maissa K. Evaluation of prothrombin induced by vitamin K absence, macrophage migration inhibitory factor and Golgi protein-73 versus alpha fetoprotein for hepatocellular carcinoma diagnosis and surveillance. Scandinavian journal of clinical and laboratory investigation 2017;77(3):175-83. | Duplicate (including Cochrane includes) |
| Ismail, Saber A, El Saadany, Sherif, Ziada, Dina H, Zakaria, Soha S, Mayah, Wael W, Elashry, Heba, Arafa, Mona. Cytokeratin-18 in Diagnosis of HCC in Patients with Liver Cirrhosis. Asian Pacific journal of cancer prevention : APJCP 2017;18(4):1105-11. | Duplicate (including Cochrane includes) |
| Ismail, Saber A., El Saadany, Sherif, Ziada, Dina H., Zakaria, Soha S., Mayah, Wael W., Elashry, Heba, Arafa, Mona. Cytokeratin-18 in Diagnosis of HCC in Patients with Liver Cirrhosis. Asian Pacific journal of cancer prevention : APJCP 2017;18(4):1105-11. | Continuous test without threshold |
| Ismail, Saber, Mayah, Wael, Battia, Hassan El, Gaballah, Hanaa, Jiman-Fatani, Asif, Hamouda, Hala, et al. Plasma nuclear factor kappa B and serum peroxiredoxin 3 in early diagnosis of hepatocellular carcinoma. Asian Pacific journal of cancer prevention : APJCP 2015;16(4):1657-63. | Exclude on population: healthy controls |
| Iyer A., Devdas K., Sreesh S., Sathar S., Sandesh K. To assess the diagnostic accuracy of HCC-Alpha fetoprotein-routine test (HCC-ART) score based on age, AFP, aspartate aminotransferase (AST)/alanine aminotransferase (ALT) ratio, alkaline phosphatase (ALP), and albumin (Alb) for early detection of hepatocellular carcinoma in cirrhotic patients. Hepatology International 2018;12(2):S396. | CONFERENCE ABSTRACT |
| Izuno, K., Fujiyama, S., Yamasaki, K., Sato, M. Early detection of hepatocellular carcinoma associated with cirrhosis by combined assay of des-gamma-carboxy prothrombin and alpha-fetoprotein: a prospective study. Hepato-gastroenterology 1995;42(4):387-93. | Does not meet full eligibility criteria: pre-2005 |
| Izzo, F, Cremona, F, Delrio, P, Leonardi, E, Castello, G, Pignata, S, Daniele, B. Soluble interleukin-2 receptor levels in hepatocellular cancer: a more sensitive marker than alfa fetoprotein. Annals of surgical oncology 1999;6(2):178-85. | Exclude on population: not cirrhosis |
| Izzo, F., Cremona, F., Ruffolo, F., Palaia, R., Parisi, V. Detection of hepatocellular cancer during screening of 1125 patients with chronic hepatitis virus infection. Journal of chemotherapy (Florence, Italy) 1997;9(2):151-52. | Exclude on population: not cirrhosis |
| Jain, Surbhi, Chen, Sitong, Chang, Kung-Chao, Lin, Yih-Jyh, Hu, Chi-Tan, Boldbaatar, Batbold, et al. Impact of the location of CpG methylation within the GSTP1 gene on its specificity as a DNA marker for hepatocellular carcinoma. PloS one 2012;7(4):e35789. | Exclude on population: not cirrhosis |
| Jalal, P. J., King, B. J., Saeed, A., Adedeji, Y., Mason, C. P., Ball, J. K., et al. Elevated serum activity of MBL and ficolin-2 as biomarkers for progression to hepatocellular carcinoma in chronic HCV infection. Virology 2019;530():99-106. | Exclude on index test |
| Jalli, Reza, Jafari, Seyed Hamed, Sefidbakht, Sepideh. Comparison of the Accuracy of DWI and Ultrasonography in Screening Hepatocellular Carcinoma | Duplicate (including Cochrane includes) |

| Reference | Reason for exclusion |
|---|---|
| in Patients With Chronic Liver Disease. Iranian journal of radiology : a quarterly journal published by the Iranian Radiological Society 2015;12(1):e12708. | |
| Jang, Eun Sun, Jeong, Sook-Hyang, Kim, Jin-Wook, Choi, Yun Suk, Leissner, Philippe. Diagnostic Performance of Alpha-Fetoprotein, Protein Induced by Vitamin K Absence, Osteopontin, Dickkopf-1 and Its Combinations for Hepatocellular Carcinoma. PloS one 2016;11(3):e0151069. | Does not meet full eligibility criteria: includes people without cirrhosis |
| Jang, Eun Sun, Jeong, Sook-Hyang, Kim, Jin-Wook, Choi, Yun Suk, Leissner, Philippe. Diagnostic Performance of Alpha-Fetoprotein, Protein Induced by Vitamin K Absence, Osteopontin, Dickkopf-1 and Its Combinations for Hepatocellular Carcinoma. PloS one 2016;11(3):e0151069. | Duplicate (including Cochrane includes) |
| Jang, Hyun-Jung, Kim, Tae Kyoung, Khalili, Korosh, Yazdi, Leyla, Menezes, Ravi, Park, Seong Ho. Characterization of 1-to 2-cm liver nodules detected on hcc surveillance ultrasound according to the criteria of the American Association for the Study of Liver Disease: is quadriphasic CT necessary? AJR. American journal of roentgenology 2013;201(2):314-21. | Exclude on population: clinical signs and symptoms of HCC |
| Jang, Tyng-Yuan. Cutoff values of protein induced by vitamin K absence or antagonist II for diagnosing hepatocellular carcinoma. Medicine 2022;101(39):e30936. | Exclude on population: clinical signs and symptoms of HCC |
| Janssen, S. Detection of hepatocellular carcinoma with ferucarbotran-enhanced dynamic MR imaging. Zeitschrift fur Gastroenterologie 2007;45(2):164-66. | Exclude on population: clinical signs and symptoms of HCC |
| Jasirwan, Chyntia Olivia Maurine, Fahira, Alessa, Siregar, Lianda. The alpha-fetoprotein serum is still reliable as a biomarker for the surveillance of hepatocellular carcinoma in Indonesia. BMC gastroenterology 2020;20(1):215. | Exclude on population: not cirrhosis |
| Jeng, Jen-Eing, Wu, Hui-Fang, Tsai, Meng-Feng, Tsai, Huey-Ru, Chuang, Lea-Yea, Lin, Zu-Yau, et al. Independent and additive interaction between tumor necrosis factor beta +252 polymorphisms and chronic hepatitis B and C virus infection on risk and prognosis of hepatocellular carcinoma: a case-control study. Asian Pacific journal of cancer prevention : APJCP 2014;15(23):10209-15. | Exclude on study design: not a 1- or 2-gate test accuracy study |
| Jeon, Yejoo, Jang, Eun Sun, Choi, Yun Suk, Kim, Jin-Wook. Glypican-3 level assessed by the enzyme-linked immunosorbent assay is inferior to alpha-fetoprotein level for hepatocellular carcinoma diagnosis. Clinical and molecular hepatology 2016;22(3):359-65. | Does not meet full eligibility criteria: includes people without cirrhosis |
| Jeon, Yejoo, Jang, Eun Sun, Choi, Yun Suk, Kim, Jin-Wook. Glypican-3 level assessed by the enzyme-linked immunosorbent assay is inferior to alpha-fetoprotein level for hepatocellular carcinoma diagnosis. Clinical and molecular hepatology 2016;22(3):359-65. | Duplicate (including Cochrane includes) |
| Jhaveri, Kartik S., Babaei Jandaghi, Ali, Bhayana, Rajesh, Elbanna, Khaled Y., Espin-Garcia, Osvaldo, Fischer, Sandra E., Ghanekar, Anand. Prospective evaluation of Gadoxetate-enhanced magnetic resonance imaging and computed tomography for hepatocellular carcinoma detection and transplant eligibility assessment with explant histopathology correlation. Cancer imaging : the official publication of the International Cancer Imaging Society 2023;23(1):22. | Exclude on population: clinical signs and symptoms of HCC |
| Ji, Dong, Chen, Guo-Feng, Niu, Xiao-Xia, Zhang, Mingjie, Wang, Cheng, Shao, Qing, et al. Non-alcoholic fatty liver disease is a risk factor for occurrence of hepatocellular carcinoma after sustained virologic response in chronic hepatitis C patients: A prospective four-years follow-up study. Metabolism open 2021;10():100090. | Exclude on population: not cirrhosis |
| Ji, Jun, Wang, Hao, Li, Yan, Zheng, Lei, Yin, Yuepeng, Zou, Zhenzhen, et al. Diagnostic Evaluation of Des-Gamma-Carboxy Prothrombin versus alpha-Fetoprotein for Hepatitis B Virus-Related Hepatocellular Carcinoma in China: A Large-Scale, Multicentre Study. PloS one 2016;11(4):e0153227. | Does not meet full eligibility criteria: includes people without cirrhosis |
| Ji, Jun, Wang, Hao, Li, Yan, Zheng, Lei, Yin, Yuepeng, Zou, Zhenzhen, et al. Diagnostic Evaluation of Des-Gamma-Carboxy Prothrombin versus alpha-Fetoprotein for Hepatitis B Virus-Related Hepatocellular Carcinoma in China: A Large-Scale, Multicentre Study. PloS one 2016;11(4):e0153227. | Duplicate (including Cochrane includes) |

| Reference | Reason for exclusion |
|---|---|
| Ji, Shuwen & Wang, Ziyong. Application of ultrasound combined with enhanced MRI by Gd-BOPTA in diagnosing hepatocellular carcinoma. American journal of translational research 2021;13(6):7172-78. | Exclude on population: clinical signs and symptoms of HCC |
| Ji, Xiang-Fen, Fan, Yu-Chen, Gao, Shuai, Yang, Yang, Zhang, Jian-Jun. MT1M and MT1G promoter methylation as biomarkers for hepatocellular carcinoma. World journal of gastroenterology 2014;20(16):4723-29. | Exclude on population: not cirrhosis |
| Jia, H. L., Ye, Q. H., Qin, L. X., Budhu, A., Forgues, M., Chen, Y., et al. Gene expression profiling reveals potential biomarkers of human hepatocellular carcinoma. Clinical Cancer Research 2007;13(4):1133-39. | Does not meet full eligibility criteria: includes people without cirrhosis |
| Jia, Jian-An, Zhang, Shuqin, Bai, Xin, Fang, Meng, Chen, Shipeng, Liang, Xiaotao, et al. Sparse logistic regression revealed the associations between HBV PreS quasispecies and hepatocellular carcinoma. Virology journal 2022;19(1):114. | Exclude on population: not cirrhosis |
| Jia, K. D. & Shi, S. X. Values of reactive oxygen toxic species of finger blood dot in the diagnosis of hepatocellular carcinoma. World Chinese Journal of Digestology 2006;14(24):2406-10. | Foreign language |
| Jia, Ke Dong & Zou, Zheng Yu. The Value of Survivin Gene and Proliferation of Hepatocytes in Screening for Hepatocellular Carcinoma. Gastroenterology research 2009;2(6):333-37. | Exclude on population: tissue samples |
| Jia, Xiaobo, Gao, Yingtang, Zhai, Daokuan, Liu, Jiao, Cai, Junjun, Wang, Yajie, Jing, Li. Assessment of the Clinical Utility of Glypican 3 as a Serum Marker for the Diagnosis of Hepatocellular Carcinoma. Technology in cancer research & treatment 2016;15(6):780-86. | Does not meet full eligibility criteria: includes people without cirrhosis |
| Jia, Zhiling, Wang, Li, Liu, Chang, Yu, Zhonghe, Chai, Lina. Evaluation of alpha-fetoprotein-L3 and Golgi protein 73 detection in diagnosis of hepatocellular carcinoma. Contemporary oncology (Poznan, Poland) 2014;18(3):192-96. | Exclude on population: not cirrhosis |
| Jiang, Chao, Zhang, Yun, Yu, Hai-Feng, Yu, Xiao-Tian, Zhou, Su-Jun. Expression of ADAM8 and its clinical values in diagnosis and prognosis of hepatocellular carcinoma. Tumour biology : the journal of the International Society for Oncodevelopmental Biology and Medicine 2012;33(6):2167-72. | No 2x2 data |
| Jiang, H. J., Xu, K., Bai, R. J., Liu, B. L., Wang, D. Quantitative study of hepatic dynamic flow in hepatocellular carcinoma by multi-slice CT perfusion. Chinese Journal of Medical Imaging Technology 2007;23(5):711-14. | Foreign language |
| Jiang, Hanyu, Liu, Xijiao, Chen, Jie, Wei, Yi, Lee, Jeong Min, Cao, Likun, et al. Man or machine? Prospective comparison of the version 2018 EASL, LI-RADS criteria and a radiomics model to diagnose hepatocellular carcinoma. Cancer imaging : the official publication of the International Cancer Imaging Society 2019;19(1):84. | Exclude on population: clinical signs and symptoms of HCC |
| Jiang, Hao, Chen, Jing, Jiang, Jia-ji. [Diagnostic value of contrast-enhanced ultrasound for small hepatocellular carcinoma in cirrhotic patients: a meta-analysis]. Zhonghua gan zang bing za zhi = Zhonghua ganzangbing zazhi = Chinese journal of hepatology 2012;20(11):828-32. | Exclude on study design: not a 1- or 2-gate test accuracy study |
| Jiang, Jingting, Wu, Changping, Shen, Yueping, Xu, Bin, Zheng, Xiao, Li, Xiaodong. Clinical application of determining serum AFP-IgM complexes for diagnosis of small hepatocellular carcinoma. Anticancer research 2011;31(2):687-91. | Exclude on population: not cirrhosis |
| Jiang, L., Li, X., Cheng, Q. Plasma microRNA might as a potential biomarker for hepatocellular carcinoma and chronic liver disease screening. Tumor Biology 2015;36(9):7167-74. | Exclude on population: not cirrhosis |
| Jiang, Li, Cheng, Qi, Zhang, Bin-Hao. Circulating microRNAs as biomarkers in hepatocellular carcinoma screening: a validation set from China. Medicine 2015;94(10):e603. | Exclude on population: not cirrhosis |
| Jiang, Peiyong, Chan, Carol W. M., Chan, K. C. Allen, Cheng, Suk Hang, Wong, John, Wong, Vincent Wai-Sun, et al. Lengthening and shortening of plasma DNA in hepatocellular carcinoma patients. Proceedings of the National Academy of Sciences of the United States of America 2015;112(11):E1317-25. | Does not meet full eligibility criteria: includes people without cirrhosis |

| Reference | Reason for exclusion |
|---|---|
| Jiang, Yandong, Zhang, Meng, Zhu, Yanting. Diagnostic role of contrast-enhanced ultrasonography versus conventional B-mode ultrasonography in cirrhotic patients with early hepatocellular carcinoma: a retrospective study. Journal of gastrointestinal oncology 2021;12(5):2403-11. | Exclude on population: clinical signs and symptoms of HCC |
| Jiang, Yingying, Tie, Cai, Wang, Yang, Bian, Dandan, Liu, Mei, Wang, Ting, et al. Upregulation of Serum Sphingosine (d18:1)-1-P Potentially Contributes to Distinguish HCC Including AFP-Negative HCC From Cirrhosis. Frontiers in oncology 2020;10():1759. | Does not meet full eligibility criteria: includes people without cirrhosis |
| Jiao, Congcong, Cui, Lianhua, Piao, Jinmei, Qi, Yunpeng. Clinical significance and expression of serum Golgi protein 73 in primary hepatocellular carcinoma. Journal of cancer research and therapeutics 2018;14(6):1239-44. | Exclude on population: not cirrhosis |
| Jiao, Congcong, Cui, Lianhua, Piao, Jinmei, Qi, Yunpeng. Clinical significance and expression of serum Golgi protein 73 in primary hepatocellular carcinoma. Journal of cancer research and therapeutics 2018;14(6):1239-44. | Duplicate (including Cochrane includes) |
| Jiao, Jingjing, Sanchez, Jessica I., Thompson, Erika J., Mao, Xizeng, McCormick, Joseph B., Fisher-Hoch, Susan P., et al. Somatic Mutations in Circulating Cell-Free DNA and Risk for Hepatocellular Carcinoma in Hispanics. International journal of molecular sciences 2021;22(14):n. pag.. | Exclude on population: not cirrhosis |
| Jin, Guang-Zhi, Li, Yan, Cong, Wen-Ming, Yu, Hua, Dong, Hui, Shu, Hong, et al. iTRAQ-2DLC-ESI-MS/MS based identification of a new set of immunohistochemical biomarkers for classification of dysplastic nodules and small hepatocellular carcinoma. Journal of proteome research 2011;10(8):3418-28. | Does not meet full eligibility criteria: includes people without cirrhosis |
| Jin, J., Zhang, X. Y., Shi, J. L., Xue, X. F., Lu, L. L., Lu, J. H., et al. Application of AFP whole blood one-step rapid detection kit in screening for HCC in Qidong. American Journal of Cancer Research 2017;7(6):1384-88. | Exclude on population: not cirrhosis |
| Jin, K., Wu, J., Yang, J., Chen, B., Xu, J., Bao, H., et al. Identification of serum tsRNA-Thr-5-0015 and combined with AFP and PIVKA-II as novel biomarkers for hepatocellular carcinoma. Scientific reports 2024;14(1):28834. | Exclude on population: not cirrhosis |
| Jin, X. & Wang, H. Expression and Clinical Values of Serum miR-155 and miR-224 in Chinese Patients with HCV Infection. International Journal of General Medicine 2022;15():1393-4003. | Exclude on population: not cirrhosis |
| Jin, Young-Joo, Lee, Danbi, Chung, Young-Hwa, Kim, Jeong A., Kim, Sung Eun, Lee, Yoon-Seon, et al. Tumor Necrosis Factor-Alpha Gene Polymorphism Associated With Development of Hepatitis B Virus-associated Hepatocellular Carcinoma. Journal of clinical gastroenterology 2015;49(8):e76-81. | Does not meet full eligibility criteria: includes people without cirrhosis |
| Jin, Yu, Wong, Ye Shen, Goh, Brian K. P., Chan, Chung Yip, Cheow, Peng Chung, Chow, Pierce K. H., et al. Circulating microRNAs as Potential Diagnostic and Prognostic Biomarkers in Hepatocellular Carcinoma. Scientific reports 2019;9(1):10464. | No 2x2 data |
| Jing, Wei, Luo, Ping, Zhu, Man, Ai, Qian, Chai, Hongyan. Prognostic and Diagnostic Significance of SDPR-Cavin-2 in Hepatocellular Carcinoma. Cellular physiology and biochemistry : international journal of experimental cellular physiology, biochemistry, and pharmacology 2016;39(3):950-60. | Exclude on index test |
| Jingting, Jiang, Changping, Wu, Ning, Xu, yibei, Zhu, Jun, Wu, Mei, Ji, et al. Clinical evaluation of serum alpha-fetoprotein-IgM immune complexes on the diagnosis of primary hepatocellular carcinoma. Journal of clinical laboratory analysis 2009;23(4):213-18. | Exclude on population: not cirrhosis |
| Jin-no, K., Tanimizu, M., Hyodo, I., Nishikawa, Y., Hosokawa, Y., Endo, H., et al. Circulating platelet-derived endothelial cell growth factor increases in hepatocellular carcinoma patients. Cancer 1998;82(7):1260-67. | Exclude on study design: not a 1- or 2-gate test accuracy study |
| Jirun, Peng, Zhang, Guoxin, Ha, Seon-Ah, Kim, Hyun Kee, Yoo, Jinah, Kim, Sanghee, et al. HCCR-1 for detecting small hepatocellular carcinoma latent in a cirrhotic liver: a prospective cohort study. Gut 2012;61(10):1514-15. | Exclude on population: healthy controls |
| Jirun, Peng, Zhang, Guoxin, Kim, Hyun Kee, Ha, Seon-Ah, Zhongtian, Jin, Shishi, Qiao, et al. Clinical utility of alpha fetoprotein and HCCR-1, alone or in combination, in patients with chronic hepatitis, liver cirrhosis and hepatocellular carcinoma. Disease markers 2011;30(6):307-15. | Does not meet full eligibility criteria: includes people without cirrhosis |

| Reference | Reason for exclusion |
| --- | --- |
| Jodo, S., Kobayashi, S., Nakajima, Y., Matsunaga, T., Nakayama, N., Ogura, N., et al. Elevated serum levels of soluble Fas/APO-1 (CD95) in patients with hepatocellular carcinoma. Clinical and experimental immunology 1998;112(2):166-71. | Exclude on index test |
| Johnson, Andrea M., Dudek, Jeanne M., Edwards, David K., Myers, Theran A., Joseph, Patrick, Laffin, Jennifer J. Analytical validation of a novel multi-target blood-based test to detect hepatocellular carcinoma. Expert review of molecular diagnostics 2021;21(11):1245-52. | Exclude on population: not cirrhosis |
| Johnson, P J & Portmann, B. Alpha-fetoprotein concentrations measured by radioimmunoassay in diagnosing and excluding hepatocellular carcinoma. British medical journal 1978;2(6138):661-3. | Does not meet full eligibility criteria: includes people without cirrhosis |
| Johnson, P. J. & Portmann, B. Alpha-fetoprotein concentrations measured by radioimmunoassay in diagnosing and excluding hepatocellular carcinoma. British Medical Journal 1978;2(6138):661-63. | Does not meet full eligibility criteria: includes people without cirrhosis |
| Johnson, P. J., Bhatti, E., Toyoda, H. Serologic Detection of Hepatocellular Carcinoma: Application of Machine Learning and Implications for Diagnostic Models. JCO Clinical Cancer Informatics 2024;8():e2300199. | Does not meet full eligibility criteria: includes people without cirrhosis |
| Johnson, P. J., Leung, N., Cheng, P., Welby, C., Leung, W. T., Lau, W. Y., Yu, S. 'Hepatoma-specific' alphafetoprotein may permit preclinical diagnosis of malignant change in patients with chronic liver disease. British journal of cancer 1997;75(2):236-40. | Exclude on population: not cirrhosis |
| Johnson, Philip J., Pirrie, Sarah J., Cox, Trevor F., Berhane, Sarah, Teng, Mabel, Palmer, Daniel, et al. The detection of hepatocellular carcinoma using a prospectively developed and validated model based on serological biomarkers. Cancer epidemiology, biomarkers & prevention : a publication of the American Association for Cancer Research, cosponsored by the American Society of Preventive Oncology 2014;23(1):144-53. | Does not meet full eligibility criteria: includes people without cirrhosis |
| Jun, Tomi, Hsu, Yao-Chun, Ogawa, Shintaro, Huang, Yen-Tsung, Yeh, Ming-Lun, Tseng, Cheng-Hao, et al. Mac-2 Binding Protein Glycosylation Isomer as a Hepatocellular Carcinoma Marker in Patients With Chronic Hepatitis B or C Infection. Hepatology communications 2019;3(4):493-503. | No 2x2 data |
| Juratli, M. A., Pollmann, N. S., Oppermann, E., Mohr, A., Roy, D., Schnitzbauer, A., et al. Extracellular vesicles as potential biomarkers for diagnosis and recurrence detection of hepatocellular carcinoma. Scientific reports 2024;14(1):5322. | Exclude on population: not cirrhosis |
| Juratli, M. A., Stoffers, P. C., Zokai, R., Oppermann, E., Bechstein, W. O. Circulating tumor cells dynamic as therapy surveillance of liver tumor surgery. European Surgical Research 2017;58(5-6):317. | CONFERENCE ABSTRACT |
| Kakihara, Daisuke, Nishie, Akihiro, Harada, Noboru, Shirabe, Ken, Tajima, Tsuyoshi, Asayama, Yoshiki, et al. Performance of gadoxetic acid-enhanced MRI for detecting hepatocellular carcinoma in recipients of living-related-liver-transplantation: comparison with dynamic multidetector row computed tomography and angiography-assisted computed tomography. Journal of magnetic resonance imaging : JMRI 2014;40(5):1112-20. | Exclude on population: clinical signs and symptoms of HCC |
| Kalinich, Mark, Bhan, Irun, Kwan, Tanya T., Miyamoto, David T., Javaid, Sarah, LiCausi, Joseph A., et al. An RNA-based signature enables high specificity detection of circulating tumor cells in hepatocellular carcinoma. Proceedings of the National Academy of Sciences of the United States of America 2017;114(5):1123-28. | Does not meet full eligibility criteria: includes people without cirrhosis |
| Kamei, M., Misawa, A., Arai, J., Kamakura, K. Erythrina cristagalli lectin-reactive alpha-fetoprotein-E2: a marker of hepatocellular carcinoma and other malignancies. The International journal of biological markers 1998;13(1):24-29. | Exclude on index test |
| Kamel, Mahmoud M., Saad, Mohamed F., Mahmoud, Amal A., Edries, Awatief A. Evaluation of serum PIVKA-II and MIF as diagnostic markers for HCV/HBV induced hepatocellular carcinoma. Microbial pathogenesis 2014;77():31-35. | Exclude on population: not cirrhosis |
| Kamel, Marwa M., Matboli, Marwa, Sallam, Maha, Montasser, Iman F., Saad, Amr S. Investigation of long noncoding RNAs expression profile as potential serum biomarkers in patients with hepatocellular carcinoma. Translational research : the journal of laboratory and clinical medicine 2016;168():134-45. | Does not meet full eligibility criteria: includes people without cirrhosis |

| Reference | Reason for exclusion |
|---|---|
| Kamel, R. R., Amr, K. S., Afify, M., Elhosary, Y. A., Hegazy, A. E., Fahim, H. H. Relation between microRNAs and apoptosis in hepatocellular carcinoma. Open Access Macedonian Journal of Medical Sciences 2016;4(1):31-37. | Exclude on population: not cirrhosis |
| Kan, Miki, Hiraoka, Atsushi, Uehara, Takahide, Hidaka, Satoshi, Ichiryu, Misa, Nakahara, Hiromasa, et al. Evaluation of contrast-enhanced ultrasonography using perfluorobutane (Sonazoid( R)) in patients with small hepatocellular carcinoma: comparison with dynamic computed tomography. Oncology letters 2010;1(3):485-88. | Exclude on population: clinical signs and symptoms of HCC |
| Kandil, D. H. Glypican-3: A novel diagnostic marker for hepatocellular carcinoma and more. Advances in Anatomic Pathology 2009;16(2):125-29. | Exclude on study design: not a 1- or 2-gate test accuracy study |
| Kanematsu, M., Hoshi, H., Murakami, T., Inaba, Y., Kim, T., Yamada, T., et al. Detection of hepatocellular carcinoma in patients with cirrhosis: MR imaging versus angiographically assisted helical CT. AJR. American journal of roentgenology 1997;169(6):1507-15. | Exclude on population: clinical signs and symptoms of HCC |
| Kanematsu, T., Takenaka, K., Matsumata, T., Furuta, T., Sugimachi, K. Small liver cancer. The Japanese journal of surgery 1983;13(1):32-36. | Exclude on population: clinical signs and symptoms of HCC |
| Kang, C., Xu, R., Xu, F. The value of dynamic combined detection of serum tumor biomarkers in diagnosing primary hepatic carcinoma. Cancer Research and Clinic 2014;26(8):531-38. | Foreign language |
| Kang, Xiaonan, Sun, Lu, Guo, Kun, Shu, Hong, Yao, Jun, Qin, Xue. Serum protein biomarkers screening in HCC patients with liver cirrhosis by ICAT-LC-MS/MS. Journal of cancer research and clinical oncology 2010;136(8):1151-59. | Does not meet full eligibility criteria: includes people without cirrhosis |
| Kang, Yun Hee, Ji, Na Young, Lee, Chung Il, Lee, Hee Gu, Kim, Jae Wha, Yeom, Young Il, et al. ESM-1 silencing decreased cell survival, migration, and invasion and modulated cell cycle progression in hepatocellular carcinoma. Amino acids 2011;40(3):1003-13. | Does not meet full eligibility criteria: includes people without cirrhosis |
| Kang, Zhongjing & Jin, Kai. The Value of MRI Combined with AFP, AFP-L3, GP73, and DCP in the Diagnosis of Early Primary Liver Cancer. Disease markers 2022;2022():8640999. | Retracted |
| Kanmura, Shuji, Uto, Hirofumi, Kusumoto, Kazunori, Ishida, Yoichi, Hasuike, Satoru, Nagata, Kenji, et al. Early diagnostic potential for hepatocellular carcinoma using the SELDI ProteinChip system. Hepatology (Baltimore, Md.) 2007;45(4):948-56. | Exclude on population: not cirrhosis |
| Kanmura, Shuji, Uto, Hirofumi, Kusumoto, Kazunori, Ishida, Yoichi, Hasuike, Satoru, Nagata, Kenji, et al. Early diagnostic potential for hepatocellular carcinoma using the SELDI ProteinChip system. Hepatology (Baltimore, Md.) 2007;45(4):948-56. | Exclude on population: not cirrhosis |
| Kanmura, Shuji, Uto, Hirofumi, Sato, Yuko, Kumagai, Koutarou, Sasaki, Fumisato, Moriuchi, Akihiro, et al. The complement component C3a fragment is a potential biomarker for hepatitis C virus-related hepatocellular carcinoma. Journal of gastroenterology 2010;45(4):459-67. | Exclude on population: not cirrhosis |
| Kardum, D., Huskic, J., Fabijanic, D., Banic, M., Buljevac, M., Kujundzic, M. Activity of serum angiotensin-converting enzyme as a tumour marker of hepatocellular carcinoma. European journal of gastroenterology & hepatology 1999;11(11):1209-13. | Exclude on population: healthy controls |
| Kasahara, A., Hayashi, N., Fusamoto, H., Kawada, Y., Imai, Y., Yamamoto, H., et al. Clinical evaluation of plasma des-gamma-carboxylic prothrombin as a marker protein of hepatocellular carcinoma in patients with tumors of various sizes. Digestive Diseases and Sciences 1993;38(12):2170-76. | Does not meet full eligibility criteria: includes people without cirrhosis |
| Kasai, Yoshitaka, Moriyasu, Fuminori, Saito, Kazuhiro, Hara, Takeshi, Kobayashi, Yoshiyuki, Nakamura, Ikuo. Value of shear wave elastography for predicting hepatocellular carcinoma and esophagogastric varices in patients with chronic liver disease. Journal of medical ultrasonics (2001) 2015;42(3):349-55. | Exclude on population: not cirrhosis |
| Kawata, H. & Shimizu, Y. Retrospective study on the evaluation of diagnostic procedure for hepatoma in patients with cirrhosis of the liver. Acta Hepato-Gastroenterologica 1974;24(2):106-19. | Does not meet full eligibility criteria: pre-2005 |

| Reference | Reason for exclusion |
|---|---|
| Kawata, N., Takahashi, H., Iwane, S., Inoue, K., Kojima, M., Kohno, M., et al. FIB-4 index-based surveillance for advanced liver fibrosis in diabetes patients. Diabetology International 2021;12(1):118-25. | Exclude on index test |
| Kawata, Shuji, Murakami, Takamichi, Kim, Tonsok, Hori, Masatoshi, Federle, Michael P., Kumano, Seishi, et al. Multidetector CT: diagnostic impact of slice thickness on detection of hypervascular hepatocellular carcinoma. AJR. American journal of roentgenology 2002;179(1):61-66. | Exclude on population: clinical signs and symptoms of HCC |
| Ke, W. M. & Satoru, I. Clinical values of AFP in the diagnosis of hepatocellular carcinoma. Chinese Journal of Clinical Oncology 1995;22(10):694-96. | Does not meet full eligibility criteria: pre-2005 |
| Kedda, M. A., Kew, M. C., Skelton, M. Non-specificity of messenger RNA of alpha-fetoprotein in peripheral blood in detecting early spread of hepatocellular carcinoma in black Africans. Journal of gastroenterology and hepatology 1998;13(9):885-91. | Exclude on population: not cirrhosis |
| Kew, M. C. The value of serum concentrations of tissue polypeptide antigen in the diagnosis of hepatocellular carcinoma. Cancer 1986;58(1):127-30. | Exclude on population: not cirrhosis |
| Khairy, Ahmed, Hamza, Iman, Shaker, Olfat. Serum miRNA Panel in Egyptian Patients with Chronic Hepatitis C Related Hepatocellular Carcinoma. Asian Pacific journal of cancer prevention : APJCP 2016;17(5):2699-7003. | Exclude on population: not cirrhosis |
| Khalifa, A., Abdeen, N. S., Lewis, N., ElMalah, S. G. CHRIST: CD44-INCORPORATED HEPATOCELLULAR CARCINOMA RISK INDEX SCORING TOOL - A NOVEL PROGNOSTIC SCORING SYSTEM FOR HEPATOCELLULAR CARCINOMA AGGRESSIVENESS. Gastroenterology 2022;162(7 Supplement):S-1161. | CONFERENCE ABSTRACT |
| Khalifa, A., Mady, E. A., Abadeer, N. Differential tumor markers and hepatitis markers profile in liver tumors. Anticancer research 1999;19(4A):2495-5000. | Does not meet full eligibility criteria: pre-2005 |
| Khalil, Ashraf, Elgedawy, Jamalat, Faramawi, Mohammed F., Elfert, Ashraf, Salama, Ibrahim, Abbass, Ahmed, Elsaid, Hala. Plasma osteopontin level as a diagnostic marker of hepatocellular carcinoma in patients with radiological evidence of focal hepatic lesions. Tumori 2013;99(1):100-07. | Exclude on target condition: not HCC |
| Khan, Asra S., Hussain, Hero K., Johnson, Timothy D., Weadock, William J., Pelletier, Shawn J. Value of delayed hypointensity and delayed enhancing rim in magnetic resonance imaging diagnosis of small hepatocellular carcinoma in the cirrhotic liver. Journal of magnetic resonance imaging : JMRI 2010;32(2):360-66. | Exclude on population: clinical signs and symptoms of HCC |
| Khan, Ilvira M., Gjuka, Donjeta, Jiao, Jingjing, Song, Xiaoling, Wang, Ying, Wang, Jing, et al. A Novel Biomarker Panel for the Early Detection and Risk Assessment of Hepatocellular Carcinoma in Patients with Cirrhosis. Cancer prevention research (Philadelphia, Pa.) 2021;14(6):667-74. | Exclude on study design: not a 1- or 2-gate test accuracy study |
| Khien, V. V., Mao, H. V., Chinh, T. T., Ha, P. T., Bang, M. H., Lac, B. V., et al. Clinical evaluation of lentil lectin-reactive alpha-fetoprotein-L3 in histology-proven hepatocellular carcinoma. The International journal of biological markers 2001;16(2):105-11. | Does not meet full eligibility criteria: includes people without cirrhosis |
| Kienle, P., Weitz, J., Klaes, R., Koch, M., Benner, A., Lehnert, T., Herfarth, C. Detection of isolated disseminated tumor cells in bone marrow and blood samples of patients with hepatocellular carcinoma. Archives of surgery (Chicago, Ill. : 1960) 2000;135(2):213-18. | Exclude on population: not cirrhosis |
| Kim M.-R., Gu G.-G., Kim S.Y. AFP and PIVKAII as surveillance biomarkers for hepatocellular Carcinoma. American Journal of Clinical Pathology 2012;138(SUPPL. 2):A190. | Exclude on population: not cirrhosis |
| Kim, A. K., Lin, S. Y., Jain, S., Cui, Y., Gade, T., Shieh, F. S., et al. Urine as a non-invasive alternative to blood for germline and somatic mutation detection in hepatocellular carcinoma. medRxiv 2021;():n. pag.. | Exclude on population: not cirrhosis |
| Kim, Ah Yeong, Kim, Young Kon, Lee, Min Woo, Park, Min Jung, Hwang, Jiyoung, Lee, Mi Hee. Detection of hepatocellular carcinoma in gadoxetic acid-enhanced MRI and diffusion-weighted MRI with respect to the severity of liver cirrhosis. Acta radiologica (Stockholm, Sweden : 1987) 2012;53(8):830-38. | Exclude on population: clinical signs and symptoms of HCC |
| Kim, Amy K., Hamilton, James P., Lin, Selena Y., Chang, Ting-Tsung, Hann, Hie-Won, Hu, Chi-Tan, et al. Urine DNA biomarkers for hepatocellular carcinoma screening. British journal of cancer 2022;126(10):1432-38. | Does not meet full eligibility criteria: includes people without cirrhosis |

| Reference | Reason for exclusion |
|---|---|
| Kim, C K & Lim, J H. Detection of hepatocellular carcinomas and dysplastic nodules in cirrhotic liver: accuracy of ultrasonography in transplant patients. Journal of ultrasound in medicine : official journal of the American Institute of Ultrasound in Medicine 2001;20(2):99-104. | Does not meet full eligibility criteria: pre-2005 |
| Kim, D. W., Choi, S. H., Kim, S. Y., Byun, J. H., Lee, S. S., Park, S. H. Diagnostic performance of MRI for HCC according to contrast agent type: a systematic review and meta-analysis. Hepatology International 2020;14(6):1009-22. | Exclude on study design: not a 1- or 2-gate test accuracy study |
| Kim, Da Jung, Cho, Eun Ju, Yu, Kyung-Sang, Jang, In-Jin, Yoon, Jung-Hwan, Park, Taesung. Comprehensive Metabolomic Search for Biomarkers to Differentiate Early Stage Hepatocellular Carcinoma from Cirrhosis. Cancers 2019;11(10):n. pag.. | Duplicate (including Cochrane includes) |
| Kim, Da Jung, Cho, Eun Ju, Yu, Kyung-Sang, Jang, In-Jin, Yoon, Jung-Hwan, Park, Taesung. Comprehensive Metabolomic Search for Biomarkers to Differentiate Early Stage Hepatocellular Carcinoma from Cirrhosis. Cancers 2019;11(10):n. pag.. | Does not meet full eligibility criteria: includes people without cirrhosis |
| Kim, Dong Hwan, Yoon, Jeong Hee, Choi, Moon Hyung, Lee, Chang Hee, Kang, Tae Wook, Kim, Hyun A., et al. Comparison of non-contrast abbreviated MRI and ultrasound as surveillance modalities for HCC. Journal of hepatology 2024;81(3):461â€“470. | No 2x2 data |
| Kim, Hyunsoo, Kim, Kyunggon, Jin, Jonghwa, Park, Jiyoung, Yu, Su Jong, Yoon, Jung-Hwan. Measurement of glycosylated alpha-fetoprotein improves diagnostic power over the native form in hepatocellular carcinoma. PloS one 2014;9(10):e110366. | Does not meet full eligibility criteria: includes people without cirrhosis |
| Kim, Hyunsoo, Kim, Kyunggon, Jin, Jonghwa, Park, Jiyoung, Yu, Su Jong, Yoon, Jung-Hwan. Measurement of glycosylated alpha-fetoprotein improves diagnostic power over the native form in hepatocellular carcinoma. PloS one 2014;9(10):e110366. | Duplicate (including Cochrane includes) |
| Kim, Hyunsoo, Park, JiYoung, Kim, Yongkang, Sohn, Areum, Yeo, Injun, Jong Yu, Su, et al. Serum fibronectin distinguishes the early stages of hepatocellular carcinoma. Scientific reports 2017;7(1):9449. | Exclude on target condition: not HCC |
| Kim, J. W., Ye, Q., Forgues, M., Chen, Y., Budhu, A., Sime, J., et al. Cancer-Associated Molecular Signature in the Tissue Samples of Patients with Cirrhosis. Hepatology 2004;39(2):518-27. | Exclude on population: tissue samples |
| Kim, Ji Hyun, Kang, Seong Hee, Lee, Minjong, Choi, Hoon Sung, Jun, Baek Gyu, Kim, Tae Suk, et al. Improved detection of hepatocellular carcinoma by dynamic computed tomography in cirrhotic patients with chronic hepatitis B: A multicenter study. Journal of gastroenterology and hepatology 2020;35(10):1795-8003. | Exclude on study design: not a 1- or 2-gate test accuracy study |
| Kim, Jin Sil, Lee, Jeong Kyong, Baek, Seung Yon. Diagnostic performance of a minimized protocol of non-contrast MRI for hepatocellular carcinoma surveillance. Abdominal radiology (New York) 2020;45(1):211-19. | Exclude on population: not cirrhosis |
| Kim, Jin, Ki, Seung S, Lee, Sang D, Han, Chul J, Kim, Yu C, Park, Sun H, et al. Elevated plasma osteopontin levels in patients with hepatocellular carcinoma. The American journal of gastroenterology 2006;101(9):2051-9. | Does not meet full eligibility criteria: includes people without cirrhosis |
| Kim, Jin, Ki, Seung S., Lee, Sang D., Han, Chul J., Kim, Yu C., Park, Sun H., et al. Elevated plasma osteopontin levels in patients with hepatocellular carcinoma. The American journal of gastroenterology 2006;101(9):2051-59. | Exclude on population: not cirrhosis |
| Kim, Junmo, Min, Ji Hye, Kim, Seon Kyoung, Shin, Soo-Yong. Detection of Hepatocellular Carcinoma in Contrast-Enhanced Magnetic Resonance Imaging Using Deep Learning Classifier: A Multi-Center Retrospective Study. Scientific reports 2020;10(1):9458. | Exclude on study design: not a 1- or 2-gate test accuracy study |
| Kim, K. H., Lee, S. Y., Hwang, H., Lee, J. Y., Ji, E. S., An, H. J., Kim, J. Y. Direct Monitoring of Fucosylated Glycopeptides of Alpha-Fetoprotein in Human Serum for Early Hepatocellular Carcinoma by Liquid Chromatography-Tandem Mass Spectrometry with Immunoprecipitation. Proteomics - Clinical Applications 2018;12(6):1800062. | Duplicate (including Cochrane includes) |
| Kim, Kwang Hoe, Lee, Sang Yoon, Baek, Je-Hyun, Lee, Soo-Youn, Kim, Jin Young. Measuring fucosylated alpha-fetoprotein in hepatocellular carcinoma: A | Exclude on index test |

| Reference | Reason for exclusion |
| --- | --- |
| comparison of muTAS and parallel reaction monitoring. Proteomics. Clinical applications 2021;15(4):e2000096. | |
| Kim, Kwang Hoe, Lee, Soo-Youn, Hwang, Heeyoun, Lee, Ju Yeon, Ji, Eun Sun, An, Hyun Joo, Kim, Jin Young. Direct Monitoring of Fucosylated Glycopeptides of Alpha-Fetoprotein in Human Serum for Early Hepatocellular Carcinoma by Liquid Chromatography-Tandem Mass Spectrometry with Immunoprecipitation. Proteomics. Clinical applications 2018;12(6):e1800062. | Duplicate (including Cochrane includes) |
| Kim, Kwang Hoe, Lee, Soo-Youn, Hwang, Heeyoun, Lee, Ju Yeon, Ji, Eun Sun, An, Hyun Joo, Kim, Jin Young. Direct Monitoring of Fucosylated Glycopeptides of Alpha-Fetoprotein in Human Serum for Early Hepatocellular Carcinoma by Liquid Chromatography-Tandem Mass Spectrometry with Immunoprecipitation. Proteomics. Clinical applications 2018;12(6):e1800062. | Does not meet full eligibility criteria: includes people without cirrhosis |
| Kim, Kwang Hoe, Park, Gun Wook, Jeong, Ji Eun, Ji, Eun Sun, An, Hyun Joo, Kim, Jin Young. Parallel reaction monitoring with multiplex immunoprecipitation of N-glycoproteins in human serum for detection of hepatocellular carcinoma. Analytical and bioanalytical chemistry 2019;411(14):3009-19. | No 2x2 data |
| Kim, Kyeezu, Zheng, Yinan, Joyce, Brian T., Nannini, Drew R., Wang, Jun, Qu, Yishu, et al. Cell-free DNA methylation-based inflammation score as a marker for hepatocellular carcinoma among people living with HIV. Hepatology international 2025;19(3):596â€“606. | Does not meet full eligibility criteria: includes people without cirrhosis |
| Kim, Kyung Won, Lee, Jeong Min, Klotz, Ernst, Park, Hee Sun, Lee, Dong Ho, Kim, Ji Young, et al. Quantitative CT color mapping of the arterial enhancement fraction of the liver to detect hepatocellular carcinoma. Radiology 2009;250(2):425-34. | No 2x2 data |
| Kim, Kyung-Ah, Lee, June Sung, Jung, Eun Sook, Kim, Jong Yeon, Bae, Won Ki, Kim, Nam-Hoon. [Usefulness of serum alpha-fetoprotein (AFP) as a marker for hepatocellular carcinoma (HCC) in hepatitis C virus related cirrhosis: analysis of the factors influencing AFP elevation without HCC development]. The Korean journal of gastroenterology = Taehan Sohwagi Hakhoe chi 2006;48(5):321-6. | Foreign language |
| Kim, Kyung-Ah, Lee, June Sung, Jung, Eun Sook, Kim, Jong Yeon, Bae, Won Ki, Kim, Nam-Hoon. [Usefulness of serum alpha-fetoprotein (AFP) as a marker for hepatocellular carcinoma (HCC) in hepatitis C virus related cirrhosis: analysis of the factors influencing AFP elevation without HCC development]. The Korean journal of gastroenterology = Taehan Sohwagi Hakhoe chi 2006;48(5):321-26. | Secondary publication of included study |
| Kim, M. N., Kim, B. K., Kim, S. U., Park, J. Y., Ahn, S. H., Han, K. H. Longitudinal assessment of alpha-fetoprotein for early detection of hepatocellular carcinoma in patients with cirrhosis. Scandinavian Journal of Gastroenterology 2019;54(10):1283-90. | Duplicate (including Cochrane includes) |
| Kim, Myong Jin, Bae, Kang Woo, Seo, Pyoung Ju, Jeong, In Kook, Kim, Jung Hyuk, Lee, Bo Han, et al. [Optimal cut-off value of PIVKA-II for diagnosis of hepatocellular carcinoma--using ROC curve]. The Korean journal of hepatology 2006;12(3):404-11. | Foreign language |
| Kim, S. C., Kim, D. W., Cho, E. J., Lee, J. Y., Kim, J., Kwon, C., et al. A circulating cell-free DNA methylation signature for the detection of hepatocellular carcinoma. Molecular Cancer 2023;22(1):164. | Exclude on population: not cirrhosis |
| Kim, S. U., Park, J. H., Kim, H. S., Lee, J. M., Lee, H. G., Kim, H., et al. Serum dickkopf-1 as a biomarker for the diagnosis of hepatocellular carcinoma. Yonsei Medical Journal 2015;56(5):1296-3006. | Exclude on population: HCC participants not treatment-naive |
| Kim, Se Hyung, Lee, Jeong Min, Kim, Young Jun, Choi, Jin Young, Kim, Gi Hyeon, Lee, Ho Yun. Detection of hepatocellular carcinoma on CT in liver transplant candidates: comparison of PACS tile and multisynchronized stack modes. AJR. American journal of roentgenology 2007;188(5):1337-42. | Exclude on population: clinical signs and symptoms of HCC |
| Kim, Seung Kwon, Lim, Jae Hoon, Lee, Won Jae, Kim, Seung Hoon, Choi, Dongil, Lee, Soon Jin, Lim, Hyo K. Detection of hepatocellular carcinoma: comparison of dynamic three-phase computed tomography images and four-phase computed tomography images using multidetector row helical computed tomography. Journal of computer assisted tomography 2002;26(5):691-98. | Exclude on population: clinical signs and symptoms of HCC |

| Reference | Reason for exclusion |
|---|---|
| Kim, Si-Cho, Kim, Da-Won, Cho, Eun Ju, Lee, Jin-Young, Kim, Jiwon, Kwon, Chaesun, et al. A circulating cell-free DNA methylation signature for the detection of hepatocellular carcinoma. Molecular cancer 2023;22(1):164. | Exclude on population: not cirrhosis |
| Kim, Soon Sun, Baek, Geum Ok, Ahn, Hye Ri, Sung, Suna, Seo, Chul Won, Cho, Hyo Jung, et al. Serum small extracellular vesicle-derived LINC00853 as a novel diagnostic marker for early hepatocellular carcinoma. Molecular oncology 2020;14(10):2646-59. | Does not meet full eligibility criteria: includes people without cirrhosis |
| Kim, Soon Sun, Baek, Geum Ok, Son, Ju A., Ahn, Hye Ri, Yoon, Moon Kyung, Cho, Hyo Jung, et al. Early detection of hepatocellular carcinoma via liquid biopsy: panel of small extracellular vesicle-derived long noncoding RNAs identified as markers. Molecular oncology 2021;15(10):2715-31. | Does not meet full eligibility criteria: includes people without cirrhosis |
| Kim, Sun Jo, Jung, Cheol Woon, Anh, Nguyen Hoang, Yoon, Young Cheol, Long, Nguyen Phuoc, Hong, Soon-Sun, Cho, Eun Ju. Metabolic phenotyping combined with transcriptomics metadata fortifies the diagnosis of early-stage Hepatocellular carcinoma. Journal of advanced research 2025;74():153â€“163. | No 2x2 data |
| Kim, Y. Y., An, C., Kim, S. Diagnostic accuracy of prospective application of the Liver Imaging Reporting and Data System (LI-RADS) in gadoxetate-enhanced MRI. European Radiology 2018;28(5):2038-46. | Exclude on population: clinical signs and symptoms of HCC |
| Kim, Yi Kyung, Kim, Young Kon, Park, Hyun Jeong, Park, Min Jung, Lee, Won Jae. Noncontrast MRI with diffusion-weighted imaging as the sole imaging modality for detecting liver malignancy in patients with high risk for hepatocellular carcinoma. Magnetic resonance imaging 2014;32(6):610-18. | Exclude on target condition: not HCC |
| Kim, Young Kon, Kim, Chong Soo, Lee, Young Hwan, Kwak, Hyo Sung. Comparison of superparamagnetic iron oxide-enhanced and gadobenate dimeglumine-enhanced dynamic MRI for detection of small hepatocellular carcinomas. AJR. American journal of roentgenology 2004;182(5):1217-23. | Exclude on population: clinical signs and symptoms of HCC |
| Kim, Young Kon, Kwak, Hyo Sung, Han, Young Min. Usefulness of combining sequentially acquired gadobenate dimeglumine-enhanced magnetic resonance imaging and resovist-enhanced magnetic resonance imaging for the detection of hepatocellular carcinoma: comparison with computed tomography hepatic arteriography and computed tomography arterioportography using 16-slice multidetector computed tomography. Journal of computer assisted tomography 2007;31(5):702-11. | Exclude on population: clinical signs and symptoms of HCC |
| Kim, Younghwan, Park, Yo-Han, Hwang, Shin, Kim, Ki-Hun, Ahn, Chul-Soo, Moon, Deok-Bog, et al. Diagnostic Role of Blood Tumor Markers in Predicting Hepatocellular Carcinoma in Liver Cirrhosis Patients Undergoing Liver Transplantation. Annals of transplantation 2016;21():660-67. | Duplicate (including Cochrane includes) |
| Kinami, Y., Yokota, H., Takata, M., Takashima, S. Magnetic resonance imaging in the diagnosis of tumors of the liver. Gastroenterologia Japonica 1988;23(2):139-46. | No 2x2 data |
| King, Michael J., Lee, Karen M., Rosberger, Sonam, Huang, Hsin-Hui, Meza, Gabriela Hernandez, Lewis, Sara. HCC screening with ultrasound: assessment of quality using ultrasound LI-RADS score. Abdominal radiology (New York) 2023;48(1):263-70. | Does not meet full eligibility criteria: includes people without cirrhosis |
| Kirk, G. D., Camus-Randon, A. M., Mendy, M., Goedert, J. J., Merle, P., Trepo, C., et al. Ser-249 p53 mutations in plasma DNA of patients with hepatocellular carcinoma from The Gambia. Journal of the National Cancer Institute 2000;92(2):148-53. | Does not meet full eligibility criteria: includes people without cirrhosis |
| Kisiel, John B., Dukek, Brian A., V S R Kanipakam, Reddappa, Ghoz, Hassan M., Yab, Tracy C., Berger, Calise K., et al. Hepatocellular Carcinoma Detection by Plasma Methylated DNA: Discovery, Phase I Pilot, and Phase II Clinical Validation. Hepatology (Baltimore, Md.) 2019;69(3):1180-92. | Does not meet full eligibility criteria: includes people without cirrhosis |
| Kitamura, Takatoshi, Ichikawa, Tomoaki, Erturk, Sukru Mehmet, Nakajima, Hiroto, Sou, Hironobu, Araki, Tsutomu, Okada, Shunichi. Detection of hypervascular hepatocellular carcinoma with multidetector-row CT: single arterial-phase imaging with computer-assisted automatic bolus-tracking technique compared with double arterial-phase imaging. Journal of computer assisted tomography 2008;32(5):724-29. | Exclude on population: clinical signs and symptoms of HCC |
| Kiyokawa, Hirofumi, Yasuda, Hiroshi, Oikawa, Ritsuko, Okuse, Chiaki, Matsumoto, Nobuyuki, Ikeda, Hiroki, et al. Serum monomeric laminin-gamma2 | Exclude on population: not cirrhosis |

| Reference | Reason for exclusion |
|---|---|
| as a novel biomarker for hepatocellular carcinoma. Cancer science 2017;108(7):1432-39. | |
| Kloeckner, Roman, Pinto Dos Santos, Daniel, Kreitner, Karl-Friedrich, Leicher-Duber, Anne, Weinmann, Arndt, Mittler, Jens. Quantitative assessment of washout in hepatocellular carcinoma using MRI. BMC cancer 2016;16(1):758. | Exclude on population: clinical signs and symptoms of HCC |
| Ko, Young Sun, Bae, Joo Hwan, Sinn, Dong Hyun, Gwak, Geum Youn, Kang, Wonseok, Paik, Yong Han, et al. [The Clinical Significance of Serum Alpha-fetoprotein in Diagnosing Hepatocellular Carcinoma in a Health Screening Population]. The Korean journal of gastroenterology = Taehan Sohwagi Hakhoe chi 2017;69(4):232-38. | Foreign language |
| Kobashi, Haruhiko, Miyake, Yasuhiro, Ikeda, Fusao, Yasunaka, Tetsuya, Nishino, Ken, Moriya, Akio, et al. Long-term outcome and hepatocellular carcinoma development in chronic hepatitis B or cirrhosis patients after nucleoside analog treatment with entecavir or lamivudine. Hepatology research : the official journal of the Japan Society of Hepatology 2011;41(5):405-16. | Exclude on population: not cirrhosis |
| Kobayashi, K., Sugimoto, T., Makino, H., Kumagai, M., Unoura, M., Tanaka, N., Kato, Y. Screening methods for early detection of hepatocellular carcinoma. Hepatology (Baltimore, Md.) 1985;5(6):1100-05. | Does not meet full eligibility criteria: pre-2005 |
| Kobayashi, Toshihiko, Ichihara, Kiyoshi, Goda, Shuhei, Hidaka, Isao, Yamasaki, Takahiro. Exploration and time-serial validation of logistic regression models composed of multiple laboratory tests for early detection of HCV-associated hepatocellular carcinoma. Clinica chimica acta; international journal of clinical chemistry 2021;521():137-43. | Exclude on population: not cirrhosis |
| Koberle, V., Kronenberger, B., Pleli, T., Trojan, J., Imelmann, E., Peveling-Oberhag, J., et al. Serum microRNA-1 and microRNA-122 are prognostic markers in patients with hepatocellular carcinoma. European Journal of Cancer 2013;49(16):3442-49. | No 2x2 data |
| Kobi, M., Paroder, V., Flusberg, M., Rozenblit, A. M. Limitations of GD-EOB-DTPA-enhanced MRI: can clinical parameters predict suboptimal hepatobiliary phase? Clinical radiology 2017;72(1):55-62. | Exclude on index test |
| Koda, T., Yamazaki, S., Tamura, I., Nakaba, H., Takao, T., Katayama, S. Abnormal prothrombin: evaluation as a tumour marker and localization in tissues of patients with hepatocellular carcinoma. Journal of gastroenterology and hepatology 1993;8(3):212-16. | Does not meet full eligibility criteria: pre-2005 |
| Kohansal-Nodehi, M., Swiatek-de Lange, M., Kroeniger, K., Rolny, V., Tabares, G., Piratvisuth, T., et al. Discovery of a haptoglobin glycopeptides biomarker panel for early diagnosis of hepatocellular carcinoma. Frontiers in Oncology 2023;13():1213898. | No 2x2 data |
| Koksal, A. R., Ekmen, N., Aydin, Y., Nunez, K., Sandow, T., Delk, M., et al. A Single-Step Immunocapture Assay to Quantify HCC Exosomes Using the Highly Sensitive Fluorescence Nanoparticle-Tracking Analysis. Journal of Hepatocellular Carcinoma 2023;10():1935 “ 1954. | Does not meet full eligibility criteria: includes people without cirrhosis |
| Kong, Deok-Hoon, Jung, Jae-Wan, Na, Keun, Jeong, Seul-Ki, Paik, Young-Ki, Jung, Se-Hui, et al. Normalization using a tagged-internal standard assay for analysis of antibody arrays and the evaluation of serological biomarkers for liver disease. Analytica chimica acta 2012;718():92-98. | Exclude on population: healthy controls |
| Kong, Yunpeng, Jing, Yan, Sun, Hongmei. The Diagnostic Value of Contrast-Enhanced Ultrasound and Enhanced CT Combined with Tumor Markers AFP and CA199 in Liver Cancer. Journal of healthcare engineering 2022;2022():5074571. | Exclude on population: clinical signs and symptoms of HCC |
| Konishi, Hirotaka, Ichikawa, Daisuke, Yamamoto, Yusuke, Arita, Tomohiro, Shoda, Katsutoshi, Hiramoto, Hidekazu, et al. Plasma level of metastasis-associated lung adenocarcinoma transcript 1 is associated with liver damage and predicts development of hepatocellular carcinoma. Cancer science 2016;107(2):149-54. | Exclude on population: not cirrhosis |
| Koshikawa, N., Shimakami, T., Terashima, T., Nakagawa, M., Nio, K., Horii, R., et al. Serum Laminin gamma2 Monomer as a Diagnostic and Predictive Biomarker for Hepatocellular Carcinoma. Hepatology 2021;74(2):760-75. | Does not meet full eligibility criteria: includes people without cirrhosis |

| Reference | Reason for exclusion |
|---|---|
| Kotoh, Yurika, Suehiro, Yutaka, Saeki, Issei, Hoshida, Tomomi, Maeda, Masaki, Iwamoto, Takuya, et al. Novel Liquid Biopsy Test Based on a Sensitive Methylated SEPT9 Assay for Diagnosing Hepatocellular Carcinoma. Hepatology communications 2020;4(3):461-70. | Does not meet full eligibility criteria: includes people without cirrhosis |
| Koyama, Noriyuki, Yamazaki, Tomoko, Kanetsuki, Yuka, Hirota, Jiro, Asai, Tomohide, Mitsumoto, Yasuhide, et al. Activation of apoptosis inhibitor of macrophage is a sensitive diagnostic marker for NASH-associated hepatocellular carcinoma. Journal of gastroenterology 2018;53(6):770-79. | No 2x2 data |
| Koziol, James A., Imai, Haruhiko, Dai, Liping, Zhang, Jian-Ying. Early detection of hepatocellular carcinoma using autoantibody profiles from a panel of tumor-associated antigens. Cancer immunology, immunotherapy : CII 2018;67(5):835-41. | Exclude on population: healthy controls |
| Krinsky, G. A., Lee, V. S., Theise, N. D., Weinreb, J. C., Rofsky, N. M., Diflo, T. Hepatocellular carcinoma and dysplastic nodules in patients with cirrhosis: prospective diagnosis with MR imaging and explantation correlation. Radiology 2001;219(2):445-54. | Does not meet full eligibility criteria: pre-2005 |
| Ksiazek, W., Abdar, M., Acharya, U. R. A novel machine learning approach for early detection of hepatocellular carcinoma patients. Cognitive Systems Research 2019;54():116-27. | No 2x2 data |
| Kuaaroon, W., Tiyarattanachai, T., Apiparakoon, T., Marukatat, S., Tanpowpong, N., Treeprasertsuk, S., et al. Machine learning models for predicting hepatocellular carcinoma development in patients with chronic viral hepatitis B infection. Asian Biomedicine 2025;19(1):51 " 59. | Exclude on population: not cirrhosis |
| Kubo, Y., Okuda, K., Musha, H. Detection of hepatocellular carcinoma during a clinical follow-up of chronic liver disease. Observations in 31 patients. Gastroenterology 1978;74(3):578-82. | Exclude on study design: not a 1- or 2-gate test accuracy study |
| Kudo, Masatoshi, Zheng, Rong Qin, Kim, Soo Ryang, Okabe, Yoshihiro, Osaki, Yukio, Iijima, Hiroko, et al. Diagnostic accuracy of imaging for liver cirrhosis compared to histologically proven liver cirrhosis. A multicenter collaborative study. Intervirology 2008;51 Suppl 1():17-26. | Exclude on target condition: not HCC |
| Kumada, Takashi, Toyoda, Hidenori, Kiriyama, Seiki, Tanikawa, Makoto, Hisanaga, Yasuhiro, Kanamori, Akira, et al. Predictive value of tumor markers for hepatocarcinogenesis in patients with hepatitis C virus. Journal of gastroenterology 2011;46(4):536-44. | Exclude on population: not cirrhosis |
| Kumada, Takashi, Toyoda, Hidenori, Tada, Toshifumi, Kiriyama, Seiki, Tanikawa, Makoto, Hisanaga, Yasuhiro, et al. High-sensitivity Lens culinaris agglutinin-reactive alpha-fetoprotein assay predicts early detection of hepatocellular carcinoma. Journal of gastroenterology 2014;49(3):555-63. | Exclude on population: not cirrhosis |
| Kumada, Takashi, Toyoda, Hidenori, Tada, Toshifumi, Kiriyama, Seiki, Tanikawa, Makoto, Hisanaga, Yasuhiro, et al. High-sensitivity Lens culinaris agglutinin-reactive alpha-fetoprotein assay predicts early detection of hepatocellular carcinoma. Journal of gastroenterology 2014;49(3):555-63. | Duplicate (including Cochrane includes) |
| Kumagai, Y., Chiba, J., Sata, T., Ohtaki, S. A new tumor-associated antigen useful for serodiagnosis of hepatocellular carcinoma, defined by monoclonal antibody KM-2. Cancer Research 1992;52(18):4987-94. | No 2x2 data |
| Kumar, D. & Sharma, M. K. Comparative Study of CT and MRI in the Early Detection and Staging of Hepatocellular Carcinoma: A Prospective Diagnostic Accuracy Cohort Study. European Journal of Cardiovascular Medicine 2025;15(4):766 " 773. | Does not meet full eligibility criteria: includes people without cirrhosis |
| Kunadirek, Pattapon, Ariyachet, Chaiyaboot, Sriphoosanaphan, Supachaya, Pinjaroen, Nutcha, Sirichindakul, Pongserath, Nookaew, Intawat, Chuaypen, Natthaya. Identification of BHLHE40 expression in peripheral blood mononuclear cells as a novel biomarker for diagnosis and prognosis of hepatocellular carcinoma. Scientific reports 2021;11(1):11201. | Exclude on population: not cirrhosis |
| Kunadirek, Pattapon, Chuaypen, Natthaya, Jenjaroenpun, Piroon, Wongsurawat, Thidathip, Pinjaroen, Nutcha, Sirichindakul, Pongserath, Nookaew, Intawat. Cell-Free DNA Analysis by Whole-Exome Sequencing for Hepatocellular Carcinoma: A Pilot Study in Thailand. Cancers 2021;13(9):n. pag.. | Exclude on population: not cirrhosis |

| Reference | Reason for exclusion |
|---|---|
| Kunimune, Y., Suehiro, Y., Saeki, I., Yamauchi, Y., Tanabe, N., Matsumoto, T., et al. Combination Assay of Methylated HOXA1 with Tumor Markers Shows High Sensitivity for Detection of Early-Stage Hepatocellular Carcinoma. Liver Cancer 2024;13(5):487 “ 497. | Does not meet full eligibility criteria: includes people without cirrhosis |
| Kuo, Chih-Chi, Lin, Ching-Yu, Shih, Yu-Lueng, Hsieh, Chung-Bao, Lin, Pei-Yu, Guan, Shuh-Bing, et al. Frequent methylation of HOXA9 gene in tumor tissues and plasma samples from human hepatocellular carcinomas. Clinical chemistry and laboratory medicine 2014;52(8):1235-45. | Exclude on population: not cirrhosis |
| Kuo, Yuan-Hung, Lu, Sheng-Nan, Hung, Chao-Hung, Kee, Kwong-Ming, Chen, Chien-Hung, Hu, Tsung-Hui, et al. Liver stiffness measurement in the risk assessment of hepatocellular carcinoma for patients with chronic hepatitis. Hepatology international 2010;4(4):700-06. | Exclude on population: not cirrhosis |
| Kuromatsu, R. & Tanaka, M. Serum alpha-fetoprotein and lens culinaris agglutinin-reactive fraction of alpha-fetoprotein in patients with hepatocellular carcinoma. Liver 1993;13(4):177-82. | Does not meet full eligibility criteria: pre-2005 |
| Kuromatsu, R., Tanaka, M., Shimauchi, Y., Shimada, M., Tanikawa, K., Watanabe, K. Usefulness of ED036 kit for measuring serum PIVKA-II levels in small hepatocellular carcinoma. Journal of gastroenterology 1997;32(4):507-12. | Does not meet full eligibility criteria: includes people without cirrhosis |
| Kurosaki, M., Hiramatsu, N., Sakamoto, M., Suzuki, Y., Iwasaki, M., Tamori, A., et al. Data mining model using simple and readily available factors could identify patients at high risk for hepatocellular carcinoma in chronic hepatitis C. Journal of Hepatology ;():n. pag.. | Exclude on study design: not a 1- or 2-gate test accuracy study |
| Kurosaki, M., Izumi, N., Onuki, Y., Nishimura, Y., Ueda, K., Tsuchiya, K., et al. Serum KL-6 as a novel tumor marker for hepatocellular carcinoma in hepatitis C virus infected patients. Hepatology Research 2005;33(3):250-57. | Exclude on index test |
| Kwon, Mi-Hye, Kong, Deok-Hoon, Jung, Se-Hui, Suh, In-Bum, Kim, Young-Myeong. Rapid determination of blood coagulation factor XIII activity using protein arrays for serodiagnosis of human plasma. Analytical chemistry 2011;83(6):2317-23. | Exclude on index test |
| Ladep, Nimzing G., Dona, Anthony C., Lewis, Matthew R., Crossey, Mary M. E., Lemoine, Maud, Okeke, Edith, et al. Discovery and validation of urinary metabotypes for the diagnosis of hepatocellular carcinoma in West Africans. Hepatology (Baltimore, Md.) 2014;60(4):1291-3001. | Does not meet full eligibility criteria: includes people without cirrhosis |
| Lamerz, R., Runge, M., Stieber, P. Use of serum PIVKA-II (DCP) determination for differentiation between benign and malignant liver diseases. Anticancer research 1999;19(4A):2489-93. | Does not meet full eligibility criteria: pre-2005 |
| Laroia, S. T., Bhadoria, Ajeet Singh, Venigalla, Yamini, Chibber, G. K., Bihari, Chagan, Rastogi, Archana. Role of dual energy spectral computed tomography in characterization of hepatocellular carcinoma: Initial experience from a tertiary liver care institute. European journal of radiology open 2016;3():162-71. | Exclude on population: clinical signs and symptoms of HCC |
| Laroia, Shalini Thapar, Bawa, Simranjeet Singh, Jain, Deepak, Mukund, Amar. Contrast ultrasound in hepatocellular carcinoma at a tertiary liver center: First Indian experience. World journal of radiology 2013;5(6):229-40. | Exclude on population: clinical signs and symptoms of HCC |
| Le Moigne, Francois, Durieux, Marion, Bancel, Brigitte, Boublay, Nawele, Boussel, Loic, Ducerf, Christian, Berthezene, Yves. Impact of diffusion-weighted MR imaging on the characterization of small hepatocellular carcinoma in the cirrhotic liver. Magnetic resonance imaging 2012;30(5):656-65. | Exclude on population: clinical signs and symptoms of HCC |
| Leandro, G., Basso, D., Fabris, C., Zizzari, S., Elba, S., Del Favero, G., et al. Alpha-fetoprotein, tissue polypeptide antigen and ferritin in diagnosing primary hepatocellular carcinoma in patients with liver cirrhosis. Journal of cancer research and clinical oncology 1989;115(3):276-78. | Does not meet full eligibility criteria: pre-2005 |
| Leandro, G., Zizzari, S., Fabris, C., Basso, D., Elba, S., Del Favero, G., et al. Do CA 19-9 and TPA play a minor role as compared to AFP in diagnosing primary hepatocellular carcinoma? Oncology 1989;46(6):381-85. | Does not meet full eligibility criteria: pre-2005 |
| Lee, Chung Seop, Jung, Yong Jin, Kim, Soon Sun, Cheong, Jae Youn, Lee, Ga Ram, Kim, Han Gyeol, et al. Liver volume-based prediction model stratifies risks for hepatocellular carcinoma in chronic hepatitis B patients on surveillance. PloS one 2018;13(1):e0190261. | Exclude on index test |

| Reference | Reason for exclusion |
|---|---|
| Lee, Dong Ho, Lee, Jeong Min, Baek, Jee Hyun, Shin, Cheong-Il, Han, Joon Koo. Diagnostic performance of gadoxetic acid-enhanced liver MR imaging in the detection of HCCs and allocation of transplant recipients on the basis of the Milan criteria and UNOS guidelines: correlation with histopathologic findings. Radiology 2015;274(1):149-60. | Exclude on population: not cirrhosis |
| Lee, E., Edward, S., Singal, A. G., Lavieri, M. S. Improving screening for hepatocellular carcinoma by incorporating data on levels of alpha-fetoprotein, over time. Clinical Gastroenterology and Hepatology 2013;11(4):437-40. | Exclude on population: not cirrhosis |
| Lee, F. Y., Lee, S. D., Tsai, Y. T., Wu, J. C., Lai, K. H. Serum C-reactive protein as a serum marker for the diagnosis of hepatocellular carcinoma. Cancer 1989;63(8):1567-71. | Exclude on population: not cirrhosis |
| Lee, H. B., Yoo, O. J., Ham, J. S. Serum alpha 1-antitrypsin in patients with hepatocellular carcinoma. Clinica chimica acta; international journal of clinical chemistry 1992;206(3):225-30. | Exclude on index test |
| Lee, H. S. & Chung, Y. H. Specificities of serum alpha-fetoprotein in HBsAg+ and HBsAg- patients in the diagnosis of hepatocellular carcinoma. Hepatology (Baltimore, Md.) 1991;14(1):68-72. | Does not meet full eligibility criteria: includes people without cirrhosis |
| Lee, Heon Young, Jung, Jae Hoon, Kang, Yoon Sae, Kim, Yeon Soo, Moon, Hee Seok, Park, Ki Oh, et al. [Clinical significance of transiently elevated serum AFP level in developing hepatocellular carcinoma in HBsAg positive-liver cirrhosis]. The Korean journal of gastroenterology = Taehan Sohwagi Hakhoe chi 2004;43(4):252-9. | Does not meet full eligibility criteria: pre-2005 |
| Lee, Heon Young, Jung, Jae Hoon, Kang, Yoon Sae, Kim, Yeon Soo, Moon, Hee Seok, Park, Ki Oh, et al. [Clinical significance of transiently elevated serum AFP level in developing hepatocellular carcinoma in HBsAg positive-liver cirrhosis]. The Korean journal of gastroenterology = Taehan Sohwagi Hakhoe chi 2004;43(4):252-59. | Does not meet full eligibility criteria: pre-2005 |
| Lee, Hye Won, Kim, Esl, Cho, Kyung Joo, Park, Hye Jung, Seo, Jieun, Lee, Hyeonah, et al. Applications of molecular barcode sequencing for the detection of low-frequency variants in circulating tumour DNA from hepatocellular carcinoma. Liver international : official journal of the International Association for the Study of the Liver 2022;():n. pag.. | Exclude on population: clinical signs and symptoms of HCC |
| Lee, Hyun Jung, Yeon, Jong Eun, Suh, Sang Jun, Lee, Sun Jae, Yoon, Eileen L, Kang, Keunhee, et al. Clinical utility of plasma glypican-3 and osteopontin as biomarkers of hepatocellular carcinoma. Gut and liver 2014;8(2):177-85. | Exclude on population: not cirrhosis |
| Lee, Hyun Jung, Yeon, Jong Eun, Suh, Sang Jun, Lee, Sun Jae, Yoon, Eileen L., Kang, Keunhee, et al. Clinical utility of plasma glypican-3 and osteopontin as biomarkers of hepatocellular carcinoma. Gut and liver 2014;8(2):177-85. | Duplicate (including Cochrane includes) |
| Lee, Ji Young, Kim, Seong Hyun, Jeon, Yong Hwan, Lee, Jongmee, Kim, Min Ju, Choi, Dongil. Ferucarbotran-enhanced magnetic resonance imaging versus gadoxetic acid-enhanced magnetic resonance imaging for the preoperative detection of hepatocellular carcinoma: initial experience. Journal of computer assisted tomography 2010;34(1):127-34. | Exclude on population: clinical signs and symptoms of HCC |
| Lee, Jihyeon, Lim, Young-Suk, Lee, Jeong-Hoon, Gwak, Geum-Youn, Do, Misol, Yeo, Injoon, et al. Inclusive Quantification Assay of Serum Des-gamma-Carboxyprothrombin Proteoforms for Hepatocellular Carcinoma Surveillance by Targeted Mass Spectrometry. Hepatology communications 2021;5(10):1767-83. | Does not meet full eligibility criteria: includes people without cirrhosis |
| Lee, Jung Hun, Kim, Youe Ree, Lee, Guy Mok, Ryu, Jong Hyun, Cho, Eun Young, Lee, Young Hwan. Coefficient of variation on Gd-EOB MR imaging: Correlation with the presence of early-stage hepatocellular carcinoma in patients with chronic hepatitis B. European journal of radiology 2018;102():95-101. | Exclude on population: clinical signs and symptoms of HCC |
| Lee, Mi Hee, Kim, Seong Hyun, Park, Min Jung, Park, Cheol Keun. Gadoxetic acid-enhanced hepatobiliary phase MRI and high-b-value diffusion-weighted imaging to distinguish well-differentiated hepatocellular carcinomas from benign nodules in patients with chronic liver disease. AJR. American journal of roentgenology 2011;197(5):W868-75. | Exclude on population: clinical signs and symptoms of HCC |
| Lee, Mi-Jin, Yu, Gyung-Ran, Park, Seon-Hwa, Cho, Baik-Hwan, Ahn, Jong-Seong, Park, Hae-Joon, Song, Eun-Young. Identification of cystatin B as a | Exclude on population: not cirrhosis |

| Reference | Reason for exclusion |
|---|---|
| potential serum marker in hepatocellular carcinoma. Clinical cancer research : an official journal of the American Association for Cancer Research 2008;14(4):1080-89. | |
| Lee, Qiuyan & Yu, Xixiang. The value of PIVKA-II versus AFP for the diagnosis and detection of postoperative changes in hepatocellular carcinoma. Journal of interventional medicine 2021;4(2):77-81. | Exclude on population: not cirrhosis |
| Lee, Si Eun, An, Chansik, Hwang, Shin Hye, Choi, Jin-Young, Han, Kyunghwa. Extracellular contrast agent-enhanced MRI: 15-min delayed phase may improve the diagnostic performance for hepatocellular carcinoma in patients with chronic liver disease. European radiology 2018;28(4):1551-59. | Exclude on population: clinical signs and symptoms of HCC |
| Lee, Taehee, Rawding, Piper A., Bu, Jiyoon, Hyun, Sunghee, Rou, Woosun, Jeon, Hongjae, et al. Machine-Learning-Based Clinical Biomarker Using Cell-Free DNA for Hepatocellular Carcinoma (HCC). Cancers 2022;14(9):n. pag.. | Does not meet full eligibility criteria: includes people without cirrhosis |
| Lee, Young-Sun, Ko, Eunjung, Yoon, Eileen L., Jung, Young Kul, Kim, Ji Hoon, Seo, Yeon Seok, et al. Multiplexed Proteomic Approach for Identification of Serum Biomarkers in Hepatocellular Carcinoma Patients with Normal AFP. Journal of clinical medicine 2020;9(2):n. pag.. | Exclude on index test |
| Leerapun, Apinya, Suravarapu, Sri V., Bida, John P., Clark, Raynell J., Sanders, Elizabeth L., Mettler, Teresa A., et al. The utility of Lens culinaris agglutinin-reactive alpha-fetoprotein in the diagnosis of hepatocellular carcinoma: evaluation in a United States referral population. Clinical gastroenterology and hepatology : the official clinical practice journal of the American Gastroenterological Association 2007;5(3):394-267. | Does not meet full eligibility criteria: includes people without cirrhosis |
| Lefrere, J. J., Conard, J., Mavier, P., Bettan, L., Beaugrand, M., Gozin, D., et al. Coagulation assays as diagnostic markers of hepatocellular carcinoma. Thrombosis and haemostasis 1988;60(3):468-70. | Does not meet full eligibility criteria: includes people without cirrhosis |
| Lei, J., Xu, Y., Jiang, S., Tian, S. Automated detection of small hepatocellular carcinoma in cirrhotic livers: applying deep learning to Gd-EOB-DTPA-enhanced MRI. Abdominal Radiology 2025;50(9):4087 “ 4099. | No 2x2 data |
| Lei, Y., Lu, X., Duan, X., Tang, W. Bilirubin is a superior biomarker for hepatocellular carcinoma diagnosis and for differential diagnosis of benign liver disease. Journal of Laboratory Medicine 2023;():n. pag.. | Does not meet full eligibility criteria: includes people without cirrhosis |
| Leoni, S., Piscaglia, F., Granito, A., Borghi, A., Galassi, M., Marinelli, S., Terzi, E. Characterization of primary and recurrent nodules in liver cirrhosis using contrast-enhanced ultrasound: which vascular criteria should be adopted? Ultraschall in der Medizin (Stuttgart, Germany : 1980) 2013;34(3):280-87. | Exclude on population: clinical signs and symptoms of HCC |
| Leoni, Simona, Piscaglia, Fabio, Golfieri, Rita, Camaggi, Valeria, Vidili, Gianpaolo, Pini, Patrizia. The impact of vascular and nonvascular findings on the noninvasive diagnosis of small hepatocellular carcinoma based on the EASL and AASLD criteria. The American journal of gastroenterology 2010;105(3):599-609. | Exclude on population: clinical signs and symptoms of HCC |
| Lersritwimanmaen, Patharapan. Hepatocellular Carcinoma Surveillance: Benefit of Serum Alfa-fetoprotein in Real-world Practice. Euroasian journal of hepato-gastroenterology 2018;8(1):83-87. | Does not meet full eligibility criteria: includes people without cirrhosis |
| Lewinska, M., Santos-Laso, A., Arretxe, E., Alonso, C., Zhuravleva, E., Jimenez-Aguero, R., et al. The altered serum lipidome and its diagnostic potential for Non-Alcoholic Fatty Liver (NAFL)-associated hepatocellular carcinoma: Diagnosis of NAFLD-HCC utilising serum lipidomics. EBioMedicine 2021;73():103661. | Exclude on population: not cirrhosis |
| Lewis, Sara, Kamath, Amita, Chatterji, Manjil, Patel, Amish, Shyknevsky, Inna, Dyvorne, Hadrien A., Kuehn, Bernd. Diffusion-weighted imaging of the liver in patients with chronic liver disease: comparison of monopolar and bipolar diffusion gradients for image quality and lesion detection. AJR. American journal of roentgenology 2015;204(1):59-68. | Does not meet full eligibility criteria: includes people without cirrhosis |
| Li, B., Guo, T., Sun, Z., Li, X., Chen, L., Chen, W., et al. ITIH4: Effective Serum Marker, Early Warning and Diagnosis, Hepatocellular Carcinoma. Pathology and Oncology Research 2018;24(3):663-70. | No 2x2 data |
| Li, B., Guo, T., Sun, Z., Li, X., Chen, L., Zhao, J. Artificial neural network models for early diagnosis of hepatocellular carcinoma using serum levels of | Does not meet full eligibility criteria: includes people without cirrhosis |

| Reference | Reason for exclusion |
|---|---|
| alpha-fetoprotein, a-fetoprotein-L3, des-gamma-carboxy prothrombin, and Golgi protein 73. Oncotarget 2017;8(46):80521-30. | |
| Li, B., Hao, K., Li, M., Wang, A., Tang, H., Xu, L., et al. Five miRNAs identified in fucosylated extracellular vesicles as non-invasive diagnostic signatures for hepatocellular carcinoma. Cell Reports Medicine 2024;5(9):101716. | Exclude on population: not cirrhosis |
| Li, B., Hao, K., Ma, C., Li, Z., Li, H., Du, W., et al. Isolation and characterization of fucosylated extracellular vesicles based on a novel high-throughput GlyExo-Capture technique. bioRxiv 2021;():n. pag.. | Exclude on population: not cirrhosis |
| Li, B., Liu, H., Shang, H. W., Li, P., Li, N. Diagnostic value of glypican-3 in alpha fetoprotein negative hepatocellular carcinoma patients. African health sciences 2013;13(3):703-09. | Exclude on population: healthy controls |
| Li, Bao-ding, Zhao, Qing-chuan, Zhu, Yang-ting, Zhang, Fu-qin. [Significance of glypican-3 mRNA expression in hepatocellular carcinoma tissues and peripheral blood cells]. Zhonghua wai ke za zhi [Chinese journal of surgery] 2006;44(7):458-62. | Foreign language |
| Li, Baoliang, Huang, Hao, Huang, Ronghai, Zhang, Wei, Zhou, Guangpeng, Wu, Zhen, et al. SEPT9 Gene Methylation as a Noninvasive Marker for Hepatocellular Carcinoma. Disease markers 2020;2020():6289063. | Does not meet full eligibility criteria: includes people without cirrhosis |
| Li, Bing, Su, Huiting, Cao, Ju. CXCL13 rather than IL-31 is a potential indicator in patients with hepatocellular carcinoma. Cytokine 2017;89():91-97. | Exclude on population: healthy controls |
| Li, Bo, Li, Boan, Guo, Tongsheng, Sun, Zhiqiang, Li, Xiaohan, Li, Xiaoxi, et al. Artificial neural network models for early diagnosis of hepatocellular carcinoma using serum levels of alpha-fetoprotein, alpha-fetoprotein-L3, des-gamma-carboxy prothrombin, and Golgi protein 73. Oncotarget 2017;8(46):80521-30. | Does not meet full eligibility criteria: includes people without cirrhosis |
| Li, Bo, Li, Boan, Guo, Tongsheng, Sun, Zhiqiang, Li, Xiaohan, Li, Xiaoxi, et al. The Clinical Values of Serum Markers in the Early Prediction of Hepatocellular Carcinoma. BioMed research international 2017;2017(101600173):5358615. | Exclude on study design: not a 1- or 2-gate test accuracy study |
| Li, Bo, Zhao, Youyun, Cai, Wangxi, Ming, Anping. Validation and update of a multivariable prediction model for the identification and management of patients at risk for hepatocellular carcinoma. Clinical proteomics 2021;18(1):21. | Exclude on population: not cirrhosis |
| Li, D., Zhang, J. Z., Zheng, Y. H., Ji, X. L., You, H., Shu, Q. M., Fan, L. N. Using protein chip technology to screen for tissue proteomic profiles and tumor markers in hepatocellular carcinoma. World Chinese Journal of Digestology 2007;15(22):2424-30. | Foreign language |
| Li, F., Bao, Q. W., Jiang, L. Y. Q., Huang, Q. L., Zhang, X. Diagnostic role of microRNA-125b for hepatocellular carcinoma. Hepatobiliary and Pancreatic Diseases International 2019;18(4):395-97. | Exclude on study design: not a 1- or 2-gate test accuracy study |
| Li, F., Fan, Y. C., Gao, S., Sun, F. K., Yang, Y. Methylation of serum insulin-like growth factor-binding protein 7 promoter in hepatitis B virus-associated hepatocellular carcinoma. Genes Chromosomes and Cancer 2014;53(1):90-97. | Exclude on population: not cirrhosis |
| Li, G. J., Chen, Q. Y., Harrison, T. J., Wang, X. Y., Hu, L. P., Yang, Q. L., Li, K. W. Des-gamma carboxyprothrombin may not be a good biomarker for hepatocellular carcinoma in those chronically infected with hepatitis B virus with basal core promoter double mutations (T1762, A1764), a prospective study. Cancer Biomarkers 2017;18(3):241-48. | Duplicate (including Cochrane includes) |
| Li, G., Shen, Q., Li, C., Li, D., Chen, J. Identification of circulating MicroRNAs as novel potential biomarkers for hepatocellular carcinoma detection: a systematic review and meta-analysis. Clinical & translational oncology : official publication of the Federation of Spanish Oncology Societies and of the National Cancer Institute of Mexico 2015;17(9):684-93. | Exclude on study design: not a 1- or 2-gate test accuracy study |
| Li, Gang, Shi, Hao, Wang, Xinyi, Wang, Bei, Qu, Qianqian, Geng, Haiyang. Identification of diagnostic long non-coding RNA biomarkers in patients with hepatocellular carcinoma. Molecular medicine reports 2019;20(2):1121-30. | Exclude on population: not cirrhosis |
| Li, Guo-Jian, Chen, Qin-Yan, Harrison, Tim J, Wang, Xue-Yan, Hu, Li-Ping, Yang, Qing-Li, Li, Kai-Wen. Des-gamma carboxyprothrombin may not be a good biomarker for hepatocellular carcinoma in those chronically infected with hepatitis B virus with basal core promoter double mutations (T^{1762}, A^{1764}), a prospective study. Cancer biomarkers : section A of Disease markers 2017;18(3):241-48. | Exclude on population: not cirrhosis |

| Reference | Reason for exclusion |
|---|---|
| Li, H. & Liu, Z. Role of AFP, AFP-L3, PIVKA-II, and CA199 in diagnosis and prognosis evaluation of liver cancer. International Journal of Clinical and Experimental Medicine 2020;13(12):9778-85. | Exclude on population: not cirrhosis |
| Li, H., Cui, M. L., Chen, T. Y., Xie, H. Y., Cui, Y., Tu, H., et al. Serum DLK1 is a potential prognostic biomarker in patients with hepatocellular carcinoma. Tumor Biology 2015;36(11):8399-4004. | No 2x2 data |
| Li, H., Tang, X. M., Liu, Y., Li, W., Chen, Q. Association of Functional Genetic Variants of HOTAIR with Hepatocellular Carcinoma (HCC) Susceptibility in a Chinese Population. Cellular Physiology and Biochemistry 2017;44(2):447-54. | Exclude on population: not cirrhosis |
| Li, J. MiR-145-5p inhibits proliferation of hepatocellular carcinoma, acting through PAI-1. American Journal of Translational Research 2025;17(2):888 “ 896. | No 2x2 data |
| Li, J. P., Zhao, D. L., Jiang, H. J., Huang, Y. H., Li, D. Q., Wan, Y., Liu, X. D. Assessment of tumor vascularization with functional computed tomography perfusion imaging in patients with cirrhotic liver disease. Hepatobiliary and Pancreatic Diseases International 2011;10(1):43-49. | Exclude on study design: not a 1- or 2-gate test accuracy study |
| Li, Jian, Jin, Boxun, Wang, Tiezheng, Li, Wenlei, Wang, Zhenshun, Zhang, Haitao, Song, Yunjun. Serum microRNA expression profiling identifies serum biomarkers for HCV-related hepatocellular carcinoma. Cancer biomarkers : section A of Disease markers 2019;26(4):501-12. | No 2x2 data |
| Li, Jian, Qiyu, Sun, Wang, Tiezheng, Jin, Boxun. Improving the Detection of Hepatocellular Carcinoma Using Serum AFP Expression in Combination with GPC3 and Micro-RNA MiR-122 Expression. Open life sciences 2019;14():53-61. | Does not meet full eligibility criteria: includes people without cirrhosis |
| Li, Jianbiao, Shi, Wenxia, Gao, Yingtang, Yang, Bin, Jing, Xiang, Shan, Shigang, Wang, Yijun. Analysis of microRNA expression profiles in human hepatitis B virus-related hepatocellular carcinoma. Clinical laboratory 2013;59(9-10):1009-15. | Exclude on population: tissue samples |
| Li, Jiang, Tao, Haisu, Zhang, Erlei. Diagnostic value of gamma-glutamyl transpeptidase to alkaline phosphatase ratio combined with gamma-glutamyl transpeptidase to aspartate aminotransferase ratio and alanine aminotransferase to aspartate aminotransferase ratio in alpha-fetoprotein-negative hepatocellular carcinoma. Cancer medicine 2021;10(14):4844-54. | Exclude on index test |
| Li, Jiaqi, Wei, Lei, Zhang, Xianglin, Zhang, Wei, Wang, Haochen, Zhong, Bixi, et al. DISMIR: Deep learning-based noninvasive cancer detection by integrating DNA sequence and methylation information of individual cell-free DNA reads. Briefings in bioinformatics 2021;22(6):n. pag.. | Exclude on population: not cirrhosis |
| Li, Jia-Wu, Ling, Wen-Wu, Lu, Qiang, Lu, Chang-Li, He, Du. Liver Stiffness and Serum Alpha-Fetoprotein in Discriminating Small Hepatocellular Carcinoma from Cirrhotic Nodule. Ultrasound quarterly 2016;32(4):319-26. | Exclude on population: clinical signs and symptoms of HCC |
| Li, Jie. [Significance of arginase-1, glypican-3, hepatocyte paraffin antigen 1 and alpha-fetoprotein in diagnosis and differential diagnosis of liver tumors]. Zhonghua bing li xue za zhi = Chinese journal of pathology 2014;43(4):246-50. | Foreign language |
| Li, Jin-Ping, Zhao, De-Li, Jiang, Hui-Jie, Huang, Ya-Hua, Li, Da-Qing, Wan, Yong, Liu, Xin-Ding. Assessment of tumor vascularization with functional computed tomography perfusion imaging in patients with cirrhotic liver disease. Hepatobiliary & pancreatic diseases international : HBPD INT 2011;10(1):43-49. | Exclude on study design: not a 1- or 2-gate test accuracy study |
| Li, Jun, Cheng, Zhang-Jun, Liu, Yang, Yan, Zhen-Lin, Wang, Kui, Wu, Dong, et al. Serum thioredoxin is a diagnostic marker for hepatocellular carcinoma. Oncotarget 2015;6(11):9551-63. | Exclude on population: not cirrhosis |
| Li, Jun, Wang, Xiaochen, Tang, Junwei, Jiang, Runqiu, Zhang, Wenjie, Ji, Jie. HULC and Linc00152 Act as Novel Biomarkers in Predicting Diagnosis of Hepatocellular Carcinoma. Cellular physiology and biochemistry : international journal of experimental cellular physiology, biochemistry, and pharmacology 2015;37(2):687-96. | Exclude on population: healthy controls |
| Li, Kang, Song, Yi, Qin, Ling, Li, Ang, Jiang, Sanjie, Ren, Lei, et al. A CpG Methylation Signature as a Potential Marker for Early Diagnosis of Hepatocellular Carcinoma From HBV-Related Liver Disease Using Multiplex Bisulfite Sequencing. Frontiers in oncology 2021;11():756326. | Does not meet full eligibility criteria: includes people without cirrhosis |

| Reference | Reason for exclusion |
|---|---|
| Li, Liang, Chen, Jianguo, Chen, Xin, Tang, Jing, Guo, Huan, Wang, Xiaofeng, et al. Serum miRNAs as predictive and preventive biomarker for pre-clinical hepatocellular carcinoma. Cancer letters 2016;373(2):234-40. | Exclude on population: not cirrhosis |
| Li, Liang, Chen, Jianguo, Chen, Xin, Tang, Jing, Guo, Huan, Wang, Xiaofeng, et al. Serum miRNAs as predictive and preventive biomarker for pre-clinical hepatocellular carcinoma. Cancer letters 2016;373(2):234-40. | Duplicate (including Cochrane includes) |
| Li, Lihua, Guo, Zijan, Wang, Juanhua, Mao, Yong. Serum miR-18a: a potential marker for hepatitis B virus-related hepatocellular carcinoma screening. Digestive diseases and sciences 2012;57(11):2910-16. | Exclude on population: not cirrhosis |
| Li, Li-Min, Hu, Zhi-Bin, Zhou, Zhen-Xian, Chen, Xi, Liu, Fen-Yong, Zhang, Jun-Feng, et al. Serum microRNA profiles serve as novel biomarkers for HBV infection and diagnosis of HBV-positive hepatocarcinoma. Cancer research 2010;70(23):9798-8007. | Exclude on population: not cirrhosis |
| Li, Lingling, Zheng, Wei, Wang, Jianwei, Han, Jing, Guo, Zhixing, Hu, Yixin, Li, Xiaoxian. Contrast-Enhanced Ultrasound Using Perfluorobutane: Impact of Proposed Modified LI-RADS Criteria on Hepatocellular Carcinoma Detection. AJR. American journal of roentgenology 2022;():1-10. | Exclude on population: clinical signs and symptoms of HCC |
| Li, M., Ni, R., Huang, J., Xiao, M., Zhang, H., Wei, Q., Jiang, F. The value of serum GPDA-F for the diagnosis of primary hepatocellular carcinoma. Zhonghua gan zang bing za zhi = Zhonghua ganzangbing zazhi = Chinese journal of hepatology 2000;8(3):139-41. | Does not meet full eligibility criteria: pre-2005 |
| Li, N., Fan, X., Wang, X., Deng, H., Zhang, K., Zhang, X., et al. PRDM1 levels are associated with clinical diseases in chronic HBV infection and survival of patients with HBV-related hepatocellular carcinoma. International Immunopharmacology 2019;73():156-62. | Exclude on index test |
| Li, Na, Zhu, Qianqian, Yang, Cuiling, Li, Fang, Zhou, Zhihua, Lv, Yi, et al. Elevated serum soluble CD14 levels in chronic HBV infection are significantly associated with HBV-related hepatocellular carcinoma. Tumour biology : the journal of the International Society for Oncodevelopmental Biology and Medicine 2016;37(5):6607-17. | No 2x2 data |
| Li, P., Zhai, Y., Liu, H., Lv, F. D., Li, N. Diagnostic value of serum AFP alone or in combination with glypican 3, VEGF or IGF-II for patients with primary hepatocellular carcinoma. World Chinese Journal of Digestology 2010;18(25):2702-06. | Foreign language |
| Li, Ran, Shui, Liyan, Jia, Junling. Construction and Validation of Novel Diagnostic and Prognostic DNA Methylation Signatures for Hepatocellular Carcinoma. Frontiers in genetics 2020;11():906. | Exclude on population: not cirrhosis |
| Li, S., Deng, K., Qiu, J., Wang, P., Yin, D., Xie, Y. ghted imaging; Based on Gadolinium Ethoxybenzyl DTPA-Enhanced MRI: Diagnostic Performance of the Category-Modified LR-5 Criteria in Patients At Risk for Hepatocellular Carcinoma. ghted imaging; Technology in Cancer Research and Treatment 2024;23():n. pag.. | Does not meet full eligibility criteria: includes people without cirrhosis |
| Li, Siwen, Li, Xiaojin, Xu, Anjian, Zhang, Bei, He, Xiaomin, Chen, Hongda. Screening and clinical evaluation of dominant peptides of centromere protein F antigen for early diagnosis of hepatocellular carcinoma. Molecular medicine reports 2018;17(3):4720-28. | Does not meet full eligibility criteria: includes people without cirrhosis |
| Li, Tao, Li, Hongguang, Wang, Aihua, Su, Xiaoyan, Zhao, Jingfang, Cui, Yi, Liu, Jun. Development And Validation Of A Simple Model For Detection Of Early Hepatocellular Carcinoma In A Liver Cirrhosis Cohort. Cancer management and research 2019;11():9379-86. | Duplicate (including Cochrane includes) |
| Li, Tao, Yin, Jikai, Yuan, Lijuan, Wang, Shouli, Yang, Lin, Du, Xilin. Downregulation of microRNA-139 is associated with hepatocellular carcinoma risk and short-term survival. Oncology reports 2014;31(4):1699-7006. | Exclude on population: not cirrhosis |
| Li, X. & Wu, K. Serum Golgi phosphoprotein 2 level: A better marker than alpha-fetoprotein for diagnosing early hepatocellular carcinoma (Hepatology (2009) 50, 1, (325) DOI:10.1002/hep.23028). Hepatology 2009;50(5):1682. | Exclude on study design: not a 1- or 2-gate test accuracy study |
| Li, X., Gu, X., Li, L., Xiang, L., Jiang, Z., Zhang, Y., et al. Integrated microbiome and metabolome analysis reveals the interaction between intestinal flora and serum metabolites as potential biomarkers in hepatocellular carcinoma patients. medRxiv 2022;():n. pag.. | Does not meet full eligibility criteria: includes people without cirrhosis |

| Reference | Reason for exclusion |
|---|---|
| Li, X., Li, Y., Li, X., Jiang, L. N., Zhu, L., Lu, F. M. [A preliminary discussion on carnosine dipeptidase 1 as a potential novel biomarker for the diagnostic and prognostic evaluation of hepatocellular carcinoma]. Zhonghua gan zang bing za zhi = Zhonghua ganzangbing zazhi = Chinese journal of hepatology 2023;31(6):627-33. | Foreign language |
| Li, Xiaohan, Li, Bo, Li, Boan, Guo, Tongsheng, Sun, Zhiqiang, Li, Xiaoxi, et al. ITIH4: Effective Serum Marker, Early Warning and Diagnosis, Hepatocellular Carcinoma. Pathology oncology research : POR 2018;24(3):663-70. | No 2x2 data |
| Li, Xinyu, Liu, Yuanjie, Fan, Yunpeng, Tian, Gang, Shen, Bo, Zhang, Songzhi, et al. Advanced Nanoencapsulation-Enabled Ultrasensitive Analysis: Unraveling Tumor Extracellular Vesicle Subpopulations for Differential Diagnosis of Hepatocellular Carcinoma via DNA Cascade Reactions. ACS nano 2024;18(17):11389â€“11403. | Does not meet full eligibility criteria: includes people without cirrhosis |
| Li, Xiuping, Guo, Yuzhen, Wang, Xiyan, Ge, Anning, Wang, Hui, Fan, Kaiyun. Clinical significance of serum miR-487b in HBV-related hepatocellular carcinoma and its potential mechanism. Infectious diseases (London, England) 2021;53(7):546-54. | Exclude on population: not cirrhosis |
| Li, Xu & Xu, Hongqin. Fibrosis Index Based on 4 Factors (FIB-4) Predicts Liver Cirrhosis and Hepatocellular Carcinoma in Chronic Hepatitis C Virus (HCV) Patients. Medical science monitor : international medical journal of experimental and clinical research 2019;25():7243-50. | Exclude on population: not cirrhosis |
| Li, Xue-Qin, Wang, Xing, Zhao, Da-Wei, Sun, Jun, Liu, Jiao-Jiao, Lin, Dong-Dong, et al. Application of Gd-EOB-DTPA-enhanced magnetic resonance imaging (MRI) in hepatocellular carcinoma. World journal of surgical oncology 2020;18(1):219. | Exclude on study design: not a 1- or 2-gate test accuracy study |
| Li, Y., Li, S., Li, Q., Li, K., Han, J., Mao, S., et al. nner; Combination of CT/MRI LI-RADS With Second-Line Contrast-Enhanced Ultrasound Using Sulfur Hexafluoride or Perfluorobutane for Diagnosing Hepatocellular Carcinoma in High-Risk Patients. Korean Journal of Radiology 2025;26(3):e29. | Exclude on population: clinical signs and symptoms of HCC |
| Li, Ying, Li, Ronghua, Cheng, Da, Fu, Xiaoyu, Fu, Lei. The potential of CircRNA1002 as a biomarker in hepatitis B virus-related hepatocellular carcinoma. PeerJ 2022;10():e13640. | Exclude on population: not cirrhosis |
| Li, Zhiqin, Hu, Yushu, Wang, Hongyan, Wang, Meng, Gu, Xinyu, Ping, Yu, et al. Predictors for the progression of hepatic cirrhosis to hepatocellular carcinoma under long-term antiviral therapy. European journal of gastroenterology & hepatology 2020;32(3):447-53. | No 2x2 data |
| Li, Zuhua, Zhou, Ye, Yang, Guohua, He, Siying, Qiu, Xueping, Zhang, Lei, Deng, Qianyun. Using circular RNA SMARCA5 as a potential novel biomarker for hepatocellular carcinoma. Clinica chimica acta; international journal of clinical chemistry 2019;492():37-44. | Does not meet full eligibility criteria: includes people without cirrhosis |
| Lian, S., Lu, C., Li, F., Yu, X., Ai, L., Wu, B., et al. Monitoring Hepatocellular Carcinoma Using Tumor Content in Circulating Cell-Free DNA. Clinical Cancer Research 2024;30(13):2772 “ 2779. | Exclude on population: not cirrhosis |
| Liang, Hongyan, Block, Timothy M., Wang, Mengjun, Nefsky, Bradley, Long, Ronald, Hafner, Julie, et al. Interleukin-6 and oncostatin M are elevated in liver disease in conjunction with candidate hepatocellular carcinoma biomarker GP73. Cancer biomarkers : section A of Disease markers 2012;11(4):161-71. | Exclude on index test |
| Liang, Jing, Zhu, Jianhui, Wang, Mengmeng, Singal, Amit G., Odewole, Mobolaji, Kagan, Sofia, et al. Evaluation of AGP Fucosylation as a Marker for Hepatocellular Carcinoma of Three Different Etiologies. Scientific reports 2019;9(1):11580. | Does not meet full eligibility criteria: includes people without cirrhosis |
| Liang, R., Luo, X. L., Ge, L. Y., Yuan, W. P., Yue, H. F., Jia, B. C. The diagnostic value of serum Golgi protein-73 in hepatocellular carcinoma. Tumor 2012;32(2):115-18. | Foreign language |
| Liang, Y. & Wu, H. nner; Development and validation of a CT-based nomogram for accurate hepatocellular carcinoma detection in high risk patients. Frontiers in Oncology 2024;14():1374373. | Exclude on population: clinical signs and symptoms of HCC |
| Liang, Yingying, Xu, Fan, Guo, Yuan, Lai, Lisha, Jiang, Xinqing, Wei, Xinhua, Wu, Hongzhen. Diagnostic performance of LI-RADS for MRI and CT detection | Exclude on study design: not a 1- or 2-gate test accuracy study |

| Reference | Reason for exclusion |
|---|---|
| of HCC: A systematic review and diagnostic meta-analysis. European journal of radiology 2021;134():109404. | |
| Liangpunsakul, S., Agarwal, D., Horlander, J. C., Kieff, B. Positron emission tomography for detecting occult hepatocellular carcinoma in hepatitis C cirrhotics awaiting for liver transplantation. Transplantation proceedings 2003;35(8):2995-97. | Exclude on population: clinical signs and symptoms of HCC |
| Liao, J., Zhang, R., Qian, H., Cao, L., Zhang, Y., Xu, W., et al. Serum profiling based on fucosylated glycoproteins for differentiating between chronic hepatitis B and hepatocellular carcinoma. Biochemical and Biophysical Research Communications 2012;420(2):308-14. | Duplicate (including Cochrane includes) |
| Liao, Jian, Zhang, Ruixiu, Qian, Haihua, Cao, Lu, Zhang, Yu, Xu, Wen, et al. Serum profiling based on fucosylated glycoproteins for differentiating between chronic hepatitis B and hepatocellular carcinoma. Biochemical and biophysical research communications 2012;420(2):308-14. | Does not meet full eligibility criteria: includes people without cirrhosis |
| Liao, W., Mao, Y., Ge, P., Yang, H., Xu, H., Lu, X., Sang, X. Value of quantitative and qualitative analyses of circulating cell-free DNA as diagnostic tools for hepatocellular carcinoma. Medicine (United States) 2015;94(14):n. pag.. | Exclude on study design: not a 1- or 2-gate test accuracy study |
| Liaw, Y. F. & Tai, D. I. Alpha-fetoprotein changes in the course of chronic hepatitis: Relation to bridging hepatic necrosis and hepatocellular carcinoma. Liver 1986;6(3):133-37. | Does not meet full eligibility criteria: pre-2005 |
| Libbrecht, Louis, Bielen, Didier, Verslype, Chris, Vanbeckevoort, Dirk, Pirenne, Jacques, Nevens, Frederik, Desmet, Valeer. Focal lesions in cirrhotic explant livers: pathological evaluation and accuracy of pretransplantation imaging examinations. Liver transplantation : official publication of the American Association for the Study of Liver Diseases and the International Liver Transplantation Society 2002;8(9):749-61. | Does not meet full eligibility criteria: pre-2005 |
| Libbrecht, Louis, Bielen, Didier, Verslype, Chris, Vanbeckevoort, Dirk, Pirenne, Jacques, Nevens, Frederik, Desmet, Valeer. Focal lesions in cirrhotic explant livers: pathological evaluation and accuracy of pretransplantation imaging examinations. Liver transplantation : official publication of the American Association for the Study of Liver Diseases and the International Liver Transplantation Society 2002;8(9):749-61. | Duplicate (including Cochrane includes) |
| Libbrecht, Louis, Severi, Tamara, Cassiman, David, Vander Borght, Sara, Pirenne, Jacques, Nevens, Frederik, et al. Glypican-3 expression distinguishes small hepatocellular carcinomas from cirrhosis, dysplastic nodules, and focal nodular hyperplasia-like nodules. The American journal of surgical pathology 2006;30(11):1405-11. | Exclude on population: clinical signs and symptoms of HCC |
| Liebman, H. A., Furie, B. C., Tong, M. J., Blanchard, R. A., Lo, K. J., Lee, S. D., Coleman, M. S. Des-gamma-carboxy (abnormal) prothrombin as a serum marker of primary hepatocellular carcinoma. The New England journal of medicine 1984;310(22):1427-31. | Does not meet full eligibility criteria: pre-2005 |
| Lim, D. Y. Z., Koh, H. K., Lim, J. J. X. Regression discontinuity analysis of alpha-fetoprotein with a cut-off of 20 ng/mL in hepatocellular carcinoma surveillance for chronic hepatitis B. GastroHep 2019;1(2):70-79. | No 2x2 data |
| Lim, J. H., Choi, D., Cho, S. K., Kim, S. H., Lee, W. J., Lim, H. K., et al. Conspicuity of hepatocellular nodular lesions in cirrhotic livers at ferumoxides-enhanced MR imaging: importance of Kupffer cell number. Radiology 2001;220(3):669-76. | Does not meet full eligibility criteria: pre-2005 |
| Lim, J. H., Kim, C. K., Lee, W. J., Park, C. K., Koh, K. C., Paik, S. W. Detection of hepatocellular carcinomas and dysplastic nodules in cirrhotic livers: accuracy of helical CT in transplant patients. AJR. American journal of roentgenology 2000;175(3):693-98. | Does not meet full eligibility criteria: pre-2005 |
| Lim, Jae Hoon, Kim, Min Ju, Chiang, Liu Wei, Lim, Hyo Keun, Park, Cheol Keun, Paik, Seung Woon, Koh, Kwang Cheol. CT detection of hepatocellular carcinoma in advanced liver cirrhosis: correlation of helical CT and explanted liver. Taehan Kan Hakhoe chi = The Korean journal of hepatology 2002;8(2):201-08. | Does not meet full eligibility criteria: pre-2005 |
| Lim, Tae Seop, Kim, Do Young, Han, Kwang-Hyub, Kim, Hyon-Suk, Shin, Seung Hwan, Jung, Kyu Sik, et al. Combined use of AFP, PIVKA-II, and AFP- | Duplicate (including Cochrane includes) |

| Reference | Reason for exclusion |
|---|---|
| L3 as tumor markers enhances diagnostic accuracy for hepatocellular carcinoma in cirrhotic patients. Scandinavian journal of gastroenterology 2016;51(3):344-53. | |
| Lin, B., Zhang, W., Jiang, Y., Qin, Y., Mishra, P. K., Chen, J. Y., Zeng, Y. D. Diagnostic performance of LR-5 based on hypointensity on Gd-EOB-DTPA-enhanced MRI in the hepatobiliary phase for sHCC using LI-RADS v2018 criteria. Clinical Radiology 2025;81():106784. | Exclude on population: clinical signs and symptoms of HCC |
| Lin, Daniel, Luo, Rui, Ye, Zhong, Wei, Qiang, Bae, Ho, Juon, Hee-Soon, et al. Genomic characterization of early-stage hepatocellular carcinoma patients with Hepatitis B using circulating tumor DNA. Clinics and research in hepatology and gastroenterology 2023;47(7):102161. | Does not meet full eligibility criteria: includes people without cirrhosis |
| Lin, H., Jiao, X., Yu, B., Du, J., Xu, H., Dong, A. Clinical significance of serum 14-3-3 beta in patients with hepatocellular carcinoma. Cancer Biomarkers 2017;20(2):143-50. | No 2x2 data |
| Lin, Huajun. Diagnostic value of a microRNA signature panel in exosomes for patients with hepatocellular carcinoma. International journal of clinical and experimental pathology 2019;12(4):1478-87. | No 2x2 data |
| Lin, Jie, Lin, Wansong, Bai, Yannan, Liao, Yanling, Lin, Qiaoyan, Chen, Lingfeng. Identification of exosomal hsa-miR-483-5p as a potential biomarker for hepatocellular carcinoma via microRNA expression profiling of tumor-derived exosomes. Experimental cell research 2022;417(2):113232. | Exclude on population: healthy controls |
| Lin, M. T., Chang, K. C., Chou, Y. P., Tseng, P. L., Yen, Y. H., Wang, C. C., et al. The validation of the 2010 American Association for the Study of Liver Diseases guideline for the diagnosis of hepatocellular carcinoma in an endemic area. Journal of Gastroenterology and Hepatology (Australia) 2015;30(2):345-51. | Exclude on population: clinical signs and symptoms of HCC |
| Lin, Ming-Tzung, Chen, Chao-Long, Wang, Chih-Chi, Cheng, Yu-Fan, Eng, Hock-Liew, Wang, Jing-Houng, et al. Diagnostic sensitivity of hepatocellular carcinoma imaging and its application to non-cirrhotic patients. Journal of gastroenterology and hepatology 2011;26(4):745-50. | Exclude on population: not cirrhosis |
| Lin, Nan, Lin, Yongping, Xu, Jianfeng, Liu, Dan, Li, Diange, Meng, Hongyu, et al. A multi-analyte cell-free DNA-based blood test for early detection of hepatocellular carcinoma. Hepatology communications 2022;6(7):1753-63. | Exclude on population: not cirrhosis |
| Lin, Q., Chen, L. B., Tang, Y. M. Promoter hypermethylation of p16 gene and DAPK gene in sera from hepatocellular carcinoma (HCC) patients. Chinese Journal of Cancer Research 2005;17(4):250-54. | Exclude on population: not cirrhosis |
| Lin, S. H., Lin, C. Y., Hsu, N. T., Yen, Y. H., Kee, K. M., Wang, J. H., et al. Reappraisal of the roles of alpha-fetoprotein in hepatocellular carcinoma surveillance using large-scale nationwide database and hospital-based information. Journal of the Formosan Medical Association 2022;():n. pag.. | Exclude on population: clinical signs and symptoms of HCC |
| Lin, S., Yang, X., Tang, M., Yao, X., Ye, Y., Huang, Q., et al. Monocyte distribution width as a promising biomarker for differential diagnosis of chronic hepatitis, cirrhosis, and hepatocellular carcinoma. Frontiers in Immunology 2024;15():1406671. | No 2x2 data |
| Lin, Shan-Zu, Chen, Chun-Chia, Lee, Kuei-Chuan, Tseng, Chih-Wei, Lin, Hsiao-Yi, Chen, Yi-Chou. DR-70 immunoassay for the surveillance of hepatocellular carcinoma. Journal of gastroenterology and hepatology 2012;27(3):547-52. | Exclude on population: not cirrhosis |
| Lin, Shu-Hsien, Lin, Chih-Yun, Hsu, Nien-Tzu, Yen, Yi-Hao, Kee, Kwong-Ming, Wang, Jing-Houng, et al. Reappraisal of the roles of alpha-fetoprotein in hepatocellular carcinoma surveillance using large-scale nationwide database and hospital-based information. Journal of the Formosan Medical Association = Taiwan yi zhi 2022;121(10):2085-92. | Does not meet full eligibility criteria: includes people without cirrhosis |
| Lin, Shu-Hsien, Lin, Chih-Yun, Hsu, Nien-Tzu, Yen, Yi-Hao, Kee, Kwong-Ming, Wang, Jing-Houng, et al. Reappraisal of the roles of alpha-fetoprotein in hepatocellular carcinoma surveillance using large-scale nationwide database and hospital-based information. Journal of the Formosan Medical Association = Taiwan yi zhi 2022;():n. pag.. | Exclude on population: clinical signs and symptoms of HCC |

| Reference | Reason for exclusion |
| --- | --- |
| Lin, Weizhen, He, Na, Zeng, Qingjin, Wu, Lili, Qiu, Shaodong. Efficacy of Multiple Modified Methods of Criteria for LR-M Liver Nodules of Different Sizes: Clinical Practice and Discussion in CEUS LI-RADS Version 2017. Journal of ultrasound in medicine : official journal of the American Institute of Ultrasound in Medicine 2023;42(12):2739â€“2748. | Exclude on population: clinical signs and symptoms of HCC |
| Lin, Xue-Jia, Chong, Yutian, Guo, Zhi-Wei, Xie, Chen, Yang, Xiao-Jing, Zhang, Qi, et al. A serum microRNA classifier for early detection of hepatocellular carcinoma: a multicentre, retrospective, longitudinal biomarker identification study with a nested case-control study. The Lancet. Oncology 2015;16(7):804-15. | Duplicate (including Cochrane includes) |
| Lin, Xue-Jia, Chong, Yutian, Guo, Zhi-Wei, Xie, Chen, Yang, Xiao-Jing, Zhang, Qi, et al. A serum microRNA classifier for early detection of hepatocellular carcinoma: a multicentre, retrospective, longitudinal biomarker identification study with a nested case-control study. The Lancet. Oncology 2015;16(7):804-15. | Does not meet full eligibility criteria: includes people without cirrhosis |
| Lin, Y., Zhang, J., Arroyo, A., Singal, A. G., Parikh, N. D. A Fucosylated Glycopeptide as a Candidate Biomarker for Early Diagnosis of NASH Hepatocellular Carcinoma Using a Stepped HCD Method and PRM Evaluation. Frontiers in Oncology 2022;12():818001. | Does not meet full eligibility criteria: includes people without cirrhosis |
| Lin, Yu, Zhu, Jianhui, Pan, Lingyun, Zhang, Jie, Tan, Zhijing, Olivares, Jocelyn, et al. A Panel of Glycopeptides as Candidate Biomarkers for Early Diagnosis of NASH Hepatocellular Carcinoma Using a Stepped HCD Method and PRM Evaluation. Journal of proteome research 2021;20(6):3278-89. | Does not meet full eligibility criteria: includes people without cirrhosis |
| Lin, Yu, Zhu, Jianhui, Zhang, Jie, Dai, Jianliang, Liu, Suyu, Arroyo, Ana, et al. Glycopeptides with Sialyl Lewis Antigen in Serum Haptoglobin as Candidate Biomarkers for Nonalcoholic Steatohepatitis Hepatocellular Carcinoma Using a Higher-Energy Collision-Induced Dissociation Parallel Reaction Monitoring-Mass Spectrometry Method. ACS omega 2022;7(26):22850-60. | Does not meet full eligibility criteria: includes people without cirrhosis |
| Lin, Yu-Ju, Chang, Chia-Ling, Chen, Liang-Chun, Hu, Hui-Han, Liu, Jessica, Korenaga, Massaki, et al. A Glycomarker for Short-term Prediction of Hepatocellular Carcinoma: A Longitudinal Study With Serial Measurements. Clinical and translational gastroenterology 2018;9(9):183. | Exclude on population: not cirrhosis |
| Lin, Z Y, Wang, L Y, Yu, M L, Chen, S C, Chuang, W L, Hsieh, M Y, Tsai, J F. Role of serum C-reactive protein as a marker of hepatocellular carcinoma in patients with cirrhosis. Journal of gastroenterology and hepatology 2000;15(4):417-21. | Does not meet full eligibility criteria: pre-2005 |
| Lin, Z. Y., Wang, L. Y., Yu, M. L., Chen, S. C., Chuang, W. L., Hsieh, M. Y., Tsai, J. F. Role of serum C-reactive protein as a marker of hepatocellular carcinoma in patients with cirrhosis. Journal of gastroenterology and hepatology 2000;15(4):417-21. | Does not meet full eligibility criteria: pre-2005 |
| Liou, Wei-Lun, Tan, Si-Yu, Yamada, Hiroyuki, Krishnamoorthy, Thinesh, Chang, Jason Pik-Eu, Yeo, Chin-Pin. Performance of the GALAD Model in an Asian Cohort Undergoing Hepatocellular Carcinoma Surveillance: A Prospective Cohort Study. Journal of gastroenterology and hepatology 2025;40(7):1818â€“1824. | Exclude on population: not cirrhosis |
| Liu, Angela M., Yao, Tzy-Jyun, Wang, Wei, Wong, Kwong-Fai, Lee, Nikki P., Fan, Sheung Tat, et al. Circulating miR-15b and miR-130b in serum as potential markers for detecting hepatocellular carcinoma: a retrospective cohort study. BMJ open 2012;2(2):e000825. | Exclude on population: not cirrhosis |
| Liu, D., Luo, Y., Chen, L., Zuo, D., Li, Y., Wu, J., et al. Diagnostic value of 5 serum biomarkers for hepatocellular carcinoma with different epidemiological backgrounds: A large-scale, retrospective study. Cancer Biology and Medicine 2021;18(1):256-70. | Exclude on population: not cirrhosis |
| Liu, Dandan, Cai, Tianxi, Lok, Anna. Nonparametric Maximum Likelihood Estimators of Time-Dependent Accuracy Measures for Survival Outcome Under Two-Stage Sampling Designs. Journal of the American Statistical Association 2018;113(522):882-92. | Exclude on study design: not a 1- or 2-gate test accuracy study |
| Liu, E. Micronucleus test in diagnosis of primary liver cancer. Chinese Journal of Clinical Oncology 1994;21(4):287-89. | Does not meet full eligibility criteria: pre-2005 |

| Reference | Reason for exclusion |
|---|---|
| Liu, Hong-Dao, Ni, Run-Zhou, Xiao, Ming-Bing, Li, Li-Ren, Hua, Guo-Ping. [Clinical significance of serum GPDA-F determined by immunoelectrophoresis in diagnosis of hepatocellular carcinoma]. Ai zheng = Aizheng = Chinese journal of cancer 2006;25(2):247-49. | Foreign language |
| Liu, Hui, Li, Peng, Zhai, Yun, Qu, Chun-Feng, Zhang, Li-Jie, Tan, Yu-Fen, Li, Ning. Diagnostic value of glypican-3 in serum and liver for primary hepatocellular carcinoma. World journal of gastroenterology 2010;16(35):4410-15. | Does not meet full eligibility criteria: includes people without cirrhosis |
| Liu, Hui, Li, Peng, Zhai, Yun, Qu, Chun-Feng, Zhang, Li-Jie, Tan, Yu-Fen, Li, Ning. Diagnostic value of glypican-3 in serum and liver for primary hepatocellular carcinoma. World journal of gastroenterology 2010;16(35):4410-5. | Does not meet full eligibility criteria: includes people without cirrhosis |
| Liu, Hui-Hui, Fang, Yu, Wang, Jing-Wen, Yuan, Xiao-Dong, Fan, Yu-Chen, Gao, Shuai, Han, Li-Yan. Hypomethylation of the cyclin D1 promoter in hepatitis B virus-associated hepatocellular carcinoma. Medicine 2020;99(20):e20326. | Exclude on population: not cirrhosis |
| Liu, Hui-Hui, Fang, Yu, Wang, Jing-Wen, Yuan, Xiao-Dong, Fan, Yu-Chen, Gao, Shuai, Han, Li-Yan. Hypomethylation of the cyclin D1 promoter in hepatitis B virus-associated hepatocellular carcinoma. Medicine 2020;99(20):e20326. | Duplicate (including Cochrane includes) |
| Liu, Hui-Ying, Qian, Hai-Hua, Zhang, Xiao-Feng, Li, Jun, Yang, Xia, Sun, Bin, et al. Improved method increases sensitivity for circulating hepatocellular carcinoma cells. World journal of gastroenterology 2015;21(10):2918-25. | Exclude on index test |
| Liu, J., Tang, W., Budhu, A., Forgues, M., Hernandez, M. O., Candia, J., et al. A Viral Exposure Signature Defines Early Onset of Hepatocellular Carcinoma. Cell 2020;182(2):317-28.e110. | Exclude on population: not cirrhosis |
| Liu, K., Xu, H., Wen, L., Chen, J. INDIVIDUAL and COMBINED DIAGNOSTIC VALUES of the SERUM TUMOR MARKERS alpha-FETOPROTEIN, THYMIDINE KINASE 1, and MIR-202 for PRIMARY HEPATIC CARCINOMA. Acta Medica Mediterranea 2021;37(1):183-87. | Exclude on population: not cirrhosis |
| Liu, Kehui, Ding, Yezhou, Wang, Yun, Zhao, Qingqing, Yan, Lei, Xie, Jingdong, et al. Combination of IL-34 and AFP improves the diagnostic value during the development of HBV related hepatocellular carcinoma. Clinical and experimental medicine 2022;():n. pag.. | Exclude on population: not cirrhosis |
| Liu, Kehui, Ding, Yezhou, Wang, Yun, Zhao, Qingqing, Yan, Lei, Xie, Jingdong, et al. Combination of IL-34 and AFP improves the diagnostic value during the development of HBV related hepatocellular carcinoma. Clinical and experimental medicine 2023;23(2):397-409. | Exclude on population: not cirrhosis |
| Liu, Li, Li, Yi-Ning, Zhang, Aimin, Yin, Yue, Yue, Zhihong, Pei, Lin, et al. Clinical potential of serum prostaglandin A2 as a novel diagnostic biomarker for hepatocellular cancer. Clinica chimica acta; international journal of clinical chemistry 2024;561():119814. | Does not meet full eligibility criteria: includes people without cirrhosis |
| Liu, M., Liu, X., Ren, P., Li, J., Chai, Y., Zheng, S. J., et al. A cancer-related protein 14-3-3zeta is a potential tumor-associated antigen in immunodiagnosis of hepatocellular carcinoma. Tumor Biology 2014;35(5):4247-56. | Exclude on index test |
| Liu, Mei, Varela-Ramirez, Armando, Li, Jitian, Dai, Liping, Aguilera, Renato J. Humoral autoimmune response to nucleophosmin in the immunodiagnosis of hepatocellular carcinoma. Oncology reports 2015;33(5):2245-52. | Exclude on index test |
| Liu, Miaoxia, Wu, Ruihong, Liu, Xu, Xu, Hongqin, Chi, Xiumei, Wang, Xiaomei, et al. Validation of the GALAD Model and Establishment of GAAP Model for Diagnosis of Hepatocellular Carcinoma in Chinese Patients. Journal of hepatocellular carcinoma 2020;7():219-32. | Does not meet full eligibility criteria: includes people without cirrhosis |
| Liu, Peng, Lu, Di, Al-Ameri, Abdulahad, Wei, Xuyong, Ling, Sunbin, Li, Jie, et al. Glutamine synthetase promotes tumor invasion in hepatocellular carcinoma through mediating epithelial-mesenchymal transition. Hepatology research : the official journal of the Japan Society of Hepatology 2020;50(2):246-57. | Exclude on population: healthy controls |
| Liu, Ruoyu, Li, Yi, Wu, Anqi, Kong, Mingzhu, Ding, Weijia, Hu, Zeyang, et al. Identification of Plasma hsa_circ_0005397 and Combined With Serum AFP, AFP-L3 as Potential Biomarkers for Hepatocellular Carcinoma. Frontiers in pharmacology 2021;12():639963. | Exclude on population: not cirrhosis |

| Reference | Reason for exclusion |
|---|---|
| Liu, Taotao, Xue, Ruyi, Dong, Ling, Wu, Hao, Zhang, Danying. Rapid determination of serological cytokine biomarkers for hepatitis B virus-related hepatocellular carcinoma using antibody microarrays. Acta biochimica et biophysica Sinica 2011;43(1):45-51. | Exclude on population: not cirrhosis |
| Liu, Taotao, Xue, Ruyi, Huang, Xiaowu, Zhang, Danying, Dong, Ling, Wu, Hao. Proteomic profiling of hepatitis B virus-related hepatocellular carcinoma with magnetic bead-based matrix-assisted laser desorption/ionization time-of-flight mass spectrometry. Acta biochimica et biophysica Sinica 2011;43(7):542-50. | Exclude on population: not cirrhosis |
| Liu, Tianhui, Yao, Mingjie, Liu, Shuhong, Wang, Lu, Wang, Leijie, Hou, Jinlin, et al. Serum Golgi protein 73 is not a suitable diagnostic marker for hepatocellular carcinoma. Oncotarget 2017;8(10):16498-50606. | No 2x2 data |
| Liu, Weifeng, Hu, Jie, Zhou, Kaiqian, Chen, Feiyu, Wang, Zheng, Liao, Boyi, et al. Serum exosomal miR-125b is a novel prognostic marker for hepatocellular carcinoma. OncoTargets and therapy 2017;10():3843-51. | Exclude on study design: not a 1- or 2-gate test accuracy study |
| Liu, X Y, Fan, Y C, Gao, S, Zhao, J, Chen, L Y, Li, F. Methylation of SOX1 and VIM promoters in serum as potential biomarkers for hepatocellular carcinoma. Neoplasma 2017;64(5):745-53. | Exclude on population: not cirrhosis |
| Liu, X. E., Desmyter, L., Gao, C. F., Laroy, W., Dewaele, S., Vanhooren, V., et al. N-glycomic changes in hepatocellular carcinoma patients with liver cirrhosis induced by hepatitis B virus. Hepatology 2007;46(5):1426-35. | Duplicate (including Cochrane includes) |
| Liu, X. F., Thin, K. Z., Ming, X. L., Shuo, Li, Ping, Luo, Man, Zhu, Li, N. D. Small nucleolar RNA host gene 18 acts as a tumor suppressor and a diagnostic indicator in hepatocellular carcinoma. Technology in Cancer Research and Treatment 2018;17():1-8. | Duplicate (including Cochrane includes) |
| Liu, X. Y., Fan, Y. C., Gao, S., Zhao, J., Chen, L. Y., Li, F. Methylation of SOX1 and VIM promoters in serum as potential biomarkers for hepatocellular carcinoma. Neoplasma 2017;64(5):745-53. | Duplicate (including Cochrane includes) |
| Liu, X., Fu, B., Chen, J., Sun, Z., Zheng, D., Li, Z., et al. High-throughput intact Glycopeptide quantification strategy with targeted-MS (HTiGQs-target) reveals site-specific IgG N-glycopeptides as biomarkers for hepatic disorder diagnosis and staging. Carbohydrate polymers 2024;325():121499. | No 2x2 data |
| Liu, X., Gong, Q., Gao, L., Niu, Y., Chi, X., Cheng, M., et al. Association of serum level of growth differentiation factor 15 with liver cirrhosis and hepatocellular carcinoma. PLoS ONE 2015;10(5):e0127518. | No 2x2 data |
| Liu, X., Lina, M., Xia, L., Tang, Y., Liu, S. Diagnostic value of liver stiffness measurement combined with serum high-sensitivity C-reactive protein detection in HBV-related cirrhosis patients complicated with primary liver cancer. Chinese Journal of Oncology 2015;37(2):119-22. | Foreign language |
| Liu, Xiao-Yan, Ma, Li-Na, Yan, Ting-Ting, Lu, Zhen-Hui, Tang, Yuan-Yuan, Luo, Xia. Combined detection of liver stiffness and C-reactive protein in patients with hepatitis B virus-related liver cirrhosis, with and without hepatocellular carcinoma. Molecular and clinical oncology 2016;4(4):587-90. | Exclude on index test |
| Liu, Xijiao, Zou, Li, Liu, Fei, Zhou, Yin. Gadoxetic acid disodium-enhanced magnetic resonance imaging for the detection of hepatocellular carcinoma: a meta-analysis. PloS one 2013;8(8):e70896. | Exclude on study design: not a 1- or 2-gate test accuracy study |
| Liu, Xu, Meng, Jing, Xu, Hongqin. Alpha-fetoprotein to transaminase ratio is related to higher diagnostic efficacy for hepatocellular carcinoma. Medicine 2019;98(17):e15414. | Does not meet full eligibility criteria: includes people without cirrhosis |
| Liu, Xu, Meng, Jing, Xu, Hongqin. Alpha-fetoprotein to transaminase ratio is related to higher diagnostic efficacy for hepatocellular carcinoma. Medicine 2019;98(17):e15414. | Duplicate (including Cochrane includes) |
| Liu, Xue-Fang, Thin, Khaing Zar, Ming, Xin-Liang, Shuo-Li, Ping-Luo, Man-Zhu, Li, Nan-Di. Small Nucleolar RNA Host Gene 18 Acts as a Tumor Suppressor and a Diagnostic Indicator in Hepatocellular Carcinoma. Technology in cancer research & treatment 2018;17(101140941):1533033818794494. | Does not meet full eligibility criteria: includes people without cirrhosis |
| Liu, Y., He, J., Li, C., Benitez, R., Fu, S., Marrero, J. Identification and confirmation of biomarkers using an integrated platform for quantitative analysis of glycoproteins and their glycosylations. Journal of Proteome Research 2010;9(2):798-805. | Does not meet full eligibility criteria: includes people without cirrhosis |

| Reference | Reason for exclusion |
|---|---|
| Liu, Y., Jiang, W., Li, X., Zhao, H. The Diagnostic Performance of AFP, AFP-L3, DCP, CA199, and Their Combination for Primary Liver Cancer. Journal of Hepatocellular Carcinoma 2025;12():513 " 526. | Does not meet full eligibility criteria: includes people without cirrhosis |
| Liu, Y., Peng, F., Wang, S., Jiao, H., Zhou, K., Guo, W., et al. Aberrant fragmentomic features of circulating cell-free mitochondrial DNA enable early detection and prognosis prediction of hepatocellular carcinoma. Clinical and Molecular Hepatology 2025;31(1):196 " 212. | Exclude on population: not cirrhosis |
| Liu, Y., Sogawa, K., Sunaga, M., Umemura, H., Satoh, M., Kazami, T., et al. Increased concentrations of apo A-I and apo A-II fragments in the serum of patients with hepatocellular carcinoma by magnetic beads-assisted MALDI-TOF mass spectrometry. American Journal of Clinical Pathology 2014;141(1):52-61. | Does not meet full eligibility criteria: includes people without cirrhosis |
| Liu, Y., Wu, M. C., Qian, G. X. Detection of circulating hepatocellular carcinoma cells in peripheral venous blood by reverse transcription-polymerase chain reaction. Hepatobiliary and Pancreatic Diseases International 2002;1(1):72-76. | Does not meet full eligibility criteria: pre-2005 |
| Liu, Yang, Wang, Yue-ru, Wang, Long, Song, Rui-mei, Zhou, Bo. Significance of detecting circulating hepatocellular carcinoma cells in peripheral blood of hepatocellular carcinoma patients by nested reverse transcription-polymerase chain reaction and its clinical value: a retrospective study. Tumori 2014;100(5):536-40. | Does not meet full eligibility criteria: includes people without cirrhosis |
| Liu, Yi, Jin, Jia, Ji, Jian, Gao, Xi-Mei. Tumor necrosis factor-alpha-induced protein 8-like 2 mRNA in peripheral blood mononuclear cells is associated with the disease progression of chronic hepatitis B virus infection. Virology journal 2019;16(1):120. | Exclude on population: not cirrhosis |
| Liu, Yue, Hong, Zhanying, Tan, Guangguo, Dong, Xin, Yang, Genjin, Zhao, Liang, et al. NMR and LC/MS-based global metabolomics to identify serum biomarkers differentiating hepatocellular carcinoma from liver cirrhosis. International journal of cancer 2014;135(3):658-68. | No 2x2 data |
| Liu, Zhaobo, Wu, Min, Lin, Dongdong. Des-gamma-carboxyprothrombin is a favorable biomarker for the early diagnosis of alfa-fetoprotein-negative hepatitis B virus-related hepatocellular carcinoma. The Journal of international medical research 2020;48(2):300060520902575. | Does not meet full eligibility criteria: includes people without cirrhosis |
| Liu, Zhikun, Li, Jie, Chen, Jun, Shan, Qiaonan, Dai, Haojiang, Xie, Haiyang, et al. MCM family in HCC: MCM6 indicates adverse tumor features and poor outcomes and promotes S/G2 cell cycle progression. BMC cancer 2018;18(1):200. | Exclude on population: tissue samples |
| Liu, Zhiying, Liu, Hongtao, Chen, Zhiji, Deng, Chao, Zhou, Li, Chen, Siyuan, et al. Identification of a novel plasma metabolite panel as diagnostic biomarker for hepatocellular carcinoma. Clinica chimica acta; international journal of clinical chemistry 2023;543():117302. | Does not meet full eligibility criteria: includes people without cirrhosis |
| Liu, Zixin, Guo, Weixing, Zhang, Dandan, Pang, Yanan, Shi, Jie, Wan, Siqin, et al. Circulating tumor cell detection in hepatocellular carcinoma based on karyoplasmic ratios using imaging flow cytometry. Scientific reports 2016;6():39808. | Exclude on population: not cirrhosis |
| Llovet, Josep M., Chen, Yingbei, Wurmbach, Elisa, Roayaie, Sasan, Fiel, M. Isabel, Schwartz, Myron, et al. A molecular signature to discriminate dysplastic nodules from early hepatocellular carcinoma in HCV cirrhosis. Gastroenterology 2006;131(6):1758-67. | Exclude on population: clinical signs and symptoms of HCC |
| Loglio A., Iavarone M., Facchetti F., Di Paolo D., Perbellini R., Lunghi G., et al. The combination of pivka-II and AFP levels improves the diagnostic accuracy of HCC diagnosis in long-term nuc suppressed HBV caucasian cirrhotics. Hepatology 2018;68(Supplement 1):517A. | CONFERENCE ABSTRACT |
| Loglio, A., Iavarone, M., Vigano, M., Orenti, A., Facchetti, F., Cortinovis, I., et al. Minimal increases of serum alpha-foetoprotein herald HCC detection in Caucasian HBV cirrhotic patients under long-term oral therapy. Liver International 2019;39(10):1964-74. | Duplicate (including Cochrane includes) |
| Lok, A. S. alpha-Fetoprotein monitoring in Chinese patients with chronic hepatitis B virus infection: role in the early detection of hepatocellular carcinoma. Hepatology (Baltimore, Md.) 1989;9(1):110-15. | Does not meet full eligibility criteria: pre-2005 |

| Reference | Reason for exclusion |
|---|---|
| Lok, A. S. F. alpha-Fetoprotein monitoring in Chinese patients with chronic hepatitis B virus infection: Role in the early detection of hepatocellular carcinoma. Hepatology 1989;9(1):110-15. | Exclude on population: not cirrhosis |
| Lok, Anna S, Sterling, Richard K, Everhart, James E, Wright, Elizabeth C, Hoefs, John C, Di Bisceglie, Adrian M, et al. Des-gamma-carboxy prothrombin and alpha-fetoprotein as biomarkers for the early detection of hepatocellular carcinoma. Gastroenterology 2010;138(2):493-502. | Does not meet full eligibility criteria: includes people without cirrhosis |
| Lok, Anna S., Sterling, Richard K., Everhart, James E., Wright, Elizabeth C., Hoefs, John C., Di Bisceglie, Adrian M., et al. Des-gamma-carboxy prothrombin and alpha-fetoprotein as biomarkers for the early detection of hepatocellular carcinoma. Gastroenterology 2010;138(2):493-502. | Duplicate (including Cochrane includes) |
| Long, Jiang, Lang, Zhen-Wei, Wang, Hua-Guang, Wang, Tai-Ling, Wang, Bao-En. Glutamine synthetase as an early marker for hepatocellular carcinoma based on proteomic analysis of resected small hepatocellular carcinomas. Hepatobiliary & pancreatic diseases international : HBPD INT 2010;9(3):296-305. | Does not meet full eligibility criteria: includes people without cirrhosis |
| Long, Jiang, Wang, Huaguang, Lang, Zhenwei, Wang, Tailing, Long, Mei. Expression level of glutamine synthetase is increased in hepatocellular carcinoma and liver tissue with cirrhosis and chronic hepatitis B. Hepatology international 2011;5(2):698-706. | Does not meet full eligibility criteria: includes people without cirrhosis |
| Long, Xiao-Ran, Zhang, Yao-Jun, Zhang, Mei-Yin, Chen, Keng, Zheng, X. F. Steven. Identification of an 88-microRNA signature in whole blood for diagnosis of hepatocellular carcinoma and other chronic liver diseases. Aging 2017;9(6):1565-84. | Does not meet full eligibility criteria: includes people without cirrhosis |
| Lopez, J. B. & Balasegaram, M. Serum CA 125 as a marker of hepatocellular carcinoma. The International journal of biological markers 1996;11(3):178-82. | Exclude on population: not cirrhosis |
| Lou, Zhong-Han, Xu, Ke-Yang, Qiao, Liang, Su, Xiao-Qian, Ou-Yang, Yuan, Miao, Liang-Bin, et al. Diagnostic Potential of the Serum lncRNAs HOTAIR, BRM and ICR for Hepatocellular Carcinoma. Frontiers in bioscience (Landmark edition) 2022;27(9):264. | Does not meet full eligibility criteria: includes people without cirrhosis |
| Lu, C. Y., Lin, K. Y., Tien, M. T., Wu, C. T., Uen, Y. H. Frequent DNA methylation of MiR-129-2 and its potential clinical implication in hepatocellular carcinoma. Genes Chromosomes and Cancer 2013;52(7):636-43. | Does not meet full eligibility criteria: includes people without cirrhosis |
| Lu, C. Y., Tsai, K. F., Yen, C. J., Hsieh, C. J., Peng, P. J., Huang, S. C., Chuang, M. R. Circulating tumor DNA methylation markers for the early diagnosis of hepatocellular carcinoma. Clinical and Experimental Medicine 2025;25(1):83. | Does not meet full eligibility criteria: includes people without cirrhosis |
| Lu, Chang-Yi, Chen, Shih-Ya, Peng, Hui-Ling, Kan, Pu-Yeh, Chang, Wan-Chi. Cell-free methylation markers with diagnostic and prognostic potential in hepatocellular carcinoma. Oncotarget 2017;8(4):6406-18. | Exclude on population: healthy controls |
| Lu, Chang-Yi, Lin, Kai-Yuan, Tien, Meng-Tsung, Wu, Cheng-Tao, Uen, Yih-Huei. Frequent DNA methylation of MiR-129-2 and its potential clinical implication in hepatocellular carcinoma. Genes, chromosomes & cancer 2013;52(7):636-43. | Does not meet full eligibility criteria: includes people without cirrhosis |
| Lu, Chang-Yi, Tsai, Kun-Feng, Yen, Chia-Jui, Hsieh, Chueh-Jung, Peng, Pey-Jey, Huang, Shao-Chang, Chuang, Meng-Rong. Circulating tumor DNA methylation markers for the early diagnosis of hepatocellular carcinoma. Clinical and experimental medicine 2025;25(1):83. | Does not meet full eligibility criteria: includes people without cirrhosis |
| Lu, D., Bai, X., Zou, Q., Gan, Z. Identification of the association between HMMR expression and progression of hepatocellular carcinoma via construction of a co-expression network. Oncology Letters 2020;20(3):2645-54. | Exclude on population: tissue samples |
| Lu, F., Chen, H., Gao, M. Clinical value of des-gamma-carboxy prothrombin in the diagnosis of hepatocellular carcinoma. Chinese Journal of Clinical Oncology 2009;36(7):361-64. | Foreign language |
| Lu, F., Ott, C., Bista, P. Three-Dimensional Structure of Novel Liver Cancer Biomarker Liver Cancer-Specific Serine Protease Inhibitor Kazal (LC-SPIK) and Its Performance in Clinical Diagnosis of Hepatocellular Carcinoma (HCC). Diagnostics 2024;14(7):725. | Secondary publication of included study |

| Reference | Reason for exclusion |
|---|---|
| Lu, Jiongjiong, Xie, Feng, Geng, Li, Shen, Weifeng, Sui, Chengjun. Investigation of serum lncRNA-uc003wbd and lncRNA-AF085935 expression profile in patients with hepatocellular carcinoma and HBV. Tumour biology : the journal of the International Society for Oncodevelopmental Biology and Medicine 2015;36(5):3231-36. | Exclude on population: not cirrhosis |
| Lu, L., Zhu, C., Yi, L., Li, S., Fan, M., Lin, J. The diagnostic value of midkine as a novel serum biomarker in alpha-fetoprotein-negative hepatocellular carcinoma. International Journal of Biological Markers 2025;():03936155251358265. | Does not meet full eligibility criteria: includes people without cirrhosis |
| Lu, Shi-Xun, Huang, Yu-Hua, Liu, Li-Li, Zhang, Chris Zhiyi, Yang, Xia, Yang, Yuan-Zhong, et al. alpha-Fetoprotein mRNA in situ hybridisation is a highly specific marker of hepatocellular carcinoma: a multi-centre study. British journal of cancer 2021;124(12):1988-96. | Exclude on population: clinical signs and symptoms of HCC |
| Lu, X., Nie, H., Zhan, C., Liu, X., Shi, X., Shi, M., Zhang, Y. Comprehensive characterization and evaluation of hepatocellular carcinoma by LC-MS based serum metabolomics. Metabolomics 2015;11(5):1381-93. | Exclude on population: not cirrhosis |
| Lu, Y., Huang, C., Gao, L., Xu, Y. J., Chia, S. E., Chen, S., et al. Identification of serum biomarkers associated with hepatitis B virus-related hepatocellular carcinoma and liver cirrhosis using mass-spectrometry-based metabolomics. Metabolomics 2015;11(6):1526-38. | Exclude on population: healthy controls |
| Lu, Yiyu, Fang, Zhaoyuan, Li, Meiyi, Chen, Qian, Zeng, Tao, Lu, Lina, et al. Dynamic edge-based biomarker non-invasively predicts hepatocellular carcinoma with hepatitis B virus infection for individual patients based on blood testing. Journal of molecular cell biology 2019;11(8):665-77. | Does not meet full eligibility criteria: includes people without cirrhosis |
| Lu, Yonghai, Fang, Jinling, Zou, Li, Cui, Liang, Liang, Xu, Lim, Seng Gee, Dan, Yock-Young. Omega-6-derived oxylipin changes in serum of patients with hepatitis B virus-related liver diseases. Metabolomics : Official journal of the Metabolomic Society 2018;14(3):26. | Does not meet full eligibility criteria: includes people without cirrhosis |
| Lu, Yonghai, Li, Ning, Gao, Liang, Xu, Yong-Jiang, Huang, Chong, Yu, Kangkang, et al. Acetylcarnitine Is a Candidate Diagnostic and Prognostic Biomarker of Hepatocellular Carcinoma. Cancer research 2016;76(10):2912-20. | Exclude on index test |
| Lu, Yunjie, Duan, Yunfei, Xu, Qinghua, Zhang, Li, Chen, Weibo, Qu, Zhen, et al. Circulating exosome-derived bona fide long non-coding RNAs predicting the occurrence and metastasis of hepatocellular carcinoma. Journal of cellular and molecular medicine 2020;24(2):1311-18. | Exclude on population: not cirrhosis |
| Luca, Angelo, Caruso, Settimo, Milazzo, Mariapina, Mamone, Giuseppe, Marrone, Gianluca, Miraglia, Roberto, et al. Multidetector-row computed tomography (MDCT) for the diagnosis of hepatocellular carcinoma in cirrhotic candidates for liver transplantation: prevalence of radiological vascular patterns and histological correlation with liver explants. European radiology 2010;20(4):898-907. | Exclude on population: clinical signs and symptoms of HCC |
| Lucatelli, Pierleone, Argiro, Renato, Ginanni Corradini, Stefano, Saba, Luca, Cirelli, Carlo, Fanelli, Fabrizio, et al. Comparison of Image Quality and Diagnostic Performance of Cone-Beam CT during Drug-Eluting Embolic Transarterial Chemoembolization and Multidetector CT in the Detection of Hepatocellular Carcinoma. Journal of vascular and interventional radiology : JVIR 2017;28(7):978-86. | Exclude on population: clinical signs and symptoms of HCC |
| Ludwig, Daniel R., Fraum, Tyler J., Cannella, Roberto, Ballard, David H., Tsai, Richard, Naeem, Muhammad, et al. Hepatocellular carcinoma (HCC) versus non-HCC: accuracy and reliability of Liver Imaging Reporting and Data System v2018. Abdominal radiology (New York) 2019;44(6):2116-32. | Exclude on population: clinical signs and symptoms of HCC |
| Lui, Ka Yin, Peng, Hao-Ran, Lin, Jin-Rong, Qiu, Chun-Hui, Chen, Hu-An, Fu, Rong-Dang, Cai, Chang-Jie. Pseudogene integrator complex subunit 6 pseudogene 1 (INTS6P1) as a novel plasma-based biomarker for hepatocellular carcinoma screening. Tumour biology : the journal of the International Society for Oncodevelopmental Biology and Medicine 2016;37(1):1253-60. | Exclude on population: not cirrhosis |

| Reference | Reason for exclusion |
| --- | --- |
| Luo, Biyuan, Ma, Fang, Liu, Hao, Hu, Jixiong, Rao, Le, Liu, Chun, et al. Cell-free DNA methylation markers for differential diagnosis of hepatocellular carcinoma. BMC medicine 2022;20(1):8. | Does not meet full eligibility criteria: includes people without cirrhosis |
| Luo, K. & Liu, Z. Effect of antiviral treatment on alfa-fetoprotein levels in HBV-related cirrhotic patients: early detection of hepatocellular carcinoma. Journal of viral hepatitis 2010;17(7):511-17. | Exclude on population: clinical signs and symptoms of HCC |
| Luo, L., Wang, X., Peng, X., Zhong, R., Xuan, X., Lin, H., Lin, X. Analysis of the optimal patterns of serum alpha fetoprotein (AFP), AFP-L3% and protein induced by vitamin K absence or antagonist-II (PIVKA-II) detection in the diagnosis of liver cancers. PeerJ 2025;13():e19712. | Exclude on population: not cirrhosis |
| Luo, Ping, Feng, Xiaobo, Jing, Wei, Zhu, Man, Li, Nandi, Zhou, Hu, et al. Clinical and Diagnostic Significance of Homer1 in hepatitis B virus-induced Hepatocellular Carcinoma. Journal of Cancer 2018;9(4):683-89. | Does not meet full eligibility criteria: includes people without cirrhosis |
| Luo, Ping, Liang, Chunzi, Jing, Wei, Zhu, Man, Zhou, Hu, Chai, Hongyan, Worley, Paul F. Homer2 and Homer3 Act as Novel Biomarkers in Diagnosis of hepatitis B virus-induced Hepatocellular Carcinoma. Journal of Cancer 2021;12(12):3439-47. | Exclude on index test |
| Luo, Ping, Liang, Chunzi, Zhang, Xianwei, Liu, Xuefang, Wang, Yingchao, Wu, Mengmeng, Feng, Xiaobo. Identification of long non-coding RNA ZFAS1 as a novel biomarker for diagnosis of HCC. Bioscience reports 2018;38(4):n. pag.. | Exclude on population: healthy controls |
| Luo, Ping, Yin, Peiyuan, Hua, Rui, Tan, Yexiong, Li, Zaifang, Qiu, Gaokun, et al. A Large-scale, multicenter serum metabolite biomarker identification study for the early detection of hepatocellular carcinoma. Hepatology (Baltimore, Md.) 2018;67(2):662-75. | Duplicate (including Cochrane includes) |
| Luo, Ping, Yin, Peiyuan, Hua, Rui, Tan, Yexiong, Li, Zaifang, Qiu, Gaokun, et al. A Large-scale, multicenter serum metabolite biomarker identification study for the early detection of hepatocellular carcinoma. Hepatology (Baltimore, Md.) 2018;67(2):662-75. | Does not meet full eligibility criteria: includes people without cirrhosis |
| Luo, Qin, Zhang, Yurong, Wang, Ning, Jin, Guangzhi, Jin, Haojie, Gu, Dishui, et al. Leukemia inhibitory factor receptor is a novel immunomarker in distinction of well-differentiated HCC from dysplastic nodules. Oncotarget 2015;6(9):6989-99. | Exclude on population: clinical signs and symptoms of HCC |
| Lv, F., Gao, Y. F., Rao, J. G., Zhang, W., Zou, G. Z. Clinical characteristics of patients with hepatitis B virus related liver cirrhosis and primary liver cancer. World Chinese Journal of Digestology 2015;23(17):2798-8004. | Foreign language |
| Lv, Y., Wang, W., Jia, W. D., Sun, Q. K., Huang, M., Zhou, H. C., et al. High preoparative levels of serum periostin are associated with poor prognosis in patients with hepatocellular carcinoma after hepatectomy. European journal of surgical oncology : the journal of the European Society of Surgical Oncology and the British Association of Surgical Oncology 2013;39(10):1129-35. | No 2x2 data |
| Lv, Zhihua, Tao, Yu, Cai, Xuan, Zhou, Xin. Cluster of specified microRNAs in tissues and serum as biomarkers for early diagnosis of hepatocellular carcinoma. International journal of clinical and experimental pathology 2018;11(2):990-97. | Exclude on population: healthy controls |
| Lyu, Lihua, Yang, Wenjing, Yao, Jiayi, Wang, Hao, Zhu, Jie, Jin, Anli, et al. The diagnostic value of plasma exosomal hsa_circ_0070396 for hepatocellular carcinoma. Biomarkers in medicine 2021;15(5):359-71. | Does not meet full eligibility criteria: includes people without cirrhosis |
| Ma, Jiaxiu, Jin, Jiapei, Lu, Huishuang, Zhang, Jin, Li, Yalan. Exonuclease 1 is a Potential Diagnostic and Prognostic Biomarker in Hepatocellular Carcinoma. Frontiers in molecular biosciences 2022;9():889414. | Exclude on population: not cirrhosis |
| Ma, Lifang, Lin, Jiafei, Qiao, Yongxia, Weng, Wenhao, Liu, Weiwei, Wang, Jiayi. Serum CD166: a novel hepatocellular carcinoma tumor marker. Clinica chimica acta; international journal of clinical chemistry 2015;441():156-62. | Exclude on population: healthy controls |
| Ma, Q. Q., Huang, J. T., Tong, H. B. Glypican-3, a novel tumor marker in diagnosis of human hepatocellular carcinoma. Chinese Journal of Cancer Prevention and Treatment 2014;21(2):127-32. | Foreign language |
| Ma, Xiang, Wang, Xin, Yang, Chao, Wang, Zusen, Han, Bing, Wu, Liqun. DANCR Acts as a Diagnostic Biomarker and Promotes Tumor Growth and | Exclude on population: not cirrhosis |

| Reference | Reason for exclusion |
| --- | --- |
| Metastasis in Hepatocellular Carcinoma. Anticancer research 2016;36(12):6389-98. | |
| Ma, Xiang, Yuan, Tingdong, Yang, Chao, Wang, Zusen, Zang, Yunjin, Wu, Liqun. X-inactive-specific transcript of peripheral blood cells is regulated by exosomal Jpx and acts as a biomarker for female patients with hepatocellular carcinoma. Therapeutic advances in medical oncology 2017;9(11):665-77. | Exclude on population: healthy controls |
| Ma, Xiao-Lu, Jiang, Mi, Zhao, Ying, Wang, Bei-Li, Shen, Min-Na, Zhou, Yan, et al. Application of Serum Annexin A3 in Diagnosis, Outcome Prediction and Therapeutic Response Evaluation for Patients with Hepatocellular Carcinoma. Annals of surgical oncology 2018;25(6):1686-94. | Duplicate (including Cochrane includes) |
| Ma, Xiao-Lu, Jiang, Mi, Zhao, Ying, Wang, Bei-Li, Shen, Min-Na, Zhou, Yan, et al. Application of Serum Annexin A3 in Diagnosis, Outcome Prediction and Therapeutic Response Evaluation for Patients with Hepatocellular Carcinoma. Annals of surgical oncology 2018;25(6):1686-94. | Exclude on population: not cirrhosis |
| Macarini, L., Marini, S., Milillo, P., Vinci, R. Double-contrast MRI (DC-MRI) in the study of the cirrhotic liver: utility of administering Gd-DTPA as a complement to examinations in which SPIO liver uptake and distribution alterations (SPIO-LUDA) are present and in the identification and characterisation of focal lesions. La Radiologia medica 2006;111(8):1087-1002. | No 2x2 data |
| Macarini, L., Milillo, P., Cascavilla, A., Scalzo, G., Stoppino, L., Vinci, R., Moretti, G. MR characterisation of dysplastic nodules and hepatocarcinoma in the cirrhotic liver with hepatospecific superparamagnetic contrast agents: pathological correlation in explanted livers. La Radiologia medica 2009;114(8):1267-82. | Exclude on population: clinical signs and symptoms of HCC |
| Machida, M., Ambo, M., Mishina, R., Hada, N., Tachibana, F., Yamashita, M., Konda, A. Integrated Predictors by Propensity Scoring With Tumor Markers and Plasma Levels of microRNA-21-5p, IL-17, and IL-10 Complement Early Detection of Hepatocellular Carcinoma in Patients With Liver Cirrhosis. Technology in Cancer Research and Treatment 2023;22():n. pag.. | Exclude on population: not cirrhosis |
| Maciel, Antonio Carlos, Cerski, Carlos Thadeu, Moreira, Roger Klein, Resende, Vinicius Labrea, Zanotelli, Maria Lucia. Hepatocellular carcinoma in patients undergoing orthotopic liver transplantation: radiological findings with anatomopathological correlation in Brazil. Arquivos de gastroenterologia 2006;43(1):24-29. | No 2x2 data |
| Madkour, B. A., Sabry, O. M., Wahdan, M. S., Abdelaziz, A. H., Rahim, A. A., Morsy, S. A. An update of hepatic biomarkers in Hepatocellular Carcinoma among HCV patients in Egypt. Pakistan Journal of Medical and Health Sciences 2021;15(4):940-46. | Exclude on population: not cirrhosis |
| Maeda, Takahiro, Kanzaki, Hiroaki, Chiba, Tetsuhiro, Ao, Junjie, Kanayama, Kengo, Maruta, Susumu, et al. Serum fibroblast growth factor 19 serves as a potential novel biomarker for hepatocellular carcinoma. BMC cancer 2019;19(1):1088. | Does not meet full eligibility criteria: includes people without cirrhosis |
| Mahalingam, Devalingam, Chelis, Leonidas, Nizamuddin, Imran, Lee, Sunyoung S., Kakolyris, Stylianos, Halff, Glenn, et al. Detection of Hepatocellular Carcinoma in a High-Risk Population by a Mass Spectrometry-Based Test. Cancers 2021;13(13):n. pag.. | Does not meet full eligibility criteria: includes people without cirrhosis |
| Maimaitiming, Abuduaiheti, Zhou, Xing, Ma, Xianxiong, Huang, Yongming, Wang, Qingbo, Deng, Rui, et al. Clinicopathological and Prognostic Value of Plasma CD24 Level in Hepatocellular Carcinoma. Journal of investigative surgery : the official journal of the Academy of Surgical Research 2020;33(6):536-41. | Exclude on study design: not a 1- or 2-gate test accuracy study |
| Maiwald, Bettina, Lobsien, Donald, Kahn, Thomas. Is 3-Tesla Gd-EOB-DTPA-enhanced MRI with diffusion-weighted imaging superior to 64-slice contrast-enhanced CT for the diagnosis of hepatocellular carcinoma? PloS one 2014;9(11):e111935. | Exclude on population: clinical signs and symptoms of HCC |
| Makita, O., Yamashita, Y., Arakawa, A., Nakayama, Y., Mitsuzaki, K., Ando, M., et al. Diffuse perfusion abnormality of the liver parenchyma on angiography-assisted helical CT in relation to cirrhosis and previous treatments: a potential diagnostic pitfall for detecting hepatocellular carcinoma. Clinical imaging 2000;24(5):292-97. | Exclude on population: clinical signs and symptoms of HCC |

| Reference | Reason for exclusion |
|---|---|
| Malaguarnera, Michele, Vacante, Marco, Fichera, Rosangela, Cappellani, Alessandro, Cristaldi, Erika. Chromogranin A (CgA) serum level as a marker of progression in hepatocellular carcinoma (HCC) of elderly patients. Archives of gerontology and geriatrics 2010;51(1):81-85. | No 2x2 data |
| Malik, J., Klammer, M., Rolny, V., Chan, H. L. Y., Piratvisuth, T., Tanwandee, T., et al. Comprehensive evaluation of microRNA as a biomarker for the diagnosis of hepatocellular carcinoma. World Journal of Gastroenterology 2022;28(29):3917-33. | Does not meet full eligibility criteria: includes people without cirrhosis |
| Malik, Juliane, Klammer, Martin, Rolny, Vinzent, Chan, Henry Lik-Yuen, Piratvisuth, Teerha, Tanwandee, Tawesak, et al. Comprehensive evaluation of microRNA as a biomarker for the diagnosis of hepatocellular carcinoma. World journal of gastroenterology 2022;28(29):3917-33. | Does not meet full eligibility criteria: includes people without cirrhosis |
| Malov, S. I., Malov, I. V., Dvornichenko, V. V., Rasulov, R. I., Kuvshinov, A. G., Marche, P. N., et al. [Application of alpha-fetoprotein and osteopontin combination for early diagnosis of hepatocellular carcinoma associated with hepatitis C.]. Klinicheskaia laboratornaia diagnostika 2019;64(10):607-12. | Foreign language |
| Malov, S. I., Yushchuk, N. D., Malov, I. V., Dvornichenko, V. V., Rasulov, R. I., Marche, P. N., et al. A study of serum miRNA-122 in hepatitis C and associated hepatocellular carcinoma. Vestnik Rossiiskoi Akademii Meditsinskikh Nauk 2019;74(6):388-95. | Foreign language |
| Mamdouh, Fatma, Abdel Alem, Shereen, Abdo, Mahmoud, Abdelaal, Amaal, Salem, Amel, Rabiee, Ahmed. Serum Serotonin as a Potential Diagnostic Marker for Hepatocellular Carcinoma. Journal of interferon & cytokine research : the official journal of the International Society for Interferon and Cytokine Research 2019;39(12):780-85. | Exclude on index test |
| Mamdouh, Samah, Khorshed, Fatma, Aboushousha, Tarek, Hamdy, Hussam, Diab, Ayman, Seleem, Mohamed. Evaluation of Mir-224, Mir-215 and Mir-143 as Serum Biomarkers for HCV Associated Hepatocellular Carcinoma. Asian Pacific journal of cancer prevention : APJCP 2017;18(11):3167-71. | Exclude on population: healthy controls |
| Manganelli, Michele, Grossi, Ilaria, Corsi, Jessica, D'Agostino, Vito Giuseppe, Jurikova, Katarina, Cusanelli, Emilio, et al. Expression of Cellular and Extracellular TERRA, TERC and TERT in Hepatocellular Carcinoma. International journal of molecular sciences 2022;23(11):n. pag.. | Exclude on population: healthy controls |
| Mansour, Lamiaa A., El Raziky, Maissa, Mohamed, Amal A., Mahmoud, Enas H., Hamdy, Sherif. Circulating Hypermethylated RASSF1A as a Molecular Biomarker for Diagnosis of Hepatocellular Carcinoma. Asian Pacific journal of cancer prevention : APJCP 2017;18(6):1637-43. | Does not meet full eligibility criteria: includes people without cirrhosis |
| Mantovani, G. Circulating interleukin-6 as a tumor marker for hepatocellular carcinoma. Annals of Oncology 2008;19(7):1355. | Exclude on study design: not a 1- or 2-gate test accuracy study |
| Mao, Liping, Wang, Yueguo, Wang, Delin, Han, Gang, Fu, Shouzhong. TEMs but not DKK1 could serve as complementary biomarkers for AFP in diagnosing AFP-negative hepatocellular carcinoma. PloS one 2017;12(9):e0183880. | Duplicate (including Cochrane includes) |
| Mao, Liping, Wang, Yueguo, Wang, Delin, Han, Gang, Fu, Shouzhong. TEMs but not DKK1 could serve as complementary biomarkers for AFP in diagnosing AFP-negative hepatocellular carcinoma. PloS one 2017;12(9):e0183880. | Does not meet full eligibility criteria: includes people without cirrhosis |
| Mao, Y. L., Yang, H. Y., Xu, H. F., Sang, X. T., Lu, X., Yang, Z. Y., et al. Significance of Golgi glycoprotein 73, a new tumor marker in diagnosis of hepatocellular carcinoma: A primary study. National Medical Journal of China 2008;88(14):948-51. | Foreign language |
| Mao, Yilei, Yang, Huayu, Xu, Haifeng, Lu, Xin, Sang, Xinting, Du, Shunda, et al. Golgi protein 73 (GOLPH2) is a valuable serum marker for hepatocellular carcinoma. Gut 2010;59(12):1687-93. | Exclude on population: healthy controls |
| Marchiano, A., Spreafico, C., Lanocita, R., Frigerio, L., Di Tolla, G., Patelli, G., et al. Does iodine concentration affect the diagnostic efficacy of biphasic spiral CT in patients with hepatocellular carcinoma? Abdominal imaging 2005;30(3):274-80. | Exclude on population: clinical signs and symptoms of HCC |
| Marchio, A., Dhifallah, I., Bahri, O. Circulating aflatoxin B1-related TP53 mutation detected by digital PCR in Tunisian patients with and without hepatocellular carcinoma. Hepatitis Monthly 2019;19(3):e85775. | Exclude on population: not cirrhosis |

| Reference | Reason for exclusion |
|---|---|
| Mariam, Arshiya, Miller-Atkins, Galen, Moro, Amika, Rodarte, Alejandro I., Siddiqi, Shirin, Acevedo-Moreno, Lou-Anne, et al. Salivary miRNAs as non-invasive biomarkers of hepatocellular carcinoma: a pilot study. PeerJ 2022;10():e12715. | Unvalidated model / threshold not pre-specified |
| Marin, Daniele, Catalano, Carlo, De Filippis, Gianmaria, Di Martino, Michele, Guerrisi, Antonino, Rossi, Massimo. Detection of hepatocellular carcinoma in patients with cirrhosis: added value of coronal reformations from isotropic voxels with 64-MDCT. AJR. American journal of roentgenology 2009;192(1):180-87. | Exclude on population: clinical signs and symptoms of HCC |
| Maringhini, A, Cottone, M, Sciarrino, E, Marceno, M P, La Seta, F, Fusco, G, Rinaldi, F. Ultrasonography and alpha-fetoprotein in diagnosis of hepatocellular carcinoma in cirrhosis. Digestive diseases and sciences 1988;33(1):47-51. | Exclude on population: clinical signs and symptoms of HCC |
| Marks, Robert M., Ryan, Andrew, Heba, Elhamy R., Tang, An, Wolfson, Tanya J., Gamst, Anthony C., Sirlin, Claude B. Diagnostic per-patient accuracy of an abbreviated hepatobiliary phase gadoxetic acid-enhanced MRI for hepatocellular carcinoma surveillance. AJR. American journal of roentgenology 2015;204(3):527-35. | Exclude on population: not cirrhosis |
| Marotta, F., Chui, D. H., Safran, P. Serum alpha-L-fucosidase. A more sensitive marker for hepatocellular carcinoma? Digestive diseases and sciences 1991;36(7):993-97. | Does not meet full eligibility criteria: pre-2005 |
| Marquardt, C., Tolstik, T., Bielecki, C., Kaufmann, R., Crecelius, A. C., Schubert, U. S., et al. MALDI imaging-based classification of hepatocellular carcinoma and non-malignant lesions in fibrotic liver tissue. Zeitschrift fur Gastroenterologie 2015;53(1):33-39. | Exclude on population: tissue samples |
| Marquardt, Jens U., Nguyen-Tat, Marc, Galle, Peter R. Surveillance of Hepatocellular Carcinoma and Diagnostic Algorithms in Patients with Liver Cirrhosis. Visceral medicine 2016;32(2):110-15. | Exclude on study design: not a 1- or 2-gate test accuracy study |
| Marrero, Jorge A, Romano, Patrick R, Nikolaeva, Olga, Steel, Laura, Mehta, Anand, Fimmel, Claus J, et al. GP73, a resident Golgi glycoprotein, is a novel serum marker for hepatocellular carcinoma. Journal of hepatology 2005;43(6):1007-12. | Does not meet full eligibility criteria: includes people without cirrhosis |
| Marrero, Jorge A, Su, Grace L, Wei, Wei, Emick, Dawn, Conjeevaram, Hari S, Fontana, Robert J. Des-gamma carboxyprothrombin can differentiate hepatocellular carcinoma from nonmalignant chronic liver disease in american patients. Hepatology (Baltimore, Md.) 2003;37(5):1114-21. | Does not meet full eligibility criteria: pre-2005 |
| Marrero, Jorge A. Newer markers for hepatocellular carcinoma. Gastroenterology 2004;127(5 Suppl 1):S113-19. | Exclude on study design: not a 1- or 2-gate test accuracy study |
| Marrero, Jorge A., Feng, Ziding, Wang, Yinghui, Nguyen, Mindie H., Befeler, Alex S., Roberts, Lewis R., et al. Alpha-fetoprotein, des-gamma carboxyprothrombin, and lectin-bound alpha-fetoprotein in early hepatocellular carcinoma. Gastroenterology 2009;137(1):110-18. | Duplicate (including Cochrane includes) |
| Marrero, Jorge A., Su, Grace L., Wei, Wei, Emick, Dawn, Conjeevaram, Hari S., Fontana, Robert J. Des-gamma carboxyprothrombin can differentiate hepatocellular carcinoma from nonmalignant chronic liver disease in american patients. Hepatology (Baltimore, Md.) 2003;37(5):1114-21. | Does not meet full eligibility criteria: includes people without cirrhosis |
| Marschner, C. A., Zhang, L., Schwarze, V., Volckers, W., Froelich, M. F., Von Munchhausen, N., et al. The diagnostic value of contrast-enhanced ultrasound (CEUS) for assessing hepatocellular carcinoma compared to histopathology; A retrospective single-center analysis of 119 patients. Clinical Hemorheology and Microcirculation 2020;76(4):453-58. | Exclude on population: not cirrhosis |
| Marsh, T. L., Johnston, J. M., Homan, C., Townshend-Bulson, L. J., Kim, N. J., VoPham, T., et al. HCC surveillance in hepatitis C: A longitudinal algorithm improves alpha-fetoprotein screening. Hepatology Communications 2025;9(6):e0719. | No 2x2 data |
| Maryam, Maria. Study of promoter hypomethylation profiles of RAS oncogenes in hepatocellular carcinoma derived from hepatitis C virus genotype 3a in Pakistani population. Journal of medical virology 2018;90(9):1516-23. | Exclude on population: not cirrhosis |

| Reference | Reason for exclusion |
|---|---|
| Masarone, M., Troisi, J., Aglitti, A., Torre, P., Colucci, A., Dallio, M., et al. Untargeted metabolomics as a diagnostic tool in NAFLD: discrimination of steatosis, steatohepatitis and cirrhosis. Metabolomics 2021;17(2):12. | Exclude on target condition: not HCC |
| Mashaly, Aya H, Anwar, Rokiah, Ebrahim, Mohamed A, Eissa, Laila A. Diagnostic and Prognostic Value of Talin-1 and Midkine as Tumor Markers in Hepatocellular Carcinoma in Egyptian Patients. Asian Pacific journal of cancer prevention : APJCP 2018;19(6):1503-08. | Does not meet full eligibility criteria: includes people without cirrhosis |
| Mashaly, Aya H., Anwar, Rokiah, Ebrahim, Mohamed A., Eissa, Laila A. Diagnostic and Prognostic Value of Talin-1 and Midkine as Tumor Markers in Hepatocellular Carcinoma in Egyptian Patients. Asian Pacific journal of cancer prevention : APJCP 2018;19(6):1503-08. | Does not meet full eligibility criteria: includes people without cirrhosis |
| Masuda, Koichi, Kaneko, Junichi, Kawaguchi, Yoshikuni, Togashi, Junichi, Arita, Junichi, Akamatsu, Nobuhisa, et al. Diagnostic accuracy of indocyanine green fluorescence imaging and multidetector row computed tomography for identifying hepatocellular carcinoma with liver explant correlation. Hepatology research : the official journal of the Japan Society of Hepatology 2017;47(12):1299-3007. | Exclude on population: clinical signs and symptoms of HCC |
| Masuzaki, R., Tateishi, R., Yoshida, H., Sato, S., Kato, N., Kanai, F., et al. Risk assessment of hepatocellular carcinoma in chronic hepatitis C patients by transient elastography. Journal of Clinical Gastroenterology 2008;42(7):839-43. | Exclude on population: not cirrhosis |
| Matboli, M., Labib, M. E., Nasser, H. E. T., El-Tawdi, A. H. F., Habib, E. K. Exosomal miR-1298 and lncRNA-rp11-583F2.2 expression in hepatocellular carcinoma. Current Genomics 2020;21(1):37-46. | Exclude on population: not cirrhosis |
| Matboli, M., Shafei, A. E., Ali, M. A., Ashry, A. M., Kamal, K. M., Agag, M. A., et al. circRNAs (hsa_circ_00156, hsa_circ _000224, and hsa_circ _000520) are novel potential biomarkers in hepatocellular carcinoma. Journal of Cellular Biochemistry 2019;120(5):7711-24. | Exclude on population: not cirrhosis |
| Matievskaya, N. V., Tsyrkunov, V. M., Wawrzynowicz-Syczewska, M. Serum alpha-fetoprotein (AFP) levels in patients with chronic HBV and HCV infections. Gastroenterologia Polska 2003;10(1):35-40. | Does not meet full eligibility criteria: pre-2005 |
| Matsubara, Tokuhiro, Kanto, Tatsuya, Kuroda, Shoko, Yoshio, Sachiyo, Higashitani, Koyo, Kakita, Naruyasu, et al. TIE2-expressing monocytes as a diagnostic marker for hepatocellular carcinoma correlates with angiogenesis. Hepatology (Baltimore, Md.) 2013;57(4):1416-25. | Exclude on population: HCC participants not treatment-naive |
| Matsuda, Yasunori, Yamagiwa, Yoko, Fukushima, Koji, Ueno, Yoshiyuki. Expression of galectin-3 involved in prognosis of patients with hepatocellular carcinoma. Hepatology research : the official journal of the Japan Society of Hepatology 2008;38(11):1098-11. | Exclude on population: not cirrhosis |
| Matsuki, Y., Mitamura, K., Yamaguchi, T., Tanaka, N., Aikawa, T., Takahashi, A., et al. Clinical significance of abnormal prothrombin (PIVKA-II) in patients with hepatocellular carcinoma. Detection of PIVKA-II by ELISA using anti-PIVKA-II monoclonal antibody. Acta Hepatologica Japonica 1987;28(8):1073-79. | Does not meet full eligibility criteria: pre-2005 |
| Matsumoto, Y. & Suzuki, T. Early detection of hepatoma by screening test of minimal determination of serum AFP. Journal of Japan Society for Cancer Therapy 1977;15 TH CONGR.():285. | Does not meet full eligibility criteria: pre-2005 |
| Matsumura, M., Niwa, Y., Kato, N., Komatsu, Y., Shiina, S., Kawabe, T., et al. Detection of alpha-fetoprotein mRNA, an indicator of hematogenous spreading hepatocellular carcinoma, in the circulation: A possible predictor of metastatic hepatocellular carcinoma. Hepatology 1994;20(6):1418-25. | Does not meet full eligibility criteria: includes people without cirrhosis |
| Matteucci, Claudia, Sorrentino, Roberta, Bellis, Lia, Ettorre, Giuseppe Maria, Svicher, Valentina, Santoro, Roberto, et al. Detection of high levels of Survivin-immunoglobulin M immune complex in sera from hepatitis C virus infected patients with cirrhosis. Hepatology research : the official journal of the Japan Society of Hepatology 2014;44(9):1008-18. | Exclude on index test |
| Mauduit Astolfi, I & Nogueira Soriano, J M. [Hepatic cirrhosis and hepatocarcinoma. Diagnostic value of echography]. Cirrosis hepatica y hepatocarcinoma. Valor diagnostico de la ecografia. 1987;72(6):701-4. | Does not meet full eligibility criteria: pre-2005 |

| Reference | Reason for exclusion |
|---|---|
| Maussier, M. L., Valenza, V., Schinco, G. AFP, CEA, CA 19-9 and TPA in the hepatocellular carcinoma. International Journal of Biological Markers 1990;5(3):121-26. | Does not meet full eligibility criteria: includes people without cirrhosis |
| McMahon, B J, Bulkow, L, Harpster, A, Snowball, M, Lanier, A, Sacco, F, Dunaway, E. Screening for hepatocellular carcinoma in Alaska natives infected with chronic hepatitis B: a 16-year population-based study. Hepatology (Baltimore, Md.) 2000;32(4 Pt 1):842-6. | Exclude on population: not cirrhosis |
| McNamara, M. M., Thomas, J. V., Alexander, L. F., Little, M. D., Bolus, D. N., Li, Yufeng E. Diffusion-weighted MRI as a screening tool for hepatocellular carcinoma in cirrhotic livers: correlation with explant data-a pilot study. Abdominal radiology (New York) 2018;43(10):2686-92. | Exclude on population: clinical signs and symptoms of HCC |
| Mehinovic, Lejla, Islamagic, Erna, Husic-Selimovic, Azra, Kurtovic-Kozaric, Amina, Vukobrat-Bijedic, Zora. Evaluation of Diagnostic Efficiency of Alpha-Fetoprotein in Patients with Liver Cirrhosis and Hepatocellular Carcinoma: Single-Center Experience. Open access Macedonian journal of medical sciences 2018;6(9):1668-73. | Does not meet full eligibility criteria: includes people without cirrhosis |
| Mehinovic, Lejla, Islamagic, Erna, Husic-Selimovic, Azra, Kurtovic-Kozaric, Amina, Vukobrat-Bijedic, Zora. Evaluation of Diagnostic Efficiency of Alpha-Fetoprotein in Patients with Liver Cirrhosis and Hepatocellular Carcinoma: Single-Center Experience. Open access Macedonian journal of medical sciences 2018;6(9):1668-73. | Duplicate (including Cochrane includes) |
| Mehta, Anand S., Lau, Daryl T. Y., Wang, Mengjun, Aslam, Aysha, Nasir, Bilal, Javaid, Asad, Poongkunran, Mugilan. Application of the Doylestown algorithm for the early detection of hepatocellular carcinoma. PloS one 2018;13(8):e0203149. | Exclude on population: not cirrhosis |
| Mei, M., Liu, D., Tang, X., You, Y., Peng, B., He, X. Vitamin B6 Metabolic Pathway is Involved in the Pathogenesis of Liver Diseases via Multi-Omics Analysis. Journal of Hepatocellular Carcinoma 2022;9():729-50. | Does not meet full eligibility criteria: includes people without cirrhosis |
| Mendy, M., Kirk, G. D., van der Sande, M., Jeng-Barry, A., Lesi, O. A., Hainaut, P., et al. Hepatitis B surface antigenaemia and alpha-foetoprotein detection from dried blood spots: applications to field-based studies and to clinical care in hepatitis B virus endemic areas. Journal of viral hepatitis 2005;12(6):642-47. | Does not meet full eligibility criteria: includes people without cirrhosis |
| Meng, Fan-Long & Wang, Wei. Diagnostic and prognostic significance of serum miR-24-3p in HBV-related hepatocellular carcinoma. Medical oncology (Northwood, London, England) 2014;31(9):177. | Exclude on population: not cirrhosis |
| Meng, X., Gao, J., Sun, Y., Duan, F., Chen, B., Lv, G., et al. Fusing Positive and Negative CT Contrast Nanoagent for the Sensitive Detection of Hepatoma. Advanced science (Weinheim, Baden-Wurttemberg, Germany) 2023;():e2304668. | Exclude on study design: not a 1- or 2-gate test accuracy study |
| Meng, Zuowei, Ren, Qingqi, Zhong, Guolin, Li, Shiyong, Chen, Yan, Wu, Wei, et al. Noninvasive Detection of Hepatocellular Carcinoma with Circulating Tumor DNA Features and alpha-Fetoprotein. The Journal of molecular diagnostics : JMD 2021;23(9):1174-84. | Exclude on population: healthy controls |
| Meren, E., Sawai, Y., Fukuda, K., Igura, T., Kogita, S., Yukimura, Y., et al. Risk stratification of hepatocellular carcinoma in patients with chronic liver disease by combining gadolinium-ethoxybenzyl diethylenetriamine-pentaacetic acid-enhanced magnetic resonance imaging and magnetic resonance elastography. GastroHep 2021;3(7):435-42. | Exclude on population: not cirrhosis |
| Metwally, A. The value of king's score as a predictor of risk of hepatocellular carcinoma among Egyptian patients with hepatitis C virus-related cirrhosis. Middle East Journal of Cancer 2021;12(4):499-505. | Exclude on index test |
| Miao, L. L., Wang, J. W., Liu, H. H., Gao, S., Fan, Y. C. Hypomethylation of glycine dehydrogenase promoter in peripheral blood mononuclear cells is a new diagnostic marker of hepatitis B virus-associated hepatocellular carcinoma. Hepatobiliary and Pancreatic Diseases International 2023;():n. pag.. | Does not meet full eligibility criteria: includes people without cirrhosis |
| Miao, Li-Li, Wang, Jing-Wen, Liu, Hui-Hui, Gao, Shuai, Fan, Yu-Chen. Hypomethylation of glycine dehydrogenase promoter in peripheral blood mononuclear cells is a new diagnostic marker of hepatitis B virus-associated hepatocellular carcinoma. Hepatobiliary & pancreatic diseases international : HBPD INT 2024;23(1):35â€“42. | Exclude on population: not cirrhosis |

| Reference | Reason for exclusion |
| --- | --- |
| Miao, X., Peng, C., Yan, F., Guo, X., Xia, L., Song, Q., An, X. Serum beta-klotho is a potential biomarker for the progression of hepatitis B virus-related liver diseases. Journal of Infection in Developing Countries 2024;18(4):618 " 626. | Does not meet full eligibility criteria: includes people without cirrhosis |
| Michael, T. G., Helal, E. M. B., Sayed, M. M., Agwa, S. H., Elwakeel, S. M. Serum microrna-122 levels in egyptian patients with chronic hepatitis c virus genotype 4 infection before and after treatment with direct acting antiviral drugs. European Journal of Molecular and Clinical Medicine 2020;7(11):1321-33. | CONFERENCE ABSTRACT |
| Middleton, Catrin H., Irving, William, Robertson, John F. R., Murray, Andrea, Parsy-Kowalska, Celine B., Macdonald, Isabel K., et al. Serum autoantibody measurement for the detection of hepatocellular carcinoma. PloS one 2014;9(8):e103867. | Exclude on population: not cirrhosis |
| Miller, W. J., Baron, R. L., Dodd, G. D., 3rd. Malignancies in patients with cirrhosis: CT sensitivity and specificity in 200 consecutive transplant patients. Radiology 1994;193(3):645-50. | Does not meet full eligibility criteria: pre-2005 |
| Miller-Atkins, Galen, Acevedo-Moreno, Lou-Anne, Grove, David, Dweik, Raed A., Tonelli, Adriano R., Brown, J. Mark, et al. Breath Metabolomics Provides an Accurate and Noninvasive Approach for Screening Cirrhosis, Primary, and Secondary Liver Tumors. Hepatology communications 2020;4(7):1041-55. | Exclude on index test |
| Millet, John D., Kamaya, Aya, Choi, Hailey H., Dahiya, Nirvikar, Murphy, Paul M., Naveed, Mujtaba Z., et al. ACR Ultrasound Liver Reporting and Data System: Multicenter Assessment of Clinical Performance at One Year. Journal of the American College of Radiology : JACR 2019;16(12):1656-62. | Does not meet full eligibility criteria: includes people without cirrhosis |
| Mima, S. & Fukuda, M. Studies on early detection of hepatocellular carcinoma. Mass survey by real-time ultrasound and serum battery tests. Acta Hepatologica Japonica 1985;26(8):1026-33. | Does not meet full eligibility criteria: pre-2005 |
| Min B., Moon I., Park H., Jang E., Jeong S.-H. Serial measurements do not increase performance of alpha-fetoprotein for hepatocellular carcinoma surveillance in hepatitis B virus-related liver cirrhosis. Journal of Hepatology 2014;60(1 SUPPL. 1):S258. | CONFERENCE ABSTRACT |
| Minami, T., Tateishi, R., Kondo, M., Nakagomi, R., Fujiwara, N., Sato, M., et al. Serum Alpha-Fetoprotein Has High Specificity for the Early Detection of Hepatocellular Carcinoma after Hepatitis C Virus Eradication in Patients. Medicine (United States) 2015;94(23):e901. | Duplicate (including Cochrane includes) |
| Minami, Tatsuya, Tateishi, Ryosuke, Kondo, Masyuko, Nakagomi, Ryo, Fujiwara, Naoto, Sato, Masaya, et al. Serum Alpha-Fetoprotein Has High Specificity for the Early Detection of Hepatocellular Carcinoma After Hepatitis C Virus Eradication in Patients. Medicine 2015;94(23):e901. | Exclude on population: not cirrhosis |
| Ming, X. L., Feng, Y. L., He, D. D., Luo, C. L., Rong, J. L., Zhang, W. W., et al. Role of BCYRN1 in hepatocellular carcinoma pathogenesis by lncRNA-miRNA-mRNA network analysis and its diagnostic and prognostic value. Epigenomics 2019;11(10):1209-31. | Does not meet full eligibility criteria: includes people without cirrhosis |
| Mita, Y., Aoyagi, Y., Yanagi, M., Suda, T., Suzuki, Y. The usefulness of determining des-gamma-carboxy prothrombin by sensitive enzyme immunoassay in the early diagnosis of patients with hepatocellular carcinoma. Cancer 1998;82(9):1643-48. | Does not meet full eligibility criteria: includes people without cirrhosis |
| Miura, N., Shiota, G., Nakagawa, T., Maeda, Y., Sano, A., Marumoto, A., et al. Sensitive detection of human telomerase reverse transcriptase mRNA in the serum of patients with hepatocellular carcinoma. Oncology 2003;64(4):430-34. | Does not meet full eligibility criteria: pre-2005 |
| Miura, Norimasa, Maeda, Yoshiko, Kanbe, Takamasa, Yazama, Hiroaki, Takeda, Yohei, Sato, Reina, et al. Serum human telomerase reverse transcriptase messenger RNA as a novel tumor marker for hepatocellular carcinoma. Clinical cancer research : an official journal of the American Association for Cancer Research 2005;11(9):3205-09. | Exclude on population: not cirrhosis |
| Miura, Norimasa, Maruyama, Shigeo, Oyama, Kenji, Horie, Yutaka, Kohno, Michimori, Noma, Eijiro, et al. Development of a novel assay to quantify serum human telomerase reverse transcriptase messenger RNA and its significance as a tumor marker for hepatocellular carcinoma. Oncology 2007;72 Suppl 1(ohw, 0135054):45-51. | Exclude on population: not cirrhosis |

| Reference | Reason for exclusion |
|---|---|
| Miura, Norimasa, Maruyama, Shigeo, Oyama, Kenji, Horie, Yutaka, Kohno, Michimori, Noma, Eijiro, et al. Development of a novel assay to quantify serum human telomerase reverse transcriptase messenger RNA and its significance as a tumor marker for hepatocellular carcinoma. Oncology 2007;72 Suppl 1():45-51. | Exclude on population: not cirrhosis |
| Miura, Norimasa, Osaki, Yukio, Nagashima, Miki, Kohno, Michimori, Yorozu, Kensho, Shomori, Kohei, et al. A novel biomarker TERTmRNA is applicable for early detection of hepatoma. BMC gastroenterology 2010;10():46. | Duplicate (including Cochrane includes) |
| Miura, Norimasa, Osaki, Yukio, Nagashima, Miki, Kohno, Michimori, Yorozu, Kensho, Shomori, Kohei, et al. A novel biomarker TERTmRNA is applicable for early detection of hepatoma. BMC gastroenterology 2010;10(100968547):46. | Exclude on population: not cirrhosis |
| Mo, Cuiju, Wu, Junrong, Sui, Jingzhe, Deng, Yan, Li, Meng, Cao, Zhao, et al. Long non-coding RNA LINC01793 as a potential diagnostic biomarker of hepatitis B virus-related hepatocellular carcinoma. Clinical biochemistry 2022;():n. pag.. | Does not meet full eligibility criteria: includes people without cirrhosis |
| Mo, Cuiju, Wu, Junrong, Sui, Jingzhe, Deng, Yan, Li, Meng, Cao, Zhao, et al. Long non-coding RNA LINC01793 as a potential diagnostic biomarker of hepatitis B virus-related hepatocellular carcinoma. Clinical biochemistry 2022;108():56-62. | No 2x2 data |
| Mocan, T., Simao, A. L., Castro, R. E., Rodrigues, C. M. P., Slomka, A., Wang, B., et al. Liquid biopsies in hepatocellular carcinoma: Are we winning? Journal of Clinical Medicine 2020;9(5):1541. | Exclude on study design: not a 1- or 2-gate test accuracy study |
| Mohamed A.A., El-Toukhy N., Ghaith D.M., Badawy I., Abdo S.M., Elkadeem M., Mahrous M.N. Talin-1 gene expression as a tumor marker in hepatocellular carcinoma patients: A pilot study. Open Biomarkers Journal 2020;10(1):15-22. | Exclude on population: healthy controls |
| Mohamed, A. A., Abd-Elsalam, S., El-Daly, M. M., Kamal, N., Saed, S. M., Mohamoud, S., et al. Insulin growth factor-1 as a predictor for the progression of hepatic disease in chronic hepatitis B virus infection. Open Biomarkers Journal 2021;11(1):1-7. | Does not meet full eligibility criteria: includes people without cirrhosis |
| Mohamed, A. A., Abo-Amer, Y. E. E., Aalkhalegy, A., Fathalla, L. A., Elmaghraby, M. B., Elhoseeny, M. M., et al. COL1A1 Gene Expression in Hepatitis B Virus (HBV) Related Hepatocellular Carcinoma (HCC) Egyptian's Patients. Open Biomarkers Journal 2021;11(1):108-14. | No 2x2 data |
| Mohamed, A. A., El-Toukhy, N., Alkhalegy, A. A. Osteopontin as a tumor marker for hepatocellular carcinoma. Journal of Gastroenterology and Hepatology Research 2016;5(4):2140-46. | Duplicate (including Cochrane includes) |
| Mohamed, A. A., El-Toukhy, N., Ghaith, D. M., Badawy, I., Abdo, S. M., Elkadeem, M., Mahrous, M. N. Talin-1 gene expression as a tumor marker in hepatocellular carcinoma patients: A pilot study. Open Biomarkers Journal 2020;10(1):15-22. | Duplicate (including Cochrane includes) |
| Mohamed, A. A., Ghanem, H. M., Kamal, M. M., Ahmed, R., Madkour, N. K., Abdou, D., et al. Dickkopf-1 and beta-catenin as biomarkers for early diagnosis of hepato-cellular carcinoma. Current Cancer Therapy Reviews 2020;16(2):136-44. | Does not meet full eligibility criteria: includes people without cirrhosis |
| Mohamed, A. A., Hashem, A., Yassin, A. S., Elhusseiny, E. M., Saleh, M. A., Ahmed, O. A., Shabana, S. S. Human aldehyde dehydrogenase (ALDH) in cirrhotic and hepatocellular carcinoma patients related to hepatitis C virus. Journal of Gastroenterology and Hepatology Research 2015;4(10):1792-96. | Does not meet full eligibility criteria: includes people without cirrhosis |
| Mohamed, A. A., Nagah Amer, N., Osama, N., Hafez, W., Abdelrahman Ali, A. E., Shaheen, M. M., et al. Expression of miR-15b-5p and toll-like receptor4 as potential novel diagnostic biomarkers for hepatitis C virus-induced hepatocellular carcinoma. Non-coding RNA Research 2025;10():262 " 268. | Does not meet full eligibility criteria: includes people without cirrhosis |
| Mohamed, A. A., Omran, D., El-Feky, S., Darwish, H., Kassas, A. E. L., Farouk, A., et al. Toll-like receptor 7 mRNA is reduced in hepatitis C-based liver cirrhosis and hepatocellular carcinoma, out-performs alpha-fetoprotein levels, and with age and serum aspartate aminotransferase is a new diagnostic index. British Journal of Biomedical Science 2020;():1-5. | Does not meet full eligibility criteria: includes people without cirrhosis |
| Mohamed, Amal Ahmed, Ali-Eldin, Zainab A., Elbedewy, Tamer A., El-Serafy, Magdy, Ali-Eldin, Fatma A. MicroRNAs and clinical implications in hepatocellular carcinoma. World journal of hepatology 2017;9(23):1001-07. | Exclude on population: healthy controls |

| Reference | Reason for exclusion |
| --- | --- |
| Mohamed, F. Z., Hussein, Y. M., El-Deen, I. M. Cyclooxygenase-2 single-nucleotide polymorphisms and hepatocellular carcinoma in Egypt. Molecular Biology Reports 2014;41(3):1461-68. | Genomic biomarker not validated |
| Mohamed, Ghada Abdelrahman, Nashaat, Ehab Hasan, Fawzy, Hadeer Mohamed. Assessment of fibroblast growth factor 19 as a non-invasive serum marker for hepatocellular carcinoma. World journal of hepatology 2022;14(3):623-33. | Exclude on population: healthy controls |
| Mohamed, Nadia A., Swify, Eman M., Amin, Nabila F., Soliman, Mona M., Tag-Eldin, Lubna M. Is serum level of methylated RASSF1A valuable in diagnosing hepatocellular carcinoma in patients with chronic viral hepatitis C? Arab journal of gastroenterology : the official publication of the Pan-Arab Association of Gastroenterology 2012;13(3):111-15. | Exclude on population: not cirrhosis |
| Mohamedein, A., Yousif-Kadaru, A. G., Ahmed, S. A., Saida, H., Zaki, Z. A., Eldin. Acarboxy prothrombin (PIVKA II) as a tumour marker for hepatocellular carcinoma and other liver diseases. East African medical journal 1995;72(9):584-87. | Does not meet full eligibility criteria: pre-2005 |
| Mohammed, M. A., Omar, N. M., Mohammed, S. A. Serum microRNA-1246 as a potential biomarker for HCV-related early-stage hepatocellular carcinoma. International Journal of Cancer Research 2019;15(2):47-57. | Does not meet full eligibility criteria: includes people without cirrhosis |
| Mohana Devi, S., Balachandar, V., Arun, M., Suresh Kumar, S., Balamurali Krishnan, B. Analysis of genetic damage and gene polymorphism in hepatocellular carcinoma (HCC) patients in a South Indian population. Digestive Diseases and Sciences 2013;58(3):759-67. | Exclude on population: healthy controls |
| Mohyeldeen, Mai, Ibrahim, Safinaz, Shaker, Olfat. Serum expression and diagnostic potential of long non-coding RNAs NEAT1 and TUG1 in viral hepatitis C and viral hepatitis C-associated hepatocellular carcinoma. Clinical biochemistry 2020;84():38-44. | Exclude on population: not cirrhosis |
| Mok, Tony S K, Yu, Simon C H, Lee, Conrad, Sung, Joseph, Leung, Nancy, Lai, Paul, et al. False-negative rate of abdominal sonography for detecting hepatocellular carcinoma in patients with hepatitis B and elevated serum alpha-fetoprotein levels. AJR. American journal of roentgenology 2004;183(2):453-8. | Exclude on population: clinical signs and symptoms of HCC |
| Mok, Tony S. K., Yeo, Winnie, Yu, Simon, Lai, Paul, Chan, Henry L. Y., Chan, Anthony T. C., et al. An intensive surveillance program detected a high incidence of hepatocellular carcinoma among hepatitis B virus carriers with abnormal alpha-fetoprotein levels or abdominal ultrasonography results. Journal of clinical oncology : official journal of the American Society of Clinical Oncology 2005;23(31):8041-47. | Exclude on study design: not a 1- or 2-gate test accuracy study |
| Molina-Pelayo, F. A., Zarate-Lopez, D., Garcia-Carrillo, R., Rodriguez-Beas, C., Iniguez-Palomares, R., Rodriguez-Mejia, J. L., et al. miRNAs-Set of Plasmatic Extracellular Vesicles as Novel Biomarkers for Hepatocellular Carcinoma Diagnosis Across Tumor Stage and Etiologies. International Journal of Molecular Sciences 2025;26(6):2563. | Does not meet full eligibility criteria: includes people without cirrhosis |
| Montalto, G., Iovanna, J. L., Soresi, M., Dusetti, N., Carroccio, A., Barthelemy-Bialas, S., Cartabellotta, A. Clinical evaluation of pancreatitis-associated protein as a serum marker of hepatocellular carcinoma: comparison with alpha-fetoprotein. Oncology 1998;55(5):421-25. | Does not meet full eligibility criteria: includes people without cirrhosis |
| Moon, C. M., Shin, S. S., Heo, S. H. Metabolic alterations associated with early-stage hepatocellular carcinoma and their correlation with aging and enzymatic activity in patients with viral hepatitis-induced liver cirrhosis: A preliminary study. Journal of Clinical Medicine 2020;9(3):765. | Exclude on index test |
| Morace, C., Cucunato, M., Bellerone, R., De Caro, G., Crino, S., Fortiguerra, A., et al. Insulin-like growth factor-II is a useful marker to detect hepatocellular carcinoma? European journal of internal medicine 2012;23(6):e157-61. | Does not meet full eligibility criteria: includes people without cirrhosis |
| Moradi, N., Paryan, M., Khansarinejad, B., Sarmadian, H. Plasma level of miR-5193 as a novel biomarker for diagnosis of HBV-related hepatocellular carcinoma. Hepatitis Monthly 2019;19(2):e84455. | Exclude on population: not cirrhosis |
| Morgul, M. H., Klunk, S., Anastasiadou, Z., Gauger, U., Dietel, C., Reutzel-Selke, A., et al. Diagnosis of HCC for patients with cirrhosis using miRNA profiles of the tumor-surrounding tissue - A statistical model based on stepwise | Exclude on population: tissue samples |

| Reference | Reason for exclusion |
|---|---|
| penalized logistic regression. Experimental and Molecular Pathology 2016;101(2):165-71. | |
| Mori, Kensaku, Scheidler, Juergen, Helmberger, Thomas, Holzknecht, Nicolaus, Schauer, Rolf, Schirren, Carl Albrecht, et al. Detection of malignant hepatic lesions before orthotopic liver transplantation: accuracy of ferumoxides-enhanced MR imaging. AJR. American journal of roentgenology 2002;179(4):1045-51. | Does not meet full eligibility criteria: includes people without cirrhosis |
| Mori, S., Aoyagi, Y., Yanagi, M., Suzuki, Y. Serum N-acetylglucosaminyltransferase III activities in hepatocellular carcinoma. Journal of gastroenterology and hepatology 1998;13(6):610-19. | Exclude on index test |
| Mori, Y., Iwama, S., Mori, T., Ueda, S., Iesato, K., Yoshida, H., et al. Liver-specific F antigen in the serum of patients with liver diseases and the detection of early stage hepatocellular carcinoma. Clinica chimica acta; international journal of clinical chemistry 1987;164(2):127-37. | Exclude on index test |
| Moriya, Satoshi, Morimoto, Manabu, Numata, Kazushi, Nozaki, Akito, Shimoyama, Yu, Kondo, Masaaki, et al. Fucosylated fraction of alpha-fetoprotein as a serological marker of early hepatocellular carcinoma. Anticancer research 2013;33(3):997-1001. | Exclude on population: not cirrhosis |
| Moriya, Satoshi, Morimoto, Manabu, Numata, Kazushi, Nozaki, Akito, Shimoyama, Yu, Kondo, Masaaki, et al. Fucosylated fraction of alpha-fetoprotein as a serological marker of early hepatocellular carcinoma. Anticancer research 2013;33(3):997-1001. | Exclude on population: not cirrhosis |
| Moriyama, A, Tabaru, A, Unoki, H, Abe, S, Masumoto, A. Plasma nitrite/nitrate concentrations as a tumor marker for hepatocellular carcinoma. Clinica chimica acta; international journal of clinical chemistry 2000;296(1-2):181-91. | Exclude on population: not cirrhosis |
| Moriyama, A., Tabaru, A., Unoki, H., Abe, S., Masumoto, A. Plasma nitrite/nitrate concentrations as a tumor marker for hepatocellular carcinoma. Clinica chimica acta; international journal of clinical chemistry 2000;296(1-2):181-91. | Exclude on population: not cirrhosis |
| Morota, Kaori, Nakagawa, Masatoshi, Sekiya, Rika, Hemken, Philip M., Sokoll, Lori J., Elliott, Debra, Chan, Daniel W. A comparative evaluation of Golgi protein-73, fucosylated hemopexin, alpha-fetoprotein, and PIVKA-II in the serum of patients with chronic hepatitis, cirrhosis, and hepatocellular carcinoma. Clinical chemistry and laboratory medicine 2011;49(4):711-18. | Exclude on population: healthy controls |
| Morsi, M. I., Hussein, A. E., Mostafa, M., El-Abd, E. Evaluation of tumour necrosis factor-alpha, soluble P-selectin, gamma-glutamyl transferase, glutathione S-transferase-pi and alpha-fetoprotein in patients with hepatocellular carcinoma before and during chemotherapy. British Journal of Biomedical Science 2006;63(2):74-78. | Exclude on population: not cirrhosis |
| Mortele, K. J., De Keukeleire, K., Praet, M., Van Vlierberghe, H., de Hemptinne, B. Malignant focal hepatic lesions complicating underlying liver disease: dual-phase contrast-enhanced spiral CT sensitivity and specificity in orthotopic liver transplant patients. European radiology 2001;11(9):1631-38. | Does not meet full eligibility criteria: pre-2005 |
| Moshiri, Farzaneh, Salvi, Alessandro, Gramantieri, Laura, Sangiovanni, Angelo, Guerriero, Paola, De Petro, Giuseppina, et al. Circulating miR-106b-3p, miR-101-3p and miR-1246 as diagnostic biomarkers of hepatocellular carcinoma. Oncotarget 2018;9(20):15350-64. | Does not meet full eligibility criteria: includes people without cirrhosis |
| Mostafa, A., Ibrahim, N. E., Sabry, D., Fathy, W. Insulin-like growth factor initiates hepatocellular carcinoma in chronic hepatitis c virus patients through induction of long non-coding ribonucleic acids af085935. Open Access Macedonian Journal of Medical Sciences 2021;9():222-28. | Exclude on population: not cirrhosis |
| Motawi, Tarek K., Shaker, Olfat G., El-Maraghy, Shohda A. Serum MicroRNAs as Potential Biomarkers for Early Diagnosis of Hepatitis C Virus-Related Hepatocellular Carcinoma in Egyptian Patients. PloS one 2015;10(9):e0137706. | Does not meet full eligibility criteria: includes people without cirrhosis |
| Motawi, Tarek M. K., El-Maraghy, Shohda A., Sabry, Dina. The expression of long non coding RNA genes is associated with expression with polymorphisms of HULC rs7763881 and MALAT1 rs619586 in hepatocellular carcinoma and HBV Egyptian patients. Journal of cellular biochemistry 2019;120(9):14645-56. | Exclude on population: not cirrhosis |

| Reference | Reason for exclusion |
|---|---|
| Motawi, Tarek M. K., Sadik, Nermin A. H., Shaker, Olfat G. Elevated serum microRNA-122/222 levels are potential diagnostic biomarkers in Egyptian patients with chronic hepatitis C but not hepatic cancer. Tumour biology : the journal of the International Society for Oncodevelopmental Biology and Medicine 2016;37(7):9865-74. | Exclude on population: not cirrhosis |
| Motosugi, U., Ichikawa, T., Koshiishi, T., Sano, K., Morisaka, H., Ichikawa, S., et al. Liver stiffness measured by magnetic resonance elastography as a risk factor for hepatocellular carcinoma: A preliminary case-control study. European Radiology 2013;23(1):156-62. | Exclude on population: not cirrhosis |
| Motosugi, Utaroh, Ichikawa, Tomoaki, Sou, Hironobu, Sano, Katsuhiro, Tominaga, Licht, Muhi, Ali. Distinguishing hypervascular pseudolesions of the liver from hypervascular hepatocellular carcinomas with gadoxetic acid-enhanced MR imaging. Radiology 2010;256(1):151-58. | Exclude on population: clinical signs and symptoms of HCC |
| Moudi, Bita & Mahmoudzadeh-Sagheb, Hamidreza. Hepatocyte paraffin 1 and arginase-1 are effective panel of markers in HBV-related HCC diagnosis in fine-needle aspiration specimens. BMC research notes 2020;13(1):388. | Exclude on population: not cirrhosis |
| Mourad, Lobna, El-Ahwany, Eman, Zoheiry, Mona, Abu-Taleb, Hoda, Hassan, Marwa, Ouf, Amged, et al. Expression analysis of liver-specific circulating microRNAs in HCV-induced hepatocellular Carcinoma in Egyptian patients. Cancer biology & therapy 2018;19(5):400-06. | Exclude on population: healthy controls |
| Mucke, Victoria T., Thomas, Dominique, Mucke, Marcus M., Waidmann, Oliver, Zeuzem, Stefan, Sarrazin, Christoph, et al. Serum sphingolipids predict de novo hepatocellular carcinoma in hepatitis C cirrhotic patients with sustained virologic response. Liver international : official journal of the International Association for the Study of the Liver 2019;39(11):2174-83. | No 2x2 data |
| Mukozu, Takanori, Nagai, Hidenari, Matsui, Daigo, Kanekawa, Takenori. Serum VEGF as a tumor marker in patients with HCV-related liver cirrhosis and hepatocellular carcinoma. Anticancer research 2013;33(3):1013-21. | Duplicate (including Cochrane includes) |
| Murayama, Hiroshi, Fukuda, Yoshihiro, Tsunekawa, Shoji, Ikemoto, Masaki. Ratio of serum ornithine carbamoyltransferase to alanine aminotransferase as a potent indicator for hepatocellular carcinoma. Clinical biochemistry 2007;40(13-14):1077-80. | Does not meet full eligibility criteria: includes people without cirrhosis |
| Murugavel, Kailapuri G., Mathews, S., Jayanthi, V., Shankar, Esaki Muthu, Hari, R., Surendran, R., et al. Alpha-fetoprotein as a tumor marker in hepatocellular carcinoma: investigations in south Indian subjects with hepatotropic virus and aflatoxin etiologies. International journal of infectious diseases : IJID : official publication of the International Society for Infectious Diseases 2008;12(6):e71-76. | Does not meet full eligibility criteria: includes people without cirrhosis |
| Mustika, Syifa & Wijaya, Hengki. The Expressions of CD44, CD90 and Alpha Fetoprotein Biomarkers in Indonesian Patients with Advanced Liver Disease: an Observational Study. Acta medica Indonesiana 2019;51(2):137-44. | Does not meet full eligibility criteria: includes people without cirrhosis |
| Mustika, Syifa & Wijaya, Hengki. The Expressions of CD44, CD90 and Alpha Fetoprotein Biomarkers in Indonesian Patients with Advanced Liver Disease: an Observational Study. Acta medica Indonesiana 2019;51(2):137-44. | Exclude on population: not cirrhosis |
| Muzzillo, D. A., Imoto, M., Fukuda, Y., Koyama, Y., Saga, S., Nagai, Y. Clinical evaluation of serum tissue inhibitor of metalloproteinases-1 levels in patients with liver diseases. Journal of gastroenterology and hepatology 1993;8(5):437-41. | Does not meet full eligibility criteria: pre-2005 |
| N Zekri, Abdel-Rahman, El Kassas, Mohamed, Salam, El SayedTarekAbd El, Hassan, Reem Mostafa, Mohanad, Marwa, Gabr, Reham Mohamed, et al. The possible role of Dickkopf-1, Golgi protein- 73 and Midkine as predictors of hepatocarcinogenesis: a review and an Egyptian study. Scientific reports 2020;10(1):5156. | Exclude on population: healthy controls |
| N.E, E. L-Abd, Fawzy, N. A., S.M, E. L-Sheikh. Circulating miRNA-122, miRNA-199a, and miRNA-16 as Biomarkers for Early Detection of Hepatocellular Carcinoma in Egyptian Patients with Chronic Hepatitis C Virus Infection. Molecular Diagnosis and Therapy 2015;19(4):213-20. | Duplicate (including Cochrane includes) |
| Na, Keun, Jeong, Seul-Ki, Lee, Min Jung, Cho, Sang Yun, Kim, Sun A, Lee, Min-Ji, et al. Human liver carboxylesterase 1 outperforms alpha-fetoprotein as | Does not meet full eligibility criteria: includes people without cirrhosis |

| Reference | Reason for exclusion |
|---|---|
| biomarker to discriminate hepatocellular carcinoma from other liver diseases in Korean patients. International journal of cancer 2013;133(2):408-15. | |
| Na, Keun, Jeong, Seul-Ki, Lee, Min Jung, Cho, Sang Yun, Kim, Sun A., Lee, Min-Ji, et al. Human liver carboxylesterase 1 outperforms alpha-fetoprotein as biomarker to discriminate hepatocellular carcinoma from other liver diseases in Korean patients. International journal of cancer 2013;133(2):408-15. | Duplicate (including Cochrane includes) |
| Na, Keun, Lee, Eun-Young, Lee, Hyoung-Joo, Kim, Kwang-Youl, Lee, Hanna, Jeong, Seul-Ki, et al. Human plasma carboxylesterase 1, a novel serologic biomarker candidate for hepatocellular carcinoma. Proteomics 2009;9(16):3989-99. | Exclude on index test |
| Nabih, Mona I & Aref, Wael M. Significance of plasma osteopontin in diagnosis of hepatitis C virus-related hepatocellular carcinoma. Arab journal of gastroenterology : the official publication of the Pan-Arab Association of Gastroenterology 2014;15(3-4):103-7. | Exclude on population: healthy controls |
| Nabih, Mona I. & Aref, Wael M. Significance of plasma osteopontin in diagnosis of hepatitis C virus-related hepatocellular carcinoma. Arab journal of gastroenterology : the official publication of the Pan-Arab Association of Gastroenterology 2014;15(3-4):103-07. | Duplicate (including Cochrane includes) |
| Nadarevic, Tin, Colli, Agostino, Giljaca, Vanja, Fraquelli, Mirella, Casazza, Giovanni, Manzotti, Cristina, Stimac, Davor. Magnetic resonance imaging for the diagnosis of hepatocellular carcinoma in adults with chronic liver disease. The Cochrane database of systematic reviews 2022;5():CD014798. | Exclude on study design: not a 1- or 2-gate test accuracy study |
| Nafee, Abeer M., Pasha, Heba F., Abd El Aal, Salah M. Clinical significance of serum clusterin as a biomarker for evaluating diagnosis and metastasis potential of viral-related hepatocellular carcinoma. Clinical biochemistry 2012;45(13-14):1070-74. | Exclude on population: not cirrhosis |
| Nagai, T., Murota, M., Nishioka, M., Fujita, J., Ohtsuki, Y., Dohmoto, K., et al. Elevation of cytokeratin 19 fragment in serum in patients with hepatoma: its clinical significance. European journal of gastroenterology & hepatology 2001;13(2):157-61. | Exclude on index test |
| Nagakawa, K., Hidaka, M., Hara, T., Matsushima, H., Imamura, H., Tanaka, T., et al. Serum wisteria floribunda agglutinin-positive human Mac-2 binding protein is unsuitable as a diagnostic marker of occult hepatocellular carcinoma in end-stage liver cirrhosis. PLoS ONE 2023;18(11 NOVEMBER):e0293593. | Does not meet full eligibility criteria: includes people without cirrhosis |
| Nahon, Pierre, Amathieu, Roland, Triba, Mohamed N., Bouchemal, Nadia, Nault, Jean-Charles, Ziol, Marianne, et al. Identification of serum proton NMR metabolomic fingerprints associated with hepatocellular carcinoma in patients with alcoholic cirrhosis. Clinical cancer research : an official journal of the American Association for Cancer Research 2012;18(24):6714-22. | Exclude on index test |
| Nakagawa, C., Oikawa, T., Yamada, K., Tsubota, A., Saeki, C., Katagiri, K., et al. Protein kinase C delta enhances the diagnostic performance of hepatocellular carcinoma. Biomarkers 2024;29(2):55 “ 67. | Exclude on population: not cirrhosis |
| Nakagawa, Chika, Oikawa, Tsunekazu, Yamada, Kohji, Tsubota, Akihito, Saeki, Chisato, Katagiri, Kuniko, et al. Protein kinase C delta enhances the diagnostic performance of hepatocellular carcinoma. Biomarkers : biochemical indicators of exposure, response, and susceptibility to chemicals 2024;29(2):55â€“67. | Duplicate (including Cochrane includes) |
| Nakagawa, T., Seki, T., Shiro, T., Wakabayashi, M., Itoh, T., Tagawa, Y., et al. Usefulness of the high sensitivity PIVKA-II measurement method in diagnosis of hepatocellular carcinoma: A comparison with the conventional method. International Hepatology Communications 1994;2(2):94-98. | Does not meet full eligibility criteria: includes people without cirrhosis |
| Nakamura, J., Yazawa, S., Hada, T., Asao, T., Naitoh, H., Takenoshita, S., et al. The usefulness of anti-fucosylated antigen antibody YB-2 for diagnosis of hepatocellular carcinoma. Glycoconjugate journal 1997;14(1):81-87. | Does not meet full eligibility criteria: includes people without cirrhosis |
| Nakamura, Shinichiro, Nouso, Kazuhiro, Sakaguchi, Kohsaku, Ito, Yoichi M, Ohashi, Yasuo, Kobayashi, Yoshiyuki, et al. Sensitivity and specificity of des-gamma-carboxy prothrombin for diagnosis of patients with hepatocellular carcinomas varies according to tumor size. The American journal of gastroenterology 2006;101(9):2038-43. | Exclude on population: not cirrhosis |

| Reference | Reason for exclusion |
|---|---|
| Nakamura, Shinichiro, Nouso, Kazuhiro, Sakaguchi, Kohsaku, Ito, Yoichi M., Ohashi, Yasuo, Kobayashi, Yoshiyuki, et al. Sensitivity and specificity of des-gamma-carboxy prothrombin for diagnosis of patients with hepatocellular carcinomas varies according to tumor size. The American journal of gastroenterology 2006;101(9):2038-43. | Exclude on population: not cirrhosis |
| Nakano, S., Kumada, T., Sugiyama, K., Watahiki, H. Clinical significance of serum ferritin determination for hepatocellular carcinoma. The American journal of gastroenterology 1984;79(8):623-27. | Does not meet full eligibility criteria: pre-2005 |
| Nakashio, R., Kitamoto, M., Tahara, H., Nakanishi, T., Ide, T. Significance of telomerase activity in the diagnosis of small differentiated hepatocellular carcinoma. International journal of cancer 1997;74(2):141-47. | No 2x2 data |
| Nakatsura, Tetsuya, Yoshitake, Yoshihiro, Senju, Satoru, Monji, Mikio, Komori, Hiroyuki, Motomura, Yutaka, et al. Glypican-3, overexpressed specifically in human hepatocellular carcinoma, is a novel tumor marker. Biochemical and biophysical research communications 2003;306(1):16-25. | Exclude on index test |
| Nakatsura, Tetsuya. Usefulness of the novel oncofetal antigen glypican-3 for diagnosis of hepatocellular carcinoma and melanoma. BioDrugs : clinical immunotherapeutics, biopharmaceuticals and gene therapy 2005;19(2):71-77. | Exclude on study design: not a 1- or 2-gate test accuracy study |
| Nartey, Y. A., Yang, J. D., Zemla, T. J., Ayawin, J., Asibey, S. O., El-Kassas, M., et al. GALAD Score for the Diagnosis of Hepatocellular Carcinoma in Sub-Saharan Africa: A Validation Study in Ghanaian Patients. Cancer research communications 2024;4(10):2653 “ 2659. | No 2x2 data |
| Nartey, Yvonne Ayerki, Awuku, Yaw Asante, Agyei-Nkansah, Adwoa, Duah, Amoako, Bampoh, Sally Afua, Ayawin, Joshua, et al. Ambulatory end-stage liver disease in Ghana; patient profile and utility of alpha fetoprotein and aspartate aminotransferase: platelet ratio index. BMC gastroenterology 2020;20(1):428. | Exclude on population: not cirrhosis |
| Nassar, A. K., El-Toukhy, N. E. T. R., Mokhles, M. A., Mohamed, A. A., Siddik, R. I., El-Hanafi, H. RECK gene polymorphism in patients with hepatocellular carcinoma. Meta Gene 2019;19():149-54. | Does not meet full eligibility criteria: includes people without cirrhosis |
| Nassar, Eman Saad, Elkalbashawy, Yomna Abdelrazek, Kamal, Ahmed. Galectin-3 is not useful for hepatocellular carcinoma surveillance in cirrhotic patients but it may be a marker of cirrhosis development. Clinical and experimental hepatology 2021;7(1):74-78. | Exclude on study design: not a 1- or 2-gate test accuracy study |
| Nasser, Mona Zaky, Zayed, Naglaa Ali, Mohamed, Ahmed Mahmoud, Attia, Dina, Esmat, Gamal. Circulating microRNAs (miR-21, miR-223, miR-885-5p) along the clinical spectrum of HCV-related chronic liver disease in Egyptian patients. Arab journal of gastroenterology : the official publication of the Pan-Arab Association of Gastroenterology 2019;20(4):198-204. | Exclude on population: healthy controls |
| Nasser, R., Al Haddad, M., El-Kassas, M. M., Metwally, F. M., El-Mezayen, H. A. Diagnostic Performance of Epithelial Cell Adhesion Molecule for Early Detection of Hepatocellular Carcinoma Among HCV High-Risk Patients. Asian Pacific journal of cancer prevention : APJCP 2024;25(3):1045 “ 1052. | Continuous test without threshold |
| Navadurong, H., Laohasurayotin, K., Yorwittaya, K., Tiyarattanachai, T., Tanpowpong, N., Pisuchpen, N., et al. ghted imaging; PERFORMANCE OF ABBREVIATED MAGNETIC RESONANCE IMAGING VERSUS ULTRASONOGRAPHY AS AN IMAGING TOOL FOR HEPATOCELLULAR CARCINOMA SURVEILLANCE. ghted imaging; Gastroenterology 2022;162(7 Supplement):S-1164. | CONFERENCE ABSTRACT |
| Nayak, Akash, Baidya Kayal, Esha, Arya, Manish, Culli, Jayanth, Krishan, Sonal, Agarwal, Sumeet. Computer-aided diagnosis of cirrhosis and hepatocellular carcinoma using multi-phase abdomen CT. International journal of computer assisted radiology and surgery 2019;14(8):1341-52. | Exclude on index test |
| Negut, A. C., O, S. Andulescu, Carap, M., Toderici, M. A. Early serum screening for hepatocellular-carcinoma in patients with hepatitis. BMC Infectious Diseases 2014;14(7):n. pag.. | CONFERENCE ABSTRACT |
| Nelles, C., Wagner, A., Lennartz, S., Reimer, R. W. M., Bunck, A. C., Pennig, L., et al. Clinical Benefit of Structured Reporting Using the Liver Imaging Reporting and Data System (LI-RADS) in MRI in Patients at Risk for | Exclude on population: not cirrhosis |

| Reference | Reason for exclusion |
|---|---|
| Hepatocellular Carcinoma. RoFo Fortschritte auf dem Gebiet der Rontgenstrahlen und der Bildgebenden Verfahren 2025;():n. pag.. | |
| Ngo, M., Dao, T., Hoang, T., Nguyen, U., Stenman, J., Duong, H. Accurate quantification of cell-free Ceruloplasmin mRNA as a biomarker for early detection of hepatocellular carcinoma. Scientific reports 2025;15(1):14660. | Exclude on population: not cirrhosis |
| Nguyen Huu, H., Suong, N. T. B., Nguyen Hoang, B. Human telomerase reverse transcriptase messenger RNA (HTERT MRNA) for hepatocellular carcinoma detection in comparison with galad score. Clinica Chimica Acta 2024;558(Supplement 1):118875. | CONFERENCE ABSTRACT |
| Nguyen, Hoang Bac, Le, Xuan-Thao Thi, Nguyen, Huy Huu, Vo, Thanh Thanh, Le, Minh Khoi, Nguyen, Ngan Trung, et al. Diagnostic Value of hTERT mRNA and in Combination With AFP, AFP-L3%, Des-gamma-carboxyprothrombin for Screening of Hepatocellular Carcinoma in Liver Cirrhosis Patients HBV or HCV-Related. Cancer informatics 2022;21():11769351221100730. | Does not meet full eligibility criteria: includes people without cirrhosis |
| Nguyen, Mindie H, Garcia, Ruel T, Simpson, Peter W, Wright, Teresa L. Racial differences in effectiveness of alpha-fetoprotein for diagnosis of hepatocellular carcinoma in hepatitis C virus cirrhosis. Hepatology (Baltimore, Md.) 2002;36(2):410-7. | Does not meet full eligibility criteria: includes people without cirrhosis |
| Nguyen, Mindie H., Garcia, Ruel T., Simpson, Peter W., Wright, Teresa L. Racial differences in effectiveness of alpha-fetoprotein for diagnosis of hepatocellular carcinoma in hepatitis C virus cirrhosis. Hepatology (Baltimore, Md.) 2002;36(2):410-17. | Does not meet full eligibility criteria: includes people without cirrhosis |
| Ni, R. Z., Huang, J. F., Xiao, M. B., Zhang, P. Y. Detection of GGT-II by dot-ELISA with monoclonal antibody in the diagnosis of hepatocellular carcinoma. Ai zheng = Aizheng = Chinese journal of cancer 2004;23(1):66-68. | Does not meet full eligibility criteria: pre-2005 |
| Ni, Run-Zhou, Huang, Jie-Fei, Xiao, Ming-Bing, Li, Mei. Glycylproline dipeptidyl aminopeptidase isoenzyme in diagnosis of primary hepatocellular carcinoma. World journal of gastroenterology 2003;9(4):710-13. | Exclude on population: not cirrhosis |
| Nie, Guilin, Peng, Dingzhong, Li, Bei, Lu, Jiong, Cai, Yulong, Xiong, Xianze. Diagnostic Accuracy of Serum/Plasma Circular RNAs and the Combination of Circular RNAs and alpha-Fetoprotein for Detecting Hepatocellular Carcinoma: A Meta-Analysis. Frontiers in genetics 2021;12():722208. | Exclude on study design: not a 1- or 2-gate test accuracy study |
| Nie, Guilin, Peng, Dingzhong, Li, Bei, Lu, Jiong. Diagnostic Accuracy of Circular RNAs in Different Types of Samples for Detecting Hepatocellular Carcinoma: A Meta-Analysis. Frontiers in genetics 2021;12():794105. | Exclude on study design: not a 1- or 2-gate test accuracy study |
| Nikolic, J. A., Stajic, M., Cuperlovic, M., Hajdukovic, L. Serum alpha-fetoprotein levels and microheterogeneity in patients with different liver diseases. Journal of hepatology 1990;11(2):252-56. | Does not meet full eligibility criteria: includes people without cirrhosis |
| Nikzad, N., Fuentes, D. T., Roach, M., Chowdhury, T., Cagley, M., Badawy, M., et al. Enhancement Pattern Mapping for Early Detection of Hepatocellular Carcinoma in Patients with Cirrhosis. Journal of Hepatocellular Carcinoma 2024;11():595 “ 606. | Exclude on index test |
| Ning, Chun, Cai, Peng, Liu, Xiaofan, Li, Guangtao, Bao, Pengfei, Yan, Lu, et al. A comprehensive evaluation of full-spectrum cell-free RNAs highlights cell-free RNA fragments for early-stage hepatocellular carcinoma detection. EBioMedicine 2023;93():104645. | Does not meet full eligibility criteria: includes people without cirrhosis |
| Noda, Yoshifumi, Goshima, Satoshi, Kajita, Kimihiro, Kawada, Hiroshi, Kawai, Nobuyuki, Koyasu, Hiromi, Matsuo, Masayuki. Biliary tract enhancement in gadoxetic acid-enhanced MRI correlates with liver function biomarkers. European journal of radiology 2016;85(11):2001-07. | Exclude on target condition: not HCC |
| Noda, Yoshifumi, Kanematsu, Masayuki, Goshima, Satoshi, Kondo, Hiroshi, Watanabe, Haruo, Kawada, Hiroshi, et al. Reducing iodine load in hepatic CT for patients with chronic liver disease with a combination of low-tube-voltage and adaptive statistical iterative reconstruction. European journal of radiology 2015;84(1):11-18. | Exclude on population: clinical signs and symptoms of HCC |
| Noh, Choong-Kyun, Kim, Soon Sun, Kim, Dong-Kyu, Lee, Hyun-Young, Cho, Hyo Jung, Yoon, So Young, et al. Inter-alpha-trypsin inhibitor heavy chain H4 as a diagnostic and prognostic indicator in patients with hepatitis B virus-associated hepatocellular carcinoma. Clinical biochemistry 2014;47(13-14):1257-61. | Exclude on index test |

| Reference | Reason for exclusion |
|---|---|
| Nomair, Azhar Mohamed, Issa, Noha M., Madkour, Marwa Ahmed. The clinical significance of serum miRNA-224 expression in hepatocellular carcinoma. Clinical and experimental hepatology 2020;6(1):20-27. | Meets all eligibility criteria but data are untrustworthy |
| Nomair, Azhar Mohamed, Madkour, Marwa Ahmed, Shamseya, Mohammed Mohammed, Elsheredy, Heba Gaber. Profiling of plasma metabolomics in patients with hepatitis C-related liver cirrhosis and hepatocellular carcinoma. Clinical and experimental hepatology 2019;5(4):317-26. | Duplicate (including Cochrane includes) |
| Nomura, F, Ishijima, M, Horikoshi, A, Nakai, T. Determination of serum des-gamma-carboxy prothrombin levels in patients with small-sized hepatocellular carcinoma: comparison of the conventional enzyme immunoassay and two modified methods. The American journal of gastroenterology 1996;91(7):1380-3. | Does not meet full eligibility criteria: pre-2005 |
| Nomura, F, Ishijima, M, Kuwa, K, Tanaka, N, Nakai, T. Serum des-gamma-carboxy prothrombin levels determined by a new generation of sensitive immunoassays in patients with small-sized hepatocellular carcinoma. The American journal of gastroenterology 1999;94(3):650-4. | Does not meet full eligibility criteria: includes people without cirrhosis |
| Nomura, F., Ishijima, M., Horikoshi, A., Nakai, T. Determination of serum des-gamma-carboxy prothrombin levels in patients with small-sized hepatocellular carcinoma: comparison of the conventional enzyme immunoassay and two modified methods. The American journal of gastroenterology 1996;91(7):1380-83. | Does not meet full eligibility criteria: pre-2005 |
| Nomura, F., Ishijima, M., Kuwa, K., Tanaka, N., Nakai, T. Serum des-gamma-carboxy prothrombin levels determined by a new generation of sensitive immunoassays in patients with small-sized hepatocellular carcinoma. The American journal of gastroenterology 1999;94(3):650-54. | Does not meet full eligibility criteria: includes people without cirrhosis |
| Nomura, Fumio, Sogawa, Kazuyuki, Noda, Kenta, Seimiya, Masanori, Matsushita, Kazuyuki, Miura, Toshihide, et al. Serum anti-Ku86 is a potential biomarker for early detection of hepatitis C virus-related hepatocellular carcinoma. Biochemical and biophysical research communications 2012;421(4):837-43. | Does not meet full eligibility criteria: includes people without cirrhosis |
| Nomura, Fumio, Sogawa, Kazuyuki, Noda, Kenta, Seimiya, Masanori, Matsushita, Kazuyuki, Miura, Toshihide, et al. Serum anti-Ku86 is a potential biomarker for early detection of hepatitis C virus-related hepatocellular carcinoma. Biochemical and biophysical research communications 2012;421(4):837-43. | Duplicate (including Cochrane includes) |
| Nouso, K., Furubayashi, Y., Shiota, S., Miyake, N., Oonishi, A., Wakuta, A., et al. Early detection of hepatocellular carcinoma in patients with diabetes mellitus. European Journal of Gastroenterology and Hepatology 2020;32(7):877-81. | Exclude on population: not cirrhosis |
| Nwude, V. N., Lesi, O. A., Onyekwere, C., Charpentier, E. Clinical Characteristics of Hepatitis B Virus-Associated Hepatocellular Carcinoma Patients in Southwest Nigeria. Pathogens 2025;14(2):169. | Exclude on population: not cirrhosis |
| Obuz, Funda, Oksuzler, Mahmut, Secil, Mustafa, Sagol, Ozgul, Karademir, Sedat. Efficiency of MR imaging in the detection of malignant liver lesions. Diagnostic and interventional radiology (Ankara, Turkey) 2006;12(1):17-21. | Exclude on target condition: not HCC |
| Oda, Kohei, Ido, Akio, Tamai, Tsutomu, Matsushita, Masakaze, Kumagai, Kotaro, Mawatari, Sei-ichi, et al. Highly sensitive lens culinaris agglutinin-reactive alpha-fetoprotein is useful for early detection of hepatocellular carcinoma in patients with chronic liver disease. Oncology reports 2011;26(5):1227-33. | Exclude on population: not cirrhosis |
| Odedra, Devang, Jandaghi, Ali Babaei, Bhayana, Rajesh, Elbanna, Khaled Y., Espin-Garcia, Osvaldo, Fischer, Sandra E., et al. Comparison of international guidelines for diagnosis of hepatocellular carcinoma and implications for transplant allocation in liver transplantation candidates with gadoxetic acid enhanced liver MRI versus contrast enhanced CT: a prospective study with liver explant histopathological correlation. Cancer imaging : the official publication of the International Cancer Imaging Society 2022;22(1):55. | Exclude on population: clinical signs and symptoms of HCC |
| Ogawa, K., Suzuki, K., Nakai, M., Sho, T., Suda, G., Morikawa, K. Evaluation of clinical utility of PIVKA-II using a chemiluminescent immunoassay. Acta Hepatologica Japonica 2019;60(11):397-404. | Foreign language |

| Reference | Reason for exclusion |
|---|---|
| Ogle, Laura F., Orr, James G., Willoughby, Catherine E., Hutton, Claire, McPherson, Stuart, Plummer, Ruth, et al. Imagestream detection and characterisation of circulating tumour cells - A liquid biopsy for hepatocellular carcinoma? Journal of hepatology 2016;65(2):305-13. | Exclude on population: not cirrhosis |
| Ohashi, I. & Hanafusa, K. Small hepatocellular carcinomas: two-phase dynamic incremental CT in detection and evaluation. Radiology 1993;189(3):851-55. | Does not meet full eligibility criteria: pre-2005 |
| Ohno, Tsuyoshi, Isoda, Hiroyoshi, Furuta, Akihiro, Arizono, Shigeki, Yamashita, Rikiya, Ono, Ayako. Usefulness of breath-hold inversion recovery-prepared T1-weighted two-dimensional gradient echo sequence for detection of hepatocellular carcinoma in Gd-EOB-DTPA-enhanced MR imaging. Clinical imaging 2016;40(5):997-1003. | Exclude on population: clinical signs and symptoms of HCC |
| Oka, H, Saito, A, Ito, K, Kumada, T, Satomura, S, Kasugai, H, et al. Multicenter prospective analysis of newly diagnosed hepatocellular carcinoma with respect to the percentage of Lens culinaris agglutinin-reactive alpha-fetoprotein. Journal of gastroenterology and hepatology 2001;16(12):1378-83. | Exclude on population: not cirrhosis |
| Oka, H, Tamori, A, Kuroki, T, Kobayashi, K. Prospective study of alpha-fetoprotein in cirrhotic patients monitored for development of hepatocellular carcinoma. Hepatology (Baltimore, Md.) 1994;19(1):61-6. | Does not meet full eligibility criteria: pre-2005 |
| Okabe, Hirohisa, Delgado, Evan, Lee, Jung Min, Yang, Jing, Kinoshita, Hiroki, Hayashi, Hiromitsu, et al. Role of leukocyte cell-derived chemotaxin 2 as a biomarker in hepatocellular carcinoma. PloS one 2014;9(6):e98817. | Exclude on index test |
| Okajima, Wataru, Komatsu, Shuhei, Ichikawa, Daisuke, Miyamae, Mahito, Kawaguchi, Tsutomu, Hirajima, Shoji, et al. Circulating microRNA profiles in plasma: identification of miR-224 as a novel diagnostic biomarker in hepatocellular carcinoma independent of hepatic function. Oncotarget 2016;7(33):53820-36. | Exclude on population: not cirrhosis |
| Okanoue, T., Yamaguchi, K., Shima, T., Mitsumoto, Y., Katayama, T., Okuda, K., et al. Artificial intelligence/neural network system that accurately diagnoses hepatocellular carcinoma in nonalcoholic steatohepatitis. Hepatology Research 2023;():n. pag.. | Does not meet full eligibility criteria: includes people without cirrhosis |
| Okanoue, Takeshi, Yamaguchi, Kanji, Shima, Toshihide, Mitsumoto, Yasuhide, Mizuno, Masayuki, Katayama, Takayuki, et al. The serum levels of IgM-free apoptosis inhibitor of macrophage /CD5L as a predictive and early diagnostic marker for NASH-associated hepatocellular carcinoma. Hepatology research : the official journal of the Japan Society of Hepatology 2022;():n. pag.. | Exclude on population: not cirrhosis |
| Okazaki, N, Yoshida, T, Yoshino, M. Screening of patients with chronic liver disease for hepatocellular carcinoma by ultrasonography. Clinical oncology 1984;10(3):241-6. | Does not meet full eligibility criteria: pre-2005 |
| Okazaki, N., Yoshida, T., Yoshino, M. Screening of patients with chronic liver disease for hepatocellular carcinoma by ultrasonography. Clinical oncology 1984;10(3):241-46. | Does not meet full eligibility criteria: pre-2005 |
| Okazaki, N., Yoshino, M., Yoshida, T., Takayasu, K., Moriyama, N., Makuuchi, M., et al. Early diagnosis of hepatocellular carcinoma. Hepato-gastroenterology 1990;37(5):480-83. | Does not meet full eligibility criteria: pre-2005 |
| Okimoto, Naomasa, Yasaka, Koichiro, Kaiume, Masafumi, Kanemaru, Noriko, Suzuki, Yuichi. Improving detection performance of hepatocellular carcinoma and interobserver agreement for liver imaging reporting and data system on CT using deep learning reconstruction. Abdominal radiology (New York) 2023;48(4):1280-89. | Exclude on population: not cirrhosis |
| Okoth, Frederick A., Iida, Fusae, Tuei, Julius, Kaiguri, Peter M., Kaptich, David C., Kasomo, Anthony M., Ireri, Edmund K. Serological studies and ultrasonographic diagnosis of chronic liver disease in Kenya. African journal of health sciences 1994;1(2):76-78. | Does not meet full eligibility criteria: pre-2005 |
| Okuda, H., Nakanishi, T., Takatsu, K., Saito, A., Hayashi, N., Watanabe, K., et al. Measurement of serum levels of des-gamma-carboxy prothrombin in patients with hepatocellular carcinoma by a revised enzyme immunoassay kit with increased sensitivity. Cancer 1999;85(4):812-18. | Does not meet full eligibility criteria: pre-2005 |
| Okuda, H., Obata, H., Nakanishi, T., Furukawa, R. Production of abnormal prothrombin (des-gamma-carboxy prothrombin) by hepatocellular carcinoma. A clinical and experimental study. Journal of hepatology 1987;4(3):357-63. | Does not meet full eligibility criteria: pre-2005 |

| Reference | Reason for exclusion |
|---|---|
| Ola, O. S., Ogunbiyi, O. J., Olaleye, O. D. Tumour markers and hepatitis C virus infection in Nigerian patients with liver diseases. Nigerian journal of medicine : journal of the National Association of Resident Doctors of Nigeria 2006;15(4):417-20. | Exclude on population: healthy controls |
| Ola, S. O. & Akanji, A. O. The diagnostic utility of serum ferritin. Estimation in patients with primary hepatocellular carcinoma. Tropical and geographical medicine 1995;47(6):302-04. | Does not meet full eligibility criteria: pre-2005 |
| Olivero, A., Gaia, S., Abate, M. L., Troshina, G., Carucci, P., Nicolosi, A., et al. Identification of appropriate cut-off value for the use of protein induced by vitamin K absence or antagonist-II for surveillance of patients with cirrhosis at risk of hepatocellular carcinoma development. Biochimica Clinica 2022;46(3):S47. | CONFERENCE ABSTRACT |
| Omar A., Omran D., Mahmoud M., El Baz T., Medhat E., Hosny A., Salah A. Hepatocellular carcinoma multidisciplinary clinic-Cairo university (HMC-CU) score; a new simple score for early diagnosis of HCC. United European Gastroenterology Journal 2017;5(5 Supplement 1):A184. | CONFERENCE ABSTRACT |
| Omar, A. A. A., El-Awady, R. R., Hassan, S. M. A., Eitah, W. M. S., Ahmed, R., Khater, A., Tantawi, O. M. S. MiR-155 and MiR-665 role as potential non-invasive biomarkers for hepatocellular carcinoma in Egyptian patients with chronic hepatitis C virus infection. Journal of Translational Internal Medicine 2020;8(1):32-40. | Duplicate (including Cochrane includes) |
| Omar, Haniza, Lim, Chun Ren, Chao, Samuel, Lee, Michelle Mei Lin, Bong, Chin Wei, Ooi, Edie Jian Jiek, et al. Blood gene signature for early hepatocellular carcinoma detection in patients with chronic hepatitis B. Journal of clinical gastroenterology 2015;49(2):150-57. | Exclude on population: not cirrhosis |
| Omar, M., Farid, K., Emran, T., El-Taweel, F., Tabll, A. HCC-Mark: a simple non-invasive model based on routine parameters for predicting hepatitis C virus related hepatocellular carcinoma. British journal of biomedical science 2021;78(2):72-77. | Does not meet full eligibility criteria: includes people without cirrhosis |
| Ominde, Sheila T. Multicentre study on dynamic contrast computed tomography findings of focal liver lesions with clinical and histological correlation. SA journal of radiology 2019;23(1):1667. | Exclude on population: clinical signs and symptoms of HCC |
| Omran, Dalia Abd El Hamid, Awad, AbuBakr Hussein, Mabrouk, Mahasen Abd El Rahman, Soliman, Ahmad Fouad. Application of data mining techniques to explore predictors of HCC in Egyptian patients with HCV-related chronic liver disease. Asian Pacific journal of cancer prevention : APJCP 2015;16(1):381-85. | Does not meet full eligibility criteria: includes people without cirrhosis |
| Omran, Mohamed M, Emran, Tarek M, Farid, Khaled, Eltaweel, Fathy M, Omar, Mona A. An Easy and Useful Noninvasive Score Based on alpha-1-acid Glycoprotein and C-Reactive Protein for Diagnosis of Patients with Hepatocellular Carcinoma Associated with Hepatitis C Virus Infection. Journal of immunoassay & immunochemistry 2016;37(3):273-88. | Does not meet full eligibility criteria: includes people without cirrhosis |
| Omran, Mohamed M, Farid, Khaled, Omar, Mona A, Emran, Tarek M, El-Taweel, Fathy M. A combination of alpha-fetoprotein, midkine, thioredoxin and a metabolite for predicting hepatocellular carcinoma. Annals of hepatology 2020;19(2):179-85. | Does not meet full eligibility criteria: includes people without cirrhosis |
| Omran, Mohamed M., Emran, Tarek M., Farid, Khaled, Eltaweel, Fathy M., Omar, Mona A. An Easy and Useful Noninvasive Score Based on alpha-1-acid Glycoprotein and C-Reactive Protein for Diagnosis of Patients with Hepatocellular Carcinoma Associated with Hepatitis C Virus Infection. Journal of immunoassay & immunochemistry 2016;37(3):273-88. | Duplicate (including Cochrane includes) |
| Omran, Mohamed M., Farid, Khaled, Omar, Mona A., Emran, Tarek M., El-Taweel, Fathy M. A combination of alpha-fetoprotein, midkine, thioredoxin and a metabolite for predicting hepatocellular carcinoma. Annals of hepatology 2020;19(2):179-85. | Duplicate (including Cochrane includes) |
| Omran, Mohamed M., Mosaad, Sara, Emran, Tarek M., Eltaweel, Fathy M. A novel model based on interleukin 6 and insulin-like growth factor II for detection of hepatocellular carcinoma associated with hepatitis C virus. Journal, genetic engineering & biotechnology 2021;19(1):168. | Does not meet full eligibility criteria: includes people without cirrhosis |
| Omran, N. M., El-Sherbini, S. M., Hegazy, O., Elshaarawy, A. A. Crosstalk between miR-215 and epithelial-mesenchymal transition specific markers (E- | Exclude on population: healthy controls |

| Reference | Reason for exclusion |
|---|---|
| cadherin and N-cadherin) in different stages of chronic HCV Infection. Journal of Medical Virology 2020;92(8):1231-38. | |
| Ooka, Yoshihiko, Kanai, Fumihiko, Okabe, Shinichiro, Ueda, Takuya, Shimofusa, Ryota, Ogasawara, Sadahisa, et al. Gadoxetic acid-enhanced MRI compared with CT during angiography in the diagnosis of hepatocellular carcinoma. Magnetic resonance imaging 2013;31(5):748-54. | Exclude on population: clinical signs and symptoms of HCC |
| Oon, C. J., Yo, S. L., Chio, L. F. A pilot study on the screening of primary hepatocellular carcinoma in selected high risk groups in the population using multiple tumour markers. Annals of the Academy of Medicine, Singapore 1980;9(2):240-44. | Does not meet full eligibility criteria: pre-2005 |
| Orloff, M. J., Isenberg, J. I., Wheeler, H. O., Haynes, K. S., Jinich-Brook, H., Rapier, R., et al. Hepatocellular carcinoma in a randomized controlled trial of emergency treatment of bleeding esophageal varices in cirrhosis. American Journal of Surgery ;():n. pag.. | Exclude on study design: not a 1- or 2-gate test accuracy study |
| Oshiro, M. M., Akhbari, R. K., Watts, G. S., Futscher, B. W., Vrba, L., Bernert, R. A., Nelson, M. A. NOVEL PLASMA BIOMARKER SET (SENTINEL-10TM) CAN DETECT SMALL HCC WITH ALL OTHER BIOMARKERS NEGATIVE. Gastroenterology 2023;164(6 Supplement):S “ 1314. | CONFERENCE ABSTRACT |
| Osman, A. G., Shaaban Hashem, K., Youssef, L. M. Circulating MicroRNA-21 as a Novel Noninvasive Biomarker for hepatocellular Carcinoma Compared with Alpha Fetoprotein Gold test. Journal of Pure and Applied Microbiology 2021;15(4):2473-85. | Exclude on population: healthy controls |
| Osman, D., Ali, O., Obada, M., El-Mezayen, H. Chromatographic determination of some biomarkers of liver cirrhosis and hepatocellular carcinoma in Egyptian patients. Biomedical Chromatography 2017;31(6):e3893. | Exclude on index test |
| Otsuru, A., Nagataki, S., Koji, T. Analysis of alpha-fetoprotein gene expression in hepatocellular carcinoma and liver cirrhosis by in situ hybridization. Cancer 1988;62(6):1105-12. | Exclude on population: tissue samples |
| Ouda, S. M., Khairy, A. M., Sorour, Ashraf E. Serum Beta-2 Microglobulin: a Possible Marker for Disease Progression in Egyptian Patients with Chronic HCV Related Liver Diseases. Asian Pacific journal of cancer prevention : APJCP 2015;16(17):7825-29. | Exclude on index test |
| Ouedraogo, W., Tran-Van Nhieu, J., Baranes, L., Lin, S. J., Decaens, T., Laurent, A., et al. [Evaluation of noninvasive diagnostic criteria for hepatocellular carcinoma on pretransplant MRI (2010): correlation between MR imaging features and histological features on liver specimen]. Evaluation des criteres diagnostiques non invasifs du carcinome hepatocellulaire sur IRM pre-greffe hepatique (2010) : correlations IRM - anatomopathologiques sur explants hepatiques. 2011;92(7-8):688-700. | Foreign language |
| Oura, Kyoko, Fujita, Koji, Morishita, Asahiro, Iwama, Hisakazu, Nakahara, Mai, Tadokoro, Tomoko, et al. Serum microRNA-125a-5p as a potential biomarker of HCV-associated hepatocellular carcinoma. Oncology letters 2019;18(1):882-90. | Exclude on population: not cirrhosis |
| Oyunsuren, Tsendsuren, Kurbanov, Fuat, Tanaka, Yasuhito, Elkady, Abeer, Sanduijav, Ruvjir, Khajidsuren, Onkhoon, Dagvadorj, Byambin. High frequency of hepatocellular carcinoma in Mongolia; association with mono-, or co-infection with hepatitis C, B, and delta viruses. Journal of medical virology 2006;78(12):1688-95. | Exclude on population: not cirrhosis |
| Oyunsuren, Tsendsuren, Sanduijav, Ruvjir, Davaadorj, Duger. Hepatocellular carcinoma and its early detection by AFP testing in Mongolia. Asian Pacific journal of cancer prevention : APJCP 2006;7(3):460-62. | Does not meet full eligibility criteria: includes people without cirrhosis |
| Ozaki, Kumi, Ohtani, Takashi, Ishida, Tomokazu, Takahashi, Kouki, Ishida, Shota, Takata, Kenji, et al. Liver fibrosis estimated using extracellular volume fraction obtained from dual-energy CT as a risk factor for hepatocellular carcinoma after sustained virologic response: A preliminary case-control study. European journal of radiology 2023;168():111112. | Does not meet full eligibility criteria: includes people without cirrhosis |
| Ozer, Birol, Serin, Ender, Yilmaz, Ugur, Gumurdulu, Yuksel, Saygili, Ozlem B., Kayaselcuk, Fazilet. Clinicopathologic features and risk factors for hepatocellular carcinoma: results from a single center in southern Turkey. The Turkish journal of gastroenterology : the official journal of Turkish Society of Gastroenterology 2003;14(2):85-90. | No 2x2 data |

| Reference | Reason for exclusion |
|---|---|
| Ozkan, H., Erdal, H., Tutkak, H., Karaeren, Z., Yakut, M., Yuksel, O. Diagnostic and prognostic validity of Golgi protein 73 in hepatocellular carcinoma. Digestion 2010;83(1-2):83-88. | Retracted |
| Ozkan, Hasan, Erdal, Harun, Kocak, Erdem, Tutkak, Huseyin, Karaeren, Zihni, Yakut, Mustafa. Diagnostic and prognostic role of serum glypican 3 in patients with hepatocellular carcinoma. Journal of clinical laboratory analysis 2011;25(5):350-53. | Duplicate (including Cochrane includes) |
| Pahade, Jay K., Juice, David, Staib, Lawrence, Israel, Gary, Cornfeld, Dan, Mitchell, Kisha. Is there an added value of a hepatobiliary phase with gadoxetate disodium following conventional MRI with an extracellular gadolinium agent in a single imaging session for detection of primary hepatic malignancies? Abdominal radiology (New York) 2016;41(7):1270-84. | Exclude on population: not cirrhosis |
| Paik, N., Sinn, D. H., Oh, I. S., Kim, J. H., Kang, W., Gwak, G. Y., et al. Non-invasive tests for liver disease severity and the hepatocellular carcinoma risk in chronic hepatitis B patients with low-level viremia. Liver International 2018;38(1):68-75. | Exclude on index test |
| Pan, Hong Y., Wu, Qing Q., Yin, Qiao Q., Dai, Yi N., Huang, Yi C., Zheng, Wei, et al. LC/MS-Based Global Metabolomic Identification of Serum Biomarkers Differentiating Hepatocellular Carcinoma from Chronic Hepatitis B and Liver Cirrhosis. ACS omega 2021;6(2):1160-70. | Exclude on index test |
| Pan, Liuhong, Yao, Min, Zheng, Wenjie, Gu, Juanjuan, Yang, Xuli, Qiu, Liwei, et al. Abnormality of Wnt3a expression as novel specific biomarker for diagnosis and differentiation of hepatocellular carcinoma. Tumour biology : the journal of the International Society for Oncodevelopmental Biology and Medicine 2016;37(4):5561-68. | Exclude on population: not cirrhosis |
| Pan, Y., Dai, J., Hua, X., Chen, J., Chen, Y. The value of combined detection of Pivka-II, AFP and AFP-L3 in the diagnosis of hepatocellular carcinoma. Acta Medica Mediterranea 2019;35(5):2793-97. | Exclude on population: not cirrhosis |
| Pan, Z., Tang, B., Hou, Z., Zhang, J., Liu, H., Huang, G., Yang, Y. XAGE-1b expression is associated with the diagnosis and early recurrence of hepatocellular carcinoma. Molecular and Clinical Oncology 2014;2(6):1155-59. | Exclude on population: healthy controls |
| Papatheodoridi, Alkistis, Chatzigeorgiou, Antonios, Chrysavgis, Lampros, Lembessis, Panagiotis, Loglio, Alessandro, Facchetti, Floriana, et al. Circulating cell-free DNA species affect the risk of hepatocellular carcinoma in treated chronic hepatitis B patients. Journal of viral hepatitis 2021;28(3):464-74. | Exclude on study design: not a 1- or 2-gate test accuracy study |
| Paradis, V. In the new area of noninvasive markers of hepatocellular carcinoma. Journal of Hepatology 2007;46(1):9-11. | Exclude on study design: not a 1- or 2-gate test accuracy study |
| Park Y.-H. Diagnostic predictability of blood tumor markers for hepatocellular carcinoma in patients with liver cirrhosis undergoing liver transplantation. Transplant International 2017;30(Supplement 2):286. | CONFERENCE ABSTRACT |
| Park, Hyo Jung, Jang, Hye Young, Kim, So Yeon, Lee, So Jung, Won, Hyung Jin, Byun, Jae Ho, et al. Non-enhanced magnetic resonance imaging as a surveillance tool for hepatocellular carcinoma: Comparison with ultrasound. Journal of hepatology 2020;72(4):718-24. | Duplicate (including Cochrane includes) |
| Park, Hyo Jung, Jang, Hye Young, Kim, So Yeon, Lee, So Jung, Won, Hyung Jin, Byun, Jae Ho, et al. Non-enhanced magnetic resonance imaging as a surveillance tool for hepatocellular carcinoma: Comparison with ultrasound. Journal of hepatology 2020;72(4):718-24. | Secondary publication of included study |
| Park, Hyo Jung, Kim, So Yeon, Singal, Amit G., Lee, So Jung, Won, Hyung Jin, Byun, Jae Ho, et al. Abbreviated magnetic resonance imaging vs ultrasound for surveillance of hepatocellular carcinoma in high-risk patients. Liver international : official journal of the International Association for the Study of the Liver 2022;42(9):2080-92. | Secondary publication of included study |
| Park, Hyo Jung, Lee, Tae Young, Kim, So Yeon, Kim, Min-Ju, Singal, Amit G., Lee, So Jung, et al. Hypervascular transformation of hepatobiliary phase hypointense nodules without arterial phase hyperenhancement on gadoxetic acid-enhanced MRI: long-term follow-up in a surveillance cohort. European radiology 2022;32(8):5064-74. | Exclude on study design: not a 1- or 2-gate test accuracy study |

| Reference | Reason for exclusion |
|---|---|
| Park, Joong-Won, Kim, Ji Hoon, Kim, Seok Ki, Kang, Keon Wook, Park, Kyung Woo, Choi, Jun-Il, et al. A prospective evaluation of 18F-FDG and 11C-acetate PET/CT for detection of primary and metastatic hepatocellular carcinoma. Journal of nuclear medicine : official publication, Society of Nuclear Medicine 2008;49(12):1912-21. | Exclude on population: clinical signs and symptoms of HCC |
| Park, M. J., Kim, Y. K., Lee, M. W., Lee, W. J., Kim, Y. S., Kim, S. H., Choi, D. Small hepatocellular carcinomas: Improved sensitivity by combining gadoxetic acid-enhanced and diffusion-weighted MR imaging patterns. Radiology 2012;264(3):761-70. | Exclude on population: clinical signs and symptoms of HCC |
| Park, Min Jung, Kim, Young Kon, Lee, Mi Hee. Validation of diagnostic criteria using gadoxetic acid-enhanced and diffusion-weighted MR imaging for small hepatocellular carcinoma (<= 2.0 cm) in patients with hepatitis-induced liver cirrhosis. Acta radiologica (Stockholm, Sweden : 1987) 2013;54(2):127-36. | Exclude on population: clinical signs and symptoms of HCC |
| Park, Mi-Suk, Kim, Sooah, Patel, Jignesh, Hajdu, Cristina H., Do, Richard K. G., Mannelli, Lorenzo, Babb, James S. Hepatocellular carcinoma: detection with diffusion-weighted versus contrast-enhanced magnetic resonance imaging in pretransplant patients. Hepatology (Baltimore, Md.) 2012;56(1):140-48. | Exclude on population: HCC participants not treatment-naive |
| Park, Sang Joon, Jang, Jae Young, Jeong, Soung Won, Cho, Young Kyu, Lee, Sae Hwan, Kim, Sang Gyune, et al. Usefulness of AFP, AFP-L3, and PIVKA-II, and their combinations in diagnosing hepatocellular carcinoma. Medicine 2017;96(11):e5811. | Does not meet full eligibility criteria: includes people without cirrhosis |
| Park, Sang Joon, Jang, Jae Young, Jeong, Soung Won, Cho, Young Kyu, Lee, Sae Hwan, Kim, Sang Gyune, et al. Usefulness of AFP, AFP-L3, and PIVKA-II, and their combinations in diagnosing hepatocellular carcinoma. Medicine 2017;96(11):e5811. | Duplicate (including Cochrane includes) |
| Park, So Hyun, Choi, Moon Hyung, Kim, Bohyun, Lee, Hyun-Soo, Yoon, Sungjin, Lee, Young Joon, Nickel, Dominik. Deep Learning-Accelerated Non-Contrast Abbreviated Liver MRI for Detecting Malignant Focal Hepatic Lesions: Dual-Center Validation. Korean journal of radiology 2025;26(4):333â€“345. | Exclude on population: clinical signs and symptoms of HCC |
| Park, Y., Lim, H. S., Kim, Y. S., Hong, D. J. Soluble human leukocyte antigen-G expression in hepatitis B virus infection and hepatocellular carcinoma. Tissue Antigens ;():n. pag.. | Exclude on index test |
| Pasha, Heba F. & Mohamed, Randa H. RASSF1A and SOCS1 genes methylation status as a noninvasive marker for hepatocellular carcinoma. Cancer biomarkers : section A of Disease markers 2019;24(2):241-47. | Does not meet full eligibility criteria: includes people without cirrhosis |
| Passos-Castilho, Ana Maria, Carvalho, Valdemir Melechco, Cardozo, Karina Helena Morais, Kikuchi, Luciana, Chagas, Aline Lopes, Gomes-Gouvea, Michele Soares, et al. Serum lipidomic profiling as a useful tool for screening potential biomarkers of hepatitis B-related hepatocellular carcinoma by ultraperformance liquid chromatography-mass spectrometry. BMC cancer 2015;15(100967800):985. | Does not meet full eligibility criteria: includes people without cirrhosis |
| Passos-Castilho, Ana Maria, Carvalho, Valdemir Melechco, Cardozo, Karina Helena Morais, Kikuchi, Luciana, Chagas, Aline Lopes, Gomes-Gouvea, Michele Soares, et al. Serum lipidomic profiling as a useful tool for screening potential biomarkers of hepatitis B-related hepatocellular carcinoma by ultraperformance liquid chromatography-mass spectrometry. BMC cancer 2015;15():985. | Duplicate (including Cochrane includes) |
| Patarat, Rathasapa, Riku, Shoji, Kunadirek, Pattapon, Chuaypen, Natthaya, Tangkijvanich, Pisit, Mutirangura, Apiwat. The expression of FLNA and CLU in PBMCs as a novel screening marker for hepatocellular carcinoma. Scientific reports 2021;11(1):14838. | Exclude on population: not cirrhosis |
| Patel, Jaimin, Yopp, Adam, Waljee, Akbar K. Development and Internal Validation of a Model for Early Detection of Hepatocellular Carcinoma in Patients With Cirrhosis. Journal of clinical gastroenterology 2016;50(2):175-79. | No 2x2 data |
| Pateron, D, Ganne, N, Trinchet, J C, Aurousseau, M H, Mal, F, Meicler, C, et al. Prospective study of screening for hepatocellular carcinoma in Caucasian patients with cirrhosis. Journal of hepatology 1994;20(1):65-71. | Does not meet full eligibility criteria: pre-2005 |
| Paul, Shashi Bala, Gulati, Manpreet Singh, Sreenivas, Vishnubhatla, Madan, Kaushal, Gupta, Arun Kumar, Mukhopadhyay, Sima. Evaluating patients with cirrhosis for hepatocellular carcinoma: value of clinical symptomatology, | Duplicate (including Cochrane includes) |

| Reference | Reason for exclusion |
|---|---|
| imaging and alpha-fetoprotein. Oncology 2007;72 Suppl 1(ohw, 0135054):117-23. | |
| Paul, Shashi Bala, Gulati, Manpreet Singh, Sreenivas, Vishnubhatla, Madan, Kaushal, Gupta, Arun Kumar, Mukhopadhyay, Sima. Evaluating patients with cirrhosis for hepatocellular carcinoma: value of clinical symptomatology, imaging and alpha-fetoprotein. Oncology 2007;72 Suppl 1():117-23. | Duplicate (including Cochrane includes) |
| Pauleit, Dirk, Textor, Jochen, Bachmann, Reinald, Conrad, Rudi, Flacke, Sebastian, Layer, Gunter, Kreft, Burkhard. Hepatocellular carcinoma: detection with gadolinium- and ferumoxides-enhanced MR imaging of the liver. Radiology 2002;222(1):73-80. | Does not meet full eligibility criteria: pre-2005 |
| Pavesi, F., Lotzniker, M., Scarabelli, M., Garbagnoli, P. Efficiency of composite laboratory tests in the diagnosis of liver malignancies. The International journal of biological markers 1989;4(3):163-69. | Exclude on population: not cirrhosis |
| Pazgan-Simon, M., Szymanek-Pasternal, A., Gorka-Dynysiewicz, J., Nowicka, A., Simon, K., Grzebyk, E. Serum chemerin level in patients with liver cirrhosis and primary and multifocal hepatocellular carcinoma with consideration of insulin level. Archives of Medical Science 2024;20(5):1504 “ 1510. | No 2x2 data |
| Pei, M. M. & Lu, M. Clinical significance of serum miR-888-5p in patients with primary hepatocellular carcinoma. World Chinese Journal of Digestology 2020;28(7):247-53. | Foreign language |
| Peng, Chengzhi, Yu, Philip Leung Ho, Lu, Jianliang, Cheng, Ho Ming, Shen, Xin-Ping, Chiu, Keith Wan-Hang. Opportunistic Detection of Hepatocellular Carcinoma Using Noncontrast CT and Deep Learning Artificial Intelligence. Journal of the American College of Radiology : JACR 2025;22(3):249â€“259. | Exclude on population: not cirrhosis |
| Peng, Fang, Yuan, Hao, Zhou, Yi-Feng, Wu, Si-Xian, Long, Zhen-Yi. Diagnostic Value of Combined Detection via Protein Induced by Vitamin K Absence or Antagonist II, Alpha-Fetoprotein, and D-Dimer in Hepatitis B Virus-Related Hepatocellular Carcinoma. International journal of general medicine 2022;15():5763-73. | Does not meet full eligibility criteria: includes people without cirrhosis |
| Peng, Y. C. & Chan, C. S. The effectiveness of serum alpha-fetoprotein level in anti-HCV positive patients for screening hepatocellular carcinoma. Hepato-gastroenterology 1999;46(30):3208-11. | Does not meet full eligibility criteria: pre-2005 |
| Peterson, M. S., Baron, R. L., Marsh, J. W., Jr., Oliver, J. H., 3rd, Confer, S. R. Pretransplantation surveillance for possible hepatocellular carcinoma in patients with cirrhosis: epidemiology and CT-based tumor detection rate in 430 cases with surgical pathologic correlation. Radiology 2000;217(3):743-49. | Does not meet full eligibility criteria: pre-2005 |
| Petkevich, A. A., Abramov, A. A., Pospelov, V. I., Malinina, N. A., Kuhareva, E. I., Mazurchik, N. V. Exosomal and non-exosomal mirna expression levels in patients with hcv-related cirrhosis and liver cancer. Oncotarget 2021;12(17):1697-7006. | Does not meet full eligibility criteria: includes people without cirrhosis |
| Phan, Thanh Hai, Chi Nguyen, Van Thien, Thi Pham, Thu Thuy, Nguyen, Van-Chu, Ho, Tan Dat, Quynh Pham, Thi Mong, et al. Circulating DNA methylation profile improves the accuracy of serum biomarkers for the detection of nonmetastatic hepatocellular carcinoma. Future oncology (London, England) 2022;18(39):4399-4113. | Exclude on population: not cirrhosis |
| Phuong, P. C., Khoa, M. T., Loi, N. T., Quynh, V. T. T., Luan, N. D., Mai, N. T. H., et al. GALAD score and a proposal for GALADUS model for detecting hepatocellular carcinoma in Vietnamese patients with chronic liver disease. European Review for Medical and Pharmacological Sciences 2024;28(3):1111 “ 1122. | Exclude on population: not cirrhosis |
| Piciocchi, Marika, Cardin, Romilda, Vitale, Alessandro, Vanin, Veronica, Giacomin, Anna, Pozzan, Caterina, et al. Circulating free DNA in the progression of liver damage to hepatocellular carcinoma. Hepatology international 2013;7(4):1050-57. | Duplicate (including Cochrane includes) |
| Piciocchi, Marika, Cardin, Romilda, Vitale, Alessandro, Vanin, Veronica, Giacomin, Anna, Pozzan, Caterina, et al. Circulating free DNA in the progression of liver damage to hepatocellular carcinoma. Hepatology international 2013;7(4):1050-7. | Exclude on population: not cirrhosis |
| Pinto, E., Lazzarini, E., Pelizzaro, F., Gambato, M., Santarelli, L., Potente, S., et al. Somatic Copy Number Alterations in Circulating Cell-Free DNA as a | No 2x2 data |

| Reference | Reason for exclusion |
|---|---|
| Prognostic Biomarker for Hepatocellular Carcinoma: Insights from a Proof-of-Concept Study. Cancers 2025;17(7):1115. | |
| Piratvisuth, T., Hou, J., Tanwandee, T., Berg, T., Vogel, A., Trojan, J., et al. Development and clinical validation of a novel algorithmic score (GAAD) for detecting HCC in prospective cohort studies. Hepatology Communications 2023;7(11):e0317. | Duplicate (including Cochrane includes) |
| Piratvisuth, Teerha, Hou, Jinlin, Tanwandee, Tawesak, Berg, Thomas, Vogel, Arndt, Trojan, Jorg, et al. Development and clinical validation of a novel algorithmic score (GAAD) for detecting HCC in prospective cohort studies. Hepatology communications 2023;7(11):n. pag.. | Does not meet full eligibility criteria: includes people without cirrhosis |
| Piratvisuth, Teerha, Tanwandee, Tawesak, Thongsawat, Satawat, Sukeepaisarnjaroen, Wattana, Esteban, Juan Ignacio, Bes, Marta, et al. Multimarker Panels for Detection of Early Stage Hepatocellular Carcinoma: A Prospective, Multicenter, Case-Control Study. Hepatology communications 2022;6(4):679-91. | Does not meet full eligibility criteria: includes people without cirrhosis |
| Pissaia, A., Jr., Bernard, D., Scatton, O., Soubrane, O., Conti, F. Significance of serum tumor markers carcinoembryonic antigen, CA 19-9, CA 125, and CA 15-3 in pre-orthotopic liver transplantation evaluation. Transplantation proceedings 2009;41(2):682-84. | Exclude on index test |
| Pizzocolo, G., Salmi, A., Lanza, E., Rangoni, G., Gussago, A., Dovis, M., Callegaro, L. Alpha-fetoprotein monoclonal assay: preliminary clinical findings in a high risk population. The International journal of biological markers 1988;3(1):10-14. | Exclude on target condition: not HCC |
| Polterauer, P. & Legenstein, E. Serum concentrations of tissue polypeptide antigen and alpha1-fetoprotein in patients with primary liver cancer, metastatic liver cancer and cirrhosis of the liver. Wiener Klinische Wochenschrift 1985;97(9):417-20. | Does not meet full eligibility criteria: pre-2005 |
| Pompili, Maurizio, Addolorato, Giovanni, Pignataro, Giulia, Rossi, Cristina, Zuppi, Cecilia, Covino, Marcello, et al. Evaluation of the albumin-gamma-glutamyltransferase isoenzyme as a diagnostic marker of hepatocellular carcinoma-complicating liver cirrhosis. Journal of gastroenterology and hepatology 2003;18(3):288-95. | Does not meet full eligibility criteria: pre-2005 |
| Pompili, Maurizio, Addolorato, Giovanni, Pignataro, Giulia, Rossi, Cristina, Zuppi, Cecilia, Covino, Marcello, et al. Evaluation of the albumin-gamma-glutamyltransferase isoenzyme as a diagnostic marker of hepatocellular carcinoma-complicating liver cirrhosis. Journal of gastroenterology and hepatology 2003;18(3):288-95. | Does not meet full eligibility criteria: pre-2005 |
| Poon, T C, Chan, A T, Zee, B, Ho, S K, Mok, T S, Leung, T W. Application of classification tree and neural network algorithms to the identification of serological liver marker profiles for the diagnosis of hepatocellular carcinoma. Oncology 2001;61(4):275-83. | Does not meet full eligibility criteria: pre-2005 |
| Poon, T. C. W., Chan, A. T. C., Zee, B., Ho, S. K. W., Mok, T. S. K., Leung, T. W. T. Application of classification tree and neural network algorithms to the identification of serological liver marker profiles for the diagnosis of hepatocellular carcinoma. Oncology 2001;61(4):275-83. | Duplicate (including Cochrane includes) |
| Poon, T. C. W., Mok, T. S. K., Chan, A. T. C., Chan, C. M. L., Leong, V., Tsui, S. H. T., et al. Quantification and utility of monosialylated alpha-fetoprotein in the diagnosis of hepatocellular carcinoma with nondiagnostic serum total-alpha-fetoprotein. Clinical Chemistry 2002;48(7):1021-27. | Does not meet full eligibility criteria: includes people without cirrhosis |
| Poon, Terence C. W., Yip, Tai-Tung, Chan, Anthony T. C., Yip, Christine, Yip, Victor, Mok, Tony S. K., et al. Comprehensive proteomic profiling identifies serum proteomic signatures for detection of hepatocellular carcinoma and its subtypes. Clinical chemistry 2003;49(5):752-60. | Exclude on population: not cirrhosis |
| Poortahmasebi, Vahdat, Nejati, Ahmad, Abazari, Mohammad Foad, Nasiri Toosi, Mohsen, Ghaziasadi, Azam, Mohammadzadeh, Nader, et al. Identifying Potential New Gene Expression-Based Biomarkers in the Peripheral Blood Mononuclear Cells of Hepatitis B-Related Hepatocellular Carcinoma. Canadian journal of gastroenterology & hepatology 2022;2022():9541600. | No 2x2 data |

| Reference | Reason for exclusion |
| --- | --- |
| Porta, C, De Amici, M, Quaglini, S, Paglino, C, Tagliani, F, Boncimino, A, Moratti, R. Circulating interleukin-6 as a tumor marker for hepatocellular carcinoma. Annals of oncology : official journal of the European Society for Medical Oncology 2008;19(2):353-8. | Does not meet full eligibility criteria: includes people without cirrhosis |
| Porta, C., De Amici, M., Quaglini, S., Paglino, C., Tagliani, F., Boncimino, A., Moratti, R. Circulating interleukin-6 as a tumor marker for hepatocellular carcinoma. Annals of oncology : official journal of the European Society for Medical Oncology 2008;19(2):353-58. | Does not meet full eligibility criteria: includes people without cirrhosis |
| Pote, Nicolas, Cauchy, Francois, Albuquerque, Miguel, Voitot, Helene, Belghiti, Jacques, Castera, Laurent, et al. Performance of PIVKA-II for early hepatocellular carcinoma diagnosis and prediction of microvascular invasion. Journal of hepatology 2015;62(4):848-54. | Duplicate (including Cochrane includes) |
| Pote, Nicolas, Cauchy, Francois, Albuquerque, Miguel, Voitot, Helene, Belghiti, Jacques, Castera, Laurent, et al. Performance of PIVKA-II for early hepatocellular carcinoma diagnosis and prediction of microvascular invasion. Journal of hepatology 2015;62(4):848-54. | Does not meet full eligibility criteria: includes people without cirrhosis |
| Powell, H., Coarfa, C., Ruiz-Echartea, E., Grimm, S. L., Najjar, O., Yu, B., et al. Differences in Prediagnostic Serum Metabolomic and Lipidomic Profiles Between Cirrhosis Patients with and without Incident Hepatocellular Carcinoma. Journal of Hepatocellular Carcinoma 2024;11():1699 “ 1712. | No 2x2 data |
| Powell-Jackson, P, Forbes, A, Michell, M. Effect of cirrhosis on accuracy of 99Tcm-tin colloid scintigraphy and ultrasound scanning in diagnosis of hepatocellular carcinoma. The British journal of radiology 1987;60(720):1221-2. | Does not meet full eligibility criteria: includes people without cirrhosis |
| Powell-Jackson, P., Forbes, A., Michell, M. Effect of cirrhosis on accuracy of 99Tcm-tin colloid scintigraphy and ultrasound scanning in diagnosis of hepatocellular carcinoma. The British journal of radiology 1987;60(720):1221-22. | Does not meet full eligibility criteria: pre-2005 |
| Pozzan, Caterina, Cardin, Romilda, Piciocchi, Marika, Cazzagon, Nora, Maddalo, Gemma, Vanin, Veronica, et al. Diagnostic and prognostic role of SCCA-IgM serum levels in hepatocellular carcinoma (HCC). Journal of gastroenterology and hepatology 2014;29(8):1637-44. | Does not meet full eligibility criteria: includes people without cirrhosis |
| Pratedrat, Pornpitra, Chuaypen, Natthaya, Nimsamer, Pattaraporn, Payungporn, Sunchai, Pinjaroen, Nutcha, Sirichindakul, Boonchoo. Diagnostic and prognostic roles of circulating miRNA-223-3p in hepatitis B virus-related hepatocellular carcinoma. PloS one 2020;15(4):e0232211. | Exclude on population: not cirrhosis |
| Pu, C., Huang, H., Wang, Z., Zou, W., Lv, Y., Zhou, Z., et al. Extracellular vesicle-associated mir-21 and mir-144 are markedly elevated in serum of patients with hepatocellular carcinoma. Frontiers in Physiology 2018;9(JUL):930. | Exclude on population: not cirrhosis |
| Pu, Li, Jing, Shi, Bianqin, Guo, Ping, Liu, Qindong, Liang, Chenggui, Liu, et al. Development of a Chemiluminescence Immunoassay for Serum YB-1 and its Clinical Application as a Potential Diagnostic Marker for Hepatocellular Carcinoma. Hepatitis monthly 2013;13(7):e8918. | Exclude on population: not cirrhosis |
| Putz, Franz J., Verloh, Niklas, Erlmeier, Anna, Schelker, Roland C., Schreyer, Andreas G., Hautmann, Matthias G., et al. Influence of limited examination conditions on contrast-enhanced sonography for characterising liver lesions. Clinical hemorheology and microcirculation 2019;71(2):267-76. | Exclude on population: not cirrhosis |
| Qayyum, A., Thoeni, R. F., Coakley, F. V., Lu, Y., Quay, J. P. Detection of hepatocellular carcinoma by ferumoxides-enahanced MR imaging in cirrhosis: Incremental value of dynamic gadolinium-enhancement. Journal of Magnetic Resonance Imaging 2006;23(1):17-22. | Exclude on population: clinical signs and symptoms of HCC |
| Qi, Famei, Zhou, Aihua, Yan, Li, Yuan, Xiumei, Wang, Danni, Chang, Ruoyun, et al. The diagnostic value of PIVKA-II, AFP, AFP-L3, CEA, and their combinations in primary and metastatic hepatocellular carcinoma. Journal of clinical laboratory analysis 2020;34(5):e23158. | Exclude on population: not cirrhosis |
| Qi, Famei, Zhou, Aihua, Yan, Li, Yuan, Xiumei, Wang, Danni, Chang, Ruoyun, et al. The diagnostic value of PIVKA-II, AFP, AFP-L3, CEA, and their combinations in primary and metastatic hepatocellular carcinoma. Journal of clinical laboratory analysis 2020;34(5):e23158. | Duplicate (including Cochrane includes) |

| Reference | Reason for exclusion |
|---|---|
| Qi, J., Wang, J., Katayama, H., Sen, S. Circulating microRNAs (cmiRNAs) as novel potential biomarkers for hepatocellular carcinoma. Neoplasma 2013;60(2):135-42. | Exclude on study design: not a 1- or 2-gate test accuracy study |
| Qi, Peng, Cheng, Shu-qun, Wang, Hao, Li, Nan, Chen, Yue-feng. Serum microRNAs as biomarkers for hepatocellular carcinoma in Chinese patients with chronic hepatitis B virus infection. PloS one 2011;6(12):e28486. | Exclude on population: not cirrhosis |
| Qian Yy, M., Yuwei, J. R., Angus, P., Schelleman, T., Johnson, L. Efficacy and cost of a hepatocellular carcinoma screening program at an Australian teaching hospital. Journal of Gastroenterology and Hepatology (Australia) 2010;25(5):951-56. | Does not meet full eligibility criteria: includes people without cirrhosis |
| Qian, E. Na, Han, Shuang-Yin, Ding, Song-Ze. Expression and diagnostic value of CCT3 and IQGAP3 in hepatocellular carcinoma. Cancer cell international 2016;16():55. | Exclude on index test |
| Qian, Xian, Zhan, Qi, Lv, Lei, Zhang, Hai, Hong, Zhanying, Li, Yunqing, et al. Steroid hormone profiles plus alpha-fetoprotein for diagnosing primary liver cancer by liquid chromatography tandem mass spectrometry. Clinica chimica acta; international journal of clinical chemistry 2016;457():92-98. | No 2x2 data |
| Qian, Xiangjun, Liu, Shuhong, Long, Huiling, Zhang, Siyu, Yan, Xiaotong, Yao, Mingjie, et al. Reappraisal of the diagnostic value of alpha-fetoprotein for surveillance of HBV-related hepatocellular carcinoma in the era of antiviral therapy. Journal of viral hepatitis 2021;28(1):20-29. | Does not meet full eligibility criteria: includes people without cirrhosis |
| Qian, Xiangjun, Liu, Yanna, Wu, Fengping, Zhang, Siyu, Gong, Jiao, Nan, Yuemin, et al. The Performance of Serum Alpha-Fetoprotein for Detecting Early-Stage Hepatocellular Carcinoma Is Influenced by Antiviral Therapy and Serum Aspartate Aminotransferase: A Study in a Large Cohort of Hepatitis B Virus-Infected Patients. Viruses 2022;14(8):n. pag.. | Does not meet full eligibility criteria: includes people without cirrhosis |
| Qian, Xiang-Jun, Wen, Zhu-Mei, Huang, Xiao-Ming, Feng, Hui-Juan, Lin, Shan-Shan, Liu, Yan-Na, et al. Better performance of PIVKA-II for detecting hepatocellular carcinoma in patients with chronic liver disease with normal total bilirubin. World journal of gastroenterology 2023;29(8):1359-73. | Exclude on population: not cirrhosis |
| Qian, Yu, Wang, Jing-Wen, Fang, Yu, Yuan, Xiao-Dong, Fan, Yu-Chen, Gao, Shuai. Measurement of Cyclin D2 (CCND2) Gene Promoter Methylation in Plasma and Peripheral Blood Mononuclear Cells and Alpha-Fetoprotein Levels in Patients with Hepatitis B Virus-Associated Hepatocellular Carcinoma. Medical science monitor : international medical journal of experimental and clinical research 2020;26():e927444. | Does not meet full eligibility criteria: includes people without cirrhosis |
| Qiao, Guang-Lei, Chen, Li, Jiang, Wei-Hua, Yang, Cheng, Yang, Chun-Mei, Song, Li-Na, et al. Hsa_circ_0003998 may be used as a new biomarker for the diagnosis and prognosis of hepatocellular carcinoma. OncoTargets and therapy 2019;12():5849-60. | Exclude on population: not cirrhosis |
| Qiao, Y., Chen, J., Li, X., Xiao, F., Chang, L., Zhang, R., Hao, X. Serum gp73 is also a biomarker for diagnosing cirrhosis in population with chronic HBV infection. Clinical biochemistry 2014;47(16-17):216-22. | Does not meet full eligibility criteria: includes people without cirrhosis |
| Qin, Meilin, Liu, Gang, Huo, Xisong, Tao, Xuemei, Sun, Xiaomeng, Ge, Zhouhong, et al. Hsa_circ_0001649: A circular RNA and potential novel biomarker for hepatocellular carcinoma. Cancer biomarkers : section A of Disease markers 2016;16(1):161-69. | Exclude on population: tissue samples |
| Qin, Tao, Liu, Hu, Song, Qi, Song, Geng, Wang, Hong-zhi, Pan, Yue-yin, et al. The screening of volatile markers for hepatocellular carcinoma. Cancer epidemiology, biomarkers & prevention : a publication of the American Association for Cancer Research, cosponsored by the American Society of Preventive Oncology 2010;19(9):2247-53. | Exclude on index test |
| Qin, X., Tang, G., Gao, R., Guo, Z., Liu, Z., Yu, S., et al. A multicenter study on PIVKA reference interval of healthy population and establishment of PIVKA cutoff value for hepatocellular carcinoma diagnosis in China. International journal of laboratory hematology 2017;39(4):392-401. | Does not meet full eligibility criteria: includes people without cirrhosis |
| Qiong, Lan, Jie, Zhao, Zhong, Zheng, Wen, Sheng, Jun, Zhao, Liping, Lu. Detection of hepatocellular carcinoma in a population at risk: iodine-enhanced multidetector CT and/or gadoxetic acid-enhanced 3.0 T MRI. BMJ open 2022;12(2):e058461. | Exclude on population: not cirrhosis |

| Reference | Reason for exclusion |
|---|---|
| Qiu, Cuipeng, Ma, Yangcheng, Wang, Bofei, Zhang, Xiaojun, Wang, Xiao. Autoantibodies to PAX5, PTCH1, and GNA11 as Serological Biomarkers in the Detection of Hepatocellular Carcinoma in Hispanic Americans. International journal of molecular sciences 2023;24(4):n. pag.. | No 2x2 data |
| Qiu, Feng, Qiu, Fanghua, Liu, Lifang, Liu, Jiawei, Xu, Jianhua. The Role of Dermcidin in the Diagnosis and Staging of Hepatocellular Carcinoma. Genetic testing and molecular biomarkers 2018;22(4):218-23. | Does not meet full eligibility criteria: includes people without cirrhosis |
| Qiu, Geng & Fang, Jiancheng. 5' CpG island methylation analysis identifies the MAGE-A1 and MAGE-A3 genes as potential markers of HCC. Clinical biochemistry 2006;39(3):259-66. | Exclude on population: tissue samples |
| Qiu, L. W., Wu, W., Sai, W. L., Yang, J. L., Zhang, H. J., Gu, X., et al. Diagnostic value of expression of micro RNA-183 family members in hepatocellular carcinoma. World Chinese Journal of Digestology 2013;21(16):1487-92. | Foreign language |
| Qiu, Lixia, Xu, Shanshan, Qiu, Yundong, Liu, Yali. Comparison of acknowledged hepatocellular carcinoma risk scores in high-risk hepatitis C patients with sustained virological response. Journal of viral hepatitis 2023;30(6):559-66. | Exclude on study design: not a 1- or 2-gate test accuracy study |
| Qu, B., He, W., Yao, X., Shan, D. nner; DR-CapsNet: deep residual capsule network with dynamic routing for automated identification of hepatocellular carcinoma and cirrhosis in CT images. Biomedical Signal Processing and Control 2025;110():108201. | Exclude on index test |
| Qu, C. & Jiao, Y. A liquid biopsy assay for identifying early-stage hepatocellular carcinoma in asymptomatic HBsAg-seropositive individuals. Molecular and Cellular Oncology 2019;6(5):e1614419. | Exclude on study design: not a 1- or 2-gate test accuracy study |
| Qu, Chunfeng, Wang, Yuting, Wang, Pei, Chen, Kun, Wang, Minjie, Zeng, Hongmei, et al. Detection of early-stage hepatocellular carcinoma in asymptomatic HBsAg-seropositive individuals by liquid biopsy. Proceedings of the National Academy of Sciences of the United States of America 2019;116(13):6308-12. | Exclude on population: not cirrhosis |
| Qu, Kevin Z., Zhang, Ke, Li, HaiRong, Afdhal, Nezam H. Circulating microRNAs as biomarkers for hepatocellular carcinoma. Journal of clinical gastroenterology 2011;45(4):355-60. | Exclude on population: not cirrhosis |
| Qu, Kevin Z., Zhang, Ke, Ma, Wanlong, Li, Hairong, Wang, Xiuqiang, Zhang, Xi, et al. Ubiquitin-proteasome profiling for enhanced detection of hepatocellular carcinoma in patients with chronic liver disease. Journal of gastroenterology and hepatology 2011;26(4):751-58. | Exclude on population: not cirrhosis |
| Qu, Li-Shuai, Liu, Tao-Tao, Jin, Fei, Guo, Yan-Mei, Chen, Tao-Yang, Ni, Zheng-Pin. Combined pre-S deletion and core promoter mutations related to hepatocellular carcinoma: A nested case-control study in China. Hepatology research : the official journal of the Japan Society of Hepatology 2011;41(1):54-63. | Exclude on population: not cirrhosis |
| Queiroz, Marcos Roberto Gomes de, Francisco Neto, Miguel Jose, Garcia, Rodrigo Gobbo, Rahal Junior, Antonio, Salvalaggio, Paolo. High correlation between microbubble contrast-enhanced ultrasound, magnetic resonance and histopathology in the evaluation of hepatocellular carcinoma. Einstein (Sao Paulo, Brazil) 2013;11(4):500-06. | Exclude on population: not cirrhosis |
| Quoc, Nguyen Bao, Phuong, Nguyen Doan Nguyen, Ngan, Tang Kim, Linh, Nguyen Thi Minh, Cuong, Pham Hung. Expression of Plasma hsa-miR122 in HBV-Related Hepatocellular Carcinoma (HCC) in Vietnamese Patients. MicroRNA (Shariqah, United Arab Emirates) 2018;7(2):92-99. | Exclude on population: not cirrhosis |
| Raedle, J, Oremek, G, Truschnowitsch, M, Lorenz, M, Roth, W K, Caspary, W F. Clinical evaluation of autoantibodies to p53 protein in patients with chronic liver disease and hepatocellular carcinoma. European journal of cancer (Oxford, England : 1990) 1998;34(8):1198-203. | Exclude on population: not cirrhosis |
| Raedle, J, Roth, W K, Oremek, G, Caspary, W F. Alpha-fetoprotein and p53 autoantibodies in patients with chronic hepatitis C. Digestive diseases and sciences 1995;40(12):2587-94. | Exclude on population: not cirrhosis |
| Raedle, J., Oremek, G., Truschnowitsch, M., Lorenz, M., Roth, W. K., Caspary, W. F. Clinical evaluation of autoantibodies to p53 protein in patients with | Exclude on population: not cirrhosis |

| Reference | Reason for exclusion |
|---|---|
| chronic liver disease and hepatocellular carcinoma. European Journal of Cancer 1998;34(8):1198-2003. | |
| Raff E., Kakati D.D., Shoreibah M.G., Bloomer J.R. CT and MRI scans are overused for hepatocellular carcinoma screening in patients with cirrhosis. Gastroenterology 2014;146(5 SUPPL. 1):S-606. | CONFERENCE ABSTRACT |
| Rahbin, N., Siosteen, A. K., Elvin, A., Blomqvist, L., Hagen, K., Hultcrantz, R. Detection and characterization of focal liver lesions with contrast-enhanced ultrasonography in patients with hepatitis C-induced liver cirrhosis. Acta radiologica (Stockholm, Sweden : 1987) 2008;49(3):251-57. | Exclude on population: clinical signs and symptoms of HCC |
| Ramachandran, P., Xu, G., Huang, H. H., Rice, R., Zhou, B., Lind-Paintner, K. Serum glycoprotein markers in non-alcoholic steatohepatitis and hepatocellular carcinoma. bioRxiv 2021;():n. pag.. | Exclude on population: not cirrhosis |
| Ramadan, R. A., Madkour, M. A., El-Nagarr, M. M. Serum clusterin as a marker for diagnosing hepatocellular carcinoma. Alexandria Journal of Medicine 2014;50(3):227-34. | Exclude on population: healthy controls |
| Rammohan, A., Jothimani, D., Sreedurgalakshmi, K., Farouk, M., Lakshmi, S., Vasanthakumar, G., et al. Validation of a Multiplex Hypermethylation-based Blood Test to Detect Hepatocellular Carcinoma: A Prospective Case-control Study. Journal of Clinical and Experimental Hepatology 2025;15(5):102578. | Does not meet full eligibility criteria: includes people without cirrhosis |
| Rashid, M. M., Varghese, R. S., Ding, Y. Biomarker Discovery for Hepatocellular Carcinoma in Patients with Liver Cirrhosis Using Untargeted Metabolomics and Lipidomics Studies. Metabolites 2023;13(10):1047. | No 2x2 data |
| Reddy, S. T., Thai, N. L., Doyle, M., Oliva, J., Tom, K. B., Maheshwary, R., et al. Is the detection of hepatocellular carcinoma in liver transplantation candidates impaired using a dedicated CMR stress test as a 'one-stopshop'? a pathological correlation study. Journal of Cardiovascular Magnetic Resonance 2014;16(SUPPL. 1):n. pag.. | Exclude on index test |
| Refai, N. S., Louka, M. L., Halim, H. Y. Long non-coding RNAs (CASC2 and TUG1) in hepatocellular carcinoma: Clinical significance. Journal of Gene Medicine 2019;21(9):e3112. | Exclude on population: not cirrhosis |
| Reichl, Patrick, Fang, Meng, Starlinger, Patrick, Staufer, Katharina, Nenutil, Rudolf, Muller, Petr, et al. Multicenter analysis of soluble Axl reveals diagnostic value for very early stage hepatocellular carcinoma. International journal of cancer 2015;137(2):385-94. | Does not meet full eligibility criteria: includes people without cirrhosis |
| Reis, Henning, Putter, Carolin, Megger, Dominik A., Bracht, Thilo, Weber, Frank, Hoffmann, Andreas- C., et al. A structured proteomic approach identifies 14-3-3Sigma as a novel and reliable protein biomarker in panel based differential diagnostics of liver tumors. Biochimica et biophysica acta 2015;1854(6):641-50. | Exclude on index test |
| Ren, Biqiong, Zou, Guoying, Xu, Fei, Huang, Yiran, Xu, Guofeng, He, Junyu, et al. Serum levels of anti-sperm-associated antigen 9 antibody are elevated in patients with hepatocellular carcinoma. Oncology letters 2017;14(6):7608-14. | Exclude on population: not cirrhosis |
| Ren, Fen-Yu & Piao, Xi-Xu. Efficacy of ultrasonography and alpha-fetoprotein on early detection of hepatocellular carcinoma. World journal of gastroenterology 2006;12(29):4656-59. | Exclude on study design: not a 1- or 2-gate test accuracy study |
| Ren, S. H. Studies on serum alpha-fetoprotein levels in cases of hepatocellular carcinoma with negative or positive HBsAg. Chinese Journal of Clinical Oncology 1994;21(7):505-07. | Does not meet full eligibility criteria: pre-2005 |
| Ren, Y., Yang, L., Li, M., Wang, J., Yan, H., Ma, N., et al. 4210 Da and 1866 Da polypeptides as potential biomarkers of liver disease progression in hepatitis B virus patients. Scientific reports 2021;11(1):16982. | Does not meet full eligibility criteria: includes people without cirrhosis |
| Ressom, Habtom W., Varghese, Rency S., Goldman, Lenka, An, Yanming, Loffredo, Christopher A., Abdel-Hamid, Mohamed, et al. Analysis of MALDI-TOF mass spectrometry data for discovery of peptide and glycan biomarkers of hepatocellular carcinoma. Journal of proteome research 2008;7(2):603-10. | Exclude on index test |
| Ricco, Gabriele, Cavallone, Daniela, Cosma, Chiara, Caviglia, Gian Paolo, Oliveri, Filippo, Biasiolo, Alessandra, et al. Impact of etiology of chronic liver disease on hepatocellular carcinoma biomarkers. Cancer biomarkers : section A of Disease markers 2018;21(3):603-12. | Duplicate (including Cochrane includes) |

| Reference | Reason for exclusion |
|---|---|
| Ricco, Gabriele, Cosma, Chiara, Bedogni, Giorgio, Biasiolo, Alessandra, Guarino, Maria, Pontisso, Patrizia, et al. Modeling the time-related fluctuations of AFP and PIVKA-II serum levels in patients with cirrhosis undergoing surveillance for hepatocellular carcinoma. Cancer biomarkers : section A of Disease markers 2020;29(2):189-96. | Exclude on study design: not a 1- or 2-gate test accuracy study |
| Riener, M. O., Stenner, F., Liewen, H., Hellerbrand, C., Bahra, M. Alpha-fetoprotein and serum Golgi phosphoprotein 2 are equally discriminative in detecting early hepatocellular carcinomas. Hepatology 2009;50(1):326. | Exclude on study design: not a 1- or 2-gate test accuracy study |
| Riener, M. O., Stenner, F., Liewen, H., Soll, C., Bretenstein, S., Pestalozzi, B. C., et al. Golgi phosphoprotein 2 (GOLPH2) expression in liver tumors and its value as a serum marker in hepatocellular carcinomas. Hepatology 2009;49(5):1602-09. | Exclude on population: clinical signs and symptoms of HCC |
| Riener, Marc-Oliver, Stenner, Frank, Liewen, Heike, Soll, Christopher, Breitenstein, Stefan, Pestalozzi, Bernhard Cornelius, et al. Golgi phosphoprotein 2 (GOLPH2) expression in liver tumors and its value as a serum marker in hepatocellular carcinomas. Hepatology (Baltimore, Md.) 2009;49(5):1602-09. | Exclude on population: not cirrhosis |
| Rizk, Nehal I., Sallam, Al-Aliaa M., El-Ansary, Amira R. HMGB1 and SEPP1 as predictors of hepatocellular carcinoma in patients with viral C hepatitis: Effect of DAAs. Clinical biochemistry 2019;70():8-13. | Exclude on population: not cirrhosis |
| Rizvi, S., Camci, C., Yong, Y., Parker, G., Shrago, S., Stokes, K., et al. Is post-Lipiodol CT better than i.v. contrast CT scan for early detection of HCC? A single liver transplant center experience. Transplantation proceedings 2006;38(9):2993-95. | Exclude on population: clinical signs and symptoms of HCC |
| Rode, A., Bancel, B., Douek, P., Chevallier, M., Vilgrain, V., Picaud, G., et al. Small nodule detection in cirrhotic livers: evaluation with US, spiral CT, and MRI and correlation with pathologic examination of explanted liver. Journal of computer assisted tomography 2001;25(3):327-36. | Exclude on population: clinical signs and symptoms of HCC |
| Roget, L., Coppere, H., Richard, P., Barthelemy, C., Veyret, C. Value of real time ultrasonography for early detection of hepatocellular carcinoma in patients hospitalized for cirrhosis. Gastroenterologie Clinique et Biologique 1988;12(11):827-32. | Does not meet full eligibility criteria: pre-2005 |
| Rong, Dailin, He, Bingjun, Tang, Wenjie, Xie, Sidong, Kuang, Sichi, Grazioli, Luigi, et al. Comparison of Gadobenate-Enhanced MRI and Gadoxetate-Enhanced MRI for Hepatocellular Carcinoma Detection Using LI-RADS Version 2018: A Prospective Intraindividual Randomized Study. AJR. American journal of roentgenology 2022;218(4):687-98. | Does not meet full eligibility criteria: includes people without cirrhosis |
| Ronzoni, Annalisa, Artioli, Diana, Scardina, Rosa, Battistig, Luca, Minola, Ernesto, Sironi, Sandro. Role of MDCT in the diagnosis of hepatocellular carcinoma in patients with cirrhosis undergoing orthotopic liver transplantation. AJR. American journal of roentgenology 2007;189(4):792-98. | Exclude on population: HCC participants not treatment-naive |
| Rosenkrantz, Andrew B., Campbell, Naomi, Wehrli, Natasha, Triolo, Michael J. New OPTN/UNOS classification system for nodules in cirrhotic livers detected with MR imaging: effect on hepatocellular carcinoma detection and transplantation allocation. Radiology 2015;274(2):426-33. | Exclude on study design: not a 1- or 2-gate test accuracy study |
| Roshdy, F., Farag, M. M. S., El-Ahwany, E., Mahmode, O., Mousa, A. A., El Talkawy, M. Long non-coding RNA HOTAIR and HOTTIP as potential biomarkers for hepatitis C virus genotype 4-induced hepatocellular carcinoma. Egyptian Journal of Medical Human Genetics 2020;21(1):7. | Does not meet full eligibility criteria: includes people without cirrhosis |
| Rui, Tao, Wang, Kai, Xiang, Aizhai, Guo, Jufeng, Tang, Ning, Jin, Xin, et al. Serum Exosome-Derived piRNAs Could Be Promising Biomarkers for HCC Diagnosis. International journal of nanomedicine 2023;18():1989-2001. | Exclude on population: not cirrhosis |
| Ryu, M. R. & Kang, E. S. Performance evaluation of serum PIVKA-II measurement using HISCL-5000 and a method comparison of HISCL-5000, LUMIPULSE G1200, and ARCHITECT i2000. Journal of Clinical Laboratory Analysis 2019;33(6):e22921. | Exclude on population: not cirrhosis |
| Saad, Yasmin, El-Serafy, Magdy, Eldin, Mona S., Abdellatif, Zeinab, Khatab, Hany, Elbaz, Tamer. New genetic markers for diagnosis of hepatitis C related hepatocellular carcinoma in Egyptian patients. Journal of gastrointestinal and liver diseases : JGLD 2013;22(4):419-25. | Exclude on population: not cirrhosis |

| Reference | Reason for exclusion |
|---|---|
| Saada, J, Bhattacharya, S, Dhillon, A P, Dick, R, Burroughs, A K, Rolles, K. Detection of small hepatocellular carcinomas in cirrhotic livers using iodised oil computed tomography. Gut 1997;41(3):404-7. | Does not meet full eligibility criteria: pre-2005 |
| Saada, J., Bhattacharya, S., Dhillon, A. P., Dick, R., Burroughs, A. K., Rolles, K. Detection of small hepatocellular carcinomas in cirrhotic livers using iodised oil computed tomography. Gut 1997;41(3):404-07. | Does not meet full eligibility criteria: pre-2005 |
| Sabry, H. S., Tayel, S. I., Enar, M. E. Differential expression of long noncoding RNA in hepatocellular carcinoma on top of chronic HCV and HBV infections. Clinical and Experimental Hepatology 2021;7(4):337-50. | Does not meet full eligibility criteria: includes people without cirrhosis |
| Sabry, Hany S., Tayel, Safaa I., Enar, Mohamed E. Differential expression of long noncoding RNA in hepatocellular carcinoma on top of chronic HCV and HBV infections. Clinical and experimental hepatology 2021;7(4):337-50. | Does not meet full eligibility criteria: includes people without cirrhosis |
| Sacchetti, L., Castaldo, G., Cimino, L., Budillon, G. Diagnostic efficiency in discriminating liver malignancies from cirrhosis by serum gamma-glutamyltransferase isoforms. Clinica Chimica Acta 1988;177(2):167-72. | Exclude on index test |
| Sachan, Anurag & Kushwah, Saraswati. GALAD Score for HCC Screening and Surveillance. Clinical gastroenterology and hepatology : the official clinical practice journal of the American Gastroenterological Association 2023;21(2):556-57. | Exclude on study design: not a 1- or 2-gate test accuracy study |
| Sadeghi, M., Lahdou, I., Oweira, H., Daniel, V., Terness, P., Schmidt, J., et al. Serum levels of chemokines CCL4 and CCL5 in cirrhotic patients indicate the presence of hepatocellular carcinoma. British journal of cancer 2015;113(5):756-62. | Duplicate (including Cochrane includes) |
| Sadik N.A., Ahmed N.R., Mohamed M.F. Serum vascular endothelial growth factor in patients with hepatocellular carcinoma and its validity as a tumor biomarker. Open Biomarkers Journal 2019;9(1):84-94. | Does not meet full eligibility criteria: includes people without cirrhosis |
| Sadik, N. A., Ahmed, N. R., Mohamed, M. F. Serum vascular endothelial growth factor in patients with hepatocellular carcinoma and its validity as a tumor biomarker. Open Biomarkers Journal 2019;9(1):84-94. | Duplicate (including Cochrane includes) |
| Sahin, Enes, Tatar, Ozan Can, Ulutas, Mehmet Esref, Guler, Sertac Ata, Simsek, Turgay, Turgay, Nihat Zafer. Diagnostic Performance of Deep Learning Applications in Hepatocellular Carcinoma Detection Using Computed Tomography Imaging. The Turkish journal of gastroenterology : the official journal of Turkish Society of Gastroenterology 2024;36(2):124â€“130. | Exclude on population: clinical signs and symptoms of HCC |
| Sahin, T., Serin, A., Emek, E., Bozkurt, B., Arikan, B. T. Effectiveness of Noninvasive Fibrosis Markers for the Prediction of Hepatocellular Carcinoma in Chronic Hepatitis B and Chronic Hepatitis B+D Induced Cirrhosis. Transplantation Proceedings 2019;51(7):2397-4002. | No 2x2 data |
| Sai, Wenli, Wang, Li, Zheng, Wenjie, Yang, Junling, Pan, Liuhong, Cai, Yin, et al. Abnormal Expression of Golgi Protein 73 in Clinical Values and Their Role in HBV-Related Hepatocellular Carcinoma Diagnosis and Prognosis. Hepatitis monthly 2015;15(12):e32918. | Does not meet full eligibility criteria: includes people without cirrhosis |
| Said, Ebada M., Salem, Abdelhamed A., Shousha, Hend I., Ahmad, Enas S., Alazzouny, Mahmoud A., Ahmed, Inas A., Elfeky, Hala M. RECK gene polymorphisms in hepatitis B-related hepatocellular carcinoma: A case-control study. Arab journal of gastroenterology : the official publication of the Pan-Arab Association of Gastroenterology 2022;23(3):201-05. | Exclude on population: not cirrhosis |
| Said, Ebada M., Salem, Abdelhamed A., Shousha, Hend I., Ahmad, Enas S., Alazzouny, Mahmoud A., Ahmed, Inas A., Elfeky, Hala M. RECK gene polymorphisms in hepatitis B-related hepatocellular carcinoma: A case-control study. Arab journal of gastroenterology : the official publication of the Pan-Arab Association of Gastroenterology 2022;():n. pag.. | Exclude on population: not cirrhosis |
| Saito, Masaya, Yano, Yoshihiko, Hirano, Hirotaka, Momose, Kenji, Mouri, Kentaro, Hishimoto, Akitoyo, Yoshida, Masaru. The serum level of NX-DCP-R, but not DCP, is not increased in alcoholic liver disease without hepatocellular carcinoma. Cancer biomarkers : section A of Disease markers 2016;16(1):171-80. | Exclude on population: not cirrhosis |
| Saito, S., Ikeda, K., Koida, I., Tsubota, A., Arase, Y., Chayama, K., et al. The diagnosis of hepatocellular carcinoma determined by pattern of AFP bands | Does not meet full eligibility criteria: pre-2005 |

| Reference | Reason for exclusion |
|---|---|
| separated by Con A affinity electrophoresis. Japanese Journal of Gastroenterology 1994;91(8):1301-08. | |
| Saito, Yumi, Oba, Nobuyuki, Nishinakagawa, Syuta, Mizuguchi, Yasuhiro, Kojima, Tatsuya, Nomura, Kazuhiro. Identification of beta2-microgloblin as a candidate for early diagnosis of imaging-invisible hepatocellular carcinoma in patient with liver cirrhosis. Oncology reports 2010;23(5):1325-30. | Exclude on index test |
| Saitoh, S., Ikeda, K., Koida, I., Tsubota, A., Arase, Y., Chayama, K. Serum des-gamma-carboxyprothrombin concentration determined by the avidin-biotin complex method in small hepatocellular carcinomas. Cancer 1994;74(11):2918-23. | Does not meet full eligibility criteria: includes people without cirrhosis |
| Saitta, Carlo, Raffa, Giuseppina, Alibrandi, Angela, Brancatelli, Santa, Lombardo, Daniele, Tripodi, Gianluca, Raimondo, Giovanni. PIVKA-II is a useful tool for diagnostic characterization of ultrasound-detected liver nodules in cirrhotic patients. Medicine 2017;96(26):e7266. | Exclude on population: clinical signs and symptoms of HCC |
| Sajid, M. S., Ding, Y., Varghese, R. S., Kroemer, A. Unveiling Endogenous Serum Peptides as Potential Biomarkers for Hepatocellular Carcinoma in Patients with Liver Cirrhosis. Journal of Proteome Research 2024;23(9):3974 " 3983. | No 2x2 data |
| Sajid, M. S., Varghese, R. S., Kroemer, A. Low-Abundance Serum Protein Biomarker Candidates for HCC in Patients with Liver Cirrhosis. Journal of Proteome Research 2025;24(7):3656 " 3665. | No 2x2 data |
| Sakamoto, M. p53 in sera for the early detection of hepatocellular carcinoma. Journal of Gastroenterology 2001;36(12):865-66. | Exclude on study design: not a 1- or 2-gate test accuracy study |
| Sakr, Mohammad, Abdelhakam, Sara M., Dabbous, Hany, Hamed, Amr, Hefny, Zeinab, Abdelmoaty, Waleed, et al. Characteristics of hepatocellular carcinoma in Egyptian patients with primary Budd-Chiari syndrome. Liver international : official journal of the International Association for the Study of the Liver 2017;37(3):415-22. | Does not meet full eligibility criteria: includes people without cirrhosis |
| Salama, Hosni M., Abdel-Haleem, Hanan, Taha, Fatma M., Abdel-Azeez, Rasha A., Marzaban, Raghda N., El-Nahaas, Saeed M. Plasma proteosome level as a potential marker for hepatocellular carcinoma. Arab journal of gastroenterology : the official publication of the Pan-Arab Association of Gastroenterology 2013;14(3):87-93. | Does not meet full eligibility criteria: includes people without cirrhosis |
| Salama, M. M., Nomir, M. M., Fahmi, M. W., El-Gayar, A. M. Potential role of microfibrillar-associated protein 4, fibrotic indices and oxidative stress in hepatocellular carcinoma. Scientia Pharmaceutica 2018;86(4): 44;. | Exclude on index test |
| Salem, Mona, Atti, Sahar Abdel, Raziky, Maisa El, Darweesh, Samar Kamal. Clinical Significance of Plasma Osteopontin Level as a Biomarker of Hepatocellular Carcinoma. Gastroenterology research 2013;6(5):191-99. | Does not meet full eligibility criteria: includes people without cirrhosis |
| Salem, Mona, Atti, Sahar Abdel, Raziky, Maisa El, Darweesh, Samar Kamal. Clinical Significance of Plasma Osteopontin Level as a Biomarker of Hepatocellular Carcinoma. Gastroenterology research 2013;6(5):191-99. | Exclude on population: healthy controls |
| Salerno, F., Restelli, B., Incerti, P., Annoni, G., Capozza, L., Badalamenti, S., et al. Utility of ascitic fluid analysis in patients with malignancy-related ascites. Scandinavian journal of gastroenterology 1990;25(3):251-56. | Does not meet full eligibility criteria: pre-2005 |
| Salman, T., El-Ahmady, O., Hamza, S., Sadek, S. Biological markers sensitivity in patients with chronic liver diseases and hepatocellular carcinoma. Cancer Molecular Biology 1995;2(6):683-89. | Does not meet full eligibility criteria: pre-2005 |
| Samal, J., Gautam, P., Gupta, A., Rooge, S., Bihari, C. An exploratory study to find out circulating miRNAs in Hepatitis C virus associated Hepatocellular carcinoma. Journal of Clinical and Experimental Hepatology 2022;12(Supplement 2):S90-91. | Exclude on population: not cirrhosis |
| Samir, A., Abdeldaim, A., Mohammed, A., Ali, A., Alorabi, M., Hussein, M. M., et al. Analysis of four long non-coding RNAs for hepatocellular carcinoma screening and prognosis by the aid of machine learning techniques. Scientific reports 2024;14(1):29582. | Does not meet full eligibility criteria: includes people without cirrhosis |

| Reference | Reason for exclusion |
|---|---|
| Samuell, Ereny, Abdel-Aziz, Abdel-Aziz F., Shehata, Ahmed S., Abdel-Wahab, Mohamed. The Combined Use of Dickkopf-1 and Soluble Axl Improves Hepatocellular Carcinoma Diagnostic Efficacy in Hepatitis C Patients. Asian Pacific journal of cancer prevention : APJCP 2024;25(6):2185â€“2191. | Does not meet full eligibility criteria: includes people without cirrhosis |
| Sanchez, J. I., Fontillas, A. C., Kwan, S. Y., Sanchez, C. I., Calderone, T. L., Lee, J. L., et al. Metabolomics biomarkers of hepatocellular carcinoma in a prospective cohort of patients with cirrhosis. JHEP Reports 2024;6(8):101119. | No 2x2 data |
| Sanchez, Jessica I., Jiao, Jingjing, Kwan, Suet-Ying, Veillon, Lucas, Warmoes, Marc O., Tan, Lin, et al. Lipidomic Profiles of Plasma Exosomes Identify Candidate Biomarkers for Early Detection of Hepatocellular Carcinoma in Patients with Cirrhosis. Cancer prevention research (Philadelphia, Pa.) 2021;14(10):955-62. | Exclude on study design: not a 1- or 2-gate test accuracy study |
| Sangiovanni, Angelo, Del Ninno, Ersilio, Fasani, Pierangelo, De Fazio, Cristina, Ronchi, Guido, Romeo, Raffaella, et al. Increased survival of cirrhotic patients with a hepatocellular carcinoma detected during surveillance. Gastroenterology 2004;126(4):1005-14. | Exclude on study design: not a 1- or 2-gate test accuracy study |
| Sartorius, Kurt, Sartorius, Benn, Winkler, Cheryl, Chuturgoon, Anil, Shen, Tsai-Wei, Zhao, Yongmei. Serum microRNA Profiles and Pathways in Hepatitis B-Associated Hepatocellular Carcinoma: A South African Study. International journal of molecular sciences 2024;25(2):n. pag.. | No 2x2 data |
| Sarvari, J., Mojtahedi, Z., Taghavi, S. A. R., Kuramitsu, Y., Shamsi Shahrabadi, M., Ghaderi, A. Differentially expressed proteins in chronic active hepatitis, cirrhosis, and HCC related to HCV infection in comparison with HBV infection: A proteomics study. Hepatitis Monthly 2013;13(7):e8351. | Exclude on index test |
| Sarwar, Shahid & Khan, Anwaar Ahmad. Validity of alpha fetoprotein for diagnosis of hepatocellular carcinoma in cirrhosis. Journal of the College of Physicians and Surgeons--Pakistan : JCPSP 2014;24(1):18-22. | Does not meet full eligibility criteria: includes people without cirrhosis |
| Sarwar, Shahid & Khan, Anwaar Ahmad. Validity of alpha fetoprotein for diagnosis of hepatocellular carcinoma in cirrhosis. Journal of the College of Physicians and Surgeons--Pakistan : JCPSP 2014;24(1):18-22. | Does not meet full eligibility criteria: includes people without cirrhosis |
| Sassa, T, Kumada, T, Nakano, S. Clinical utility of simultaneous measurement of serum high-sensitivity des-gamma-carboxy prothrombin and Lens culinaris agglutinin A-reactive alpha-fetoprotein in patients with small hepatocellular carcinoma. European journal of gastroenterology & hepatology 1999;11(12):1387-92. | Does not meet full eligibility criteria: includes people without cirrhosis |
| Sassa, T., Kumada, T., Nakano, S. Clinical utility of simultaneous measurement of serum high-sensitivity des-gamma-carboxy prothrombin and Lens culinaris agglutinin A-reactive alpha-fetoprotein in patients with small hepatocellular carcinoma. European journal of gastroenterology & hepatology 1999;11(12):1387-92. | Does not meet full eligibility criteria: pre-2005 |
| Sato, Y, Nakata, K, Kato, Y, Shima, M, Ishii, N, Koji, T, et al. Early recognition of hepatocellular carcinoma based on altered profiles of alpha-fetoprotein. The New England journal of medicine 1993;328(25):1802-6. | Does not meet full eligibility criteria: pre-2005 |
| Sato, Y., Nakata, K., Kato, Y., Shima, M., Ishii, N., Koji, T., et al. Early recognition of hepatocellular carcinoma based on altered profiles of alpha-fetoprotein. The New England journal of medicine 1993;328(25):1802-06. | Does not meet full eligibility criteria: pre-2005 |
| Sayed, G. I., Solyman, M., El Gedawy, G., Moemen, Y. S., Aboul-Ella, H. Circulating miRNA's biomarkers for early detection of hepatocellular carcinoma in Egyptian patients based on machine learning algorithms. Scientific reports 2024;14(1):4989. | Exclude on index test |
| Sayeed, A., Dalvano, B. E., Kaplan, D. E., Viswanathan, U., Kulp, J., Janneh, A. H., et al. Research Paper Profiling the circulating mRNA transcriptome in human liver disease. Oncotarget 2020;11(23):2226-42. | No 2x2 data |
| Schoenberger, H., Chong, N., Fetzer, D. T., Rich, N. E., Arroyo, A., Yokoo, T., et al. ULTRASOUND QUALITY FOR HEPATOCELLULAR CARCINOMA IN PATIENTS WITH CIRRHOSIS. Gastroenterology 2021;160(6 Supplement):S-805. | CONFERENCE ABSTRACT |
| Scholz, Arne, Rehm, Vanessa Annina, Rieke, Svenja, Derkow, Katja, Schulz, Petra, Neumann, Konrad, et al. Angiopoietin-2 serum levels are elevated in | Does not meet full eligibility criteria: includes people without cirrhosis |

| Reference | Reason for exclusion |
|---|---|
| patients with liver cirrhosis and hepatocellular carcinoma. The American journal of gastroenterology 2007;102(11):2471-81. | |
| Schotten, C., Ostertag, B., Sowa, J. P., Manka, P., Bechmann, L. P., Hilgard, G., et al. Galad score detects early-stage hepatocellular carcinoma in a european cohort of chronic hepatitis b and c patients. Pharmaceuticals 2021;14(8):735. | Exclude on population: not cirrhosis |
| Schulze, K., Gasch, C., Staufer, K., Nashan, B., Lohse, A. W., Pantel, K., Riethdorf, S. Presence of EpCAM-positive circulating tumor cells as biomarker for systemic disease strongly correlates to survival in patients with hepatocellular carcinoma. International Journal of Cancer 2013;133(9):2165-71. | Exclude on population: not cirrhosis |
| Schulze, K., Rose, T. D., Adlung, L., Peschka, M., Frundt, T. W., Labgaa, I., et al. Metabolomic liquid biopsy dynamics predict early onset of human hepatocarcinogenesis. Zeitschrift fur Gastroenterologie 2023;61(8):e492. | CONFERENCE ABSTRACT |
| Schulze, K., Rose, T. D., Adlung, L., Peschka, M., Pagani, F., Gorgulho, J., et al. Metabolomic liquid biopsy dynamics predict early-stage HCC and actionable candidates of human hepatocarcinogenesis. JHEP Reports 2025;7(5):101340. | Exclude on index test |
| Schwegler, E. E., Cazares, L., Steel, L. F., Adam, B. L., Johnson, D. A., Semmes, O. J., et al. SELDI-TOF MS profiling of serum for detection of the progression of chronic hepatitis C to hepatocellular carcinoma. Hepatology 2005;41(3):634-42. | Exclude on index test |
| Se, H. K., Lee, J. M., Young, J. K., Jin, Y. C., Gi, H. K., Ho, Y. L. Detection of hepatocellular carcinoma on CT in liver transplant candidates: Comparison of PACS tile and multisynchronized stack modes. American Journal of Roentgenology 2007;188(5):1337-42. | Exclude on population: clinical signs and symptoms of HCC |
| Secil, Mustafa, Obuz, Funda, Altay, Canan, Gencel, Omur, Igci, Enis, Sagol, Ozgul. The role of dynamic subtraction MRI in detection of hepatocellular carcinoma. Diagnostic and interventional radiology (Ankara, Turkey) 2008;14(4):200-04. | Exclude on population: clinical signs and symptoms of HCC |
| Seif El Dahan, K., Yokoo, T., Mendiratta-Lala, M., Fetzer, D., Davenport, M., Daher, D., et al. Exam quality of ultrasound and dynamic contrast-enhanced abbreviated MRI and impact on early-stage HCC detection. Abdominal Radiology 2025;50(5):2097 “ 2109. | Secondary publication of included study |
| Seif, A. A., Aly, H. H., Elzoghby, D. M., Elbreedy, A. M. Aberrant p16 methylation as an early diagnostic marker in blood of hepatocellular carcinoma patients. Egyptian Journal of Medical Human Genetics 2019;20(1):27. | Does not meet full eligibility criteria: includes people without cirrhosis |
| Seimiya, Masanori, Tomonaga, Takeshi, Matsushita, Kazuyuki, Sunaga, Masahiko, Oh-Ishi, Masamichi, Kodera, Yoshio, et al. Identification of novel immunohistochemical tumor markers for primary hepatocellular carcinoma; clathrin heavy chain and formiminotransferase cyclodeaminase. Hepatology (Baltimore, Md.) 2008;48(2):519-30. | Exclude on population: tissue samples |
| Sekiguchi, R., Kuwajima, A., Nagamoto, M., Ohno, H. Hepatocellular carcinoma: the diagnostic difficulties of ultrasonography and analysis of risk factors in MHTS. Journal of medical systems 1993;17(3-4):133-37. | Exclude on population: clinical signs and symptoms of HCC |
| Sekine, C., Aoyagi, Y., Suzuki, Y. The reactivity of alpha-1-antitrypsin with Lens culinaris agglutinin and its usefulness in the diagnosis of neoplastic diseases of the liver. British journal of cancer 1987;56(3):371-75. | Exclude on index test |
| Seo, Seung In, Kim, Hyoung Su, Kim, Won Jin, Shin, Woon Geon, Kim, Doo Jin, Kim, Kyung Ho, et al. Diagnostic value of PIVKA-II and alpha-fetoprotein in hepatitis B virus-associated hepatocellular carcinoma. World journal of gastroenterology 2015;21(13):3928-35. | Duplicate (including Cochrane includes) |
| Seo, Seung In, Kim, Hyoung Su, Kim, Won Jin, Shin, Woon Geon, Kim, Doo Jin, Kim, Kyung Ho, et al. Diagnostic value of PIVKA-II and alpha-fetoprotein in hepatitis B virus-associated hepatocellular carcinoma. World journal of gastroenterology 2015;21(13):3928-35. | Does not meet full eligibility criteria: includes people without cirrhosis |
| Serilmez, M., Akyuz, F., Ormeci Ciftcibasi, A., Karabulut, S., Kaymakoglu, S., Yasasever, V. Serum glypican-3, vascular endothelial growth factor, and interleukin-6 levels in hepatocellular carcinoma. Turk Onkoloji Dergisi 2018;33(1):18-27. | Exclude on index test |

| Reference | Reason for exclusion |
|---|---|
| Seung, H. C., Lee, J. M., Yu, N. C., Suh, K. S., Jang, J. J., Se, H. K. Hepatocellular carcinoma in liver transplantation candidates: Detection with gadobenate dimeglumine-enhanced MRI. American Journal of Roentgenology 2008;191(2):529-36. | Secondary publication of included study |
| Sevco, T. J., Masch, W. R., Maturen, K. E., Mendiratta-Lala, M., Wasnik, A. P. Ultrasound (US) LI-RADS: Outcomes of category US-3 observations. American Journal of Roentgenology 2021;217(3):644-50. | No 2x2 data |
| Shaalan, U. F. & El-Halafawy, K. A. Molecular approach for early diagnosis of hepatocellular carcinoma in egyptian patients by Alpha fetoprotein (AFP) and Vascular Endothelial Growth Factor (VEGF). Pakistan Journal of Biotechnology 2010;7(1-2):67-74. | Exclude on population: healthy controls |
| Shaheen, Karim Y A, Abdel-Mageed, Abeer I, Safwat, Eslam. The value of serum midkine level in diagnosis of hepatocellular carcinoma. International journal of hepatology 2015;2015(101564967):146389. | Does not meet full eligibility criteria: includes people without cirrhosis |
| Shaheen, Karim Y. A., Abdel-Mageed, Abeer I., Safwat, Eslam. The value of serum midkine level in diagnosis of hepatocellular carcinoma. International journal of hepatology 2015;2015():146389. | Duplicate (including Cochrane includes) |
| Shaheen, Noha Mohamed Hosni, Zayed, Naglaa, Riad, Nermine Magdi, Tamim, Hend H, Shahin, Rasha Mohamed Hosny, Labib, Dalia A, et al. Role of circulating miR-182 and miR-150 as biomarkers for cirrhosis and hepatocellular carcinoma post HCV infection in Egyptian patients. Virus research 2018;255(x98, 8410979):77-84. | Exclude on population: not cirrhosis |
| Shaheen, Noha Mohamed Hosni, Zayed, Naglaa, Riad, Nermine Magdi, Tamim, Hend H., Shahin, Rasha Mohamed Hosny, Labib, Dalia A., et al. Role of circulating miR-182 and miR-150 as biomarkers for cirrhosis and hepatocellular carcinoma post HCV infection in Egyptian patients. Virus research 2018;255():77-84. | Duplicate (including Cochrane includes) |
| Shaikh, Jamil, Stoddard, Paul B., Levine, Evan G., Roh, Albert T., Saranathan, Manojkumar, Chang, Stephanie T., et al. View-Sharing Artifact Reduction With Retrospective Compressed Sensing Reconstruction in the Context of Contrast-Enhanced Liver MRI for Hepatocellular Carcinoma (HCC) Screening. Journal of magnetic resonance imaging : JMRI 2019;49(4):984-93. | Exclude on study design: not a 1- or 2-gate test accuracy study |
| Shaker, Mohamed K., Abdel Fattah, Hanzada I., Sabbour, Ghada S., Montasser, Iman F., Abdelhakam, Sara M., El Hadidy, Eman, Yousry, Rehab. Annexin A2 as a biomarker for hepatocellular carcinoma in Egyptian patients. World journal of hepatology 2017;9(9):469-76. | Exclude on population: not cirrhosis |
| Shaker, O. G., Khairy, A. M., Ali, R. M. M. miRNA-141 and its target long non-coding RNA HOTAIR as diagnostic marker in hepatocellular carcinoma on top of hepatitis C virus. Gene Reports 2020;21():100807. | Exclude on population: not cirrhosis |
| Shaker, Olfat, Alhelf, Maha, Morcos, George. miRNA-101-1 and miRNA-221 expressions and their polymorphisms as biomarkers for early diagnosis of hepatocellular carcinoma. Infection, genetics and evolution : journal of molecular epidemiology and evolutionary genetics in infectious diseases 2017;51():173-81. | Exclude on population: not cirrhosis |
| Shaker, Olfat, Mahfouz, Hala, Salama, Ahmad. Long Non-Coding HULC and miRNA-372 as Diagnostic Biomarkers in Hepatocellular Carcinoma. Reports of biochemistry & molecular biology 2020;9(2):230-40. | Exclude on population: not cirrhosis |
| Shalably, N. M., Badawi, R., Hawash, N., Abd-Elsalam, S., Elkhalawany, W., Ei Hameed, A. A. Evaluation of fucosylated haptoglobin as a diagnostic biomarker for hepatocellular carcinoma in egypt. Open Biomarkers Journal 2019;9(1):31-37. | Exclude on population: healthy controls |
| Shang, Shuxin, Sun, Lu, Li, Wei, Qin, Xue, Zhang, Shu. Rapid diagnosis of hepatocellular carcinoma using a haptoglobin ELISA assay based on AAL-coated magnetic beads. Discovery medicine 2016;22(120):97-104. | Exclude on population: healthy controls |
| Shang, Sufen, Plymoth, Amelie, Ge, Shaokui, Feng, Ziding, Rosen, Hugo R, Sangrajrang, Suleeporn, et al. Identification of osteopontin as a novel marker for early hepatocellular carcinoma. Hepatology (Baltimore, Md.) 2012;55(2):483-90. | Does not meet full eligibility criteria: includes people without cirrhosis |
| Shang, Sufen, Plymoth, Amelie, Ge, Shaokui, Feng, Ziding, Rosen, Hugo R., Sangrajrang, Suleeporn, et al. Identification of osteopontin as a novel marker | Duplicate (including Cochrane includes) |

| Reference | Reason for exclusion |
| --- | --- |
| for early hepatocellular carcinoma. Hepatology (Baltimore, Md.) 2012;55(2):483-90. | |
| Shao, Qing, Ren, Pengfei, Li, Yang, Peng, Bo, Dai, Liping, Lei, Ningjing, et al. Autoantibodies against glucose-regulated protein 78 as serological diagnostic biomarkers in hepatocellular carcinoma. International journal of oncology 2012;41(3):1061-67. | No 2x2 data |
| Shapiro, R. S., Katz, R., Mendelson, D. S., Halton, K. P., Schwartz, M. E. Detection of hepatocellular carcinoma in cirrhotic patients: sensitivity of CT and ultrasonography. Journal of ultrasound in medicine : official journal of the American Institute of Ultrasound in Medicine 1996;15(7):497-94. | Exclude on population: clinical signs and symptoms of HCC |
| Sharafeldin, M. A., Suef, R. A., Mousa, A. A., Ziada, D. H. Serum miRNA-101 expression signature as non-invasive diagnostic biomarker for Hepatitis C virus-associated hepatocellular carcinoma in Egyptian patients. Scientific reports 2025;15(1):645. | Does not meet full eligibility criteria: includes people without cirrhosis |
| Sharafeldin, M. A., Suef, R. A., Mousa, A. A., Ziadah, D. H. Serum interleukin-10 and alpha-fetoprotein: A combined diagnostic approach for hepatocellular carcinoma in Egyptians with HCV. Pathology Research and Practice 2024;258():155327. | Does not meet full eligibility criteria: includes people without cirrhosis |
| Shariff, Mohamed I F, Kim, Jin U, Ladep, Nimzing G, Crossey, Mary M E, Koomson, Larry K, Zabron, Abigail, et al. Urinary Metabotyping of Hepatocellular Carcinoma in a UK Cohort Using Proton Nuclear Magnetic Resonance Spectroscopy. Journal of clinical and experimental hepatology 2016;6(3):186-94. | Does not meet full eligibility criteria: includes people without cirrhosis |
| Shariff, Mohamed I F, Ladep, Nimzing G, Cox, I Jane, Williams, Horace R T, Okeke, Edith, Malu, Abraham, et al. Characterization of urinary biomarkers of hepatocellular carcinoma using magnetic resonance spectroscopy in a Nigerian population. Journal of proteome research 2010;9(2):1096-03. | Does not meet full eligibility criteria: includes people without cirrhosis |
| Shariff, Mohamed I. F., Kim, Jin U., Ladep, Nimzing G., Crossey, Mary M. E., Koomson, Larry K., Zabron, Abigail, et al. Urinary Metabotyping of Hepatocellular Carcinoma in a UK Cohort Using Proton Nuclear Magnetic Resonance Spectroscopy. Journal of clinical and experimental hepatology 2016;6(3):186-94. | Does not meet full eligibility criteria: includes people without cirrhosis |
| Sharma, B. K., Srinivasan, R., Kapil, S., Singla, B., Saini, N., Chawla, Y. K., et al. Serum levels of angiogenic and anti-angiogenic factors: Their prognostic relevance in locally advanced hepatocellular carcinoma. Molecular and Cellular Biochemistry 2013;383(1-2):103-12. | Exclude on population: healthy controls |
| Sharma, Balkrishan, Srinivasan, Radhika, Chawla, Yogesh Kumar, Kapil, Shweta, Saini, Nitin, Singla, Bhupesh, et al. Clinical utility of prothrombin induced by vitamin K absence in the detection of hepatocellular carcinoma in Indian population. Hepatology international 2010;4(3):569-76. | Does not meet full eligibility criteria: includes people without cirrhosis |
| Sharma, Balkrishan, Srinivasan, Radhika, Chawla, Yogesh Kumar, Kapil, Shweta, Saini, Nitin, Singla, Bhupesh, et al. Clinical utility of prothrombin induced by vitamin K absence in the detection of hepatocellular carcinoma in Indian population. Hepatology international 2010;4(3):569-76. | Duplicate (including Cochrane includes) |
| She, Sha, Xiang, Yi, Yang, Min, Ding, Xiangchun, Liu, Xiaoyan, Ma, Lina, et al. C-reactive protein is a biomarker of AFP-negative HBV-related hepatocellular carcinoma. International journal of oncology 2015;47(2):543-54. | Exclude on index test |
| Shehab-Eldeen, Somaia, Metwaly, Mohamed F., Saber, Safa M., El-Kousy, Salah M., Badr, Eman A. E. MicroRNA-29a and MicroRNA-124 as novel biomarkers for hepatocellular carcinoma. Digestive and liver disease : official journal of the Italian Society of Gastroenterology and the Italian Association for the Study of the Liver 2023;55(2):283-90. | Does not meet full eligibility criteria: includes people without cirrhosis |
| Shehab-Eldeen, Somaia, Metwaly, Mohamed F., Saber, Safa M., El-Kousy, Salah M., Badr, Eman Ae. MicroRNA-29a and MicroRNA-124 as novel biomarkers for hepatocellular Carcinoma. Digestive and liver disease : official journal of the Italian Society of Gastroenterology and the Italian Association for the Study of the Liver 2022;():n. pag.. | Does not meet full eligibility criteria: includes people without cirrhosis |
| Shehab-Eldeen, Somaia, Nada, Ali, Abou-Elela, Dalia, El-Naidany, Sherin, Arafat, Eman. Diagnostic Performance of microRNA-122 and microRNA-224 in | Does not meet full eligibility criteria: includes people without cirrhosis |

| Reference | Reason for exclusion |
|---|---|
| Hepatitis C Virus-Induced Hepatocellular Carcinoma (HCC). Asian Pacific journal of cancer prevention : APJCP 2019;20(8):2515-22. | |
| Shehata, F., Monem, N. A., Sakr, M., Kasem, S. Epidermal growth factor, its receptor and transforming growth factor-beta1 in the diagnosis of HCV-induced hepatocellular carcinoma. Medical Oncology 2013;30(3):673. | Exclude on population: not cirrhosis |
| Shen, Jing, Wang, Antai, Wang, Qiao, Gurvich, Irina, Siegel, Abby B., Remotti, Helen. Exploration of genome-wide circulating microRNA in hepatocellular carcinoma: MiR-483-5p as a potential biomarker. Cancer epidemiology, biomarkers & prevention : a publication of the American Association for Cancer Research, cosponsored by the American Society of Preventive Oncology 2013;22(12):2364-73. | Exclude on population: healthy controls |
| Shen, Qiujin, Fan, Jia, Yang, Xin-Rong, Tan, Yexiong, Zhao, Weifeng, Xu, Yang, et al. Serum DKK1 as a protein biomarker for the diagnosis of hepatocellular carcinoma: a large-scale, multicentre study. The Lancet. Oncology 2012;13(8):817-26. | Exclude on population: not cirrhosis |
| Shen, Qiujin, Fan, Jia, Yang, Xin-Rong, Tan, Yexiong, Zhao, Weifeng, Xu, Yang, et al. Serum DKK1 as a protein biomarker for the diagnosis of hepatocellular carcinoma: a large-scale, multicentre study. The Lancet. Oncology 2012;13(8):817-26. | Duplicate (including Cochrane includes) |
| Shen, Xianjuan, Xue, Yajing, Cong, Hui, Wang, Xudong. Dysregulation of serum microRNA-574-3p and its clinical significance in hepatocellular carcinoma. Annals of clinical biochemistry 2018;55(4):478-84. | Exclude on population: healthy controls |
| Shen, Y., Chen, J., Wu, J., Li, T., Yi, C., Wang, K., et al. Combination of an Autoantibody Panel and Alpha-Fetoprotein for Early Detection of Hepatitis B Virus-Associated Hepatocellular Carcinoma. Cancer Prevention Research 2024;17(5):227 “ 235. | Does not meet full eligibility criteria: includes people without cirrhosis |
| Sheneef, Abeer, Gouda, Asmaa M., Mohammad, Asmaa N., Yousef, Laila M. Serum MicroRNA-122 and MicroRNA-155: Markers of Disease Progression in Hepatitis C viral infection. The Egyptian journal of immunology 2017;24(2):33-46. | Exclude on population: not cirrhosis |
| Sheng, Lang-Qing, Li, Jia-Rong, Qin, Hao, Liu, Ling, Zhang, Da-Dong, Zhang, Qi, et al. Blood exosomal micro ribonucleic acid profiling reveals the complexity of hepatocellular carcinoma and identifies potential biomarkers for differential diagnosis. World journal of gastrointestinal oncology 2020;12(10):1195-2008. | Does not meet full eligibility criteria: includes people without cirrhosis |
| Sheng, Shi Le, Wang, Qing, Huang, Gang, Yu, Bin. Simultaneous determination of alpha-fetoprotein immune complexes and alpha-fetoprotein concentration in hepatocellular carcinoma using dual-label time-resolved immunofluorometric assays. Journal of clinical laboratory analysis 2009;23(3):179-85. | Does not meet full eligibility criteria: includes people without cirrhosis |
| Sherman, M & Peltekian, K M. Screening for hepatocellular carcinoma in chronic carriers of hepatitis B virus: incidence and prevalence of hepatocellular carcinoma in a North American urban population. Hepatology (Baltimore, Md.) 1995;22(2):432-8. | Exclude on population: not cirrhosis |
| Sheu, J. C. & Sung, J. L. Early detection of hepatocellular carcinoma by real-time ultrasonography. A prospective study. Cancer 1985;56(3):660-66. | No 2x2 data |
| Shi, Bao-Min, Lu, Wen, Ji, Kun, Wang, Yu-Feng, Xiao, Shuai. Study on the value of serum miR-106b for the early diagnosis of hepatocellular carcinoma. World journal of gastroenterology 2017;23(20):3713-20. | Exclude on population: not cirrhosis |
| Shi, Liang, Wu, Li-Li, Yang, Jian-Rong, Chen, Xiao-Fei, Zhang, Yi, Chen, Zeng-Qiang, et al. Serum peroxiredoxin3 is a useful biomarker for early diagnosis and assessemnt of prognosis of hepatocellular carcinoma in Chinese patients. Asian Pacific journal of cancer prevention : APJCP 2014;15(7):2979-86. | No 2x2 data |
| Shi, M., Chen, M. S., Sekar, K., Tan, C. K., Ooi, L. L. A blood-based three-gene signature for the non-invasive detection of early human hepatocellular carcinoma. European Journal of Cancer 2014;50(5):928-36. | Exclude on population: healthy controls |
| Shi, Y., Chen, J., Li, L., Sun, Z., Zen, L., Xu, S., Zhang, Y. A study of diagnostic value of golgi protein GP73 and its genetic assay in primary hepatic carcinoma. Technology in cancer research & treatment 2011;10(3):287-94. | Exclude on population: not cirrhosis |

| Reference | Reason for exclusion |
|---|---|
| Shim, J. J., Kim, J. W., Lee, C. K., Jang, J. Y. Oral antiviral therapy improves the diagnostic accuracy of alpha-fetoprotein levels in patients with chronic hepatitis B. Journal of Gastroenterology and Hepatology (Australia) 2014;29(9):1699-7005. | Does not meet full eligibility criteria: includes people without cirrhosis |
| Shimagaki, Tomonari, Yoshio, Sachiyo, Kawai, Hironari, Sakamoto, Yuzuru, Doi, Hiroyoshi, Matsuda, Michitaka, et al. Serum milk fat globule-EGF factor 8 (MFG-E8) as a diagnostic and prognostic biomarker in patients with hepatocellular carcinoma. Scientific reports 2019;9(1):15788. | Exclude on population: not cirrhosis |
| Shimauchi, Y., Tanaka, M., Kuromatsu, R., Ogata, R., Tateishi, Y., Itano, S., et al. A simultaneous monitoring of Lens culinaris agglutinin A-reactive alpha-fetoprotein and des-gamma-carboxy prothrombin as an early diagnosis of hepatocellular carcinoma in the follow-up of cirrhotic patients. Oncology reports 2000;7(2):249-56. | Does not meet full eligibility criteria: pre-2005 |
| Shimizu, A., Ito, K., Sasaki, K., Hayashida, M., Tanabe, M., Shimizu, K. Small hyperintense hepatic lesions on T1-weighted images in patients with cirrhosis: evaluation with serial MRI and imaging features for clinical benignity. Magnetic Resonance Imaging 2007;25(10):1430-36. | Exclude on population: HCC participants not treatment-naive |
| Shimizu, Atsuya, Shiraki, Katsuya, Ito, Takeshi, Sugimoto, Kazushi, Sakai, Takahisa, Ohmori, Shigeru, et al. Sequential fluctuation pattern of serum des-gamma-carboxy prothrombin levels detected by high-sensitive electrochemiluminescence system as an early predictive marker for hepatocellular carcinoma in patients with cirrhosis. International journal of molecular medicine 2002;9(3):245-50. | Does not meet full eligibility criteria: pre-2005 |
| Shimizu, Atsuya, Shiraki, Katsuya, Ito, Takeshi, Sugimoto, Kazushi, Sakai, Takahisa, Ohmori, Shigeru, et al. Sequential fluctuation pattern of serum des-gamma-carboxy prothrombin levels detected by high-sensitive electrochemiluminescence system as an early predictive marker for hepatocellular carcinoma in patients with cirrhosis. International journal of molecular medicine 2002;9(3):245-50. | Does not meet full eligibility criteria: pre-2005 |
| Shimizu, I. & Ogawa, K. Clinical investigations of alpha fetoprotein by radioimmunoassay. IRYO - Japanese Journal of National Medical Services 1977;31(5):420-02. | Does not meet full eligibility criteria: pre-2005 |
| Shimizu, K., Taniichi, T., Satomura, S., Matsuura, S., Taga, H. Establishment of assay kits for the determination of microheterogeneities of alpha-fetoprotein using lectin-affinity electrophoresis. Clinica chimica acta; international journal of clinical chemistry 1993;214(1):3-12. | Does not meet full eligibility criteria: includes people without cirrhosis |
| Shimizu, Tomo, Sawada, Takashi, Asai, Tomohide, Kanetsuki, Yuka, Hirota, Jiro, Moriguchi, Michihisa, et al. Hepatocellular carcinoma diagnosis using a novel electrochemiluminescence immunoassay targeting serum IgM-free AIM. Clinical journal of gastroenterology 2022;15(1):41-51. | Exclude on population: not cirrhosis |
| Shimizu, Y., Minemura, M., Tsukishiro, T., Kashii, Y., Miyamoto, M., Nishimori, H., Higuchi, K. Serum concentration of intercellular adhesion molecule-1 in patients with hepatocellular carcinoma is a marker of the disease progression and prognosis. Hepatology 1995;22(2):525-31. | Exclude on index test |
| Shin, Hyunjae, Hur, Moon Haeng, Song, Byeong Geun, Park, Soo Young, Kim, Gi-Ae, Choi, Gwanghyeon, et al. AI model using CT-based imaging biomarkers to predict hepatocellular carcinoma in patients with chronic hepatitis B. Journal of hepatology 2025;82(6):1080â€"1088. | Exclude on study design: not a 1- or 2-gate test accuracy study |
| Shin, S., Sohn, W., Chang, Y., Cho, Y., Kwon, M. J., Wild, S. H., Byrne, C. D. Potential role of Fibrosis-4 score in hepatocellular carcinoma screening: The Kangbuk Samsung Health Study. Hepatology Research 2024;54(6):551 " 561. | Exclude on population: not cirrhosis |
| Shiode, Y., Kodama, T., Sato, Y., Takahashi, R., Matsumae, T., Shirai, K., et al. Folate receptor 1 is a stemness trait-associated diagnostic and prognostic marker for hepatocellular carcinoma. Biomarker Research 2025;13(1):37. | Exclude on population: not cirrhosis |
| Shiraki, K., Takase, K., Tameda, Y., Hamada, M., Kosaka, Y. A clinical study of lectin-reactive alpha-fetoprotein as an early indicator of hepatocellular carcinoma in the follow-up of cirrhotic patients. Hepatology (Baltimore, Md.) 1995;22(3):802-07. | Does not meet full eligibility criteria: pre-2005 |
| Shitani, Masahiro, Sasaki, Shigeru, Akutsu, Noriyuki, Takagi, Hideyasu, Suzuki, Hiromu, Nojima, Masanori, et al. Genome-wide analysis of DNA methylation | Exclude on population: tissue samples |

| Reference | Reason for exclusion |
|---|---|
| identifies novel cancer-related genes in hepatocellular carcinoma. Tumour biology : the journal of the International Society for Oncodevelopmental Biology and Medicine 2012;33(5):1307-17. | |
| Shu, H., Li, W., Shang, S., Qin, X., Zhang, S. Diagnosis of AFP-negative early-stage hepatocellular carcinoma using Fuc-PON1. Discovery Medicine 2017;23(126):n. pag.. | Exclude on index test |
| Shu, Hong, Kang, Xiaonan, Guo, Kun, Li, Shan, Li, Mei, Sun, Lu, et al. Diagnostic value of serum haptoglobin protein as hepatocellular carcinoma candidate marker complementary to alpha fetoprotein. Oncology reports 2010;24(5):1271-6. | Does not meet full eligibility criteria: includes people without cirrhosis |
| Shu, Hong, Kang, Xiaonan, Guo, Kun, Li, Shan, Li, Mei, Sun, Lu, et al. Diagnostic value of serum haptoglobin protein as hepatocellular carcinoma candidate marker complementary to alpha fetoprotein. Oncology reports 2010;24(5):1271-76. | Does not meet full eligibility criteria: includes people without cirrhosis |
| Si, J., Zou, Y., Gao, Y., Chen, J., Jiang, W., Shen, X., Zhu, C. tRF-3a-Pro: A Transfer RNA-Derived Small RNA as a Novel Biomarker for Diagnosis of Hepatitis B Virus-Related Hepatocellular Carcinoma. Cell Proliferation 2025;58(7):e70006. | Does not meet full eligibility criteria: includes people without cirrhosis |
| Si, Yuan-Quan, Wang, Xiu-Qin, Fan, Gang, Wang, Chang-Yin, Zheng, Yuan-Wen, Song, Xie, et al. Value of AFP and PIVKA-II in diagnosis of HBV-related hepatocellular carcinoma and prediction of vascular invasion and tumor differentiation. Infectious agents and cancer 2020;15(1):70. | Exclude on population: healthy controls |
| Siaw, A. D. J., Armasu, S. M., Frank, J. A., Yan, I. K., Wangensteen, K. J., Patel, T. Protein biomarkers of HCC development in patients with metabolic liver disease. Cancer Research 2025;85(8 Supplement 1):n. pag.. | CONFERENCE ABSTRACT |
| Sieg, A., Schonpflug, A., Seitz, H. K. [Thyroxine-binding globulin--not a tumor marker of hepatocellular cancer]. Thyroxin-bindendes Globulin--Kein Tumormarker des hepatozellularen Karzinoms. 1991;29(3):101-04. | Does not meet full eligibility criteria: pre-2005 |
| Simao, Adelia, Madaleno, Joao, Silva, Nuno, Rodrigues, Fernando, Caseiro, Paula, Costa, Jose Nascimento. Plasma osteopontin is a biomarker for the severity of alcoholic liver cirrhosis, not for hepatocellular carcinoma screening. BMC gastroenterology 2015;15():73. | Duplicate (including Cochrane includes) |
| Simon, G., Link, T. M., Wortler, K., Doebereiner, F., Schulte-Frohlinde, E., Daldrup-Link, H., Settles, M. Detection of hepatocellular carcinoma: comparison of Gd-DTPA- and ferumoxides-enhanced MR imaging. European radiology 2005;15(5):895-903. | Exclude on population: clinical signs and symptoms of HCC |
| Singal, A. G. The efficacy and effectiveness of hepatocellular carcinoma surveillance in patients with cirrhosis. Hepatic Oncology 2015;2(2):97-99. | Exclude on study design: not a 1- or 2-gate test accuracy study |
| Singal, A. G., Conjeevaram, H. S., Volk, M. L., Fu, S., Fontana, R. J., Askari, F., et al. Effectiveness of hepatocellular carcinoma surveillance in patients with cirrhosis. Cancer Epidemiology Biomarkers and Prevention 2012;21(5):793-99. | Duplicate (including Cochrane includes) |
| Singal, Amit G., Mukherjee, Ashin, Elmunzer, B. Joseph, Higgins, Peter D. R., Lok, Anna S., Zhu, Ji, Marrero, Jorge A. Machine learning algorithms outperform conventional regression models in predicting development of hepatocellular carcinoma. The American journal of gastroenterology 2013;108(11):1723-30. | Exclude on study design: not a 1- or 2-gate test accuracy study |
| Singal, Amit G., Patibandla, Sruthi, Obi, Joseph, Fullington, Hannah, Parikh, Neehar D., Yopp, Adam C. Benefits and Harms of Hepatocellular Carcinoma Surveillance in a Prospective Cohort of Patients With Cirrhosis. Clinical gastroenterology and hepatology : the official clinical practice journal of the American Gastroenterological Association 2021;19(9):1925-32.e1e1. | No 2x2 data |
| Singal, Amit G., Tayob, Nabihah, Mehta, Anand, Marrero, Jorge A., El-Serag, Hashem, Jin, Qingchun, et al. GALAD demonstrates high sensitivity for HCC surveillance in a cohort of patients with cirrhosis. Hepatology (Baltimore, Md.) 2022;75(3):541-49. | Secondary publication of included study |
| Singh, H., Choudhary, V., Choudhary, B. EVALUATION OF SERUM RETINOL BINDING PROTEIN 4 AS A DIAGNOSTIC BIOMARKER IN HEPATOCELLULAR CARCINOMA. International Journal of Academic Medicine and Pharmacy 2025;7(1):880 “ 884. | Exclude on population: not cirrhosis |

| Reference | Reason for exclusion |
|---|---|
| Singh, Pankaj, Erickson, Richard A., Mukhopadhyay, Phalguni, Gopal, Shanthi, Kiss, Alex, Khan, Ahmed. EUS for detection of the hepatocellular carcinoma: results of a prospective study. Gastrointestinal endoscopy 2007;66(2):265-73. | Exclude on population: clinical signs and symptoms of HCC |
| Sinharay, R., Grant, A. J., Rivett, L., Blackwell, R., Mells, G. Assessing efficacy of hepatocellular carcinoma prediction scores to prioritise hepatitis B surveillance in the COVID-19 era. GastroHep 2021;3(2):80-87. | Exclude on population: not cirrhosis |
| Sinn, D. H., Yi, J., Choi, M. S., Kim, Y. J., Gwak, G. Y., Lee, J. H., et al. Serum alpha-fetoprotein may have a significant role in the surveillance of hepatocellular carcinoma in hepatitis b endemic areas. Hepato-Gastroenterology 2015;62(138):327-32. | Does not meet full eligibility criteria: includes people without cirrhosis |
| Snowberger, N, Chinnakotla, S, Lepe, R M, Peattie, J, Goldstein, R, Klintmalm, G B. Alpha fetoprotein, ultrasound, computerized tomography and magnetic resonance imaging for detection of hepatocellular carcinoma in patients with advanced cirrhosis. Alimentary pharmacology & therapeutics 2007;26(9):1187-94. | Does not meet full eligibility criteria: includes people without cirrhosis |
| Sofue, K., Tsurusaki, M., Kawasaki, R., Fujii, M. Evaluation of hypervascular hepatocellular carcinoma in cirrhotic liver: Comparison of different concentrations of contrast material with multi-detector row helical CT - A prospective randomized study. European Journal of Radiology 2011;80(3):e237-42. | Exclude on population: clinical signs and symptoms of HCC |
| Soga, K., Watanabe, T., Aikawa, K., Toshima, M., Shibasaki, K. Serum des-gamma-carboxyprothrombin level by a modified enzyme immunoassay method in hepatocellular carcinoma: clinical significance in small hepatocellular carcinoma. Hepato-gastroenterology 1998;45(23):1737-41. | Does not meet full eligibility criteria: pre-2005 |
| Soga, Tomoyoshi, Sugimoto, Masahiro, Honma, Masashi, Mori, Masayo, Igarashi, Kaori, Kashikura, Kasumi, et al. Serum metabolomics reveals gamma-glutamyl dipeptides as biomarkers for discrimination among different forms of liver disease. Journal of hepatology 2011;55(4):896-905. | No 2x2 data |
| Sohail, M., Ahmad, W., Zeb, A., Khalil, M. N., Fazleamin, Khursheed, W. A., et al. INVESTIGATING THE ROLE OF NON-INVASIVE BIOMARKERS IN THE EARLY DIAGNOSIS OF HEPATOCELLULAR CARCINOMA, ESPECIALLY IN PATIENTS WITH LIVER CIRRHOSIS. Journal of Population Therapeutics and Clinical Pharmacology 2024;31(9):415 " 421. | Exclude on index test |
| Soliman, Hanan H., Nagy, Hala, Kotb, Nesreen. The role of chemokine CC ligand 20 in patients with liver cirrhosis and hepatocellular carcinoma. The International journal of biological markers 2012;27(2):e125-31. | Exclude on index test |
| Son, J. A., Weon, J. H., Baek, G. O., Ahn, H. R., Choi, J. Y., Yoon, M. G., et al. Circulating small extracellular vesicle-derived splicing factor 3b subunit 4 as a non-invasive diagnostic biomarker of early hepatocellular carcinoma. Journal of Experimental and Clinical Cancer Research 2023;42(1):288. | No 2x2 data |
| Son, J. A., Weon, J. H., Baek, G. O., Ahn, H. R., Choi, J. Y., Yoon, M. G., et al. Circulating small extracellular vesicle-derived splicing factor 3b subunit 4 as a non-invasive diagnostic biomarker of early hepatocellular carcinoma. Journal of Experimental and Clinical Cancer Research 2023;42(1):288. | Does not meet full eligibility criteria: includes people without cirrhosis |
| Son, Jung Hee, Choi, Sang Hyun, Kim, So Yeon, Jang, Hye Young, Byun, Jae Ho, Won, Hyung Jin, Lee, So Jung. Validation of US Liver Imaging Reporting and Data System Version 2017 in Patients at High Risk for Hepatocellular Carcinoma. Radiology 2019;292(2):390-97. | Secondary publication of included study |
| Song J. Significance of alpha fetoprotein in the surveillance of HBV-associated hepatocellular carcinoma. Hepatology 2011;54(SUPPL. 1):1419A. | CONFERENCE ABSTRACT |
| Song, Byung-Cheol, Chung, Young-Hwa, Kim, Jung A, Choi, Won-Beom, Suh, Dong Dae, Pyo, Seung Il, et al. Transforming growth factor-beta1 as a useful serologic marker of small hepatocellular carcinoma. Cancer 2002;94(1):175-80. | Exclude on population: healthy controls |
| Song, Byung-Cheol, Chung, Young-Hwa, Kim, Jung A., Choi, Won-Beom, Suh, Dong Dae, Pyo, Seung Il, et al. Transforming growth factor-beta1 as a useful serologic marker of small hepatocellular carcinoma. Cancer 2002;94(1):175-80. | Does not meet full eligibility criteria: includes people without cirrhosis |
| Song, K. S., Lee, A., Choi, J. R. Diagnostic efficacy of plasma urokinase-type plasminogen activator and plasminogen activator inhibitor-2 in differentiation of hepatocellular carcinoma from cirrhosis. Thrombosis and haemostasis 1995;74(3):864-67. | Does not meet full eligibility criteria: pre-2005 |

| Reference | Reason for exclusion |
|---|---|
| Song, Peipei, Feng, Xiaobin, Inagaki, Yoshinori, Song, Tianqiang, Zhang, Keming, Wang, Zhigang, et al. Clinical utility of simultaneous measurement of alpha-fetoprotein and des-gamma-carboxy prothrombin for diagnosis of patients with hepatocellular carcinoma in China: A multi-center case-controlled study of 1,153 subjects. Bioscience trends 2014;8(5):266-73. | Exclude on population: not cirrhosis |
| Song, Peipei, Feng, Xiaobin, Inagaki, Yoshinori, Song, Tianqiang, Zhang, Keming, Wang, Zhigang, et al. Clinical utility of simultaneous measurement of alpha-fetoprotein and des-gamma-carboxy prothrombin for diagnosis of patients with hepatocellular carcinoma in China: A multi-center case-controlled study of 1,153 subjects. Bioscience trends 2014;8(5):266-73. | Exclude on population: not cirrhosis |
| Song, Ting, Wang, Lili, Su, Bin, Zeng, Weiping, Jiang, Taiyi, Zhang, Tong, Sun, Guizhen. Diagnostic value of alpha-fetoprotein, Lens culinaris agglutinin-reactive alpha-fetoprotein, and des-gamma-carboxyprothrombin in hepatitis B virus-related hepatocellular carcinoma. The Journal of international medical research 2020;48(3):300060519889270. | Does not meet full eligibility criteria: includes people without cirrhosis |
| Song, Ting, Wang, Lili, Su, Bin, Zeng, Weiping, Jiang, Taiyi, Zhang, Tong, Sun, Guizhen. Diagnostic value of alpha-fetoprotein, Lens culinaris agglutinin-reactive alpha-fetoprotein, and des-gamma-carboxyprothrombin in hepatitis B virus-related hepatocellular carcinoma. The Journal of international medical research 2020;48(3):300060519889270. | Duplicate (including Cochrane includes) |
| Song, W., Wu, J., Wu, L. Signifi cance of serum lncRNA-PVT1 expression in diagnosis and prognosis of hepatocellular carcinoma. Journal of Practical Oncology 2020;35(4):317-21. | Foreign language |
| Song, Xiaoting, Wu, Ailu, Ding, Zhixiao, Liang, Shixiong. Soluble Axl Is a Novel Diagnostic Biomarker of Hepatocellular Carcinoma in Chinese Patients with Chronic Hepatitis B Virus Infection. Cancer research and treatment 2020;52(3):789-97. | Duplicate (including Cochrane includes) |
| Song, Xiaoting, Wu, Ailu, Ding, Zhixiao, Liang, Shixiong. Soluble Axl Is a Novel Diagnostic Biomarker of Hepatocellular Carcinoma in Chinese Patients with Chronic Hepatitis B Virus Infection. Cancer research and treatment 2020;52(3):789-97. | Exclude on population: not cirrhosis |
| Soresi, M., Terranova, A., Licata, A., Serruto, A., Montalto, G., Brancatelli, G. Surveillance program for diagnosis of hcc in liver cirrhosis: Role of ultrasound echo patterns. BioMed Research International 2017;2017():4932759. | No 2x2 data |
| Soresi, Maurizio, Magliarisi, Carmela, Campagna, Pietro, Leto, Gaetano, Bonfissuto, Giulio, Riili, Anna, et al. Usefulness of alpha-fetoprotein in the diagnosis of hepatocellular carcinoma. Anticancer research 2003;23(2C):1747-53. | Does not meet full eligibility criteria: pre-2005 |
| Soroida, Yoko, Ohkawa, Ryunosuke, Nakagawa, Hayato, Satoh, Yumiko, Yoshida, Haruhiko, Kinoshita, Hiroto, et al. Increased activity of serum mitochondrial isoenzyme of creatine kinase in hepatocellular carcinoma patients predominantly with recurrence. Journal of hepatology 2012;57(2):330-6. | Exclude on population: HCC participants not treatment-naive |
| Soroida, Yoko, Ohkawa, Ryunosuke, Nakagawa, Hayato, Satoh, Yumiko, Yoshida, Haruhiko, Kinoshita, Hiroto, et al. Increased activity of serum mitochondrial isoenzyme of creatine kinase in hepatocellular carcinoma patients predominantly with recurrence. Journal of hepatology 2012;57(2):330-36. | Duplicate (including Cochrane includes) |
| Sorop, Andrei, Iacob, Razvan, Iacob, Speranta, Constantinescu, Diana, Chitoiu, Leona, Fertig, Tudor Emanuel, et al. Plasma Small Extracellular Vesicles Derived miR-21-5p and miR-92a-3p as Potential Biomarkers for Hepatocellular Carcinoma Screening. Frontiers in genetics 2020;11():712. | Exclude on population: HCC participants not treatment-naive |
| Sorvillo, Francesca, Mazziotti, Gherardo, Carbone, Antonella, Morisco, Filomena, Cioffi, Michele, Rotondi, Mario, et al. Increased serum reverse triiodothyronine levels at diagnosis of hepatocellular carcinoma in patients with compensated HCV-related liver cirrhosis. Clinical endocrinology 2003;58(2):207-12. | Does not meet full eligibility criteria: pre-2005 |
| Soulier, J. P. & Gozin, D. [A new method of functional assay of des-gamma-carboxyprothrombin using staphylocoagulase. Application to the diagnosis of hepatocellular carcinoma]. Nouvelle methode de dosage fonctionnel de la de- | Does not meet full eligibility criteria: pre-2005 |

| Reference | Reason for exclusion |
|---|---|
| gamma-carboxyprothrombine a l'aide de staphylocoagulase. Application au diagnostic des carcinomes hepatocellulaires. 1985;14(40):2049-52. | |
| Soulier, J. P. & Gozin, D. A new method to assay des-gamma-carboxyprothrombin. Results obtained in 75 cases of hepatocellular carcinoma. Gastroenterology 1986;91(5):1258-62. | Does not meet full eligibility criteria: includes people without cirrhosis |
| Soundararajan, R., Pooja, A. B., Gupta, P., Gulati, A., Kalra, N., Singh, S., et al. ghted imaging; Diagnostic Performance of Abbreviated MRI for HCC Detection in Patients with Non-alcoholic Fatty Liver Disease. ghted imaging; Journal of Clinical and Experimental Hepatology 2023;():n. pag.. | Does not meet full eligibility criteria: includes people without cirrhosis |
| Spadaro, A., Ajello, A., Morace, C., Zirilli, A., D'Arrigo, G., Luigiano, C., et al. Serum chromogranin-A in hepatocellular carcinoma: Diagnostic utility and limits. World Journal of Gastroenterology 2005;11(13):1987-90. | Does not meet full eligibility criteria: includes people without cirrhosis |
| Spadaro, Aldo, Ajello, Antonino, Luigiano, Carmelo, Morace, Carmela, Resta, Maria Letizia, Berlinghieri, Grazia, et al. Low utility of plasma Nociceptin/orphanin FQ in the diagnosis of hepatocellular carcinoma. World journal of gastroenterology 2006;12(29):4716-20. | Does not meet full eligibility criteria: includes people without cirrhosis |
| Stanciu, L., Dumitrascu, D., Radu, D. Non-specific tumoral markers in hepatocellular carcinoma. Medecine interne 1990;28(2):139-44. | Does not meet full eligibility criteria: pre-2005 |
| Steingruber, Iris E., Mallouhi, Ammar, Czermak, Benedikt V., Waldenberger, Peter, Gassner, Eva, Offner, Felix, et al. Pretransplantation evaluation of the cirrhotic liver with explantation correlation: accuracy of CT arterioportography and digital subtraction hepatic angiography in revealing hepatocellular carcinoma. AJR. American journal of roentgenology 2003;181(1):99-108. | Exclude on population: clinical signs and symptoms of HCC |
| Sterling, R. K., Wright, E. C., Morgan, T. R., Seeff, L. B., Hoefs, J. C., Di Bisceglie, A. M., Dienstag, J. L. Frequency of Elevated Hepatocellular Carcinoma (HCC) Biomarkers in Patients With Advanced Hepatitis C. American Journal of Gastroenterology 2011;():n. pag.. | Duplicate (including Cochrane includes) |
| Sterling, Richard K., Jeffers, Lennox, Gordon, Fredric, Sherman, Morris, Venook, Alan P., Reddy, K. Rajender, Satomura, Shinji. Clinical utility of AFP-L3% measurement in North American patients with HCV-related cirrhosis. The American journal of gastroenterology 2007;102(10):2196-2005. | Secondary publication of included study |
| Sterling, Richard K., Jeffers, Lennox, Gordon, Fredric, Venook, Alan P., Reddy, K. Rajender, Satomura, Shinji, et al. Utility of Lens culinaris agglutinin-reactive fraction of alpha-fetoprotein and des-gamma-carboxy prothrombin, alone or in combination, as biomarkers for hepatocellular carcinoma. Clinical gastroenterology and hepatology : the official clinical practice journal of the American Gastroenterological Association 2009;7(1):104-13. | Duplicate (including Cochrane includes) |
| Sterling, Richard K., Wright, Elizabeth C., Morgan, Timothy R., Seeff, Leonard B., Hoefs, John C., Di Bisceglie, Adrian M., Dienstag, Jules L. Frequency of elevated hepatocellular carcinoma (HCC) biomarkers in patients with advanced hepatitis C. The American journal of gastroenterology 2012;107(1):64-74. | Duplicate (including Cochrane includes) |
| Stojakovic, T., Putz-Bankuti, C., Scharnagl, H., Pock, H. Alpha-fetoprotein and des-gamma-carboxy prothrombin asserum markers for hepatocellular carcinoma. Clinical Chemistry and Laboratory Medicine 2011;49(SUPPL. 1):S275. | CONFERENCE ABSTRACT |
| Stoker, J., Romijn, M. G., de Man, R. A., Brouwer, J. T., Weverling, G. J., van Muiswinkel, J. M., et al. Prospective comparative study of spiral computer tomography and magnetic resonance imaging for detection of hepatocellular carcinoma. Gut 2002;51(1):105-07. | Exclude on population: clinical signs and symptoms of HCC |
| Strobel, D., Kleinecke, C., Hansler, J., Frieser, M., Handl, T., Hahn, E. G. Contrast-enhanced sonography for the characterisation of hepatocellular carcinomas--correlation with histological differentiation. Ultraschall in der Medizin (Stuttgart, Germany : 1980) 2005;26(4):270-76. | Exclude on population: clinical signs and symptoms of HCC |
| Su, Benzhe, Luo, Ping, Yang, Zhao, Yu, Pei, Li, Zaifang, Yin, Peiyuan, et al. A novel analysis method for biomarker identification based on horizontal relationship: identifying potential biomarkers from large-scale hepatocellular carcinoma metabolomics data. Analytical and bioanalytical chemistry 2019;411(24):6377-86. | Does not meet full eligibility criteria: includes people without cirrhosis |
| Suarez-Munoz, Miguel Angel, Leiva-Vera, Maria Carmen, Santoyo-Santoyo, Julio, Fernandez-Aguilar, Jose Luis, Perez-Daga, Jose Antonio, Sanchez- | Foreign language |

| Reference | Reason for exclusion |
|---|---|
| Perez, Belinda, et al. [Detection of neoplastic lesions in cirrhotic patients waiting for liver transplantation]. Deteccion de lesiones neoplasicas en pacientes cirroticos candidatos a trasplante hepatico. 2006;80(3):157-61. | |
| Sugimoto, Katsutoshi, Kakegawa, Tatsuya, Takahashi, Hiroshi, Tomita, Yusuke, Abe, Masakazu, Yoshimasu, Yu, et al. Usefulness of Modified CEUS LI-RADS for the Diagnosis of Hepatocellular Carcinoma Using Sonazoid. Diagnostics (Basel, Switzerland) 2020;10(10):n. pag.. | Exclude on population: clinical signs and symptoms of HCC |
| Suh, Beomseok, Park, Sehhoon, Shin, Dong Wook, Yun, Jae Moon, Yang, Hyung-Kook, Yu, Su Jong, et al. High liver fibrosis index FIB-4 is highly predictive of hepatocellular carcinoma in chronic hepatitis B carriers. Hepatology (Baltimore, Md.) 2015;61(4):1261-68. | Exclude on study design: not a 1- or 2-gate test accuracy study |
| Sukaram, T., Tansawat, R., Phathong, C., Rerknimitr, R. Volatile organic compounds for diagnosis of early hepatocellular carcinoma in at-risk patients. Clinica Chimica Acta 2024;556():117831. | Does not meet full eligibility criteria: includes people without cirrhosis |
| Sultanik, P., Ginguay, A., Vandame, J., Popovici, T., Meritet, J. F., Cynober, L., Pol, S. Diagnostic accuracy of des-gamma-carboxy prothrombin for hepatocellular carcinoma in a French cohort using the Lumipulse G600 analyzer. Journal of Viral Hepatitis 2017;24(1):80-85. | Duplicate (including Cochrane includes) |
| Sun, B., Wang, B., Yu, Y., Lin, S., Luo, L., Wang, Y. Significance of glypican-3 (GPC3) expression in hepatocellular cancer diagnosis. Medical Science Monitor 2017;23():850-55. | Exclude on population: not cirrhosis |
| Sun, G., Zhang, C., Feng, M., Liu, W., Xie, H., Qin, Q., Zhao, E. Methylation analysis of p16, SLIT2, SCARA5, and Runx3 genes in hepatocellular carcinoma. Medicine (United States) 2017;96(41):e8279. | Exclude on population: not cirrhosis |
| Sun, H. & Liu, N. Diagnostic value of serum STIP1 in HCC and AFP-negative HCC. Laboratory Medicine 2024;55(6):700 " 707. | Exclude on population: not cirrhosis |
| Sun, Hongbo, Chua, Mei-Sze, Yang, Dorothy, Tsalenko, Anya, Peter, Brian J. Antibody Arrays Identify Potential Diagnostic Markers of Hepatocellular Carcinoma. Biomarker insights 2008;3():1-18. | Exclude on population: not cirrhosis |
| Sun, J., Sun, X., He, W., Huang, B., Feng, W., Han, Z., et al. A liquid biopsy approach detects HCC and identifies GJA4 as a potential biomarker for HBV-HCC via plasma cfDNA methylome profiling. Clinical Epigenetics 2025;17(1):98. | No 2x2 data |
| Sun, Jianping, Zhao, Yanan, Qin, Ling, Li, Kang, Zhao, Yan, Sun, Huanqin, Zhang, Ting. Metabolomic Profiles for HBV Related Hepatocellular Carcinoma Including Alpha-Fetoproteins Positive and Negative Subtypes. Frontiers in oncology 2019;9():1069. | Exclude on index test |
| Sun, L. Y., Wang, N. Y., Diao, Y. K., Yan, C. L., Fan, Z. P., Wei, L. H., et al. Comparison between models for detecting hepatocellular carcinoma in patients with chronic liver diseases of various etiologies: ASAP score versus GALAD score. Hepatobiliary and Pancreatic Diseases International 2025;24(4):412 " 422. | Duplicate (including Cochrane includes) |
| Sun, Li, Xu, Mu, Zhang, Guoying, Dong, Lin, Wu, Jie, Wei, Chenchen, Xu, Kexin. Identification of Circulating Exosomal miR-101 and miR-125b Panel Act as a Potential Biomarker for Hepatocellular Carcinoma. International journal of genomics 2021;2021():1326463. | Exclude on population: not cirrhosis |
| Sun, Li-Yang, Wang, Nan-Ya, Diao, Yong-Kang, Yan, Cun-Lin, Fan, Zhu-Ping, Wei, Lian-Hua, et al. Comparison between models for detecting hepatocellular carcinoma in patients with chronic liver diseases of various etiologies: ASAP score versus GALAD score. Hepatobiliary & pancreatic diseases international : HBPD INT 2025;24(4):412â€"422. | Does not meet full eligibility criteria: includes people without cirrhosis |
| Sun, Na, Lee, Yi-Te, Zhang, Ryan Y., Kao, Rueihung, Teng, Pai-Chi, Yang, Yingying, et al. Purification of HCC-specific extracellular vesicles on nanosubstrates for early HCC detection by digital scoring. Nature communications 2020;11(1):4489. | Does not meet full eligibility criteria: includes people without cirrhosis |
| Sun, Na, Zhang, Ceng, Lee, Yi-Te, Tran, Benjamin V., Wang, Jing, Kim, Hyoyong, et al. HCC EV ECG Score: An Extracellular Vesicle-based Protein Assay for Detection of Early-Stage Hepatocellular Carcinoma. Hepatology (Baltimore, Md.) 2022;():n. pag.. | Does not meet full eligibility criteria: includes people without cirrhosis |

| Reference | Reason for exclusion |
|---|---|
| Sun, Qiyu, Li, Jian, Jin, Boxun, Wang, Tiezheng. Evaluation of miR-331-3p and miR-23b-3p as serum biomarkers for hepatitis c virus-related hepatocellular carcinoma at early stage. Clinics and research in hepatology and gastroenterology 2020;44(1):21-28. | Duplicate (including Cochrane includes) |
| Sun, Qiyu, Li, Jian, Jin, Boxun, Wang, Tiezheng. Evaluation of miR-331-3p and miR-23b-3p as serum biomarkers for hepatitis c virus-related hepatocellular carcinoma at early stage. Clinics and research in hepatology and gastroenterology 2020;44(1):21-28. | Exclude on population: not cirrhosis |
| Sun, Stella, Poon, Ronnie T P, Lee, Nikki P, Yeung, Chun, Chan, K L, Ng, Irene O L, Day, Philip J R. Proteomics of hepatocellular carcinoma: serum vimentin as a surrogate marker for small tumors (. Journal of proteome research 2010;9(4):1923-30. | Exclude on population: not cirrhosis |
| Sun, Stella, Poon, Ronnie T. P., Lee, Nikki P., Yeung, Chun, Chan, K. L., Ng, Irene O. L., Day, Philip J. R. Proteomics of hepatocellular carcinoma: serum vimentin as a surrogate marker for small tumors ( | Duplicate (including Cochrane includes) |
| Sun, Stella, Xu, Michelle Z., Poon, Ronnie T., Day, Philip J. Circulating Lamin B1 (LMNB1) biomarker detects early stages of liver cancer in patients. Journal of proteome research 2010;9(1):70-78. | Does not meet full eligibility criteria: includes people without cirrhosis |
| Sun, Ting, Li, Ruicen, Qiu, Yiwen, Shen, Shu. New Thresholds for AFP and Des-gamma-Carboxy Prothrombin in Chronic Liver Disease Depending on the Use of Nucleoside Analogs and an Integrated Nomogram. International journal of general medicine 2021;14():6149-65. | Exclude on population: not cirrhosis |
| Sun, Wei, Xing, Baocai, Guo, Lihai, Liu, Zhilei, Mu, Jinsong, Sun, Longqin, et al. Quantitative Proteomics Analysis of Tissue Interstitial Fluid for Identification of Novel Serum Candidate Diagnostic Marker for Hepatocellular Carcinoma. Scientific reports 2016;6():26499. | Exclude on index test |
| Sun, Xiao-Lan, Yao, Hui, Men, Qiong, Hou, Ke-Zhu, Chen, Zhen, Xu, Chang-Qing. Combination of acoustic radiation force impulse imaging, serological indexes and contrast-enhanced ultrasound for diagnosis of liver lesions. World journal of gastroenterology 2017;23(30):5602-09. | Exclude on population: clinical signs and symptoms of HCC |
| Sun, Xinfeng, Feng, Wenxing, Cui, Pin, Ruan, Ruyun, Ma, Wenfeng, Han, Zhiyi, et al. Detection and monitoring of HBV-related hepatocellular carcinoma from plasma cfDNA fragmentation profiles. Genomics 2022;114(6):110502. | Does not meet full eligibility criteria: includes people without cirrhosis |
| Sun, Yifan, Zhu, Shengbo, Wu, Zhitong, Huang, Yiyong, Liu, Chunming, Tang, Shifu. Elevated serum visfatin levels are associated with poor prognosis of hepatocellular carcinoma. Oncotarget 2017;8(14):23427-35. | Does not meet full eligibility criteria: includes people without cirrhosis |
| Sun, Yulin, Gao, Guangzhou, Cai, Jianqiang, Wang, Youliang, Qu, Xiuhua, He, Lidong, et al. Annexin A2 is a discriminative serological candidate in early hepatocellular carcinoma. Carcinogenesis 2013;34(3):595-604. | No 2x2 data |
| Sun, Z. H., Feng, Y. L., Li, L. H., Yang, Y. Q. Plasma cell-free DNA level and integrity as biomarkers to diagnose hepatocellular carcinoma. Medical Journal of Chinese People's Liberation Army 2016;41(12):1016-19. | Foreign language |
| Sutherland, Tom, Watts, Jane, Ryan, Marno, Galvin, Angela, Temple, Faye, Vuong, Jason. Diffusion-weighted MRI for hepatocellular carcinoma screening in chronic liver disease: Direct comparison with ultrasound screening. Journal of medical imaging and radiation oncology 2017;61(1):34-39. | Exclude on population: not cirrhosis |
| Sutherland, Tom, Watts, Jane, Ryan, Marno, Galvin, Angela, Temple, Faye, Vuong, Jason. Diffusion-weighted MRI for hepatocellular carcinoma screening in chronic liver disease: Direct comparison with ultrasound screening. Journal of medical imaging and radiation oncology 2017;61(1):34-39. | Duplicate (including Cochrane includes) |
| Suzuki, Y., Aoyagi, Y., Mori, S., Suda, T., Naitoh, A., Isokawa, O., et al. Microheterogeneity of serum transferrin in the diagnosis of hepatocellular carcinoma. Journal of Gastroenterology and Hepatology (Australia) 1996;11(4):358-65. | Does not meet full eligibility criteria: pre-2005 |
| Svobodova, Sarka, Karlikova, Marie, Topolcan, Ondrej, Pecen, Ladislav, Pestova, Martina, Kott, Otto, et al. PIVKA-II as a Potential New Biomarker for Hepatocellular Carcinoma - A Pilot Study. In vivo (Athens, Greece) 2018;32(6):1551-54. | Does not meet full eligibility criteria: includes people without cirrhosis |

| Reference | Reason for exclusion |
|---|---|
| Syriha, A., Pantzios, S., Mandilara, D., Galanis, P., Stathopoulou, I., Barla, G. Diagnostic accuracy of serum protein induced by vitamin K absence (PIVKA-II), serum a-fetoprotein and their combination for hepatocellular carcinoma among Caucasian cirrhotic patients with diagnostic or non-diagnostic serum a-fetoprotein levels. Cancer Medicine 2024;13(3):e6825. | No 2x2 data |
| Tabu, Kazuaki, Mawatari, Seiichi, Oda, Kohei, Taniyama, Ohki, Toyodome, Ai, Ijuin, Sho, et al. Highly sensitive Lens culinaris agglutinin-reactive fraction of alpha-fetoprotein is a predictive marker for hepatocarcinogenesis in long-term observation of patients with chronic liver disease. Molecular and clinical oncology 2021;15(3):174. | Exclude on population: not cirrhosis |
| Tacke, Frank, Kanig, Nicolas, En-Nia, Abdelaziz, Kaehne, Thilo, Eberhardt, Christiane S., Shpacovitch, Victoria, Trautwein, Christian. Y-box protein-1/p18 fragment identifies malignancies in patients with chronic liver disease. BMC cancer 2011;11():185. | Does not meet full eligibility criteria: includes people without cirrhosis |
| Tada, T., Kumada, T., Toyoda, H., Kiriyama, S., Tanikawa, M., Hisanaga, Y., et al. HBcrAg predicts hepatocellular carcinoma development: An analysis using time-dependent receiver operating characteristics. Journal of Hepatology 2016;65(1):48-56. | Exclude on population: not cirrhosis |
| Tada, T., Kurosaki, M., Tamaki, N., Yasui, Y., Mori, N., Tsuji, K., et al. General evaluation score for predicting the development of hepatocellular carcinoma in patients with advanced liver fibrosis associated with hepatitis C virus genotype 1 or 2 after direct-acting antiviral therapy. JGH Open 2022;6(7):487-95. | Exclude on study design: not a 1- or 2-gate test accuracy study |
| Taga, H., Hirai, H., Ishizuka, H. Early diagnosis of hepatocellular carcinoma with lectin electrophoresis of serum alpha-fetoprotein. Tumor Biology 1988;9(2-3):110-15. | Exclude on study design: not a 1- or 2-gate test accuracy study |
| Tahir, B., Sandrasegaran, K., Ramaswamy, R., Bertrand, K., Mhapsekar, R., Akisik, F. M. Does the hepatocellular phase of gadobenate dimeglumine help to differentiate hepatocellular carcinoma in cirrhotic patients according to histological grade? Clinical radiology 2011;66(9):845-52. | Exclude on population: clinical signs and symptoms of HCC |
| Tahon, Ahmed M., El-Ghanam, Magdy Z., Zaky, Samy, Emran, Tarek Mostafa, Bersy, Ali M., El-Raey, Fathiya, et al. Significance of Glypican-3 in Early Detection of Hepatocellular Carcinoma in Cirrhotic Patients. Journal of gastrointestinal cancer 2019;50(3):434-41. | Duplicate (including Cochrane includes) |
| Takagi, H. & Abe, T. Serial changes of the tumor markers (ferritin, immunosuppressive acid protein, beta2 microglobulin, sialic acid) after TAE in hepatocellular carcinoma. Japanese Journal of Gastroenterology 1987;84(5):1077-83. | Does not meet full eligibility criteria: pre-2005 |
| Takahashi, H., Saibara, T., Iwamura, S., Tomita, A., Maeda, T., Onishi, S., Yamamoto, Y. Serum alpha-L-fucosidase activity and tumor size in hepatocellular carcinoma. Hepatology (Baltimore, Md.) 1994;19(6):1414-17. | Exclude on population: healthy controls |
| Takaya, Hiroaki, Namisaki, Tadashi, Kitade, Mitsuteru, Kaji, Kosuke, Nakanishi, Keisuke, Tsuji, Yuki, et al. VWF/ADAMTS13 ratio as a potential biomarker for early detection of hepatocellular carcinoma. BMC gastroenterology 2019;19(1):167. | Duplicate (including Cochrane includes) |
| Takaya, Hiroaki, Namisaki, Tadashi, Kitade, Mitsuteru, Shimozato, Naotaka, Kaji, Kosuke, Tsuji, Yuki, et al. Acylcarnitine: Useful biomarker for early diagnosis of hepatocellular carcinoma in non-steatohepatitis patients. World journal of gastrointestinal oncology 2019;11(10):887-97. | Exclude on index test |
| Taketa, K., Endo, Y., Sekiya, C., Tanikawa, K., Koji, T., Taga, H., et al. A collaborative study for the evaluation of lectin-reactive alpha-fetoproteins in early detection of hepatocellular carcinoma. Cancer research 1993;53(22):5419-23. | Does not meet full eligibility criteria: includes people without cirrhosis |
| Taketa, K., Ichikawa, E., Yamamoto, T., Kato, H., Matsuura, S., Taga, H. Datura stramonium agglutinin-reactive alpha-fetoprotein isoforms in hepatocellular carcinoma and other tumors. Tumour biology : the journal of the International Society for Oncodevelopmental Biology and Medicine 1990;11(4):220-28. | Does not meet full eligibility criteria: pre-2005 |
| Taketa, Kazuhisa, Okada, Shigeru, Win, Ne, Hlaing, Naomi Khaing Than. Evaluation of tumor markers for the detection of hepatocellular carcinoma in | Does not meet full eligibility criteria: includes people without cirrhosis |

| Reference | Reason for exclusion |
|---|---|
| Yangon General Hospital, Myanmar. Acta medica Okayama 2002;56(6):317-20. | |
| Takezaki, E., Murakami, S., Nishibayashi, H., Kagawa, K. [A clinical study of complements as a marker of a hepatocellular carcinoma]. Gan no rinsho. Japan journal of cancer clinics 1990;36(12):2119-22. | Does not meet full eligibility criteria: pre-2005 |
| Takikawa, Y, Suzuki, K, Yamazaki, K, Goto, T, Madarame, T, Miura, Y, et al. Plasma abnormal prothrombin (PIVKA-II): a new and reliable marker for the detection of hepatocellular carcinoma. Journal of gastroenterology and hepatology 1992;7(1):1-6. | Does not meet full eligibility criteria: includes people without cirrhosis |
| Takikawa, Y., Suzuki, K., Yamazaki, K., Goto, T., Madarame, T., Miura, Y., et al. Plasma abnormal prothrombin (PIVKA-PI): A new and reliable marker for the detection of hepatocellular carcinoma. Journal of Gastroenterology and Hepatology 1992;7(1):1-6. | Does not meet full eligibility criteria: pre-2005 |
| Talbot, J. N., Fartoux, L., Balogova, S., Nataf, V., Kerrou, K., Gutman, F., et al. Detection of hepatocellular carcinoma with PET/CT: A prospective comparison of 18F-fluorocholine and 18F-FDG in patients with cirrhosis or chronic liver disease. Journal of Nuclear Medicine 2010;51(11):1699-7006. | Exclude on population: HCC participants not treatment-naive |
| Talbot, Jean-Noel, Fartoux, Laetitia, Balogova, Sona, Nataf, Valerie, Kerrou, Khaldoun, Gutman, Fabrice, et al. Detection of hepatocellular carcinoma with PET/CT: a prospective comparison of 18F-fluorocholine and 18F-FDG in patients with cirrhosis or chronic liver disease. Journal of nuclear medicine : official publication, Society of Nuclear Medicine 2010;51(11):1699-7006. | Exclude on population: clinical signs and symptoms of HCC |
| Taleb, I., Thiefin, G., Gobinet, C., Untereiner, V., Bernard-Chabert, B., Heurgue, A., et al. Diagnosis of hepatocellular carcinoma in cirrhotic patients: a proof-of-concept study using serum micro-Raman spectroscopy. The Analyst 2013;138(14):4006-14. | Exclude on index test |
| Talkahn M., El Sayed E.Y., Fouad M.H.A. Evaluation of serum endoglin level as a new diagnostic biomarker for hepatocellular carcinoma. United European Gastroenterology Journal 2018;6(8 Supplement):A157. | CONFERENCE ABSTRACT |
| Tamano, M., Kuniyoshi, T., Katayama, Y., Takada, H. Positioning of novel tumor marker NX-PVKA-R in the diagnosis of hepatocellular carcinoma in comparison with PIVKA-II. Dokkyo Journal of Medical Sciences 2013;40(3):163-68. | Exclude on population: not cirrhosis |
| Tameda, Masahiko, Shiraki, Katsuya, Sugimoto, Kazushi, Ogura, Suguru, Inagaki, Yuji, Yamamoto, Norihiko, et al. Des-gamma-carboxy prothrombin ratio measured by P-11 and P-16 antibodies is a novel biomarker for hepatocellular carcinoma. Cancer science 2013;104(6):725-31. | Exclude on population: HCC participants not treatment-naive |
| Tamura, A., Oita, T., Sakizono, K., Nakajima, T. [Clinical usefulness of lectin-reactive fraction of alpha-fetoprotein in hepatocellular carcinoma]. Rinsho byori. The Japanese journal of clinical pathology 1998;46(2):158-62. | Does not meet full eligibility criteria: pre-2005 |
| Tamura, S., Fujioka, H., Nakano, T., Amuro, Y., Hada, T., Nakao, N. Urinary pseudouridine as a biochemical marker in the diagnosis and monitoring of primary hepatocellular carcinoma. The American journal of gastroenterology 1988;83(8):841-45. | Does not meet full eligibility criteria: pre-2005 |
| Tan, Chang, Cao, Jingyi, Chen, Lu, Xi, Xiaochen, Wang, Siqi, Zhu, Yumin, et al. Noncoding RNAs Serve as Diagnosis and Prognosis Biomarkers for Hepatocellular Carcinoma. Clinical chemistry 2019;65(7):905-15. | Exclude on population: not cirrhosis |
| Tan, Yexiong, Yin, Peiyuan, Tang, Liang, Xing, Wenbin, Huang, Qiang, Cao, Dan, et al. Metabolomics study of stepwise hepatocarcinogenesis from the model rats to patients: potential biomarkers effective for small hepatocellular carcinoma diagnosis. Molecular & cellular proteomics : MCP 2012;11(2):M111.010694. | Does not meet full eligibility criteria: includes people without cirrhosis |
| Tan, Yexiong, Yin, Peiyuan, Tang, Liang, Xing, Wenbin, Huang, Qiang, Cao, Dan, et al. Metabolomics study of stepwise hepatocarcinogenesis from the model rats to patients: potential biomarkers effective for small hepatocellular carcinoma diagnosis. Molecular & cellular proteomics : MCP 2012;11(2):M111.010694. | Duplicate (including Cochrane includes) |
| Tan, Youwen, Ge, Guohong, Pan, Tengli, Wen, Danfeng, Chen, Li, Yu, Xuejun, Zhou, Xinbei. A serum microRNA panel as potential biomarkers for | Does not meet full eligibility criteria: includes people without cirrhosis |

| Reference | Reason for exclusion |
|---|---|
| hepatocellular carcinoma related with hepatitis B virus. PloS one 2014;9(9):e107986. | |
| Tanabe, Kazuhiro, Kitagawa, Kae, Kojima, Nozomi. Multifucosylated Alpha-1-acid Glycoprotein as a Novel Marker for Hepatocellular Carcinoma. Journal of proteome research 2016;15(9):2935-44. | Exclude on index test |
| Tanabe, Masahiro, Kanki, Akihiko, Wolfson, Tanya, Costa, Eduardo A. C., Mamidipalli, Adrija, Ferreira, Marilia P. F. D., et al. Imaging Outcomes of Liver Imaging Reporting and Data System Version 2014 Category 2, 3, and 4 Observations Detected at CT and MR Imaging. Radiology 2016;281(1):129-39. | Exclude on population: clinical signs and symptoms of HCC |
| Tanaka, S, Kitamura, T, Ohshima, A, Umeda, K, Okuda, S, Ohtani, T, Tatsuta, M. Diagnostic accuracy of ultrasonography for hepatocellular carcinoma. Cancer 1986;58(2):344-7. | Exclude on population: not cirrhosis |
| Tanaka, S., Kitamura, T., Ohshima, A., Umeda, K., Okuda, S., Ohtani, T., Tatsuta, M. Diagnostic accuracy of ultrasonography for hepatocellular carcinoma. Cancer 1986;58(2):344-47. | Exclude on population: not cirrhosis |
| Tanaka, Takahiro, Taniguchi, Tatsuya, Sannomiya, Katsutaka, Takenaka, Hidetaka, Tomonari, Tetsu, Okamoto, Koichi, et al. Novel des-gamma-carboxy prothrombin in serum for the diagnosis of hepatocellular carcinoma. Journal of gastroenterology and hepatology 2013;28(8):1348-55. | Exclude on population: not cirrhosis |
| Tanaka, Y., Kashiwagi, T., Tsutsumi, H., Nagasawa, M., Toyama, T., Ozaki, S., et al. Sensitive measurement of serum abnormal prothrombin (PIVKA-II) as a marker of hepatocellular carcinoma. Hepato-gastroenterology 1999;46(28):2464-68. | Does not meet full eligibility criteria: pre-2005 |
| Tang X.-Q., Li H., Yan L.-B., Zhou L.-Y., Chen E.-Q., Liu M., Zhang D.-M. Diagnostic value of PIVKA-II in detecting hepatocellular carcinoma. Future Virology 2017;12(5):259-67. | Does not meet full eligibility criteria: includes people without cirrhosis |
| Tang, Si Ying, Xu, Ying, Jiao, Cong Cong, Jiang, Meng Hui, Kong, Nan, Ding, Hao, Cui, Lian Hua. Clinical significance of cylindromatosis expression in primary hepatocellular carcinoma. Arab journal of gastroenterology : the official publication of the Pan-Arab Association of Gastroenterology 2023;24(1):58-64. | Does not meet full eligibility criteria: includes people without cirrhosis |
| Tang, X. Q., Li, H., Yan, L. B., Zhou, L. Y., Chen, E. Q., Liu, M., Zhang, D. M. Diagnostic value of PIVKA-II in detecting hepatocellular carcinoma. Future Virology 2017;12(5):259-67. | Duplicate (including Cochrane includes) |
| Tang, X., Wang, D., Ding, T., Lin, R., He, M., Wang, R. Assessment of combined serum sST2 and AFP levels in the diagnosis of hepatocellular carcinoma. PeerJ 2024;12(10):e18142. | Exclude on population: not cirrhosis |
| Tang, Y. & Xie, S. Diagnostic efficacy of alpha-fetoprotein and alpha-fetoprotein L3% in hepatitis B virus-related early-stage hepatocellular carcinoma. Journal of Clinical Hepatology 2023;39(11):2607 “ 2613. | Foreign language |
| Tang, Y., Li, K., Cai, Z., Xie, Y., Tan, X., Su, C. HSP90alpha combined with AFP and TK1 improved the diagnostic value for hepatocellular carcinoma. Biomarkers in Medicine 2020;14(10):869-78. | Exclude on population: not cirrhosis |
| Tanggo, Y. & Fujiyama, S. Fundamental study of enzyme immunoassay of alpha-fetoprotein and its clinical application. Kumamoto Medical Journal 1982;35(3):95-101. | Does not meet full eligibility criteria: pre-2005 |
| Tangkijvanich, P., Tosukhowong, P., Bunyongyod, P., Lertmaharit, S., Hanvivatvong, O., Kullavanijaya, P. Alpha-L-fucosidase as a serum marker of hepatocellular carcinoma in Thailand. The Southeast Asian journal of tropical medicine and public health 1999;30(1):110-14. | Exclude on population: not cirrhosis |
| Tangkijvanich, Pisit, Chanmee, Theerawut, Komtong, Sanpoj, Mahachai, Varocha, Wisedopas, Naruemon, Pothacharoen, Peraphan. Diagnostic role of serum glypican-3 in differentiating hepatocellular carcinoma from non-malignant chronic liver disease and other liver cancers. Journal of gastroenterology and hepatology 2010;25(1):129-37. | Exclude on population: not cirrhosis |
| Tangkijvanich, Pisit, Chanmee, Theerawut, Komtong, Sanpoj, Mahachai, Varocha, Wisedopas, Naruemon, Pothacharoen, Peraphan. Diagnostic role of serum glypican-3 in differentiating hepatocellular carcinoma from non-malignant chronic liver disease and other liver cancers. Journal of gastroenterology and hepatology 2010;25(1):129-37. | Duplicate (including Cochrane includes) |

| Reference | Reason for exclusion |
|---|---|
| Tao, Kaishan, Bian, Zhenyuan, Zhang, Qiong, Guo, Xu, Yin, Chun, Wang, Yang, et al. Machine learning-based genome-wide interrogation of somatic copy number aberrations in circulating tumor DNA for early detection of hepatocellular carcinoma. EBioMedicine 2020;56():102811. | Does not meet full eligibility criteria: includes people without cirrhosis |
| Tao, Li-Ping, Fan, Xiao-Peng, Fan, Yu-Chen, Zhao, Jing, Gao, Shuai. Combined detection of insulin-like growth factor-binding protein 7 promoter methylation improves the diagnostic efficacy of AFP in hepatitis B virus-associated hepatocellular carcinoma. Pathology, research and practice 2018;214(1):144-50. | Exclude on population: not cirrhosis |
| Taouli, Bachir, Losada, Mariela, Holland, Agnes. Magnetic resonance imaging of hepatocellular carcinoma. Gastroenterology 2004;127(5 Suppl 1):S144-52. | Exclude on study design: not a 1- or 2-gate test accuracy study |
| Taourel, P. G., Pageaux, G. P., Coste, V., Fabre, J. M., Pradel, J. A., Ramos, J., et al. Small hepatocellular carcinoma in patients undergoing liver transplantation: detection with CT after injection of iodized oil. Radiology 1995;197(2):377-80. | Does not meet full eligibility criteria: pre-2005 |
| Tarek, M., Louka, M. L., Khairy, E., Ali-Labib, R., Zaky, D. Z. Role of microRNA-7 and selenoprotein P in hepatocellular carcinoma. Tumor Biology 2017;39(5):n. pag.. | Does not meet full eligibility criteria: includes people without cirrhosis |
| Tat Trung, Ngo, Duong, Dang Chieu, Tong, Hoang Van, Hien, Tran Thi Thu, Hoan, Phan Quoc, Bang, Mai Hong, et al. Optimisation of quantitative miRNA panels to consolidate the diagnostic surveillance of HBV-related hepatocellular carcinoma. PloS one 2018;13(4):e0196081. | No 2x2 data |
| Tatsuma, T., Goto, S., Kitano, S., Lin, Y. C., Lee, C. M. Telomerase activity in peripheral blood for diagnosis of hepatoma. Journal of gastroenterology and hepatology 2000;15(9):1064-70. | Exclude on index test |
| Tatsuta, M. & Yamamura, H. Value of serum alpha-fetoprotein and ferritin in the diagnosis of hepatocellular carcinoma. Oncology 1986;43(5):306-10. | Exclude on population: not cirrhosis |
| Taura, Naota, Fukuda, Sachiko, Ichikawa, Tatsuki, Miyaaki, Hisamitsu, Shibata, Hidetaka, Honda, Takuya, et al. Relationship of alpha-fetoprotein levels and development of hepatocellular carcinoma in hepatitis C patients with liver cirrhosis. Experimental and therapeutic medicine 2012;4(6):972-76. | No 2x2 data |
| Taylor, K. J. W., Gorelick, F. S., Rosenfield, A. T. Ultrasonography of alcoholic liver disease with histological correlation. Radiology 1981;141(1):157-61. | Does not meet full eligibility criteria: pre-2005 |
| Tayob, Nabihah & Lok, Anna S. F. A multivariate parametric empirical Bayes screening approach for early detection of hepatocellular carcinoma using multiple longitudinal biomarkers. Statistics in medicine 2022;41(13):2338-53. | Exclude on population: not cirrhosis |
| Tayob, Nabihah, Christie, Israel, Richardson, Peter, Feng, Ziding, White, Donna L, Davila, Jessica, et al. Validation of the Hepatocellular Carcinoma Early Detection Screening (HES) Algorithm in a Cohort of Veterans With Cirrhosis. Clinical gastroenterology and hepatology : the official clinical practice journal of the American Gastroenterological Association 2019;17(9):1886-93.e5e5. | Continuous test without threshold |
| Tayob, Nabihah, Christie, Israel, Richardson, Peter, Feng, Ziding, White, Donna L., Davila, Jessica, et al. Validation of the Hepatocellular Carcinoma Early Detection Screening (HES) Algorithm in a Cohort of Veterans With Cirrhosis. Clinical gastroenterology and hepatology : the official clinical practice journal of the American Gastroenterological Association 2019;17(9):1886-93.e5e5. | Duplicate (including Cochrane includes) |
| Tayob, Nabihah, Corley, Douglas A., Christie, Israel, Almers, Lucy, Rahal, Ahmed K., Richardson, Peter, et al. Validation of the Updated Hepatocellular Carcinoma Early Detection Screening Algorithm in a Community-Based Cohort of Patients With Cirrhosis of Multiple Etiologies. Clinical gastroenterology and hepatology : the official clinical practice journal of the American Gastroenterological Association 2021;19(7):1443-50.e6e6. | Exclude on index test |
| Tayob, Nabihah, Kanwal, Fasiha, Alsarraj, Abeer, Hernaez, Ruben. The Performance of AFP, AFP-3, DCP as Biomarkers for Detection of Hepatocellular Carcinoma (HCC): A Phase 3 Biomarker Study in the United States. Clinical gastroenterology and hepatology : the official clinical practice | Exclude on study design: not a 1- or 2-gate test accuracy study |

| Reference | Reason for exclusion |
|---|---|
| journal of the American Gastroenterological Association 2023;21(2):415-23.ee4. | |
| Tayob, Nabihah, Lok, Anna S F, Do, Kim-Anh. Improved Detection of Hepatocellular Carcinoma by Using a Longitudinal Alpha-Fetoprotein Screening Algorithm. Clinical gastroenterology and hepatology : the official clinical practice journal of the American Gastroenterological Association 2016;14(3):469-75.ee2. | Secondary publication of included study |
| Tayob, Nabihah, Lok, Anna S. F., Do, Kim-Anh. Improved Detection of Hepatocellular Carcinoma by Using a Longitudinal Alpha-Fetoprotein Screening Algorithm. Clinical gastroenterology and hepatology : the official clinical practice journal of the American Gastroenterological Association 2016;14(3):469-75.ee2. | Duplicate (including Cochrane includes) |
| Tayob, Nabihah, Stingo, Francesco, Do, Kim-Anh, Lok, Anna S. F. A Bayesian screening approach for hepatocellular carcinoma using multiple longitudinal biomarkers. Biometrics 2018;74(1):249-59. | Secondary publication of included study |
| Teama, S. H., Agwa, S. H. A., ElNakeeb, N. A., Hamid, M. A., Maher, A. M. Multiple molecular markers MAGE-1, MAGE-3 and AFP mRNAs expression nested PCR assay for sensitive and specific detection of circulating hepatoma cells: Enhanced detection of hepatocellular carcinoma. Egyptian Journal of Medical Human Genetics 2013;14(1):21-28. | Does not meet full eligibility criteria: includes people without cirrhosis |
| Teefey, Sharlene A, Hildeboldt, Charles C, Dehdashti, Farrokh, Siegel, Barry A, Peters, Marion G, Heiken, Jay P, et al. Detection of primary hepatic malignancy in liver transplant candidates: prospective comparison of CT, MR imaging, US, and PET. Radiology 2003;226(2):533-42. | Exclude on population: clinical signs and symptoms of HCC |
| Teefey, Sharlene A., Hildeboldt, Charles C., Dehdashti, Farrokh, Siegel, Barry A., Peters, Marion G., Heiken, Jay P., et al. Detection of primary hepatic malignancy in liver transplant candidates: prospective comparison of CT, MR imaging, US, and PET. Radiology 2003;226(2):533-42. | Exclude on population: clinical signs and symptoms of HCC |
| Teerasamit, Wanwarang, Saiviroonporn, Pairash, Pongpaibul, Ananya. Benefit of double contrast MRI in diagnosis of hepatocellular carcinoma in patients with chronic liver diseases. Journal of the Medical Association of Thailand = Chotmaihet thangphaet 2014;97(5):540-47. | Exclude on population: not cirrhosis |
| Teng, M., Pirrie, S., Ward, D. G., Assi, L. K., Hughes, R. G., Stocken, D. Diagnostic and mechanistic implications of serum free light chains, albumin and alpha-fetoprotein in hepatocellular carcinoma. British journal of cancer 2014;110(9):2277-82. | Exclude on population: not cirrhosis |
| Teng, W., Li, H., Yang, H., Chen, Y., Xi, L., Xin, F., et al. Discovery and validation of a novel dual-target blood test for the detection of hepatocellular carcinoma across stages from cirrhosis. BMC Medicine 2025;23(1):278. | Exclude on index test |
| Teng, Y, Fan, Y C, Mu, N N, Zhao, J, Sun, F K. Serum SOX11 promoter methylation is a novel biomarker for the diagnosis of Hepatitis B virus-related hepatocellular carcinoma. Neoplasma 2016;63(3):419-26. | Exclude on population: not cirrhosis |
| Teng, Y., Fan, Y. C., Mu, N. N., Zhao, J., Sun, F. K. Serum SOX11 promoter methylation is a novel biomarker for the diagnosis of Hepatitis B virus-related hepatocellular carcinoma. Neoplasma 2016;63(3):419-26. | Duplicate (including Cochrane includes) |
| Thanapirom, K., Suksawatamnuay, S., Thaimai, P., Siripon, N., Geratikornsupuk, N., Treeprasertsuk, S. Comparison of the GALAD, GAAP, and ASAP Scores for Hepatocellular Carcinoma Detection in Patients With Chronic Liver Diseases. Journal of Clinical and Experimental Hepatology 2025;15(6):102607. | Does not meet full eligibility criteria: includes people without cirrhosis |
| Tian, Liyuan, Wang, Yu, Xu, Dabin, Gui, Junhao, Jia, Xingwang, Tong, Hongli, et al. Serological AFP/Golgi protein 73 could be a new diagnostic parameter of hepatic diseases. International journal of cancer 2011;129(8):1923-31. | Does not meet full eligibility criteria: includes people without cirrhosis |
| Tian, Ming-Ming, Fan, Yu-Chen, Zhao, Jing, Gao, Shuai, Zhao, Ze-Hua, Chen, Long-Yan. Hepatocellular carcinoma suppressor 1 promoter hypermethylation in serum. A diagnostic and prognostic study in hepatitis B. Clinics and research in hepatology and gastroenterology 2017;41(2):171-80. | Exclude on population: not cirrhosis |
| Tian, Ming-Ming, Fan, Yu-Chen, Zhao, Jing, Gao, Shuai, Zhao, Ze-Hua, Chen, Long-Yan. Hepatocellular carcinoma suppressor 1 promoter hypermethylation | Duplicate (including Cochrane includes) |

| Reference | Reason for exclusion |
|---|---|
| in serum. A diagnostic and prognostic study in hepatitis B. Clinics and research in hepatology and gastroenterology 2017;41(2):171-80. | |
| Tian, Shan, Chen, Yongyi, Zhang, Yimin. Clinical value of serum AFP and PIVKA-II for diagnosis, treatment and prognosis of hepatocellular carcinoma. Journal of clinical laboratory analysis 2023;37(1):e24823. | Exclude on population: not cirrhosis |
| Tian, Z. B., Liu, H., Sun, G. R., Kong, X. J., Zhang, C. P. Application of surface enhanced laser desorption ionization time-of-flight mass spectrometry technology in the diagnosis of hepatocellular carcinoma. World Chinese Journal of Digestology 2006;14(25):2499-5003. | Foreign language |
| Tian, Zeyu, Yu, Tanbo, Wei, Hongyan. Clinical value of LHPP-associated microRNAs combined with protein induced by vitamin K deficiency or antagonist-II in the diagnosis of alpha-fetoprotein-negative hepatocellular carcinoma. Journal of clinical laboratory analysis 2020;34(2):e23071. | Exclude on population: healthy controls |
| Tillman, B. G., Gorman, J. D., Hru, J. M., Lee, M. H., King, M. C., Sirlin, C. B. Diagnostic per-lesion performance of a simulated gadoxetate disodium-enhanced abbreviated MRI protocol for hepatocellular carcinoma screening. Clinical radiology 2018;73(5):485-93. | Does not meet full eligibility criteria: includes people without cirrhosis |
| Tiyarattanachai, T. & Fetzer, D. T. Multicenter Study of ACR Ultrasound LI-RADS Visualization Scores on Serial Examinations: Implications for Changes in Surveillance Strategies. AJR. American journal of roentgenology 2022;():n. pag.. | Exclude on population: not cirrhosis |
| Tomimaru, Yoshito, Eguchi, Hidetoshi, Nagano, Hiroaki, Wada, Hiroshi, Kobayashi, Shogo, Marubashi, Shigeru, et al. Circulating microRNA-21 as a novel biomarker for hepatocellular carcinoma. Journal of hepatology 2012;56(1):167-75. | Exclude on population: not cirrhosis |
| Tomiya, T. Serum transforming growth factor alpha level as a marker of hepatocellular carcinoma complicating cirrhosis. Cancer 1996;77(6):1056-60. | Exclude on index test |
| Tong, H. B., Ting, Y. J., Luo, L. M. Value of alpha fetoprotein, Dickkopf1 glycoprotein and alpha-L - Fucosidase in early diagnosis of liver cancer. World Chinese Journal of Digestology 2014;22(24):3670-74. | Foreign language |
| Tong, L., Gao, Z., Huang, C., Feng, H., Sun, X., Ji, J., et al. Role of GALAD serological model in the clinical diagnosis of primary hepatocellular carcinoma. Chinese Journal of Laboratory Medicine 2019;42(12):1037-41. | Foreign language |
| Tong, M J & Blatt, L M. Surveillance for hepatocellular carcinoma in patients with chronic viral hepatitis in the United States of America. Journal of gastroenterology and hepatology 2001;16(5):553-9. | Exclude on population: not cirrhosis |
| Tong, M. J. & Blatt, L. M. Surveillance for hepatocellular carcinoma in patients with chronic viral hepatitis in the United States of America. Journal of Gastroenterology and Hepatology (Australia) 2001;16(5):553-59. | Duplicate (including Cochrane includes) |
| Toraih, E. A., Ellawindy, A., Fala, S. Y., Al Ageeli, E., Gouda, N. S., Fawzy, M. S. Oncogenic long noncoding RNA MALAT1 and HCV-related hepatocellular carcinoma. Biomedicine and Pharmacotherapy 2018;102():653-69. | Duplicate (including Cochrane includes) |
| Toyoda, H., Kumada, T., Osaki, Y., Tada, T., Kaneoka, Y. Novel method to measure serum levels of des-gamma-carboxy prothrombin for hepatocellular carcinoma in patients taking warfarin: A preliminary report. Cancer Science 2012;103(5):921-25. | Exclude on population: not cirrhosis |
| Toyoda, Hidenori, Kikuchi, Kan, Tsuruta, Yuki, Hiraoka, Atsushi, Tsuji, Kunihiko. Utility of serological tumor biomarkers for surveillance of hepatocellular carcinoma in patients undergoing dialysis. Nephrology, dialysis, transplantation : official publication of the European Dialysis and Transplant Association - European Renal Association 2021;36(6):1097-1003. | Exclude on population: not cirrhosis |
| Toyoda, Hidenori, Kumada, Takashi, Tada, Toshifumi, Kaneoka, Yuji, Maeda, Atsuyuki, Kanke, Futoshi. Clinical utility of highly sensitive Lens culinaris agglutinin-reactive alpha-fetoprotein in hepatocellular carcinoma patients with alpha-fetoprotein | Exclude on population: not cirrhosis |
| Tran, T. H., Pham, N. T., Nguyen, V. T. C., Vo, D. H., Nguyen, T. H. H., Tran, T. T., et al. Supervised non-negative matrix factorization on cell-free DNA fragmentomic features enhances early cancer detection. bioRxiv 2024;():n. pag.. | CONFERENCE ABSTRACT |

| Reference | Reason for exclusion |
|---|---|
| Tran, Vinh Thanh, Phan, Thang Thanh, Nguyen, Tran Bao, Le, Thao Thi, Tran, Thanh-Tram Thi, Nguyen, Anh-Thu Thi, et al. The diagnostic performance of AFP and PIVKA-II models for non-B non-C hepatocellular carcinoma. BMC research notes 2023;16(1):317. | Exclude on population: not cirrhosis |
| Tran, Y. H., Dao, T. T., Nguyen, U. D., Tran, T. B., Luu, L. P., Duong, H. Q. Sensitive detection of circulating methylated SEPT9 in hepatocellular carcinoma patients using a novel quantitative PCR assay. Analytical methods : advancing methods and applications 2025;17(9):2181 “ 2190. | Exclude on population: not cirrhosis |
| Tremolda, F, Benevegnu, L, Drago, C, Casarin, C, Cechetto, A, Realdi, G. Early detection of hepatocellular carcinoma in patients with cirrhosis by alphafetoprotein, ultrasound and fine-needle biopsy. Hepato-gastroenterology 1989;36(6):519-21. | Does not meet full eligibility criteria: pre-2005 |
| Tremolda, F., Benevegnu, L., Drago, C., Casarin, C., Cechetto, A., Realdi, G. Early detection of hepatocellular carcinoma in patients with cirrhosis by alphafetoprotein, ultrasound and fine-needle biopsy. Hepato-Gastroenterology 1989;36(6):519-21. | Does not meet full eligibility criteria: pre-2005 |
| Trevisani, F, D'Intino, P E, Morselli-Labate, A M, Mazzella, G, Accogli, E, Caraceni, P, et al. Serum alpha-fetoprotein for diagnosis of hepatocellular carcinoma in patients with chronic liver disease: influence of HBsAg and anti-HCV status. Journal of hepatology 2001;34(4):570-5. | Does not meet full eligibility criteria: includes people without cirrhosis |
| Trevisani, Franco, Santi, Valentina, Gramenzi, Annagiulia, Di Nolfo, Maria Anna, Del Poggio, Paolo, Benvegnu, Luisa, et al. Surveillance for early diagnosis of hepatocellular carcinoma: is it effective in intermediate/advanced cirrhosis? The American journal of gastroenterology 2007;102(11):2448-58. | Exclude on study design: not a 1- or 2-gate test accuracy study |
| Trung, Ngo Tat, Hoan, Nghiem Xuan, Trung, Pham Quang, Binh, Mai Thanh, Van Tong, Hoang, Toan, Nguyen Linh, Bang, Mai Hong. Clinical significance of combined circulating TERT promoter mutations and miR-122 expression for screening HBV-related hepatocellular carcinoma. Scientific reports 2020;10(1):8181. | No 2x2 data |
| Truong, Bui Xuan, Yano, Yoshihiko, Van, Vu Tuong, Seo, Yasushi, Nam, Nguyen Hoai, Trach, Nguyen Khanh, et al. Clinical utility of protein induced by vitamin K absence in patients with chronic hepatitis B virus infection. Biomedical reports 2013;1(1):122-28. | Exclude on population: healthy controls |
| Tsai J.-F., Yi M.L., Chen S.-C., Lin Z.-Y., Yu M.-L., Dai C.-Y., Chuang W.-L. The optimal cutoff value of serum alpha-fetoprotein in the diagnosis of hepatocellular carcinoma. Journal of Hepatology 2017;66(1 Supplement 1):S454. | CONFERENCE ABSTRACT |
| Tsai, J F, Jeng, J E, Chuang, L Y, Yang, M L, Ho, M S, Chang, W Y, et al. Clinical evaluation of urinary transforming growth factor-beta1 and serum alpha-fetoprotein as tumour markers of hepatocellular carcinoma. British journal of cancer 1997;75(10):1460-6. | Does not meet full eligibility criteria: pre-2005 |
| Tsai, J F, Jeng, J E, Ho, M S, Chang, W Y, Lin, Z Y. Clinical evaluation of serum alpha-fetoprotein and circulating immune complexes as tumour markers of hepatocellular carcinoma. British journal of cancer 1995;72(2):442-6. | Does not meet full eligibility criteria: pre-2005 |
| Tsai, J. F., Chuang, L. Y., Jeng, J. E., Yang, M. L., Chang, W. Y., Hsieh, M. Y., Lin, Z. Y. Clinical relevance of transforming growth factor-beta 1 in the urine of patients with hepatocellular carcinoma. Medicine 1997;76(3):213-26. | Does not meet full eligibility criteria: pre-2005 |
| Tsai, J. F., Jeng, J. E., Chuang, L. Y., Chang, W. Y., Hsieh, M. Y., Lin, Z. Y. Urinary transforming growth factor-beta 1 in relation to serum alpha-fetoprotein in hepatocellular carcinoma. Scandinavian journal of gastroenterology 1997;32(3):254-60. | Exclude on population: not cirrhosis |
| Tsai, J. F., Jeng, J. E., Chuang, L. Y., Yang, M. L., Ho, M. S., Chang, W. Y., et al. Clinical evaluation of urinary transforming growth factor-beta1 and serum alpha-fetoprotein as tumour markers of hepatocellular carcinoma. British journal of cancer 1997;75(10):1460-66. | Does not meet full eligibility criteria: pre-2005 |
| Tsai, J. F., Jeng, J. E., Chuang, L. Y., Yang, M. L., Ho, M. S., Chang, W. Y., et al. Elevated urinary transforming growth factor-beta1 level as a tumour marker and predictor of poor survival in cirrhotic hepatocellular carcinoma. British Journal of Cancer 1997;76(2):244-50. | Does not meet full eligibility criteria: pre-2005 |

| Reference | Reason for exclusion |
|---|---|
| Tsai, J. F., Jeng, J. E., Chuang, L. Y., You, H. L., Ho, M. S., Lai, C. S., et al. Serum insulin-like growth factor-II and alpha-fetoprotein as tumor markers of hepatocellular carcinoma. Tumour biology : the journal of the International Society for Oncodevelopmental Biology and Medicine 2003;24(6):291-98. | Does not meet full eligibility criteria: pre-2005 |
| Tsai, J. F., Jeng, J. E., Ho, M. S., Chang, W. Y., Lin, Z. Y. Clinical evaluation of serum alpha-fetoprotein and circulating immune complexes as tumour markers of hepatocellular carcinoma. British journal of cancer 1995;72(2):442-46. | Duplicate (including Cochrane includes) |
| Tsai, S. L., Huang, G. T., Yang, P. M., Sheu, J. C., Sung, J. L. Plasma des-gamma-carboxyprothrombin in the early stage of hepatocellular carcinoma. Hepatology (Baltimore, Md.) 1990;11(3):481-88. | Exclude on population: HCC participants not treatment-naive |
| Tsai, Tsung-Heng, Wang, Minkun, Di Poto, Cristina, Hu, Yunli, Zhou, Shiyue, Zhao, Yi, et al. LC-MS profiling of N-Glycans derived from human serum samples for biomarker discovery in hepatocellular carcinoma. Journal of proteome research 2014;13(11):4859-68. | Exclude on index test |
| Tsang, L. L. C., Chen, C. L., Huang, T. L., Chen, T. Y., Ou, H. Y., Eng, H. L., et al. Superparamagnectic iron oxide-enhanced magnetic resonance for tumor surveillance in cirrhotic liver before liver transplantation with explanted liver correlation. Transplantation proceedings 2011;43(5):1674-77. | Exclude on target condition: not HCC |
| Tsuda Y., Fukuda A., Kobayashi H., Itou D., Yoshimoto S., Iwata K. Serum neopterin as a marker for screening of hepatocellular carcinoma. Pteridines 2004;15(4):161-69. | Does not meet full eligibility criteria: pre-2005 |
| Tsuda, Y., Fukuda, A., Kobayashi, H., Itou, D., Yoshimoto, S., Iwata, K. Serum neopterin as a marker for screening of hepatocellular carcinoma. Pteridines 2004;15(4):161-69. | Duplicate (including Cochrane includes) |
| Tsurusaki, Masakatsu, Sofue, Keitaro, Isoda, Hiroyoshi, Okada, Masahiro, Kitajima, Kazuhiro. Comparison of gadoxetic acid-enhanced magnetic resonance imaging and contrast-enhanced computed tomography with histopathological examinations for the identification of hepatocellular carcinoma: a multicenter phase III study. Journal of gastroenterology 2016;51(1):71-79. | Exclude on population: clinical signs and symptoms of HCC |
| Tu, D. G., Wang, S. T., Chang, T. T., Chiu, N. T. The value of serum tissue polypeptide specific antigen in the diagnosis of hepatocellular carcinoma. Cancer 1999;85(5):1039-43. | Exclude on population: not cirrhosis |
| Tung, E. K. K. Significance of serum DKK1 as a diagnostic biomarker in hepatocellular carcinoma. Future Oncology 2012;8(12):1525-28. | Does not meet full eligibility criteria: includes people without cirrhosis |
| Ueda, K., Kitagawa, K., Kadoya, M., Matsui, O., Takashima, T. Detection of hypervascular hepatocellular carcinoma by using spiral volumetric CT: comparison of US and MR imaging. Abdominal imaging 1995;20(6):547-53. | Exclude on population: clinical signs and symptoms of HCC |
| Uenishi, T., Kubo, S., Hirohashi, K., Tanaka, H., Shuto, T., Yamamoto, T. Cytokeratin-19 fragments in serum (CYFRA 21-1) as a marker in primary liver cancer. British journal of cancer 2003;88(12):1894-99. | Exclude on population: not cirrhosis |
| Ueno, M., Takeda, H., Takai, A. Risk factors and diagnostic biomarkers for nonalcoholic fatty liver disease-associated hepatocellular carcinoma: Current evidence and future perspectives. World Journal of Gastroenterology 2022;28(27):3410-21. | Exclude on study design: not a 1- or 2-gate test accuracy study |
| Ungtrakul, T., Mahidol, C., Chun-On, P., Laohapand, C., Siripongsakun, S., Worakitsitisatorn, A., et al. Hepatocellular carcinoma screening and surveillance in 2293 chronic hepatitis B patients in an endemic area. World Journal of Gastroenterology 2016;22(34):7806-12. | Duplicate (including Cochrane includes) |
| Ungtrakul, Teerapat, Mahidol, Chulabhorn, Chun-On, Pattra, Laohapand, Charlie, Siripongsakun, Surachate, Worakitsitisatorn, Akeanong, et al. Hepatocellular carcinoma screening and surveillance in 2293 chronic hepatitis B patients in an endemic area. World journal of gastroenterology 2016;22(34):7806-12. | Exclude on population: not cirrhosis |
| Unic A., Grgurevic I., Derek L., Marijancevic D., Serdar T. AFP-l3 - Screening marker for a hepatocellular carcinoma in patients with alchocolic cirrhosis. Biochimica Clinica 2013;37(SUPPL. 1):S679. | CONFERENCE ABSTRACT |

| Reference | Reason for exclusion |
|---|---|
| Unoura, M., Kaneko, S., Matsushita, E., Shimoda, A., Takeuchi, M., Adachi, H., et al. High-risk groups and screening strategies for early detection of hepatocellular carcinoma in patients with chronic liver disease. Hepato-gastroenterology 1993;40(4):305-10. | Does not meet full eligibility criteria: pre-2005 |
| Uthamalingam, P., Das, A., Behra, A., Kalra, N. Diagnostic Value of Glypican3, Heat Shock Protein 70 and Glutamine Synthetase in Hepatocellular Carcinoma Arising in Cirrhotic and Non-Cirrhotic Livers. Journal of Clinical and Experimental Hepatology 2018;8(2):173-80. | Exclude on index test |
| Valls, Carlos, Cos, Monica, Figueras, Juan, Andia, Eduard, Ramos, Emilio, Sanchez, Anna, Serrano, Teresa. Pretransplantation diagnosis and staging of hepatocellular carcinoma in patients with cirrhosis: value of dual-phase helical CT. AJR. American journal of roentgenology 2004;182(4):1011-17. | Exclude on population: clinical signs and symptoms of HCC |
| van der Meer, A. J., Farid, W. R. R., Sonneveld, M. J., de Ruiter, P. E., Boonstra, A., van Vuuren, A. J., et al. Sensitive detection of hepatocellular injury in chronic hepatitis C patients with circulating hepatocyte-derived microRNA-122. Journal of viral hepatitis 2013;20(3):158-66. | Exclude on target condition: not HCC |
| van Staden, L., Bukofzer, S., Kew, M. C. Differential lectin reactivities of alpha-fetoprotein in hepatocellular carcinoma: diagnostic value when serum alpha-fetoprotein levels are slightly raised. Journal of gastroenterology and hepatology 1992;7(3):260-65. | Does not meet full eligibility criteria: pre-2005 |
| Van Thiel, David H, Yong, Sherri, Li, S David, Kennedy, Marc. The development of de novo hepatocellular carcinoma in patients on a liver transplant list: frequency, size, and assessment of current screening methods. Liver transplantation : official publication of the American Association for the Study of Liver Diseases and the International Liver Transplantation Society 2004;10(5):631-7. | Exclude on population: not cirrhosis |
| Van Thiel, David H., Yong, Sherri, Li, S. David, Kennedy, Marc. The development of de novo hepatocellular carcinoma in patients on a liver transplant list: frequency, size, and assessment of current screening methods. Liver transplantation : official publication of the American Association for the Study of Liver Diseases and the International Liver Transplantation Society 2004;10(5):631-37. | Does not meet full eligibility criteria: pre-2005 |
| Veltri, A., Robba, T., Anselmetti, G. C., Martina, M. C., Regge, D., Grosso, M. Lipiodol CT and liver transplantation. Evaluation of diagnostic accuracy in comparison with pathologic examination. Radiologia Medica 1998;96(1-2):81-86. | Does not meet full eligibility criteria: pre-2005 |
| Venturini, I., Amedei, R., Modonesi, G., Cosenza, R., Miglioli, L., Cioni, G. May plasma cholesterol level be considered a neoplastic marker in liver disease from cirrhosis to hepatocellular carcinoma? Italian journal of gastroenterology and hepatology 1999;31(1):61-65. | Does not meet full eligibility criteria: pre-2005 |
| Vermehren, J., Polta, A., Zimmermann, O., Herrmann, E., Poynard, T., Hofmann, W. P., et al. Comparison of acoustic radiation force impulse imaging with transient elastography for the detection of complications in patients with cirrhosis. Liver International ;():n. pag.. | Exclude on index test |
| Vidili, G., Arru, M., Calvisi, D. F., Meloni, P., Sauchella, A., Turilli, D., et al. Contrast-enhanced ultrasound Liver Imaging Reporting and Data System: Lights and shadows in hepatocellular carcinoma and cholangiocellular carcinoma diagnosis. World Journal of Gastroenterology 2022;28(27):3488-5002. | Exclude on population: clinical signs and symptoms of HCC |
| Vietti Violi, Naik, Fowler, Kathryn J., Sirlin, Claude B. Abbreviated Magnetic Resonance Imaging for HCC Surveillance. Clinical liver disease 2021;17(3):133-38. | Exclude on study design: not a 1- or 2-gate test accuracy study |
| Vietti Violi, Naik, Lewis, Sara, Liao, Joseph, Hulkower, Miriam, Hernandez-Meza, Gabriela, Smith, Katherine, et al. Gadoxetate-enhanced abbreviated MRI is highly accurate for hepatocellular carcinoma screening. European radiology 2020;30(11):6003-13. | Does not meet full eligibility criteria: includes people without cirrhosis |
| Viggiani, Valentina, Palombi, Sara, Gennarini, Giuseppina, D'Ettorre, Gabriella, De Vito, Corrado, Angeloni, Antonio, Frati, Luigi. Protein induced by vitamin K absence or antagonist-II (PIVKA-II) specifically increased in Italian | Exclude on population: not cirrhosis |

| Reference | Reason for exclusion |
|---|---|
| hepatocellular carcinoma patients. Scandinavian journal of gastroenterology 2016;51(10):1257-62. | |
| Villa, E., Donghia, R., Baldaccini, V., Tedesco, C. C., Shahini, E., Cozzolongo, R., et al. GALAD outperforms aMAP and ALBI for predicting HCC in patients with compensated advanced chronic liver disease: A 12-year prospective study. Hepatology Communications 2023;7(10):e0262. | Exclude on study design: not a 1- or 2-gate test accuracy study |
| Villa, Erica, Donghia, Rossella, Baldaccini, Valentina, Tedesco, Calogero C., Shahini, Endrit, Cozzolongo, Raffaele, et al. GALAD outperforms aMAP and ALBI for predicting HCC in patients with compensated advanced chronic liver disease: A 12-year prospective study. Hepatology communications 2023;7(10):n. pag.. | Exclude on study design: not a 1- or 2-gate test accuracy study |
| Villacastin Ruiz, Elena, Caro-Paton Gomez, Agustin, Calero Aguilar, Hermogenes, Perez Saborido, Baltasar, Garcia Pajares, Felix, Sanchez Antolin, Gloria, et al. Review of imaging techniques in the diagnosis of hepatocellular carcinoma in patients who require a liver transplant. European journal of gastroenterology & hepatology 2016;28(4):412-20. | Duplicate (including Cochrane includes) |
| Virmani, Jitendra, Kumar, Vinod, Kalra, Naveen. SVM-based characterization of liver ultrasound images using wavelet packet texture descriptors. Journal of digital imaging 2013;26(3):530-43. | Exclude on index test |
| Vivas-Arceo, C. & Bastidas-Ramirez, B. E. Hepatocellular carcinoma is rarely present in Western Mexico. Hepatology Research 1999;16(1):26-35. | Exclude on population: not cirrhosis |
| Volk, Michael L., Hernandez, Jose C., Su, Grace L., Lok, Anna S. Risk factors for hepatocellular carcinoma may impair the performance of biomarkers: a comparison of AFP, DCP, and AFP-L3. Cancer biomarkers : section A of Disease markers 2007;3(2):79-87. | Duplicate (including Cochrane includes) |
| von Felden, Johann, Garcia-Lezana, Teresa, Dogra, Navneet, Gonzalez-Kozlova, Edgar, Ahsen, Mehmet Eren, Craig, Amanda, et al. Unannotated small RNA clusters associated with circulating extracellular vesicles detect early stage liver cancer. Gut 2021;():n. pag.. | Exclude on population: HCC participants not treatment-naive |
| Vongsuvanh, Roslyn, van der Poorten, David, Iseli, Tristan, Strasser, Simone I, McCaughan, Geoffrey W. Midkine Increases Diagnostic Yield in AFP Negative and NASH-Related Hepatocellular Carcinoma. PloS one 2016;11(5):e0155800. | Does not meet full eligibility criteria: includes people without cirrhosis |
| Vongsuvanh, Roslyn, van der Poorten, David, Iseli, Tristan, Strasser, Simone I., McCaughan, Geoffrey W. Midkine Increases Diagnostic Yield in AFP Negative and NASH-Related Hepatocellular Carcinoma. PloS one 2016;11(5):e0155800. | Duplicate (including Cochrane includes) |
| Voss, B. A., Khandelwal, A., Wells, M. L., Inoue, A., Venkatesh, S. K., Lee, Y. S., Johnson, M. P. Impact of dual-energy 50-keV virtual monoenergetic images on radiologist confidence in detection of key imaging findings of small hepatocellular carcinomas using multiphase liver CT. Acta Radiologica 2021;():n. pag.. | Exclude on population: not cirrhosis |
| Voss, Benjamin A., Khandelwal, Ashish, Wells, Michael L., Inoue, Akitoshi, Venkatesh, Sudhakar K., Lee, Yong S., Johnson, Matthew P. Impact of dual-energy 50-keV virtual monoenergetic images on radiologist confidence in detection of key imaging findings of small hepatocellular carcinomas using multiphase liver CT. Acta radiologica (Stockholm, Sweden : 1987) 2022;63(11):1443-52. | Exclude on study design: not a 1- or 2-gate test accuracy study |
| Waguri, Nobuo, Suda, Takeshi, Nomoto, Minoru, Kawai, Hirokazu, Mita, Yusaku, Kuroiwa, Takashi, et al. Sensitive and specific detection of circulating cancer cells in patients with hepatocellular carcinoma; detection of human telomerase reverse transcriptase messenger RNA after immunomagnetic separation. Clinical cancer research : an official journal of the American Association for Cancer Research 2003;9(8):3004-11. | Does not meet full eligibility criteria: includes people without cirrhosis |
| Wahb, Amany Mohamed Salah Eldin, El Kassas, Mohamed, Khamis, Ahmed Kamal, Elhelbawy, Mostafa, Elhelbawy, Nesreen. Circulating microRNA 9-3p and serum endocan as potential biomarkers for hepatitis C virus-related hepatocellular carcinoma. World journal of hepatology 2021;13(11):1753-65. | Exclude on population: not cirrhosis |
| Waidmann, Oliver, Koberle, Verena, Bettinger, Dominik, Trojan, Jorg, Zeuzem, Stefan, Schultheis, Michael, Kronenberger, Bernd. Diagnostic and prognostic significance of cell death and macrophage activation markers in patients with hepatocellular carcinoma. Journal of hepatology 2013;59(4):769-79. | No 2x2 data |

| Reference | Reason for exclusion |
|---|---|
| Wan, F., Zhu, Y., Wu, F., Huang, X., Chen, Y., Zhou, Y., et al. Retinol-binding protein 4 as a promising serum biomarker for the diagnosis and prognosis of hepatocellular Carcinoma. Translational Oncology 2024;45():101979. | Does not meet full eligibility criteria: includes people without cirrhosis |
| Wan, Lijun, Guo, Li, Hu, Youwen, Huang, Hongyan, Zhang, Moran, Xu, Kedong, et al. Comparing the diagnostic value of serum oligosaccharide chain (G-test) and alpha-fetoprotein for hepatitis B virus-related liver cancer. Clinical biochemistry 2021;89():44-50. | Does not meet full eligibility criteria: includes people without cirrhosis |
| Wang, B. L., Chen, X. P., Zhai, S. P. Clinical significance of mrp gene in primary hepatocellular carcinoma. Hepatobiliary and Pancreatic Diseases International 2003;2(3):397-403. | Does not meet full eligibility criteria: pre-2005 |
| Wang, B., Chen, D., Hu, Z., Cao, M., Xie, Q., Chen, Y., et al. Metabonomic profiles discriminate hepatocellular carcinoma from liver cirrhosis by ultraperformance liquid chromatography-mass spectrometry. Journal of Proteome Research 2012;11(2):1217-27. | Does not meet full eligibility criteria: includes people without cirrhosis |
| Wang, Chaur-Shine, Lin, Chih-Lin, Lee, Hsi-Chang, Chen, Kuan-Yang, Chiang, Ming-Feng, Chen, Hung-Sheng, Lin, Tsung-Jung. Usefulness of serum des-gamma-carboxy prothrombin in detection of hepatocellular carcinoma. World journal of gastroenterology 2005;11(39):6115-9. | Does not meet full eligibility criteria: includes people without cirrhosis |
| Wang, Chaur-Shine, Lin, Chih-Lin, Lee, Hsi-Chang, Chen, Kuan-Yang, Chiang, Ming-Feng, Chen, Hung-Sheng, Lin, Tsung-Jung. Usefulness of serum des-gamma-carboxy prothrombin in detection of hepatocellular carcinoma. World journal of gastroenterology 2005;11(39):6115-19. | Does not meet full eligibility criteria: includes people without cirrhosis |
| Wang, D., Dai, Z., Bai, M., Liu, D., Feng, Y., Sun, Q., et al. Integrating cell-free DNA methylation of SEPT9 and SFRP2 into a machine learning model for early diagnosis of HCC. Biomarkers in Medicine 2025;():n. pag.. | Exclude on population: not cirrhosis |
| Wang, Fang, Liu, Hui, Bai, Youxi, Li, Hui, Wang, Zhonglin. Performance of SPINK1 and SPINK1-based diagnostic model in detection of hepatocellular carcinoma. Journal of clinical laboratory analysis 2021;35(11):e24025. | Does not meet full eligibility criteria: includes people without cirrhosis |
| Wang, Feng, Wang, Jun, Ju, Linlin, Chen, Lin, Cai, Weihua. Diagnostic and prognostic potential of serum miR-132/212 cluster in patients with hepatocellular carcinoma. Annals of clinical biochemistry 2018;55(5):576-82. | Exclude on population: not cirrhosis |
| Wang, Feng, Ying, Houqun, He, Bangshun, Pan, Yuqin, Sun, Huiling. Circulating miR-148/152 family as potential biomarkers in hepatocellular carcinoma. Tumour biology : the journal of the International Society for Oncodevelopmental Biology and Medicine 2016;37(4):4945-53. | Exclude on population: not cirrhosis |
| Wang, Guangrong, Lu, Xiaolan, Du, Qin, Zhang, Guoyuan, Wang, Dongsheng, Wang, Qiang. Diagnostic value of the gamma-glutamyltransferase and alanine transaminase ratio, alpha-fetoprotein, and protein induced by vitamin K absence or antagonist II in hepatitis B virus-related hepatocellular carcinoma. Scientific reports 2020;10(1):13519. | Exclude on population: not cirrhosis |
| Wang, H. & Mu, X. T. Comparative study of dynamic MRI and MSCT in hepatocellular carcinoma with cirrhosis. Chinese Journal of Medical Imaging Technology 2007;23(7):1046-48. | Foreign language |
| Wang, H., Dong, J., Bao, J. F., Wang, C. B. [Analysis of the diagnostic efficiency of combining multiple laboratory hematological indicators in alpha-fetoprotein-negative hepatocellular carcinoma]. Zhonghua yi xue za zhi 2022;102(17):1303-10. | Foreign language |
| Wang, H., Xiang, Y., Li, X., Liu, S. High lymphocyte-to-monocyte ratio is associated with low alpha-fetoprotein expression in patients with hepatitis B virus-associated hepatocellular carcinoma. Molecular Medicine Reports 2020;22(4):2673-84. | Exclude on index test |
| Wang, Hongwei, Hou, Lijuan, Li, Aihui, Duan, Yuxiu, Gao, Haili. Expression of serum exosomal microRNA-21 in human hepatocellular carcinoma. BioMed research international 2014;2014():864894. | Exclude on population: not cirrhosis |
| Wang, J. H., Chang, K. C., Kee, K. M., Chen, P. F., Yen, Y. H., Tseng, P. L., et al. Hepatocellular carcinoma surveillance at 4-vs. 12-month intervals for patients with chronic viral hepatitis: A randomized study in community. American Journal of Gastroenterology 2013;108(3):416-24. | Exclude on population: not cirrhosis |

| Reference | Reason for exclusion |
|---|---|
| Wang, J., Tang, X., Weng, W., Qiao, Y., Lin, J., Liu, W., et al. The membrane protein melanoma cell adhesion molecule (MCAM) is a novel tumor marker that stimulates tumorigenesis in hepatocellular carcinoma. Oncogene 2015;34(47):5781-95. | No 2x2 data |
| Wang, Jeremy, Jain, Surbhi, Chen, Dion, Song, Wei, Hu, Chi-Tan. Development and Evaluation of Novel Statistical Methods in Urine Biomarker-Based Hepatocellular Carcinoma Screening. Scientific reports 2018;8(1):3799. | Exclude on population: not cirrhosis |
| Wang, Jia-Hui, Qiu, Qian-Sai, Dong, San-Yuan, Chen, Xiao-Shan, Wang, Wen-Tao, Yang, Yu-Tao, Sun, Wei. Diagnostic performance of gadoxetic acid-enhanced abbreviated magnetic resonance imaging protocol in small hepatocellular carcinoma (<=2 cm) in high-risk patients. Acta radiologica (Stockholm, Sweden : 1987) 2023;64(10):2687-96. | Does not meet full eligibility criteria: includes people without cirrhosis |
| Wang, Jing-Wen, Qian, Yu, Wu, Chen-Si, Zhao, Ning-Hui, Fang, Yu, Yuan, Xiao-Dong, et al. Combined use of murine double minute-2 promoter methylation and serum AFP improves diagnostic efficiency in hepatitis B virus-related hepatocellular carcinoma. International journal of medical sciences 2020;17(18):3190-99. | Does not meet full eligibility criteria: includes people without cirrhosis |
| Wang, Jinhong, Qin, Yang, Li, Bo, Sun, Zhilin. Detection of aberrant promoter methylation of GSTP1 in the tumor and serum of Chinese human primary hepatocellular carcinoma patients. Clinical biochemistry 2006;39(4):344-48. | Does not meet full eligibility criteria: includes people without cirrhosis |
| Wang, Juan, Yang, Liu, Diao, Yanjun, Liu, Jiayun, Li, Jinjie, Li, Rui, et al. Circulating tumour DNA methylation in hepatocellular carcinoma diagnosis using digital droplet PCR. The Journal of international medical research 2021;49(3):300060521992962. | Exclude on population: not cirrhosis |
| Wang, Ju-Jun. Rapid kinetic rate assay of the serum alpha-L-fucosidase in patients with hepatocellular carcinoma by using a novel substrate. Clinica chimica acta; international journal of clinical chemistry 2004;347(1-2):103-09. | Exclude on population: not cirrhosis |
| Wang, K., Wang, X., Jiao, J., Li, Y., Gu, W. Automatic time-resolved fluorescence immunoassay of serum alpha fetoprotein-l3 variant via lca magnetic cationic polymeric liposomes improves the diagnostic accuracy of liver cancer. International Journal of Nanomedicine 2020;15():4933-41. | Does not meet full eligibility criteria: includes people without cirrhosis |
| Wang, Kaijuan, Xu, Xueqin, Nie, Yifei, Dai, Liping, Wang, Peng. Identification of tumor-associated antigens by using SEREX in hepatocellular carcinoma. Cancer letters 2009;281(2):144-50. | Exclude on population: not cirrhosis |
| Wang, Kang, Guo, Wei Xing, Li, Nan, Gao, Chun Fang, Shi, Jie, Tang, Yu Fu, et al. Serum LncRNAs Profiles Serve as Novel Potential Biomarkers for the Diagnosis of HBV-Positive Hepatocellular Carcinoma. PloS one 2015;10(12):e0144934. | Does not meet full eligibility criteria: includes people without cirrhosis |
| Wang, Ke, Bai, Yansen, Chen, Shi, Huang, Jiao, Yuan, Jing, Chen, Weihong, et al. Genetic correction of serum AFP level improves risk prediction of primary hepatocellular carcinoma in the Dongfeng-Tongji cohort study. Cancer medicine 2018;7(6):2691-98. | Exclude on population: not cirrhosis |
| Wang, Keyan, Li, Miao, Qin, Jiejie, Sun, Guiying, Dai, Liping, Wang, Peng, et al. Serological Biomarkers for Early Detection of Hepatocellular Carcinoma: A Focus on Autoantibodies against Tumor-Associated Antigens Encoded by Cancer Driver Genes. Cancers 2020;12(5):n. pag.. | Does not meet full eligibility criteria: includes people without cirrhosis |
| Wang, L., Pan, L., Yao, M., Zheng, W., Fang, M., Qiu, L., Dong, Z. Clinical values of Wnt3a as a novel biomarker in diagnosis and prognosis of hepatocellular carcinoma. National Medical Journal of China 2016;96(44):3554-58. | Foreign language |
| Wang, M., Li, H., Chen, K., Zeng, H., Bi, X., Zhu, Z., et al. A male-abcd algorithm for hepatocellular carcinoma risk prediction in hbsag carriers. Chinese Journal of Cancer Research 2021;33(3):352-63. | Exclude on study design: not a 1- or 2-gate test accuracy study |
| Wang, Meiyun, Fu, Fangfang, Zheng, Bingjie, Bai, Yan, Wu, Qingxia, Wu, Jianqiang, et al. Development of an AI system for accurately diagnose hepatocellular carcinoma from computed tomography imaging data. British journal of cancer 2021;125(8):1111-21. | Exclude on population: not cirrhosis |
| Wang, Mengjun, Block, Timothy M., Marrero, Jorge, Di Bisceglie, Adrian M., Devarajan, Karthik. Improved biomarker performance for the detection of hepatocellular carcinoma by inclusion of clinical parameters. Proceedings. | Exclude on index test |

| Reference | Reason for exclusion |
|---|---|
| IEEE International Conference on Bioinformatics and Biomedicine 2012;2012():n. pag.. | |
| Wang, Mengjun, Devarajan, Karthik, Singal, Amit G, Marrero, Jorge A, Dai, Jianliang, Feng, Ziding, et al. The Doylestown Algorithm: A Test to Improve the Performance of AFP in the Detection of Hepatocellular Carcinoma. Cancer prevention research (Philadelphia, Pa.) 2016;9(2):172-9. | Secondary publication of included study |
| Wang, Mengjun, Devarajan, Karthik, Singal, Amit G., Marrero, Jorge A., Dai, Jianliang, Feng, Ziding, et al. The Doylestown Algorithm: A Test to Improve the Performance of AFP in the Detection of Hepatocellular Carcinoma. Cancer prevention research (Philadelphia, Pa.) 2016;9(2):172-79. | Duplicate (including Cochrane includes) |
| Wang, Mengjun, Long, Ronald E, Comunale, Mary Ann, Junaidi, Omer, Marrero, Jorge, Di Bisceglie, Adrian M, Block, Timothy M. Novel fucosylated biomarkers for the early detection of hepatocellular carcinoma. Cancer epidemiology, biomarkers & prevention : a publication of the American Association for Cancer Research, cosponsored by the American Society of Preventive Oncology 2009;18(6):1914-21. | Continuous test without threshold |
| Wang, Mengjun, Long, Ronald E., Comunale, Mary Ann, Junaidi, Omer, Marrero, Jorge, Di Bisceglie, Adrian M., Block, Timothy M. Novel fucosylated biomarkers for the early detection of hepatocellular carcinoma. Cancer epidemiology, biomarkers & prevention : a publication of the American Association for Cancer Research, cosponsored by the American Society of Preventive Oncology 2009;18(6):1914-21. | Duplicate (including Cochrane includes) |
| Wang, Mengjun, Mehta, Anand, Block, Timothy M., Marrero, Jorge, Di Bisceglie, Adrian M. A comparison of statistical methods for the detection of hepatocellular carcinoma based on serum biomarkers and clinical variables. BMC medical genomics 2013;6 Suppl 3():S9. | Unvalidated model / threshold not pre-specified |
| Wang, N. Y., Zhao, W., Zhang, D., Zhang, Y. C. Clinical application of avidin-biotin ELISA to detect serum hepatoma-specific gamma-glutamyltransferase in patients with primary hepatic cancer. Zhonghua zhong liu za zhi [Chinese journal of oncology] 2009;31(2):114-17. | Foreign language |
| Wang, Na, Cao, Yuan, Song, Wei, He, Kun, Li, Tao, Wang, Jie, et al. Serum peptide pattern that differentially diagnoses hepatitis B virus-related hepatocellular carcinoma from liver cirrhosis. Journal of gastroenterology and hepatology 2014;29(7):1544-50. | Does not meet full eligibility criteria: includes people without cirrhosis |
| Wang, Na, Cao, Yuan, Song, Wei, He, Kun, Li, Tao, Wang, Jie, et al. Serum peptide pattern that differentially diagnoses hepatitis B virus-related hepatocellular carcinoma from liver cirrhosis. Journal of gastroenterology and hepatology 2014;29(7):1544-50. | Duplicate (including Cochrane includes) |
| Wang, Nian-Yue, Zhang, Dai, Zhao, Wei, Fang, Guo-Xiang, Shi, Yu-Ling. Clinical application of an enzyme-linked immunosorbent assay detecting hepatoma-specific gamma-glutamyltransferase. Hepatology research : the official journal of the Japan Society of Hepatology 2009;39(10):979-87. | Does not meet full eligibility criteria: includes people without cirrhosis |
| Wang, Pei, Song, Qianqian, Ren, Jie, Zhang, Weilong, Wang, Yuting, Zhou, Lin, et al. Simultaneous analysis of mutations and methylations in circulating cell-free DNA for hepatocellular carcinoma detection. Science translational medicine 2022;14(672):eabp8704. | Exclude on population: not cirrhosis |
| Wang, Q., Cui, H., Zhu, Y. Serum Golgi protein 73 (GP73) is a diagnostic and prognostic marker of hepatocellular carcinoma. Frontiers in Medicine 2025;12():1571761. | Exclude on population: not cirrhosis |
| Wang, Q., Xie, Y., Zhang, L., Wu, K., Zhu, Y., Wu, F. Diagnostic value of abnormal prothrombin level combined with afp and afp-l3% for chronic hepatitis b-related liver cancer. International Journal of Clinical and Experimental Medicine 2020;13(11):8875-81. | Exclude on target condition: not HCC |
| Wang, Qiang, Chen, Qi, Zhang, Xia, Lu, Xiao-Lan, Du, Qin, Zhu, Tao, et al. Diagnostic value of gamma-glutamyltransferase/aspartate aminotransferase ratio, protein induced by vitamin K absence or antagonist II, and alpha-fetoprotein in hepatitis B virus-related hepatocellular carcinoma. World journal of gastroenterology 2019;25(36):5515-29. | Does not meet full eligibility criteria: includes people without cirrhosis |
| Wang, Qiang, Chen, Qi, Zhang, Xia, Lu, Xiao-Lan, Du, Qin, Zhu, Tao, et al. Diagnostic value of gamma-glutamyltransferase/aspartate aminotransferase | Duplicate (including Cochrane includes) |

| Reference | Reason for exclusion |
|---|---|
| ratio, protein induced by vitamin K absence or antagonist II, and alpha-fetoprotein in hepatitis B virus-related hepatocellular carcinoma. World journal of gastroenterology 2019;25(36):5515-29. | |
| Wang, Rongming, Zang, Weiwei, Hu, Bobin, Deng, Deli, Ling, Xiaozhang, Zhou, Huikun, Su, Minghua. Serum ESPL1 Can Be Used as a Biomarker for Patients With Hepatitis B Virus-Related Liver Cancer: A Chinese Case-Control Study. Technology in cancer research & treatment 2020;19():1533033820980785. | Exclude on study design: not a 1- or 2-gate test accuracy study |
| Wang, S. H., Du, J., Xu, H., Yang, D., Ye, Y., Chen, Y., et al. Automatic discrimination of different sequences and phases of liver MRI using a dense feature fusion neural network: a preliminary study. Abdominal Radiology 2021;46(10):4576-87. | Exclude on population: not cirrhosis |
| Wang, Shuwei, Chen, Shixiang, Jin, Ming, Hu, Mengyuan, Huang, Weiguang, Jiang, Zhenluo, et al. Diagnostic and prognostic value of serum Chitinase 3-like protein 1 in hepatocellular carcinoma. Journal of clinical laboratory analysis 2022;36(2):e24234. | Exclude on population: not cirrhosis |
| Wang, T., Liu, M., Zheng, S. J., Bian, D. D., Zhang, J. Y., Yao, J., et al. Tumor-associated autoantibodies are useful biomarkers in immunodiagnosis of a-fetoprotein-negative hepatocellular carcinoma. World Journal of Gastroenterology 2017;23(19):3496-5004. | Exclude on population: not cirrhosis |
| Wang, T., Wang, F. M., Gao, Y. T., Zhu, Z. Y., Guo, H. S., Yang, B., et al. Significance of GPC3 expression in liver biopsy specimens for differential diagnosis of liver diseases. World Chinese Journal of Digestology 2011;19(7):693-99. | Foreign language |
| Wang, Ting, Liu, Mei, Zheng, Su-Jun, Bian, Dan-Dan, Zhang, Jin-Yan, Yao, Jia, et al. Tumor-associated autoantibodies are useful biomarkers in immunodiagnosis of alpha-fetoprotein-negative hepatocellular carcinoma. World journal of gastroenterology 2017;23(19):3496-5004. | Exclude on index test |
| Wang, Ting, Zhang, Kun-He, Hu, Piao-Ping, Huang, Zeng-Yong, Zhang, Pan, Wan, Qin-Si, Huang, De-Qiang. Simple and robust diagnosis of early, small and AFP-negative primary hepatic carcinomas: an integrative approach of serum fluorescence and conventional blood tests. Oncotarget 2016;7(39):64053-70. | Does not meet full eligibility criteria: includes people without cirrhosis |
| Wang, Ting, Zhang, Kun-He, Hu, Piao-Ping, Wan, Qin-Si, Han, Fang-Li, Zhou, Jian-Ming, Huang, De-Qiang. Combination of dual serum fluorescence, AFP and hepatic function tests is valuable to identify HCC in AFP-elevated liver diseases. Oncotarget 2017;8(58):97758-68. | Exclude on population: not cirrhosis |
| Wang, W. & Wang, J. H. Detection of serum markers of primary hepatocellular carcinoma by ELISA using synthetic polypeptide antigen. Chinese Journal of Biologicals 2009;22(4):399-401. | Foreign language |
| Wang, W., Wang, J. H., Miao, X. H. Detection of serum markers of hepatocellular carcinoma by ELISA. Chinese Journal of Biologicals 2010;23(3):307-09. | Foreign language |
| Wang, W., Zhao, L. J., Wang, Y., Tao, Q. Y., Feitelson, M. A., Zhao, P., Ren, H. Application of HBx-induced anti-URGs as early warning biomarker of cirrhosis and HCC. Cancer Biomarkers 2012;11(1):29-39. | Exclude on population: not cirrhosis |
| Wang, Wen-Chao, Zhang, Xiao-Feng, Tang, Er-Jiang, Li, A. Jian, Chen, Lei, Wang, Jia-Qi, et al. Thymosin beta4, a potential marker of malignancy and prognosis in hepatocellular carcinoma. Scandinavian journal of gastroenterology 2023;58(4):380-91. | Does not meet full eligibility criteria: includes people without cirrhosis |
| Wang, Wenjie, Li, Huiyu, Zhou, Yan. Peripheral blood microvesicles are potential biomarkers for hepatocellular carcinoma. Cancer biomarkers : section A of Disease markers 2013;13(5):351-57. | Duplicate (including Cochrane includes) |
| Wang, Wenjie, Li, Huiyu, Zhou, Yan. Peripheral blood microvesicles are potential biomarkers for hepatocellular carcinoma. Cancer biomarkers : section A of Disease markers 2013;13(5):351-7. | Does not meet full eligibility criteria: includes people without cirrhosis |
| Wang, Who-Whong, Ang, Soo Fan, Kumar, Rajneesh, Heah, Charmain, Utama, Andi, Tania, Navessa Padma, et al. Identification of serum monocyte chemoattractant protein-1 and prolactin as potential tumor markers in hepatocellular carcinoma. PloS one 2013;8(7):e68904. | Exclude on population: not cirrhosis |

| Reference | Reason for exclusion |
|---|---|
| Wang, Who-Whong, Ang, Soo Fan, Kumar, Rajneesh, Heah, Charmain, Utama, Andi, Tania, Navessa Padma, et al. Identification of serum monocyte chemoattractant protein-1 and prolactin as potential tumor markers in hepatocellular carcinoma. PloS one 2013;8(7):e68904. | Duplicate (including Cochrane includes) |
| Wang, Xiao, Wang, Keyan, Qiu, Cuipeng, Wang, Bofei, Zhang, Xiaojun, Ma, Yangcheng, Dai, Liping. Autoantibody to GNAS in Early Detection of Hepatocellular Carcinoma: A Large-Scale Sample Study Combined with Verification in Serial Sera from HCC Patients. Biomedicines 2022;10(1):n. pag.. | Does not meet full eligibility criteria: includes people without cirrhosis |
| Wang, Xin, Li, Ming-Ming, Niu, Ye, Zhang, Xin, Yin, Ji-Bin, Zhao, Chang-Jiu. Serum Zonulin in HBV-Associated Chronic Hepatitis, Liver Cirrhosis, and Hepatocellular Carcinoma. Disease markers 2019;2019():5945721. | Duplicate (including Cochrane includes) |
| Wang, Xin, Li, Ming-Ming, Niu, Ye, Zhang, Xin, Yin, Ji-Bin, Zhao, Chang-Jiu. Serum Zonulin in HBV-Associated Chronic Hepatitis, Liver Cirrhosis, and Hepatocellular Carcinoma. Disease markers 2019;2019(dim, 8604127):5945721. | Does not meet full eligibility criteria: includes people without cirrhosis |
| Wang, Xinmei, Kwak, Kwang Joo, Yang, Zhaogang, Zhang, Aili, Zhang, Xiaoli, Sullivan, Rachael, et al. Extracellular mRNA detected by molecular beacons in tethered lipoplex nanoparticles for diagnosis of human hepatocellular carcinoma. PloS one 2018;13(6):e0198552. | Exclude on population: healthy controls |
| Wang, Xiumei, Zhang, Weiwei, Liu, Youde, Gong, Wenjing, Sun, Ping, Kong, Xiangshuo, Yang, Miaomiao. Diagnostic value of prothrombin induced by the absence of vitamin K or antagonist-II (PIVKA-II) for early stage HBV related hepatocellular carcinoma. Infectious agents and cancer 2017;12(101276559):47. | Does not meet full eligibility criteria: includes people without cirrhosis |
| Wang, Xiumei, Zhang, Weiwei, Liu, Youde, Gong, Wenjing, Sun, Ping, Kong, Xiangshuo, Yang, Miaomiao. Diagnostic value of prothrombin induced by the absence of vitamin K or antagonist-II (PIVKA-II) for early stage HBV related hepatocellular carcinoma. Infectious agents and cancer 2017;12():47. | Duplicate (including Cochrane includes) |
| Wang, Y. Y., Yang, W. X., Du, Q. J., Liu, Z. H., Lu, M. H. Construction and evaluation of a liver cancer risk prediction model based on machine learning. World Journal of Gastrointestinal Oncology 2024;16(9):3839 “ 3850. | Does not meet full eligibility criteria: includes people without cirrhosis |
| Wang, Y., Jian, W., Yuan, Z., Guan, F. nner; Deep learning with attention modules and residual transformations improves hepatocellular carcinoma (HCC) differentiation using multiphase CT. Precision Radiation Oncology 2025;9(1):13 “ 22. | Exclude on population: not cirrhosis |
| Wang, Y., Liang, Z., Gao, Y., Zhai, D., Rao, Q., Shi, W., et al. Factors influencing circulating MicroRNA level in the studies of hepatocellular carcinoma biomarker. Neoplasma 2015;62(5):798-804. | Exclude on population: healthy controls |
| Wang, Yangyang, Chi, Shengqiang, Tian, Yu, Li, Xueyao, Zhang, Hang, Xu, Yiting, et al. Construction of an artificially intelligent model for accurate detection of HCC by integrating clinical, radiological, and peripheral immunological features. International journal of surgery (London, England) 2025;111(4):2942â€“2952. | Exclude on population: not cirrhosis |
| Wang, Yanping, Wang, Huan, Wei, Shuai, Gao, Zhiliang, Gao, Haili, Wang, Xinwei, Liang, Haijun. Metabolomic comparative study in patients with liver cirrhosis and hepatocellular carcinoma related to hepatitis B virus infection. European journal of gastroenterology & hepatology 2025;37(9):1040â€“1048. | Exclude on index test |
| Wang, Yibo, Luo, Fang, Zheng, Yi, Fan, Xiaohan, Chen, Jinxing, Zhang, Yu. VKORC1 haplotypes influence the performance characteristics of PIVKAII for screening of hepatocellular carcinoma. Clinical chemistry and laboratory medicine 2010;48(10):1475-79. | Exclude on population: not cirrhosis |
| Wang, Yichen, Yang, Huayu, Xu, Haifeng, Lu, Xin, Sang, Xinting, Zhong, Shouxian, Huang, Jiefu. Golgi protein 73, not Glypican-3, may be a tumor marker complementary to alpha-Fetoprotein for hepatocellular carcinoma diagnosis. Journal of gastroenterology and hepatology 2014;29(3):597-602. | Does not meet full eligibility criteria: includes people without cirrhosis |
| Wang, Yichen, Yang, Huayu, Xu, Haifeng, Lu, Xin, Sang, Xinting, Zhong, Shouxian, Huang, Jiefu. Golgi protein 73, not Glypican-3, may be a tumor marker complementary to alpha-Fetoprotein for hepatocellular carcinoma diagnosis. Journal of gastroenterology and hepatology 2014;29(3):597-602. | Duplicate (including Cochrane includes) |

| Reference | Reason for exclusion |
|---|---|
| Wang, Yu, Yu, Weixin, He, Mingqing, Huang, Yan, Wang, Mingyue. Serum cytoskeleton-associated protein 4 as a biomarker for the diagnosis of hepatocellular carcinoma. OncoTargets and therapy 2019;12():359-64. | Exclude on population: healthy controls |
| Wang, Yuanxiao, Pei, Lin, Yue, Zhihong, Jia, Mei, Wang, Hui. The Potential of Serum Exosomal hsa_circ_0028861 as the Novel Diagnostic Biomarker of HBV-Derived Hepatocellular Cancer. Frontiers in genetics 2021;12():703205. | Does not meet full eligibility criteria: includes people without cirrhosis |
| Wang, Yuan-Yuan, Zhou, Chen-Jie, Li, Jing, Zhou, Ling, Li, Ming-Song. [Value of detection of serum glypican-3 level in diagnosis and therapeutic effect evaluation of primary hepatocellular carcinoma]. Nan fang yi ke da xue xue bao = Journal of Southern Medical University 2017;37(8):1060-65. | Foreign language |
| Wang, Yurong, Zhang, Chunyan, Zhang, Pengjun, Guo, Guanghong, Jiang, Tao, Zhao, Xiumei, et al. Serum exosomal microRNAs combined with alpha-fetoprotein as diagnostic markers of hepatocellular carcinoma. Cancer medicine 2018;7(5):1670-79. | Does not meet full eligibility criteria: includes people without cirrhosis |
| Wang, Z., Kong, L., Zhang, R., Yang, X., Cao, Z., Xu, T., Zhang, H. Serum Aldo-Keto Reductase Family 1 Member B10 (AKR1B10) as a Potential Biomarker for Diagnosis of Hepatocellular Carcinoma. Journal of Hepatocellular Carcinoma 2024;11():131 “ 143. | Exclude on population: not cirrhosis |
| Wang, Zhihuai, Gao, Peng, Sun, Weijun, Rehman, Adeel Ur, Jiang, Jiakai, Xu, Suobao, et al. Long noncoding RNA MyD88 functions as a promising diagnostic biomarker in hepatocellular carcinoma. Frontiers in endocrinology 2023;14():938102. | Does not meet full eligibility criteria: includes people without cirrhosis |
| Ward, D. G., Cheng, Y., N'Kontchou, G., Thar, T. T., Barget, N., Wei, W., et al. Changes in the serum proteome associated with the development of hepatocellular carcinoma in hepatitis C-related cirrhosis. British journal of cancer 2006;94(2):287-92. | Exclude on index test |
| Watanabe, H., Kanematsu, M., Goshima, S., Kondo, H., Kajita, K., Kawada, H., Noda, Y. Detection of focal hepatic lesions with 3-T MRI: Comparison of two-dimensional and three-dimensional T2-weighted sequences. Japanese Journal of Radiology 2012;30(9):721-28. | Exclude on population: clinical signs and symptoms of HCC |
| Wei, H., Luo, S., Bi, Y., Liao, C., Lian, Y., Zhang, J. Plasma microRNA-15a/16-1-based machine learning for early detection of hepatitis B virus-related hepatocellular carcinoma. Liver Research 2024;8(2):105 “ 117. | Exclude on population: not cirrhosis |
| Wei, M. Analysis of expressions and clinical significance of CXCR1, CXCR2 and CXCL8 in patients with primary hepatic carcinoma. Acta Medica Mediterranea 2018;34(6):1721-26. | Exclude on population: not cirrhosis |
| Wei, Rongrong, Huang, Guo-Liang, Zhang, Mei-Yin, Li, Bin-Kui, Zhang, Hui-Zhong, Shi, Ming, et al. Clinical significance and prognostic value of microRNA expression signatures in hepatocellular carcinoma. Clinical cancer research : an official journal of the American Association for Cancer Research 2013;19(17):4780-91. | Exclude on population: not cirrhosis |
| Wei, Siwei, Suryani, Yuliana, Gowda, G. A. Nagana, Skill, Nicholas, Maluccio, Mary. Differentiating hepatocellular carcinoma from hepatitis C using metabolite profiling. Metabolites 2012;2(4):701-16. | Exclude on population: not cirrhosis |
| Weis, Anna, Marquart, Louise, Calvopina, Diego A., Genz, Berit, Ramm, Grant A. Serum MicroRNAs as Biomarkers in Hepatitis C: Preliminary Evidence of a MicroRNA Panel for the Diagnosis of Hepatocellular Carcinoma. International journal of molecular sciences 2019;20(4):n. pag.. | Duplicate (including Cochrane includes) |
| Welberry, C., Macdonald, I., McElveen, J., Parsy-Kowalska, C., Allen, J., Healey, G., et al. Tumor-associated autoantibodies in combination with alpha-fetoprotein for detection of early stage hepatocellular carcinoma. PLoS ONE 2020;15(5):e0232247. | Exclude on population: not cirrhosis |
| Welling, Theodore H., Fu, Sherry, Wan, Shanshan, Zou, Weiping. Elevated serum IL-8 is associated with the presence of hepatocellular carcinoma and independently predicts survival. Cancer investigation 2012;30(10):689-97. | No 2x2 data |
| Wen, B., Mi, J. J., Hou, T. H., Guo, X. H., Bai, C. A. Expression of serum bone morphogenetic protein 2 in patients with primary hepatic carcinoma. World Chinese Journal of Digestology 2010;18(4):397-99. | Foreign language |

| Reference | Reason for exclusion |
|---|---|
| Wen, Chi-Pang, Lin, Jie, Yang, Yi Chen, Tsai, Min Kuang, Tsao, Chwen Keng, Etzel, Carol, et al. Hepatocellular carcinoma risk prediction model for the general population: the predictive power of transaminases. Journal of the National Cancer Institute 2012;104(20):1599-6111. | Exclude on study design: not a 1- or 2-gate test accuracy study |
| Wen, Lu, Li, Jingyi, Guo, Huahu, Liu, Xiaomeng, Zheng, Shengmin, Zhang, Dafang, et al. Genome-scale detection of hypermethylated CpG islands in circulating cell-free DNA of hepatocellular carcinoma patients. Cell research 2015;25(11):1250-64. | Exclude on population: healthy controls |
| Wen, Yang, Han, Jing, Chen, Jianguo, Dong, Jing, Xia, Yongxiang, Liu, Jibin, et al. Plasma miRNAs as early biomarkers for detecting hepatocellular carcinoma. International journal of cancer 2015;137(7):1679-90. | Exclude on population: not cirrhosis |
| Whang, Sunyoung, Choi, Moon Hyung, Choi, Joon-Il, Youn, Seo Yeon, Kim, Dong Hwan. Comparison of diagnostic performance of non-contrast MRI and abbreviated MRI using gadoxetic acid in initially diagnosed hepatocellular carcinoma patients: a simulation study of surveillance for hepatocellular carcinomas. European radiology 2020;30(8):4150-63. | Does not meet full eligibility criteria: includes people without cirrhosis |
| White, D. L., Richardson, P., Tayoub, N., Davila, J. A., Kanwal, F. The Updated Model: An Adjusted Serum Alpha-Fetoprotein-Based Algorithm for Hepatocellular Carcinoma Detection with Hepatitis C Virus-Related Cirrhosis. Gastroenterology 2015;149(7):1986-87. | Exclude on study design: not a 1- or 2-gate test accuracy study |
| Wiwanitkit, Viroj. Alpha fetoprotein for screening for hepatocellular cancer in populations with viral hepatitis B: an appraisal of Thai reports. Asian Pacific journal of cancer prevention : APJCP 2005;6(4):535-36. | Exclude on study design: not a 1- or 2-gate test accuracy study |
| Wong, G. L. H., Hui, V. W. K., Tan, Q., Xu, J., Lee, H. W., Yip, T. C. F., et al. Novel machine learning models outperform risk scores in predicting hepatocellular carcinoma in patients with chronic viral hepatitis. JHEP Reports 2022;4(3):100441. | Exclude on population: not cirrhosis |
| Wong, Grace L H, Chan, Henry L Y, Tse, Yee-Kit, Chan, Hoi-Yun, Tse, Chi-Hang, Lo, Angeline O S. On-treatment alpha-fetoprotein is a specific tumor marker for hepatocellular carcinoma in patients with chronic hepatitis B receiving entecavir. Hepatology (Baltimore, Md.) 2014;59(3):986-95. | Exclude on population: not cirrhosis |
| Wong, Grace L. H., Chan, Henry L. Y., Tse, Yee-Kit, Chan, Hoi-Yun, Tse, Chi-Hang, Lo, Angeline O. S. On-treatment alpha-fetoprotein is a specific tumor marker for hepatocellular carcinoma in patients with chronic hepatitis B receiving entecavir. Hepatology (Baltimore, Md.) 2014;59(3):986-95. | Duplicate (including Cochrane includes) |
| Wong, Grace Lai-Hung, Chan, Henry Lik-Yuen, Chan, Hoi-Yun, Tse, Pete Chi-Hang, Tse, Yee-Kit, Mak, Christy Wing-Hin, et al. Accuracy of risk scores for patients with chronic hepatitis B receiving entecavir treatment. Gastroenterology 2013;144(5):933-44. | Exclude on population: not cirrhosis |
| Wong, Grace Lai-Hung, Wong, Vincent Wai-Sun, Tan, Guang-Ming, Ip, Ka-Ian, Lai, Wing-Kwan, Li, Yuk-Wah, et al. Surveillance programme for hepatocellular carcinoma improves the survival of patients with chronic viral hepatitis. Liver international : official journal of the International Association for the Study of the Liver 2008;28(1):79-87. | Exclude on population: clinical signs and symptoms of HCC |
| Wong, I. H. N., Lo, Y. M. D., Yeo, W., Lau, W. Y. Frequent p15 promoter methylation in tumor and peripheral blood from hepatocellular carcinoma patients. Clinical Cancer Research 2000;6(9):3516-21. | Exclude on population: not cirrhosis |
| Wong, K. F. Discovery of lamin B1 and vimentin as circulating biomarkers for early hepatocellular carcinoma. Liver Proteomics: Methods and Protocols 2012;909():295-310. | Exclude on index test |
| Wong, Katherine, Ozeki, Katharine, Kwong, Allison, Patel, Bhavik N. The effects of a transjugular intrahepatic portosystemic shunt on the diagnosis of hepatocellular cancer. PloS one 2018;13(12):e0208233. | Exclude on study design: not a 1- or 2-gate test accuracy study |
| Wong, Kwong-Fai. Discovery of lamin B1 and vimentin as circulating biomarkers for early hepatocellular carcinoma. Methods in molecular biology (Clifton, N.J.) 2012;909():295-310. | Exclude on index test |
| Wong, Vincent Wai-Sun, Yu, Jun, Cheng, Alfred Sze-Lok, Wong, Grace Lai-Hung, Chan, Hoi-Yun, Chu, Eagle Siu-Hong, et al. High serum interleukin-6 level predicts future hepatocellular carcinoma development in patients with chronic hepatitis B. International journal of cancer 2009;124(12):2766-70. | Exclude on population: not cirrhosis |

| Reference | Reason for exclusion |
| --- | --- |
| Wu, Chen, Deng, Lei, Zhuo, Han, Chen, Xiang, Tan, Zhongming, Han, Sheng, et al. Circulating circRNA predicting the occurrence of hepatocellular carcinoma in patients with HBV infection. Journal of cellular and molecular medicine 2020;24(17):10216-22. | Exclude on population: not cirrhosis |
| Wu, Cheng, Wang, Zhanfeng, Liu, Lijie, Zhao, Peng, Wang, Wenjing, Yao, Dingkang, et al. Surface enhanced laser desorption/ionization profiling: New diagnostic method of HBV-related hepatocellular carcinoma. Journal of gastroenterology and hepatology 2009;24(1):55-62. | Does not meet full eligibility criteria: includes people without cirrhosis |
| Wu, Cheng, Wang, Zhanfeng, Liu, Lijie, Zhao, Peng, Wang, Wenjing, Yao, Dingkang, et al. Surface enhanced laser desorption/ionization profiling: New diagnostic method of HBV-related hepatocellular carcinoma. Journal of gastroenterology and hepatology 2009;24(1):55-62. | Duplicate (including Cochrane includes) |
| Wu, Chen-Shiou, Lee, Teng-Yu, Chou, Ruey-Hwang, Yen, Chia-Jui, Huang, Wei-Chien, Wu, Chung-Yi. Development of a highly sensitive glycan microarray for quantifying AFP-L3 for early prediction of hepatitis B virus-related hepatocellular carcinoma. PloS one 2014;9(6):e99959. | Exclude on population: not cirrhosis |
| Wu, Chen-Shiou, Yen, Chia-Jui, Chou, Ruey-Hwang, Li, Shiou-Ting, Huang, Wei-Chien, Ren, Chien-Tai, Wu, Chung-Yi. Cancer-associated carbohydrate antigens as potential biomarkers for hepatocellular carcinoma. PloS one 2012;7(7):e39466. | Exclude on population: not cirrhosis |
| Wu, F. & Wang, C. X. Expression and clinical significance of LncRNA ZFAS1 in serum exosomes of patients with hepatocellular carcinoma. Chinese Journal of Cancer Prevention and Treatment 2019;26(12):849-54. | Foreign language |
| Wu, Fei-Xiang, Wang, Qi, Zhang, Zhi-Ming, Huang, Shang, Yuan, Wei-Ping, Liu, Jian-Yong, Ban, Ke-Chen. Identifying serological biomarkers of hepatocellular carcinoma using surface-enhanced laser desorption/ionization-time-of-flight mass spectroscopy. Cancer letters 2009;279(2):163-70. | Exclude on population: healthy controls |
| Wu, Hu-bing, Wang, Quan-shi, Li, Bao-yuan, Li, Hong-sheng, Zhou, Wen-lan. F-18 FDG in conjunction with 11C-choline PET/CT in the diagnosis of hepatocellular carcinoma. Clinical nuclear medicine 2011;36(12):1092-97. | Exclude on study design: not a 1- or 2-gate test accuracy study |
| Wu, Hui-Chen, Yang, Hwai- I., Wang, Qiao, Chen, Chien-Jen. Plasma DNA methylation marker and hepatocellular carcinoma risk prediction model for the general population. Carcinogenesis 2017;38(10):1021-28. | Exclude on population: not cirrhosis |
| Wu, J. W., Meng, X. Y., Xu, K. C., Shi, Y. C. Simultaneous determination of multiple markers of primary liver cancer: Diagnostic significance. Journal of Gastroenterology and Hepatology 1988;3(1):29-35. | Does not meet full eligibility criteria: pre-2005 |
| Wu, Jiali, Xiang, Zheyi, Bai, Le, He, Lagu, Tan, Li, Hu, Min. Diagnostic value of serum PIVKA-II levels for BCLC early hepatocellular carcinoma and correlation with HBV DNA. Cancer biomarkers : section A of Disease markers 2018;23(2):235-42. | Does not meet full eligibility criteria: includes people without cirrhosis |
| Wu, Jiali, Xiang, Zheyi, Bai, Le, He, Lagu, Tan, Li, Hu, Min. Diagnostic value of serum PIVKA-II levels for BCLC early hepatocellular carcinoma and correlation with HBV DNA. Cancer biomarkers : section A of Disease markers 2018;23(2):235-42. | Duplicate (including Cochrane includes) |
| Wu, Jin-Lin, Su, Tung-Hung, Chen, Pei-Jer. Acute-phase serum amyloid A for early detection of hepatocellular carcinoma in cirrhotic patients with low AFP level. Scientific reports 2022;12(1):5799. | Does not meet full eligibility criteria: includes people without cirrhosis |
| Wu, Jinyu, Wang, Peng, Han, Zhuo, Li, Tiandong, Yi, Chuncheng, Qiu, Cuipeng, et al. A novel immunodiagnosis panel for hepatocellular carcinoma based on bioinformatics and the autoantibody-antigen system. Cancer science 2022;113(2):411-22. | Exclude on index test |
| Wu, L. Q., Wang, X. J., Zhang, B., Lu, Y. Expression of cancer-testis antigen SSX-2 and SSX-5 in tissues and peripheral blood of patients with hepatocellular carcinoma. World Chinese Journal of Digestology 2005;13(14):1667-72. | Foreign language |
| Wu, L., Liu, Z., Huang, H., Pan, D., Fu, C., Lu, Y., et al. Development and validation of an interpretable machine learning model for predicting the risk of hepatocellular carcinoma in patients with chronic hepatitis B: a case-control study. BMC Gastroenterology 2025;25(1):157. | Exclude on population: not cirrhosis |

| Reference | Reason for exclusion |
|---|---|
| Wu, Li-Qun, Lu, Yun, Wang, Xue-Feng, Lv, Zhen-Hua, Zhang, Bin. Expression of cancer-testis antigen (CTA) in tumor tissues and peripheral blood of Chinese patients with hepatocellular carcinoma. Life sciences 2006;79(8):744-48. | Does not meet full eligibility criteria: includes people without cirrhosis |
| Wu, M., Zou, F., He, S., Pi, Y., Song, Y., Chen, S. Serum Osteopontin Enhances Hepatocellular Carcinoma Diagnosis and Predicts Anti-PD-L1 Immunotherapy Benefit. Journal of Hepatocellular Carcinoma 2025;12():729 " 745. | Exclude on population: not cirrhosis |
| Wu, Min, Liu, Zhaobo, Li, Xin, Zhang, Aiying. Dynamic Changes in Serum Markers and Their Utility in the Early Diagnosis of All Stages of Hepatitis B-Associated Hepatocellular Carcinoma. OncoTargets and therapy 2020;13():827-40. | Duplicate (including Cochrane includes) |
| Wu, Min, Liu, Zhaobo, Li, Xin, Zhang, Aiying. Dynamic Changes in Serum Markers and Their Utility in the Early Diagnosis of All Stages of Hepatitis B-Associated Hepatocellular Carcinoma. OncoTargets and therapy 2020;13(101514322):827-40. | Does not meet full eligibility criteria: includes people without cirrhosis |
| Wu, Min, Liu, Zhaobo, Zhang, Aiying. Associated measurement of fucosylated levels of AFP, DCP, and GPC3 for early diagnosis in hepatocellular carcinoma. The International journal of biological markers 2019;34(1):20-26. | Exclude on population: not cirrhosis |
| Wu, Qiong, Zheng, Xubin, Leung, Kwong-Sak, Wong, Man-Hon, Tsui, Stephen Kwok-Wing. meGPS: a multi-omics signature for hepatocellular carcinoma detection integrating methylome and transcriptome data. Bioinformatics (Oxford, England) 2022;():n. pag.. | Exclude on population: not cirrhosis |
| Wu, Tong, Fan, Rong, Bai, Jian, Yang, Zhao, Qian, Yun-Song, Du, Lu-Tao, et al. The development of a cSMART-based integrated model for hepatocellular carcinoma diagnosis. Journal of hematology & oncology 2023;16(1):1. | Does not meet full eligibility criteria: includes people without cirrhosis |
| Wu, W., Yao, D. F., Qiu, L. W., Wu, X. H., Yao, M., Su, X. Q. Abnormal expression of hepatomas and circulating telomerase and its clinical values. Hepatobiliary and Pancreatic Diseases International 2005;4(4):544-49. | Does not meet full eligibility criteria: includes people without cirrhosis |
| Wu, W., Yao, D. F., Yuan, Y. M., Fan, J. W., Lu, X. F., Li, X. H., et al. Combined serum hepatoma-specific alpha-fetoprotein and circulating alpha-fetoprotein-mRNA in diagnosis of hepatocellular carcinoma. Hepatobiliary and Pancreatic Diseases International 2006;5(4):538-44. | Does not meet full eligibility criteria: includes people without cirrhosis |
| Wu, Wei, Yao, Deng-Fu, Yuan, Yong-Mei, Fan, Ji-Wei, Lu, Xiu-Feng, Li, Xiao-Hua, et al. Combined serum hepatoma-specific alpha-fetoprotein and circulating alpha-fetoprotein-mRNA in diagnosis of hepatocellular carcinoma. Hepatobiliary & pancreatic diseases international : HBPD INT 2006;5(4):538-44. | Does not meet full eligibility criteria: includes people without cirrhosis |
| Wu, X., Zhou, J., Sun, Y., Ding, H., Chen, G., Xie, W., et al. Prediction of liver-related events in patients with compensated HBV-induced cirrhosis receiving antiviral therapy. Hepatology International 2021;15(1):82-92. | Exclude on study design: not a 1- or 2-gate test accuracy study |
| Wu, Xue-Min, Xi, Zhi-Feng, Liao, Pinhu, Huang, Hong-Dong, Huang, Xiao-Ying, Wang, Chao, et al. Diagnostic and prognostic potential of serum microRNA-4651 for patients with hepatocellular carcinoma related to aflatoxin B1. Oncotarget 2017;8(46):81235-49. | Does not meet full eligibility criteria: includes people without cirrhosis |
| Wu, Xue-Min, Xi, Zhi-Feng, Liao, Pinhu, Huang, Hong-Dong, Huang, Xiao-Ying, Wang, Chao, et al. Diagnostic and prognostic potential of serum microRNA-4651 for patients with hepatocellular carcinoma related to aflatoxin B1. Oncotarget 2017;8(46):81235-49. | Duplicate (including Cochrane includes) |
| Wu, Y. T., Ran, X. J., Li, Q. Z., Wu, Y. Q., Shen, X. X., Mu, M. Level and clinical significance of serum CXC chemokine ligand 13 in patients with hepatocellular carcinoma. Translational Cancer Research 2025;14(1):424 " 433. | Exclude on population: not cirrhosis |
| Wu, Y., Chen, Y., Wei, L., Ding, Z., Zhao, S., Bao, S., et al. The Value of CEUS LI-RADS combined with AFP in early diagnosis of hepatocellular carcinoma in low- and high-risk patients. Journal of Cancer Research and Therapeutics 2024;20(4):1274 " 1283. | Exclude on population: clinical signs and symptoms of HCC |
| Wu, Y., Fan, Z., Tian, Y., Zhang, L. Establishment and Validation of a Blood Test-based Nomogram to Diagnose Patients with AFP-negative HCC. Current Cancer Drug Targets 2024;24(5):556 " 564. | No 2x2 data |

| Reference | Reason for exclusion |
|---|---|
| Wu, Y., Huang, L., Li, B. Original article the diagnostic value of gd-eob-dtpa-enhanced mri scans in small hepatocellular carcinoma in patients with liver cirrhosis. International Journal of Clinical and Experimental Medicine 2020;13(9):6909-15. | Exclude on population: clinical signs and symptoms of HCC |
| Wu, Y., Wang, M., Zhang, Z., Chen, G. A Novel Nomogram Model for Predicting the Risk of Hepatocellular Carcinoma in Patients with Chronic Hepatitis B Infection. Journal of Hepatocellular Carcinoma 2025;12():765 “ 775. | Exclude on population: not cirrhosis |
| Wu, Y., Zhang, X., Zeng, H., Liu, Y., Wu, Q., Chen, Y., et al. Angiopoietin-2 (Ang-2) is a useful serum tumor marker for liver cancer in the Chinese population. Clinica Chimica Acta 2018;478():18-27. | Exclude on population: not cirrhosis |
| Wu, Z. M. [Delayed Tc-99m-PMT imaging in the specific diagnosis of hepatocellular carcinoma]. Zhonghua zhong liu za zhi [Chinese journal of oncology] 1988;10(5):360-63. | Does not meet full eligibility criteria: pre-2005 |
| Wu, Zhixian, Cheng, Hongwei, Liu, Jie, Zhang, Shuaishuai, Zhang, Minda, Liu, Fangzhou, et al. The Oncogenic and Diagnostic Potential of Stanniocalcin 2 in Hepatocellular Carcinoma. Journal of hepatocellular carcinoma 2022;9():141-55. | Does not meet full eligibility criteria: includes people without cirrhosis |
| Wun, Y. T. Alpha-fetoprotein and/or liver ultrasonography for liver cancer screening in patients with chronic hepatitis B. The Cochrane database of systematic reviews 2003;(2):CD002799. | Exclude on study design: not a 1- or 2-gate test accuracy study |
| Xian, Meng-Fei, Huang, Yang, Xie, Wen-Xuan, Pan, Kang-Ming, Zeng, Dan, Huang, Hui, et al. LR-M Observations on Contrast-Enhanced Ultrasound: Detection of Hepatocellular Carcinoma Using Additional Features in Comparison With Current LI-RADS Criteria. AJR. American journal of roentgenology 2022;219(1):76-85. | Exclude on population: clinical signs and symptoms of HCC |
| Xiang, Lin, Chen, La-Mei, Zhai, Yu-Jia, Sun, Wei-Juan, Yang, Jie-Ru, Fan, Yu-Chen. Hypermethylation of secreted frizzled related protein 2 gene promoter serves as a noninvasive biomarker for HBV-associated hepatocellular carcinoma. Life sciences 2021;270():119061. | Exclude on population: not cirrhosis |
| Xiang, X., Amdur, R., Yu, H., Wong, L. L., Rao, S., He, A. R., et al. BLOOD-BASED TGF-beta PATHWAY FUNCTIONAL BIOMARKERS FOR DETECTING HEPATOCELLULAR CANCER (HCC). Gastroenterology 2022;162(7 Supplement):S-891. | CONFERENCE ABSTRACT |
| Xiang, X., Bhowmick, K., Shetty, K., Ohshiro, K., Yang, X., Wong, L. L., et al. Mechanistically based blood proteomic markers in the tgf-s pathway stratify risk of hepatocellular cancer in patients with cirrhosis. Genes and Cancer 2024;15():1 “ 14. | No 2x2 data |
| Xiao, J., Long, F., Peng, T., Hu, L. B., Cai, H., Chen, R. Development and potential application of a simultaneous multiplex assay of Golgi protein 73 and alpha-fetoprotein for hepatocellular carcinoma diagnosis. European review for medical and pharmacological sciences 2019;23(8):3302-10. | Exclude on population: healthy controls |
| Xiao, Jun Feng, Varghese, Rency S., Zhou, Bin, Nezami Ranjbar, Mohammad R., Zhao, Yi, Tsai, Tsung-Heng, et al. LC-MS based serum metabolomics for identification of hepatocellular carcinoma biomarkers in Egyptian cohort. Journal of proteome research 2012;11(12):5914-23. | Exclude on index test |
| Xiao, Junfeng, Zhao, Yi, Varghese, Rency S., Zhou, Bin, Di Poto, Cristina, Zhang, Lihua, et al. Evaluation of metabolite biomarkers for hepatocellular carcinoma through stratified analysis by gender, race, and alcoholic cirrhosis. Cancer epidemiology, biomarkers & prevention : a publication of the American Association for Cancer Research, cosponsored by the American Society of Preventive Oncology 2014;23(1):64-72. | No 2x2 data |
| Xiao, Xiaojiao & Zhao, Jianfeng. Task relevance driven adversarial learning for simultaneous detection, size grading, and quantification of hepatocellular carcinoma via integrating multi-modality MRI. Medical image analysis 2022;81():102554. | Exclude on population: healthy controls |
| Xiaohua, L. & Kaichun, W. Serum Golgi phosphoprotein 2 level: A better marker than alpha-fetoprotein for diagnosing early hepatocellular carcinoma. Hepatology 2009;50(1):325. | Does not meet full eligibility criteria: includes people without cirrhosis |

| Reference | Reason for exclusion |
|---|---|
| Xie, Guo-Feng, Xu, Yu-Xia, Xu, Fei, Sun, Li-Yue, Ye, Zu-Lu, Ma, Jiang-Jun, Wang, Hai-Yun. Plasma SGIP1 methylation in diagnosis and prognosis prediction in hepatocellular carcinoma. Neoplasma 2021;68(1):62-70. | Does not meet full eligibility criteria: includes people without cirrhosis |
| Xie, L. Diagnostic Value of Combined Serum Marker Tests in Hepatitis B Virus-Associated Hepatocellular Carcinoma. Alternative therapies in health and medicine 2024;30(5):168 " 173. | Does not meet full eligibility criteria: includes people without cirrhosis |
| Xie, W. & Zhu, Z. The value of serum QSOX1, Gp73 and AFP alone or in combination in the diagnosis of primary liver cancer. Acta Medica Mediterranea 2020;36(3):1515-19. | Exclude on population: not cirrhosis |
| Xie, Yun, Yao, Qinwei, Butt, Azeem Mehmood, Guo, Jia, Tian, Zhou, Bao, Xuli, et al. Expression profiling of serum microRNA-101 in HBV-associated chronic hepatitis, liver cirrhosis, and hepatocellular carcinoma. Cancer biology & therapy 2014;15(9):1248-55. | Does not meet full eligibility criteria: includes people without cirrhosis |
| Xie, Z. Value of gut microbiota changes and 1ger/1gfp in patients with chronic liver disease in diagnosing liver cancer. Acta Medica Mediterranea 2021;37(6):3369-73. | Exclude on target condition: not HCC |
| Xie, Zijun, Zhou, Fangyuan, Yang, Yidong, Li, Leijia, Lei, Yiming, Lin, Xianyi, et al. Lnc-PCDH9-13:1 Is a Hypersensitive and Specific Biomarker for Early Hepatocellular Carcinoma. EBioMedicine 2018;33():57-67. | Does not meet full eligibility criteria: includes people without cirrhosis |
| Xing, H., Qiu, H., Ding, X., Han, J., Li, Z., Wu, H., et al. Clinical performance of alpha-L-fucosidase for early detection of hepatocellular carcinoma. Biomarkers in Medicine 2019;13(7):545-55. | Duplicate (including Cochrane includes) |
| Xing, Hao, Qiu, Haibo, Ding, Xuemei, Han, Jun, Li, Zhenli, Wu, Han, et al. Clinical performance of alpha-L-fucosidase for early detection of hepatocellular carcinoma. Biomarkers in medicine 2019;13(7):545-55. | Does not meet full eligibility criteria: includes people without cirrhosis |
| Xing, Hao, Zheng, Yi-Jie, Han, Jun, Zhang, Han, Li, Zhen-Li, Lau, Wan-Yee, Shen, Feng. Protein induced by vitamin K absence or antagonist-II versus alpha-fetoprotein in the diagnosis of hepatocellular carcinoma: A systematic review with meta-analysis. Hepatobiliary & pancreatic diseases international : HBPD INT 2018;17(6):487-95. | Exclude on study design: not a 1- or 2-gate test accuracy study |
| Xing, T. J., Jiang, D. F., Huang, J. X. Expression and clinical significance of miR-122 and miR-29 in hepatitis B virus-related liver disease. Genetics and molecular research : GMR 2014;13(3):7912-18. | Exclude on study design: not a 1- or 2-gate test accuracy study |
| Xu, A. F., Wang, M. C., Sui, D. M., Yuan, Y. H., Chen, G. Subject diagnostic value of detecting a1pha-fetoprotein variants with a new microspincolumn method in hepatocellular carcinoma. Zhonghua shi yan he lin chuang bing du xue za zhi = Zhonghua shiyan he linchuang bingduxue zazhi = Chinese journal of experimental and clinical virology 2007;21(1):67-69. | Foreign language |
| Xu, Cheng, Zeng, Xiang-Hua, Wang, Li, Tao, Shi-Qi, Wu, Quan-Xin, Zhu, Peng, Deng, Guo-Hong. sFRP-4, a potential novel serum marker for chronic hepatitis B-related hepatocellular carcinoma. Hepatobiliary & pancreatic diseases international : HBPD INT 2015;14(2):164-70. | Exclude on population: not cirrhosis |
| Xu, F., Li, X. L., Wang, Y. M. Study on diagnostic significance of GPC3 in the patients with primary hepatocellular carcinoma. Journal of Dalian Medical University 2013;35(4):381-83. | Foreign language |
| Xu, Fei, Zhang, Lulu, He, Wenting, Song, Di, Ji, Xiaomeng. The Diagnostic Value of Serum PIVKA-II Alone or in Combination with AFP in Chinese Hepatocellular Carcinoma Patients. Disease markers 2021;2021():8868370. | Does not meet full eligibility criteria: includes people without cirrhosis |
| Xu, Fei, Zhang, Lulu, Xu, Yuxia, Song, Di, He, Wenting, Ji, Xiaomeng. Hypermethylation of SCAND3 and Myo1g Gene Are Potential Diagnostic Biomarkers for Hepatocellular Carcinoma. Cancers 2020;12(8):n. pag.. | Does not meet full eligibility criteria: includes people without cirrhosis |
| Xu, H., Zhu, X., Xu, Z., Hu, Y., Bo, S., Xing, T. Non-invasive analysis of genomic copy number variation in patients with hepatocellular carcinoma by next generation DNA sequencing. Journal of Cancer 2015;6(3):247-53. | Exclude on population: not cirrhosis |
| Xu, Hong, Chen, Yueming, Dong, Xueyan. Serum Exosomal Long Noncoding RNAs ENSG00000258332.1 and LINC00635 for the Diagnosis and Prognosis of Hepatocellular Carcinoma. Cancer epidemiology, biomarkers & prevention : a publication of the American Association for Cancer Research, cosponsored by the American Society of Preventive Oncology 2018;27(6):710-16. | Exclude on population: not cirrhosis |

| Reference | Reason for exclusion |
|---|---|
| Xu, Hong, Dong, Xueyan, Chen, Yueming. Serum exosomal hnRNPH1 mRNA as a novel marker for hepatocellular carcinoma. Clinical chemistry and laboratory medicine 2018;56(3):479-84. | Duplicate (including Cochrane includes) |
| Xu, Hong, Dong, Xueyan, Chen, Yueming. Serum exosomal hnRNPH1 mRNA as a novel marker for hepatocellular carcinoma. Clinical chemistry and laboratory medicine 2018;56(3):479-84. | Does not meet full eligibility criteria: includes people without cirrhosis |
| Xu, J. B., Qi, F. Z., Xu, G., Chen, G. F., Qin, L. X. Value of alpha-fetoprotein and clinical characteristics in patients with liver neoplasm. Neoplasma 2014;61(2):218-24. | Exclude on population: clinical signs and symptoms of HCC |
| Xu, J., Chen, B., Qi, J., Wu, J., Feng, W., Jin, K., et al. Evaluation of serum hsa_tsr014055 as a potential biomarker for diagnosis and prognosis of hepatocellular carcinoma. Annals of Medicine 2025;57(1):2528978. | Exclude on population: not cirrhosis |
| Xu, Jian, Wu, Chen, Che, Xu, Wang, Li, Yu, Dianke, Zhang, Tongwen, et al. Circulating microRNAs, miR-21, miR-122, and miR-223, in patients with hepatocellular carcinoma or chronic hepatitis. Molecular carcinogenesis 2011;50(2):136-42. | Exclude on population: not cirrhosis |
| Xu, K. C. & Wei, Q. Serum alpha-1-antitrypsin and alpha-1-antichymotrypsin in the diagnosis of primary hepatocellular carcinoma. Chinese medical journal 1989;102(11):834-38. | Exclude on index test |
| Xu, K., Meng, X. Y., Wu, J. W., Shen, B., Shi, Y. C. Diagnostic value of serum gamma-glutamyl transferase isoenzyme for hepatocellular carcinoma: a 10-year study. The American journal of gastroenterology 1992;87(8):991-95. | Does not meet full eligibility criteria: pre-2005 |
| Xu, L., Wei, B., Hui, H. Association of serum microRNA-125b and HBV-related hepatocellular carcinoma in chinese han patients. International Journal of Clinical and Experimental Medicine 2018;11(4):3699-7003. | Does not meet full eligibility criteria: includes people without cirrhosis |
| Xu, P. J., Yan, F. H., Wang, J. H., Lin, J., Ji, Y., Chen, C. Z., Shen, J. Z. The value of breath-hold diffusion-weighted imaging in small hepatocellular carcinoma lesion (<=3 cm) detection. National Medical Journal of China 2009;89(9):592-96. | Foreign language |
| Xu, Peng-Ju, Yan, Fu-Hua, Wang, Jian-Hua, Lin, Jiang. Added value of breathhold diffusion-weighted MRI in detection of small hepatocellular carcinoma lesions compared with dynamic contrast-enhanced MRI alone using receiver operating characteristic curve analysis. Journal of magnetic resonance imaging : JMRI 2009;29(2):341-49. | Exclude on population: clinical signs and symptoms of HCC |
| Xu, W. J., Guo, B. L., Han, Y. G., Shi, L. RETRACTED ARTICLE:Diagnostic value of alpha-fetoprotein-L3 and Golgi protein 73 in hepatocellular carcinomas with low AFP levels. Tumor Biology 2014;35(12):12069-74. | Retracted |
| Xu, Wen-fang, Fei, Ying-ming, Zhou, Jian-kang, Shen, Hua-jing, Chen, Xue-fang, Lv, Qiu-qiong. [Significance of serum golgi protein 73 (GP73), alpha-fetoprotein (AFP) and lectin-reactive alpha-fetoprotein (AFP-L3) expresssion in primary hepatic carcinoma]. Zhonghua shi yan he lin chuang bing du xue za zhi = Zhonghua shiyan he linchuang bingduxue zazhi = Chinese journal of experimental and clinical virology 2011;25(4):286-88. | Foreign language |
| Xu, X., Xiao, X., Huang, C., Gao, Z., Ji, J., Fang, M. Establishment of lectin-ELISA for sialylated fetuin-A and its diagnostic value in primary hepatocellular carcinoma. Chinese Journal of Laboratory Medicine 2022;45(4):366-72. | Foreign language |
| Xu, Yuan, Zhang, Bei, Zhou, Fan, Yi, Ying-Ping, Yang, Xin-Lei, Ouyang, Xiao. Development of machine learning-based personalized predictive models for risk evaluation of hepatocellular carcinoma in hepatitis B virus-related cirrhosis patients with low levels of serum alpha-fetoprotein. Annals of hepatology 2024;29(6):101540. | Exclude on index test |
| Xu, Zhong-Ping, Liu, Yan, Wu, Zhen-Ru, Gong, Jian-Ping. Prognostic and diagnostic value of SOX9 in cirrhotic and noncirrhotic hepatocellular carcinoma. Translational cancer research 2021;10(6):2738-46. | No 2x2 data |
| Yagin, F. H., El Shawi, R., Algarni, A., Colak, C., Al-Hashem, F. Metabolomics Biomarker Discovery to Optimize Hepatocellular Carcinoma Diagnosis: Methodology Integrating AutoML and Explainable Artificial Intelligence. Diagnostics 2024;14(18):2049. | Exclude on index test |

| Reference | Reason for exclusion |
|---|---|
| Yahya, Raida S., Ghanem, Osman H., Foyouh, Abdel-Aziz A., Atwa, Mohamed. Role of interleukin-8 and oxidative stress in patients with hepatocellular carcinoma. Clinical laboratory 2013;59(9-10):969-76. | Exclude on population: not cirrhosis |
| Yamagamim, Hiroaki, Moriyama, Mitsuhiko, Matsumura, Hiroshi, Aoki, Hiroshi, Shimizu, Toshihiro, Saito, Takahide, et al. Serum concentrations of human hepatocyte growth factor is a useful indicator for predicting the occurrence of hepatocellular carcinomas in C-viral chronic liver diseases. Cancer 2002;95(4):824-34. | Exclude on index test |
| Yamamoto, T., Amuro, Y., Matsuda, Y., Nakaoka, H., Shimomura, S., Hada, T. Boronate affinity chromatography of gamma-glutamyltransferase in patients with hepatocellular carcinoma. American Journal of Gastroenterology 1991;86(4):495-99. | Does not meet full eligibility criteria: pre-2005 |
| Yamamoto, Yusuke, Kondo, Shunsuke, Matsuzaki, Juntaro, Esaki, Minoru, Okusaka, Takuji, Shimada, Kazuaki, et al. Highly Sensitive Circulating MicroRNA Panel for Accurate Detection of Hepatocellular Carcinoma in Patients With Liver Disease. Hepatology communications 2020;4(2):284-97. | Does not meet full eligibility criteria: includes people without cirrhosis |
| Yamasaki, Kazumi, Tateyama, Masakuni, Abiru, Seigo, Komori, Atsumasa, Nagaoka, Shinya, Saeki, Akira, et al. Elevated serum levels of Wisteria floribunda agglutinin-positive human Mac-2 binding protein predict the development of hepatocellular carcinoma in hepatitis C patients. Hepatology (Baltimore, Md.) 2014;60(5):1563-70. | Exclude on study design: not a 1- or 2-gate test accuracy study |
| Yamashiki, N., Sugawara, Y., Tamura, S., Kaneko, J., Yoshida, H., Aoki, T., et al. Diagnostic accuracy of alpha-fetoprotein and des-gamma-carboxy prothrombin in screening for hepatocellular carcinoma in liver transplant candidates. Hepatology Research 2011;41(12):1199-2007. | Does not meet full eligibility criteria: includes people without cirrhosis |
| Yamashita, Satoyoshi, Kato, Akira, Akatsuka, Toshitaka, Sawada, Takashi, Asai, Tomohide, Koyama, Noriyuki. Clinical relevance of increased serum preneoplastic antigen in hepatitis C-related hepatocellular carcinoma. World journal of gastroenterology 2020;26(13):1463-73. | Exclude on population: not cirrhosis |
| Yamashita, Taro, Koshikawa, Naohiko, Shimakami, Tetsuro, Terashima, Takeshi, Nakagawa, Masatoshi, Nio, Kouki, et al. Serum Laminin gamma2 Monomer as a Diagnostic and Predictive Biomarker for Hepatocellular Carcinoma. Hepatology (Baltimore, Md.) 2021;74(2):760-75. | Exclude on population: not cirrhosis |
| Yamashita, Y., Mitsuzaki, K., Yi, T., Ogata, I., Nishiharu, T., Urata, J. Small hepatocellular carcinoma in patients with chronic liver damage: prospective comparison of detection with dynamic MR imaging and helical CT of the whole liver. Radiology 1996;200(1):79-84. | Does not meet full eligibility criteria: pre-2005 |
| Yamauchi, Kazuhiko, Akbar, S. M. Fazle, Horiike, Norio, Michitaka, Kojiro. Increased serum levels of macrophage inflammatory protein-3alpha in hepatocellular carcinoma: relationship with clinical factors and prognostic importance during therapy. International journal of molecular medicine 2003;11(5):601-05. | Exclude on target condition: not HCC |
| Yamauchi, Naoko, Watanabe, Akira, Hishinuma, Michiyo, Ohashi, Ken-Ichi, Midorikawa, Yutaka, Morishita, Yasuyuki, et al. The glypican 3 oncofetal protein is a promising diagnostic marker for hepatocellular carcinoma. Modern pathology : an official journal of the United States and Canadian Academy of Pathology, Inc 2005;18(12):1591-98. | Exclude on population: tissue samples |
| Yan, C. Y., Fu, Z. J., Liu, G. S., Li, J. Value of heat shock protein 90 combined with alpha-fetoprotein in early diagnosis of hepatocellular carcinoma. World Chinese Journal of Digestology 2021;29(21):1215-21. | Foreign language |
| Yan, Dong, He, Qingfang, Chen, Yaping, Wang, Lixin. Detection of alpha-fetoprotein and glypican-3 mRNAs in the peripheral blood of hepatocellular carcinoma patients by using multiple FQ-RT-PCR. Journal of clinical laboratory analysis 2011;25(2):113-17. | Does not meet full eligibility criteria: includes people without cirrhosis |
| Yan, Linlin, Chen, Yanhui, Zhou, Jiyuan, Zhao, Hong, Zhang, Henghui. Diagnostic value of circulating cell-free DNA levels for hepatocellular carcinoma. International journal of infectious diseases : IJID : official publication of the International Society for Infectious Diseases 2018;67():92-97. | Duplicate (including Cochrane includes) |
| Yan, Linlin, Chen, Yanhui, Zhou, Jiyuan, Zhao, Hong, Zhang, Henghui. Diagnostic value of circulating cell-free DNA levels for hepatocellular | Exclude on population: not cirrhosis |

| Reference | Reason for exclusion |
|---|---|
| carcinoma. International journal of infectious diseases : IJID : official publication of the International Society for Infectious Diseases 2018;67(c3r, 9610933):92-97. | |
| Yan, S. Optimizing early screening for hepatocellular carcinoma: Diagnostic value of ultrasonography combined with serum biomarkers. Medicine (United States) 2025;104(28):e43205. | Does not meet full eligibility criteria: includes people without cirrhosis |
| Yang, C., Li, N., Wang, Y., Zhang, P., Zhu, Q., Li, F., et al. Serum levels of b-cell activating factor in chronic hepatitis b virus infection: Association with clinical diseases. Journal of Interferon and Cytokine Research 2014;34(10):787-94. | Exclude on population: not cirrhosis |
| Yang, Chao, Geng, Huijuan, Zhu, Shanshan, Zheng, Xiaomeng, Li, Tiemin. Multiple Diagnostic Indicators in the Development of Chronic Hepatitis B, Liver Cirrhosis, and Liver Cancer. Alternative therapies in health and medicine 2023;29(3):153-59. | Does not meet full eligibility criteria: includes people without cirrhosis |
| Yang, Hao, Chen, Guo-Dong, Fang, Feng, Liu, Zhen, Lau, Stephanie Hiu Yan, Zhang, Jin-Fan, Lau, Wan Yee. Dickkopf-1: as a diagnostic and prognostic serum marker for early hepatocellular carcinoma. The International journal of biological markers 2013;28(3):286-97. | Does not meet full eligibility criteria: includes people without cirrhosis |
| Yang, Hyun Kyung, Lee, Sunyoung, Lee, Min Young. Effectiveness of noncontrast-abbreviated magnetic resonance imaging in a real-world hepatocellular carcinoma surveillance. European radiology 2025;35(9):5792â€“5800. | No 2x2 data |
| Yang, J. D. Surveillance for hepatocellular carcinoma in patients with cirrhosis. Clinical Gastroenterology and Hepatology 2012;10(1):16-21. | Exclude on study design: not a 1- or 2-gate test accuracy study |
| Yang, J. D., Kim, E., Pedersen, R. A., Kim, W. R., Pungpapong, S. Utility of serum YKL-40 as a tumor-specific marker of hepatobiliary malignancies. Gut and Liver 2010;4(4):537-42. | Exclude on study design: not a 1- or 2-gate test accuracy study |
| Yang, J. R., Tian, Y. X., Li, J. E., Zhang, Y., Fan, Y. C. Mex3a promoter hypomethylation can be utilized to diagnose HBV-associated hepatocellular carcinoma: a randomized controlled trial. Frontiers in Pharmacology 2024;15():1325869. | Exclude on population: not cirrhosis |
| Yang, Jie-Ru, Wang, Ju, Li, Hai-Ming, Gao, Shuai, Fan, Yu-Chen. IL-6 Promoter Hypomethylation Acts As a Diagnostic Biomarker in Hepatitis B Virus-Associated Hepatocellular Carcinoma. Frontiers in oncology 2022;12():746643. | Exclude on population: not cirrhosis |
| Yang, Ju Dong, Addissie, Benyam D, Mara, Kristin C, Harmsen, William S, Dai, Jianliang, Zhang, Ning, et al. GALAD Score for Hepatocellular Carcinoma Detection in Comparison with Liver Ultrasound and Proposal of GALADUS Score. Cancer epidemiology, biomarkers & prevention : a publication of the American Association for Cancer Research, cosponsored by the American Society of Preventive Oncology 2019;28(3):531-38. | Does not meet full eligibility criteria: includes people without cirrhosis |
| Yang, Ju Dong, Addissie, Benyam D., Mara, Kristin C., Harmsen, William S., Dai, Jianliang, Zhang, Ning, et al. GALAD Score for Hepatocellular Carcinoma Detection in Comparison with Liver Ultrasound and Proposal of GALADUS Score. Cancer epidemiology, biomarkers & prevention : a publication of the American Association for Cancer Research, cosponsored by the American Society of Preventive Oncology 2019;28(3):531-38. | Duplicate (including Cochrane includes) |
| Yang, Ju Dong, Dai, Jianliang, Singal, Amit G, Gopal, Purva, Addissie, Benyam D, Nguyen, Mindie H, et al. Improved Performance of Serum Alpha-Fetoprotein for Hepatocellular Carcinoma Diagnosis in HCV Cirrhosis with Normal Alanine Transaminase. Cancer epidemiology, biomarkers & prevention : a publication of the American Association for Cancer Research, cosponsored by the American Society of Preventive Oncology 2017;26(7):1085-92. | Secondary publication of included study |
| Yang, Jun, Xu, Guowang, Zheng, Yufang, Kong, Hongwei, Pang, Tao, Lv, Shen. Diagnosis of liver cancer using HPLC-based metabonomics avoiding false-positive result from hepatitis and hepatocirrhosis diseases. Journal of chromatography. B, Analytical technologies in the biomedical and life sciences 2004;813(1-2):59-65. | Exclude on target condition: not HCC |

| Reference | Reason for exclusion |
| --- | --- |
| Yang, L., Wei, C., Li, Y., He, X. MiR-224 is an early-stage biomarker of hepatocellular carcinoma with miR-224 and miR-125b as prognostic biomarkers. Biomarkers in Medicine 2020;14(15):1485-5000. | Exclude on study design: not a 1- or 2-gate test accuracy study |
| Yang, L., Xu, Q., Xie, H., Gu, G. Expression of serum miR-218 in hepatocellular carcinoma and its prognostic significance. Clinical & translational oncology : official publication of the Federation of Spanish Oncology Societies and of the National Cancer Institute of Mexico 2016;18(8):841-47. | Exclude on population: not cirrhosis |
| Yang, Lei, Rong, Weiqi, Xiao, Ting, Zhang, Ying, Xu, Bin, Liu, Yu, et al. Secretory/releasing proteome-based identification of plasma biomarkers in HBV-associated hepatocellular carcinoma. Science China. Life sciences 2013;56(7):638-46. | Does not meet full eligibility criteria: includes people without cirrhosis |
| Yang, Meng-Yuan, Wu, Fan, Fang, Feng, Yang, Hao, Zhang, Jing-Fan, Chen, Guo-Dong. Serum epidermal growth factor-like domain 7 serves as a novel diagnostic marker for early hepatocellular carcinoma. BMC cancer 2021;21(1):772. | Does not meet full eligibility criteria: includes people without cirrhosis |
| Yang, Qian, Ye, Hua, Sun, Guiying, Wang, Keyan, Dai, Liping, Qiu, Cuipeng, et al. Human Proteome Microarray identifies autoantibodies to tumor-associated antigens as serological biomarkers for the diagnosis of hepatocellular carcinoma. Molecular oncology 2023;17(5):887-900. | Does not meet full eligibility criteria: includes people without cirrhosis |
| Yang, Shi-zhong, Dong, Jia-hong, Zhu, Jin, Li, Kun. [Detection of alpha-fetoproteins mRNA and melanoma antigen-1 mRNA in peripheral blood of patients with hepatocellular carcinoma and its clinical significance]. Zhonghua wai ke za zhi [Chinese journal of surgery] 2004;42(17):1060-63. | Does not meet full eligibility criteria: pre-2005 |
| Yang, Tian, Xing, Hao, Wang, Guoqiang, Wang, Nianyue, Liu, Miaoxia, Yan, Cunling, et al. A Novel Online Calculator Based on Serum Biomarkers to Detect Hepatocellular Carcinoma among Patients with Hepatitis B. Clinical chemistry 2019;65(12):1543-53. | Does not meet full eligibility criteria: includes people without cirrhosis |
| Yang, X. & Shen, D. [Clinical evaluation of serum transforming growth factor beta 1 assay in the diagnosis of primary hepatic carcinoma]. Zhonghua nei ke za zhi 1997;36(10):669-72. | Does not meet full eligibility criteria: pre-2005 |
| Yang, Xiao-Ping, Zhou, Li-Xing, Yang, Qi-Jun, Liu, Ling, Cai, Yang. Diagnostic and prognostic roles of serum vitronectin in hepatitis B-related hepatocellular carcinoma. Cancer biomarkers : section A of Disease markers 2016;17(3):271-79. | Does not meet full eligibility criteria: includes people without cirrhosis |
| Yang, Y. J., Chen, H., Huang, P., Li, C. H., Dong, Z. H. Quantification of plasma hTERT DNA in hepatocellular carcinoma patients by quantitative fluorescent polymerase chain reaction. Clinical and Investigative Medicine 2011;34(4):E238-44. | Exclude on population: not cirrhosis |
| Yang, Yang, Fan, Yu-Chen, Gao, Shuai, Dou, Cheng-Yun, Zhang, Jian-Jun, Sun, Feng-Kai. Methylated cysteine dioxygenase-1 gene promoter in the serum is a potential biomarker for hepatitis B virus-related hepatocellular carcinoma. The Tohoku journal of experimental medicine 2014;232(3):187-94. | Does not meet full eligibility criteria: includes people without cirrhosis |
| Yang, Yang, Fan, Yu-Chen, Gao, Shuai, Dou, Cheng-Yun, Zhang, Jian-Jun, Sun, Feng-Kai. Methylated cysteine dioxygenase-1 gene promoter in the serum is a potential biomarker for hepatitis B virus-related hepatocellular carcinoma. The Tohoku journal of experimental medicine 2014;232(3):187-94. | Does not meet full eligibility criteria: includes people without cirrhosis |
| Yangmei, S. Z., Chen, G., Dang, Y. W., Chen, S. Clinical significance of elevated serum DcR3 in patients with hepatocellular carcinoma. World Chinese Journal of Digestology 2009;17(20):2042-47. | Foreign language |
| Yanming, L., Yue, C., Wencan, C., Liangyin, W. U., Xijun, L., Jiafeng, Z. Combined detection of AFP-L3, GP73 and TIP30 enhances diagnostic accuracy for HBV-related cirrhosis and hepatocellular carcinoma. JPMA. The Journal of the Pakistan Medical Association 2019;69(9):1279-86. | Does not meet full eligibility criteria: includes people without cirrhosis |
| Yao, D. F., Wu, W., Yao, M., Qui, L. W., Wu, X. H., Su, X. Q., et al. Dynamic alteration of telomerase expression and its diagnostic significance in liver or peripheral blood for hepatocellular carcinoma. World Journal of Gastroenterology 2006;12(31):4966-72. | Exclude on index test |
| Yao, M. & Pan, L. H. Glypican-3 as a specific biomarker for hepatocellular carcinoma. Hepatobiliary and Pancreatic Diseases International 2015;14(2):122-23. | Exclude on study design: not a 1- or 2-gate test accuracy study |

| Reference | Reason for exclusion |
|---|---|
| Yao, Min, Yao, Deng-Fu, Bian, Yin-Zhu, Wu, Wei, Yan, Xiao-Di, Yu, Dan-Dan, et al. Values of circulating GPC-3 mRNA and alpha-fetoprotein in detecting patients with hepatocellular carcinoma. Hepatobiliary & pancreatic diseases international : HBPD INT 2013;12(2):171-79. | Does not meet full eligibility criteria: includes people without cirrhosis |
| Yao, Mingjie & Zhao, Jingmin. Alpha-fetoprotein still is a valuable diagnostic and prognosis predicting biomarker in hepatitis B virus infection-related hepatocellular carcinoma. Oncotarget 2016;7(4):3702-8. | Does not meet full eligibility criteria: includes people without cirrhosis |
| Yao, Weirong, Wang, Kaiyu, Jiang, Yu, Huang, Zhufeng, Huang, Yiyun, Yan, Huihui, et al. Serum profile of low molecular weight fucosylated glycoproteins for early diagnosis of hepatocellular carcinoma. Oncology letters 2020;20(2):1597-6006. | Exclude on index test |
| Yao, Zhicheng, Jia, Changchang, Tai, Yan, Liang, Hao, Zhong, Zhaozhong, Xiong, Zhiyong, Deng, Meihai. Serum exosomal long noncoding RNAs lnc-FAM72D-3 and lnc-EPC1-4 as diagnostic biomarkers for hepatocellular carcinoma. Aging 2020;12(12):11843-63. | Does not meet full eligibility criteria: includes people without cirrhosis |
| Yasmin Anum, M. Y., Looi, M. L., Nor Aini, A. H., Merican, I., Wahidah, A., Mohd Radzi, A. H., Nor Azizah, A. Combined assessment of TGF-beta-1 and alpha-fetoprotein values improves specificity in the diagnosis of hepatocellular carcinoma and other chronic liver diseases in Malaysia. The Medical journal of Malaysia 2009;64(3):223-27. | Exclude on population: not cirrhosis |
| Yasser, Montaser Bellah, Abdellatif, Mahmoud, Emad, Esraa, Jafer, Afnan, Ahmed, Samar, Nageb, Lobna, et al. Circulatory miR-221 & miR-542 expression profiles as potential molecular biomarkers in Hepatitis C Virus mediated liver cirrhosis and hepatocellular carcinoma. Virus research 2021;296():198341. | Does not meet full eligibility criteria: includes people without cirrhosis |
| Yasuda, E., Kumada, T., Toyoda, H., Kaneoka, Y., Maeda, A., Okuda, S., Yoshimi, N. Evaluation for clinical utility of GPC3, measured by a commercially available ELISA kit with Glypican-3 (GPC3) antibody, as a serological and histological marker for hepatocellular carcinoma. Hepatology Research 2010;40(5):477-85. | Exclude on population: not cirrhosis |
| Ye, F., Song, Y., Yu, X. D., Zhang, H. M., Ouyang, H. Hepatobiliary phase imaging with Gd-EOB-DTPA for detection and characterization of hepatocellular carcinoma in patients with chronic liver disease. Chinese Journal of Medical Imaging Technology 2015;31(4):571-75. | Foreign language |
| Ye, Fei, Huang, Wei, Xue, Yuan, Tang, Erjiang, Wang, Mingjie, Shi, Fengchun, et al. Serum Levels of ITGBL1 as an Early Diagnostic Biomarker for Hepatocellular Carcinoma with Hepatitis B Virus Infection. Journal of hepatocellular carcinoma 2021;8():285-300. | Does not meet full eligibility criteria: includes people without cirrhosis |
| Ye, X., Li, C., Zu, X., Lin, M., Liu, Q., Liu, J., et al. A Large-Scale Multicenter Study Validates Aldo-Keto Reductase Family 1 Member B10 as a Prevalent Serum Marker for Detection of Hepatocellular Carcinoma. Hepatology 2019;69(6):2489-5001. | Duplicate (including Cochrane includes) |
| Ye, Xu, Li, Cunyan, Zu, Xuyu, Lin, Minglin, Liu, Qiang, Liu, Jianghua, et al. A Large-Scale Multicenter Study Validates Aldo-Keto Reductase Family 1 Member B10 as a Prevalent Serum Marker for Detection of Hepatocellular Carcinoma. Hepatology (Baltimore, Md.) 2019;69(6):2489-5001. | Does not meet full eligibility criteria: includes people without cirrhosis |
| Yeh, C. T. & Wei, J. S. Biliary alkaline phosphatase measured by mini-column chromatography on DEAE-cellulose: application to detection of hepatobiliary diseases. Clinical chemistry 1989;35(8):1684-87. | Does not meet full eligibility criteria: pre-2005 |
| Yen, Chih-Wei, Kuo, Yuan-Hung, Wang, Jing-Houng, Chang, Kuo-Chin, Kee, Kwong-Ming, Hung, Shu-Feng, et al. Did AFP-L3 save ultrasonography in community screening? The Kaohsiung journal of medical sciences 2018;34(10):583-87. | Exclude on population: not cirrhosis |
| Yeo, Injoon, Kim, Gi-Ae, Kim, Hyunsoo, Lee, Ji Hyeon, Sohn, Areum, Gwak, Geum-Youn, et al. Proteome Multimarker Panel With Multiple Reaction Monitoring-Mass Spectrometry for Early Detection of Hepatocellular Carcinoma. Hepatology communications 2020;4(5):753-68. | Does not meet full eligibility criteria: includes people without cirrhosis |
| Yi, C. H., Weng, H. L., Zhou, F. G., Fang, M., Ji, J., Cheng, C., et al. Elevated core-fucosylated IgG is a new marker for hepatitis B virus-related hepatocellular carcinoma. OncoImmunology 2015;4(12):n. pag.. | Does not meet full eligibility criteria: includes people without cirrhosis |

| Reference | Reason for exclusion |
|---|---|
| Yi, Kezhen, Zhang, Zhonglin, Chen, Peng, Xi, Xiaodan, Zhao, Xudong, Rong, Yuan, et al. Tidal microfluidic chip-based isolation and transcriptomic profiling of plasma extracellular vesicles for clinical monitoring of high-risk patients with hepatocellular carcinoma-associated precursors. Biosensors & bioelectronics 2025;276():117228. | Exclude on population: not cirrhosis |
| Yin, Haidi, Lin, Zhenxin, Nie, Song, Wu, Jing, Tan, Zhijing, Zhu, Jianhui, et al. Mass-selected site-specific core-fucosylation of ceruloplasmin in alcohol-related hepatocellular carcinoma. Journal of proteome research 2014;13(6):2887-96. | Does not meet full eligibility criteria: includes people without cirrhosis |
| Yin, Haidi, Tan, Zhijing, Wu, Jing, Zhu, Jianhui, Shedden, Kerby A., Marrero, Jorge. Mass-Selected Site-Specific Core-Fucosylation of Serum Proteins in Hepatocellular Carcinoma. Journal of proteome research 2015;14(11):4876-84. | No 2x2 data |
| Yin, Z. F., Tu, Z. X., Cui, Z. F. Alpha-fetoprotein reaction to Pisum sativum agglutinin in differentiation of benign liver diseases from hepatocellular carcinoma. Chinese medical journal 1993;106(8):615-18. | Does not meet full eligibility criteria: includes people without cirrhosis |
| Ying, Xia, Zhao, Yue, Wang, Jun-Lan, Zhou, Xia, Zhao, Jing, He, Chen-Chen, et al. Serum anti-osteopontin autoantibody as a novel diagnostic and prognostic biomarker in patients with hepatocellular carcinoma. Oncology reports 2014;32(4):1550-56. | No 2x2 data |
| Yoo, Hye Jin, Lee, Jeong Min, Lee, Jae Young, Kim, Se Hyung, Kim, Soo Jin, Han, Joon Koo. Additional value of SPIO-enhanced MR imaging for the noninvasive imaging diagnosis of hepatocellular carcinoma in cirrhotic liver. Investigative radiology 2009;44(12):800-07. | Exclude on population: clinical signs and symptoms of HCC |
| Yoon, Eileen L., Yeon, Jong Eun, Ko, Eunjung, Lee, Hyun Jung, Je, Ji Hye, Yoo, Yang Jae, et al. An Explorative Analysis for the Role of Serum miR-10b-3p Levels in Predicting Response to Sorafenib in Patients with Advanced Hepatocellular Carcinoma. Journal of Korean medical science 2017;32(2):212-20. | No 2x2 data |
| Yoon, J. H., Kim, Y. K., Kim, J. W., Chang, W., Choi, J. I., Park, B. J., et al. Comparison of Four Diagnostic Guidelines for Hepatocellular Carcinoma Using Gadoxetic Acid-enhanced Liver MRI. ghted imaging; Radiology 2024;311(1):e233114. | Exclude on population: clinical signs and symptoms of HCC |
| Yoon, Ki Woong & Song, Ji Soo. The usefulness of the sum of relative enhancement ratio in making a differential diagnosis of hepatocellular carcinoma from cirrhosis-related nodules. Clinical imaging 2014;38(2):154-59. | Exclude on population: clinical signs and symptoms of HCC |
| Yoon, Seung Kew, Lim, Nam Kyu, Ha, Seon-Ah, Park, Yong Gyu, Choi, Jong Young, Chung, Kyu Won, et al. The human cervical cancer oncogene protein is a biomarker for human hepatocellular carcinoma. Cancer research 2004;64(15):5434-41. | Does not meet full eligibility criteria: includes people without cirrhosis |
| Yoon, Sungjin, Shim, Young Sup, Park, So Hyun, Sung, Jaekon, Nickel, Marcel Dominik, Kim, Ye Jin, Lee, Hee Young. Hepatobiliary phase imaging in cirrhotic patients using compressed sensing and controlled aliasing in parallel imaging results in higher acceleration. European radiology 2024;34(4):2233â€“2243. | Exclude on population: clinical signs and symptoms of HCC |
| Yoon, Young Joon & Han, Kwang-Hyub. Role of serum prothrombin induced by vitamin K absence or antagonist-II in the early detection of hepatocellular carcinoma in patients with chronic hepatitis B virus infection. Scandinavian journal of gastroenterology 2009;44(7):861-6. | Does not meet full eligibility criteria: includes people without cirrhosis |
| Yoon, Young Joon & Han, Kwang-Hyub. Role of serum prothrombin induced by vitamin K absence or antagonist-II in the early detection of hepatocellular carcinoma in patients with chronic hepatitis B virus infection. Scandinavian journal of gastroenterology 2009;44(7):861-66. | Duplicate (including Cochrane includes) |
| Yosef, Tarek, Ibrahim, Wesam Ahmed, Matboli, Marwa, Swilam, Amina Ahmed. New stem cell autophagy surrogate diagnostic biomarkers in early-stage hepatocellular carcinoma in Egypt: A pilot study. World journal of hepatology 2021;13(12):2137-49. | Exclude on population: not cirrhosis |
| Yoshida, K. New Biomarkers for Early Detection of Hepatocellular Carcinoma. EBioMedicine 2015;2(5):370-71. | Exclude on study design: not a 1- or 2-gate test accuracy study |
| Yosry, A., Fouad, R., Hafez, H. A., Al Arab, M. E., Gohar, M. Transient elastography can predict the risk of hepatocellular carcinoma in Egyptian | Exclude on population: not cirrhosis |

| Reference | Reason for exclusion |
|---|---|
| patients with chronic hepatitis C. Journal of Gastroenterology and Hepatology Research 2013;2(7):687-91. | |
| Yosry, Ayman, Zayed, Naglaa, Dawood, Reham M., Ibrahim, Marwa K., Elsharkawy, Marwa, Ekladious, Sherif M., et al. Highly Sensitive Serum miRNA Panel for the Diagnosis of Hepatocellular Carcinoma in Egyptian Patients with HCV-Related HCC. Laboratory medicine 2022;53(5):523-29. | Exclude on population: not cirrhosis |
| Yosry, Ayman, Zayed, Naglaa, Dawood, Reham M., Ibrahim, Marwa K., Elsharkawy, Marwa, Ekladious, Sherif M., et al. Highly Sensitive Serum miRNA Panel for the Diagnosis of Hepatocellular Carcinoma in Egyptian Patients with HCV-Related HCC. Laboratory medicine 2022;():n. pag.. | Exclude on population: not cirrhosis |
| Younis, Yehia Sadek, Alegaily, Hatem Samir, Elagawy, Waleed, Semeya, Atteyat Aboelmaged, Abo-Amer, Yousry Esam-Eldin, El-Abgeegy, Mohamed, et al. Serum Dickopff 1 as a Novel Biomarker in Hepatocellular Carcinoma Diagnosis and Follow Up After Ablative Therapy. Cancer management and research 2019;11():10555-62. | Exclude on population: not cirrhosis |
| Youns, Mahmoud M, Abdel Wahab, Abdel Hady A, Hassan, Zeinab A. Serum talin-1 is a potential novel biomarker for diagnosis of hepatocellular carcinoma in Egyptian patients. Asian Pacific journal of cancer prevention : APJCP 2013;14(6):3819-23. | Does not meet full eligibility criteria: includes people without cirrhosis |
| Youns, Mahmoud M., Abdel Wahab, Abdel Hady A., Hassan, Zeinab A. Serum talin-1 is a potential novel biomarker for diagnosis of hepatocellular carcinoma in Egyptian patients. Asian Pacific journal of cancer prevention : APJCP 2013;14(6):3819-23. | Does not meet full eligibility criteria: includes people without cirrhosis |
| Yousef, Eman H., El-Mesery, Mohamed E., Habeeb, Maha R. Polo-like kinase 1 as a promising diagnostic biomarker and potential therapeutic target for hepatocellular carcinoma. Tumour biology : the journal of the International Society for Oncodevelopmental Biology and Medicine 2020;42(4):1010428320914475. | Exclude on population: healthy controls |
| Youssef, A. A., Issa, H. A., Omar, M. Z., Behiry, E. G., Elfallah, A. A., Hasaneen, A., Darwish, M. Serum human endothelial cell-specific molecule-I (Endocan) and vascular endothelial growth factor in cirrhotic HCV patients with hepatocellular carcinoma as predictors of mortality. Clinical and Experimental Gastroenterology 2018;11():431-38. | Exclude on population: healthy controls |
| Youssef, Samar Samir, Youness, Rana Ahmed, Abbas, Eman Abd El-Razek, Osman, Noha Mohamed, Elfiky, Asmaa. miR-516a-3P, a potential circulating biomarker in hepatocellular carcinoma, correlated with rs738409 polymorphism in PNPLA3. Personalized medicine 2022;19(6):483-93. | Exclude on population: not cirrhosis |
| Yu, Bin, Zhou, Shujun, Liang, Han, Ye, Qifa. Development and Validation of a Novel Circulating miRNA-Based Diagnostic Score for Early Detection of Hepatocellular Carcinoma. Digestive diseases and sciences 2022;67(6):2283-92. | Exclude on population: not cirrhosis |
| Yu, Chuanwen. Diagnostic Value of Multislice Spiral Computed Tomography Combined with Serum AFP, TSGF, and GP73 Assay in the Diagnosis of Primary Liver Cancer. Evidence-based complementary and alternative medicine : eCAM 2022;2022():6581127. | Exclude on population: healthy controls |
| Yu, Fujun, Lu, Zhongqiu, Chen, Bicheng, Dong, Peihong. microRNA-150: a promising novel biomarker for hepatitis B virus-related hepatocellular carcinoma. Diagnostic pathology 2015;10():129. | Exclude on population: not cirrhosis |
| Yu, Jian, Ding, Wen-Bing, Wang, Meng-Chao, Guo, Xing-Gang, Xu, Jian, Xu, Qing-Guo, et al. Plasma circular RNA panel to diagnose hepatitis B virus-related hepatocellular carcinoma: A large-scale, multicenter study. International journal of cancer 2020;146(6):1754-63. | Does not meet full eligibility criteria: includes people without cirrhosis |
| Yu, Jian, Ding, Wen-Bing, Wang, Meng-Chao, Guo, Xing-Gang, Xu, Jian, Xu, Qing-Guo, et al. Plasma circular RNA panel to diagnose hepatitis B virus-related hepatocellular carcinoma: A large-scale, multicenter study. International journal of cancer 2020;146(6):1754-63. | Duplicate (including Cochrane includes) |
| Yu, L., Wang, X., Zhang, X., Liu, Z., Guo, Y., Wang, M., Han, Q. Construction and validation of a nomogram for predicting the incidence of hepatocellular carcinoma based on serum abnormal prothrombin and alpha-fetoprotein. Chinese Journal of Hepatobiliary Surgery 2025;31(1):1 “ 5. | Foreign language |

| Reference | Reason for exclusion |
|---|---|
| Yu, Nam C., Chaudhari, Vinika, Raman, Steven S., Lassman, Charles, Tong, Myron J., Busuttil, Ronald W. CT and MRI improve detection of hepatocellular carcinoma, compared with ultrasound alone, in patients with cirrhosis. Clinical gastroenterology and hepatology : the official clinical practice journal of the American Gastroenterological Association 2011;9(2):161-67. | Duplicate (including Cochrane includes) |
| Yu, Rentao, Ding, Shitao, Tan, Wenting, Tan, Shun, Tan, Zhaoxia, Xiang, Shiqing, et al. Performance of Protein Induced by Vitamin K Absence or Antagonist-II (PIVKA-II) for Hepatocellular Carcinoma Screening in Chinese Population. Hepatitis monthly 2015;15(7):e28806. | Exclude on population: not cirrhosis |
| Yu, Rentao, Tan, Zhaoxia, Xiang, Xiaomei, Dan, Yunjie. Effectiveness of PIVKA-II in the detection of hepatocellular carcinoma based on real-world clinical data. BMC cancer 2017;17(1):608. | No 2x2 data |
| Yu, Rentao, Xiang, Xiaomei, Tan, Zhaoxia, Zhou, Yi, Wang, Haoliang. Corrigendum: Efficacy of PIVKA-II in prediction and early detection of hepatocellular carcinoma: a nested case-control study in Chinese patients. Scientific reports 2016;6():39184. | Exclude on study design: not a 1- or 2-gate test accuracy study |
| Yu, Rentao, Xiang, Xiaomei, Tan, Zhaoxia, Zhou, Yi, Wang, Haoliang. Efficacy of PIVKA-II in prediction and early detection of hepatocellular carcinoma: a nested case-control study in Chinese patients. Scientific reports 2016;6():35050. | Duplicate (including Cochrane includes) |
| Yu, Rentao, Xiang, Xiaomei, Tan, Zhaoxia, Zhou, Yi, Wang, Haoliang. Efficacy of PIVKA-II in prediction and early detection of hepatocellular carcinoma: a nested case-control study in Chinese patients. Scientific reports 2016;6(101563288):35050. | Does not meet full eligibility criteria: includes people without cirrhosis |
| Yu, Zhou, Chen, Dongmei, Zheng, Yansong, Wang, Xuedan, Huang, Shuna, Lin, Tiansheng, et al. Development and validation of a diagnostic model for AFP-negative hepatocellular carcinoma. Journal of cancer research and clinical oncology 2023;149(13):11295-30808. | No 2x2 data |
| Yuan, Weidong, Sun, Yong, Liu, Ling, Zhou, Bing, Wang, Shuming. Circulating LncRNAs Serve as Diagnostic Markers for Hepatocellular Carcinoma. Cellular physiology and biochemistry : international journal of experimental cellular physiology, biochemistry, and pharmacology 2017;44(1):125-32. | Exclude on population: not cirrhosis |
| Yuan, Xiao-Dong, Wang, Jing-Wen, Fang, Yu, Qian, Yu, Gao, Shuai, Fan, Yu-Chen. Methylation status of the T-cadherin gene promotor in peripheral blood mononuclear cells is associated with HBV-related hepatocellular carcinoma progression. Pathology, research and practice 2020;216(5):152914. | Exclude on population: not cirrhosis |
| Yuan, Y., Yin, D., Yang, X., Liu, D., Shan, H., Luo, J., Li, X. Plasma lipidomic analysis reveals disruption of ether phosphatidylcholine biosynthesis and facilitates early detection of hepatitis B-related hepatocellular carcinoma. Lipids in Health and Disease 2025;24(1):69. | Does not meet full eligibility criteria: includes people without cirrhosis |
| Yuen, M. F. Screening for hepatocellular carcinoma: Survival benefit and cost-effectiveness. Annals of Oncology 2003;14(10):1463-67. | Exclude on study design: not a 1- or 2-gate test accuracy study |
| Yun-Fan, Liaw & Dar-In, Tai. Early detection of hepatocellular carcinoma in patients with chronic type B hepatitis. A prospective study. Gastroenterology 1986;90(2):263-67. | Exclude on population: not cirrhosis |
| Yutani, S., Tanaka, M., Mutsumoto, H., Imai, N., Sata, M., Shichijo, S., Harada, M. Elevation of serum MAGE-4 protein levels and prediction of hepatocellular carcinogenesis in patients with liver cirrhosis. Japanese Journal of Cancer Research 2002;93(4):453-58. | Does not meet full eligibility criteria: pre-2005 |
| Yvamoto, E. Y., Ferreira, R. F., Nogueira, V., Pinhel, M. A. S., Tenani, G. D., Andrade, J. G. S. C., et al. Influence of vascular endothelial growth factor and alpha-fetoprotein on hepatocellular carcinoma. Genetics and Molecular Research 2015;14(4):17453-62. | Does not meet full eligibility criteria: includes people without cirrhosis |
| Zahid, M., Adnan, S., Sadaf, R., Khattak, M. I. Frequency of hepatocellular carcinoma in patients with HCV induced liver cirrhosis. Journal of Medical Sciences (Peshawar) 2012;20(4):168-70. | Exclude on study design: not a 1- or 2-gate test accuracy study |
| Zakhary, Nadia I., Khodeer, Sherif M., Shafik, Hanan E. Impact of PIVKA-II in diagnosis of hepatocellular carcinoma. Journal of advanced research 2013;4(6):539-46. | Exclude on population: not cirrhosis |

| Reference | Reason for exclusion |
|---|---|
| Zakir, Umaira, Siddiqui, Nadir Naveed, Naqvi, Faizan-Ul-Hassan. Aberrant STAT1 methylation as a non-invasive biomarker in blood of HCV induced hepatocellular carcinoma. Cancer biomarkers : section A of Disease markers 2022;34(1):95-103. | Exclude on population: healthy controls |
| Zekri, A. R. N., Moharram, R. A. N., Mohamed, W. S., Bahnassy, A. A., Alam El-Din, H. M., Abo-Shadi, M. M., et al. Disease progression from chronic hepatitis C to cirrhosis and hepatocellular carcinoma is associated with repression of interferon regulatory factor-1. European Journal of Gastroenterology and Hepatology 2010;22(4):450-56. | Genomic biomarker not validated |
| Zekri, A. R. N., Youssef, A. S. E. D., El-Desouky, E. D., Ahmed, O. S., Lotfy, M. M., Nassar, A. A. M. Serum microRNA panels as potential biomarkers for early detection of hepatocellular carcinoma on top of HCV infection. Tumor Biology 2016;37(9):12273-86. | No 2x2 data |
| Zekri, Abdel-Rahman N., Bahnassy, Abeer A., Alam El-Din, Hanaa M., Morsy, Heba M., Shaarawy, Sabry, Moharram, Nagia Z. Serum levels of beta-catenin as a potential marker for genotype 4/hepatitis C-associated hepatocellular carcinoma. Oncology reports 2011;26(4):825-31. | Exclude on index test |
| Zekri, Abdel-Rahman Nabawy, Youssef, Amira Salah El-Din, Bakr, Yasser Mabrouk, Gabr, Reham Mohamed, Ahmed, Ola Sayed, Elberry, Mostafa Hamed, et al. Early detection of hepatocellular carcinoma co-occurring with hepatitis C virus infection: A mathematical model. World journal of gastroenterology 2016;22(16):4168-82. | Does not meet full eligibility criteria: includes people without cirrhosis |
| Zekri, Abdel-Rahman Nabawy, Youssef, Amira Salah El-Din, El-Desouky, Eman Desouky, Ahmed, Ola Sayed, Lotfy, Mai M., Nassar, Auhood Abdel-Monem. Serum microRNA panels as potential biomarkers for early detection of hepatocellular carcinoma on top of HCV infection. Tumour biology : the journal of the International Society for Oncodevelopmental Biology and Medicine 2016;37(9):12273-86. | No 2x2 data |
| Zekri, Abdel-Rahman, El-Kassas, Mohamed, Saad, Yasmin, Bahnassy, Abeer, El-Din, Hany Khatab Sameh Seif, Darweesh, Samar K, Abdel Hafez, Hanan. Caspase recruitment domains. New potential markers for diagnosis of hepatocellular carcinoma associated with HCV in Egyptian patients. Annals of hepatology 2013;12(5):774-81. | Exclude on population: not cirrhosis |
| Zekri, Abdel-Rahman, El-Kassas, Mohamed, Saad, Yasmin, Bahnassy, Abeer, El-Din, Hany Khatab Sameh Seif, Darweesh, Samar K., Abdel Hafez, Hanan. Caspase recruitment domains. New potential markers for diagnosis of hepatocellular carcinoma associated with HCV in Egyptian patients. Annals of hepatology 2013;12(5):774-81. | Exclude on population: not cirrhosis |
| Zekri, Abdel-Rahman, Youssef, Amira Salah El-Din, Bakr, Yasser Mabrouk, Gabr, Reham Mohamed, El-Rouby, Mahmoud Nour El-Din, Hammad, Ibtisam, et al. Serum biomarkers for early detection of hepatocellular carcinoma associated with HCV infection in egyptian patients. Asian Pacific journal of cancer prevention : APJCP 2015;16(3):1281-87. | Exclude on index test |
| Zeng, Jun, Huang, Xin, Zhou, Lina, Tan, Yexiong, Hu, Chunxiu, Wang, Xiaomei, et al. Metabolomics Identifies Biomarker Pattern for Early Diagnosis of Hepatocellular Carcinoma: from Diethylnitrosamine Treated Rats to Patients. Scientific reports 2015;5():16101. | No 2x2 data |
| Zeng, Jun, Yin, Peiyuan, Tan, Yexiong, Dong, Liwei, Hu, Chunxiu, Huang, Qiang, et al. Metabolomics study of hepatocellular carcinoma: discovery and validation of serum potential biomarkers by using capillary electrophoresis-mass spectrometry. Journal of proteome research 2014;13(7):3420-31. | Does not meet full eligibility criteria: includes people without cirrhosis |
| Zenlander, R., Salter, H., Gilg, S., Eggertsen, G. MicroRNAs as Plasma Biomarkers of Hepatocellular Carcinoma in Patients with Liver Cirrhosis-A Cross-Sectional Study. International Journal of Molecular Sciences 2024;25(4):2414. | No 2x2 data |
| Zenlander, Robin, Fredolini, Claudia, Schwenk, Jochen M., Ryden, Ingvar, Pahlsson, Peter, Lowbeer, Christian, Eggertsen, Gosta. A wide scan of plasma proteins demonstrates thioredoxin reductase 1 as a potential new diagnostic biomarker for hepatocellular carcinoma. Scandinavian journal of gastroenterology 2023;58(9):998â€“1008. | No 2x2 data |

| Reference | Reason for exclusion |
|---|---|
| Zertuche-Martinez, Cecilia, Velazquez-Enriquez, Juan Manuel, Gonzalez-Garcia, Karina, Baltierrez-Hoyos, Rafael, Carrasco-Torres, Gabriela, Garcia-Roman, Rebeca, et al. Identification of ABCC3 and its isoforms as potential biomarker in hepatocellular carcinoma. Toxicology mechanisms and methods 2024;34(4):398â€“407. | Exclude on study design: not a 1- or 2-gate test accuracy study |
| Zezulinski, D., Hoteit, M. A., Kaplan, D. E., Simeone, A., Zhan, T., Doria, C., et al. Detection of Circulating mRNA Variants in Hepatocellular Carcinoma Patients Using Targeted RNAseq. Liver Cancer 2025;():n. pag.. | No 2x2 data |
| Zhan, X., Fang, M., Xiao, X., Ji, J. The value of core fucosylated alpha 2 macroglobulin in the diagnosis of hepatocellular carcinoma. Chinese Journal of Laboratory Medicine 2019;42(3):193-97. | Foreign language |
| Zhan, Z., Guan, Y., Mew, K., Zeng, W., Peng, M., Hu, P., et al. Urine alpha-fetoprotein and orosomucoid 1 as biomarkers of hepatitis B virus-associated hepatocellular carcinoma. American Journal of Physiology - Gastrointestinal and Liver Physiology 2020;318(2):G305-12. | Duplicate (including Cochrane includes) |
| Zhan, Zhu, Guan, Yalan, Mew, Kenley, Zeng, Weiqiong, Peng, Mingli, Hu, Peng, et al. Urine alpha-fetoprotein and orosomucoid 1 as biomarkers of hepatitis B virus-associated hepatocellular carcinoma. American journal of physiology. Gastrointestinal and liver physiology 2020;318(2):G305-12. | Does not meet full eligibility criteria: includes people without cirrhosis |
| Zhang, Delin, Huang, Jianzhao, Luo, Dan, Feng, Xinfu, Liu, Yan. Glycosylation change of alpha-1-acid glycoprotein as a serum biomarker for hepatocellular carcinoma and cirrhosis. Biomarkers in medicine 2017;11(5):423-30. | Exclude on index test |
| Zhang, H., Chen, X., Yang, B., Zhang, M. Research on the serum level of microRNA-224 in hepatocellular carcinoma patients and its clinical diagnostic significance. Chinese Journal of Clinical Oncology 2014;41(9):576-79. | Foreign language |
| Zhang, Hai-Jian, Yao, Deng-Fu, Yao, Min, Huang, Hua, Wu, Wei, Yan, Mei-Juan, Yan, Xiao-Di. Expression characteristics and diagnostic value of annexin A2 in hepatocellular carcinoma. World journal of gastroenterology 2012;18(41):5897-9004. | Does not meet full eligibility criteria: includes people without cirrhosis |
| Zhang, Haikun, Dong, Peiling, Guo, Shicheng, Tao, Chengcheng, Chen, Wei, Zhao, Wenmin, et al. Hypomethylation in HBV integration regions aids non-invasive surveillance to hepatocellular carcinoma by low-pass genome-wide bisulfite sequencing. BMC medicine 2020;18(1):200. | Exclude on population: not cirrhosis |
| Zhang, Hong, Huang, Dong-feng, Wei, Qun, Hua, Ting-yan, Li, Feng. [Expression and its clinicopathological features of tumor rejective antigen 1 in human tissues of hepatocellular carcinoma and liver cirrhosis]. Zhonghua gan zang bing za zhi = Zhonghua ganzangbing zazhi = Chinese journal of hepatology 2010;18(9):661-65. | Foreign language |
| Zhang, J. M., Zhao, S. X., Liu, L. D., Han, F., Ren, W. G., Wu, X. Q., et al. Study on the value of abnormal prothrombin in the diagnosis of HBV-related hepatocellular carcinoma. Zhonghua gan zang bing za zhi = Zhonghua ganzangbing zazhi = Chinese journal of hepatology 2025;33(4):340 " 347. | Foreign language |
| Zhang, J. W., Guan, L. Y., E, C. Y., Yang, J. H., Xuan, W., Meng, Z. H. [The value of serum abnormal prothrombin in clinical application of hepatocellular carcinoma]. Zhonghua wai ke za zhi [Chinese journal of surgery] 2020;58(10):776-81. | Foreign language |
| Zhang, J., Hao, W., Liu, X., Meng, Y., Liu, J., Wu, L., et al. Proteome microarray identifies autoantibody biomarkers for diagnosis of hepatitis B-related hepatocellular carcinoma. Clinica Chimica Acta 2024;554():117727. | No 2x2 data |
| Zhang, Jiyun, Lai, Zhizhen, Ding, Rui, Zhou, Jinyu, Yuan, Zhonghao, Li, Dan, et al. Diagnostic potential of site-specific serotransferrin N-glycosylation in discriminating different liver diseases. Clinica chimica acta; international journal of clinical chemistry 2023;539():175-83. | Exclude on target condition: not HCC |
| Zhang, L. X. ghted imaging; Abbreviated MRI for Hepatocellular Carcinoma Surveillance: Counterpoint-Hepatobiliary Contrast Agents Improve Detection and Characterization. ghted imaging; American Journal of Roentgenology 2024;222(4):n. pag.. | Exclude on study design: not a 1- or 2-gate test accuracy study |
| Zhang, Lei, Wu, Guang-Ye, Wu, Yu-Jing. The serum metabolic profiles of different Barcelona stages hepatocellular carcinoma associated with hepatitis B virus. Oncology letters 2018;15(1):956-62. | No 2x2 data |

| Reference | Reason for exclusion |
|---|---|
| Zhang, Lijie, Liu, Hui, Sun, Lin, Li, Ning, Ding, Huiguo. Glypican-3 as a potential differential diagnosis marker for hepatocellular carcinoma: a tissue microarray-based study. Acta histochemica 2012;114(6):547-52. | Exclude on population: tissue samples |
| Zhang, P. J., Run, W. W., P, Liang, Wang, C. B., Deng, X. X., Wang, B. B., et al. Peripheral blood mRNA expression patterns to differentiate hepatocellular carcinoma from other hepatic diseases. Frontiers in bioscience (Elite edition) 2012;4():620-30. | Exclude on population: not cirrhosis |
| Zhang, Pengjun, Wen, Xinyu, Gu, Feng, Deng, Xinxin, Li, Juan, Dong, Jin, Jiao, Jiao. Methylation profiling of serum DNA from hepatocellular carcinoma patients using an Infinium Human Methylation 450 BeadChip. Hepatology international 2013;7(3):893-900. | Exclude on population: healthy controls |
| Zhang, Qi, Xu, Hai-Feng, Song, Wen-Yue, Zhang, Peng-Jun. Potential microRNA panel for the diagnosis and prediction of overall survival of hepatocellular carcinoma with hepatitis B virus infection. World journal of gastrointestinal oncology 2020;12(4):383-93. | Exclude on population: tissue samples |
| Zhang, Qian-Yun, Chen, Hui, Lin, Zhen. Chemiluminescence enzyme immunoassay based on magnetic nanoparticles for detection of hepatocellular carcinoma marker glypican-3. Journal of pharmaceutical analysis 2011;1(3):166-74. | Exclude on index test |
| Zhang, S. Y., Cao, N., Chen, M. K., Zhang, L. D., Bai, Y. Q. Diagnostic value of plasma HLA-F adjacent transcript 10 mRNA expression in patients with hepatocellular carcinoma or colon cancer. World Chinese Journal of Digestology 2015;23(36):5775-83. | Foreign language |
| Zhang, S., Chen, J., Shu, H., Shen, S., Li, Y., Lu, X., et al. Autoantibody signature in hepatocellular carcinoma using seromics. Journal of Hematology and Oncology 2020;13(1):85. | Does not meet full eligibility criteria: includes people without cirrhosis |
| Zhang, S., Gao, C., Wang, Y., Chen, L. Evaluating the combined diagnostic power of alpha-fetoprotein and protein induced by vitamin K absence or antagonist-II for hepatocellular carcinoma. Journal of Gastrointestinal Oncology 2025;16(3):1157 “ 1175. | Exclude on population: not cirrhosis |
| Zhang, S., Tuo, P., Ji, Y., Huang, Z., Xiong, Z., Li, H. Identification of 1-Methylnicotinamide as a specific biomarker for the progression of cirrhosis to hepatocellular carcinoma. Journal of Cancer Research and Clinical Oncology 2024;150(6):310. | Exclude on study design: not a 1- or 2-gate test accuracy study |
| Zhang, S., Wu, M., Chen, H., Tu, Z., Cui, Z., Huang, L., et al. The implication of serum glycosidase enzymogram in the diagnosis of hepatocellular carcinoma. Clinical Chemistry and Enzymology Communications 1991;4(1):9-19. | Does not meet full eligibility criteria: pre-2005 |
| Zhang, Shu, Jiang, Kai, Zhang, Qinle, Guo, Kun. Serum fucosylated paraoxonase 1 as a potential glycobiomarker for clinical diagnosis of early hepatocellular carcinoma using ELISA Index. Glycoconjugate journal 2015;32(3-4):119-25. | Does not meet full eligibility criteria: includes people without cirrhosis |
| Zhang, Shu, Liu, Yuming, Chen, Jing, Shu, Hong, Shen, Siyun, Li, Yin, et al. Autoantibody signature in hepatocellular carcinoma using seromics. Journal of hematology & oncology 2020;13(1):85. | Does not meet full eligibility criteria: includes people without cirrhosis |
| Zhang, T., Zhang, G., Deng, X., Zeng, J., Jin, J., Huang, Z., Wu, M. APS (age, platelets, 2D shear-wave elastography) score predicts hepatocellular carcinoma in chronic hepatitis B. Radiology 2021;301(2):350-59. | Exclude on population: not cirrhosis |
| Zhang, Ting, Yin, Minggang, Wang, Lizhen, Cao, Wenzhai. Diagnostic performance of blood-based liquid biopsies in hepatocellular carcinoma: A protocol for systematic review and meta-analysis. Medicine 2020;99(41):e22594. | Exclude on study design: not a 1- or 2-gate test accuracy study |
| Zhang, W., Chen, Z., Xue, C., Zhang, Y., Wu, L., Zhu, J., et al. The Applicability of ADA, AFU, and LAC in the Early Diagnosis and Disease Risk Assessment of Hepatitis B-Associated Liver Cirrhosis and Hepatocellular Carcinoma. Frontiers in Medicine 2021;8():740029. | Exclude on index test |
| Zhang, Wei-Bin, Hou, Si-Ze, Chen, Yan-Ling, Mao, Feng, Dong, Yi, Chen, Jian-Gang. Deep Learning for Approaching Hepatocellular Carcinoma Ultrasound Screening Dilemma: Identification of alpha-Fetoprotein-Negative Hepatocellular Carcinoma From Focal Liver Lesion Found in High-Risk Patients. Frontiers in oncology 2022;12():862297. | Exclude on population: clinical signs and symptoms of HCC |

| Reference | Reason for exclusion |
|---|---|
| Zhang, Weilu, Fu, Ting, Guo, Zhenjun, Zhang, Ye, Zhang, Lei, Su, Haixia, et al. Serum miR-375 Levels Are Closely Related to Disease Progression from HBV Infection to HBV-Related Hepatocellular Carcinoma. BioMed research international 2020;2020():5819385. | Duplicate (including Cochrane includes) |
| Zhang, Weilu, Fu, Ting, Guo, Zhenjun, Zhang, Ye, Zhang, Lei, Su, Haixia, et al. Serum miR-375 Levels Are Closely Related to Disease Progression from HBV Infection to HBV-Related Hepatocellular Carcinoma. BioMed research international 2020;2020(101600173):5819385. | Does not meet full eligibility criteria: includes people without cirrhosis |
| Zhang, Wu, Zhao, Cheng Guang, Sun, Hong Yu, Zheng, Wei E. Expression characteristics of KAI1 and vascular endothelial growth factor and their diagnostic value for hepatocellular carcinoma. Gut and liver 2014;8(5):536-42. | Exclude on population: not cirrhosis |
| Zhang, X. P. & Wang, K. Surveillance for Early-Stage Hepatocellular Carcinoma by Ultrasound Plus Alpha-Fetoprotein Measurement: More Details, More Significance. Gastroenterology 2018;155(4):1274-75. | Exclude on study design: not a 1- or 2-gate test accuracy study |
| Zhang, X., Wang, T., Zhang, K. H., Chen, S. H., He, Y. T. Simple Clinical Metrics Enhance AFP to Effectively Identify Cirrhotic Patients With Complicating Hepatocellular Carcinoma at Various AFP Levels. Frontiers in Oncology 2020;9():1478. | Duplicate (including Cochrane includes) |
| Zhang, X., Wang, Z., Tang, W., Wang, X., Liu, R., Chen, X., et al. Ultrasensitive and affordable assay for early detection of primary liver cancer using plasma cell-free DNA fragmentomics. Hepatology 2022;76(2):317-29. | Exclude on population: not cirrhosis |
| Zhang, Xianwei, Zhou, Hu, Jing, Wei, Luo, Ping, Qiu, Shili, Liu, Xuefang, et al. The Circular RNA hsa_circ_0001445 Regulates the Proliferation and Migration of Hepatocellular Carcinoma and May Serve as a Diagnostic Biomarker. Disease markers 2018;2018():3073467. | Does not meet full eligibility criteria: includes people without cirrhosis |
| Zhang, Xiaolian, Lu, Yu, Rong, Chengzhi, Yang, Dongmei, Li, Shan. Role of superoxide dismutase in hepatitis B virus-related hepatocellular carcinoma. Journal of research in medical sciences : the official journal of Isfahan University of Medical Sciences 2016;21():94. | Exclude on population: not cirrhosis |
| Zhang, Xiaoqing, Thiefin, Gerard, Gobinet, Cyril, Untereiner, Valerie, Taleb, Imane, Bernard-Chabert, Brigitte, et al. Profiling serologic biomarkers in cirrhotic patients via high-throughput Fourier transform infrared spectroscopy: toward a new diagnostic tool of hepatocellular carcinoma. Translational research : the journal of laboratory and clinical medicine 2013;162(5):279-86. | Exclude on index test |
| Zhang, Y., Sheng, R., Qian, X., Wang, H., Wu, F., Dai, H., et al. ghted imaging; Deep learning empowered gadolinium-free contrast-enhanced abbreviated MRI for diagnosing hepatocellular carcinoma. ghted imaging; JHEP Reports 2025;7(5):101392. | Exclude on population: not cirrhosis |
| Zhang, Y., Wang, J. W., Su, X., Li, J. E., Wei, X. F., Yang, J. R., et al. F-box protein 43 promoter methylation as a novel biomarker for hepatitis B virus-associated hepatocellular carcinoma. Frontiers in Microbiology 2023;14():1267844. | Exclude on population: not cirrhosis |
| Zhang, Y., Xi, Y., Fang, J., Luo, S., Wilson, J. J. Identification and characterization of monoclonal antibodies against GP73 for use as a potential biomarker in liver cancer screening and diagnosis. Journal of Immunoassay and Immunochemistry 2016;37(4):390-406. | Exclude on population: not cirrhosis |
| Zhang, Y., Yang, B., Wang, Y., Gao, Y., Bai, T., Bai, Y. Methylation profile of tumor suppressor genes in the cell-free DNA of plasma in hepatocellular carcinoma. Chinese Journal of Clinical Oncology 2013;40(23):1436-40. | Foreign language |
| Zhang, Yigan, Xi, Huaze, Nie, Xin, Zhang, Peng, Lan, Ning, Lu, Ying, Liu, Jinrong. Assessment of miR-212 and Other Biomarkers in the Diagnosis and Treatment of HBV-infection-related Liver Diseases. Current drug metabolism 2019;20(10):785-98. | Does not meet full eligibility criteria: includes people without cirrhosis |
| Zhang, Yingyu, Xu, Hongqin, Chi, Xiumei, Fan, Yuxiang, Shi, Ying. High level of serum Cripto-1 in hepatocellular carcinoma, especially with hepatitis B virus infection. Medicine 2018;97(35):e11781. | Exclude on index test |
| Zhang, Yue, Zhang, Sihua, Liu, Jianhua, Zhang, Yunli, Liu, Yanjie, Shen, Shuang, et al. Identification of serum glycobiomarkers for Hepatocellular Carcinoma using lectin microarrays. Frontiers in immunology 2022;13():973993. | Does not meet full eligibility criteria: includes people without cirrhosis |

| Reference | Reason for exclusion |
|---|---|
| Zhang, Zhejia & Xu, Linyong. Screening serum biomarkers for early primary hepatocellular carcinoma using a phage display technique. Journal of clinical laboratory analysis 2011;25(6):402-08. | Exclude on index test |
| Zhang, Zhoujing, Ge, Shengxiang, Wang, Xiaomin, Yuan, Quan, Yan, Qiang, Ye, Huiming, et al. Serum miR-483-5p as a potential biomarker to detect hepatocellular carcinoma. Hepatology international 2013;7(1):199-207. | Exclude on population: not cirrhosis |
| Zhang, Zhu-qing, Meng, Hua, Wang, Nan, Liang, Li-na, Liu, Li-na, Lu, Shu-ming. Serum microRNA 143 and microRNA 215 as potential biomarkers for the diagnosis of chronic hepatitis and hepatocellular carcinoma. Diagnostic pathology 2014;9():135. | Exclude on population: not cirrhosis |
| Zhao, Dandan, Zhang, Xiaoxiao, Tang, Yuhui, Guo, Peilin, Ai, Rong, Hou, Mengmeng, et al. Identification and Validation of Novel Biomarkers for Hepatocellular Carcinoma, Liver Fibrosis/Cirrhosis and Chronic Hepatitis B via Transcriptome Sequencing Technology. Journal of hepatocellular carcinoma 2022;9():389-403. | Exclude on population: not cirrhosis |
| Zhao, H. J., Li, S. M., Zhang, H. J., Guo, Z. J., Fan, H. Y., Yang, G. R., Wang, L. Diagnostic and prognostic value of RASSF1A and APC gene methylation in plasma of liver cancer patients. Chinese Journal of Cancer Prevention and Treatment 2013;20(1):53-57. | Foreign language |
| Zhao, J., Guo, L. Y., Yang, J. M. Sublingual vein parameters, AFP, AFP-L3, and GP73 in patients with hepatocellular carcinoma. Genetics and molecular research : GMR 2015;14(2):7062-67. | No 2x2 data |
| Zhao, K., Xu, G., Li, N., Jiang, Y. Establishment and validation of a predictive model for HBV-positive hepatocellular carcinoma. Chinese Journal of Laboratory Medicine 2022;45(5):516-21. | Exclude on study design: not a 1- or 2-gate test accuracy study |
| Zhao, Li & Yang, Qian. Clinical Value Evaluation of microRNA-324-3p and Other Available Biomarkers in Patients With HBV Infection-Related Hepatocellular Carcinoma. Open forum infectious diseases 2021;8(6):ofab108. | Does not meet full eligibility criteria: includes people without cirrhosis |
| Zhao, Ning-Hui, Qian, Yu, Wu, Chen-Si, Wang, Jing-Wen, Fang, Yu, Fan, Xiao-Peng, et al. Diagnostic value of NKG2D promoter methylation in hepatitis B virus-associated hepatocellular carcinoma. Biomarkers in medicine 2019;13(13):1093-1005. | Exclude on population: not cirrhosis |
| Zhao, Q., Liu, Y. Y., Lei, X., Lei, F. F., Li, G., Li, F., et al. PIVKA-II as a screening marker for hepatocellular carcinoma in patients with hepatitis B virus infection. World Chinese Journal of Digestology 2017;25(9):803-09. | Foreign language |
| Zhao, Qi, Sun, Xiangfei, Liu, Chao, Li, Tao, Cui, Juan. Expression of the microRNA-143/145 cluster is decreased in hepatitis B virus-associated hepatocellular carcinoma and may serve as a biomarker for tumorigenesis in patients with chronic hepatitis B. Oncology letters 2018;15(5):6115-22. | Exclude on population: not cirrhosis |
| Zhao, Qi, Xu, Yiqiu, Yuan, Dandan, Yang, Junjun, Wang, Ying, Shen, Guorong. Role of ssDNA as a Noninvasive Indicator for the Diagnosis and Prognosis of Hepatocellular Carcinoma: An Exploratory Study. Disease markers 2021;2021():9958909. | Exclude on population: healthy controls |
| Zhao, Xiu-Ying, Li, Ning, Ding, Hui-Guo. [Detection and evaluation of serum GP73, a resident Golgi glycoprotein, as a marker in diagnosis of hepatocellular carcinoma]. Zhonghua zhong liu za zhi [Chinese journal of oncology] 2010;32(12):943-45. | Foreign language |
| Zhao, Y., Li, Y., Hu, B., He, X., Huang, J., Zhan, M. Clinical application of serum Golgi protein 73 in the diagnosis and progress of hepatocellular carcinoma. National Medical Journal of China 2014;94(5):390-92. | Foreign language |
| Zhao, Y., Zhang, L., Li, H., Cui, C. Clinical significance of serum GP73, AFP, and CA199 test in the diagnosis of hepatic cancer. Chinese Journal of Clinical Oncology 2013;40(1):29. | Foreign language |
| Zhao, Y., Zhao, L., Jin, H., Xie, Y., Chen, L., Zhang, W., et al. Plasma methylated GNB4 and Riplet as a novel dual-marker panel for the detection of hepatocellular carcinoma. Epigenetics 2024;19(1):2299044. | Does not meet full eligibility criteria: includes people without cirrhosis |
| Zhao, Yangxing, Xue, Feng, Sun, Jinfeng, Guo, Shicheng, Zhang, Hongyu, Qiu, Bijun, et al. Genome-wide methylation profiling of the different stages of hepatitis B virus-related hepatocellular carcinoma development in plasma cell- | Exclude on population: healthy controls |

| Reference | Reason for exclusion |
|---|---|
| free DNA reveals potential biomarkers for early detection and high-risk monitoring of hepatocellular carcinoma. Clinical epigenetics 2014;6(1):30. | |
| Zhao, Yi-Ming, Wang, Lu, Dai, Zhi, Wang, Dan-Dan, Hei, Zhen-Yu, Zhang, Ning, et al. Validity of plasma macrophage migration inhibitory factor for diagnosis and prognosis of hepatocellular carcinoma. International journal of cancer 2011;129(10):2463-72. | Does not meet full eligibility criteria: includes people without cirrhosis |
| Zhao, Yinghua, Li, Yang, Liu, Wei, Xing, Shan, Wang, Dan, Chen, Jing, et al. Identification of noninvasive diagnostic biomarkers for hepatocellular carcinoma by urinary proteomics. Journal of proteomics 2020;225():103780. | No 2x2 data |
| Zhao, Yuanyuan, Yang, Bo, Chen, Dong, Zhou, Xiaojun, Wang, Meixi, Jiang, Jipin, Wei, Lai. Combined identification of ARID1A, CSMD1, and SENP3 as effective prognostic biomarkers for hepatocellular carcinoma. Aging 2021;13(3):4696-7112. | Exclude on population: clinical signs and symptoms of HCC |
| Zheng Y., Yang T., Soh A. Application of PIVKA-II in hepatocellular carcinoma diagnosis in Chinese population: Preliminary data from Asian multicenter study. Tumor Biology 2017;39(12):4-5. | CONFERENCE ABSTRACT |
| Zheng, Jie, Zhu, Ming-Yu, Wu, Fei, Kang, Bin, Liang, Ji, Heskia, Fabienne, Shan, Yun-Feng. A blood-based 22-gene expression signature for hepatocellular carcinoma identification. Annals of translational medicine 2020;8(5):195. | Does not meet full eligibility criteria: includes people without cirrhosis |
| Zheng, Kepu, Dai, Leiyang, Zhao, Yingpeng, Li, Laibang, Li, Wang, Zhang, Xibing, et al. Methylated SEPT9 combined with AFP and PIVKA-II is effective for the detection of HCC in high-risk population. BMC gastroenterology 2023;23(1):260. | Does not meet full eligibility criteria: includes people without cirrhosis |
| Zheng, R. Q., Huang, D. M., Zhang, B., Su, Z. Z., Kudo, M. Comparison of ultrasonography, CT and MRI in the diagnosis of liver cirrhosis. World Chinese Journal of Digestology 2005;13(8):993-96. | Foreign language |
| Zheng, Rencheng, Wang, Luna, Wang, Chengyan, Yu, Xuchen, Chen, Weibo, Li, Yan, et al. Feasibility of automatic detection of small hepatocellular carcinoma (<=2 cm) in cirrhotic liver based on pattern matching and deep learning. Physics in medicine and biology 2021;66(8):n. pag.. | Duplicate (including Cochrane includes) |
| Zheng, S. X., Xiang, B. D., Long, J. M., Qu, C., Mo, Z. J., Li, K., et al. Diagnostic value of serum SMP30 and anti-SMP30 antibody in hepatocellular carcinoma. Lab Medicine 2018;49(3):203-10. | Does not meet full eligibility criteria: includes people without cirrhosis |
| Zheng, T., Chen, M., Han, S., Zhang, L., Bai, Y., Fang, X., Ding, S. Z. Plasma minichromosome maintenance complex component 6 is a novel biomarker for hepatocellular carcinoma patients. Hepatology Research 2014;44(13):1347-56. | Does not meet full eligibility criteria: includes people without cirrhosis |
| Zheng, Tenghao, Chen, Ming, Han, Shuangyin, Zhang, Lida, Bai, Yangqiu, Fang, Xinhui, Ding, Song-Ze. Plasma minichromosome maintenance complex component 6 is a novel biomarker for hepatocellular carcinoma patients. Hepatology research : the official journal of the Japan Society of Hepatology 2014;44(13):1347-56. | Does not meet full eligibility criteria: includes people without cirrhosis |
| Zheng, Wenjie, Yang, Junling, Dong, Zhizhen, Wang, Li, Fang, Miao, Wu, Wei, Yao, Dengfu. High mobility group box 3 as an emerging biomarker in diagnosis and prognosis of hepatocellular carcinoma. Cancer management and research 2018;10():5979-89. | Does not meet full eligibility criteria: includes people without cirrhosis |
| Zheng, Wenjie, Yao, Min, Fang, Miao, Pan, Liuhong, Wang, Li, Yang, Junling, Dong, Zhizhen. Oncogenic Wnt3a: A Candidate Specific Marker and Novel Molecular Target for Hepatocellular Carcinoma. Journal of Cancer 2019;10(23):5862-73. | Exclude on population: not cirrhosis |
| Zheng, Wenjie, Yao, Min, Sai, Wenli, Qian, Qi, Pan, Liuhong, Qiu, Liwei, et al. Diagnostic and prognostic significance of secretory clusterin expression in patients with hepatocellular carcinoma. Tumour biology : the journal of the International Society for Oncodevelopmental Biology and Medicine 2016;37(1):999-1008. | Exclude on population: not cirrhosis |
| Zheng, Zhi-Kun, Pang, Cui, Yang, Yang, Duan, Qiong, Zhang, Ju. Serum long noncoding RNA urothelial carcinoma-associated 1: A novel biomarker for diagnosis and prognosis of hepatocellular carcinoma. The Journal of international medical research 2018;46(1):348-56. | Exclude on population: not cirrhosis |

| Reference | Reason for exclusion |
|---|---|
| Zhou Z., Xia D., Wang C., Lin C., Zhao W. Clinical evaluation of single or joint of golgi protein 73 and alpha-fetoprotein in hepatocellular carcinoma diagnosing. Chinese-German Journal of Clinical Oncology 2012;11(11):650-54. | Does not meet full eligibility criteria: includes people without cirrhosis |
| Zhou, C. F. . World Chinese Journal of Digestology 2017;25(35):3161-66. | Foreign language |
| Zhou, Guanlin, Zeng, Yijun, Luo, Yingmin, Guo, Sheng, Bao, Longyuan. Urine miR-93-5p is a promising biomarker for early detection of HBV-related hepatocellular carcinoma. European journal of surgical oncology : the journal of the European Society of Surgical Oncology and the British Association of Surgical Oncology 2022;48(1):95-102. | Exclude on population: not cirrhosis |
| Zhou, Hang, Zhang, Chao, Du, Linyao, Jiang, Jiapeng, Zhao, Qing, Sun, Jiawei, et al. Contrast-Enhanced Ultrasound Liver Imaging Reporting and Data System in Diagnosing Hepatocellular Carcinoma: Diagnostic Performance and Interobserver Agreement. Berichts- und Datensystem fur kontrastverstarkten Ultraschall in der Leberbildgebung (CEUS-LI-RADS) fur die Diagnose des hepatozellularen Karzinoms: Diagnostische Leistung und Interobserver-Ubereinstimmung. 2022;43(1):64-71. | Exclude on population: clinical signs and symptoms of HCC |
| Zhou, J. M., Han, F. L., Zhang, H. L., Sun, Y., Li, Z. H., Wang, T. Aptamer-Based Triple Serum Fluorescence Intensity Assay: A Novel and Feasible Method for the Clinical Diagnosis of Primary Hepatic Carcinoma. Frontiers in Oncology 2022;12():897775. | Does not meet full eligibility criteria: includes people without cirrhosis |
| Zhou, Jian, Yu, Lei, Gao, Xue, Hu, Jie, Wang, Jiping, Dai, Zhi, et al. Plasma microRNA panel to diagnose hepatitis B virus-related hepatocellular carcinoma. Journal of clinical oncology : official journal of the American Society of Clinical Oncology 2011;29(36):4781-88. | Does not meet full eligibility criteria: includes people without cirrhosis |
| Zhou, Jian-Wei, Li, Yuan, Yue, Li-Xia, Luo, Cheng-Lin, Chen, Yao. Autoantibody response to Sui1 and its tissue-specific expression in hepatocellular carcinoma. Tumour biology : the journal of the International Society for Oncodevelopmental Biology and Medicine 2016;37(2):2547-53. | No 2x2 data |
| Zhou, Jing. Diagnostic Value of OPNI in Hepatocellular Carcinoma. Oncology 2023;101(8):481-90. | Exclude on population: not cirrhosis |
| Zhou, Jiyuan, Yang, Wanna, Zhang, Shu, He, Xuanqiu, Lin, Jiatian, Zhou, Tao, et al. Diagnostic value of angiopoietin-like protein 2 for CHB-related hepatocellular carcinoma. Cancer management and research 2019;11():7159-69. | Duplicate (including Cochrane includes) |
| Zhou, Jiyuan, Yang, Wanna, Zhang, Shu, He, Xuanqiu, Lin, Jiatian, Zhou, Tao, et al. Diagnostic value of angiopoietin-like protein 2 for CHB-related hepatocellular carcinoma. Cancer management and research 2019;11(101512700):7159-69. | Exclude on population: not cirrhosis |
| Zhou, Junwei & Lai, Paul Bo-San. Identification of a non-coding KLF4 transcript generated from intron retention and downregulated in human hepatocellular carcinoma. International journal of oncology 2015;47(4):1554-62. | Exclude on population: not cirrhosis |
| Zhou, Q. & Zhang, Q. Differential diagnosis of primary hepatic carcinoma by detecting serum alpha-fetoprotein, tumor-associated substances and carcinoembryonic antigen. World Chinese Journal of Digestology 2010;18(18):1936-39. | Foreign language |
| Zhou, W., Guo, X., Cao, Z. G., Zhao, X. T. Detection of glypican 3 in serum and its clinical significance on the diagnosis of hepatocellular carcinoma. Tumor 2007;27(9):679-82. | Foreign language |
| Zhou, X. L., Mi, Y. Q., Xu, L., Liu, Y. G., Zhong, Y. Clinical value of protein induced by vitamin K absence or antagonist-II in diagnosis of hepatocellular carcinoma. World Chinese Journal of Digestology 2018;26(7):425-33. | Foreign language |
| Zhou, Z., Xia, D., Wang, C., Lin, C., Zhao, W. Clinical evaluation of single or joint of golgi protein 73 and alpha-fetoprotein in hepatocellular carcinoma diagnosing. Chinese-German Journal of Clinical Oncology 2012;11(11):650-54. | Duplicate (including Cochrane includes) |
| Zhu, H. T., Liu, R. B., Liang, Y. Y., Hasan, A. M. E., Wang, H. Y., Shao, Q., et al. Serum microRNA profiles as diagnostic biomarkers for HBV-positive hepatocellular carcinoma. Liver International 2017;37(6):888-96. | Does not meet full eligibility criteria: includes people without cirrhosis |

| Reference | Reason for exclusion |
|---|---|
| Zhu, Hao-Tu, Liu, Rong-Bin, Liang, Ya-Yong, Hasan, Abdulbaqi M. E., Wang, Hai-Yun, Shao, Qiong, et al. Serum microRNA profiles as diagnostic biomarkers for HBV-positive hepatocellular carcinoma. Liver international : official journal of the International Association for the Study of the Liver 2017;37(6):888-96. | Does not meet full eligibility criteria: includes people without cirrhosis |
| Zhu, Jianhui, Lin, Zhenxin, Wu, Jing, Yin, Haidi, Dai, Jianliang, Feng, Ziding, Marrero, Jorge. Analysis of serum haptoglobin fucosylation in hepatocellular carcinoma and liver cirrhosis of different etiologies. Journal of proteome research 2014;13(6):2986-97. | No 2x2 data |
| Zhu, Jing, Jiang, Feng, Ni, Hong-Bing, Xiao, Ming-Bing, Chen, Bu-You, Ni, Wen-Kai, Lu, Cui-Hua. Combined analysis of serum gamma-glutamyl transferase isoenzyme II, alpha-L-fucosidase and alpha-fetoprotein detected using a commercial kit in the diagnosis of hepatocellular carcinoma. Experimental and therapeutic medicine 2013;5(1):89-94. | Does not meet full eligibility criteria: includes people without cirrhosis |
| Zhu, Kai, Zhan, Hao, Peng, Yuanfei, Yang, Liuxiao, Gao, Qiang, Jia, Hao, et al. Plasma hsa_circ_0027089 is a diagnostic biomarker for hepatitis B virus-related hepatocellular carcinoma. Carcinogenesis 2020;41(3):296-302. | Does not meet full eligibility criteria: includes people without cirrhosis |
| Zhu, Lihui, Li, Tao, Ma, Xiaomin, Qiu, Yumin, Ma, Xiaoxiao, Lin, Yueke, Han, Lihui. A Simple Noninvasive Index Can Predict Hepatocellular Carcinoma in Patients with Chronic Hepatitis B. Scientific reports 2017;7(1):8954. | Does not meet full eligibility criteria: includes people without cirrhosis |
| Zhu, Mingyu, Zheng, Jie, Wu, Fei, Kang, Bin, Liang, Ji, Heskia, Fabienne, Zhang, Xinxin. OPN is a promising serological biomarker for hepatocellular carcinoma diagnosis. Journal of medical virology 2020;():n. pag.. | Duplicate (including Cochrane includes) |
| Zhu, Mingyu, Zheng, Jie, Wu, Fei, Kang, Bin, Liang, Ji, Heskia, Fabienne, Zhang, Xinxin. OPN is a promising serological biomarker for hepatocellular carcinoma diagnosis. Journal of medical virology 2020;(i9n, 7705876):n. pag.. | Does not meet full eligibility criteria: includes people without cirrhosis |
| Zhu, Qianyu, Zhou, Jialing, Li, Hongyi, Wang, Hui, Ren, Jiangbo, Sun, Shujie, et al. Dynamic changes in three biomarkers predict early-stage hepatocellular carcinoma in patients with chronic hepatitis B receiving antiviral therapy. Journal of cancer research and clinical oncology 2023;149(14):12691â€“12701. | Does not meet full eligibility criteria: includes people without cirrhosis |
| Zhu, Rongping, Xiao, Juan, Luo, Diteng, Dong, Mingjun, Sun, Tian. Serum AKR1B10 predicts the risk of hepatocellular carcinoma - A retrospective single-center study. Gastroenterologia y hepatologia 2019;42(10):614-21. | Exclude on population: healthy controls |
| Zhu, S., Liu, W., Fu, B., Yuan, Z., Zhou, Y., Li, H. Association of serum mir-205 with liver cirrhosis and cancer and its diagnostic significance. International Journal of Clinical and Experimental Medicine 2018;11(11):12375-80. | Exclude on population: healthy controls |
| Zhu, Wentao, Shi, Pei, Liang, An, Zhu, Ying, Fu, Jiwei, Yuan, Songsong. The combination of serum oligosaccharide chain (G-test), alpha-fetoprotein, and aspartate aminotransferase to alanine aminotransferase ratio provides the optimal diagnostic value for early detection of hepatocellular carcinoma. BMC cancer 2022;22(1):1061. | Does not meet full eligibility criteria: includes people without cirrhosis |
| Zhu, Wen-Wei, Guo, Jia-Jian, Guo, Lei, Jia, Hu-Liang, Zhu, Ming, Zhang, Ju-Bo, et al. Evaluation of midkine as a diagnostic serum biomarker in hepatocellular carcinoma. Clinical cancer research : an official journal of the American Association for Cancer Research 2013;19(14):3944-54. | Does not meet full eligibility criteria: includes people without cirrhosis |
| Zhu, Wen-Wei, Guo, Jia-Jian, Guo, Lei, Jia, Hu-Liang, Zhu, Ming, Zhang, Ju-Bo, et al. Evaluation of midkine as a diagnostic serum biomarker in hepatocellular carcinoma. Clinical cancer research : an official journal of the American Association for Cancer Research 2013;19(14):3944-54. | Duplicate (including Cochrane includes) |
| Zhu, X., Feng, Y., He, D., Wang, Z., Huang, F. Clinical Value and Underlying Mechanisms of Upregulated LINC00485 in Hepatocellular Carcinoma. Frontiers in Oncology 2021;11():654424. | Exclude on population: not cirrhosis |
| Zhuang, Chunbo, Jiang, Weichao, Huang, Da, Xu, Luming, Yang, Qianqian, Zheng, Lei, Wang, Xiaobei. Serum miR-21, miR-26a and miR-101 as potential biomarkers of hepatocellular carcinoma. Clinics and research in hepatology and gastroenterology 2016;40(4):386-96. | Exclude on population: not cirrhosis |
| Zhuang, H. & Wang, B. Integrative screening of DNA methylation markers enables early detection of hepatocellular carcinoma. Cancer Research 2025;85(8 Supplement 1):n. pag.. | CONFERENCE ABSTRACT |

| Reference | Reason for exclusion |
|---|---|
| Ziada, Dina H, El Sadany, Sherif, Soliman, Hanan, Abd-Elsalam, Sherief, Salama, Marwa, Hawash, Nehad, et al. Prevalence of hepatocellular carcinoma in chronic hepatitis C patients in Mid Delta, Egypt: A single center study. Journal of the Egyptian National Cancer Institute 2016;28(4):257-62. | Exclude on population: not cirrhosis |
| Zinkin, Noah T., Grall, Franck, Bhaskar, Killimangalam, Otu, Hasan H., Spentzos, Dimitrios, Kalmowitz, Brett, et al. Serum proteomics and biomarkers in hepatocellular carcinoma and chronic liver disease. Clinical cancer research : an official journal of the American Association for Cancer Research 2008;14(2):470-77. | Duplicate (including Cochrane includes) |
| Zoli, M., Magalotti, D., Bianchi, G., Gueli, C., Marchesini, G. Efficacy of a surveillance program for early detection of hepatocellular carcinoma. Cancer 1996;78(5):977-85. | No 2x2 data |
| Zou, L., Hao, D., Cai, G. [Observation of the serum acidic isoferritin levels in patients with hepatocellular carcinoma]. Hua xi yi ke da xue xue bao = Journal of West China University of Medical Sciences = Huaxi yike daxue xuebao 1997;28(4):424-27. | Does not meet full eligibility criteria: pre-2005 |
| Zou, Wenshuang, Cui, Junfeng, Ren, Zhong. NRAGE is a potential diagnostic biomarker of hepatocellular carcinoma. Medicine 2018;97(48):e13411. | Exclude on population: not cirrhosis |
| Zou, Zhi-Qiang, Ding, Yu-Ping, Long, Bo, Yuh, Ji-Guang, Xu, Ai-Ling, Lang, Zhen-Wei, et al. Gpc-3 is a notable diagnostic, prognostic and a latent targeted therapy marker in hepatocellular carcinoma. Hepato-gastroenterology 2010;57(102-103):1285-90. | Exclude on study design: not a 1- or 2-gate test accuracy study |
| Zuo, Duo, An, Haohua, Li, Jianhua, Xiao, Jiawei. The Application Value of Lipoprotein Particle Numbers in the Diagnosis of HBV-Related Hepatocellular Carcinoma with BCLC Stage 0-A. Journal of personalized medicine 2021;11(11):n. pag.. | Exclude on population: not cirrhosis |
| Zuo, Duo, Chen, Liwei, Liu, Xiaoqian, Wang, Xia, Xi, Qing, Luo, Yi, Zhang, Ning. Combination of miR-125b and miR-27a enhances sensitivity and specificity of AFP-based diagnosis of hepatocellular carcinoma. Tumour biology : the journal of the International Society for Oncodevelopmental Biology and Medicine 2016;37(5):6539-49. | Duplicate (including Cochrane includes) |
| Zuo, Duo, Chen, Liwei, Liu, Xiaoqian, Wang, Xia, Xi, Qing, Luo, Yi, Zhang, Ning. Combination of miR-125b and miR-27a enhances sensitivity and specificity of AFP-based diagnosis of hepatocellular carcinoma. Tumour biology : the journal of the International Society for Oncodevelopmental Biology and Medicine 2016;37(5):6539-49. | Exclude on population: not cirrhosis |
| Zhuang, H. & Wang, B. Integrative screening of DNA methylation markers enables early detection of hepatocellular carcinoma. Cancer Research 2025;85(8 Supplement 1):n. pag.. | CONFERENCE ABSTRACT |
| Ziada, Dina H, El Sadany, Sherif, Soliman, Hanan, Abd-Elsalam, Sherief, Salama, Marwa, Hawash, Nehad, et al. Prevalence of hepatocellular carcinoma in chronic hepatitis C patients in Mid Delta, Egypt: A single center study. Journal of the Egyptian National Cancer Institute 2016;28(4):257-62. | Exclude on population: not cirrhosis |
| Zinkin, Noah T., Grall, Franck, Bhaskar, Killimangalam, Otu, Hasan H., Spentzos, Dimitrios, Kalmowitz, Brett, et al. Serum proteomics and biomarkers in hepatocellular carcinoma and chronic liver disease. Clinical cancer research : an official journal of the American Association for Cancer Research 2008;14(2):470-77. | Duplicate (including Cochrane includes) |
| Zoli, M., Magalotti, D., Bianchi, G., Gueli, C., Marchesini, G. Efficacy of a surveillance program for early detection of hepatocellular carcinoma. Cancer 1996;78(5):977-85. | No 2x2 data |
| Zou, L., Hao, D., Cai, G. [Observation of the serum acidic isoferritin levels in patients with hepatocellular carcinoma]. Hua xi yi ke da xue xue bao = Journal of West China University of Medical Sciences = Huaxi yike daxue xuebao 1997;28(4):424-27. | Does not meet full eligibility criteria: pre-2005 |
| Zou, Wenshuang, Cui, Junfeng, Ren, Zhong. NRAGE is a potential diagnostic biomarker of hepatocellular carcinoma. Medicine 2018;97(48):e13411. | Exclude on population: not cirrhosis |
| Zou, Zhi-Qiang, Ding, Yu-Ping, Long, Bo, Yuh, Ji-Guang, Xu, Ai-Ling, Lang, Zhen-Wei, et al. Gpc-3 is a notable diagnostic, prognostic and a latent targeted | Exclude on study design: not a 1- or 2-gate test accuracy study |

| Reference | Reason for exclusion |
|---|---|
| therapy marker in hepatocellular carcinoma. Hepato-gastroenterology 2010;57(102-103):1285-90. | |
| Zuo, Duo, An, Haohua, Li, Jianhua, Xiao, Jiawei. The Application Value of Lipoprotein Particle Numbers in the Diagnosis of HBV-Related Hepatocellular Carcinoma with BCLC Stage 0-A. Journal of personalized medicine 2021;11(11):n. pag.. | Exclude on population: not cirrhosis |
| Zuo, Duo, Chen, Liwei, Liu, Xiaoqian, Wang, Xia, Xi, Qing, Luo, Yi, Zhang, Ning. Combination of miR-125b and miR-27a enhances sensitivity and specificity of AFP-based diagnosis of hepatocellular carcinoma. Tumour biology : the journal of the International Society for Oncodevelopmental Biology and Medicine 2016;37(5):6539-49. | Duplicate (including Cochrane includes) |
| Zuo, Duo, Chen, Liwei, Liu, Xiaoqian, Wang, Xia, Xi, Qing, Luo, Yi, Zhang, Ning. Combination of miR-125b and miR-27a enhances sensitivity and specificity of AFP-based diagnosis of hepatocellular carcinoma. Tumour biology : the journal of the International Society for Oncodevelopmental Biology and Medicine 2016;37(5):6539-49. | Exclude on population: not cirrhosis |

## Appendix S8 Characteristics of included studies

| Study | Study details | Study population | Index test and reference standard |
|---|---|---|---|
| Abdel Rehem & El-Shikh (2011)[1] | **Study design:** 2-gate<br>**Method of recruitment:** Clinical cohort<br>**Setting:** Egypt | **Participants, n:** 80 (HCC: 20; No HCC: 60)<br>**Age:** No HCC: Mean 50 (SD 9)<br>HCC: Mean 58 (SD 10)<br>**Aetiology:** HCC: HCV 20 (100.0%)<br>No HCC: HCV 60 (100.0%)<br>**Cirrhosis severity:** No HCC: Child's grade A 20 (33.3%) | **Index test(s):** AFP<br>**Reference standard to establish presence of HCC:** Blood tests and imaging (CT with or without AFP)<br>**Reference standard to establish absence of HCC:** Imaging (All patients had US.) |
| Abdel-Azeez et al. (2022)[2] | **Study design:** 2-gate<br>**Method of recruitment:** Clinical cohort<br>**Setting:** Egypt | **Participants, n:** 48 (HCC: 24; No HCC: 24)<br>**Age:** HCC: Mean 60.8 (SD 7.4)<br>No HCC: Mean 57 (SD 5.8)<br>**Aetiology:** HCC: HCV 22 (91.7%); HBV 2 (8.3%)<br>No HCC: HBV 1 (4.2%); HCV 24 (100.0%)<br>**Cirrhosis severity:** HCC: Child's grade A 0 (0.0%)<br>No HCC: Child's grade A 1 (4.2%) | **Index test(s):** AFP<br>**Reference standard to establish presence of HCC:** Histology/imaging (AFP and imaging (US, CT or MRI) or biopsy.)<br>**Reference standard to establish absence of HCC:** Imaging (All patients had radiological imaging.) |
| Abdelfattah et al. (2019)[3] | **Study design:** 2-gate<br>**Method of recruitment:** Clinical cohort<br>**Setting:** Egypt | **Participants, n:** 76 (HCC: 44; No HCC: 32)<br>**Age:** HCC: Mean 54.9 (SD 5.8)<br>No HCC: Mean 54.5 (SD 6.9)<br>**Aetiology:** HCV 76 (100.0%)<br>**Cirrhosis severity:** HCC: Child's grade A 18 (40.9%)<br>No HCC: Child's grade A 16 (50.0%) | **Index test(s):** AFP<br>**Reference standard to establish presence of HCC:** Imaging (All patients had US and CT.)<br>**Reference standard to establish absence of HCC:** Imaging (All patients had US and CT.) |

[1] Abdel Rehem, R. N. A. M. Serum IGF-1, IGF-2 and IGFBP-3 as parameters in the assessment of liver dysfunction in patients with hepatic cirrhosis and in the diagnosis of hepatocellular carcinoma. Hepato-Gastroenterology 2011;58(107-108):949-54.

[2] Abdel-Azeez, Hala A. & Elhady, Hoda A. Cartilage oligomeric matrix protein as a non-invasive biomarker for diagnosis of hepatocellular carcinoma in patients with liver cirrhosis. Gastroenterology and hepatology from bed to bench 2022;15(2):139-45.

[3] Abdelfattah, Shaymaa Nafady, Haseeb, Alaa Farouk, Tawfik, Mohammad Mohammad, Khalil, Doaa Mahmoud. Soluble CD25 as a predictor of hepatocellular carcinoma compared with alpha-fetoprotein. Clinical and experimental hepatology 2019;5(2):140-46.

| Study | Study details | Study population | Index test and reference standard |
|---|---|---|---|
| Abdel-Razik et al. (2016)[4] | **Study design:** 2-gate<br>**Method of recruitment:** Clinical cohort<br>**Setting:** Egypt | **Participants, n:** 162 (HCC: 82; No HCC: 80)<br>**Age:** HCC: (range 38, 70)<br>No HCC: (range 35, 62)<br>**Aetiology:** HCC: HCV 80 (100.0%)<br>No HCC: HCV 80 (100.0%) | **Index test(s):** AFP; DCP (ng/ml)<br>**Reference standard to establish presence of HCC:** Imaging ('All studied patients were subjected to a full assessment of history, clinical examination, abdominal ultrasonography, and computed tomography scan to confirm and/or exclude the presence of small HCC.')<br>**Reference standard to establish absence of HCC:** Imaging (null) |
| Abdelwahab et al. (2017)[5] | **Study design:** 2-gate<br>**Method of recruitment:** Clinical cohort<br>**Setting:** Egypt | **Participants, n:** 60 (HCC: 30; No HCC: 30) | **Index test(s):** AFP<br>**Reference standard to establish presence of HCC:** Imaging (Lesion and AFP level >400 ng mL-1 or space occupying lesion with arterial phase enhancement and rapid washout in portovenous phase by CT.)<br>**Reference standard to establish absence of HCC:** Imaging (All participants had US.) |
| Alrefai et al. (2024)[6] | **Study design:** 2-gate<br>**Method of recruitment:** Clinical cohort<br>**Setting:** Egypt | **Participants, n:** 100 (HCC: 50; No HCC: 50)<br>**Age:** HCC: Mean 59.7 (SD 6.2)<br>No HCC: Mean 57.7 (SD 4.8)<br>**Aetiology:** HCC: HCV 50 (100.0%)<br>No HCC: HCV 50 (100.0%)<br>**Cirrhosis severity:** HCC: Child's grade A 31 (62.0%)<br>No HCC: Child's grade A 35 (70.0%) | **Index test(s):** AFP; miR-331-3p; miR-23b-3p; miR-3194-5p; miR-3194-5p<br>**Reference standard to establish presence of HCC:** Imaging<br>**Reference standard to establish absence of HCC:** Not reported |

[4] Abdel-Razik, Ahmed, Elhelaly, Rania, Elzehery, Rasha, El-Diasty, Amany, Abed, Sally, Elhammady, Dina. Could serotonin be a potential marker for hepatocellular carcinoma? A prospective single-center observational study. European journal of gastroenterology & hepatology 2016;28(5):599-605.

[5] Abdelwahab, M. A., Elbedewy, M. M., Amin, A. M. Serum endoglin and IL-6 levels as complementary diagnostic biomarkers for hepatocellular carcinoma in Egyptian liver cirrhosis patients. Research Journal of Immunology 2017;10(1):1-7.

[6] Alrefai, A. A., Rizk, S. K., Khamis, A. K., Kasemy, Z. A. Utility of a microRNA panel in diagnosis and prognosis of hepatitis C-Associated hepatocellular carcinoma. Lab Medicine 2024;55(3):310 " 319.

| Study | Study details | Study population | Index test and reference standard |
|---|---|---|---|
| Aly et al. (2020)[7] | **Study design:** 2-gate<br>**Method of recruitment:** Clinical cohort<br>**Setting:** Egypt | **Participants, n:** 60 (HCC: 40; No HCC: 20)<br>**Age:** HCC: Mean 59.3 (SD 5.2)<br>**Aetiology:** HCV 60 (100.0%)<br>**Cirrhosis severity:** HCC: Child's grade A 8 (20.0%)<br>No HCC: Child's grade A 3 (15.0%) | **Index test(s):** AFP; Let-7a-1; AFP or Let-7a-1<br>**Reference standard to establish presence of HCC:** Imaging (AFP and CT according to EASL guidelines.)<br>**Reference standard to establish absence of HCC:** Not reported |
| Amr et al. (2016)[8] | **Study design:** 1-gate<br>**Method of recruitment:** Clinical cohort<br>**Setting:** Egypt | **Participants, n:** 40 (HCC: 23; No HCC: 17)<br>**Age:** HCC: Mean 54.3 (SD 9.2)<br>No HCC: Mean 45.2 (SD 9.4)<br>**Aetiology:** HCC: HCV 20 (87.0%); HBV 4 (17.4%)<br>No HCC: HCV 10 (58.8%); HBV 5 (29.4%) | **Index test(s):** mRNA-21; mRNA-199-a; AFP<br>**Reference standard:** Histology |
| Arrieta et al. (2007)[9] | **Study design:** 2-gate<br>**Method of recruitment:** Clinical cohort<br>**Setting:** Mexico | **Participants, n:** 267 (HCC: 193; No HCC: 74)<br>**Age:** HCC: Mean 59.9 (SD 1)<br>No HCC: Mean 51.7 (SD 1.7)<br>**Aetiology:** HCC: Alcohol 50 (25.9%); HBV 14 (7.3%); HCV 58 (30.1%)<br>No HCC: HCV 33 (44.6%); Alcohol 8 (10.8%); HBV 0 (0.0%)<br>**Cirrhosis severity:** HCC: Child's grade A 91 (47.2%)<br>No HCC: Child's grade A 30 (40.5%) | **Index test(s):** AFP; AFP progression rate (ng/ml per month)<br>**Reference standard to establish presence of HCC:** Histology (Histology of biopsied lesions.)<br>**Reference standard to establish absence of HCC:** Imaging (with 6-months imaging follow-up) ('Minimal follow-up of one year with monthly AFP tests and imaging studies [US, CT and MRI] every 3 to 6 months in order to assess they did not have HCC'.) |

[7] Aly, Doaa Mamdouh, Gohar, Nadida Abdul-Hameed, Abd El-Hady, Afaf Ahmed, Khairy, Marwa. Serum microRNA let-7a-1/let-7d/let-7f and miRNA 143/145 Gene Expression Profiles as Potential Biomarkers in HCV Induced Hepatocellular Carcinoma. Asian Pacific journal of cancer prevention : APJCP 2020;21(2):555-62.

[8] Amr, Khalda Said, Ezzat, Wafaa M., Elhosary, Yasser A., Hegazy, Abdelfattah E., Fahim, Hoda H. The potential role of miRNAs 21 and 199-a in early diagnosis of hepatocellular carcinoma. Gene 2016;575(1):66-70.

[9] Arrieta, Oscar, Cacho, Bernardo, Morales-Espinosa, Daniela, Ruelas-Villavicencio, Ana, Flores-Estrada, Diana. The progressive elevation of alpha fetoprotein for the diagnosis of hepatocellular carcinoma in patients with liver cirrhosis. BMC cancer 2007;7(100967800):28.

| Study | Study details | Study population | Index test and reference standard |
|---|---|---|---|
| Atiq et al. (2017)[10] | **Study design:** 1-gate<br>**Method of recruitment:** Surveillance programme<br>**Setting:** USA | **Participants, n:** 78 (HCC: 78)<br>**Age:** Mean 54.3 (SD 9.4)<br>**Aetiology:** HBV 22 (3.2%); HCV 382 (56.2%); Alcohol 175 (25.7%); NAFLD/NASH 79 (11.6%)<br>**Cirrhosis severity:** Child's grade A 203 (29.9%) | **Index test(s):** B-mode US; AFP<br>**Reference standard:** Unclear (AFP and US surveillance. Further reference standard tests only carried out when AFP or US were positive – unclear what tests consisted of.) |
| Attallah et al. (2013)[11] | **Study design:** 2-gate<br>**Method of recruitment:** Clinical cohort<br>**Setting:** Egypt | **Participants, n:** 982 (HCC: 537; No HCC: 445)<br>**Age:** HCC: Mean 59.9 (SD 9.8)<br>No HCC: Mean 65 (SD 3.8)<br>No HCC: Mean 49.3 (SD 9.3)<br>**Aetiology:** HCV 982 (100.0%)<br>**Cirrhosis severity:** No HCC: Child's grade A 420 (100.0%); Child's grade A 420 (94.4%) | **Index test(s):** AFP; HCC-ART<br>**Reference standard to establish presence of HCC:** Histology/imaging (US, confirmed by CT and/or MRI. 'The final diagnosis was confirmed by histopathologic analysis on US-assisted fine-needle biopsy, when indicated.')<br>**Reference standard to establish absence of HCC:** Not reported (null) |
| Bai et al. (2023)[12] | **Study design:** 2-gate<br>**Method of recruitment:** Clinical cohort<br>**Setting:** China | **Participants, n:** 138 (HCC: 98; No HCC: 40)<br>**Age:** HCC: Median 57 (range 51, 64)<br>No HCC: Median 51 (range 43, 57)<br>**Aetiology:** HCC: HBV 88 (89.8%); Alcohol 3 (3.1%); Other / unknown 5 (5.1%); NAFLD/NASH 2 (2.0%)<br>No HCC: HBV 35 (87.5%); Alcohol 2 (5.0%); NAFLD/NASH 0 (0.0%); Other / unknown 3 (7.5%) | **Index test(s):** HepaClear<br>**Reference standard to establish presence of HCC:** Unclear<br>**Reference standard to establish absence of HCC:** Not reported |
| Baird et al. (2013)[13] | **Study design:** 1-gate<br>**Method of recruitment:** Surveillance programme<br>**Setting:** Australia | **Participants, n:** 15 (HCC: 15)<br>**Age:** Mean 51 (SD 11)<br>**Aetiology:** NAFLD/NASH 2 (6.7%); Alcohol 2 (6.7%); HCV 20 (66.7%); HBV 2 (6.7%) | **Index test(s):** CE-MRI; AFP<br>**Reference standard:** Explant pathology following transplantation |

[10] Atiq, Omair, Tiro, Jasmin, Yopp, Adam C, Muffler, Adam, Marrero, Jorge A, Parikh, Neehar D, et al. An assessment of benefits and harms of hepatocellular carcinoma surveillance in patients with cirrhosis. Hepatology (Baltimore, Md.) 2017;65(4):1196-2005.

[11] Attallah, A M, Omran, M M, Attallah, A A, Abdallah, S O, Farid, K, Darwish, H, El-Dosoky, I. HCC-ART score, a simple, highly sensitive and specific test for early diagnosis of hepatocellular carcinoma: a large-scale, multicentre study. British journal of cancer 2013;109(6):1657-65.

[12] Bai, Yi, Xu, Juan, Li, Deqiang, Zhang, Xiaoyu, Chen, Dapeng, Xie, Fucun, et al. HepaClear, a blood-based panel combining novel methylated CpG sites and protein markers, for the detection of early-stage hepatocellular carcinoma. Clinical epigenetics 2023;15(1):99.

[13] Baird, Andrew J., Amos, Gregory J., Saad, Nivene F. Retrospective audit to determine the diagnostic accuracy of Primovist-enhanced MRI in the detection of hepatocellular carcinoma in cirrhosis with explant histopathology correlation. Journal of medical imaging and radiation oncology 2013;57(3):314-20.

| Study | Study details | Study population | Index test and reference standard |
|---|---|---|---|
| Beale et al. (2008)[14] | **Study design:** 2-gate<br>**Method of recruitment:** Clinical cohort<br>**Setting:** UK | **Participants, n:** 91 (HCC: 50; No HCC: 41)<br>**Age:** HCC: Mean 67.5 (SD 12)<br>No HCC: Mean 54.3 (SD 9.6)<br>**Aetiology:** HCC: Alcohol 30 (60.0%); NAFLD/NASH 20 (40.0%)<br>No HCC: Alcohol 33 (80.5%); NAFLD/NASH 8 (19.5%)<br>**Cirrhosis severity:** HCC: Child's grade A 27 (54.0%)<br>No HCC: Child's grade A 22 (53.7%) | **Index test(s):** AFP; DCP (ng/ml); AFP or DCP (ng/ml)<br>**Reference standard to establish presence of HCC:** Imaging ('Patients were diagnosed as having HCC as per guidelines proposed by the EASL.')<br>**Reference standard to establish absence of HCC:** Not reported |
| Beudeker et al. (2023)[15] | **Study design:** 2-gate<br>**Method of recruitment:** Clinical cohort<br>**Setting:** Taiwan | **Participants, n:** 562 (HCC: 277; No HCC: 285)<br>**Age:** HCC: Median 66.5 (range 56, 73)<br>No HCC: Median 61 (range 49, 70)<br>**Aetiology:** HCC: HBV 31 (11.2%); Alcohol 72 (26.0%); Other / unknown 38 (13.7%); HCV 35 (12.6%); NAFLD/NASH 101 (36.5%)<br>No HCC: HCV 23 (8.1%); NAFLD/NASH 137 (48.1%); Alcohol 54 (18.9%); HBV 37 (13.0%); Other / unknown 34 (11.9%) | **Index test(s):** AFP; DCP; GALAD; AFP-L3; DCP (mAU/mL)<br>**Reference standard to establish presence of HCC:** Histology/imaging (either a diagnosis assigned by the managing hepatologists or evidence of hepatic steatosis by histopathology or ultrasound in the absence of alternative liver diseases.)<br>**Reference standard to establish absence of HCC:** Not reported |
| Bhatti et al. (2021)[16] | **Study design:** 1-gate<br>**Method of recruitment:** Clinical cohort<br>**Setting:** Pakistan | **Participants, n:** 244 (HCC: 176; No HCC: 68)<br>**Age:** HCC: >50: 114 (64.8%)<br>No HCC: >50: 32 (47.1%)<br>**Aetiology:** HCC: HCV 152 (86.4%); HBV 36 (20.5%)<br>No HCC: HBV 41 (60.3%); HCV 28 (41.2%)<br>**Cirrhosis severity:** HCC: Child's grade A 87 (49.4%)<br>No HCC: Child's grade A 16 (23.5%) | **Index test(s):** AFP; DCP; AFP or DCP<br>**Reference standard:** Imaging (CT or MRI) |

[14] Beale, Gary, Chattopadhyay, Dipankar, Gray, Joe, Stewart, Stephen, Hudson, Mark, Day, Christopher, et al. AFP, PIVKAII, GP3, SCCA-1 and follisatin as surveillance biomarkers for hepatocellular cancer in non-alcoholic and alcoholic fatty liver disease. BMC cancer 2008;8():200.

[15] Beudeker, Boris J. B., Fu, Siyu, Balderramo, Domingo, Mattos, Angelo Z., Carrera, Enrique, Diaz, Javier, et al. Validation and optimization of AFP-based biomarker panels for early HCC detection in Latin America and Europe. Hepatology communications 2023;7(10):n. pag..

[16] Bhatti, Abu Bakar Hafeez H., Naz, Kiran, Abbas, Ghazanfar, Khan, Nusrat Y., Zia, Haseeb H. Clinical Utility of Protein Induced by Vitamin K Absence-II in Patients with Hepatocellular Carcinoma. Asian Pacific journal of cancer prevention : APJCP 2021;22(6):1731-36.

| Study | Study details | Study population | Index test and reference standard |
|---|---|---|---|
| Biselli et al. (2015)[17] | **Study design:** 2-gate<br>**Method of recruitment:** Surveillance programme<br>**Setting:** Italy | **Participants, n:** 240 (HCC: 80; No HCC: 160)<br>**Age:** HCC: Median 66 (range 51, 81)<br>No HCC: Median 65 (range 48, 81)<br>**Aetiology:** HCC: HCV 55 (68.8%); HBV 14 (17.5%); Alcohol 9 (11.2%)<br>No HCC: HBV 28 (17.5%); HCV 110 (68.8%); Alcohol 18 (11.2%) | **Index test(s):** AFP; Serial AFP – any increase; Combined AFP index (serial AFP and AFP)<br>**Reference standard to establish presence of HCC:** Blood tests and imaging (US and AFP surveillance. Other imaging tests performed when tests were positive or US unclear.)<br>**Reference standard to establish absence of HCC:** Blood tests and imaging (US and AFP surveillance at least 18 months from enrollment.) |
| Brigida et al. (2020)[18] | **Study design:** 2-gate<br>**Method of recruitment:** Clinical cohort<br>**Setting:** Iraq | **Participants, n:** 60 (HCC: 30; No HCC: 30)<br>**Age:** HCC: Mean 58 (SD 5.1)<br>No HCC: Mean 56.9 (SD 5)<br>**Aetiology:** HCC: HBV 15 (50.0%); HCV 19 (63.3%)<br>No HCC: HCV 21 (70.0%); HBV 15 (50.0%) | **Index test(s):** AFP; AFP-L3; DCP (ng/ml); AFP or AFP-L3 or DCP (ng/ml); AFP or DCP (ng/ml); AFP-L3 or DCP (ng/ml)<br>**Reference standard to establish presence of HCC:** Histology/imaging (US and CT/MRI with histological confirmation.)<br>**Reference standard to establish absence of HCC:** Imaging (US scan before enrollment.) |
| Cai et al. (2024)[19] | **Study design:** 2-gate<br>**Method of recruitment:** Clinical cohort<br>**Setting:** China | **Participants, n:** 171 (HCC: 107; No HCC: 64)<br>**Age:** HCC: Median 57 (range 50, 64)<br>No HCC: Median 53 (range 47.25, 58.75)<br>**Aetiology:** HCC: HBV 107 (100.0%)<br>No HCC: HBV 64 (100.0%) | **Index test(s):** AFP<br>**Reference standard to establish presence of HCC:** Histology/imaging (All patients were confirmed by liver pathological examination, X-ray examination or MRI examination,)<br>**Reference standard to establish absence of HCC:** Imaging (All patients were confirmed by liver pathological examination, X-ray examination or MRI examination,) |
| Caviglia et al. (2017)[20] | **Study design:** 2-gate<br>**Method of recruitment:** Clinical cohort<br>**Setting:** Italy | **Participants, n:** 63 (HCC: 33; No HCC: 30)<br>**Age:** HCC: Median 63 (range 62, 64)<br>No HCC: Median 54 (range 50, 58)<br>**Aetiology:** HCC: HBV 33 (100.0%)<br>No HCC: HBV 30 (100.0%) | **Index test(s):** miR-122; AFP; DCP<br>**Reference standard to establish presence of HCC:** Imaging ('Diagnosis of HCC was established by CT scan.')<br>**Reference standard to establish absence of HCC:** Not reported (null) |

[17] Biselli, M, Conti, F, Gramenzi, A, Frigerio, M, Cucchetti, A, Fatti, G, et al. A new approach to the use of alpha-fetoprotein as surveillance test for hepatocellular carcinoma in patients with cirrhosis. British journal of cancer 2015;112(1):69-76.

[18] Brigida, K., Khikmatullaeva, A., Abdukadirova, M., Lokteva, L. The diagnostic value of AFP, AFP l3 and PIVKA ii in hepatocellular carcinoma outcome of cirrhosis of the liver HBV and HCV etiology. International Journal of Pharmaceutical Research 2020;12(2):1758-62.

[19] Cai, X., Peng, S., Xiao, X., Huang, Z. Serum ApoB/ApoA1 ratio in patients with CHB and the occurrence of HBV related cirrhosis and HBV related hepatocellular carcinoma. Scientific reports 2024;14(1):10996.

[20] Caviglia, Gian P, Abate, Maria L, Gaia, Silvia, Petrini, Elisa, Bosco, Caterina, Olivero, Antonella, et al. Risk of hepatocellular carcinoma in HBV cirrhotic patients assessed by the combination of miR-122, AFP and PIVKA-II. Panminerva medica 2017;59(4):283-89.

| Study | Study details | Study population | Index test and reference standard |
|---|---|---|---|
| Caviglia et al. (2020)[21] | **Study design:** 1-gate<br>**Method of recruitment:** Surveillance programme<br>**Setting:** Italy | **Participants, n:** 349 (HCC: 149; No HCC: 200)<br>**Age:** HCC: Median 67 (range 31, 89)<br>No HCC: Median 61 (range 33, 82)<br>**Aetiology:** HCC: HCV 109 (73.2%); HBV 40 (26.8%)<br>No HCC: HCV 148 (74.0%); HBV 52 (26.0%)<br>**Cirrhosis severity:** HCC: Child's grade A 131 (87.9%)<br>No HCC: Child's grade A 188 (94.0%) | **Index test(s):** AFP; DCP; AFP and DCP<br>**Reference standard:** Histology/imaging (Histology or contrast-enhanced imaging methods.) |
| Caviglia et al. (2023)[22] | **Study design:** 2-gate<br>**Method of recruitment:** Clinical cohort<br>**Setting:** Italy | **Participants, n:** 1187 (HCC: 205; No HCC: 982)<br>**Age:** HCC: Median 67 (range 61, 77)<br>No HCC: Median 64 (range 57, 76)<br>**Aetiology:** HCC: Other / unknown 62 (30.2%); HCV 106 (51.7%); HBV 37 (18.0%)<br>No HCC: Other / unknown 75 (7.6%); HCV 859 (87.5%); HBV 48 (4.9%)<br>**Cirrhosis severity:** HCC: Child's grade A 171 (83.4%)<br>No HCC: Child's grade A 922 (93.9%) | **Index test(s):** DCP<br>**Reference standard to establish presence of HCC:** Histology/imaging (HCC was diagnosed by histology or by imaging methods (multiphasic computed tomography or dynamic contrast-enhanced magnetic resonance), showing hypervascularity in the late arterial phase and washout on the portal venous and/or delayed phases)<br>**Reference standard to establish absence of HCC:** Imaging (The diagnosis of liver cirrhosis was achieved by histologic examination, or by liver elastrography (FibroScan®, Echosens™, Paris, France) and clinical and/or US features of portal hypertension) |

[21] Caviglia, Gian Paolo, Ciruolo, Michela, Abate, Maria Lorena, Carucci, Patrizia, Rolle, Emanuela, Rosso, Chiara, et al. Alpha-Fetoprotein, Protein Induced by Vitamin K Absence or Antagonist II and Glypican-3 for the Detection and Prediction of Hepatocellular Carcinoma in Patients with Cirrhosis of Viral Etiology. Cancers 2020;12(11):n. pag..

[22] Caviglia, Gian Paolo, Abate, Maria Lorena, Troshina, Giulia, Carucci, Patrizia, Rolle, Emanuela, Risso, Alessandra, et al. Identification of the Best Cut-Off Value of PIVKA-II for the Surveillance of Patients at Risk of Hepatocellular Carcinoma Development. Biology 2023;12(1):94.

| Study | Study details | Study population | Index test and reference standard |
|---|---|---|---|
| Chalasani et al. (2022) [23]<br>Secondary publication(s): Chalasani et al. (2023) | **Study design:** 2-gate<br>**Method of recruitment:** Clinical cohort<br>**Setting:** International | **Participants, n:** 401 (Control: 245; HCC: 156)<br>**Age:** HCC: Median 63 (IQR 59-70)<br>Control: Median 59 (IQR: 55-65)<br>**Aetiology:** Control: HCV 93 (38.0%); NAFLD/NASH 100 (40.8%); HBV 33 (13.5%)<br>HCC: HBV 21 (13.5%); NAFLD/NASH 52 (33.3%); HCV 74 (47.4%)<br>**Cirrhosis severity:** Control: Child's grade A 168 (68.6%)<br>HCC: Child's grade A 105 (67.3%) | **Index test(s):** mt-HBT<br>**Reference standard to establish presence of HCC:** Imaging ('Either a 1 cm or larger lesion showing arterial-phase hyperenhancement in combination with washout appearance and/or capsule by quadruple-phase computed tomography scan or multiphase contrast-enhanced magnetic resonance imaging; or a positive biopsy result.')<br>**Reference standard to establish absence of HCC:** Imaging ('The control group comprised at-risk patients with chronic liver disease undergoing HCC surveillance without evidence of HCC based on U/S, computed tomography scan, or magnetic resonance imaging within 3months before enrollment.') |
| Chang et al. (2015)[24] | **Study design:** 1-gate<br>**Method of recruitment:** Surveillance programme<br>**Setting:** Taiwan | **Participants, n:** 1597 (HCC: 363; No HCC: 1234)<br>**Age:** HCC: Mean 56.4 (SD 12)<br>No HCC: Mean 56.4 (SD 12)<br>**Aetiology:** HCC: HBV 137 (37.7%); HCV 245 (67.5%)<br>No HCC: HBV 508 (41.2%); HCV 689 (55.8%)<br>**Cirrhosis severity:** HCC: Child's grade A 281 (77.4%)<br>No HCC: Child's grade A 952 (77.1%) | **Index test(s):** AFP; US; US or AFP; serial AFP - absolute value above threshold AND do<br>**Reference standard:** Histology/imaging (AFP and US surveillance. CT, MRI and histology used for diagnosis when surveillance tests were positive.) |
| Chen et al. (2023)[25] | **Study design:** 2-gate<br>**Method of recruitment:** Clinical cohort<br>**Setting:** China | **Participants, n:** 435 (HCC: 262; No HCC: 173)<br>**Age:** HCC: Median 61 (range 52, 68)<br>No HCC: Median 57 (range 49, 65.5)<br>**Aetiology:** No HCC: HBV 115 (66.5%) | **Index test(s):** AFP; DCP; AFP and DCP<br>**Reference standard to establish presence of HCC:** Histology/imaging (HCC was diagnosed by histopathological examination of the biopsy or met the following criteria: imaging tests (US, CT, MRI, or other imaging tests) showing typical imaging injuries of HCC.)<br>**Reference standard to establish absence of HCC:** Not reported |

[23] Chalasani, N. P., Porter, K., Bhattacharya, A., Book, A. J., Neis, B. M., Xiong, K. M., et al. Validation of a Novel Multitarget Blood Test Shows High Sensitivity to Detect Early Stage Hepatocellular Carcinoma. Clinical Gastroenterology and Hepatology 2022;20(1):173-82.ee7.

[24] Chang, Te-Sheng, Wu, Yu-Chih, Tung, Shui-Yi, Wei, Kuo-Liang, Hsieh, Yung-Yu, Huang, Hao-Chun, et al. Alpha-Fetoprotein Measurement Benefits Hepatocellular Carcinoma Surveillance in Patients with Cirrhosis. The American journal of gastroenterology 2015;110(6):836-45.

[25] Chen, Yongwu, Yang, Xiuli, Shao, Yanfei, Zhao, Hongying, Jiang, Jinying, Huang, Ping, Lu, Yi. Comparison of diagnostic performance of AFP, DCP and two diagnostic models in hepatocellular carcinoma: a retrospective study. Annals of hepatology 2023;28(4):101099.

| Study | Study details | Study population | Index test and reference standard |
|---|---|---|---|
| Chen et al. (2024)[26] | **Study design:** 1-gate<br>**Method of recruitment:** Clinical cohort<br>**Setting:** China | **Participants, n:** 2293 (HCC: 76; No HCC: 2217)<br>**Age:** HCC: Mean 56.3 (SD 9.4)<br>No HCC: Mean 50.3 (SD 9.6)<br>**Aetiology:** HCC: HBV 52 (69.3%)<br>No HCC: HBV 1877 (84.7%)<br>**Cirrhosis severity:** HCC: Child's grade A 65 (86.7%)<br>No HCC: Child's grade A 1891 (85.3%) | **Index test(s):** AFP; DCP; B-mode US; AFP or B-mode US<br>**Reference standard:** Imaging (with 6-months imaging follow-up) (All patients with LC underwent enhanced CT or MRI when they were recruited in this study and were kept follow-up every 6 months for 3 years with a blood test and enhanced CT/MRI.) |
| Chen et al. (2024)[27] | **Study design:** 2-gate<br>**Method of recruitment:** Clinical cohort<br>**Setting:** China | **Participants, n:** 2377 (HCC: 377; No HCC: 2000)<br>**Age:** HCC: Mean 57.1 (SD 10.4)<br>No HCC: Mean 50.7 (SD 9.8)<br>**Aetiology:** HCC: HBV 119 (79.3%)<br>No HCC: HBV 3478 (82.5%)<br>**Cirrhosis severity:** HCC: Child's grade A 345 (94.0%)<br>No HCC: Child's grade A 3502 (84.6%) | **Index test(s):** AFP; DCP<br>**Reference standard to establish presence of HCC:** Imaging (with 6-months imaging follow-up) (All patients with LC underwent enhanced CT or MRI when they were recruited in this study and were kept follow-up every 6 months for 3 years with a blood test and enhanced CT/MRI.)<br>**Reference standard to establish absence of HCC:** Imaging (with 6-months imaging follow-up) (All patients with LC underwent enhanced CT or MRI when they were recruited in this study and were kept follow-up every 6 months for 3 years with a blood test and enhanced CT/MRI.) |
| Choi et al. (2008)[28] | **Study design:** 1-gate<br>**Method of recruitment:** Surveillance programme<br>**Setting:** South Korea | **Participants, n:** 47 (HCC: 23; No HCC: 24)<br>**Age:** HCC: Mean 50 (range 38, 62)<br>No HCC: Mean 48<br>**Aetiology:** HCC: Alcohol 0 (0.0%); HBV 20 (87.0%); HCV 3 (13.0%)<br>No HCC: HBV 17 (70.8%); HCV 0 (0.0%); Alcohol 4 (16.7%)<br>**Cirrhosis severity:** HCC: Child's grade A 3 (13.0%)<br>No HCC: Child's grade A 1 (4.2%) | **Index test(s):** CE-MRI<br>**Reference standard:** Explant pathology following transplantation |

[26] Chen, Lei, Wu, Tong, Fan, Rong, Qian, Yun-Song, Liu, Jing-Feng, Bai, Jian, et al. CHEN STAGE II 1-gate. EBioMedicine 2024;100():104962.
[27] Chen, Lei, Wu, Tong, Fan, Rong, Qian, Yun-Song, Liu, Jing-Feng, Bai, Jian, et al. Cell-free DNA testing for early hepatocellular carcinoma surveillance. EBioMedicine 2024;100():104962.
[28] Choi, Seung Hong, Lee, Jeong Min, Yu, Nam C., Suh, Kyung-Suk, Jang, Ja-June, Kim, Se Hyung. Hepatocellular carcinoma in liver transplantation candidates: detection with gadobenate dimeglumine-enhanced MRI. AJR. American journal of roentgenology 2008;191(2):529-36.

| Study | Study details | Study population | Index test and reference standard |
|---|---|---|---|
| Chong et al. (2022)[29] | **Study design:** 1-gate<br>**Method of recruitment:** Surveillance programme<br>**Setting:** USA | **Participants, n:** 2238 (HCC: 186; No HCC: 2052)<br>**Age:** No HCC: Median 59.5 (IQR: 53.6-65.0) HCC: Median 61.6 (IQR: 57.1-67.0)<br>**Aetiology:** HCC: NAFLD/NASH 16 (8.6%); HCV 109 (58.6%); Alcohol 31 (16.7%); HBV 19 (10.2%) No HCC: Alcohol 456 (22.2%); HCV 993 (48.4%); HBV 63 (3.1%); NAFLD/NASH 369 (18.0%)<br>**Cirrhosis severity:** HCC: Child's grade A 107 (57.5%) No HCC: Child's grade A 1684 (82.1%) | **Index test(s):** US<br>**Reference standard:** Imaging (CT and MRI scans within 12 months of each US. Cirrhosis patients had imaging or clinical follow-up for >1 year without evidence of HCC.) |
| Comunale et al. (2013)[30] | **Study design:** 2-gate<br>**Method of recruitment:** Clinical cohort<br>**Setting:** USA | **Participants, n:** 40 (HCC: 20; No HCC: 20)<br>**Age:** HCC: Mean 63.7 (SD 10.4) No HCC: Mean 60.8 (SD 8.8)<br>**Aetiology:** HCC: Alcohol 2 (10.0%); HCV 9 (45.0%) No HCC: HCV 9 (45.0%); Alcohol 2 (10.0%)<br>**Cirrhosis severity:** HCC: Child's grade A 12 (60.0%) No HCC: Child's grade A 1 (5.0%) | **Index test(s):** AFP<br>**Reference standard to establish presence of HCC:** Imaging (Histology for most cases. If histology wasn't available, two imaging modalities (US, MRI or CT).)<br>**Reference standard to establish absence of HCC:** Imaging (US for all patients. 'If serum AFP was elevated a MRI of the liver within 3 months prior to enrollment and another one 6 months after enrollment that showed no liver mass.' Controls had a median of 12 months (range 7–18 months) folow-up after enrollment.) |

[29] Chong, Nicolas, Schoenberger, Haley, Yekkaluri, Sruthi, Fetzer, David T., Rich, Nicole E., Yokoo, Takeshi, et al. Association between ultrasound quality and test performance for HCC surveillance in patients with cirrhosis: a retrospective cohort study. Alimentary pharmacology & therapeutics 2022;55(6):683-90.

[30] Comunale, Mary Ann, Wang, Mengjun, Anbarasan, Nikhil, Betesh, Lucy, Karabudak, Aykan, Moritz, Ethan, et al. Total serum glycan analysis is superior to lectin-FLISA for the early detection of hepatocellular carcinoma. Proteomics. Clinical applications 2013;7(9-10):690-700.

| Study | Study details | Study population | Index test and reference standard |
|---|---|---|---|
| Dabbish et al. (2022)[31] | **Study design:** 2-gate<br>**Method of recruitment:** Clinical cohort<br>**Setting:** Egypt | **Participants, n:** 139 (HCC: 72; No HCC: 67)<br>**Age:** HCC: ≤57: 27 (37.5%)<br>HCC: >57: 45 (62.5%)<br>HCC: Mean 61.4 (SD 8.1)<br>No HCC: >57: 31 (46.3%)<br>No HCC: ≤57: 36 (53.7%)<br>No HCC: Mean 59.2 (SD 11.9)<br>**Aetiology:** HCV NA (NA)<br>**Cirrhosis severity:** HCC: Child's grade A 54 (75.0%)<br>No HCC: Child's grade A 46 (68.7%) | **Index test(s):** AFP<br>**Reference standard to establish presence of HCC:** Blood tests and imaging (US and CT or MRI, and AFP.)<br>**Reference standard to establish absence of HCC:** Not reported |
| D'Abundo et al. (2024)[32] | **Study design:** 2-gate<br>**Method of recruitment:** Clinical cohort<br>**Setting:** Italy | **Participants, n:** 37 (HCC: 23; No HCC: 14)<br>**Age:** HCC: Mean 64.5<br>No HCC: Mean 64<br>**Aetiology:** HCC: HCV 15 (65.2%); Viral 2 (8.7%); Alcohol 1 (4.3%); NAFLD/NASH 2 (8.7%); HBV 3 (13.0%)<br>No HCC: NAFLD/NASH 0 (0.0%); HBV 3 (21.4%); HCV 10 (71.4%); Viral 0 (0.0%); Alcohol 1 (7.1%)<br>**Cirrhosis severity:** HCC: Child's grade A 22 (95.7%)<br>No HCC: Child's grade A 14 (100.0%) | **Index test(s):** AFP<br>**Reference standard to establish presence of HCC:** Imaging (HCC was diagnosed and staged by imaging techniques (contrast-enhanced multiphasic CT and/or dynamic contrast-enhanced Magnetic Resonance—MRI))<br>**Reference standard to establish absence of HCC:** Imaging |

[31] Dabbish, Areeg M., Abdelzaher, Hana M., Abohawya, Moustafa, Shamma, Samir, Mahmoud, Yosra H., Maged, Amr, et al. Prognostic MicroRNA Panel for HCV-Associated HCC: Integrating Computational Biology and Clinical Validation. Cancers 2022;14(13):n. pag..

[32] D'Abundo, L., Bassi, C., Callegari, E., Moshiri, F., Guerriero, P., Michilli, A., et al. Circulating microRNAs as biomarkers for stratifying different phases of liver cancer progression and response to therapy. Scientific reports 2024;14(1):18551.

| Study | Study details | Study population | Index test and reference standard |
|---|---|---|---|
| Demirtas et al. (2020)[33] | **Study design:** 1-gate<br>**Method of recruitment:** Surveillance programme<br>**Setting:** Turkey | **Participants, n:** 294 (HCC: 35; No HCC: 259)<br>**Age:** HCC: Median 66 (range 39, 86)<br>**Aetiology:** HCC: Alcohol 1 (2.9%); NAFLD/NASH 2 (5.7%); HBV 23 (65.7%); HCV 7 (20.0%)<br>No HCC: Alcohol 20 (7.7%); HCV 51 (19.7%); HBV 99 (38.2%); NAFLD/NASH 54 (20.8%)<br>**Cirrhosis severity:** HCC: Child's grade A 24 (68.6%)<br>No HCC: Child's grade A 205 (79.2%) | **Index test(s):** MRI; AFP; MRI or AFP<br>**Reference standard:** Histology/imaging (MRI surveillance. CT or biopsy performed after MRI for diagnosis in those with suspcious lesions.) |
| Ebrahim et al. (2020)[34] | **Study design:** 1-gate<br>**Method of recruitment:** Clinical cohort<br>**Setting:** Egypt | **Participants, n:** 50 (HCC: 25; No HCC: 25)<br>**Age:** HCC: Mean 56.4 (SD 4.8)<br>No HCC: Mean 46.2 (SD 6)<br>**Aetiology:** HCV 50 (100.0%) | **Index test(s):** AFP<br>**Reference standard:** Imaging (US for all patients and CT to confirm diagnosis.) |
| El-Abd et al. (2015)[35] | **Study design:** 2-gate<br>**Method of recruitment:** Clinical cohort<br>**Setting:** Egypt | **Participants, n:** 80 (HCC: 40; No HCC: 40)<br>**Age:** HCC: Mean 56.5 (SD 5.7)<br>No HCC: Mean 56.4 (SD 7.7)<br>**Aetiology:** HCV 80 (100.0%)<br>**Cirrhosis severity:** HCC: Child's grade A 19 (47.5%)<br>No HCC: Child's grade A 11 (27.5%) | **Index test(s):** AFP; miR-16; miR-16 or AFP<br>**Reference standard to establish presence of HCC:** Imaging (CT)<br>**Reference standard to establish absence of HCC:** Not reported (null) |

[33] Demirtas, Coskun Ozer, Gunduz, Feyza, Tuney, Davut, Baltacioglu, Feyyaz, Kani, Haluk Tarik, Bugdayci, Onur, Alahdab, Yesim Ozen. Annual contrast-enhanced magnetic resonance imaging is highly effective in the surveillance of hepatocellular carcinoma among cirrhotic patients. European journal of gastroenterology & hepatology 2020;32(4):517-23.

[34] Ebrahim, A. E., Shehata, M. A. H., Abou-saif, S., Hamisa, M., Abd-Elsalam, S. Role of Fibroscan for early detection of hepatocellular carcinoma (HCC) in hepatitis C cirrhotic patients. Egyptian Journal of Radiology and Nuclear Medicine 2020;51(1):134.

[35] El-Abd, Nevine E, Fawzy, Nahla A, El-Sheikh, Suzan M. Circulating miRNA-122, miRNA-199a, and miRNA-16 as Biomarkers for Early Detection of Hepatocellular Carcinoma in Egyptian Patients with Chronic Hepatitis C Virus Infection. Molecular diagnosis & therapy 2015;19(4):213-20.

| Study | Study details | Study population | Index test and reference standard |
|---|---|---|---|
| El-Ahwany et al. (2022)[36] | **Study design:** 2-gate<br>**Method of recruitment:** Clinical cohort<br>**Setting:** Egypt | **Participants, n:** 100 (HCC: 50; No HCC: 50)<br>**Age:** No HCC: >50: 35 (70.0%)<br>HCC: >50: 41 (82.0%)<br>**Aetiology:** HCV 100 (100.0%)<br>**Cirrhosis severity:** HCC: Child's grade A 25 (50.0%)<br>No HCC: Child's grade A 23 (46.0%) | **Index test(s):** Sat-a methylation; Alu methylation; AFP<br>**Reference standard to establish presence of HCC:** Imaging (All participants underwent medical history taking, clinical examination, screening laboratory investigations, α-fetoprotein (AFP) measurement, HCV-RNA RT-PCR, ultrasonography, and abdominal computed tomography (CT) for the confirmation of HCC.)<br>**Reference standard to establish absence of HCC:** Not reported |
| Eldeeb et al. (2020)[37] | **Study design:** 2-gate<br>**Method of recruitment:** Clinical cohort<br>**Setting:** Egypt | **Participants, n:** 60 (HCC: 30; No HCC: 30)<br>**Age:** HCC: Mean 56.7 (SD 6.1)<br>No HCC: Mean 58 (SD 7.2)<br>**Cirrhosis severity:** HCC: Child's grade A 0 (0.0%)<br>No HCC: Child's grade A 2 (6.7%) | **Index test(s):** AFP<br>**Reference standard to establish presence of HCC:** Imaging (CT)<br>**Reference standard to establish absence of HCC:** Imaging (CT) |
| El-Din Bessa et al. (2010)[38] | **Study design:** 2-gate<br>**Method of recruitment:** Clinical cohort<br>**Setting:** Egypt | **Participants, n:** 60 (HCC: 30; No HCC: 30)<br>**Age:** HCC: Mean 59.4 (SD 6.7)<br>No HCC: Mean 56.2 (SD 6.1)<br>**Aetiology:** HCV 60 (100.0%)<br>**Cirrhosis severity:** HCC: Child's grade A 10 (33.3%)<br>No HCC: Child's grade A 18 (60.0%) | **Index test(s):** AFP<br>**Reference standard to establish presence of HCC:** Histology/imaging (Imaging and/or histology according to AASL guidelines)<br>**Reference standard to establish absence of HCC:** Not reported (null) |

[36] El-Ahwany, Eman, Hassan, Marwa, Elzallat, Mohamed, Abdelsalam, Lobna, El-Sawy, Mohamed Abdel-Hameed. Association of Sat-a and Alu methylation status with HCV-induced chronic liver disease and hepatocellular carcinoma. Virus research 2022;321():198928.

[37] Eldeeb, Mona Kamal, Magour, Gehan Mahmoud, Bedair, Rania Nabil, Shamseya, Mohammed Mahmoud. Study of Dickkopf-1 (DKK-1) in patients with chronic viral hepatitis C-related liver cirrhosis with and without hepatocellular carcinoma. Clinical and experimental hepatology 2020;6(2):85-91.

[38] El-Din Bessa, Sahar Saad, Elwan, Nadia Mohamed, Suliman, Ghada Abdul Moemen. Clinical significance of plasma osteopontin level in Egyptian patients with hepatitis C virus-related hepatocellular carcinoma. Archives of medical research 2010;41(7):541-7.

| Study | Study details | Study population | Index test and reference standard |
| --- | --- | --- | --- |
| Eldosoky et al. (2023)[39] | **Study design:** 2-gate<br>**Method of recruitment:** Clinical cohort<br>**Setting:** Egypt | **Participants, n:** 79 (HCC: 39; No HCC: 40)<br>**Age:** HCC: Median 61 (range 56, 67)<br>No HCC: Median 58.5 (range 54.25, 65)<br>**Aetiology:** HCV 79 (100.0%)<br>**Cirrhosis severity:** HCC: Child's grade A 24 (61.5%)<br>No HCC: Child's grade A 12 (30.0%) | **Index test(s):** AFP; hsa-miR-21-5p-fold changes; hsa-miR-155-5p-fold changes; hsa-miR-199a-5p fold changes; hsa-miR-21-5p-fold changes & AFP; hsa-miR-155-5p-fold changes & AFP; hsa-miR-199a-5p fold changes & AFP; hsa-miR-21-5p/hsa-miR-199a-5p; hsa-miR-155-5p/hsa-miR-199a-5p<br>**Reference standard to establish presence of HCC:** Imaging<br>**Reference standard to establish absence of HCC:** Imaging |
| El-Emshaty et al. (2025)[40] | **Study design:** 1-gate<br>**Method of recruitment:** Clinical cohort<br>**Setting:** Egypt | NA | **Index test(s):** AFP<br>**Reference standard:** Histology (Possibly explant histopathology, but not explicit) |
| Elgedawy et al. (2025)[41] | **Study design:** 2-gate<br>**Method of recruitment:** Clinical cohort<br>**Setting:** Egypt | **Participants, n:** 100 (HCC: 50; No HCC: 50)<br>**Age:** HCC: Mean 51.3 (SD 4.6)<br>No HCC: Mean 50.3 (SD 4.6)<br>**Cirrhosis severity:** HCC: Child's grade A 19 (38.0%)<br>No HCC: Child's grade A 17 (34.0%) | **Index test(s):** AFP; miR-485-5p<br>**Reference standard to establish presence of HCC:** Imaging (Patients with suspected HCC underwent a triphasic CT scan of the chest, abdomen, and pelvis for confirming HCC diagnosis and determining the presence of distant metastases.)<br>**Reference standard to establish absence of HCC:** Not reported |
| El-Mezayen & Darwish (2014)[42] | **Study design:** 2-gate<br>**Method of recruitment:** Clinical cohort<br>**Setting:** Egypt | **Participants, n:** 333 (HCC: 123; No HCC: 210)<br>**Age:** HCC: Mean 59.4 (SD 12.5)<br>No HCC: Mean 54.3 (SD 4.3)<br>**Aetiology:** HCV 333 (100.0%) | **Index test(s):** AFP<br>**Reference standard to establish presence of HCC:** Histology/imaging (Histology or new hepatic focal lesion by US and confirmed by CT, and AFP >200 U L−1.)<br>**Reference standard to establish absence of HCC:** Blood tests and imaging (2 years of follow-up from sample collection. The follow-up included US and AFP every 6 months.) |

[39] Eldosoky, Mona A., Hammad, Reham, Elmadbouly, Asmaa A., Aglan, Reda Badr, Abdel-Hamid, Sherihan G., Alboraie, Mohamed, et al. Diagnostic Significance of hsa-miR-21-5p, hsa-miR-192-5p, hsa-miR-155-5p, hsa-miR-199a-5p Panel and Ratios in Hepatocellular Carcinoma on Top of Liver Cirrhosis in HCV-Infected Patients. International journal of molecular sciences 2023;24(4):n. pag..

[40] El-Emshaty, H. M., Eladle, E. A., Abdel-Wahab, M. Potential role of pentraxin 3 (PTX3) and aldehyde dehydrogenase (ALDH) in liver cirrhotic patients: predicting and diagnostic impact in hepatocellular carcinoma (HCC) development. Molecular Biology Reports 2025;52(1):826.

[41] Elgedawy, G. A., Elabd, N. S., Elbrolosy, A. M., El-Morshedy, S. M., El-Gamal, A., Abozeid, M., et al. Circulating miR-485-5p as a potential diagnostic and prognostic biomarker for HCV-related hepatocellular carcinoma. Clinical and Experimental Medicine 2025;25(1):110.

[42] El-Mezayen, H. A. Development of a novel score for early detection of hepatocellular carcinoma among high-risk hepatitis C virus patients. Tumor Biology 2014;35(7):6501-09.

| Study | Study details | Study population | Index test and reference standard |
| --- | --- | --- | --- |
| El-Serag et al. (2025)[43] | **Study design:** 1-gate<br>**Method of recruitment:** Surveillance programme<br>**Setting:** USA | **Participants, n:** 2249 (HCC: 43; No HCC: 2206)<br>**Age:** HCC: Mean 62.1 (SD 8.3)<br>No HCC: Mean 59.6 (SD 10.3)<br>**Aetiology:** HCC: HCV 64 (51.2%); Alcohol 16 (12.8%); NAFLD/NASH 31 (24.8%); Other / unknown 14 (11.2%)<br>No HCC: HCV 807 (36.6%); Alcohol 377 (17.1%); NAFLD/NASH 730 (33.1%); Other / unknown 292 (13.2%)<br>**Cirrhosis severity:** HCC: Child's grade A 72 (60.0%)<br>No HCC: Child's grade A 1443 (68.2%) | **Index test(s):** AFP; AFP-L3; DCP; GALAD; HES v1; HES v2<br>**Reference standard:** Imaging (with 6-months any follow-up) (We defined new HCC according to the American Association for the Study of Liver Diseases criteria, including histological or radiological diagnosis using characteristic appearance (arterial enhancement and delayed washout of contrast) on triple-phase CT or MRI (LI-RAD 5).) |
| El-Shayeb et al. (2021)[44] | **Study design:** 1-gate<br>**Method of recruitment:** Clinical cohort<br>**Setting:** Egypt | **Participants, n:** 175 (HCC: 86; No HCC: 89)<br>**Age:** HCC: Median 59 (IQR: 55-62)<br>No HCC: Median 52 (IQR: 47-60)<br>**Aetiology:** HCV 175 (100.0%)<br>**Cirrhosis severity:** HCC: Child's grade A 26 (30.2%)<br>No HCC: Child's grade A 21 (23.6%) | **Index test(s):** AFP<br>**Reference standard:** Imaging (US for all patients, confirmed using CT.) |
| El-Shendidi et al. (2022)[45] | **Study design:** 2-gate<br>**Method of recruitment:** Clinical cohort<br>**Setting:** Egypt | **Participants, n:** 80 (HCC: 40; No HCC: 40)<br>**Age:** HCC: Mean 61 (SD 5)<br>No HCC: Mean 56 (SD 7.6)<br>**Aetiology:** HCC: HCV 40 (100.0%)<br>No HCC: HCV 40 (100.0%) | **Index test(s):** AFP; HOTAIR expression (2-ΔΔCT); AFP and 2-ΔΔct HOTAIR<br>**Reference standard to establish presence of HCC:** Imaging (Diagnosis confirmed by CT and/or MRI.)<br>**Reference standard to establish absence of HCC:** Not reported (null) |

[43] El-Serag, Hashem B., Jin, Qingchun, Tayob, Nabihah, Salem, Emad, Luster, Michelle, Alsarraj, Abeer, et al. HES V2.0 outperforms GALAD for detection of HCC: A phase 3 biomarker study in the United States. Hepatology (Baltimore, Md.) 2025;81(2):465â€“475.

[44] El-Shayeb, Ayman F., El-Habachi, Nihal M., Mansour, Amal R. Serum midkine is a more sensitive predictor for hepatocellular carcinoma than Dickkopf-1 and alpha-L-fucosidase in cirrhotic HCV patients. Medicine 2021;100(17):e25112.

[45] El-Shendidi, A. & Ghazala, R. Circulating HOTAIR potentially predicts hepatocellular carcinoma in cirrhotic liver and prefigures the tumor stage. Clinical and Experimental Hepatology 2022;8(2):139-46.

| Study | Study details | Study population | Index test and reference standard |
|---|---|---|---|
| Elshimi et al. (2019)[46] | **Study design:** 2-gate<br>**Method of recruitment:** Clinical cohort<br>**Setting:** Egypt | **Participants, n:** 200 (HCC: 100; No HCC: 100)<br>**Age:** HCC: (range 47, 71)<br>No HCC: (range 45, 69)<br>**Aetiology:** HCV 200 (100.0%) | **Index test(s):** MMP-12 mRNA overexpression; MMP-12 mRNA overexpression or AFP<br>**Reference standard to establish presence of HCC:** Blood tests and imaging (Liver profile, AFP, viral markers and imaging (mainly CT and MRI).)<br>**Reference standard to establish absence of HCC:** Blood tests and imaging (Patients without HCC had liver profile, AFP, viral markers, and US. Some cases had CT or MRI if AFP was above 200 μg/dL and/or lesions were observed at US.) |
| Ergul et al. (2024)[47] | **Study design:** 2-gate<br>**Method of recruitment:** Clinical cohort<br>**Setting:** Turkey | **Participants, n:** 72 (HCC: 33; No HCC: 39)<br>**Age:** HCC: Mean 67.2 (SD 7.9)<br>No HCC: Mean 62.9 (SD 11) | **Index test(s):** AFP<br>**Reference standard to establish presence of HCC:** Not reported<br>**Reference standard to establish absence of HCC:** Not reported |
| Farid et al. (2014)[48] | **Study design:** 2-gate<br>**Method of recruitment:** Clinical cohort<br>**Setting:** Egypt | **Participants, n:** 40 (HCC: 20; No HCC: 20)<br>**Age:** HCC: Mean 58 (SD 9)<br>No HCC: Mean 47 (SD 8)<br>**Aetiology:** No HCC: HCV 20 (100.0%)<br>**Cirrhosis severity:** HCC: Child's grade A 5 (25.0%)<br>No HCC: Child's grade A 0 (0.0%) | **Index test(s):** AFP; TGF-b1 mRNA; AFP or TGF-b1 mRNA<br>**Reference standard to establish presence of HCC:** Blood tests and imaging (AFP, CT and MRI)<br>**Reference standard to establish absence of HCC:** Imaging (US at enrollment) |
| Feier et al. (2013)[49] | **Study design:** 2-gate<br>**Method of recruitment:** Clinical cohort<br>**Setting:** Romania | **Participants, n:** 21 (HCC: 21)<br>**Age:** Mean 63.2 (validation study)<br>**Aetiology:** HCV 40 (100.0%) | **Index test(s):** Model combining LSM, LS IQR, ALAT and AFP<br>**Reference standard to establish presence of HCC:** Imaging (null)<br>**Reference standard to establish absence of HCC:** Imaging (null) |

[46] Elshimi, Esam, Sakr, Mostafa Abdel-Samed Mostafa, Morad, Wesam Saber. Optimizing the Diagnostic Role of Alpha-Fetoprotein and Abdominal Ultrasound by Adding Overexpressed Blood mRNA Matrix Metalloproteinase-12 for Diagnosis of HCV-Related Hepatocellular Carcinoma. Gastrointestinal tumors 2019;5(3-4):100-08.

[47] Ergul, B., Gul, O., Kisa, U., Erdal, H., Tekin, E. Association of Low Serum Maresin-1 Levels with Hepatocellular Carcinoma in Cirrhotic Liver. Clinical Laboratory 2024;70(12):2268 “ 2274.

[48] Farid, Ibtisam M, Hamza, Iman M, El-Abd, Dina M, Mohyi, Abeer M, AbdulLatif, Mona M A, Aref, Adel T. Transforming growth factor-beta1 gene expression in hepatocellular carcinoma: a preliminary report. Arab journal of gastroenterology : the official publication of the Pan-Arab Association of Gastroenterology 2014;15(3-4):142-7.

[49] Feier, Diana, Lupsor Platon, Monica, Stefanescu, Horia. Transient elastography for the detection of hepatocellular carcinoma in viral C liver cirrhosis. Is there something else than increased liver stiffness? Journal of gastrointestinal and liver diseases : JGLD 2013;22(3):283-89.

| Study | Study details | Study population | Index test and reference standard |
|---|---|---|---|
| Gani et al. (2015)[50] | **Study design:** 1-gate<br>**Method of recruitment:** Clinical cohort<br>**Setting:** Indonesia | **Participants, n:** 106 (HCC: 59; No HCC: 47)<br>**Age:** HCC: Mean 55.5 (SD 12)<br>No HCC: Mean 55.6 (SD 11.6)<br>**Aetiology:** HCC: HBV 20 (33.9%); HCV 39 (66.1%)<br>No HCC: HBV 15 (31.9%); HCV 28 (59.6%) | **Index test(s):** AFP<br>**Reference standard:** Blood tests and imaging (According to AASLD guidelines or 'by presence of liver nodule, AFP >200 ng/mL and supported with two imaging results'.) |
| Giannelli et al. (2005)[51] | **Study design:** 2-gate<br>**Method of recruitment:** Clinical cohort<br>**Setting:** Italy | **Participants, n:** 210 (HCC: 120; No HCC: 90)<br>**Age:** HCC: Mean 66.5 (SD 8)<br>No HCC: Mean 61.5 (SD 11.2)<br>**Aetiology:** HCC: HBV 25 (20.8%); HCV 94 (78.3%); Alcohol 2 (1.7%)<br>No HCC: HBV 29 (32.2%); HCV 67 (74.4%)<br>**Cirrhosis severity:** HCC: Child's grade A 82 (68.3%)<br>No HCC: Child's grade A 66 (73.3%) | **Index test(s):** AFP<br>**Reference standard to establish presence of HCC:** Imaging (US and CT when necessary)<br>**Reference standard to establish absence of HCC:** Not reported (null) |
| Gobel et al. (2006)[52] | **Study design:** 2-gate<br>**Method of recruitment:** Clinical cohort<br>**Setting:** Germany | **Participants, n:** 78 (HCC: 34; No HCC: 44)<br>**Age:** HCC: Mean 67 (SD 8)<br>No HCC: Mean 62 (SD 8)<br>**Aetiology:** HCV 78 (100.0%)<br>**Cirrhosis severity:** HCC: Child's grade A 20 (58.8%)<br>No HCC: Child's grade A 35 (79.5%) | **Index test(s):** AFP<br>**Reference standard to establish presence of HCC:** Histology ('Liver biopsy was available in all patients.')<br>**Reference standard to establish absence of HCC:** Not reported |

[50] Gani, Rino A, Suryamin, Maulana, Hasan, Irsan, Lesmana, C Rinaldi A. Performance of Alpha Fetoprotein in Combination with Alpha-1-acid Glycoprotein for Diagnosis of Hepatocellular Carcinoma Among Liver Cirrhosis Patients. Acta medica Indonesiana 2015;47(3):216-22.

[51] Giannelli, Gianluigi, Marinosci, Felice, Trerotoli, Paolo, Volpe, Anna, Quaranta, Michele, Dentico, Pietro. SCCA antigen combined with alpha-fetoprotein as serologic markers of HCC. International journal of cancer 2005;117(3):506-9.

[52] Gobel, T., Vordenwulbecke, S., Hauck, K., Fey, H., Haussinger, D. New multi protein patterns differentiative liver fibrosis stages and hepatocellular carcinoma in chronic hepatitis C serum samples. World Journal of Gastroenterology 2006;12(47):7604-12.

| Study | Study details | Study population | Index test and reference standard |
|---|---|---|---|
| Gomaa et al. (2020)[53] | **Study design:** 2-gate<br>**Method of recruitment:** Clinical cohort<br>**Setting:** Egypt | **Participants, n:** 60 (HCC: 30; No HCC: 30)<br>**Age:** HCC: Mean 60.9 (SD 9.7)<br>No HCC: Mean 56.7 (SD 8.7)<br>**Aetiology:** HCV 60 (100.0%)<br>**Cirrhosis severity:** HCC: Child's grade A 1 (3.3%)<br>No HCC: Child's grade A 4 (13.3%) | **Index test(s):** AFP<br>**Reference standard to establish presence of HCC:** Imaging (CT, and histology in some cases.)<br>**Reference standard to establish absence of HCC:** Imaging (US. Suspicious lesions assessed by CT.) |
| Gopal et al. (2014)[54] | **Study design:** 2-gate<br>**Method of recruitment:** Clinical cohort<br>**Setting:** USA | **Participants, n:** 1128 (HCC: 452; No HCC: 676)<br>**Age:** HCC: Median 56 (IQR: 52-61)<br>No HCC: Median 55 (IQR: 49-61)<br>**Aetiology:** HCC: NAFLD/NASH 39 (8.6%); Alcohol 56 (12.4%); HCV 314 (69.5%); HBV 41 (9.1%)<br>No HCC: HCV 362 (53.6%); HBV 23 (3.4%); NAFLD/NASH 77 (11.4%); Alcohol 192 (28.4%)<br>**Cirrhosis severity:** HCC: Child's grade A 168 (37.2%)<br>No HCC: Child's grade A 403 (59.6%) | **Index test(s):** AFP; Doylestown algorithm<br>**Reference standard to establish presence of HCC:** Imaging (AASLD guidelines (CT and MRI))<br>**Reference standard to establish absence of HCC:** Not reported (6 months follow-up - type of follow-up unclear.) |
| Guan et al. (2022)[55] | **Study design:** 2-gate<br>**Method of recruitment:** Clinical cohort<br>**Setting:** China | **Participants, n:** 104<br>**Aetiology:** NAFLD/NASH 104 (100.0%)<br>**Cirrhosis severity:** Child's grade A-B 104 (100.0%) | **Index test(s):** AFP; DCP; AFP-L3; AFP or DCP; DCP or AFP-L3; AFP or AFP-L3; AFP or DCP or AFP-L3<br>**Reference standard to establish presence of HCC:** Histology/imaging (US, CT or MRI. Most also had histology.)<br>**Reference standard to establish absence of HCC:** Not reported |

53 Gomaa, Salwa H., Abaza, Mona M., Elattar, Hoda A., Amin, Gamal A. Soluble cluster of differentiation 26/soluble dipeptidyl peptidase-4 and glypican-3 are promising serum biomarkers for the early detection of Hepatitis C virus related hepatocellular carcinoma in Egyptians. Arab journal of gastroenterology : the official publication of the Pan-Arab Association of Gastroenterology 2020;21(4):224-32.

54 Gopal, Purva, Yopp, Adam C, Waljee, Akbar K, Chiang, Jason, Nehra, Mahendra, Kandunoori, Pragathi. Factors that affect accuracy of alpha-fetoprotein test in detection of hepatocellular carcinoma in patients with cirrhosis. Clinical gastroenterology and hepatology : the official clinical practice journal of the American Gastroenterological Association 2014;12(5):870-7.

55 Guan, Ming-Cheng, Ouyang, Wei, Liu, Si-Yu, Sun, Li-Yang, Chen, Wei-Yue, Tong, Xiang-Min, Zhu, Hong. Alpha-fetoprotein, protein induced by vitamin K absence or antagonist-II, lens culinaris agglutinin-reactive fraction of alpha-fetoprotein alone and in combination for early detection of hepatocellular carcinoma from nonalcoholic fatty liver disease: A multicenter analysis. Hepatobiliary & pancreatic diseases international : HBPD INT 2022;():n. pag..

| Study | Study details | Study population | Index test and reference standard |
|---|---|---|---|
| Gui et al. (2011)[56] | **Study design:** 2-gate<br>**Method of recruitment:** Clinical cohort<br>**Setting:** China | **Participants, n:** 32 (HCC: 20; No HCC: 12)<br>**Age:** HCC: Mean 58.3 (SD 6.2) No HCC: Mean 61.2 (SD 7.5) | **Index test(s):** AFP; miRNA-885-5p<br>**Reference standard to establish presence of HCC:** Not reported (null)<br>**Reference standard to establish absence of HCC:** Not reported |
| Guo et al. (2022)[57] | **Study design:** 2-gate<br>**Method of recruitment:** Clinical cohort<br>**Setting:** China | **Participants, n:** 41 (HCC: 21; No HCC: 20)<br>**Age:** HCC: Median 59 (IQR: 52.0-65.0) No HCC: Median 49.5 (IQR: 44.0-55.0)<br>**Aetiology:** HBV 41 (100.0%) | **Index test(s):** AFP<br>**Reference standard to establish presence of HCC:** Imaging (US or CT and AFP ≥400 ng/mL.)<br>**Reference standard to establish absence of HCC:** Not reported |
| Hai et al. (2025)[58] | **Study design:** 2-gate<br>**Method of recruitment:** Surveillance programme<br>**Setting:** Vietnam | NA | **Index test(s):** AFP; DCP; GAAD<br>**Reference standard to establish presence of HCC:** Not reported<br>**Reference standard to establish absence of HCC:** Imaging (with 6-months imaging follow-up) (Eligible LC, CHB, and BLD cases were confirmed to be free of HCC by ultrasound or computed tomography imaging after 06 months (+0–8 weeks) of sampling (in the next routine examination)) |
| Hallager et al. (2018)[59] | **Study design:** 1-gate<br>**Method of recruitment:** Surveillance programme<br>**Setting:** Denmark | **Participants, n:** 107 (HCC: 107)<br>**Age:** Median 51.5 (range 26, 86)<br>**Aetiology:** HBV 38 (3.5%); HCV 1075 (100.0%) | **Index test(s):** AFP<br>**Reference standard:** ICD-9 codes (Used ICD-10 codes and Danish Cancer Registry to retrieve diagnosis.) |

[56] Gui, Junhao, Tian, Yaping, Wen, Xinyu, Zhang, Wenhui, Zhang, Pengjun, Gao, Jing, et al. Serum microRNA characterization identifies miR-885-5p as a potential marker for detecting liver pathologies. Clinical science (London, England : 1979) 2011;120(5):183-93.

[57] Guo, Li, Wan, Lijun, Hu, Youwen, Huang, Hongyan, He, Bo. Serum N-glycan profiling as a diagnostic biomarker for the identification of hepatitis B virus-associated hepatocellular carcinoma. Journal of gastrointestinal oncology 2022;13(1):344-54.

[58] Hai, N. T. T., Doi, N. A., Nguyen, N. H., Hoang, P. D., Thu, N. K., Vu, T. T. H., et al. Diagnostic validation of the GAAD algorithm for hepatitis B virus-related hepatocellular carcinoma surveillance in Vietnam. Journal of Laboratory Medicine 2025;():n. pag..

[59] Hallager, S, Ladelund, S, Kjaer, M, Madsen, L G, Belard, E, Laursen, A L, et al. Hepatocellular carcinoma in patients with chronic hepatitis C and cirrhosis in Denmark: A nationwide cohort study. Journal of viral hepatitis 2018;25(1):47-55.

| Study | Study details | Study population | Index test and reference standard |
|---|---|---|---|
| Hammad et al. (2023)[60] | **Study design:** 2-gate<br>**Method of recruitment:** Clinical cohort<br>**Setting:** Egypt | **Participants, n:** 79 (HCC: 39; No HCC: 40)<br>**Age:** HCC: Median 61 (range 56, 67)<br>No HCC: Median 58.5 (range 54.25, 65)<br>**Cirrhosis severity:** HCC: Child's grade A 24 (61.5%)<br>No HCC: Child's grade A 12 (30.0%) | **Index test(s):** AFP; hsa-miR-21-5p-fold changes; hsa-miR-155-5p-fold changes; hsa-miR-21-5p OR hsa-miR-155-5p<br>**Reference standard to establish presence of HCC:** Imaging (A blind abdominal computed tomography (CT) scan was performed using Siemens 128 (Germany). CHCV fulfilling imaging criteria in accordance with recent recommendations were used to confirm the HCC diagnosis.)<br>**Reference standard to establish absence of HCC:** Not reported |
| Hanna et al. (2008)[61] | **Study design:** 1-gate<br>**Method of recruitment:** Surveillance programme<br>**Setting:** USA | **Participants, n:** 25 (HCC: 25)<br>**Age:** Mean 54 (range 36, 68)<br>**Aetiology:** HCV 35 (72.9%); HBV 1 (2.1%); Alcohol 10 (20.8%) | **Index test(s):** Double CE-MRI<br>**Reference standard:** Explant pathology following transplantation |
| Hassan et al. (2019)[62] | **Study design:** 2-gate<br>**Method of recruitment:** Clinical cohort<br>**Setting:** Egypt | **Participants, n:** 40 (HCC: 20; No HCC: 20)<br>**Age:** HCC: Mean 61.6 (SD 6.4)<br>No HCC: Mean 59 (SD 7.2)<br>**Aetiology:** HCV 40 (100.0%) | **Index test(s):** mRNA-483-5p; mRNA-133a; AFP; miR-483-5p and AFP; miR-133a and AFP<br>**Reference standard to establish presence of HCC:** Blood tests and imaging (US, CT and AFP.)<br>**Reference standard to establish absence of HCC:** Not reported |
| Hawash et al. (2024)[63] | **Study design:** 2-gate<br>**Method of recruitment:** Clinical cohort<br>**Setting:** Egypt | **Participants, n:** 162 (HCC: 81; No HCC: 81)<br>**Age:** HCC: Mean 61.5 (SD 6.1)<br>No HCC: Mean 57.6 (SD 7.4) | **Index test(s):** AFP<br>**Reference standard to establish presence of HCC:** Imaging (all included patients had US; patients in whom focal lesions were detected had CT for confirmation of HCC)<br>**Reference standard to establish absence of HCC:** Imaging |

[60] Hammad, Reham, Eldosoky, Mona A., Elmadbouly, Asmaa A., Aglan, Reda Badr, AbdelHamid, Sherihan G., Zaky, Samy, et al. Monocytes subsets altered distribution and dysregulated plasma hsa-miR-21-5p and hsa-miR-155-5p in HCV-linked liver cirrhosis progression to hepatocellular carcinoma. Journal of cancer research and clinical oncology 2023;149(17):15349â€“15364.

[61] Hanna, Robert F., Kased, Norbert, Kwan, Sharon W., Gamst, Anthony C., Santosa, Agnes C., Hassanein, Tarek. Double-contrast MRI for accurate staging of hepatocellular carcinoma in patients with cirrhosis. AJR. American journal of roentgenology 2008;190(1):47-57.

[62] Hassan, Asmaa S., Elgendy, Naglaa A., Tawfik, Naglaa A. Serum miR-483-5p and miR-133a as Biomarkers for Diagnosis of Hepatocellular Carcinoma Post-Hepatitis C Infection in Egyptian Patients. The Egyptian journal of immunology 2019;26(2):31-40.

[63] Hawash, N., Gameaa, R., Elwy, D. Red Blood Cell Distribution Width as a Diagnostic Marker of Hepatocellular Carcinoma in Cirrhotic Patients. Open Biomarkers Journal 2024;14():e18753183275959.

| Study | Study details | Study population | Index test and reference standard |
|---|---|---|---|
| Henry et al. (2009)[64] | **Study design:** 1-gate<br>**Method of recruitment:** Clinical cohort<br>**Setting:** France | **Participants, n:** 83 (HCC: 50; No HCC: 33)<br>**Age:** HCC: Mean 62.5 (SD 9.9)<br>No HCC: Mean 56.5 (SD 11.5)<br>**Aetiology:** HCC: Alcohol 26 (52.0%); Hepatitis 20 (40.0%)<br>No HCC: Alcohol 16 (48.5%); Hepatitis 10 (30.3%)<br>**Cirrhosis severity:** HCC: Child's grade A 26 (52.0%)<br>No HCC: Child's grade A 9 (27.3%) | **Index test(s):** AFP<br>**Reference standard:** Histology/imaging (Histology or one imaging technique (MRI or CT) with arterial hypervascularisation and AFP >400 ng/ml. For cirrhosis patients without HCC, histology following transplantation. Normal MRI or two other imaging studies techniques at >1 year interval.) |
| Hernandez et al. (2011)[65] | **Study design:** 1-gate<br>**Method of recruitment:** Surveillance programme<br>**Setting:** Cuba | **Participants, n:** 189 (HCC: 22; No HCC: 167)<br>**Age:** HCC: Mean 55 (SD 12)<br>No HCC: Mean 43.2 (SD 12.5)<br>**Aetiology:** HCC: HBV 6 (27.3%); Alcohol 4 (18.2%); HCV 8 (36.4%)<br>No HCC: HBV 20 (12.0%); HCV 59 (35.3%); Alcohol 43 (25.7%) | **Index test(s):** AFP; US; AFP or US<br>**Reference standard:** Histology/imaging (EASLD criteria for patients with HCC and tumoral lesions larger than 2 cm.) |
| Hodeib et al. (2017)[66] | **Study design:** 2-gate<br>**Method of recruitment:** Clinical cohort<br>**Setting:** Egypt | **Participants, n:** 70 (HCC: 35; No HCC: 35)<br>**Age:** HCC: Mean 49.1 (SD 4.6)<br>No HCC: Mean 51.5 (SD 5.3)<br>**Aetiology:** No HCC: HCV 35 (100.0%); HCV 35 (100.0%) | **Index test(s):** AFP<br>**Reference standard to establish presence of HCC:** Histology (null)<br>**Reference standard to establish absence of HCC:** Not reported |
| Hussein et al. (2012)[67] | **Study design:** 2-gate<br>**Method of recruitment:** Clinical cohort<br>**Setting:** Egypt | **Participants, n:** 45<br>**Aetiology:** HCV 45 (100.0%) | **Index test(s):** MAGE-4 mRNA; AFP<br>**Reference standard to establish presence of HCC:** Not reported<br>**Reference standard to establish absence of HCC:** Not reported |

[64] Henry, L., Lavabre-Bertrand, T., Vercambre, L., Ramos, J., Carillo, S., Guiraud, I., et al. Plasma proteasome level is a reliable early marker of malignant transformation of liver cirrhosis. Gut 2009;58(6):833-38.

[65] Hernandez, J. C., Samada, M., Roque, A., Cruz, Y., Howland, I. Diagnostic value of alpha-fetoprotein for hepatocellular carcinoma. Biotecnologia Aplicada 2011;28(1):28-39.

[66] Hodeib, Hossam, Elshora, Ola, Selim, Amal, Sabry, Nesreen Mohammed. Serum Midkine and Osteopontin Levels as Diagnostic Biomarkers of Hepatocellular Carcinoma. Electronic physician 2017;9(1):3492-98.

[67] Hussein, Yousri M., Morad, Fouad E., Gameel, Magda A., Emam, Wafaa A., El Sawy, Wael H., El Tarhouny, Shereen A., Bayomy, Eman S. MAGE-4 gene m-RNA and TGF in blood as potential biochemical markers for HCC in HCV-infected patients. Medical oncology (Northwood, London, England) 2012;29(5):3055-62.

| Study | Study details | Study population | Index test and reference standard |
|---|---|---|---|
| Ibrahim et al. (2021)[68] | **Study design:** 2-gate<br>**Method of recruitment:** Clinical cohort<br>**Setting:** Egypt | **Participants, n:** 40 (HCC: 40) | **Index test(s):** AFP; AFP-L3<br>**Reference standard to establish presence of HCC:** Imaging (CT and US)<br>**Reference standard to establish absence of HCC:** Not reported |
| Ibrahim et al. (2025)[69] | **Study design:** 2-gate<br>**Method of recruitment:** Clinical cohort<br>**Setting:** Egypt | **Participants, n:** 105 (HCC: 70; No HCC: 35)<br>**Aetiology:** HCC: HBV 55 (78.6%); HCV 15 (21.4%)<br>**Cirrhosis severity:** HCC: Child's grade A 41 (58.6%) | **Index test(s):** AFP; miR-15b; miR-130b<br>**Reference standard to establish presence of HCC:** Unclear (diagnosed according to the guidelines proposed by the American Association for the Study of Liver Diseases (AASLD))<br>**Reference standard to establish absence of HCC:** Imaging (All cirrhotic patients underwent regular ultrasonographic screening (every 4–6 months) to  exclude the development of any hepatic nodules) |
| Istemihan et al. (2025)[70] | **Study design:** 2-gate<br>**Method of recruitment:** Surveillance programme<br>**Setting:** Turkey | **Participants, n:** 100 (HCC: 31; No HCC: 69)<br>**Age:** HCC: Mean 66.7 (SD 8.2)<br>No HCC: Mean 57.5 (SD 14.6)<br>**Aetiology:** HBV 34 (17.0%); HCV 4 (2.0%); Alcohol 7 (3.5%); Other / unknown 33 (16.5%); NAFLD/NASH 16 (8.0%); Autoimmune hepatitis 6 (3.0%) | **Index test(s):** GALAD<br>**Reference standard to establish presence of HCC:** Imaging (every cirrhotic patient is screened for HCC every 6 months with AFP and US. All patients in this study underwent MRI after US. US findings were verified with MRI)<br>**Reference standard to establish absence of HCC:** Imaging (3 months' follow-up ('In… patients [without] HCC..., MRIs were performed within 3 months at the latest after the… GALAD score, and no patients with HCC were detected in this group')) |
| Jalli et al. (2015)[71] | **Study design:** 2-gate<br>**Method of recruitment:** Surveillance programme<br>**Setting:** Iran | **Participants, n:** 96 (HCC: 30; No HCC: 66) | **Index test(s):** Noncontrast MRI; US<br>**Reference standard to establish presence of HCC:** Histology<br>**Reference standard to establish absence of HCC:** Not reported (null) |

[68] Ibrahim, Hassnaa M., Elghannam, Magdy Z., Elkhawaga, Om Ali Y. Evaluation of serum alpha fetoprotein-L3 as an accuracy novel biomarker for the early diagnosis of hepatocellular carcinoma in Egyptian patients. Saudi journal of biological sciences 2021;28(10):5760-64.

[69] Ibrahim, N. H., Elshazly, A. S., Ghareeb, M., Elkhashab, D. M. Role of miR-15b and miR-130b as Non-Invasive Biomarkers in Hepatocellular Carcinoma. Asian Pacific journal of cancer prevention : APJCP 2025;26(7):2479 “ 2487.

[70] Istemihan, Z., Senkal, I. V., Karaca, D., Nuriyev, K., Rustamzade, A., Genc Ulucecen, S., et al. The role of GALAD score in the surveillance of hepatocellular carcinoma. Scientific reports 2025;15(1):22279.

[71] Jalli, Reza, Jafari, Seyed Hamed, Sefidbakht, Sepideh. Comparison of the Accuracy of DWI and Ultrasonography in Screening Hepatocellular Carcinoma in Patients With Chronic Liver Disease. Iranian journal of radiology : a quarterly journal published by the Iranian Radiological Society 2015;12(1):e12708.

| Study | Study details | Study population | Index test and reference standard |
|---|---|---|---|
| Jeng et al. (2014)[72] | **Study design:** 2-gate<br>**Method of recruitment:** Clinical cohort<br>**Setting:** Taiwan | **Participants, n:** 180 (HCC: 90; No HCC: 90)<br>**Age:** HCC: Median 56 (range 41, 78)<br>No HCC: Median 55 (range 41, 76)<br>**Aetiology:** HCC: HBV 61 (67.8%); HCV 23 (25.6%)<br>No HCC: HBV 56 (62.2%); HCV 25 (27.8%)<br>**Cirrhosis severity:** HCC: Child's grade A 49 (54.4%)<br>No HCC: Child's grade A 55 (61.1%) | **Index test(s):** AFP<br>**Reference standard to establish presence of HCC:** Histology (Biopsy or aspiration cytology.)<br>**Reference standard to establish absence of HCC:** Imaging (No space-occupying lesion in the liver, evidenced by abdominal sonography.) |
| Ji et al. (2021)[73] | **Study design:** 2-gate<br>**Method of recruitment:** Clinical cohort<br>**Setting:** China | **Participants, n:** 446 (HCC: 157; No HCC: 289)<br>**Age:** HCC: Mean 54 (SD 11.1)<br>No HCC: Mean 56 (SD 10.5)<br>**Aetiology:** HCC: HBV 144 (91.7%); HCV 5 (3.2%); Alcohol 0 (0.0%)<br>No HCC: HCV 79 (27.3%); Alcohol 70 (24.2%); HBV 83 (28.7%) | **Index test(s):** DCP<br>**Reference standard to establish presence of HCC:** Imaging (Chinese-related guidelines.)<br>**Reference standard to establish absence of HCC:** Not reported (null) |
| Jia et al. (2017)[74] | **Study design:** 2-gate<br>**Method of recruitment:** Clinical cohort<br>**Setting:** China | **Participants, n:** 87 (HCC: 35; No HCC: 52)<br>**Age:** HCC: Mean 62 (range 45, 80)<br>No HCC: Mean 61 (range 34, 85) | **Index test(s):** CT<br>**Reference standard to establish presence of HCC:** Histology (null)<br>**Reference standard to establish absence of HCC:** Imaging (US every 3 months.) |

72 Jeng, J. E., Tsai, M. F., Tsai, H. R., Chuang, L. Y., Lin, Z. Y., Hsieh, M. Y., et al. Urinary transforming growth factor alpha and serum alpha-fetoprotein as tumor markers of hepatocellular carcinoma. Tumor Biology 2014;35(4):3689-98.

73 Ji, Jun, Liu, Lijuan, Jiang, Feifei, Wen, Xue, Zhang, Yu, Li, Shengcong, et al. The clinical application of PIVKA-II in hepatocellular carcinoma and chronic liver diseases: A multi-center study in China. Journal of clinical laboratory analysis 2021;35(11):e24013.

74 Jia, G. S., Feng, G. L., Li, J. P., Xu, H. L., Wang, H., Cheng, Y. P., Yan, L. L. Using receiver operating characteristic curves to evaluate the diagnostic value of the combination of multislice spiral CT and alpha-fetoprotein levels for small hepatocellular carcinoma in cirrhotic patients. Hepatobiliary and Pancreatic Diseases International 2017;16(3):303-09.

| Study | Study details | Study population | Index test and reference standard |
|---|---|---|---|
| Kamal et al. (2022)[75] | **Study design:** 2-gate<br>**Method of recruitment:** Clinical cohort<br>**Setting:** Egypt | **Participants, n:** 160 (HCC: 80; No HCC: 80)<br>**Age:** HCC: Mean 60.1 (SD 8.6)<br>No HCC: Mean 58.8 (SD 8.9)<br>**Aetiology:** HCV 160 (100.0%)<br>**Cirrhosis severity:** HCC: Child's grade A 60 (75.0%)<br>No HCC: Child's grade A 12 (15.0%) | **Index test(s):** AFP; cfDNA integrity index; cfDNA integrity index and AFP<br>**Reference standard to establish presence of HCC:** Blood tests and imaging (AASLD criteria, using CT or MRI with or without AFP.)<br>**Reference standard to establish absence of HCC:** Not reported |
| Khalil et al. (2024)[76] | **Study design:** 2-gate<br>**Method of recruitment:** Clinical cohort<br>**Setting:** Egypt | **Participants, n:** 120 (HCC: 60; No HCC: 60)<br>**Age:** HCC: Mean 60.8 (SD 6.1)<br>No HCC: Mean 60.3 (SD 6.1)<br>**Aetiology:** HCC: HCV 60 (100.0%); HBV 0 (0.0%)<br>No HCC: HCV 46 (82.1%); HBV 10 (17.9%)<br>**Cirrhosis severity:** HCC: Child's grade A 44 (73.3%)<br>No HCC: Child's grade A 46 (76.7%) | **Index test(s):** AFP; Host-2 lncRNA; HOTAIR expression; HOXA-AS2 lncRNA; MALAT1 lncRNA; AFP OR MALAT1 lncRNA<br>**Reference standard to establish presence of HCC:** Imaging (triphasic CT scan or MRI for patients with HCC)<br>**Reference standard to establish absence of HCC:** Imaging (abdominal ultrasound for patients with liver cirrhosis) |
| Kim et al. (2015)[77] | **Study design:** 1-gate<br>**Method of recruitment:** Surveillance programme<br>**Setting:** South Korea | **Participants, n:** 28 (HCC: 28)<br>**Age:** Mean 51 (SD 7)<br>**Aetiology:** HBV 373 (100.0%)<br>**Cirrhosis severity:** Child's grade A 131 (35.1%) | **Index test(s):** AFP<br>**Reference standard:** Histology/imaging (All patients had US. US and CT scans every 6 months if HCC could not be excluded. CT carried out when AFP was positive but US negative. MRI was performed when no lesion detected or diagnosis inconclusive. Final diagnosis based on histology and/or imaging evidence. For controls, excluded samples collected in last 6-months before final reference standard.) |

[75] Kamal, Manal Mohamed, Abdelaziz, Ashraf Omar, El-Baz, Heba Nabil, Mohamed, Ghada Maher, Saleh, Samaa Salama, Nabeel, Mohamed Mahmoud, et al. Plasma cell-free DNA integrity index and hepatocellular carcinoma treated or not with direct-acting antivirals: A case-control study. Arab journal of gastroenterology : the official publication of the Pan-Arab Association of Gastroenterology 2022;23(1):39-44.

[76] Khalil, M. M. I. M., Seddik, R. M., Mansour, M. M., Elbasuony, H. A. A. Detection and validity of long non-coding RNAs HOST2, HOTAIR, HOXA-AS2, and MALAT1 as biomarkers for hepatocellular carcinoma. Human Gene 2024;42():201349.

[77] Kim, G. A., Seock, C. H., Park, J. W., An, J., Lee, K. S., Yang, J. E., et al. Reappraisal of serum alpha-foetoprotein as a surveillance test for hepatocellular carcinoma during entecavir treatment. Liver International 2015;35(1):232-39.

| Study | Study details | Study population | Index test and reference standard |
|---|---|---|---|
| Kim et al. (2016)[78] | **Study design:** 1-gate<br>**Method of recruitment:** Surveillance programme<br>**Setting:** South Korea | **Participants, n:** 2074 (HCC: 970; Non-HCC: 1104)<br>**Age:** HCC: Mean 53.9 (SD 6.6)<br>Non-HCC: Mean 50.9 (SD 9.3)<br>**Aetiology:** HCC: HBV 805 (83.0%); HCV 66 (6.8%); Alcohol 58 (6.0%)<br>Non-HCC: Alcohol 268 (24.3%); HBV 556 (50.4%); HCV 60 (5.4%) | **Index test(s):** AFP; DCP; AFP x DCP<br>**Reference standard:** Explant pathology following transplantation |
| Kim et al. (2017)[79] | **Study design:** 2-gate<br>**Method of recruitment:** Surveillance programme<br>**Setting:** South Korea | **Participants, n:** 132 (HCC: 75; No HCC: 57)<br>**Age:** No HCC: Mean 53.2 (range 31, 74)<br>**Aetiology:** No HCC: HBV 30 (52.6%); HCV 9 (15.8%); Alcohol 17 (29.8%) | **Index test(s):** CE-MRI; CT<br>**Reference standard to establish presence of HCC:** Explant pathology following transplantation (All patients had resection, biopsy or transplant.)<br>**Reference standard to establish absence of HCC:** Explant pathology following transplantation (All patients had total hepatectomy for liver transplantation.) |
| Kim et al. (2017)[80]<br>Secondary publication(s): Son et al. (2019); Park et al. (2020); Park et al. (2022); Heo et al. (2025) | **Study design:** 1-gate<br>**Method of recruitment:** Surveillance programme<br>**Setting:** South Korea | **Participants, n:** 43 (HCC: 43)<br>**Age:** Median 56 (IQR: 52-62)<br>**Aetiology:** Alcohol 52 (12.8%); HCV 37 (9.1%); HBV 288 (70.8%)<br>**Cirrhosis severity:** Child's grade A 320 (78.6%) | **Index test(s):** B-mode US; Contrast-enhanced MRI; AFP; AAA approach; CAA approach; noncontrast MRI; AFP or B-mode US (round 1); B-mode US OR serial AFP (double enrollment or 2 consecutive increases); B-mode US OR AFP OR serial AFP (double enrollment<br>**Reference standard:** Imaging (CT for suspcious lesions, and biopsy where possible for lesions that were still suspicious. 6-month follow-up after final screening round for all patients with CT scans. 2 year follow-up after this to check HCC status.) |

[78] Kim, Younghwan, Park, Yo-Han, Hwang, Shin, Kim, Ki-Hun, Ahn, Chul-Soo, Moon, Deok-Bog, et al. Diagnostic Role of Blood Tumor Markers in Predicting Hepatocellular Carcinoma in Liver Cirrhosis Patients Undergoing Liver Transplantation. Annals of transplantation 2016;21(c78, 9802544):660-67.

[79] Kim, Bo Ram, Lee, Jeong Min, Lee, Dong Ho, Yoon, Jeong Hee, Hur, Bo Yun, Suh, Kyung Suk, et al. Diagnostic Performance of Gadoxetic Acid-enhanced Liver MR Imaging versus Multidetector CT in the Detection of Dysplastic Nodules and Early Hepatocellular Carcinoma. Radiology 2017;285(1):134-46.

[80] Kim, So Yeon, An, Jihyun, Lim, Young-Suk, Han, Seungbong, Lee, Ji-Young, Byun, Jae Ho, et al. MRI With Liver-Specific Contrast for Surveillance of Patients With Cirrhosis at High Risk of Hepatocellular Carcinoma. JAMA oncology 2017;3(4):456-63.

| Study | Study details | Study population | Index test and reference standard |
|---|---|---|---|
| Kim et al. (2019)[81] | **Study design:** 1-gate<br>**Method of recruitment:** Surveillance programme<br>**Setting:** South Korea | **Participants, n:** 64 (HCC: 64)<br>**Age:** Mean 53.6 (SD 9)<br>**Aetiology:** Alcohol 31 (7.9%); HCV 9 (2.3%); HBV 320 (81.6%); Other / unknown 32 (8.2%)<br>**Cirrhosis severity:** Child's grade A 382 (97.4%) | **Index test(s):** AFP; US or AFP; US<br>**Reference standard:** Imaging (Median of 4.7 years of US and AFP surveillance. CT and MRI carried out when surveillance tests were positive.) |
| Krygier R. et al. (2011)[82] | **Study design:** 1-gate<br>**Method of recruitment:** Surveillance programme<br>**Setting:** Poland | **Participants, n:** 118 (HCC: 29; No HCC: 89)<br>**Age:** No HCC: >50: 56 (62.9%)<br>HCC: >50: 24 (82.8%)<br>**Aetiology:** HCC: Alcohol 7 (24.1%); HBV 13 (44.8%); HCV 20 (69.0%)<br>No HCC: HBV 18 (20.2%); HCV 57 (64.0%); Alcohol 25 (28.1%)<br>**Cirrhosis severity:** HCC: Child's grade A 8 (27.6%)<br>No HCC: Child's grade A 53 (59.6%) | **Index test(s):** AFP<br>**Reference standard:** Imaging ('In all cases US and CT or MRI were performed.') |
| Kudo et al. (2019)[83] | **Study design:** 1-gate<br>**Method of recruitment:** Surveillance programme<br>**Setting:** Japan | **Participants, n:** 54 (HCC: 54)<br>**Age:** Mean 66.3 (SD 10.7)<br>**Aetiology:** HBV 204 (32.8%); HCV 418 (67.2%)<br>**Cirrhosis severity:** Child's grade A 529 (85.0%) | **Index test(s):** CEUS; B-mode US<br>**Reference standard:** Imaging ('Surveillance consisted of B-mode US or Kupffer phase CEUS every 4 ± 1 months and CT/MRI every 8 months.') |

[81] Kim, Mi Na, Kim, Beom Kyung, Kim, Seung Up, Park, Jun Yong, Ahn, Sang Hoon, Han, Kwang-Hyub. Longitudinal assessment of alpha-fetoprotein for early detection of hepatocellular carcinoma in patients with cirrhosis. Scandinavian journal of gastroenterology 2019;54(10):1283-90.

[82] Krygier R., Stefaniuk P., Mikula T., Dusza M., Jablonowska M., Cianciara J., Wasyluk H. Usefulness of alpha-fetoprotein as circulating immune complexes (AFP-IC) in the diagnosis of hepatocellular carcinoma in cirrhotic patients. Experimental and Clinical Hepatology 2011;7(1-2):44-48.

[83] Kudo, Masatoshi, Ueshima, Kazuomi, Osaki, Yukio, Hirooka, Masashi, Imai, Yasuharu, Aso, Kazunobu, et al. B-Mode Ultrasonography versus Contrast-Enhanced Ultrasonography for Surveillance of Hepatocellular Carcinoma: A Prospective Multicenter Randomized Controlled Trial. Liver cancer 2019;8(4):271-80.

| Study | Study details | Study population | Index test and reference standard |
|---|---|---|---|
| Kurniawan et al. (2024)[84] | **Study design:** 1-gate<br>**Method of recruitment:** Clinical cohort<br>**Setting:** Indonesia | **Participants, n:** 366 (HCC: 38; No HCC: 328)<br>**Age:** HCC: Median 60.5 (range 54.8, 65.2)<br>No HCC: Median 55 (range 48, 60)<br>**Aetiology:** HCC: HBV 38 (100.0%)<br>No HCC: HBV 328 (100.0%)<br>**Cirrhosis severity:** HCC: Child's grade A 27 (71.1%)<br>No HCC: Child's grade A 254 (77.4%) | **Index test(s):** AFP<br>**Reference standard:** Imaging (3-phase CT scan or MRI +/- biopsy) |
| Lambrecht et al. (2021)[85] | **Study design:** 2-gate<br>**Method of recruitment:** Clinical cohort<br>**Setting:** Germany | **Participants, n:** 267 (HCC: 122; No HCC: 145)<br>**Age:** HCC: Median 66 (IQR: 60-72)<br>No HCC: Median 54 (IQR: 47-60)<br>**Aetiology:** HCC: Alcohol 41 (33.6%); HBV 11 (9.0%); HCV 27 (22.1%); NAFLD/NASH 26 (21.3%)<br>No HCC: HBV 11 (7.6%); HCV 18 (12.4%); Alcohol 51 (35.2%); NAFLD/NASH 34 (23.4%)<br>**Cirrhosis severity:** HCC: Child's grade A 99 (81.1%)<br>No HCC: Child's grade A 57 (39.3%) | **Index test(s):** AFP<br>**Reference standard to establish presence of HCC:** Histology/imaging (CT and/or MRI, or histology.)<br>**Reference standard to establish absence of HCC:** Imaging (CT and/or MRI.) |
| Lauenstein et al. (2007)[86] | **Study design:** 1-gate<br>**Method of recruitment:** Surveillance programme<br>**Setting:** USA | **Participants, n:** 115<br>**Age:** Mean 53.9 (range 22, 73)<br>**Aetiology:** HCV 40 (34.8%); HBV 12 (10.4%); Alcohol 30 (26.1%) | **Index test(s):** CE-MRI<br>**Reference standard:** Explant pathology following transplantation |

[84] Kurniawan, J., Djianzonie, J. A. C., Mulyana, E., Tahapary, D. L., Sulaiman, A. S., Wijaya, I. P., Nasution, S. A. Updating AFP Level in Chronic Hepatitis B to Evaluate the Risk of Hepatocellular Carcinoma Occurrence. Acta medica Indonesiana 2024;56(3):282 “ 290.

[85] Lambrecht, Joeri, Porsch-Ozcurumez, Mustafa, Best, Jan, Jost-Brinkmann, Fabian, Roderburg, Christoph, Demir, Munevver, Tacke, Frank. The APAC Score: A Novel and Highly Performant Serological Tool for Early Diagnosis of Hepatocellular Carcinoma in Patients with Liver Cirrhosis. Journal of clinical medicine 2021;10(15):n. pag..

[86] Lauenstein, Thomas C., Salman, Khalil, Morreira, Roger, Heffron, Thomas, Spivey, James R., Martinez, Enrique, Sharma, Puneet. Gadolinium-enhanced MRI for tumor surveillance before liver transplantation: center-based experience. AJR. American journal of roentgenology 2007;189(3):663-70.

| Study | Study details | Study population | Index test and reference standard |
|---|---|---|---|
| Leeming et al. (2021)[87] | **Study design:** 2-gate<br>**Method of recruitment:** Clinical cohort<br>**Setting:** Australia | **Participants, n:** 170 (HCC: 84; No HCC: 86)<br>**Age:** HCC: Mean 62.4 (SD 11.4)<br>No HCC: Mean 58.8 (SD 10)<br>**Aetiology:** HCC: NAFLD/NASH 16 (19.0%); HBV 13 (15.5%); Alcohol 11 (13.1%); HCV 40 (47.6%)<br>No HCC: HCV 43 (50.0%); NAFLD/NASH 10 (11.6%); HBV 23 (26.7%); Alcohol 7 (8.1%)<br>**Cirrhosis severity:** HCC: Child's grade A 54 (64.3%)<br>No HCC: Child's grade A 78 (90.7%) | **Index test(s):** AFP<br>**Reference standard to establish presence of HCC:** Histology/imaging (Diagnosed by CT or MRI according to EASL guidelines, or histology.)<br>**Reference standard to establish absence of HCC:** Not reported |
| Lewin et al. (2021)[88] | **Study design:** 2-gate<br>**Method of recruitment:** Clinical cohort<br>**Setting:** USA | **Participants, n:** 165 (HCC: 61; No HCC: 104)<br>**Age:** HCC: Median 64 (range 34, 89)<br>No HCC: Median 57 (range 28, 74)<br>**Aetiology:** HCC: Alcohol 15 (24.6%); HBV 4 (6.6%); HCV 18 (29.5%); NAFLD/NASH 17 (27.9%)<br>No HCC: HBV 2 (1.9%); HCV 20 (19.2%); NAFLD/NASH 33 (31.7%); Alcohol 26 (25.0%)<br>**Cirrhosis severity:** HCC: Child's grade A 41 (67.2%)<br>No HCC: Child's grade A 60 (57.7%) | **Index test(s):** AFP; mSEPT9; NGS panel; mSEPT9 + AFP; NGS panel + AFP<br>**Reference standard to establish presence of HCC:** Histology/imaging (MRI or CT imaging with a LI-RADS category of LR-5 and/or histology.)<br>**Reference standard to establish absence of HCC:** Imaging ('MRI or CT imaging performed ≤90 days prior to the date of consent or a abdominal contrast-enhanced MRI performed ≤45 days after enrollment. Patients had either no lesions or lesions with a liver imaging reporting and data system (LI-RADS) score of LR-1 or LR-2.') |

[87] Leeming, D. J., Nielsen, S. H., Vongsuvanh, R., Uchila, P., Nielsen, M. J., Reese-Petersen, A. L., et al. Endotrophin, a pro-peptide of Type VI collagen, is a biomarker of survival in cirrhotic patients with hepatocellular carcinoma. Hepatic Oncology 2021;8(2):HEP32.

[88] Lewin, Jorn, Kottwitz, Denise, Aoyama, Johanna, deVos, Theo, Garces, Jorge, Hasinger, Oliver, et al. Plasma cell free DNA methylation markers for hepatocellular carcinoma surveillance in patients with cirrhosis: a case control study. BMC gastroenterology 2021;21(1):136.

| Study | Study details | Study population | Index test and reference standard |
|---|---|---|---|
| Li et al. (2019)[89] | **Study design:** 2-gate<br>**Method of recruitment:** Clinical cohort<br>**Setting:** China | **Participants, n:** 177 (Early HCC (validation cohort): 74; No HCC (validation cohort): 103)<br>**Age:** Early HCC (validation cohort): Mean 56.2 (SD 10.7)<br>No HCC (validation cohort): Mean 52.9 (SD 11.5)<br>**Cirrhosis severity:** Early HCC (validation cohort): Child's grade A 47 (63.5%)<br>No HCC (validation cohort): Child's grade A 63 (61.2%) | **Index test(s):** Logistic regression model (validation cohort); Simple logistic regression model (validation cohort); AFP (analysis cohort); DCP (analysis cohort)<br>**Reference standard to establish presence of HCC:** Histology/imaging ('HCC diagnosis was based on pathology or typical radiologic results of HCC on two dynamic image examinations or one dynamic technique with serum AFP level ≥ 200 ng/mL'.)<br>**Reference standard to establish absence of HCC:** Not reported (null) |
| Lim et al. (2016)[90] | **Study design:** 2-gate<br>**Method of recruitment:** Clinical cohort<br>**Setting:** South Korea | **Participants, n:** 637 (HCC: 361; No HCC: 276)<br>**Age:** HCC: Median 58 (IQR: 52.0-73.0)<br>No HCC: Median 55 (IQR: 48.0-64.0)<br>**Aetiology:** HCC: HCV 33 (9.1%); HBV 275 (76.2%)<br>No HCC: HBV 192 (69.6%); HCV 37 (13.4%)<br>**Cirrhosis severity:** HCC: Child's grade A 290 (80.3%)<br>No HCC: Child's grade A 247 (89.5%) | **Index test(s):** AFP; DCP; AFP-L3; AFP or DCP; DCP or AFP-L3; AFP or AFP-L3; AFP or DCP or AFP-L3<br>**Reference standard to establish presence of HCC:** Histology/imaging ('The diagnosis of HCC was made either histologically or non-invasively, and based on the guidelines of AASLD or EASL')<br>**Reference standard to establish absence of HCC:** Imaging (Imaging surveillance. 'Patients who had been diagnosed with HCC within 12 months from the tumor marker measurement (AFP, PIVKA-II, and AFP-L3 levels) were also excluded'.) |
| Lin et al. (2016)[91] | **Study design:** 2-gate<br>**Method of recruitment:** Clinical cohort<br>**Setting:** China | **Participants, n:** 183 (HCC: 122; No HCC: 61)<br>**Age:** HCC: Mean 51.4 (SD 15.4)<br>No HCC: Mean 49.7 (SD 13) | **Index test(s):** AFP; miR-224; miR-224 + AFP<br>**Reference standard to establish presence of HCC:** Histology ('All patients with HCC were confirmed by histological examination.')<br>**Reference standard to establish absence of HCC:** Not reported (No evidence of hepatic mass within 6 months prior to enrolment.) |

[89] Li, Tao, Li, Hongguang, Wang, Aihua, Su, Xiaoyan, Zhao, Jingfang, Cui, Yi, Liu, Jun. Development And Validation Of A Simple Model For Detection Of Early Hepatocellular Carcinoma In A Liver Cirrhosis Cohort. Cancer management and research 2019;11(101512700):9379-86.

[90] Lim, Tae Seop, Kim, Do Young, Han, Kwang-Hyub, Kim, Hyon-Suk, Shin, Seung Hwan, Jung, Kyu Sik, et al. Combined use of AFP, PIVKA-II, and AFP-L3 as tumor markers enhances diagnostic accuracy for hepatocellular carcinoma in cirrhotic patients. Scandinavian journal of gastroenterology 2016;51(3):344-53.

[91] Lin, Ling, Lu, Baochun, Yu, Jianhua, Liu, Wenguang. Serum miR-224 as a biomarker for detection of hepatocellular carcinoma at early stage. Clinics and research in hepatology and gastroenterology 2016;40(4):397-404.

| Study | Study details | Study population | Index test and reference standard |
|---|---|---|---|
| Liu et al. (2007)[92] | **Study design:** 2-gate<br>**Method of recruitment:** Clinical cohort<br>**Setting:** China | **Participants, n:** 307 (HCC: 227; No HCC: 80)<br>**Age:** HCC: Mean 53.2 (SD 10.4)<br>No HCC: Mean 50.2 (SD 11.5)<br>**Aetiology:** HBV 307 (100.0%) | **Index test(s):** AFP<br>**Reference standard to establish presence of HCC:** Histology/imaging (Histology or by US and/or CT along with AFP)<br>**Reference standard to establish absence of HCC:** Not reported (') |
| Loglio et al. (2019)[93] | **Study design:** 1-gate<br>**Method of recruitment:** Surveillance programme<br>**Setting:** Italy | **Participants, n:** 35 (HCC: 35)<br>**Age:** Median 61 (range 21, 83)<br>**Aetiology:** HBV 258 (100.0%) | **Index test(s):** AFP<br>**Reference standard:** Imaging (AFP and US every 6 months. CT or MRI if US could not evaluate the whole liver or whenever AFP increased >7 ng/mL. In patients with negative CT or MRI but raised AFP, a CT or MRI was repeated every 3 months. US confirmed the absence of HCC in cirrhotic cases after 6 months.) |
| Loglio et al. (2020)[94] | **Study design:** 2-gate<br>**Method of recruitment:** Clinical cohort<br>**Setting:** Italy | **Participants, n:** 212 (HCC: 64; No HCC: 148)<br>**Age:** HCC: Median 66 (range 40, 83)<br>No HCC: Median 60 (range 22, 85)<br>**Aetiology:** HBV 212 (100.0%) | **Index test(s):** AFP; DCP; US; AFP and DCP; AFP or DCP; US or AFP or DCP<br>**Reference standard to establish presence of HCC:** Imaging (HCC developed during standard surveillance.)<br>**Reference standard to establish absence of HCC:** Imaging (US, CT or MRI at sampling. No evidence of HCC during 6 monthly US surveillance, for a period of 84 months (range 60-107) after serum collection.) |
| Lu et al. (2020)[95]<br>Secondary publication(s): Lu et al. (2024) | **Study design:** 2-gate<br>**Method of recruitment:** Clinical cohort<br>**Setting:** USA | **Participants, n:** 163 (HCC (viral): 93; No HCC: 70)<br>**Aetiology:** HCC (viral): HBV 16 (17.2%); HCV 77 (82.8%)<br>No HCC: HBV 31 (47.7%); HCV 34 (52.3%) | **Index test(s):** AFP<br>**Reference standard to establish presence of HCC:** Imaging ('HCC was diagnosed through biopsy or computed tomography/MRI using Liver Imaging Reporting and Data System (LI-RADS) classifications of LR4 and LR5.')<br>**Reference standard to establish absence of HCC:** Imaging (with 6-months imaging follow-up) ('Follow-up imaging for cirrhotic patients at least 6 months after the blood draw did not show that any had developed HCC.') |

92 Liu, Xue-En, Desmyter, Liesbeth, Gao, Chun-Fang, Laroy, Wouter, Dewaele, Sylviane, Vanhooren, Valerie, et al. N-glycomic changes in hepatocellular carcinoma patients with liver cirrhosis induced by hepatitis B virus. Hepatology (Baltimore, Md.) 2007;46(5):1426-35.

93 Loglio, Alessandro, Iavarone, Massimo, Vigano, Mauro, Orenti, Annalisa, Facchetti, Floriana, Cortinovis, Ivan, et al. Minimal increases of serum alpha-foetoprotein herald HCC detection in Caucasian HBV cirrhotic patients under long-term oral therapy. Liver international : official journal of the International Association for the Study of the Liver 2019;39(10):1964-74.

94 Loglio, Alessandro, Iavarone, Massimo, Facchetti, Floriana, Di Paolo, Dhanai, Perbellini, Riccardo, Lunghi, Giovanna, et al. The combination of PIVKA-II and AFP improves the detection accuracy for HCC in HBV caucasian cirrhotics on long-term oral therapy. Liver international : official journal of the International Association for the Study of the Liver 2020;40(8):1987-96.

95 Lu, Felix, Shah, Pir Ahmad, Rao, Abhishek, Gifford-Hollingsworth, Cynthia, Chen, Anne, Trey, Gary, et al. Liver Cancer-Specific Serine Protease Inhibitor Kazal Is a Potentially Novel Biomarker for the Early Detection of Hepatocellular Carcinoma. Clinical and translational gastroenterology 2020;11(12):e00271.

| Study | Study details | Study population | Index test and reference standard |
|---|---|---|---|
| Ma et al. (2017)[96] | **Study design:** 1-gate<br>**Method of recruitment:** Clinical cohort<br>**Setting:** China | **Participants, n:** 356 (HCC: 187; No HCC: 169)<br>**Age:** HCC: Mean 48 (SD 10.6)<br>No HCC: Mean 46 (SD 10.2)<br>**Aetiology:** HBV 356 (100.0%)<br>**Cirrhosis severity:** HCC: Child's grade A 52 (27.8%)<br>No HCC: Child's grade A 47 (27.8%) | **Index test(s):** AFP<br>**Reference standard:** Imaging (All included patients had MRI, CT and US.) |
| Malov et al. (2021)[97] | **Study design:** 2-gate<br>**Method of recruitment:** Clinical cohort<br>**Setting:** Russia | **Participants, n:** 110 (HCC: 55; No HCC: 55)<br>**Age:** HCC: Mean 59.9 (SD 4.5)<br>No HCC: Mean 57.7 (SD 10.5)<br>**Aetiology:** HCV 110 (100.0%)<br>**Cirrhosis severity:** HCC: Child's grade A 11 (20.0%)<br>No HCC: Child's grade A 13 (23.6%) | **Index test(s):** AFP; AFP-L3; DCP<br>**Reference standard to establish presence of HCC:** Imaging (EASL criteria.)<br>**Reference standard to establish absence of HCC:** Blood tests and imaging ('Follow-up for 12 months: 'clinical examination, general clinical and biochemical analyses, liver elastometry, US. If necessary, CT or MRI of the liver was performed.') |
| Maneenil et al. (2025)[98] | **Study design:** 2-gate<br>**Method of recruitment:** Clinical cohort<br>**Setting:** Thailand | **Participants, n:** 347 (HCC (early only): 84; No HCC: 263)<br>**Age:** HCC (early only): Mean 64 (SD 9.7)<br>No HCC: Mean 65.2 (SD 11.1)<br>**Aetiology:** HCC (early only): HBV 30 (34.9%); HCV 20 (23.3%); Alcohol 11 (12.8%); Autoimmune hepatitis 3 (3.5%); NAFLD/NASH 20 (23.3%)<br>No HCC: HBV 99 (37.6%); HCV 38 (14.4%); Alcohol 46 (17.5%); Autoimmune hepatitis 2 (0.8%); NAFLD/NASH 47 (17.9%) | **Index test(s):** GAAD<br>**Reference standard to establish presence of HCC:** Imaging (according to the AASLD or EASL guidelines, based on a radiological hallmark lesion (size ≥ 1cm) observed on CT or MRI)<br>**Reference standard to establish absence of HCC:** Not reported |

[96] Ma, Li-Na, Liu, Xiao-Yan, Lu, Zhen-Hui, Wu, Li-Gang, Tang, Yuan-Yuan, Luo, Xia, et al. Assessment of high-sensitivity C-reactive protein tests for the diagnosis of hepatocellular carcinoma in patients with hepatitis B-associated liver cirrhosis. Oncology letters 2017;13(5):3457-64.

[97] Malov, S. I., Malov, I. V., Kuvshinov, A. G., Marche, P. N., Decaens, T., Macek-Jilkova, Z. Search for Effective Serum Tumor Markers for Early Diagnosis of Hepatocellular Carcinoma Associated with Hepatitis C. Sovremennye tekhnologii v meditsine 2021;13(1):27-33.

[98] Maneenil, C., Sripongpun, P., Chamroonkul, N., Tantisaranon, P., Jarumanokul, R., Samaeng, M., et al. Comparative performance of the GAAD and ASAP scores in predicting early-stage hepatocellular carcinoma. Gastroenterology Report 2025;13():goaf074.

| Study | Study details | Study population | Index test and reference standard |
|---|---|---|---|
| Marrero et al. (2009)[99]<br>Secondary publication(s): Wang et al. (2016); Yang et al. (2017); Hemken et al. (2019) | **Study design:** 2-gate<br>**Method of recruitment:** Clinical cohort<br>**Setting:** USA | **Participants, n:** 836 (HCC: 419; No HCC: 417)<br>**Age:** No HCC: Mean 55 (SD 8.8)<br>HCC: Mean 61 (SD 10.3)<br>HCC: Mean 60 (SD 9.9)<br>**Aetiology:** HCC: HCV 141 (67.8%); Alcohol 23 (11.1%); HCV 95 (45.0%); HBV 34 (16.1%); HBV 31 (14.9%); Alcohol 19 (9.0%)<br>No HCC: Alcohol 50 (12.0%); HBV 21 (5.0%); HCV 250 (60.0%)<br>**Cirrhosis severity:** HCC: Child's grade A 140 (66.4%); Child's grade A 146 (70.2%)<br>No HCC: Child's grade A 233 (55.9%) | **Index test(s):** AFP; AFP-L3; DCP; AFP or DCP; Doylestown algorithm; Model based on age, gender AFP and DCP<br>**Reference standard to establish presence of HCC:** Histology/imaging ('Histologic examination or by the appropriate imaging characteristics as defined by accepted guidelines'.)<br>**Reference standard to establish absence of HCC:** Imaging ('Controls must have an US, CT, or MRI showing no evidence of hepatic mass within 6 months prior to enrollment. To assure that controls did not have HCC, all controls were assessed by AFP and an imaging test (US, CT, or MRI) 6 months after enrollment.') |
| Marsh et al. (2025)[100] | **Study design:** 1-gate<br>**Method of recruitment:** Surveillance programme<br>**Setting:** USA | **Participants, n:** 1230 (HCC: 107; No HCC: 1123)<br>**Age:** HCC: Median 62 (range 56, 66)<br>No HCC: Median 60 (range 54, 65)<br>**Aetiology:** HCC: Other / unknown 10 (9.3%); NAFLD/NASH 24 (22.4%); HBV 0 (0.0%); HCV 58 (54.2%); Alcohol 15 (14.0%)<br>No HCC: Other / unknown 165 (14.7%); HBV 29 (2.6%); NAFLD/NASH 299 (26.6%); HCV 464 (41.3%); Alcohol 166 (14.8%)<br>**Cirrhosis severity:** HCC: Child's grade A 66 (62.3%)<br>No HCC: Child's grade A 843 (75.1%) | **Index test(s):** AFP; GALAD; GAAD; DCP<br>**Reference standard:** Imaging (with 6-months imaging follow-up) (Participants had US, MRI, or CT every 6mo; specimens <6mo after last negative visit were excluded) |

[99] Marrero, Jorge A, Feng, Ziding, Wang, Yinghui, Nguyen, Mindie H, Befeler, Alex S, Roberts, Lewis R, et al. Alpha-fetoprotein, des-gamma carboxyprothrombin, and lectin-bound alpha-fetoprotein in early hepatocellular carcinoma. Gastroenterology 2009;137(1):110-8.

[100] Marsh, T. L., Parikh, N. D., Roberts, L. R., Schwartz, M. E., Nguyen, M. H., Befeler, A., et al. A Phase 3 Biomarker Validation of GALAD for the Detection of Hepatocellular Carcinoma in Cirrhosis. Gastroenterology 2025;168(2):316 “ 326.e6.

| Study | Study details | Study population | Index test and reference standard |
|---|---|---|---|
| Mittal et al. (2011)[101] | **Study design:** 2-gate<br>**Method of recruitment:** Clinical cohort<br>**Setting:** Nepal | **Participants, n:** 828 (HCC: 348; No HCC: 480) | **Index test(s):** AFP<br>**Reference standard to establish presence of HCC:** Blood tests and imaging (Diagnosis based on the presence of cirrhosis and a lesion >2 cm on two or more imaging tests (including US, CT, MRI and angiography); or presence of cirrhosis and a lesion >2 cm on one imaging test (including CT, MRI, and angiography) and AFP >100 ng/ml.)<br>**Reference standard to establish absence of HCC:** Not reported |
| Mohamed et al. (2020)[102] | **Study design:** 2-gate<br>**Method of recruitment:** Clinical cohort<br>**Setting:** Egypt | **Participants, n:** 160 (HCC: 80; No HCC: 80)<br>**Age:** HCC: Mean 52 (SD 9.8)<br>No HCC: Mean 49.3 (SD 7.7)<br>**Aetiology:** HCC: HCV 80 (100.0%)<br>No HCC: HCV 80 (100.0%) | **Index test(s):** AFP; MiR-155 (RQ); MiR-665 (RQ)<br>**Reference standard to establish presence of HCC:** Not reported<br>**Reference standard to establish absence of HCC:** Not reported |
| Mohamed et al. (2022)[103] | **Study design:** 2-gate<br>**Method of recruitment:** Clinical cohort<br>**Setting:** Egypt | **Participants, n:** 52<br>**Aetiology:** HCV 52 (100.0%)<br>**Cirrhosis severity:** Child's grade A 32 (61.5%) | **Index test(s):** AFP<br>**Reference standard to establish presence of HCC:** Imaging (Diagnosed according to the AASLD practice guidelines.)<br>**Reference standard to establish absence of HCC:** Imaging (All patients had US.) |
| Monzawa et al. (2007)[104] | **Study design:** 2-gate<br>**Method of recruitment:** Clinical cohort<br>**Setting:** Japan | **Participants, n:** 113 (HCC: 48; No HCC: 65)<br>**Age:** HCC: Mean 66 (range 49, 85)<br>No HCC: Mean 61 (range 34, 85) | **Index test(s):** CT<br>**Reference standard to establish presence of HCC:** Histology/imaging (US and CT were performed to confirm the presence of HCCs, followed by biopsies of the detected tumours.)<br>**Reference standard to establish absence of HCC:** Not reported ('The absence of HCCs was confirmed by follow-up of 2 or more years.') |

[101] Mittal, Ankush, Sathian, Brijesh, Chandrashekharan, Nishida, Farooqui, Shamim Mohammad. Diagnostic significance of alpha fetoprotein in carcinomas of liver and biliary tract - a comparative study from the western region of Nepal. Asian Pacific journal of cancer prevention : APJCP 2011;12(12):3475-78.

[102] Mohamed, Amal Ahmed, Omar, Abdellah Abosrie Ali, El-Awady, Rehab R, Hassan, Sally Mohamed Aboelsayed, Eitah, Waleed Mohamed Soliman, Ahmed, Rehab, et al. MiR-155 and MiR-665 Role as Potential Non-invasive Biomarkers for Hepatocellular Carcinoma in Egyptian Patients with Chronic Hepatitis C Virus Infection. Journal of translational internal medicine 2020;8(1):32-40.

[103] Mohamed, S. Y., Esmaiel, A. E., Shabana, M. A. Assessment of Plasma Vitronectin as Diagnostic and Prognostic Marker of Hepatocellular Carcinoma in Patients with Hepatitis C Virus Cirrhosis. Gastroenterology Insights 2022;13(1):13.

[104] Monzawa, Shuichi, Ichikawa, Tomoaki, Nakajima, Hiroto, Kitanaka, Yuki, Omata, Kosaku. Dynamic CT for detecting small hepatocellular carcinoma: usefulness of delayed phase imaging. AJR. American journal of roentgenology 2007;188(1):147-53.

| Study | Study details | Study population | Index test and reference standard |
| --- | --- | --- | --- |
| Mossad et al. (2014)[105] | **Study design:** 2-gate<br>**Method of recruitment:** Clinical cohort<br>**Setting:** Egypt | **Participants, n:** 70 (HCC: 40; No HCC: 30)<br>**Age:** HCC: Mean 57.3 (SD 6.3)<br>No HCC: Mean 57.8 (SD 7.8)<br>**Aetiology:** HCC: HCV 33 (82.5%); HBV 8 (20.0%)<br>No HCC: Viral 30 (100.0%)<br>**Cirrhosis severity:** HCC: Child's grade A 0 (0.0%)<br>No HCC: Child's grade A 0 (0.0%) | **Index test(s):** AFP<br>**Reference standard to establish presence of HCC:** Imaging (AASLD guidelines.)<br>**Reference standard to establish absence of HCC:** Imaging (Regular liver US screening.) |
| Mukozu et al. (2013)[106] | **Study design:** 2-gate<br>**Method of recruitment:** Clinical cohort<br>**Setting:** Japan | **Participants, n:** 87 (HCC: 59; No HCC: 28)<br>**Age:** HCC: Mean 65.4 (SD 6)<br>HCC: Mean 69.8 (SD 5)<br>No HCC: Mean 61.4 (SD 8)<br>**Aetiology:** HCV 87 (100.0%)<br>**Cirrhosis severity:** HCC: Child's grade A 30 (62.5%); Child's grade A 9 (81.8%)<br>No HCC: Child's grade A 16 (57.1%) | **Index test(s):** AFP; AFP-L3; DCP; AFP or AFP-L3 or DCP<br>**Reference standard to establish presence of HCC:** Imaging (Clinical criteria and US, CT or MRI)<br>**Reference standard to establish absence of HCC:** Not reported |
| Nafeh et al. (2024)[107] | **Study design:** 1-gate<br>**Method of recruitment:** Surveillance programme<br>**Setting:** Egypt | **Participants, n:** 45 (HCC: 15; No HCC: 30)<br>**Age:** HCC: Mean 51.7 (SD 6.4)<br>No HCC: Mean 49.6 (SD 6.9)<br>**Aetiology:** HCC: HCV 15 (100.0%)<br>No HCC: HCV 30 (100.0%)<br>**Cirrhosis severity:** HCC: Child's grade A 15 (100.0%)<br>No HCC: Child's grade A 30 (100.0%) | **Index test(s):** miR-122<br>**Reference standard:** Not reported |

[105] Mossad, Nehad A., Mahmoud, Enas H., Osman, Enas A., Mahmoud, Sherif H. Evaluation of squamous cell carcinoma antigen-immunoglobulin M complex (SCCA-IGM) and alpha-L-fucosidase (AFU) as novel diagnostic biomarkers for hepatocellular carcinoma. Tumour biology : the journal of the International Society for Oncodevelopmental Biology and Medicine 2014;35(11):11559-64.

[106] Mukozu, Takanori, Nagai, Hidenari, Matsui, Daigo, Kanekawa, Takenori. Serum VEGF as a tumor marker in patients with HCV-related liver cirrhosis and hepatocellular carcinoma. Anticancer research 2013;33(3):1013-21.

[107] Nafeh, H. M., Rafat, A., Hassany, S., Mahmoud, A. CIRCULATING MIRNA-122, MIRNA-155, AND MIRNA- 200 AS PREDICTORS FOR HEPATOCELLULAR CARCINOMA OCCURRENCE IN EGYPTIAN PATIENTS WITH HCV RELATED LIVER CIRRHOSIS TREATED WITH DIRECT ACTING ANTIVIRAL DRUGS. Bulletin of Pharmaceutical Sciences. Assiut 2024;47(1):519 “ 532.

| Study | Study details | Study population | Index test and reference standard |
|---|---|---|---|
| Nardone et al. (2025)[108] | **Study design:** 2-gate<br>**Method of recruitment:** Clinical cohort<br>**Setting:** International | **Participants, n:** 90 (HCC: 60; No HCC: 30)<br>**Age:** HCC: Mean 66.4 (SD 8.3)<br>HCC: Mean 67 (SD 8.2)<br>HCC: Mean 65.9 (SD 8.4)<br>No HCC: Mean 53 (SD 12.3)<br>**Aetiology:** HCC: NAFLD/NASH 0 (0.0%); Autoimmune hepatitis 0 (0.0%); HBV 4 (13.3%); HCV 7 (23.3%); Other / unknown 7 (23.3%); Other / unknown 11 (18.3%); Autoimmune hepatitis 0 (0.0%); Alcohol 12 (40.0%); HBV 5 (8.3%); HCV 14 (23.3%); Alcohol 30 (50.0%); NAFLD/NASH 0 (0.0%); Alcohol 18 (60.0%); NAFLD/NASH 0 (0.0%); Autoimmune hepatitis 0 (0.0%); HBV 1 (3.3%); HCV 7 (23.3%); Other / unknown 4 (13.3%)<br>No HCC: Alcohol 17 (56.7%); NAFLD/NASH 2 (6.7%); Autoimmune hepatitis 1 (3.3%); HBV 2 (6.7%); HCV 3 (10.0%); Other / unknown 2 (6.7%)<br>**Cirrhosis severity:** HCC: Child's grade A 24 (80.0%); Child's grade B-C 6 (20.0%); Child's grade A 49 (81.7%); Child's grade B-C 11 (18.3%); Child's grade A 25 (83.3%); Child's grade B-C 5 (16.7%)<br>No HCC: Child's grade A 13 (43.3%); Child's grade B-C 17 (56.7%) | **Index test(s):** AFP<br>**Reference standard to establish presence of HCC:** Not reported<br>**Reference standard to establish absence of HCC:** Not reported |

[108] Nardone, L., Alunni-Fabbroni, M., Schinner, R., Weber, S., Mayerle, J., Schiffer, E., et al. Nuclear magnetic resonance-based lipid metabolite profiles for differentiation of patients with liver cirrhosis with and without hepatocellular carcinoma. Journal of Cancer Research and Clinical Oncology 2025;151(4):131.

| Study | Study details | Study population | Index test and reference standard |
|---|---|---|---|
| Nomair et al. (2019)[109] | **Study design:** 2-gate<br>**Method of recruitment:** Clinical cohort<br>**Setting:** Egypt | **Participants, n:** 44 (HCC: 22; No HCC: 22)<br>**Age:** HCC: Mean 60.1 (SD 5.1)<br>No HCC: Mean 58.6 (SD 7.8)<br>**Aetiology:** HCV 44 (100.0%)<br>**Cirrhosis severity:** HCC: Child's grade A 0 (0.0%)<br>No HCC: Child's grade A 2 (9.1%) | **Index test(s):** AFP<br>**Reference standard to establish presence of HCC:** Blood tests and imaging (Divided into groups using 'biochemical and radiological profile.' Diagnosed HCC using AASLD guidelines.)<br>**Reference standard to establish absence of HCC:** Imaging (Liver cirrhosis was diagnosed based on clinical, laboratory and imaging criteria (coarse echo pattern of the liver on ultrasound), with reporting of the presence/ absence of portal hypertension and splenomegaly.) |
| Oksuz et al. (2016)[110] | **Study design:** 2-gate<br>**Method of recruitment:** Clinical cohort<br>**Setting:** Turkey | **Participants, n:** 95 (HCC: 38; No HCC: 57)<br>**Age:** HCC: Mean 57.3 (SD 9.2)<br>No HCC: Mean 55.3 (SD 8.1)<br>**Cirrhosis severity:** HCC: Child's grade A 14 (36.8%)<br>No HCC: Child's grade A 29 (50.9%) | **Index test(s):** AFP<br>**Reference standard to establish presence of HCC:** Histology/imaging (Either AFP >400 ng/ml and US or CT. Supported by biopsy.)<br>**Reference standard to establish absence of HCC:** Not reported |
| Omran et al. (2022)[111] | **Study design:** 2-gate<br>**Method of recruitment:** Clinical cohort<br>**Setting:** Egypt | **Participants, n:** 166 (HCC: 116; No HCC: 50)<br>**Age:** No HCC: Median 49.4 (IQR: 7.5)<br>HCC: Median 60.4<br>**Aetiology:** HCV 166 (100.0%) | **Index test(s):** AFP<br>**Reference standard to establish presence of HCC:** Imaging (EASL and AASLD guidelines.)<br>**Reference standard to establish absence of HCC:** Not reported |

109 Nomair, Azhar Mohamed, Madkour, Marwa Ahmed, Shamseya, Mohammed Mohammed, Elsheredy, Heba Gaber. Profiling of plasma metabolomics in patients with hepatitis C-related liver cirrhosis and hepatocellular carcinoma. Clinical and experimental hepatology 2019;5(4):317-26.

110 Oksuz, E., Oksuz, M., Egesel, T., Ozgur, G. Plasma cobalamin level as a considered tumor marker for hepatocellular carcinoma. Eastern Journal of Medicine 2016;21(3):113-18.

111 Omran, Mohamed, Fouda, Manar, Abdelwahab, Abdelwahab Osama, Nabeel, Mohamed Mahmoud, Abdelaziz, Ashraf Omar, Omran, Dalia. P53 is a risk factor of de-novo hepatitis C-related hepatocellular carcinoma treated with direct-acting antivirals: a case-control study. European journal of gastroenterology & hepatology 2022;34(2):220-26.

| Study | Study details | Study population | Index test and reference standard |
|---|---|---|---|
| Oussalah et al. (2018)[112] | **Study design:** 2-gate<br>**Method of recruitment:** Clinical cohort<br>**Setting:** Germany | **Participants, n:** 103 (HCC: 47; No HCC: 56)<br>**Age:** HCC: Median 67 (IQR: 61-75)<br>No HCC: Median 63 (Replication study. IQR: 60-69)<br>**Aetiology:** HCC: Alcohol 39 (83.0%)<br>No HCC: Alcohol 38 (67.9%) | **Index test(s):** mSEPT9<br>**Reference standard to establish presence of HCC:** Imaging ('A diagnosis of HCC was confirmed using an abdominal contrast-enhanced CT scan and systematically discussed in multidisciplinary consultation meetings. The adjudicating physicians (VL, initial study; AZ, replication study) were blinded to patients' results associated with the mSEPT9 test.')<br>**Reference standard to establish absence of HCC:** Imaging (with 6-months imaging follow-up) ('Screening abdominal ultrasound showed no evidence of HCC at the time of blood sampling for the mSEPT9 test and on the next visit six months later.') |
| Oussalah et al. (2018)[113] | **Study design:** 1-gate<br>**Method of recruitment:** Clinical cohort<br>**Setting:** France | **Participants, n:** 186 (HCC: 51; No HCC: 135)<br>**Age:** HCC: Median 69 (Initial study. IQR: 61-75)<br>No HCC: Median 57 (Initial study. IQR: 49-66)<br>**Aetiology:** HCC: HBV 2 (3.9%); HCV 15 (29.4%); NAFLD/NASH 17 (33.3%); Alcohol 30 (58.8%)<br>No HCC: HCV 55 (40.7%); Alcohol 20 (14.8%); HBV 27 (20.0%); NAFLD/NASH 23 (17.0%) | **Index test(s):** mSEPT9<br>**Reference standard:** Imaging (with 6-months imaging follow-up) ('A diagnosis of HCC was confirmed using an abdominal contrast-enhanced CT scan and systematically discussed in multidisciplinary consultation meetings. The adjudicating physicians (VL, initial study; AZ, replication study) were blinded to patients' results associated with the mSEPT9 test.' 'Screening abdominal ultrasound showed no evidence of HCC at the time of blood sampling for the mSEPT9 test and on the next visit six months later.') |
| Ozkan et al. (2011)[114] | **Study design:** 2-gate<br>**Method of recruitment:** Clinical cohort<br>**Setting:** Turkey | **Participants, n:** 130 (HCC: 75; No HCC: 55)<br>**Age:** HCC: Mean 63 (SD 9.9)<br>No HCC: Mean 58 (SD 12.7)<br>**Aetiology:** HCC: HBV 44 (58.7%); HCV 19 (25.3%); Alcohol 2 (2.7%)<br>No HCC: HBV 22 (40.0%); HCV 10 (18.2%); Alcohol 6 (10.9%) | **Index test(s):** AFP<br>**Reference standard to establish presence of HCC:** Histology/imaging (Histology or two imaging modalities (US, MRI or CT).)<br>**Reference standard to establish absence of HCC:** Not reported (null) |

[112] Oussalah, Abderrahim, Rischer, Susann, Bensenane, Mouni, Conroy, Guillaume, Filhine-Tresarrieu, Pierre, Debard, Renee, et al. Plasma mSEPT9: A Novel Circulating Cell-free DNA-Based Epigenetic Biomarker to Diagnose Hepatocellular Carcinoma. EBioMedicine 2018;30():138-47.

[113] Oussalah, Abderrahim, Rischer, Susann, Bensenane, Mouni, Conroy, Guillaume, Filhine-Tresarrieu, Pierre, Debard, Renee, et al. Oussalah REPLICATION stuy. EBioMedicine 2018;30():138-47.

[114] Ozkan, Hasan, Erdal, Harun, Kocak, Erdem, Tutkak, Huseyin, Karaeren, Zihni, Yakut, Mustafa. Diagnostic and prognostic role of serum glypican 3 in patients with hepatocellular carcinoma. Journal of clinical laboratory analysis 2011;25(5):350-3.

| Study | Study details | Study population | Index test and reference standard |
|---|---|---|---|
| Pan et al. (2024)[115] | **Study design:** 2-gate<br>**Method of recruitment:** Clinical cohort<br>**Setting:** China | **Participants, n:** 68 (HCC: 37; No HCC: 31)<br>**Age:** HCC: Mean 57.4 (SD 7.7)<br>No HCC: Mean 56.2 (SD 10.8)<br>**Aetiology:** HCC: HBV 37 (100.0%)<br>No HCC: HBV 31 (100.0%) | **Index test(s):** AFP; HOTAIR expression<br>**Reference standard to establish presence of HCC:** Not reported<br>**Reference standard to establish absence of HCC:** Not reported |
| Park et al. (2019)[116] | **Study design:** 1-gate<br>**Method of recruitment:** Surveillance programme<br>**Setting:** South Korea | **Participants, n:** 524<br>**Age:** Mean 54 (SD 9)<br>**Aetiology:** HCV 24 (4.6%); Alcohol 4 (0.8%); HBV 500 (95.4%)<br>**Cirrhosis severity:** Child's grade A 297 (56.7%) | **Index test(s):** B-mode US; Perfluorobutane-enhanced US<br>**Reference standard:** Histology/imaging (CT or MRI for suspicious lesions. Other patients had 6-month follow-up surveillance. CT, MRI or histology for final HCC diagnosis.) |
| Pascut et al. (2019)[117] | **Study design:** 2-gate<br>**Method of recruitment:** Clinical cohort<br>**Setting:** Italy | **Participants, n:** 30<br>**Age:** Mean 55 (SD 11.1)<br>**Aetiology:** HCV 30 (100.0%)<br>**Cirrhosis severity:** Child's grade A 22 (73.3%) | **Index test(s):** miR-3197; miR-1228-3p<br>**Reference standard to establish presence of HCC:** Not reported<br>**Reference standard to establish absence of HCC:** Not reported |
| Patel et al. (2025)[118] | **Study design:** 2-gate<br>**Method of recruitment:** Clinical cohort<br>**Setting:** USA | **Participants, n:** 503 (HCC: 226; No HCC: 277)<br>**Age:** HCC: Mean 60.9 (range 56.2, 67.2)<br>No HCC: Mean 60.9 (range 56.2, 67.2)<br>**Aetiology:** HCC: HCV 140 (61.9%); HBV 6 (2.7%); Alcohol 43 (19.0%); NAFLD/NASH 29 (12.8%); Other / unknown 8 (3.5%)<br>No HCC: HCV 174 (62.8%); HBV 4 (1.4%); Alcohol 51 (18.4%); NAFLD/NASH 39 (14.1%); Other / unknown 9 (3.2%)<br>**Cirrhosis severity:** HCC: Child's grade A 110 (48.7%)<br>No HCC: Child's grade A 143 (51.6%) | **Index test(s):** B-mode US alone; AFP or B-mode US<br>**Reference standard to establish presence of HCC:** Unclear (HCC was diagnosed using American Association for the Study of Liver Diseases criteria)<br>**Reference standard to establish absence of HCC:** Imaging (with 6-months imaging follow-up) (control subjects were patients with cirrhosis, without evidence of HCC for >=1 year following ultrasound) |

[115] Pan, Y., Cai, M., Zhang, F., Liu, X., Li, M., Xie, B. Diagnostic Significance of Serum Long Noncoding HOX Antisense Intergenic Ribonucleic Acid in Patients with Hepatitis B Virus Related Hepatocellular Carcinoma. Turkish Journal of Gastroenterology 2024;35(5):391 “ 397.

[116] Park, Ji Hoon, Park, Mi-Suk, Lee, So Jung, Jeong, Woo Kyoung, Lee, Jae Young, Park, Min Jung, et al. Contrast-enhanced US with Perfluorobutane for Hepatocellular Carcinoma Surveillance: A Multicenter Diagnostic Trial (SCAN). Radiology 2019;292(3):638-46.

[117] Pascut, Devis, Cavalletto, Luisa, Pratama, Muhammad Yogi, Bresolin, Silvia, Trentin, Luca, Basso, Giuseppe, et al. Serum miRNA Are Promising Biomarkers for the Detection of Early Hepatocellular Carcinoma after Treatment with Direct-Acting Antivirals. Cancers 2019;11(11):n. pag..

[118] Patel, A., Daher, D., Yekkaluri, S., Ng, M., Rich, N. E. Addition of AFP Improves Sensitivity of Ultrasound for Early-Stage HCC Detection in Patients With Cirrhosis. Clinical Gastroenterology and Hepatology 2025;():n. pag..

| Study | Study details | Study population | Index test and reference standard |
|---|---|---|---|
| Pinero et al. (2015)[119] | **Study design:** 1-gate<br>**Method of recruitment:** Surveillance programme<br>**Setting:** Argentina | **Participants, n:** 643<br>**Age:** Mean 59 (SD 8)<br>**Aetiology:** HCV 53 (8.2%); HBV 15 (2.3%); Alcohol 25 (3.9%); NAFLD/NASH 11 (1.7%)<br>**Cirrhosis severity:** Child's grade A 20 (3.1%) | **Index test(s):** US<br>**Reference standard:** Explant pathology following transplantation |
| Pocha et al. (2013)[120] | **Study design:** 1-gate<br>**Method of recruitment:** Surveillance programme<br>**Setting:** USA | **Participants, n:** 17 (HCC: 17)<br>**Age:** Mean 59.2 (SD 5.3)<br>**Aetiology:** HBV 2 (2.4%); HCV 72 (86.7%); Alcohol 6 (7.2%); NAFLD/NASH 3 (3.6%); HBV 1 (1.2%); HCV 70 (87.5%); Alcohol 6 (7.5%); NAFLD/NASH 2 (2.5%); HCV 142 (87.1%); HBV 3 (1.8%); Alcohol 12 (7.4%); NAFLD/NASH 5 (3.1%)<br>**Participants, n:** 17 (HCC: 17)<br>**Age:** Mean 59.5 (SD 5.3)<br>**Aetiology:** HBV 2 (2.4%); HCV 72 (86.7%); Alcohol 6 (7.2%); NAFLD/NASH 3 (3.6%); HBV 1 (1.2%); HCV 70 (87.5%); Alcohol 6 (7.5%); NAFLD/NASH 2 (2.5%); HCV 142 (87.1%); HBV 3 (1.8%); Alcohol 12 (7.4%); NAFLD/NASH 5 (3.1%)<br>**Participants, n:** 17 (HCC: 17)<br>**Age:** Mean 59.3 (SD 5.3)<br>**Aetiology:** HBV 2 (2.4%); HCV 72 (86.7%); Alcohol 6 (7.2%); NAFLD/NASH 3 (3.6%); HBV 1 (1.2%); HCV 70 (87.5%); Alcohol 6 (7.5%); NAFLD/NASH 2 (2.5%); HCV 142 (87.1%); HBV 3 (1.8%); Alcohol 12 (7.4%); NAFLD/NASH 5 (3.1%) | **Index test(s):** US; CT; AFP<br>**Reference standard:** Histology/imaging (AFP used in combination with CT (index test). Hepatic lesions identified by US or CT (index tests) were further evaluated by biopsy or MRI.) |

[119] Pinero, Federico, Marciano, Sebastian, Anders, Margarita, Orozco, Federico, Zerega, Alina, Cabrera, Carla Ramirez, et al. Screening for liver cancer during transplant waiting list: a multicenter study from South America. European journal of gastroenterology & hepatology 2015;27(3):355-60.

[120] Pocha, C., Dieperink, E., McMaken, K. A., Knott, A., Thuras, P. Surveillance for hepatocellular cancer with ultrasonography vs. computed tomography - A randomised study. Alimentary Pharmacology and Therapeutics 2013;38(3):303-12.

| Study | Study details | Study population | Index test and reference standard |
|---|---|---|---|
| Qian et al. (2022)[121] | **Study design:** 1-gate<br>**Method of recruitment:** Surveillance programme<br>**Setting:** China | **Participants, n:** 20 (HCC: 20)<br>**Age:** Mean 52 (range 31, 75)<br>**Aetiology:** HBV 623 (84.0%); NAFLD/NASH 97 (13.1%); HCV 9 (1.2%); Alcohol 63 (8.5%) | **Index test(s):** AFP; Doylestown algorithm<br>**Reference standard:** Imaging ('HCC surveillance was performed by radiographic imaging (CT and/or MRI) and testing for tumor markers every 6 months. The diagnosis of HCC was made based on radiological imaging.') |
| Raafat et al. (2020)[122] | **Study design:** 2-gate<br>**Method of recruitment:** Clinical cohort<br>**Setting:** Egypt | **Participants, n:** 198 (HCC: 95; No HCC: 103)<br>**Age:** HCC: Mean 57.6 (SD 14.2)<br>No HCC: Mean 54.2 (SD 17.1)<br>**Cirrhosis severity:** HCC: Child's grade A 24 (25.3%)<br>No HCC: Child's grade A 39 (37.9%) | **Index test(s):** miR-17-5p<br>**Reference standard to establish presence of HCC:** Histology/imaging (US and/or CT or MRI, AFP and histology.)<br>**Reference standard to establish absence of HCC:** Not reported |
| Rashad et al. (2018)[123] | **Study design:** 2-gate<br>**Method of recruitment:** Clinical cohort<br>**Setting:** Egypt | **Participants, n:** 90 (HCC: 51; No HCC: 39)<br>**Age:** HCC: Mean 45.9 (SD 6.4)<br>No HCC: Mean 48.3 (SD 5)<br>**Aetiology:** HCV 90 (100.0%) | **Index test(s):** miRNA-27a; miRNA-18b; miRNA-27a and miRNA-18b<br>**Reference standard to establish presence of HCC:** Imaging (AASLD guidelines.)<br>**Reference standard to establish absence of HCC:** Not reported (null) |
| Rashad et al. (2024)[124] | **Study design:** 2-gate<br>**Method of recruitment:** Clinical cohort<br>**Setting:** Egypt | **Participants, n:** 76 (HCC: 38; No HCC: 38)<br>**Age:** HCC: Mean 64 (SD 8.6)<br>No HCC: Mean 62.4 (SD 8.5)<br>**Cirrhosis severity:** HCC: Child's grade A 19 (50.0%)<br>No HCC: Child's grade A 27 (71.1%) | **Index test(s):** AFP; DCP (ng/ml)<br>**Reference standard to establish presence of HCC:** Imaging (Triphasic CT or Dynamic MRI was done to confirm the diagnosis of HCC based on contrast uptake and washout.)<br>**Reference standard to establish absence of HCC:** Not reported |

[121] Qian, Yunsong, Li, Linhong, Ma, Li, Ji, Rengbin, Ying, Sheng, Zhou, Juhong, Fu, Liyun. Validation of the hepatocellular carcinoma early detection screening algorithm Doylestown and aMAP in a cohort of Chinese with cirrhosis. Journal of clinical laboratory analysis 2022;36(4):e24296.

[122] Raafat, N., Zaher, T. I., Etewa, R. L., El-gerby, K. M. Heat shock protein-27 and MiR-17-5p are novel diagnostic and prognostic biomarkers for hepatocellular carcinoma in Egyptian patients. Gene Reports 2020;21():100822.

[123] Rashad, Nearmeen M., El-Shal, Amal S., Shalaby, Sally M. Serum miRNA-27a and miRNA-18b as potential predictive biomarkers of hepatitis C virus-associated hepatocellular carcinoma. Molecular and cellular biochemistry 2018;447(1-2):125-36.

[124] Rashad, A. H., Oraby, M., Abdelaal, A. A., Salem, A. E., Maher, R. M. Potential Diagnostic Role of Serum Fibroblast Growth Factor-19 in Hepatocellular Carcinoma. Asian Pacific journal of cancer prevention : APJCP 2024;25(9):3097 “ 3104.

| Study | Study details | Study population | Index test and reference standard |
|---|---|---|---|
| Ricco et al. (2018)[125] | **Study design:** 1-gate<br>**Method of recruitment:** Surveillance programme<br>**Setting:** Italy | **Participants, n:** 388 (HCC: 258; No HCC: 130)<br>**Age:** HCC: Median 66.9 (range 37.2, 87.9)<br>No HCC: Median 60.6 (range 33.8, 85.3)<br>**Aetiology:** HCC: HBV 48 (18.6%); HCV 126 (48.8%); Non-viral 84 (32.6%)<br>No HCC: HBV 35 (26.9%); HCV 56 (43.1%); Non-viral 39 (30.0%) | **Index test(s):** AFP; DCP; AFP or DCP<br>**Reference standard:** Imaging (Diagnosed HCC by EASL guidelines. Cirrhosis patients had US surveillance for at least 12 months, with no evidence of HCC.) |
| Rui et al. (2025)[126] | **Study design:** 2-gate<br>**Method of recruitment:** Clinical cohort<br>**Setting:** China | **Participants, n:** 211 (HCC: 131; No HCC: 80)<br>**Age:** HCC: Median 58 (range 50, 65)<br>HCC: Median 60 (range 52, 69)<br>No HCC: Median 53 (range 47, 60)<br>No HCC: Median 57 (range 49, 65)<br>**Aetiology:** HCC: HBV 43 (100.0%); HBV 88 (100.0%)<br>No HCC: HBV 39 (100.0%); HBV 41 (100.0%)<br>**Cirrhosis severity:** HCC: Child's grade B-C 0 (0.0%); Child's grade B-C 88 (100.0%); Child's grade A 43 (100.0%); Child's grade A 0 (0.0%)<br>No HCC: Child's grade A 39 (100.0%); Child's grade B-C 0 (0.0%); Child's grade A 0 (0.0%); Child's grade B-C 41 (100.0%) | **Index test(s):** AFP; DCP (mAU/mL)<br>**Reference standard to establish presence of HCC:** Histology<br>**Reference standard to establish absence of HCC:** Histology of resected or biopsied lesions (NB the study only includes patients with pathological data) |
| Saad et al. (2020)[127] | **Study design:** 2-gate<br>**Method of recruitment:** Clinical cohort<br>**Setting:** Egypt | **Participants, n:** 50 (HCC: 30; No HCC: 20)<br>**Age:** HCC: (range 46, 65)<br>No HCC: (range 38, 61)<br>**Aetiology:** HCV 50 (100.0%)<br>**Cirrhosis severity:** HCC: Child's grade A-B 30 (100.0%) | **Index test(s):** AFP<br>**Reference standard to establish presence of HCC:** Blood tests and imaging (US, CT, or MRI and AFP.)<br>**Reference standard to establish absence of HCC:** Imaging (All patients had US.) |

[125] Ricco, Gabriele, Cavallone, Daniela, Cosma, Chiara, Caviglia, Gian Paolo, Oliveri, Filippo, Biasiolo, Alessandra, et al. Impact of etiology of chronic liver disease on hepatocellular carcinoma biomarkers. Cancer biomarkers : section A of Disease markers 2018;21(3):603-12.

[126] Rui, H., Yueqin, N., Wei, W., Bangtao, L. Combining AFP, PIVKA-II, and GP73 has diagnostic utility for hepatitis B-associated hepatocellular carcinoma and is consistent with liver pathology results. Scientific reports 2025;15(1):14869.

[127] Saad, Zienab M., Fouad, Yasser, Ali, Lamia H. Clinical Significance of Annexin A4 as a Biomarker in the Early Diagnosis of Hepatocellular Carcinoma. Asian Pacific journal of cancer prevention : APJCP 2020;21(9):2661-65.

| Study | Study details | Study population | Index test and reference standard |
|---|---|---|---|
| Sadeghi et al. (2015)[128] | **Study design:** 1-gate<br>**Method of recruitment:** Surveillance programme<br>**Setting:** Germany | **Participants, n:** 139 (HCC: 61; No HCC: 78)<br>**Age:** HCC: Mean 56.5 (SD 8.6)<br>No HCC: Mean 50.2 (SD 11.6)<br>**Aetiology:** HCC: Hepatitis 25 (41.0%); Alcohol 19 (31.1%)<br>No HCC: Hepatitis 15 (19.2%); Alcohol 22 (28.2%)<br>**Cirrhosis severity:** HCC: Child's grade A 25 (41.0%)<br>No HCC: Child's grade A 24 (30.8%) | **Index test(s):** AFP<br>**Reference standard:** Explant pathology following transplantation |
| Sanai et al. (2010)[129] | **Study design:** 2-gate<br>**Method of recruitment:** Clinical cohort<br>**Setting:** Saudi Arabia | **Participants, n:** 405 (HCC: 206; No HCC: 199)<br>**Age:** HCC: Median 66 (range 24, 93)<br>No HCC: Median 58 (range 11, 93)<br>**Aetiology:** HCC: HCV 105 (51.0%); HBV 69 (33.5%)<br>**Cirrhosis severity:** HCC: Child's grade A 58 (28.2%) | **Index test(s):** AFP<br>**Reference standard to establish presence of HCC:** Imaging (CT or MRI)<br>**Reference standard to establish absence of HCC:** Imaging ('US, CT, and/or MRI, one of which must have been performed at least 6 months following the measurement of AFP.') |
| Seif El Dahan et al. (2025)[130]<br>Secondary publication(s): Seif El Dahan et al. (2025) | **Study design:** 2-gate<br>**Method of recruitment:** Clinical cohort<br>**Setting:** USA | **Participants, n:** 648 (HCC: 432; No HCC: 216)<br>**Age:** HCC: Median 63 (range 58, 67)<br>No HCC: Median 59 (range 53, 65)<br>**Aetiology:** HCC: Viral 135 (62.5%); Alcohol 33 (15.3%); NAFLD/NASH 29 (13.4%); Other / unknown 19 (8.8%)<br>No HCC: Viral 243 (112.5%); Alcohol 69 (31.9%); NAFLD/NASH 66 (30.6%); Other / unknown 54 (25.0%)<br>**Cirrhosis severity:** HCC: Child's grade A 124 (57.4%)<br>No HCC: Child's grade A 225 (104.2%) | **Index test(s):** B-mode US; Dynamic contrast-enhanced MRI (abbreviated protocol)<br>**Reference standard to establish presence of HCC:** Imaging (HCC was defined by histopathology or LI-RADS diagnostic criteria (LR-5 lesion) on CT or MRI scans)<br>**Reference standard to establish absence of HCC:** Imaging (with 6-months any follow-up) (Control patients had cirrhosis without HCC, confirmed through 12 months of clinical follow-up.) |

128 Sadeghi, M, Lahdou, I, Oweira, H, Daniel, V, Terness, P, Schmidt, J, et al. Serum levels of chemokines CCL4 and CCL5 in cirrhotic patients indicate the presence of hepatocellular carcinoma. British journal of cancer 2015;113(5):756-62.

129 Sanai, Faisal M, Sobki, Samia, Bzeizi, Khalid I, Shaikh, Shaffi A, Alswat, Khalid, Al-Hamoudi, Waleed, et al. Assessment of alpha-fetoprotein in the diagnosis of hepatocellular carcinoma in Middle Eastern patients. Digestive diseases and sciences 2010;55(12):3568-75.

130 Seif El Dahan, K., Yokoo, T., Daher, D., Davenport, M. S., Fetzer, D. T., Mendiratta-Lala, M., et al. Multicenter evaluation of abbreviated MRI and ultrasound for detecting early-stage hepatocellular carcinoma. JHEP Reports 2025;7(5):101357.

| Study | Study details | Study population | Index test and reference standard |
|---|---|---|---|
| Simao et al. (2015)[131] | **Study design:** 1-gate<br>**Method of recruitment:** Clinical cohort<br>**Setting:** Portugal | **Participants, n:** 90 (HCC: 45; No HCC: 45)<br>**Age:** HCC: Mean 64.6 (SD 7.7)<br>No HCC: Mean 58.7 (SD 10.8)<br>**Aetiology:** Alcohol 90 (100.0%)<br>**Cirrhosis severity:** HCC: Child's grade A 21 (46.7%)<br>No HCC: Child's grade A 20 (44.4%) | **Index test(s):** AFP<br>**Reference standard:** Imaging (Diagnosed according to the non invasive criteria of EASL–EORTC guidelines.) |
| Singal et al. (2012)[132]<br>Secondary publication(s): Singal et al. (2022); Singal et al. (2022) | **Study design:** 1-gate<br>**Method of recruitment:** Surveillance programme<br>**Setting:** USA | **Participants, n:** 41 (HCC: 41)<br>**Age:** Median 52.8 (range 23.6, 82.4)<br>**Aetiology:** HCV 209 (47.3%); HBV 18 (4.1%); Alcohol 64 (14.5%)<br>**Cirrhosis severity:** Child's grade A 189 (42.8%) | **Index test(s):** AFP; US; US or AFP; GALAD; HES; AFP-L3; DCP (ng/ml); Longitudinal GALAD<br>**Reference standard:** Imaging ('If an AFP level was elevated or mass lesion was seen on ultrasound, the usual practice was to conduct triple-phase CT or MRI to evaluate the presence of HCCs as recommended by AASLD guidelines'.) |
| Singal et al. (2022)[133] | **Study design:** 2-gate<br>**Method of recruitment:** Surveillance programme<br>**Setting:** USA | **Participants, n:** 87 (HCC: 29; No HCC: 58)<br>**Age:** HCC: Median 53 (95% CI: 44.0-67.0)<br>No HCC: Median 51.5 (95% CI: 27.0-76.0)<br>**Aetiology:** HCC: HCV 18 (62.1%); Alcohol 5 (17.2%); HBV 0 (0.0%)<br>No HCC: HBV 4 (6.9%); Alcohol 10 (17.2%); HCV 32 (55.2%)<br>**Cirrhosis severity:** HCC: Child's grade A 9 (31.0%)<br>No HCC: Child's grade A 20 (34.5%) | **Index test(s):** GALAD; Doylestown plus algorithm; AFP-L3; DCP; AFP<br>**Reference standard to establish presence of HCC:** Imaging (AASLD criteria.)<br>**Reference standard to establish absence of HCC:** Imaging (HCC surveillance for >1 year.) |

[131] Simao, Adelia, Madaleno, Joao, Silva, Nuno, Rodrigues, Fernando, Caseiro, Paula, Costa, Jose Nascimento. Plasma osteopontin is a biomarker for the severity of alcoholic liver cirrhosis, not for hepatocellular carcinoma screening. BMC gastroenterology 2015;15(100968547):73.

[132] Singal, Amit G, Conjeevaram, Hari S, Volk, Michael L, Fu, Sherry, Fontana, Robert J, Askari, Fred, et al. Effectiveness of hepatocellular carcinoma surveillance in patients with cirrhosis. Cancer epidemiology, biomarkers & prevention : a publication of the American Association for Cancer Research, cosponsored by the American Society of Preventive Oncology 2012;21(5):793-9.

[133] Singal, Amit G., Tayob, Nabihah, Mehta, Anand, Marrero, Jorge A., Jin, Qingchun, Lau, Joy. Doylestown Plus and GALAD Demonstrate High Sensitivity for HCC Detection in Patients With Cirrhosis. Clinical gastroenterology and hepatology : the official clinical practice journal of the American Gastroenterological Association 2022;20(4):953-5.e2e2.

| Study | Study details | Study population | Index test and reference standard |
|---|---|---|---|
| Song et al. (2020)[134] | **Study design:** 2-gate<br>**Method of recruitment:** Clinical cohort<br>**Setting:** China | **Participants, n:** 119 (HCC: 55; No HCC: 64)<br>**Age:** HCC: >50: 20 (36.4%)<br>No HCC: >50: 9 (14.1%)<br>**Aetiology:** HBV 119 (100.0%)<br>**Cirrhosis severity:** HCC: Child's grade A 45 (81.8%)<br>No HCC: Child's grade A 47 (73.4%) | **Index test(s):** AFP; DCP; AFP and DCP<br>**Reference standard to establish presence of HCC:** Histology/imaging (Imaging, confirmed by histology for all patients.)<br>**Reference standard to establish absence of HCC:** Not reported (Patients had at least 6 months follow-up to exclude HCC.) |
| Sterling et al. (2009)[135]<br>Secondary publication(s): Sterling et al. (2007) | **Study design:** 1-gate<br>**Method of recruitment:** Surveillance programme<br>**Setting:** USA | **Participants, n:** 372 (HCC: 74; No HCC: 298)<br>**Age:** HCC: Mean 54.9 (SD 6.8)<br>No HCC: Mean 52.1 (SD 6.6)<br>**Aetiology:** HCV 372 (100.0%)<br>**Cirrhosis severity:** HCC: Child's grade A 17 (23.0%)<br>No HCC: Child's grade A 95 (31.9%) | **Index test(s):** AFP; AFP-L3; DCP (ng/ml); AFP or DCP (ng/ml); AFP or AFP-L3; AFP-L3 or DCP (ng/ml); AFP or AFP-L3 or DCP (ng/ml)<br>**Reference standard:** Histology/imaging (Imaging every 6-12 months. HCC diagnosed by histology or EASL criteria as follows: 'presence of cirrhosis and a newly diagnosed focal lesion 2 cm or larger with arterial enhancement on 2 imaging studies (including US, CT, MRI, or angiography) or a focal lesion 2 cm or larger with arterial enhancement on one imaging study associated with a total AFP level greater than 400 ng/ml') |
| Sterling et al. (2012)[136]<br>Secondary publication(s): Tayob et al. (2016); Tayob et al. (2018) | **Study design:** 1-gate<br>**Method of recruitment:** Clinical cohort<br>**Setting:** USA | **Participants, n:** 151 (HCC: 49; No HCC: 102)<br>**Age:** HCC: Mean 53.7 (SD 7.4)<br>No HCC: Mean 50.5 (SD 6.8)<br>**Aetiology:** HCV 409 (100.0%) | **Index test(s):** AFP; AFP-L3; DCP; AFP + AFP-L3; AFP + DCP; AFP + DCP + AFP-L3; serial AFP - absolute value above threshold AND do; PEB algorithm (AFP); mFB-J algorithm; uFB algorithm (AFP); mFB-I algorithm; uFB algorithm (DCP); PEB algorithm (DCP)<br>**Reference standard:** Imaging ('Per-protocol ultrasound examinations of the liver were repeated 6 and 12 months after enrollment and again every 12 months.' Patients testing positive for AFP or US had CT or MRI scans.) |

[134] Song, Ting, Wang, Lili, Xin, Ruopei, Zhang, Liping. Evaluation of serum AFP and DCP levels in the diagnosis of early-stage HBV-related HCC under different backgrounds. The Journal of international medical research 2020;48(10):300060520969087.

[135] Sterling, Richard K, Jeffers, Lennox, Gordon, Fredric, Venook, Alan P, Reddy, K Rajender, Satomura, Shinji, et al. Utility of Lens culinaris agglutinin-reactive fraction of alpha-fetoprotein and des-gamma-carboxy prothrombin, alone or in combination, as biomarkers for hepatocellular carcinoma. Clinical gastroenterology and hepatology : the official clinical practice journal of the American Gastroenterological Association 2009;7(1):104-13.

[136] Sterling, Richard K, Wright, Elizabeth C, Morgan, Timothy R, Seeff, Leonard B, Hoefs, John C, Di Bisceglie, Adrian M, Dienstag, Jules L. Frequency of elevated hepatocellular carcinoma (HCC) biomarkers in patients with advanced hepatitis C. The American journal of gastroenterology 2012;107(1):64-74.

| Study | Study details | Study population | Index test and reference standard |
|---|---|---|---|
| Su et al. (2025)[137] | **Study design:** 1-gate<br>**Method of recruitment:** Clinical cohort<br>**Setting:** China | **Participants, n:** 226 (HCC: 70; No HCC: 156)<br>**Age:** HCC: Mean 50.3 (SD 11.5)<br>No HCC: Mean 58.9 (SD 11.2)<br>**Aetiology:** HCC: HBV 70 (100.0%)<br>No HCC: HBV 156 (100.0%)<br>**Cirrhosis severity:** HCC: Child's grade A 49 (70.0%)<br>No HCC: Child's grade A 105 (67.3%) | **Index test(s):** AFP; DCP (mAU/mL)<br>**Reference standard:** Histology/imaging ('After integrating the patient's AFP and other serumbiomarkers with enhanced CT, enhanced MRI, and liver biopsy results, the diagnosis is confirmed by two experienced senior physicians.') |
| Sultanik et al. (2017)[138] | **Study design:** 2-gate<br>**Method of recruitment:** Clinical cohort<br>**Setting:** France | **Participants, n:** 162 (HCC: 46; No HCC: 116)<br>**Age:** No HCC: Median 52 (IQR = 45-60)<br>HCC: Median 60 (IQR = 52-68)<br>**Aetiology:** HCC: Alcohol 4 (8.7%); HCV 46 (100.0%); HBV 20 (43.5%); NAFLD/NASH 3 (6.5%)<br>No HCC: HCV 114 (98.3%); HBV 3 (2.6%); Alcohol 13 (11.2%); NAFLD/NASH 4 (3.4%)<br>**Cirrhosis severity:** HCC: Child's grade A 36 (78.3%)<br>No HCC: Child's grade A 95 (81.9%) | **Index test(s):** AFP; DCP; AFP or DCP<br>**Reference standard to establish presence of HCC:** Imaging (US, CT and MRI, with or without AFP)<br>**Reference standard to establish absence of HCC:** Not reported ('The absence of HCC was confirmed 1 year after the time of tumour biomarker measurement.') |
| Sun et al. (2012)[139] | **Study design:** 2-gate<br>**Method of recruitment:** Clinical cohort<br>**Setting:** China | **Participants, n:** 54 (HCC: 27; No HCC: 27)<br>**Age:** HCC: Mean 45 (SD 11)<br>No HCC: Mean 45 (SD 12)<br>**Aetiology:** HBV 54 (100.0%) | **Index test(s):** AFP<br>**Reference standard to establish presence of HCC:** Not reported ('The sixth edition of TNM classification of the American Joint Committee on cancer was use to select early HCC patients.')<br>**Reference standard to establish absence of HCC:** Not reported |

[137] Su, R., Tao, X., Yan, L., Liu, Y., Chen, C. C., Li, P., et al. Early screening, diagnosis and recurrence monitoring of hepatocellular carcinoma in chronic hepatitis B patients based on serum N-glycomics analysis: A cohort study. Hepatology 2025;():n. pag..

[138] Sultanik, P, Ginguay, A, Vandame, J, Popovici, T, Meritet, J-F, Cynober, L, Pol, S. Diagnostic accuracy of des-gamma-carboxy prothrombin for hepatocellular carcinoma in a French cohort using the Lumipulse R G600 analyzer. Journal of viral hepatitis 2017;24(1):80-85.

[139] Sun, Chun, Chen, Pei, Chen, Qiaopei, Sun, Lu, Kang, Xiaonan, Qin, Xue. Serum paraoxonase 1 heteroplasmon, a fucosylated, and sialylated glycoprotein in distinguishing early hepatocellular carcinoma from liver cirrhosis patients. Acta biochimica et biophysica Sinica 2012;44(9):765-73.

| Study | Study details | Study population | Index test and reference standard |
|---|---|---|---|
| Tahon et al. (2019)[140] | **Study design:** 2-gate<br>**Method of recruitment:** Clinical cohort<br>**Setting:** Egypt | **Participants, n:** 70 (HCC: 40; No HCC: 30)<br>**Age:** HCC: Mean 61.2 (SD 4.2)<br>No HCC: Mean 53 (SD 4.6)<br>**Aetiology:** HCV 70 (100.0%); HBV 4 (5.7%) | **Index test(s):** AFP; Triphasic spiral CT<br>**Reference standard to establish presence of HCC:** Imaging ('A positive US and triphasic CT for malignant focal lesion.')<br>**Reference standard to establish absence of HCC:** Imaging (US carried out for all patients.) |
| Takaya et al. (2019)[141] | **Study design:** 1-gate<br>**Method of recruitment:** Clinical cohort<br>**Setting:** Japan | **Participants, n:** 61 (HCC: 41; No HCC: 20)<br>**Age:** HCC: Median 78 (IQR = 67-79)<br>No HCC: Median 72 (IQR = 64-76)<br>**Aetiology:** HCC: NAFLD/NASH 6 (14.6%); HCV 17 (41.5%); HBV 11 (26.8%); Alcohol 6 (14.6%)<br>No HCC: NAFLD/NASH 0 (0.0%); HCV 11 (55.0%); HBV 5 (25.0%); Alcohol 2 (10.0%) | **Index test(s):** AFP; AFP-L3; DCP<br>**Reference standard:** Imaging (CT, MRI or US used to diagnose HCC. Not clear whether patients without HCC received a reference standard.) |
| Tayob et al. (2018)[142] | **Study design:** 1-gate<br>**Method of recruitment:** Clinical cohort<br>**Setting:** USA | **Participants, n:** 6062 (HCC: 451; No HCC: 5611)<br>**Age:** HCC: Mean 54.9 (SD 7.5)<br>No HCC: Mean 52.9 (SD 7.4)<br>**Aetiology:** HCV 6062 (100.0%) | **Index test(s):** PEB algorithm or AFP; AFP<br>**Reference standard:** Imaging (Used ICD-9 codes to identify HCC patients from cohort. Then manually reviewed medical recrods for lesion on US that was then confirmed by CT, MRI or biopsy.) |
| Tayob et al. (2022)[143] | **Study design:** 1-gate<br>**Method of recruitment:** Surveillance programme<br>**Setting:** USA | **Participants, n:** 534 (HCC: 50; No HCC: 484)<br>**Age:** HCC: Mean 64.7 (SD 5.7)<br>No HCC: Mean 63 (SD 6.7)<br>**Aetiology:** HCC: HCV 42 (84.0%); Alcohol 36 (72.0%); HBV 0 (0.0%); NAFLD/NASH 10 (20.0%)<br>No HCC: HCV 339 (70.0%); NAFLD/NASH 104 (21.5%); Alcohol 290 (59.9%); HBV 12 (2.5%) | **Index test(s):** AFP; AFP-L3; DCP (ng/ml); GALAD; HES; AFP or AFP-L3 or DCP (ng/ml)<br>**Reference standard:** Histology/imaging (AASLD criteria including histological or radiological diagnosis (CT or MRI).) |

[140] Tahon, Ahmed M, El-Ghanam, Magdy Z, Zaky, Samy, Emran, Tarek Mostafa, Bersy, Ali M, El-Raey, Fathiya, et al. Significance of Glypican-3 in Early Detection of Hepatocellular Carcinoma in Cirrhotic Patients. Journal of gastrointestinal cancer 2019;50(3):434-41.

[141] Takaya, Hiroaki, Namisaki, Tadashi, Kitade, Mitsuteru, Kaji, Kosuke, Nakanishi, Keisuke, Tsuji, Yuki, et al. VWF/ADAMTS13 ratio as a potential biomarker for early detection of hepatocellular carcinoma. BMC gastroenterology 2019;19(1):167.

[142] Tayob, Nabihah, Richardson, Peter, White, Donna L., Yu, Xiaoying, Davila, Jessica A., Kanwal, Fasiha, Feng, Ziding. Evaluating screening approaches for hepatocellular carcinoma in a cohort of HCV related cirrhosis patients from the Veteran's Affairs Health Care System. BMC medical research methodology 2018;18(1):1.

[143] Tayob, Nabihah, Kanwal, Fasiha, Alsarraj, Abeer, Hernaez, Ruben. The Performance of AFP, AFP-3, DCP as Biomarkers for Detection of Hepatocellular Carcinoma (HCC): A Phase 3 Biomarker Study in the United States. Clinical gastroenterology and hepatology : the official clinical practice journal of the American Gastroenterological Association 2022;():n. pag..

| Study | Study details | Study population | Index test and reference standard |
| --- | --- | --- | --- |
| Toraih et al. (2018)[144] | **Study design:** 2-gate<br>**Method of recruitment:** Clinical cohort<br>**Setting:** Egypt | **Participants, n:** 50 (HCC: 30; No HCC: 20)<br>**Age:** HCC: Mean 53.4 (SD 12.4)<br>No HCC: Mean 51.8 (SD 14.7)<br>**Aetiology:** HCV 50 (100.0%)<br>**Cirrhosis severity:** HCC: Child's grade A 0 (0.0%)<br>No HCC: Child's grade A 16 (80.0%) | **Index test(s):** AFP; MALAT1 RNA<br>**Reference standard to establish presence of HCC:** Imaging (Typical imaging findings and AFP. All HCC patients had US.)<br>**Reference standard to establish absence of HCC:** Not reported (null) |
| Toraih et al. (2019)[145] | **Study design:** 2-gate<br>**Method of recruitment:** Clinical cohort<br>**Setting:** Egypt | **Participants, n:** 30 (No HCC: 30)<br>**Age:** HCC: Mean 50.7 (SD 9.2)<br>HCC: Mean 55.2 (SD 7.2)<br>No HCC: Mean 53.4 (SD 7.2)<br>**Aetiology:** HCV 90 (100.0%) | **Index test(s):** TOM34; HSPA4; HSPA1B; HSP90AA1<br>**Reference standard to establish presence of HCC:** Imaging (US and CT.)<br>**Reference standard to establish absence of HCC:** Imaging (US and CT.) |
| Tsai et al. (2005)[146] | **Study design:** 2-gate<br>**Method of recruitment:** Clinical cohort<br>**Setting:** Taiwan | **Participants, n:** 82 (HCC: 41; No HCC: 41)<br>**Age:** HCC: Median 58 (range 29, 77)<br>No HCC: Median 57 (range 28, 78)<br>**Cirrhosis severity:** HCC: Child's grade A 25 (61.0%)<br>No HCC: Child's grade A 23 (56.1%) | **Index test(s):** AFP<br>**Reference standard to establish presence of HCC:** Imaging (Biopsy or aspiration cytology after abdominal sonography.)<br>**Reference standard to establish absence of HCC:** Imaging (No space-occupying lesion, evidenced by abdominal sonography.) |
| Unic et al. (2018)[147] | **Study design:** 1-gate<br>**Method of recruitment:** Clinical cohort<br>**Setting:** Croatia | **Participants, n:** 63 (HCC: 32; No HCC: 31)<br>**Age:** HCC: Median 67 (IQR: 53-79)<br>No HCC: Median 64 (IQR: 42-81)<br>**Aetiology:** Alcohol 63 (100.0%) | **Index test(s):** DCP (ng/ml)<br>**Reference standard:** Imaging (All patients had CT and/or MRI.) |

[144] Toraih, Eman A, Ellawindy, Alia, Fala, Salma Y, Al Ageeli, Essam, Gouda, Nawal S, Fawzy, Manal S. Oncogenic long noncoding RNA MALAT1 and HCV-related hepatocellular carcinoma. Biomedicine & pharmacotherapy = Biomedecine & pharmacotherapie 2018;102(a59, 8213295):653-69.

[145] Toraih, Eman A., Alrefai, Hani G., Hussein, Mohammad H., Helal, Ghada M., Khashana, Moataz S. Overexpression of heat shock protein HSP90AA1 and translocase of the outer mitochondrial membrane TOM34 in HCV-induced hepatocellular carcinoma: A pilot study. Clinical biochemistry 2019;63():10-17.

[146] Tsai, J. F., Jeng, J. E., Chuang, L. Y., You, H. L., Wang, L. Y., Hsieh, M. Y., et al. Serum insulin-like growth factor-II as a serologic marker of small hepatocellular carcinoma. Scandinavian journal of gastroenterology 2005;40(1):68-75.

[147] Unic, Adriana, Derek, Lovorka, Duvnjak, Marko, Patrlj, Leonardo, Rakic, Mislav, Kujundzic, Milan, et al. Diagnostic specificity and sensitivity of PIVKAII, GP3, CSTB, SCCA1 and HGF for the diagnosis of hepatocellular carcinoma in patients with alcoholic liver cirrhosis. Annals of clinical biochemistry 2018;55(3):355-62.

| Study | Study details | Study population | Index test and reference standard |
|---|---|---|---|
| Villacastin Ruiz et al. (2016)[148] | **Study design:** 1-gate<br>**Method of recruitment:** Surveillance programme<br>**Setting:** Spain | **Participants, n:** 273<br>**Age:** Mean 55 (SD 7.9)<br>**Aetiology:** HBV 11 (4.0%); HCV 52 (19.0%); HCV/HBV/alcohol (any combination of the 3) 50 (18.3%); Alcohol 134 (49.1%) | **Index test(s):** multidetector CT; US; US and CT<br>**Reference standard:** Explant pathology following transplantation |
| Volk et al. (2007)[149] | **Study design:** 2-gate<br>**Method of recruitment:** Clinical cohort<br>**Setting:** USA | **Participants, n:** 254 (HCC: 85; No HCC: 169)<br>**Age:** HCC: Mean 59 (SD 12)<br>No HCC: Mean 53 (SD 8)<br>**Aetiology:** HCC: HBV 6 (7.1%); Alcohol 7 (8.3%); HCV 48 (57.1%)<br>No HCC: HCV 61 (36.1%); Alcohol 34 (20.1%); HBV 8 (4.7%) | **Index test(s):** AFP; AFP-L3; DCP; AFP or DCP; AFP-L3 or DCP; AFP or AFP-L3 or DCP<br>**Reference standard to establish presence of HCC:** Imaging (CT and MRI)<br>**Reference standard to establish absence of HCC:** Blood tests and imaging ('US showing no mass was required if the total AFP was < 20 ng/m, and CT or MRI was required if the AFP was > 20 ng/ml.' 'Patients were followed for a median of 14 months (range: 8–37) and had at least one follow-up imaging to assure that none had developed HCC.') |
| Wang et al. (2010)[150] | **Study design:** 2-gate<br>**Method of recruitment:** Clinical cohort<br>**Setting:** China | **Participants, n:** 105 (HCC: 76; No HCC: 29)<br>**Age:** HCC: Mean 51.4 (SD 11.3)<br>No HCC: Mean 56.6 (SD 11.6)<br>**Aetiology:** HBV 105 (100.0%) | **Index test(s):** AFP<br>**Reference standard to establish presence of HCC:** Histology/imaging (Histological diagnosis when possible. Otherwise US, CT and hepatic angiography along with AFP.)<br>**Reference standard to establish absence of HCC:** Imaging (All patients had US.) |
| Wang et al. (2020)[151] | **Study design:** 2-gate<br>**Method of recruitment:** Clinical cohort<br>**Setting:** China | **Participants, n:** 100 (Control: 50; HCC: 50)<br>**Age:** HCC: Mean 62.8 (SD 8.1)<br>Control: Mean 62 (SD 9.6) | **Index test(s):** AFP<br>**Reference standard to establish presence of HCC:** Blood tests and imaging (Blood tests, US, CT and/or MRI.)<br>**Reference standard to establish absence of HCC:** Not reported |

[148] Villacastin Ruiz, Elena, Caro-Paton Gomez, Agustin, Calero Aguilar, Hermogenes, Perez Saborido, Baltasar, Garcia Pajares, Felix, Sanchez Antolin, Gloria, et al. Review of imaging techniques in the diagnosis of hepatocellular carcinoma in patients who require a liver transplant. European journal of gastroenterology & hepatology 2016;28(4):412-20.

[149] Volk, Michael L, Hernandez, Jose C, Su, Grace L, Lok, Anna S. Risk factors for hepatocellular carcinoma may impair the performance of biomarkers: a comparison of AFP, DCP, and AFP-L3. Cancer biomarkers : section A of Disease markers 2007;3(2):79-87.

[150] Wang, Y., Liu, Y. H., Mai, S. J., He, L. J., Liao, Y. J., Deng, H. X., et al. Evaluation of serum clusterin as a surveillance tool for human hepatocellular carcinoma with hepatitis B virus related cirrhosis. Journal of Gastroenterology and Hepatology (Australia) 2010;25(6):1123-28.

[151] Wang, Shuo, Yang, Yongxiang, Sun, Lili, Qiao, Guoliang, Song, Yunlong. Exosomal MicroRNAs as Liquid Biopsy Biomarkers in Hepatocellular Carcinoma. OncoTargets and therapy 2020;13():2021-30.

| Study | Study details | Study population | Index test and reference standard |
|---|---|---|---|
| Weis et al. (2019)[152] | **Study design:** 2-gate<br>**Method of recruitment:** Clinical cohort<br>**Setting:** Australia | **Participants, n:** 40 (HCC: 20; No HCC: 20)<br>**Age:** HCC: Mean 58.3 (SD 5.3)<br>No HCC: Mean 53.8 (SD 7.4)<br>**Aetiology:** HCV 40 (100.0%) | **Index test(s):** AFP<br>**Reference standard to establish presence of HCC:** Imaging ('Subdivided into 3 cohorts based on expert clinical assessment, transient elastography and medical imaging.')<br>**Reference standard to establish absence of HCC:** Imaging (null) |
| Woo et al. (2025)[153] | **Study design:** 2-gate<br>**Method of recruitment:** Clinical cohort<br>**Setting:** Malaysia | **Participants, n:** 223 (HCC: 44; No HCC: 179)<br>**Age:** HCC: Mean 64.2 (SD 11.3)<br>No HCC: Mean 64.4 (SD 10.4)<br>**Aetiology:** HCC: HBV 22 (50.0%); HCV 6 (13.6%); NAFLD/NASH 10 (22.7%); Alcohol 3 (6.8%); Other / unknown 3 (6.8%)<br>No HCC: HBV 90 (50.3%); HCV 12 (6.7%); NAFLD/NASH 63 (35.2%); Alcohol 8 (4.5%); Other / unknown 6 (3.4%)<br>**Cirrhosis severity:** HCC: Child's grade A 20 (45.5%)<br>No HCC: Child's grade A 116 (64.8%) | **Index test(s):** AFP; AFP-L3 (%); DCP (mAU/mL); GALAD<br>**Reference standard to establish presence of HCC:** Histology/imaging (diagnosis of HCC followed the EASL guidelines and was based on either pathology or imaging)<br>**Reference standard to establish absence of HCC:** Not reported |
| Worland et al. (2018)[154] | **Study design:** 1-gate<br>**Method of recruitment:** Surveillance programme<br>**Setting:** Australia | **Participants, n:** 64<br>**Age:** Mean 58.7 (range 19, 79)<br>**Aetiology:** NAFLD/NASH 12 (18.8%); Alcohol 42 (65.6%); Non-viral 64 (100.0%) | **Index test(s):** AFP; US; US or AFP<br>**Reference standard:** Histology/imaging (Histology from biopsy, imaging, or natural disease progression. Imaging (CT and MRI) carried out when surveillance tests were postiive. Cirrhosis patients had US and AFP follow-up.) |
| Xing et al. (2023)[155] | **Study design:** 1-gate<br>**Method of recruitment:** Surveillance programme<br>**Setting:** China | NA | **Index test(s):** AFP; DCP (mAU/mL)<br>**Reference standard:** Blood tests and imaging (enrolled LC patients were followed up every 6 months with AFP, liver function tests, a chest CT and ultrasound or a contrast-enhanced CT scan or MRI of the abdomen) |

[152] Weis, Anna, Marquart, Louise, Calvopina, Diego A, Genz, Berit, Ramm, Grant A. Serum MicroRNAs as Biomarkers in Hepatitis C: Preliminary Evidence of a MicroRNA Panel for the Diagnosis of Hepatocellular Carcinoma. International journal of molecular sciences 2019;20(4):n. pag..

[153] Woo, W. H., Nawawi, K. N. M., Chew, D. C. H., Kok, W. H., Wong, Z., Azman, A., et al. The GALAD score performs better than AFP in hepatocellular carcinoma screening: a single-centre, case-control study in Malaysia. Clinical and Experimental Hepatology 2025;11(1):81 “ 87.

[154] Worland, T., Harrison, B., Delmenico, L. Hepatocellular carcinoma screening utilising serum alpha-fetoprotein measurement and abdominal ultrasound is more effective than ultrasound alone in patients with non-viral cirrhosis. Journal of Gastrointestinal Cancer 2018;49(4):476-80.

[155] Xing, X., Cai, L., Ouyang, J., Wang, F., Li, Z., Liu, M., et al. Proteomics-driven noninvasive screening of circulating serum protein panels for the early diagnosis of hepatocellular carcinoma. Nature Communications 2023;14(1):8392.

| Study | Study details | Study population | Index test and reference standard |
|---|---|---|---|
| Yagmur et al. (2007)[156] | **Study design:** 2-gate<br>**Method of recruitment:** Clinical cohort<br>**Setting:** Germany | **Participants, n:** 102 (HCC: 45; No HCC: 57)<br>**Age:** HCC: Median 57 (range 38, 78)<br>No HCC: Median 60 (range 38, 82) | **Index test(s):** AFP<br>**Reference standard to establish presence of HCC:** Histology (Biopsy in all cases.)<br>**Reference standard to establish absence of HCC:** Blood tests and imaging ('The diagnosis of liver cirrhosis without US and radiological signs of HCC was based on clinical findings, regular laboratory and US analysis. CT scans were performed in 35 of these liver cirrhosis cases.') |
| Yang et al. (2005)[157] | **Study design:** 2-gate<br>**Method of recruitment:** Clinical cohort<br>**Setting:** China | **Participants, n:** 87 (HCC: 65; No HCC: 22)<br>**Age:** HCC: Mean 49 (range 28, 72)<br>No HCC: Mean 45 (range 21, 60)<br>**Aetiology:** HBV 87 (100.0%) | **Index test(s):** AFP mRNA; MAGE-1 mRNA<br>**Reference standard to establish presence of HCC:** Histology/imaging (Clinical diagnosis and US, CT and angiography. 51/65 were confirmed with histology.)<br>**Reference standard to establish absence of HCC:** Not reported ('No evidence of HCC.') |
| Yang et al. (2021)[158] | **Study design:** 1-gate<br>**Method of recruitment:** Clinical cohort<br>**Setting:** China | **Participants, n:** 266 (HCC: 121; No HCC: 145)<br>**Age:** HCC: >60: 65 (53.7%)<br>No HCC: >60: 40 (27.6%)<br>**Aetiology:** HCC: Alcohol 45 (37.2%)<br>No HCC: Alcohol 31 (21.4%)<br>**Cirrhosis severity:** HCC: Child's grade A 55 (45.5%)<br>No HCC: Child's grade A 81 (55.9%) | **Index test(s):** AFP<br>**Reference standard:** Histology/imaging (Diagnosis confirmed by 'clinical and serological characteristics, US, CT, MRI, hepatic arteriography with digital subtraction angiography (DSA) and pathological examination.') |

[156] Yagmur, Eray, Rizk, Mohamed, Stanzel, Sven, Hellerbrand, Claus, Lammert, Frank, Trautwein, Christian, Wasmuth, Hermann E. Elevation of endoglin (CD105) concentrations in serum of patients with liver cirrhosis and carcinoma. European journal of gastroenterology & hepatology 2007;19(9):755-61.

[157] Yang, Shi-Zhong, Dong, Jia-Hong, Li, Kun, Zhang, Yu. Detection of AFPmRNA and melanoma antigen gene-1mRNA as markers of disseminated hepatocellular carcinoma cells in blood. Hepatobiliary & pancreatic diseases international : HBPD INT 2005;4(2):227-33.

[158] Yang, D. H., Pan, H. Y., Huang, Y. C., Zhang, J. J., Wang, W. P. Hepatocellular carcinoma progression in hepatitis B virus-related cirrhosis patients receiving nucleoside (acid) analogs therapy: A retrospective cross-sectional study. World Journal of Gastroenterology 2021;27(17):2025-38.

| Study | Study details | Study population | Index test and reference standard |
|---|---|---|---|
| Yokoo et al. (2023)[159] | **Study design:** 1-gate<br>**Method of recruitment:** Clinical cohort<br>**Setting:** USA | **Participants, n:** 299 (HCC: 161; No HCC: 138)<br>**Age:** HCC: Median 62 (range 58, 67)<br>No HCC: Median 55 (range 47, 63)<br>**Aetiology:** HCC: HBV 13 (8.1%); HCV 108 (67.1%); Alcohol 32 (19.9%); NAFLD/NASH 24 (14.9%); Other / unknown 14 (8.7%)<br>No HCC: HBV 5 (3.6%); HCV 23 (16.7%); Alcohol 76 (55.1%); NAFLD/NASH 53 (38.4%); Other / unknown 37 (26.8%)<br>**Cirrhosis severity:** HCC: Child's grade A 129 (80.1%)<br>No HCC: Child's grade A 3 (2.2%) | **Index test(s):** MRI<br>**Reference standard:** Explant pathology following transplantation (null) |
| Yoon et al. (2020)[160] | **Study design:** 1-gate<br>**Method of recruitment:** Surveillance programme<br>**Setting:** South Korea | **Participants, n:** 24 (HCC: 24)<br>**Age:** Mean 59.3 (SD 7.6)<br>**Aetiology:** HBV 124 (89.9%); HCV 3 (2.2%); Alcohol 13 (9.4%)<br>**Cirrhosis severity:** Child's grade A 99 (71.7%) | **Index test(s):** US; AFP; CT<br>**Reference standard:** Reference standard only carried out when surveilla (CT or MRI for patients testing positive during surveillance. 'In participants without detected hepatic observations during the study period, absence or presence of HCC was determined by follow-up imaging in the 6 –12 months following the last two-phase LDCT.') |
| Youssef et al. (2018)[161] | **Study design:** 2-gate<br>**Method of recruitment:** Clinical cohort<br>**Setting:** Egypt | **Participants, n:** 195 (HCC: 105; No HCC: 90)<br>**Age:** HCC: Mean 56.6 (SD 11.4)<br>No HCC: Mean 57.3 (SD 13.5)<br>**Aetiology:** HCV 195 (100.0%) | **Index test(s):** AFP<br>**Reference standard to establish presence of HCC:** Blood tests and imaging (AFP and US, and confirmed by CT.)<br>**Reference standard to establish absence of HCC:** Imaging (All patients had US.) |

[159] Yokoo, Takeshi, Masaki, Nobuaki, Parikh, Neehar D., Lane, Barton F., Feng, Ziding, Mendiratta-Lala, Mishal, et al. Multicenter Validation of Abbreviated MRI for Detecting Early-Stage Hepatocellular Carcinoma. Radiology 2023;307(2):e220917.

[160] Yoon, Jeong Hee, Lee, Jeong Min, Lee, Dong Ho, Joo, Ijin, Jeon, Ju Hyun, Ahn, Su Joa, et al. A Comparison of Biannual Two-Phase Low-Dose Liver CT and US for HCC Surveillance in a Group at High Risk of HCC Development. Liver cancer 2020;9(5):503-17.

[161] Youssef, Anas A., Issa, Hisham A., Omar, Maha Z., Behiry, Eman G., Elfallah, Asmaa A., Hasaneen, Ali, Darwish, Mohamed. Serum human endothelial cell-specific molecule-1 (endocan) and vascular endothelial growth factor in cirrhotic HCV patients with hepatocellular carcinoma as predictors of mortality. Clinical and experimental gastroenterology 2018;11():431-38.

| Study | Study details | Study population | Index test and reference standard |
|---|---|---|---|
| Yu et al. (2011)[162] | **Study design:** 1-gate<br>**Method of recruitment:** Surveillance programme<br>**Setting:** USA | **Participants, n:** 125 (HCC arm: 125)<br>**Age:** Mean 53.2 (range 18, 75)<br>**Aetiology:** Alcohol 54 (8.5%); HCV/HBV/alcohol (any combination of the 3) 54 (8.5%); HBV 66 (10.3%); HCV 277 (43.4%) | **Index test(s):** US; MRI; AFP; CT<br>**Reference standard:** Explant pathology following transplantation |
| Zha et al. (2021)[163] | **Study design:** 1-gate<br>**Method of recruitment:** Surveillance programme<br>**Setting:** China | **Participants, n:** 41 (HCC: 41)<br>**Age:** HCC: Mean 55.1 (SD 9.4)<br>**Aetiology:** HCC: HBV 30 (73.2%); HCV 0 (0.0%); Alcohol 3 (7.3%)<br>**Cirrhosis severity:** HCC: Child's grade A 18 (43.9%) | **Index test(s):** AFP<br>**Reference standard:** Histology/imaging (Patients testing positive on AFP/surveillance imaging received CT, MRI, US, liver biopsy, and surgical pathology for final diagnosis.) |
| Zhang et al. (2019)[164] | **Study design:** 1-gate<br>**Method of recruitment:** Clinical cohort<br>**Setting:** China | **Participants, n:** 574 (HCC: 288; No HCC: 286)<br>**Age:** HCC: Mean 55.7 (SD 11.4)<br>No HCC: Mean 50.7 (SD 12.2)<br>**Aetiology:** HCC: HBV 230 (79.9%)<br>No HCC: HBV 229 (80.1%)<br>**Cirrhosis severity:** HCC: Child's grade A 160 (55.6%)<br>No HCC: Child's grade A 154 (53.8%) | **Index test(s):** AFP<br>**Reference standard:** Histology/imaging (null) |

[162] Yu, Nam C, Chaudhari, Vinika, Raman, Steven S, Lassman, Charles, Tong, Myron J, Busuttil, Ronald W. CT and MRI improve detection of hepatocellular carcinoma, compared with ultrasound alone, in patients with cirrhosis. Clinical gastroenterology and hepatology : the official clinical practice journal of the American Gastroenterological Association 2011;9(2):161-7.

[163] Zha, Zhengwei, Wu, Wenyue, Zhang, Qianqian, Wang, Xi, Mei, Xuecan, Xiang, Yi. Screening, clinical features and prognostic analysis of liver cirrhosis-related hepatocellular carcinoma. Scandinavian journal of gastroenterology 2021;56(8):948-54.

[164] Zhang, Xi, Wang, Ting, Zhang, Kun-He, Chen, Si-Hai, He, Yu-Ting. Simple Clinical Metrics Enhance AFP to Effectively Identify Cirrhotic Patients With Complicating Hepatocellular Carcinoma at Various AFP Levels. Frontiers in oncology 2019;9():1478.

| Study | Study details | Study population | Index test and reference standard |
| --- | --- | --- | --- |
| Zhao et al. (2017)[165] | **Study design:** 2-gate<br>**Method of recruitment:** Clinical cohort<br>**Setting:** China | **Participants, n:** 236 (HCC: 149; No HCC: 87)<br>**Age:** HCC: Mean 55.1 (SD 9.9)<br>No HCC: Mean 54.6 (SD 12.1)<br>**Aetiology:** HCC: HBV 61 (40.9%); HCV 40 (26.8%); Non-viral 48 (32.2%)<br>No HCC: HBV 42 (48.3%); HCV 24 (27.6%); Non-viral 21 (24.1%)<br>**Cirrhosis severity:** HCC: Child's grade A 62 (41.6%)<br>No HCC: Child's grade A 45 (51.7%) | **Index test(s):** AFP<br>**Reference standard to establish presence of HCC:** Histology (null)<br>**Reference standard to establish absence of HCC:** Not reported |
| Zheng et al. (2021)[166] | **Study design:** 2-gate<br>**Method of recruitment:** Clinical cohort<br>**Setting:** China | **Participants, n:** 64 (HCC: 32; No HCC: 32)<br>**Age:** HCC: Mean 57.1 (SD 8.8)<br>No HCC: Mean 56.3 (SD 11.9)<br>**Aetiology:** HCC: HBV 20 (62.5%); Other / unknown 10 (31.2%); HCV 2 (6.2%)<br>No HCC: HCV 4 (12.5%); Other / unknown 9 (28.1%); HBV 19 (59.4%) | **Index test(s):** Gd-DTPA-enhanced MRI; PM-DL<br>**Reference standard to establish presence of HCC:** Histology (A total of 290 consecutive patients with pathologically confirmed HCCs were recruited for liver Gd-DTPA-enhanced MRI between June 2016 and October 2019.)<br>**Reference standard to establish absence of HCC:** Imaging (Liver cirrhosis was diagnosed based on typical imaging features) |
| Zhou et al. (2020)[167] | **Study design:** 2-gate<br>**Method of recruitment:** Clinical cohort<br>**Setting:** China | **Participants, n:** 59 (HCC: 30; No HCC: 29)<br>**Age:** HCC: Mean 52.9 (SD 11)<br>No HCC: Mean 56.6 (SD 9.2)<br>**Aetiology:** HCC: HBV 26 (86.7%); HCV 2 (6.7%)<br>No HCC: HBV 24 (82.8%); HCV 2 (6.9%)<br>**Cirrhosis severity:** HCC: Child's grade A 18 (60.0%)<br>No HCC: Child's grade A 15 (51.7%) | **Index test(s):** AFP<br>**Reference standard to establish presence of HCC:** Imaging (Histology or two different imaging techniques, 'or the combination of one imaging technique and an AFP level of > 400 ng/mL'.)<br>**Reference standard to establish absence of HCC:** Not reported |

[165] Zhao, Long, Xie, Chao, Liu, Danqing, Li, Tong, Zhang, Yu. Early Detection of Hepatocellular Carcinoma in Patients with Hepatocirrhosis by Soluble B7-H3. Journal of gastrointestinal surgery : official journal of the Society for Surgery of the Alimentary Tract 2017;21(5):807-12.

[166] Zheng, R., Wang, L., Wang, C., Yu, X., Chen, W., Li, Y., et al. Feasibility of automatic detection of small hepatocellular carcinoma (<=2 cm) in cirrhotic liver based on pattern matching and deep learning. Physics in medicine and biology 2021;66(8):n. pag..

[167] Zhou, Peng-Cheng, Sun, Lun-Quan, Shao, Li, Yi, Lun-Zhao, Li, Ning. Establishment of a pattern recognition metabolomics model for the diagnosis of hepatocellular carcinoma. World journal of gastroenterology 2020;26(31):4607-23.

| Study | Study details | Study population | Index test and reference standard |
|---|---|---|---|
| Zhu et al. (2020)[168] | **Study design:** 2-gate<br>**Method of recruitment:** Clinical cohort<br>**Setting:** USA | **Participants, n:** 70 (HCC: 37; No HCC: 33)<br>**Age:** No HCC: Median 62 (IQR: 56-66)<br>HCC: Median 65.8 (IQR: 61-76)<br>**Aetiology:** NAFLD/NASH 70 (100.0%)<br>**Cirrhosis severity:** HCC: Child's grade A 24 (64.9%)<br>No HCC: Child's grade A 28 (84.8%) | **Index test(s):** AFP<br>**Reference standard to establish presence of HCC:** Not reported<br>**Reference standard to establish absence of HCC:** Not reported |
| Zhu et al. (2025)[169] | **Study design:** 2-gate<br>**Method of recruitment:** Clinical cohort<br>**Setting:** China | **Participants, n:** 145 (HCC: 66; No HCC: 79)<br>**Age:** HCC: Mean 55.2 (SD 9.3)<br>No HCC: Mean 51 (SD 12.3)<br>**Aetiology:** HCC: HBV 58 (87.9%); HCV 3 (4.5%); Other / unknown 5 (7.6%)<br>No HCC: HBV 57 (72.2%); HCV 6 (7.6%); Other / unknown 16 (20.3%)<br>**Cirrhosis severity:** HCC: Child's grade A 50 (75.8%)<br>No HCC: Child's grade A 36 (45.6%) | **Index test(s):** AFP<br>**Reference standard to establish presence of HCC:** Unclear (All patients with HCC were diagnosed in accordance with the "Diagnostic and Therapeutic Criteria for Primary Liver Cancer (2022 Edition)" issued by the Ministry of Health of China.)<br>**Reference standard to establish absence of HCC:** Not reported |
| Zinkin et al. (2008)[170] | **Study design:** 2-gate<br>**Method of recruitment:** Clinical cohort<br>**Setting:** USA | **Participants, n:** 92 (HCC: 41; No HCC: 51)<br>**Age:** HCC: Mean 60 (range 43, 82)<br>No HCC: Mean 50 (range 23, 66)<br>**Aetiology:** HCC: Alcohol 6 (14.6%); NAFLD/NASH 1 (2.4%); HBV 5 (12.2%); HCV 26 (63.4%)<br>No HCC: HCV 51 (100.0%)<br>**Cirrhosis severity:** HCC: Child's grade A 19 (46.3%) | **Index test(s):** AFP; AFP-L3; DCP; AFP and AFP-L3<br>**Reference standard to establish presence of HCC:** Histology/imaging ('At least one of the following: (a) histology, (b) new hepatic lesion with an AFP of >1,000 ng/mL, and/or (c) new hepatic lesion with arterial phase enhancement on computed tomography or magnetic resonance imaging.')<br>**Reference standard to establish absence of HCC:** Blood tests and imaging (AFP and US follow-up for at least 2 years.) |

[168] Zhu, Jianhui, Huang, Junfeng, Zhang, Jie, Chen, Zhengwei, Lin, Yu, Grigorean, Gabriela, et al. Glycopeptide Biomarkers in Serum Haptoglobin for Hepatocellular Carcinoma Detection in Patients with Nonalcoholic Steatohepatitis. Journal of proteome research 2020;19(8):3452-66.

[169] Zhu, W., Wang, H., Cai, Y., Lei, J., Yu, J., Li, A. Plasma methylated HIST1H3G as a non-invasive biomarker for diagnostic modeling of hepatocellular carcinoma. Frontiers in Medicine 2025;12():1571737.

[170] Zinkin, Noah T, Grall, Franck, Bhaskar, Killimangalam, Otu, Hasan H, Spentzos, Dimitrios, Kalmowitz, Brett, et al. Serum proteomics and biomarkers in hepatocellular carcinoma and chronic liver disease. Clinical cancer research : an official journal of the American Association for Cancer Research 2008;14(2):470-7.

## Appendix S9 Risk of bias assessment of included studies (QUADAS-2)

### S9.1. Risk of bias assessment for each study

| | Patient selection | | Index test(s) | | Reference standard | | Flow and timing | | Overall |
|---|---|---|---|---|---|---|---|---|---|
| | QUADAS-2 | QUADAS-C | QUADAS-2 | QUADAS-C | QUADAS-2 | QUADAS-C | QUADAS-2 | QUADAS-C | |
| Abdel-Azeez et al. (2022) | × | - | + | - | × | - | ? | - | × |
| Abdel-Razik et al. (2016) | × | × | × | × | × | × | ? | ? | × |
| Abdel Rehem & El-Shikh (2011) | × | - | + | - | × | - | ? | - | × |
| Abdelfattah et al. (2019) | × | - | × | - | × | - | ? | - | × |
| Abdelwahab et al. (2017) | × | - | × | - | × | - | ? | - | × |
| Alrefai et al. (2024) | × | - | × | - | + | - | + | - | × |
| Aly et al. (2020) | × | × | × | × | × | × | × | × | × |
| Amr et al. (2016) | ? | × | × | × | ? | ? | ? | ? | × |
| Arrieta et al. (2007) | × | - | + | - | × | - | × | - | × |
| Atiq et al. (2017) | × | × | + | × | × | × | × | × | × |
| Attallah et al. (2013) | × | × | + | + | × | × | × | × | × |
| Bai et al. (2023) | × | - | × | - | × | - | × | - | × |
| Baird et al. (2013) | × | × | × | × | + | + | × | × | × |
| Beale et al. (2008) | × | × | × | × | ? | ? | ? | ? | × |
| Beudeker et al. (2023) | + | × | × | × | × | × | × | × | × |
| Bhatti et al. (2021) | × | + | × | × | × | × | ? | ? | × |
| Biselli et al. (2015) | × | - | × | - | × | - | + | - | × |
| Brigida et al. (2020) | × | × | + | + | × | × | × | × | × |
| Cai et al. (2024) | × | - | × | - | + | - | + | - | × |
| Caviglia et al. (2017) | × | × | × | × | × | × | × | × | × |
| Caviglia et al. (2020) | × | + | × | × | × | × | × | × | × |
| Caviglia et al. (2023) | × | - | ? | - | × | - | × | - | × |
| Chalasani et al. (2022) | × | - | ? | - | × | - | + | - | × |
| Chang et al. (2015) | + | + | + | × | × | × | × | × | × |
| Chen et al. (2023) | × | + | ? | ? | × | × | × | × | × |
| Chen et al. (2024) | × | - | * | - | + | - | × | - | × |
| Choi et al. (2008) | + | - | + | - | + | - | + | - | + |
| Chong et al. (2022) | × | - | + | - | × | - | × | - | × |
| Comunale et al. (2013) | × | - | + | - | + | - | × | - | × |
| D'Abundo et al. (2024) | × | - | × | - | + | - | + | - | × |
| Dabbish et al. (2022) | × | - | + | - | × | - | × | - | × |
| Demirtas et al. (2020) | × | × | + | × | × | × | × | × | × |
| Ebrahim et al. (2020) | × | - | × | - | × | - | ? | - | × |
| El-Abd et al. (2015) | × | × | + | × | ? | × | × | × | × |
| El-Ahwany et al. (2022) | × | + | ? | × | ? | × | ? | ? | × |
| El-Din Bessa et al. (2010) | × | - | × | - | × | - | × | - | × |
| El-Emshaty et al. (2025) | × | - | × | - | + | - | + | - | × |
| El-Mezayen & Darwish (2014) | × | - | × | - | + | - | × | - | × |
| El-Serag et al. (2025) | + | - | + | - | + | - | + | - | + |
| El-Shayeb et al. (2021) | × | - | + | - | × | - | ? | - | × |
| El-Shendidi et al. (2022) | × | × | × | × | × | × | ? | ? | × |
| Eldeeb et al. (2020) | × | - | × | - | × | - | ? | - | × |

× HIGH RISK ? UNCLEAR RISK + LOW RISK

| | Patient selection | | Index test(s) | | Reference standard | | Flow and timing | | Overall |
|---|---|---|---|---|---|---|---|---|---|
| | QUADAS-2 | QUADAS-C | QUADAS-2 | QUADAS-C | QUADAS-2 | QUADAS-C | QUADAS-2 | QUADAS-C | |
| Eldosoky et al. (2023) | High | Low | Unclear | Unclear | High | High | Unclear | High | High |
| Elgedawy et al. (2025) | High | - | Unclear | - | Low | - | High | - | High |
| Elshimi et al. (2019) | High | High | High | High | High | High | High | High | High |
| Ergul et al. (2024) | High | - | Unclear | - | Unclear | - | High | - | High |
| Farid et al. (2014) | High | High | Low | High | High | High | High | High | High |
| Feier et al. (2013) | High | - | Low | - | High | - | Low | - | High |
| Gani et al. (2015) | Low | - | High | - | High | - | Unclear | - | High |
| Giannelli et al. (2005) | High | - | Low | - | Unclear | - | Unclear | - | High |
| Gobel et al. (2006) | High | - | High | - | Unclear | - | Unclear | - | High |
| Gomaa et al. (2020) | High | - | High | - | High | - | Unclear | - | High |
| Gopal et al. (2014) | High | High | Low | High | High | High | High | High | High |
| Guan et al. (2022) | High | Low | Low | Low | High | High | High | High | High |
| Gui et al. (2011) | High | High | Low | Low | Unclear | Unclear | Unclear | Unclear | High |
| Guo et al. (2022) | High | - | Low | - | High | - | High | - | High |
| Hai et al. (2025) | High | - | Unclear | - | Unclear | - | High | - | High |
| Hallager et al. (2018) | High | - | Low | - | High | - | High | - | High |
| Hammad et al. (2023) | High | - | High | - | Low | - | High | - | High |
| Hanna et al. (2008) | High | - | Low | - | Low | - | High | - | High |
| Hassan et al. (2019) | High | High | High | High | High | High | High | High | High |
| Hawash et al. (2024) | High | - | Unclear | - | Low | - | High | - | High |
| Henry et al. (2009) | Unclear | - | High | - | High | - | Unclear | - | High |
| Hernandez et al. (2011) | Unclear | Unclear | Low | High | High | High | High | High | High |
| Hodeib et al. (2017) | High | - | High | - | High | - | High | - | High |
| Hussein et al. (2012) | High | High | Low | Low | Unclear | Unclear | Unclear | Unclear | High |
| Ibrahim et al. (2021) | High | Low | High | High | Unclear | Unclear | Unclear | Unclear | High |
| Ibrahim et al. (2025) | High | - | High | - | Low | - | High | - | High |
| Istemihan et al. (2025) | Unclear | - | High | - | Unclear | - | Low | - | High |
| Jalli et al. (2015) | High | High | Low | High | Unclear | Unclear | High | High | High |
| Jeng et al. (2014) | High | - | Low | - | High | - | High | - | High |
| Ji et al. (2021) | High | - | High | - | High | - | High | - | High |
| Jia et al. (2017) | High | High | Low | Low | Unclear | Unclear | High | High | High |
| Kamal et al. (2022) | High | High | High | High | Unclear | High | Unclear | Unclear | High |
| Khalil et al. (2024) | High | - | High | - | Unclear | - | High | - | High |
| Kim et al. (2015) | Low | - | High | - | High | - | Low | - | High |
| Kim et al. (2016) | High | High | High | High | Low | Low | Unclear | Unclear | High |
| Kim et al. (2017) | High | High | Low | Low | Low | Low | Low | Low | High |
| Kim et al. (2019) | Unclear | Unclear | Low | High | High | High | High | High | High |
| Krygier R. et al. (2011) | Unclear | - | Low | - | Unclear | - | Unclear | - | Unclear |
| Kudo et al. (2019) | High | High | High | High | High | High | Unclear | Unclear | High |
| Kurniawan et al. (2024) | Low | - | High | - | Low | - | High | - | High |
| Lambrecht et al. (2021) | High | - | High | - | High | - | Unclear | - | High |
| Lauenstein et al. (2007) | High | - | Low | - | Low | - | Low | - | High |

× HIGH RISK ? UNCLEAR RISK + LOW RISK

| | Patient selection | | Index test(s) | | Reference standard | | Flow and timing | | Overall |
|---|---|---|---|---|---|---|---|---|---|
| | QUADAS-2 | QUADAS-C | QUADAS-2 | QUADAS-C | QUADAS-2 | QUADAS-C | QUADAS-2 | QUADAS-C | |
| Leeming et al. (2021) | × | - | × | - | × | - | × | - | × |
| Lewin et al. (2021) | × | × | + | + | × | × | + | + | × |
| Li et al. (2019) | × | × | + | + | × | × | × | × | × |
| Lim et al. (2016) | × | × | + | + | ? | × | × | × | × |
| Lin et al. (2016) | × | × | + | × | × | × | × | × | × |
| Liu et al. (2007) | × | - | + | - | × | - | × | - | × |
| Loglio et al. (2019) | × | - | + | - | × | - | + | - | × |
| Loglio et al. (2020) | × | × | × | × | ? | ? | ? | ? | × |
| Ma et al. (2017) | ? | - | × | - | × | - | ? | - | × |
| Malov et al. (2021) | × | × | × | × | + | + | ? | ? | × |
| Maneenil et al. (2025) | × | - | + | - | + | - | + | - | × |
| Marrero et al. (2009) | × | × | + | × | + | + | × | × | × |
| Marsh et al. (2025) | + | - | + | - | + | - | × | - | × |
| Mittal et al. (2011) | × | - | + | - | × | - | × | - | × |
| Mohamed et al. (2020) | × | × | × | × | ? | ? | ? | ? | × |
| Mohamed et al. (2022) | × | - | × | - | × | - | ? | - | × |
| Monzawa et al. (2007) | × | - | + | - | ? | - | × | - | × |
| Mossad et al. (2014) | × | - | × | - | ? | - | × | - | × |
| Mukozu et al. (2013) | × | × | + | + | ? | ? | × | × | × |
| Nafeh et al. (2024) | + | - | × | - | + | - | × | - | × |
| Nardone et al. (2025) | × | - | × | - | + | - | + | - | × |
| Nomair et al. (2019) | × | - | × | - | × | - | ? | - | × |
| Oksuz et al. (2016) | × | - | + | - | × | - | × | - | × |
| Omran et al. (2022) | × | - | + | - | × | - | × | - | × |
| Oussalah et al. (2018) | × | - | × | - | × | - | × | - | × |
| Ozkan et al. (2011) | × | - | + | - | × | - | × | - | × |
| Pan et al. (2024) | × | - | × | - | ? | - | ? | - | × |
| Park et al. (2019) | × | + | + | × | × | × | × | × | × |
| Pascut et al. (2019) | × | ? | × | × | ? | ? | ? | ? | × |
| Patel et al. (2025) | × | - | ? | - | + | - | × | - | × |
| Pinero et al. (2015) | + | - | × | - | + | - | × | - | × |
| Pocha et al. (2013) | × | × | + | + | × | × | × | × | × |
| Qian et al. (2022) | × | × | + | + | × | × | × | × | × |
| Raafat et al. (2020) | × | - | × | - | × | - | × | - | × |
| Rashad et al. (2018) | × | × | × | × | × | × | × | × | × |
| Rashad et al. (2024) | × | - | × | - | + | - | × | - | × |
| Ricco et al. (2018) | ? | ? | + | + | + | + | × | × | × |
| Rui et al. (2025) | × | - | ? | - | + | - | × | - | × |
| Saad et al. (2020) | × | - | × | - | × | - | ? | - | × |
| Sadeghi et al. (2015) | ? | - | × | - | + | - | ? | - | × |
| Sanai et al. (2010) | × | - | + | - | + | - | × | - | × |
| Seif El Dahan et al. (2025) | × | - | + | - | ? | - | × | - | × |

× HIGH RISK ? UNCLEAR RISK + LOW RISK

| | Patient selection | | Index test(s) | | Reference standard | | Flow and timing | | Overall |
|---|---|---|---|---|---|---|---|---|---|
| | QUADAS-2 | QUADAS-C | QUADAS-2 | QUADAS-C | QUADAS-2 | QUADAS-C | QUADAS-2 | QUADAS-C | |
| Simao et al. (2015) | Low | - | High | - | High | - | Unclear | - | High |
| Singal et al. (2012) | Low | Low | High | High | High | High | High | High | High |
| Singal et al. (2022) | High | - | Low | - | Unclear | - | High | - | High |
| Song et al. (2020) | High | High | High | High | Unclear | Unclear | High | High | High |
| Sterling et al. (2009) | High | High | Low | Low | High | High | High | Unclear | High |
| Sterling et al. (2012) | Low | Low | Low | Low | High | High | High | High | High |
| Su et al. (2025) | Unclear | - | High | - | Unclear | - | Unclear | - | High |
| Sultanik et al. (2017) | High | High | Low | Low | Unclear | High | High | High | High |
| Sun et al. (2012) | High | - | Low | - | Unclear | - | Unclear | - | High |
| Tahon et al. (2019) | High | High | High | High | High | High | Unclear | Unclear | High |
| Takaya et al. (2019) | High | High | Low | Low | High | High | High | High | High |
| Tayob et al. (2018) | High | Low | Low | Low | High | Low | High | Low | High |
| Tayob et al. (2022) | High | Low | Low | Low | High | High | High | High | High |
| Toraih et al. (2018) | High | High | High | High | High | High | High | High | High |
| Toraih et al. (2019) | High | High | High | High | High | High | Unclear | Unclear | High |
| Tsai et al. (2005) | High | - | Low | - | High | - | Unclear | - | High |
| Unic et al. (2018) | Low | - | High | - | High | - | Unclear | - | High |
| Villacastin Ruiz et al. (2016) | Low | Low | Unclear | High | Low | Low | High | High | High |
| Volk et al. (2007) | High | High | High | High | Low | Low | High | High | High |
| Wang et al. (2010) | High | - | Low | - | High | - | High | - | High |
| Wang et al. (2020) | High | - | Low | - | High | - | High | - | High |
| Weis et al. (2019) | High | - | Low | - | Unclear | - | Unclear | - | High |
| Woo et al. (2025) | High | - | High | - | Low | - | Unclear | - | High |
| Worland et al. (2018) | Unclear | Low | Unclear | High | High | High | High | High | High |
| Xing et al. (2023) | Unclear | - | High | - | Low | - | High | - | High |
| Yagmur et al. (2007) | High | - | High | - | High | - | High | - | High |
| Yang et al. (2005) | High | High | High | High | Unclear | Unclear | High | High | High |
| Yang et al. (2021) | Unclear | - | Low | - | High | - | Unclear | - | High |
| Yokoo et al. (2023) | High | - | Low | - | Unclear | - | Low | - | High |
| Yoon et al. (2020) | Unclear | - | Low | - | High | - | High | - | High |
| Youssef et al. (2018) | High | - | High | - | High | - | Unclear | - | High |
| Yu et al. (2011) | High | High | Low | High | Low | Low | High | High | High |
| Zha et al. (2021) | High | - | Low | - | High | - | Unclear | - | High |
| Zhang et al. (2019) | High | - | High | - | High | - | High | - | High |
| Zhao et al. (2017) | High | - | High | - | Unclear | - | High | - | High |
| Zheng et al. (2021) | High | High | Unclear | High | High | High | High | High | High |
| Zhou et al. (2020) | High | - | Low | - | High | - | High | - | High |
| Zhu et al. (2020) | High | - | High | - | Unclear | - | Unclear | - | High |
| Zhu et al. (2025) | High | - | High | - | Low | - | Unclear | - | High |
| Zinkin et al. (2008) | High | High | Low | Low | High | High | High | High | High |

HIGH RISK UNCLEAR RISK LOW RISK

## S9.2. Summary risk of bias across studies

### S9.2.1. Weighted by number of studies

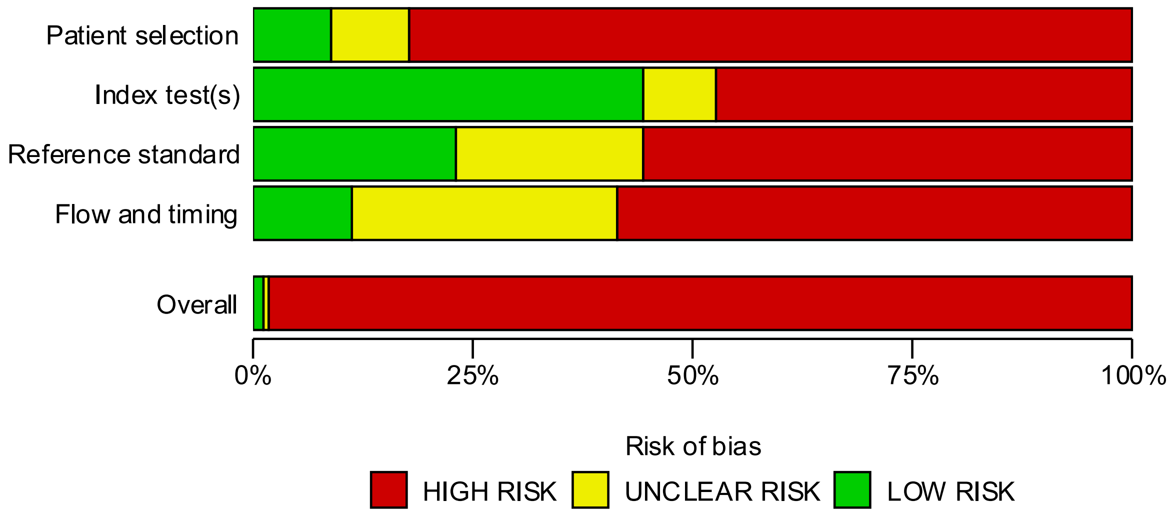


### S9.2.2. Weighted by number of participants

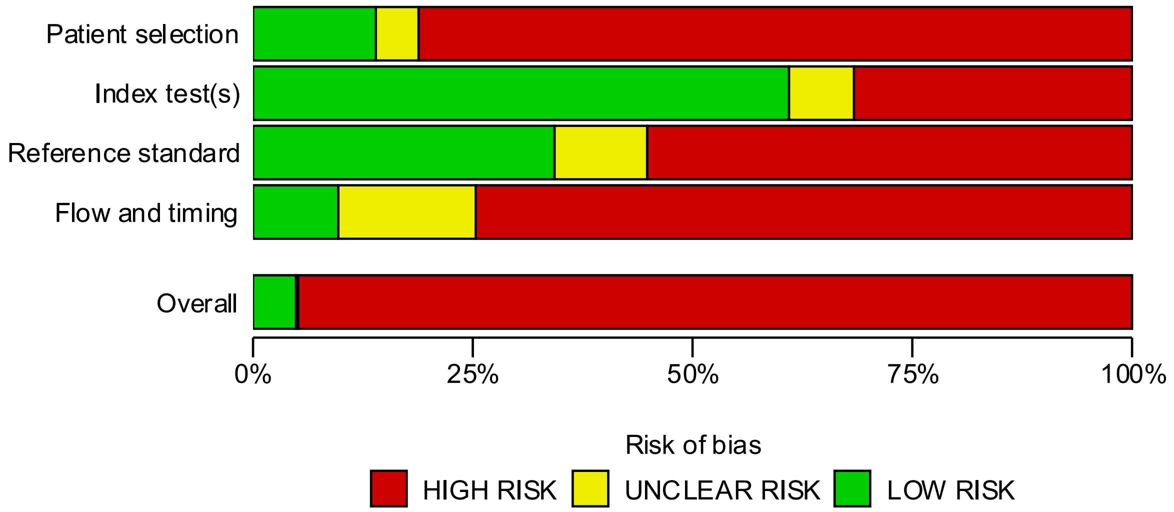

## Appendix S10 Tests not included in the common synthesis network

| Test | Study | Threshold | TP | FN | FP | TN | Sensitivity | Specificity |
|---|---|---|---|---|---|---|---|---|
| AFP mRNA | Yang et al. (2005) | | 35 | 30 | 3 | 19 | 0.538 (0.410 to 0.663) | 0.864 (0.651 to 0.971) |
| Doylestown plus algorithm | Singal et al. (2022) | | 11 | 3 | 6 | 52 | 0.786 (0.492 to 0.953) | 0.897 (0.788 to 0.961) |
| HSP90AA1 | Toraih et al. (2019) | 1.29 | 34 | 26 | 13 | 17 | 0.567 (0.432 to 0.694) | 0.567 (0.374 to 0.745) |
| HSPA1B | Toraih et al. (2019) | 1.13 | 34 | 26 | 11 | 19 | 0.567 (0.432 to 0.694) | 0.633 (0.439 to 0.801) |
| HSPA4 | Toraih et al. (2019) | 49.09 | 38 | 22 | 11 | 19 | 0.633 (0.499 to 0.754) | 0.633 (0.439 to 0.801) |
| HepaClear (model with conventional and genomic bio | Bai et al. (2023) | 0.481 | 77 | 13 | 4 | 36 | 0.856 (0.766 to 0.921) | 0.900 (0.763 to 0.972) |
| MAGE-1 mRNA | Yang et al. (2005) | | 27 | 38 | 0 | 22 | 0.415 (0.294 to 0.544) | 1.000 (0.846 to 1.000) |
| MMP-12 mRNA overexpression | Elshimi et al. (2019) | 0.7088 | 72 | 28 | 40 | 60 | 0.720 (0.621 to 0.805) | 0.600 (0.497 to 0.697) |
| MMP-12 mRNA overexpression or AFP | Elshimi et al. (2019) | | 84 | 16 | 56 | 44 | 0.840 (0.753 to 0.906) | 0.440 (0.341 to 0.543) |
| MiR-1228-3p | Pascut et al. (2019) | 0.66 | 12 | 3 | 5 | 10 | 0.800 (0.519 to 0.957) | 0.667 (0.384 to 0.882) |
| | | 26.57 | 13 | 2 | 3 | 12 | 0.867 (0.595 to 0.983) | 0.800 (0.519 to 0.957) |
| MiR-3197 | Pascut et al. (2019) | 0.45 | 13 | 2 | 4 | 11 | 0.867 (0.595 to 0.983) | 0.733 (0.449 to 0.922) |
| | | 34.69 | 13 | 2 | 4 | 11 | 0.867 (0.595 to 0.983) | 0.733 (0.449 to 0.922) |
| Model combining LSM, LS IQR, ALAT and AFP | Feier et al. (2013) | 0.5 | 16 | 5 | 6 | 13 | 0.762 (0.528 to 0.918) | 0.684 (0.434 to 0.874) |
| Multitarget Blood Test | Chalasani et al. (2022) | | 133 | 18 | 29 | 197 | 0.881 (0.818 to 0.928) | 0.872 (0.821 to 0.912) |
| TOM34 | Toraih et al. (2019) | 0.625 | 43 | 17 | 13 | 17 | 0.717 (0.586 to 0.825) | 0.567 (0.374 to 0.745) |
| miR-17-5p | Raafat et al. (2020) | 2.87 | 94 | 1 | 6 | 97 | 0.989 (0.943 to 1.000) | 0.942 (0.878 to 0.978) |
| miRNA-18b | Rashad et al. (2018) | 0.62 | 39 | 12 | 21 | 18 | 0.765 (0.625 to 0.872) | 0.462 (0.301 to 0.628) |
| miRNA-27a | Rashad et al. (2018) | 2.38 | 49 | 2 | 11 | 28 | 0.961 (0.865 to 0.995) | 0.718 (0.551 to 0.850) |
| miRNA-27a and miRNA-18b | Rashad et al. (2018) | 2.38 | 46 | 5 | 11 | 28 | 0.902 (0.786 to 0.967) | 0.718 (0.551 to 0.850) |

## Appendix S11 Characteristics of tests included in NMA-DTA analysis results

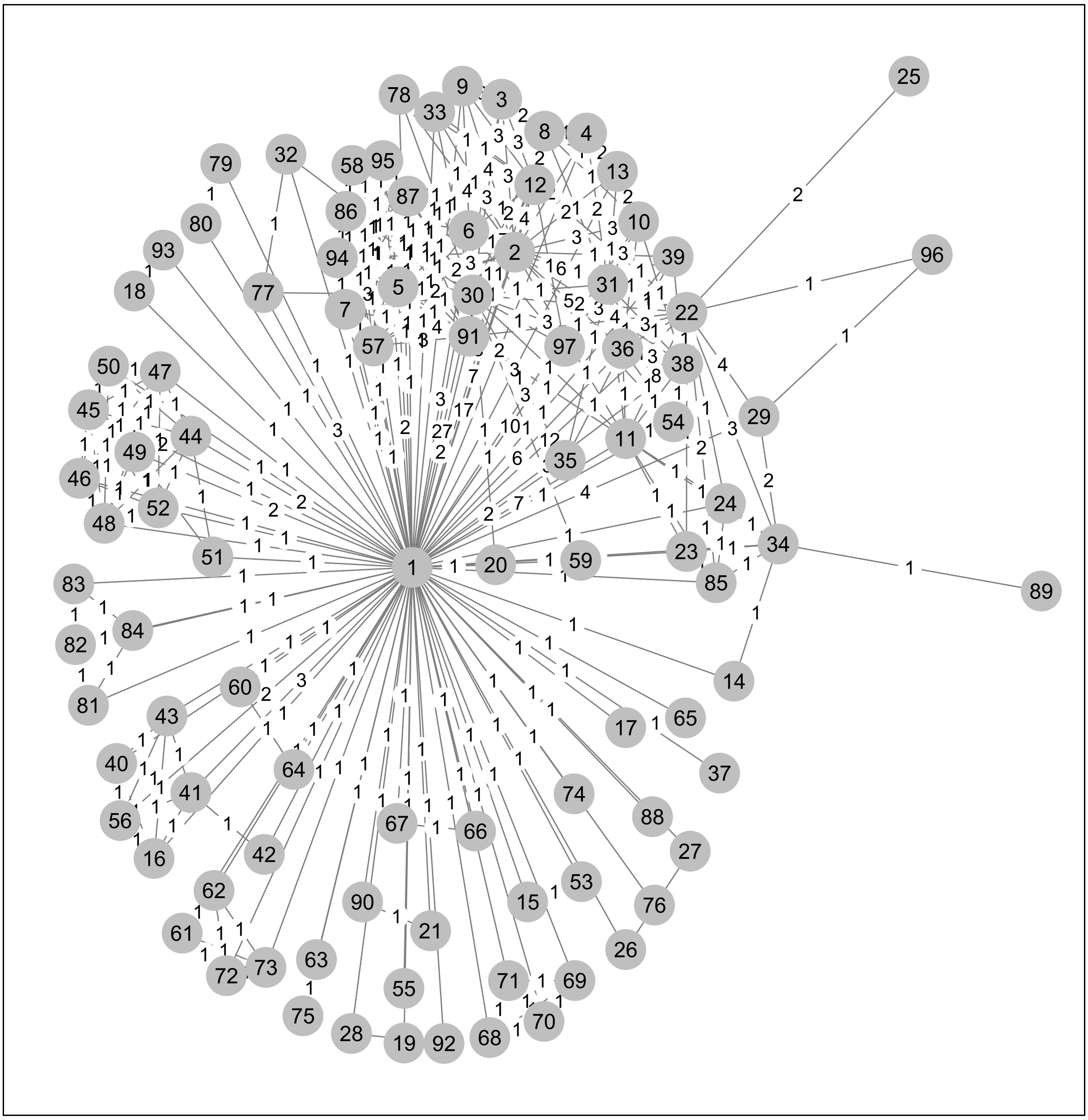

**Figure S11.1 Full connected synthesis network, comprising 97 tests.** Grey circles represent individual tests (see key below for test names). Numbers on lines show the number of studies that evaluated the particular pair of tests.

**Key for test names**: **(1)** AFP, **(2)** AFP-L3, **(3)** AFPL3 or DCP, **(4)** AFPL3 or DCP(ng/ml), **(5)** AFP and AFPL3, **(6)** AFP and DCP, **(7)** AFP and DCP and AFPL3, **(8)** AFP or AFP-L3, **(9)** AFP or AFP-L3 or DCP, **(10)** AFP or AFP-L3 or DCP (ng/ml), **(11)** AFP or B-mode US, **(12)** AFP or DCP, **(13)** AFP or DCP (ng/ml) , **(14)** AFP or DCE MRI, **(15)** AFP or Let-7a-1, **(16) AFP or MALAT lncRNA (17),** AFP or MiR-16, **(18)** AFP or TGF-b1 mRNA, **(19)** AFP progression rate, **(20)** AFP x DCP, **(21)** Alu methylation, **(22)** B-mode US, (23) B-mode US or AFP or serial AFP, (24) B-mode US or serial AFP, **(25)** CEUS, **(26)** cfDNA integrity index, **(27)** cfDNA integrity index and AFP, **(28)** Combined AFP index, (29) CT, **(30)** DCP, **(31)** DCP (ng/ml), **(32)** DCP, AFP, gender, and age, **(33)** Doylestown algorithm, **(34)** DCE MRI, (35) GAAD, **(36)** GALAD , **(37)** HCC-ART, **(38)** HES algorithm, (39) HES algorithm v2, (40) Host-2 lncRNA, **(41)** HOTAIR expression, **(42)** HOTAIR AND AFP, (43) HOXA-AS2 lncRNA, **(44)** hsa-miR-155-5p-fold, **(45)** hsa-miR-155-5p-fold and AFP , **(46)** hsa-miR-155-5p/hsa-miR-199a-5p, **(47)** hsa-miR-199a-5p fold changes, **(48)** hsa-miR-199a-5p fold changes and AFP, **(49)** hsa-miR-21-5p-fold changes, **(50)** hsa-miR-21-

5p-fold changes and AFP, (51) hsa-miR-21-5p OR hsa-miR-155-5p, **(52)** hsa-miR-21-5p/hsa-miR-199a-5p, **(53)** Let-7a-1, **(54)** Longitudinal GALAD, **(55)** MAGE-4 mRNA, **(56)** MALAT1 RNA, **(57)** mFB-I, **(58)** mFB-J, **(59)** miR-122, (60) miR-130b, **(61)** miR-133a, **(62)** miR-133a and AFP , **(63)** MiR-155 (RQ), (64) miR-15b, **(65)** miR-16, **(66)** miR-224, **(67)** miR-224 + AFP, (68) miR-23b-3p, (69) miR-3194-5p, (70) miR-331-3p, (71) miR-331-3p OR miR-23b-3p OR miR-3194-5p, **(72)** miR-483-5p, **(73)** miR-483-5p and AFP, (74) **miR-485-5p, (75)** MiR-665 (RQ), **(76)** miRNA-885-5p, **(77)** Model based on AFP and DCP, (78) Model based on age, gender, AFP and DCP, **(79)** mRNA-199-a, **(80)** mRNA-21, **(81)** mSEPT9 , **(82)** mSEPT9 and AFP , **(83)** NGS panel, **(84)** NGS panel AND AFP, **(85)** noncontrast MRI, **(86)** PEB algorithm (AFP), **(87)** PEB algorithm (DCP), **(88)** PEB algorithm or AFP, **(89)** PM-DL model , **(90)** Sat-a methylation, **(91)** serial AFP1, **(92)** Serial AFP2, **(93)** TGF-b1, **(94)** uFB (AFP), **(95)** uFB (DCP), **(96)** US and CT, **(97)** US or AFP or DCP.

### Table S11.1 Number of tests evaluated per study included in the connected synthesis network

| Number of tests | Number of studies |
|---|---|
| 1 | 75 |
| 2 | 29 |
| 3 | 29 |
| 4 | 8 |
| 5 | 4 |
| 6 | 7 |
| 7 | 3 |
| 8 | 1 |
| 9 | 2 |
| 13 | 1 |

### Table S11.2 Summary of threshold information for continuous biomarkers evaluated across multiple thresholds

| Test | Number of studies | Number of thresholds | Maximum number of thresholds in a study | Most frequently reported threshold |
|---|---|---|---|---|
| AFP (ng/mL) | 136 | 192 | 48 | 20 |
| AFP-L3 (%) | 17 | 10 | 2 | 10 |
| DCP (mAU/mL) | 29 | 87 | 50 | 40 |
| DCP (ng/ml) | 11 | 9 | 2 | 7.5 |
| GALAD | 7 | 9 | 4 | -0.63 |

**Table S11.3 Amount of evidence per test included in the analysis**

| Test | Type | Number of studies | Number of participants | | |
|---|---|---|---|---|---|
| | | | Without HCC | With HCC | Total |
| AFP | Continuous | 136 | 35,970 | 12,471 | 48,441 |
| DCP (mAU/mL) | Continuous | 29 | 10,496 | 4,784 | 15,280 |
| B-mode US | Imaging | 20 | 14,465 | 1,776 | 16,241 |
| AFP-L3 (%) | Continuous | 17 | 5,327 | 1,648 | 6,975 |
| DCP (ng/ml) | Continuous | 11 | 3,941 | 781 | 4,722 |
| Dynamic contrast-enhanced MRI | Imaging | 11 | 2,204 | 769 | 2,973 |
| AFP or B-mode US | Combination | 8 | 5,056 | 821 | 5,877 |
| CT | Imaging | 8 | 774 | 474 | 1,248 |
| AFP or DCP | Combination | 7 | 1,313 | 1,279 | 2,592 |
| GALAD | Model | 7 | 4,730 | 497 | 5,227 |
| AFP or AFP-L3 or DCP | Combination | 4 | 527 | 545 | 1,072 |
| AFP and DCP | Combination | 3 | 570 | 139 | 709 |
| AFP or AFP-L3 | Combination | 3 | 631 | 482 | 1,113 |
| AFP or AFP-L3 or DCP (ng/ml) | Combination | 3 | 841 | 130 | 971 |
| AFP or DCP (ng/ml) | Combination | 3 | 369 | 154 | 523 |
| AFP-L3 or DCP | Combination | 3 | 502 | 492 | 994 |
| Doylestown algorithm | Model | 3 | 1,758 | 862 | 2,620 |
| GAAD | Model | 3 | 1,442 | 229 | 1,671 |
| HES algorithm | Model | 3 | 3,087 | 118 | 3,205 |
| HOTAIR expression | Genomic | 3 | 131 | 137 | 268 |
| Model based on AFP and DCP | Model | 3 | 476 | 366 | 842 |
| mSEPT9 | Genomic | 3 | 294 | 158 | 452 |
| AFP and AFP-L3 | Combination | 2 | 409 | 61 | 470 |
| AFP-L3 or DCP (ng/ml) | Combination | 2 | 328 | 104 | 432 |
| CEUS | Imaging | 2 | 793 | 40 | 833 |
| hsa-miR-155-5p-fold changes | Genomic | 2 | 80 | 78 | 158 |
| hsa-miR-21-5p-fold changes | Genomic | 2 | 80 | 88 | 168 |
| MALAT1 RNA | Genomic | 2 | 80 | 90 | 170 |
| miR-122 | Genomic | 2 | 52 | 45 | 97 |
| noncontrast MRI | Imaging | 2 | 1,080 | 73 | 1,153 |
| serial AFP - absolute value AND doubling since last visit | Longitudinal | 2 | 1,592 | 383 | 1,975 |
| AFP and DCP and AFP-L3 | Combination | 1 | 358 | 20 | 378 |
| AFP or dynamic contrast-enhanced MRI | Combination | 1 | 259 | 35 | 294 |
| AFP or Let-7a-1 | Combination | 1 | 20 | 40 | 60 |
| AFP OR MALAT1 lncRNA | Genomic | 1 | 60 | 60 | 120 |
| AFP or MiR-16 | Combination | 1 | 40 | 40 | 80 |
| AFP or TGF-b1 mRNA | Combination | 1 | 20 | 20 | 40 |
| AFP progression rate (ng/ml per month) | Longitudinal | 1 | 74 | 193 | 267 |
| AFP x DCP | Combination | 1 | 1,104 | 970 | 2,074 |
| Alu methylation | Genomic | 1 | 50 | 50 | 100 |
| B-mode US AND CT | Combination | 1 | 150 | 100 | 250 |
| B-mode US OR AFP OR DCP | Combination | 1 | 148 | 64 | 212 |
| B-mode US OR absolute AFP OR serial AFP (double enrolment value) | Longitudinal | 1 | 380 | 28 | 408 |

| Test | Type | Number of studies | Number of participants | | |
|---|---|---|---|---|---|
| | | | Without HCC | With HCC | Total |
| B-mode US OR serial AFP (double enrolment value or 2 consecutive increases) | Longitudinal | 1 | 380 | 28 | 408 |
| cfDNA integrity index | Genomic | 1 | 80 | 80 | 160 |
| cfDNA integrity index and AFP | Combination | 1 | 80 | 80 | 160 |
| Combined AFP index (serial AFP and AFP) | Longitudinal | 1 | 160 | 80 | 240 |
| DCP, AFP, gender, and age | Combination | 1 | 103 | 74 | 177 |
| HCC-ART | Model | 1 | 445 | 537 | 982 |
| HES algorithm v2 | Model | 1 | 2,206 | 43 | 2,249 |
| Host-2 lncRNA | Genomic | 1 | 60 | 60 | 120 |
| HOTAIR expression AND AFP | Genomic | 1 | 40 | 40 | 80 |
| HOXA-AS2 lncRNA | Genomic | 1 | 60 | 60 | 120 |
| hsa-miR-155-5p-fold changes and AFP | Combination | 1 | 40 | 39 | 79 |
| hsa-miR-155-5p/hsa-miR-199a-5p | Genomic | 1 | 40 | 39 | 79 |
| hsa-miR-199a-5p fold changes | Genomic | 1 | 40 | 39 | 79 |
| hsa-miR-199a-5p fold changes and AFP | Combination | 1 | 40 | 39 | 79 |
| hsa-miR-21-5p OR hsa-miR-155-5p | Genomic | 1 | 40 | 39 | 79 |
| hsa-miR-21-5p-fold changes and AFP | Combination | 1 | 40 | 39 | 79 |
| hsa-miR-21-5p/hsa-miR-199a-5p | Genomic | 1 | 40 | 39 | 79 |
| Let-7a-1 | Genomic | 1 | 20 | 40 | 60 |
| Longitudinal GALAD | Longitudinal | 1 | 355 | 42 | 397 |
| MAGE-4 mRNA | Genomic | 1 | 15 | 30 | 45 |
| mFB-I (independent multivariate Bayesian screening algorithm) | Model | 1 | 361 | 48 | 409 |
| mFB-J (joint multivariate Bayesian screening algorithm) | Model | 1 | 361 | 48 | 409 |
| miR-130b | Genomic | 1 | 35 | 70 | 105 |
| miR-133a | Genomic | 1 | 20 | 20 | 40 |
| miR-133a and AFP | Combination | 1 | 20 | 20 | 40 |
| MiR-155 (RQ) | Genomic | 1 | 80 | 80 | 160 |
| miR-15b | Genomic | 1 | 35 | 70 | 105 |
| miR-16 | Genomic | 1 | 40 | 40 | 80 |
| miR-224 | Genomic | 1 | 39 | 96 | 135 |
| miR-224 + AFP | Combination | 1 | 39 | 96 | 135 |
| miR-23b-3p | Genomic | 1 | 50 | 50 | 100 |
| miR-3194-5p | Genomic | 1 | 50 | 50 | 100 |
| miR-331-3p | Genomic | 1 | 50 | 50 | 100 |
| miR-331-3p OR miR-23b-3p OR miR-3194-5p | Genomic | 1 | 50 | 50 | 100 |
| miR-483-5p | Genomic | 1 | 20 | 20 | 40 |
| miR-483-5p and AFP | Combination | 1 | 20 | 20 | 40 |
| miR-485-5p | Genomic | 1 | 50 | 50 | 100 |
| MiR-665 (RQ) | Genomic | 1 | 80 | 80 | 160 |
| miRNA-885-5p | Genomic | 1 | 26 | 46 | 72 |
| Model based on age, gender, AFP and DCP | Model | 1 | 412 | 416 | 828 |
| mRNA-199-a | Genomic | 1 | 17 | 23 | 40 |
| mRNA-21 | Genomic | 1 | 17 | 23 | 40 |
| mSEPT9 and AFP | Combination | 1 | 103 | 60 | 163 |
| NGS panel | Genomic | 1 | 103 | 60 | 163 |
| NGS panel AND AFP | Genomic | 1 | 103 | 60 | 163 |

| Test | Type | Number of studies | Number of participants | | |
|---|---|---|---|---|---|
| | | | Without HCC | With HCC | Total |
| PEB algorithm (AFP) | Model | 1 | 361 | 42 | 403 |
| PEB algorithm (DCP) | Model | 1 | 361 | 48 | 409 |
| PEB algorithm or AFP | Model | 1 | 5,611 | 365 | 5,976 |
| PM-DL model | Model | 1 | 32 | 32 | 64 |
| Sat-a methylation | Genomic | 1 | 50 | 50 | 100 |
| Serial AFP – any increase | Longitudinal | 1 | 160 | 80 | 240 |
| TGF-b1 | Genomic | 1 | 20 | 20 | 40 |
| uFB (AFP) (univariate Bayesian screening algorithm) | Model | 1 | 361 | 48 | 409 |
| uFB (DCP) (univariate Bayesian screening algorithm) | Model | 1 | 361 | 48 | 409 |

## Appendix S12 Network meta-analysis and meta-analysis of the accuracy of each test separately

**Table S12.1 Comparison of pooled estimates of the sensitivity and specificity of each test, with 95% credible intervals (CrIs).** Based on (1) meta-analysis of the accuracy of each test separately, and (2) the full network meta-analysis model (results reported in the main text). For the continuous biomarkers we report the sensitivity for the threshold that corresponds 90% specificity. The threshold reported for combinations of continuous biomarkers corresponds to the threshold of the first biomarker in the combination.

| Test | No. of studies | Threshold | (1) Results from meta-analysis of each test separately | | | | (2) Results from network meta-analysis of diagnostic test accuracy | | | |
|---|---|---|---|---|---|---|---|---|---|---|
| | | | Sensitivity | | Specificity | | Sensitivity | | Specificity | |
| | | | Estimate | 95%CrI | Estimate | 95%CrI | Estimate | 95%CrI | Estimate | 95%CrI |
| AFP | 136 | 20 | 0.63 | (0.60,0.67) | 0.89 | (0.86,0.91) | 0.60 | (0.57,0.64) | 0.90 | (0.88,0.92) |
| DCP (mAU/mL) | 29 | 40 | 0.74 | (0.64,0.83) | 0.79 | (0.64,0.90) | 0.75 | (0.68,0.81) | 0.76 | (0.67,0.84) |
| B-mode US | 20 | - | 0.62 | (0.50,0.72) | 0.96 | (0.92,0.99) | 0.70 | (0.62,0.78) | 0.94 | (0.90,0.97) |
| AFP-L3 (%) | 17 | 10 | 0.45 | (0.30,0.56) | 0.97 | (0.88,1.00) | 0.52 | (0.42,0.62) | 0.93 | (0.89,0.96) |
| DCP (ng/ml) | 11 | 7.5 | 0.56 | (0.31,0.78) | 0.94 | (0.79,0.99) | 0.57 | (0.43,0.71) | 0.92 | (0.85,0.96) |
| DCE MRI | 11 | - | 0.84 | (0.80,0.89) | 0.93 | (0.86,0.97) | 0.90 | (0.83,0.94) | 0.92 | (0.85,0.96) |
| AFP or B-mode US | 8 | 20 | 0.89 | (0.51,0.98) | 0.93 | (0.59,1.00) | 0.90 | (0.79,0.96) | 0.89 | (0.77,0.95) |
| CT | 8 | - | 0.88 | (0.67,0.98) | 0.92 | (0.80,0.98) | 0.89 | (0.79,0.96) | 0.93 | (0.85,0.97) |
| AFP or DCP | 7 | 20 | 0.86 | (0.76,0.93) | 0.78 | (0.60,0.90) | 0.87 | (0.80,0.92) | 0.77 | (0.62,0.88) |
| GALAD | 7 | -0.63 | 0.58 | (0.50,0.70) | 0.71 | (0.56,0.82) | 0.80 | (0.71,0.87) | 0.78 | (0.63,0.88) |
| AFP or AFP-L3 or DCP | 4 | 20 | 0.89 | (0.23,0.99) | 0.91 | (0.31,1.00) | 0.88 | (0.70,0.96) | 0.88 | (0.64,0.97) |
| AFP-L3 or DCP | 3 | 10 | 0.76 | (0.72,0.80) | 0.87 | (0.83,0.90) | 0.84 | (0.65,0.95) | 0.83 | (0.55,0.95) |
| AFP and DCP | 3 | 20 | 0.38 | (0,06,0.84) | 1.00 | (0.84,1.00) | 0.54 | (0.32,0.74) | 0.98 | (0.92,1.00) |
| AFP or AFP-L3 | 3 | 20 | 0.70 | (0.62,0.78) | 0.68 | (0.63,0.73) | 0.73 | (0.49,0.89) | 0.73 | (0.41,0.91) |
| AFP or AFP-L3 or DCP (ng/ml) | 3 | 20 | 0.68 | (0.09,0.98) | 0.75 | (0.17,0.98) | 0.76 | (0.53,0.89) | 0.75 | (0.50,0.90) |
| AFP or DCP (ng/ml) | 3 | 20 | 0.86 | (0.28,0.99) | 0.69 | (0.27,0.94) | 0.83 | (0.65,0.94) | 0.74 | (0.48,0.90) |
| Doylestown algorithm | 3 | | 0.36 | (0.01,0.93) | 0.95 | (0.86,0.98) | 0.50 | (0.23,0.70) | 0.94 | (0.84,0.98) |
| GAAD | 3 | | 0.81 | (0.08,0.99) | 0.65 | (0.03,0.99) | 0.78 | (0.49,0.92) | 0.78 | (0.43,0.94) |

| Test | No. of studies | Threshold | (1) Results from meta-analysis of each test separately | | | | (2) Results from network meta-analysis of diagnostic test accuracy | | | |
|---|---|---|---|---|---|---|---|---|---|---|
| | | | Sensitivity | | Specificity | | Sensitivity | | Specificity | |
| | | | Estimate | 95%CrI | Estimate | 95%CrI | Estimate | 95%CrI | Estimate | 95%CrI |
| HES algorithm | 3 | | 0.37 | (0.08,0.79) | 0.91 | (0.67,0.98) | 0.53 | (0.33,0.72) | 0.92 | (0.79,0.97) |
| HOTAIR expression | 3 | | 0.81 | (0.24,0.98) | 0.90 | (0.27,1.00) | 0.75 | (0.51,0.90) | 0.87 | (0.62,0.97) |
| Model based on AFP and DCP | 3 | | 0.73 | (0.35,0.94) | 0.87 | (0.39,0.99) | 0.74 | (0.56,0.87) | 0.87 | (0.68,0.95) |
| mSEPT9 | 3 | | 0.93 | (0.33,1.00) | 0.68 | (0.35,0.89) | 0.89 | (0.75,0.96) | 0.77 | (0.45,0.93) |
| AFP-L3 or DCP (ng/ml) | 2 | 10 | 0.66 | (0.57,0.75) | 0.84 | (0.80,0.88) | 0.68 | (0.42,0.86) | 0.94 | (0.78,0.99) |
| AFP and AFP-L3 | 2 | 20 | 0.48 | (0.36,0.60) | 0.97 | (0.96,0.99) | 0.43 | (0.18,0.71) | 0.99 | (0.94,1.00) |
| CEUS | 2 | - | 0.89 | (0.77,0.96) | 0.98 | (0.97,0.99) | 0.94 | (0.65,1.00) | 0.98 | (0.89,1.00) |
| hsa-miR-155-5p-fold changes | 2 | | 0.71 | (0.61,0.81) | 0.48 | (0.37,0.58) | 0.68 | (0.40,0.88) | 0.34 | (0.09,0.71) |
| hsa-miR-21-5p-fold changes | 2 | | 0.77 | (0.67,0.85) | 0.45 | (0.35,0.56) | 0.74 | (0.46,0.90) | 0.31 | (0.08,0.69) |
| MALAT1 RNA | 2 | | 0.84 | (0.76,0.91) | 0.82 | (0.73,0.89) | 0.67 | (0.12,0.97) | 0.78 | (0.40,0.95) |
| miR-122 | 2 | | 0.66 | (0.52,0.79) | 0.84 | (0.72,0.92) | 0.69 | (0.35,0.92) | 0.88 | (0.57,0.98) |
| noncontrast MRI | 2 | - | 0.80 | (0.70,0.88) | 0.98 | (0.97,0.99) | 0.92 | (0.75,0.97) | 0.98 | (0.90,1.00) |
| serial AFP - absolute value above threshold AND do | 2 | | 0.95 | (0.93,0.97) | 0.76 | (0.73,0.78) | 0.86 | (0.21,0.99) | 0.92 | (0.73,0.98) |
| AFP and DCP and AFP-L3 | 1 | 20 | 0.20 | (0.06,0.44) | 0.99 | (0.98,1.00) | 0.31 | (0.05,0.77) | 0.99 | (0.96,1.00) |
| AFP or DCE MRI | 1 | | 0.91 | (0.77,0.98) | 0.93 | (0.90,0.96) | 0.97 | (0.76,1.00) | 0.86 | (0.44,0.98) |
| AFP or Let-7a-1 | 1 | | 0.95 | (0.83,0.99) | 1.00 | (0.83,1.00) | 0.90 | (0.45,0.99) | 1.00 | (0.93,1.00) |
| AFP OR MALAT1 lncRNA | 1 | | 0.98 | (0.91,1.00) | 1.00 | (0.94,1.00) | 0.97 | (0.74,1.00) | 1.00 | (0.94,1.00) |
| AFP or MiR-16 | 1 | | 0.85 | (0.70,0.94) | 0.90 | (0.76,0.97) | 0.87 | (0.47,0.98) | 0.79 | (0.24,0.98) |
| AFP or TGF-b1 mRNA | 1 | | 0.80 | (0.56,0.94) | 0.75 | (0.51,0.91) | 0.83 | (0.35,0.98) | 0.65 | (0.14,0.95) |
| AFP progression rate (ng/ml per month) | 1 | | 0.67 | (0.60,0.73) | 1.00 | (0.95,1.00) | 0.63 | (0.22,0.92) | 1.00 | (0.97,1.00) |
| AFP x DCP | 1 | | 0.60 | (0.57,0.63) | 0.60 | (0.57,0.63) | 0.68 | (0.22,0.95) | 0.64 | (0.18,0.93) |
| Alu methylation | 1 | | 0.66 | (0.51,0.79) | 0.62 | (0.47,0.75) | 0.59 | (0.17,0.91) | 0.60 | (0.14,0.93) |
| B-mode US OR AFP OR serial AFP (double enrolment | 1 | | 0.64 | (0.44,0.81) | 0.82 | (0.78,0.86) | 0.87 | (0.49,0.98) | 0.69 | (0.25,0.94) |
| B-mode US OR serial AFP (double enrolment or 2 co | 1 | | 0.64 | (0.44,0.81) | 0.84 | (0.80,0.87) | 0.87 | (0.48,0.98) | 0.72 | (0.27,0.95) |

| Test | No. of studies | Threshold | (1) Results from meta-analysis of each test separately | | | | (2) Results from network meta-analysis of diagnostic test accuracy | | | |
|---|---|---|---|---|---|---|---|---|---|---|
| | | | Sensitivity | | Specificity | | Sensitivity | | Specificity | |
| | | | Estimate | 95%CrI | Estimate | 95%CrI | Estimate | 95%CrI | Estimate | 95%CrI |
| cfDNA integrity index | 1 | | 0.85 | (0.75,0.92) | 0.98 | (0.91,1.00) | 0.81 | (0.37,0.97) | 0.96 | (0.62,1.00) |
| cfDNA integrity index and AFP | 1 | | 0.95 | (0.88,0.99) | 0.95 | (0.88,0.99) | 0.94 | (0.65,0.99) | 0.91 | (0.47,0.99) |
| Combined AFP index (serial AFP and AFP) | 1 | | 0.80 | (0.70,0.88) | 0.62 | (0.55,0.70) | 0.81 | (0.39,0.97) | 0.62 | (0.16,0.93) |
| DCP, AFP, gender, and age | 1 | | 0.89 | (0.80,0.95) | 0.93 | (0.86,0.97) | 0.85 | (0.45,0.98) | 0.89 | (0.49,0.99) |
| HCC-ART | 1 | | 0.90 | (0.87,0.92) | 0.83 | (0.79,0.86) | 0.85 | (0.44,0.98) | 0.85 | (0.37,0.98) |
| HES algorithm v2 | 1 | | 0.53 | (0.38,0.69) | 0.90 | (0.89,0.91) | 0.78 | (0.35,0.96) | 0.78 | (0.33,0.96) |
| Host-2 lncRNA | 1 | | 0.88 | (0.77,0.95) | 0.78 | (0.66,0.88) | 0.78 | (0.32,0.96) | 0.63 | (0.16,0.94) |
| HOTAIR expression AND AFP | 1 | | 0.80 | (0.64,0.91) | 0.98 | (0.87,1.00) | 0.77 | (0.31,0.96) | 0.97 | (0.69,1.00) |
| HOXA-AS2 lncRNA | 1 | | 0.88 | (0.77,0.95) | 0.70 | (0.57,0.81) | 0.78 | (0.32,0.96) | 0.52 | (0.11,0.90) |
| hsa-miR-155-5p-fold changes and AFP | 1 | | 0.82 | (0.66,0.92) | 0.78 | (0.62,0.89) | 0.79 | (0.34,0.97) | 0.67 | (0.18,0.95) |
| hsa-miR-155-5p/hsa-miR-199a-5p | 1 | | 0.92 | (0.79,0.98) | 0.52 | (0.36,0.68) | 0.91 | (0.56,0.99) | 0.39 | (0.07,0.85) |
| hsa-miR-199a-5p fold changes | 1 | | 0.79 | (0.64,0.91) | 0.45 | (0.29,0.62) | 0.76 | (0.30,0.96) | 0.32 | (0.05,0.80) |
| hsa-miR-199a-5p fold changes and AFP | 1 | | 0.85 | (0.69,0.94) | 0.80 | (0.64,0.91) | 0.82 | (0.39,0.97) | 0.70 | (0.20,0.96) |
| hsa-miR-21-5p-fold changes and AFP | 1 | | 0.87 | (0.73,0.96) | 0.78 | (0.62,0.89) | 0.85 | (0.43,0.98) | 0.67 | (0.18,0.95) |
| hsa-miR-21-5p OR hsa-miR-155-5p | 1 | | 0.85 | (0.69,0.94) | 0.45 | (0.29,0.62) | 0.83 | (0.40,0.97) | 0.31 | (0.05,0.79) |
| hsa-miR-21-5p/hsa-miR-199a-5p | 1 | | 0.95 | (0.83,0.99) | 0.48 | (0.32,0.64) | 0.95 | (0.65,1.00) | 0.34 | (0.05,0.82) |
| Let-7a-1 | 1 | | 0.70 | (0.53,0.83) | 0.80 | (0.56,0.94) | 0.47 | (0.11,0.87) | 0.84 | (0.29,0.99) |
| Longitudinal GALAD | 1 | | 0.64 | (0.48,0.78) | 0.90 | (0.87,0.93) | 0.73 | (0.28,0.94) | 0.91 | (0.62,0.99) |
| MAGE-4 mRNA | 1 | | 0.30 | (0.15,0.49) | 1.00 | (0.78,1.00) | 0.12 | (0.02,0.52) | 1.00 | (0.96,1.00) |
| mFB-I (independent multivariate fully Bayesian scr | 1 | | 0.60 | (0.45,0.74) | 0.90 | (0.86,0.93) | 0.74 | (0.31,0.95) | 0.93 | (0.65,0.99) |
| mFB-J (joint multivariate fully Bayesian screening | 1 | | 0.60 | (0.45,0.74) | 0.90 | (0.86,0.93) | 0.74 | (0.30,0.95) | 0.93 | (0.66,0.99) |
| miR-130b | 1 | | 0.80 | (0.69,0.89) | 0.77 | (0.60,0.90) | 0.71 | (0.25,0.94) | 0.84 | (0.34,0.98) |
| miR-133a | 1 | | 0.70 | (0.46,0.88) | 0.90 | (0.68,0.99) | 0.58 | (0.14,0.92) | 0.89 | (0.37,0.99) |
| miR-133a and AFP | 1 | | 0.90 | (0.68,0.99) | 1.00 | (0.83,1.00) | 0.86 | (0.37,0.99) | 1.00 | (0.90,1.00) |

| Test | No. of studies | Threshold | (1) Results from meta-analysis of each test separately | | | | (2) Results from network meta-analysis of diagnostic test accuracy | | | |
|---|---|---|---|---|---|---|---|---|---|---|
| | | | Sensitivity | | Specificity | | Sensitivity | | Specificity | |
| | | | Estimate | 95%CrI | Estimate | 95%CrI | Estimate | 95%CrI | Estimate | 95%CrI |
| MiR-155 (RQ) | 1 | | 0.80 | (0.70,0.88) | 0.62 | (0.51,0.73) | 0.83 | (0.42,0.97) | 0.71 | (0.22,0.95) |
| miR-15b | 1 | | 0.77 | (0.66,0.86) | 0.97 | (0.85,1.00) | 0.67 | (0.21,0.93) | 0.99 | (0.81,1.00) |
| miR-16 | 1 | | 0.58 | (0.41,0.73) | 0.70 | (0.53,0.83) | 0.61 | (0.17,0.92) | 0.47 | (0.08,0.90) |
| miR-224 | 1 | | 0.86 | (0.78,0.93) | 0.67 | (0.50,0.81) | 0.86 | (0.46,0.97) | 0.73 | (0.24,0.96) |
| miR-224 + AFP | 1 | | 0.97 | (0.91,0.99) | 0.64 | (0.47,0.79) | 0.97 | (0.79,1.00) | 0.71 | (0.22,0.96) |
| miR-23b-3p | 1 | | 0.74 | (0.60,0.85) | 0.66 | (0.51,0.79) | 0.62 | (0.18,0.92) | 0.85 | (0.39,0.98) |
| miR-3194-5p | 1 | | 0.82 | (0.69,0.91) | 0.74 | (0.60,0.85) | 0.72 | (0.26,0.95) | 0.90 | (0.48,0.99) |
| miR-331-3p | 1 | | 0.86 | (0.73,0.94) | 0.56 | (0.41,0.70) | 0.78 | (0.32,0.96) | 0.79 | (0.31,0.97) |
| miR-331-3p OR miR-23b-3p OR miR-3194-5p | 1 | | 0.90 | (0.78,0.97) | 0.60 | (0.45,0.74) | 0.84 | (0.41,0.98) | 0.82 | (0.34,0.98) |
| miR-483-5p | 1 | | 1.00 | (0.83,1.00) | 0.75 | (0.51,0.91) | 1.00 | (0.89,1.00) | 0.71 | (0.17,0.97) |
| miR-483-5p and AFP | 1 | | 0.95 | (0.75,1.00) | 0.95 | (0.75,1.00) | 0.94 | (0.52,1.00) | 0.96 | (0.52,1.00) |
| miR-485-5p | 1 | | 0.92 | (0.81,0.98) | 0.84 | (0.71,0.93) | 0.89 | (0.49,0.99) | 0.95 | (0.65,0.99) |
| MiR-665 (RQ) | 1 | | 0.92 | (0.84,0.97) | 0.86 | (0.77,0.93) | 0.94 | (0.67,0.99) | 0.91 | (0.51,0.99) |
| miRNA-885-5p | 1 | | 1.00 | (0.92,1.00) | 0.00 | (0.00,0.13) | 1.00 | (0.97,1.00) | 0.00 | (0.00,0.06) |
| Model based on age, gender, AFP and DCP | 1 | | 0.75 | (0.71,0.79) | 0.84 | (0.80,0.87) | 0.75 | (0.31,0.94) | 0.86 | (0.48,0.98) |
| mRNA-199-a | 1 | | 0.57 | (0.34,0.77) | 1.00 | (0.80,1.00) | 0.34 | (0.06,0.82) | 1.00 | (0.93,1.00) |
| mRNA-21 | 1 | | 1.00 | (0.85,1.00) | 0.82 | (0.57,0.96) | 1.00 | (0.86,1.00) | 0.87 | (0.35,0.99) |
| mSEPT9 and AFP | 1 | | 0.42 | (0.29,0.55) | 0.97 | (0.92,0.99) | 0.59 | (0.18,0.90) | 0.97 | (0.75,1.00) |
| NGS panel | 1 | | 0.58 | (0.45,0.71) | 0.97 | (0.92,0.99) | 0.74 | (0.27,0.95) | 0.97 | (0.75,1.00) |
| NGS panel AND AFP | 1 | | 0.68 | (0.55 0.80) | 0.97 | (0.92,0.99) | 0.81 | (0.39,0.97) | 0.97 | (0.75,1.00) |
| PEB algorithm (AFP) | 1 | | 0.69 | (0.53,0.82) | 0.90 | (0.86,0.93) | 0.81 | (0.38,0.97) | 0.93 | (0.65,0.99) |
| PEB algorithm (DCP) | 1 | | 0.60 | (0.45,0.74) | 0.90 | (0.86,0.93) | 0.74 | (0.29,0.95) | 0.93 | (0.66,0.99) |
| PEB algorithm or AFP | 1 | | 0.56 | (0.50,0.61) | 0.95 | (0.94,0.96) | 0.52 | (0.15,0.87) | 0.96 | (0.75,1.00) |
| PM-DL model | 1 | | 0.94 | (0.79,0.99) | 1.00 | (0.89,1.00) | 0.96 | (0.70,1.00) | 1.00 | (0.89,1.00) |

| Test | No. of studies | Threshold | (1) Results from meta-analysis of each test separately | | | | (2) Results from network meta-analysis of diagnostic test accuracy | | | |
|---|---|---|---|---|---|---|---|---|---|---|
| | | | Sensitivity | | Specificity | | Sensitivity | | Specificity | |
| | | | Estimate | 95%CrI | Estimate | 95%CrI | Estimate | 95%CrI | Estimate | 95%CrI |
| Sat-a methylation | 1 | | 1.00 | (0.93,1.00) | 0.84 | (0.71,0.93) | 1.00 | (0.96,1.00) | 0.83 | (0.34,0.98) |
| Serial AFP - any increase | 1 | | 0.68 | (0.56,0.78) | 0.71 | (0.63,0.78) | 0.69 | (0.24,0.94) | 0.70 | (0.22,0.95) |
| TGF-b1 | 1 | | 0.65 | (0.41,0.85) | 0.75 | (0.51,0.91) | 0.69 | (0.21,0.95) | 0.65 | (0.14,0.96) |
| uFB (AFP) (univariate fully Bayesian screening algorithm) | 1 | | 0.81 | (0.67,0.91) | 0.90 | (0.86,0.93) | 0.89 | (0.55,0.98) | 0.93 | (0.65,0.99) |
| uFB (DCP) (univariate fully Bayesian screening algorithm) | 1 | | 0.71 | (0.56,0.83) | 0.90 | (0.86,0.93) | 0.82 | (0.40,0.97) | 0.93 | (0.65,0.99) |
| US and CT | 1 | | 0.88 | (0.80,0.94) | 0.96 | (0.91,0.99) | 0.88 | (0.49,0.98) | 0.94 | (0.66,0.99) |
| US or AFP or DCP | 1 | | 0.89 | (0.79,0.95) | 0.88 | (0.81,0.93) | 0.96 | (0.76,0.99) | 0.52 | (0.12,0.90) |

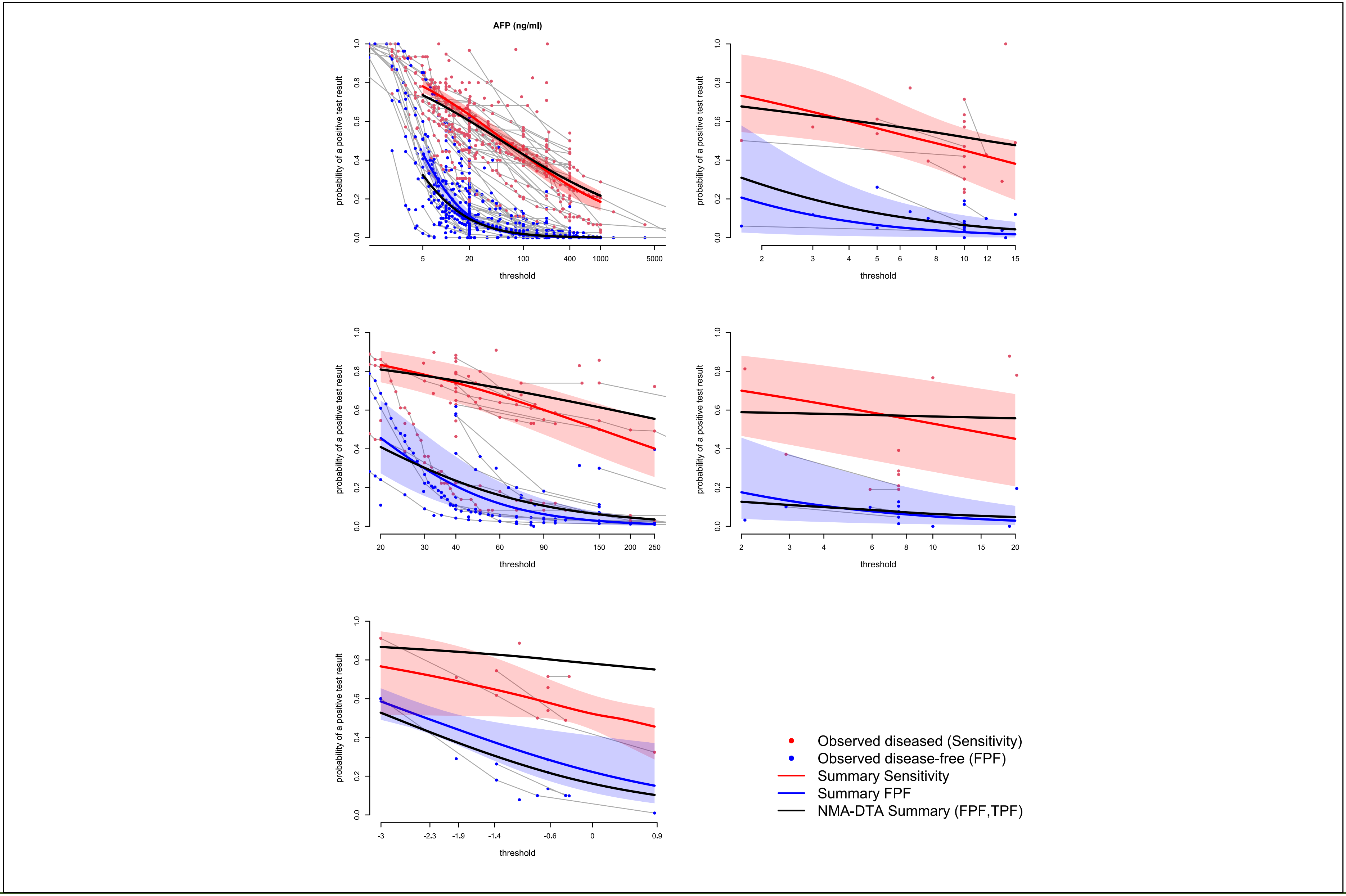
AFP (ng/ml)
probability of a positive test result
threshold
Observed diseased (Sensitivity)
Observed disease-free (FPF)
Summary Sensitivity
Summary FPF
NMA-DTA Summary (FPF,TPF)

**Figure S12.1 True positive fraction (TPF = sensitivity) and false positive fraction (FPF = 1 – specificity) of continuous biomarkers across thresholds.** Red and blue lines, and shaded regions, represent summary estimates with 95% CrIs from separate meta-analyses of the accuracy of each test. Black lines represent summary estimates from the NMA-DTA (as shown in Figure 3 of the main text). Circles represent observed study-specific estimates, with estimates from the same study linked by grey lines. First row shows AFP and AFP-L3. Second row shows DCP and DCP (ng/ml), and third row shows GALAD.

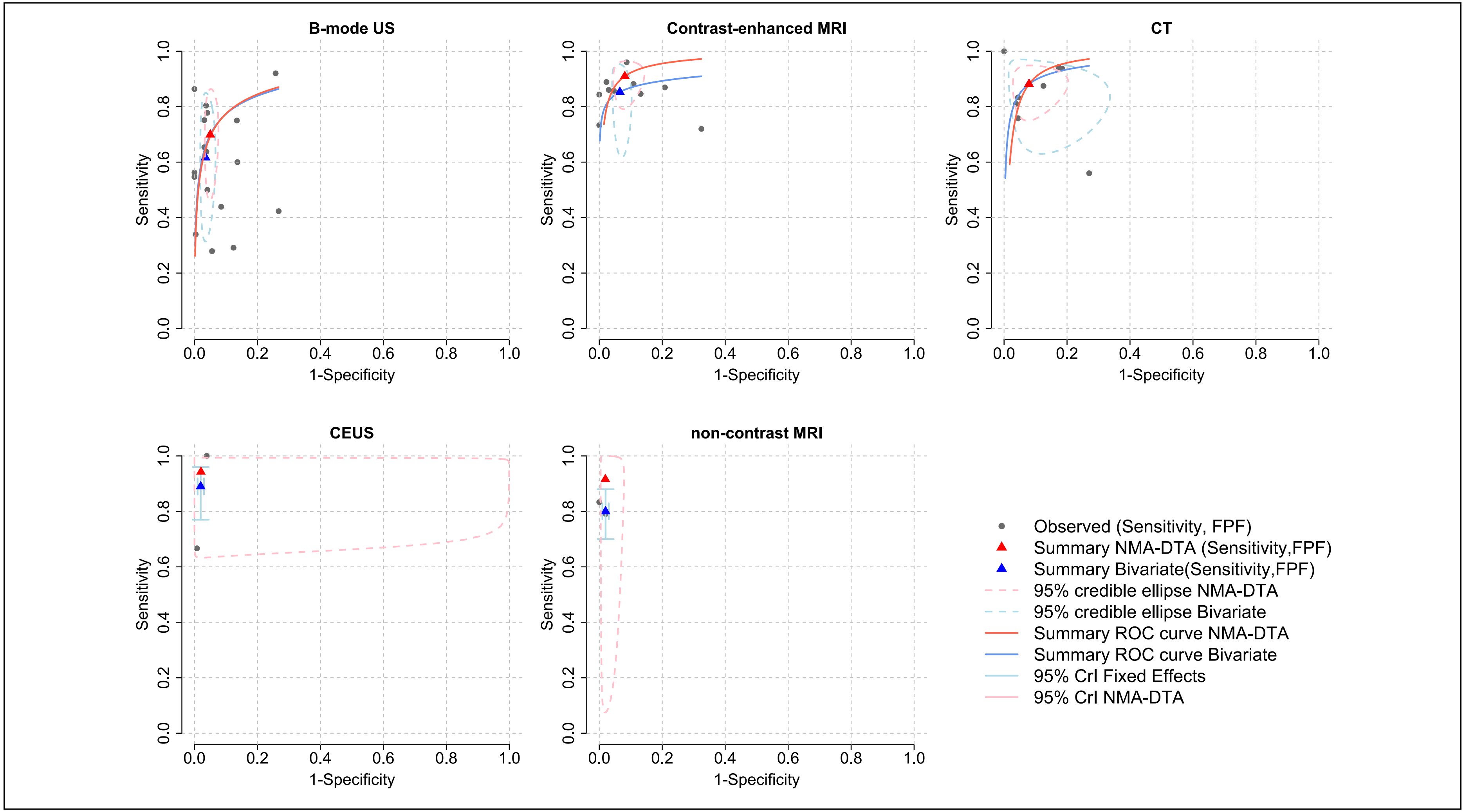


**Figure S12.2 Pooled estimates of the sensitivity and specificity of imaging tests, with 95% credible ellipses and hierarchical summary ROC (HSROC) curves.** RED=Results from NMA-DTA, BLUE=Results from meta-analyses of each test separately. Observed study-specific estimates are also shown. For tests reported in 2 studies separate meta-analysis results are based on fixed effect meta-analysis and 95% credible intervals are shown instead of ellipses.

# Appendix S13 Network meta-analysis of diagnostic test accuracy results

## S13.1. Ranking estimates

For continuous tests, we calculate ranks at the most commonly reported threshold.

**Table S13.1 Estimated ranks of tests by (1) sensitivity and (2) specificity, with 95% credible intervals (CrIs). Only tests evaluated in at least two studies were ranked across.**

| Sensitivity | | | Specificity | | |
|---|---|---|---|---|---|
| Test | Estimated rank | 95%CrI | Test | Estimated rank | 95%CrI |
| CEUS | 2 | (1,22) | AFP and AFP-L3 | 2 | (1,8) |
| noncontrast MRI | 4 | (1,16) | AFP and DCP | 3 | (1,11) |
| AFP or B-mode US | 5 | (1,14) | CEUS | 3 | (1,16) |
| dynamic contrast-enhanced MRI | 5 | (2,11) | noncontrast MRI | 3 | (1,14) |
| CT | 6 | (1,14) | AFP-L3 or DCP (ng/ml) | 7 | (1,23) |
| mSEPT9 | 6 | (1,17) | B-mode US | 8 | (4,14) |
| AFP or AFP-L3 or DCP | 7 | (1,20) | Doylestown algorithm | 8 | (3,20) |
| AFP or DCP | 8 | (3,13) | AFP-L3 (%) | 9 | (1,23) |
| serial AFP - absolute value above threshold AND do | 9 | (1,31) | CT | 11 | (4,19) |
| AFP-L3 or DCP | 10 | (2,22) | DCP (ng/ml) | 11 | (4,19) |
| AFP or DCP (ng/ml) | 11 | (2,22) | dynamic contrast-enhanced MRI | 11 | (5,19) |
| GALAD | 13 | (7,19) | HES algorithm | 12 | (4,22) |
| GAAD | 15 | (3,28) | serial AFP - absolute value above threshold AND do | 12 | (3,26) |
| AFP or AFP-L3 or DCP (ng/ml) | 16 | (6,27) | AFP | 14 | (10,18) |
| DCP (mAU/mL) | 16 | (11,22) | AFP or B-mode US | 16 | (6,24) |
| HOTAIR expression | 16 | (5,28) | AFP or AFP-L3 or DCP | 17 | (4,28) |
| hsa-miR-21-5p-fold changes | 17 | (5,29) | miR-122 | 17 | (3,29) |
| Model based on AFP and DCP | 17 | (8,26) | HOTAIR expression | 18 | (4,28) |
| AFP or AFP-L3 | 18 | (7,28) | Model based on AFP and DCP | 18 | (6,27) |
| B-mode US | 19 | (14,24) | AFP-L3 or DCP | 20 | (7,29) |
| miR-122 | 20 | (4,31) | AFP or DCP | 23 | (17,29) |
| AFP-L3 or DCP (ng/ml) | 21 | (9,30) | GAAD | 23 | (8,30) |
| hsa-miR-155-5p-fold changes | 21 | (7,30) | GALAD | 23 | (16,29) |
| MALAT1 RNA | 21 | (1,31) | MALAT1 RNA | 23 | (7,30) |
| AFP | 24 | (21,27) | DCP (mAU/mL) | 24 | (19,28) |
| DCP (ng/ml) | 26 | (19,30) | mSEPT9 | 24 | (10,30) |
| AFP and DCP | 27 | (27,17) | AFP or AFP-L3 or DCP (ng/ml) | 25 | (15,29) |
| HES algorithm | 27 | (19,31) | AFP or DCP (ng/ml) | 25 | (15,30) |

| Sensitivity | | | Specificity | | |
|---|---|---|---|---|---|
| Test | Estimated rank | 95%CrI | Test | Estimated rank | 95%CrI |
| AFP-L3 (%) | 28 | (23,31) | AFP or AFP-L3 | 26 | (14,30) |
| Doylestown algorithm | 28 | (20,31) | hsa-miR-155-5p-fold changes | 30 | (27,31) |
| AFP and AFP-L3 | 30 | (19,31) | hsa-miR-21-5p-fold changes | 31 | (27,31) |

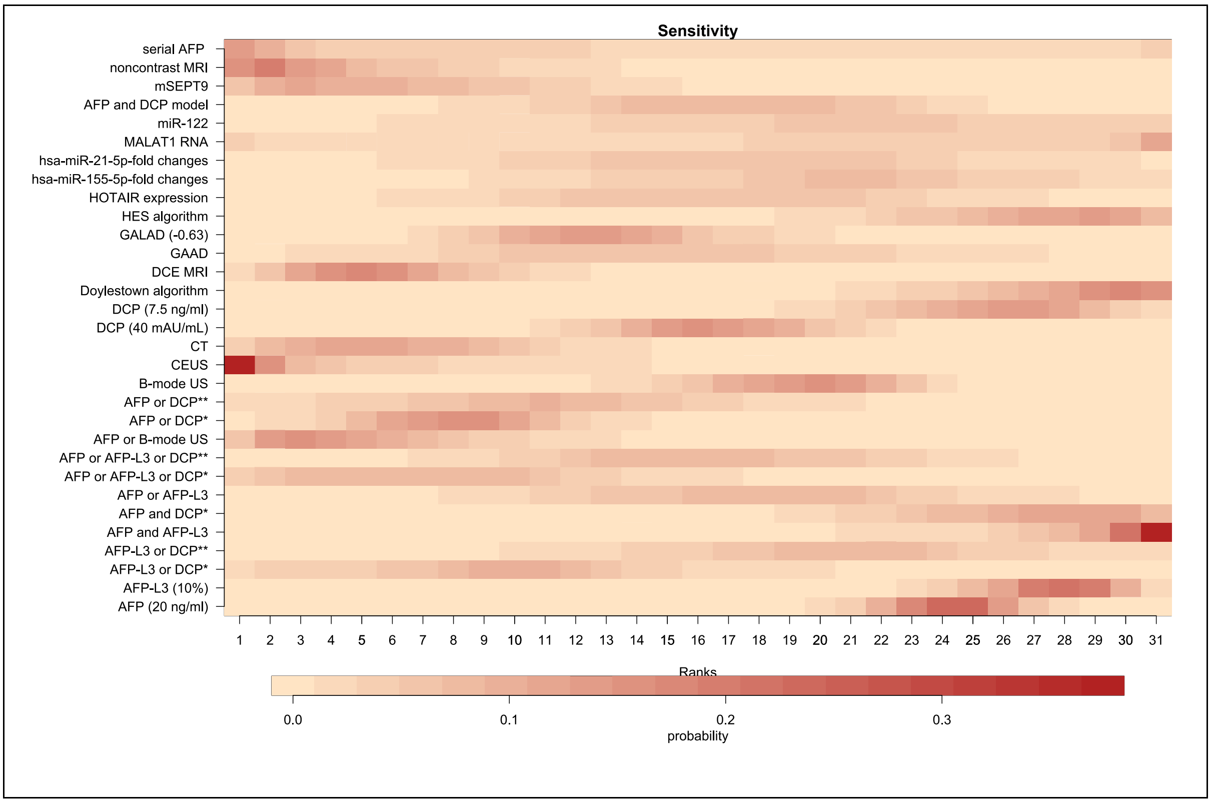

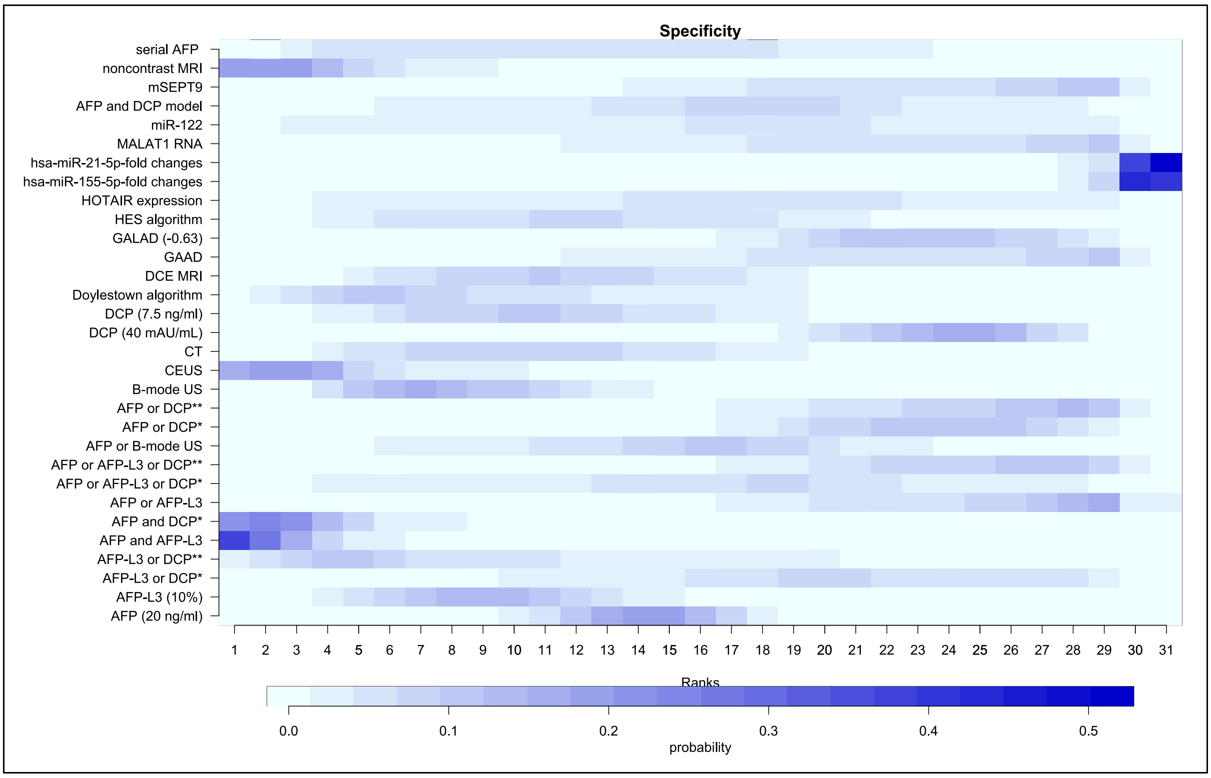


**Figure S13.1 Ranking plots**

## S13.2. ROC plots for imaging tests

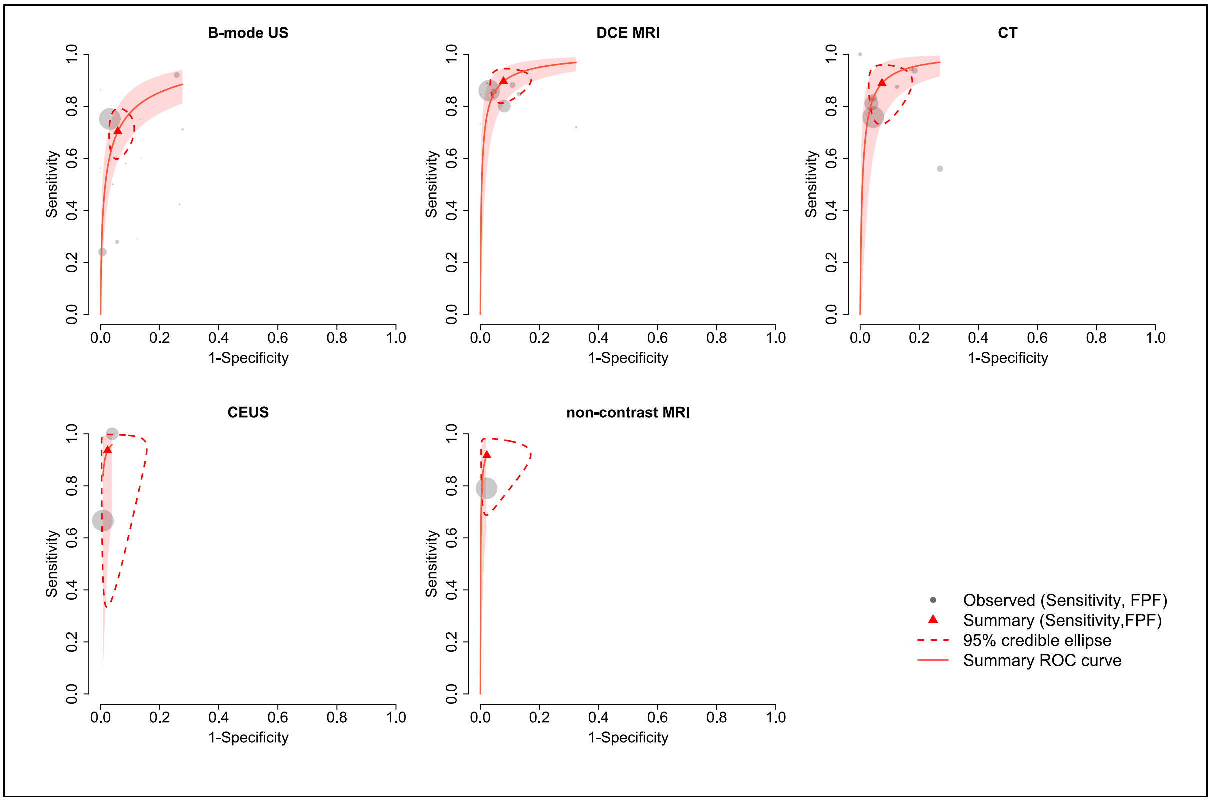


**Figure S13.2 Sensitivity and specificity of imaging tests for detecting HCC of any stage.**

## S13.3. Stage-specific, multiple-threshold model

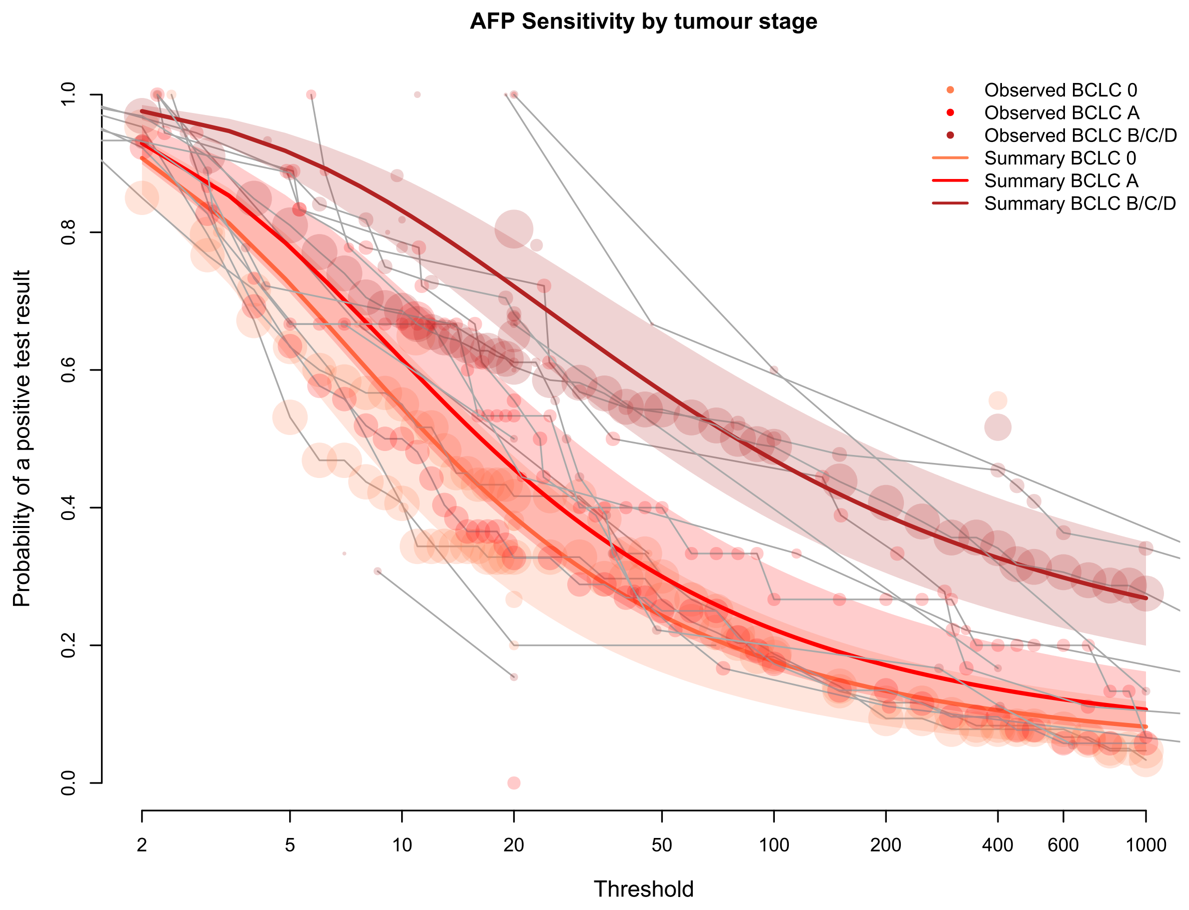


*Note that the observed datapoints plotted on this graph are only those from studies reporting subgroup-specific stratified results, whereas the synthesis model takes into account a wider range of evidence, including studies reporting sensitivity across 2 or all categories; see* Appendix S5.2.
*The results shown here are based on a model incorporating a constraint that sensitivity must remain either the same or increase as the disease stage progresses.*
*See Derezea et al.*[7] *for more details.*

**Figure S13.3 Sensitivity of AFP in stage-specific, multiple-threshold model**

# Appendix S14 Exploration of heterogeneity

## S14.1. Analyses restricted to higher-quality evidence

### S14.1.1. Network meta-analysis

When restricted to one-gate studies, the connected synthesis network comprises 39 tests evaluated across 54 studies (*Figure S14.1*).

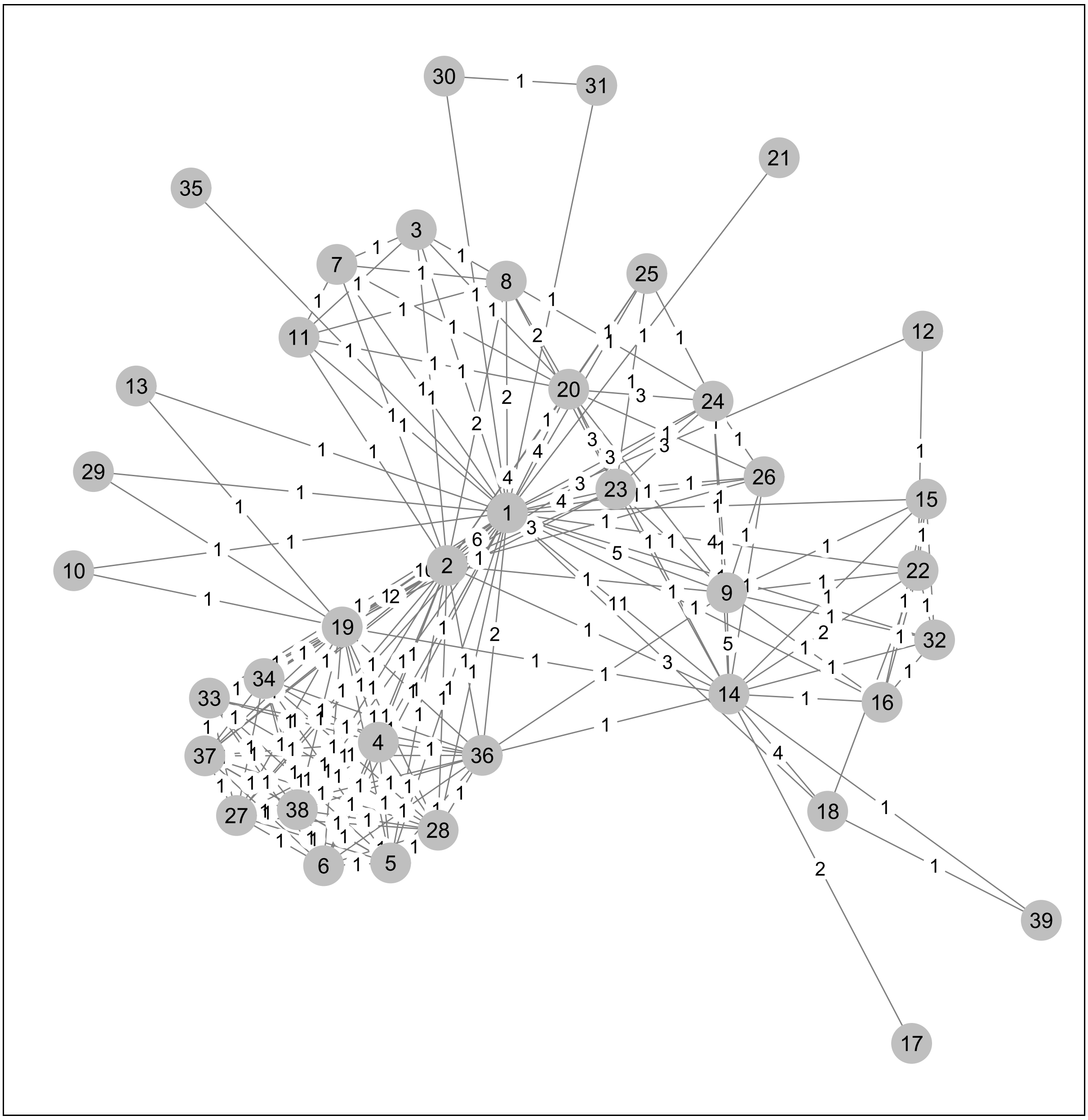

**Figure S14.1 Connected synthesis network restricted to one-gate studies. Grey circles represent individual tests (see key below for test names). Numbers on lines show the number of studies that evaluated the particular pair of tests.**

**Key for test names**: **(1)** AFP, **(2)** AFP-L3, **(3)** AFP-L3 or DCP (ng/ml), **(4)** AFP and AFP-L3, **(5)** AFP and DCP, **(6)** AFP and DCP and AFP-L3, **(7)** AFP or AFP-L3, **(8)** AFP or AFP-L3 or DCP (ng/ml), **(9)** AFP or B-mode US, **(10)** AFP or DCP, **(11)** AFP or DCP (ng/ml), **(12)** AFP or DCE MRI, **(13)** AFP x DCP, **(14)** B-mode US, **(15)** B-mode US or AFP or serial AFP, **(16)** B-mode US or Serial AFP, **(17)** CEUS, **(18)** CT, **(19)** DCP (mAU/ml), **(20)** DCP (ng/ml), **(21)** Doylestown algorithm, **(22)** DCE MRI, **(23)** GALAD, **(24)** HES algorithm, **(25)** HES algorithm 2, **(26)** Longitudinal GALAD, **(27)** mFB-I, **(29)** mFB-J, **(29)** Model based on AFP and DCP, **(30)** mRNA-199-a, **(31)** mRNA-21, **(32)** noncontrast MRI, **(33)** PEB algorithm (AFP), **(34)** PEB algorithm (DCP), **(35)** PEB algorithm or AFP, **(36)** serial AFP1, **(37)** uFB (AFP), **(38)** uFB (DCP), **(39)** US and CT.

**Table S14.1 Comparison of summary estimates of the sensitivity and specificity of each test, based on (1) all studies, (2) only one-gate studies. Both sets of results are based on the NMA-DTA model.**

| Test | (1) All studies | | | (2) Only one-gate studies | | |
|---|---|---|---|---|---|---|
| | No. of studies | Sensitivity (95%CrI) | Specificity (95%CrI) | No. of studies | Sensitivity (95%CrI) | Specificity (95%Cri) |
| AFP 20 ng/mL | 136 | 0.60 (0.57,0.64) | 0.90 (0.88,0.92) | 44 | 0.48 (0.41,0.55) | 0.93 (0.90,0.95) |
| AFP-L3 10% | 17 | 0.52 (0.42,0.62) | 0.93 (0.89,0.96) | 6 | 0.45 (0.24,0.68) | 0.94 (0.86,0.98) |
| AFP-L3 or DCP (ng/ml) | 2 | 0.68 (0.42,0.86) | 0.94 (0.78,0.99) | 1 | 0.52 (0.08,0.94) | 0.91 (0.48,0.99) |
| AFP and AFP-L3 | 2 | 0.43 (0.18,0.71) | 0.99 (0.94,1.00) | 1 | 0.24 (0.02,0.82) | 0.98 (0.83,1.00) |
| AFP and DCP | 3 | 0.54 (0.32,0.74) | 0.98 (0.92,1.00) | 1 | 0.29 (0.03,0.85) | 0.96 (0.69,1.00) |
| AFP and DCP and AFP-L3 | 1 | 0.31 (0.05,0.77) | 0.99 (0.96,1.00) | 1 | 0.19 (0.01,0.78) | 0.99 (0.93,1.00) |
| AFP or AFP-L3 | 3 | 0.73 (0.49,0.89) | 0.73 (0.41,0.91) | 1 | 0.60 (0.11,0.95) | 0.82 (0.29,0.98) |
| AFP or AFP-L3 or DCP (ng/ml) | 3 | 0.76 (0.53,0.89) | 0.75 (0.50,0.90) | 2 | 0.61 (0.24,0.87) | 0.82 (0.48,0.95) |
| AFP or B-mode US | 8 | 0.90 (0.79,0.96) | 0.89 (0.77,0.95) | 5 | 0.89 (0.71,0.97) | 0.92 (0.78,0.98) |
| AFP or DCP | 7 | 0.87 (0.80,0.92) | 0.77 (0.62,0.88) | 1 | 0.78 (0.20,0.98) | 0.75 (0.20,0.97) |
| AFP or DCP (ng/ml) | 3 | 0.83 (0.65,0.94) | 0.74 (0.48,0.90) | 1 | 0.61 (0.10,0.95) | 0.80 (0.26,0.98) |
| AFP or DCE MRI | 1 | 0.97 (0.76,1.00) | 0.86 (0.44,0.98) | 1 | 0.96 (0.56,1.00) | 0.87 (0.35,0.99) |
| AFP x DCP | 1 | 0.68 (0.22,0.95) | 0.64 (0.18,0.93) | 1 | 0.61 (0.10,0.97) | 0.64 (0.13,0.95) |
| B-mode US | 20 | 0.70 (0.62,0.78) | 0.94 (0.90,0.97) | 16 | 0.63 (0.52,0.74) | 0.96 (0.92,0.98) |
| B-mode US or AFP or Serial AFP | 1 | 0.87 (0.49,0.98) | 0.69 (0.25,0.94) | 1 | 0.84 (0.26,0.99) | 0.67 (0.17,0.96) |
| B-mode US or Serial AFP | 1 | 0.87 (0.48,0.98) | 0.72 (0.27,0.95) | 1 | 0.84 (0.27,0.99) | 0.69 (0.17,0.96) |
| CEUS | 2 | 0.94 (0.65,1.00) | 0.98 (0.89,1.00) | 2 | 0.93 (0.48,1.00) | 0.98 (0.89,1.00) |
| CT | 8 | 0.89 (0.79,0.96) | 0.93 (0.85,0.97) | 4 | 0.83 (0.66,0.93) | 0.95 (0.87,0.98) |
| DCP 40 mAU/ml | 29 | 0.75 (0.68,0.81) | 0.76 (0.67,0.84) | 10 | 0.53 (0.35,0.69) | 0.81 (0.63,0.92) |
| DCP 7.5 ng/ml | 11 | 0.57 (0.43,0.71) | 0.92 (0.85,0.96) | 5 | 0.32 (0.16,0.58) | 0.96 (0.89,0.98) |
| Doylestown algorithm | 3 | 0.50 (0.23,0.70) | 0.94 (0.84,0.98) | 1 | 0.00 (0.00,0.45) | 0.90 (0.40,0.99) |
| DCE MRI | 11 | 0.90 (0.83,0.94) | 0.92 (0.85,0.96) | 8 | 0.90 (0.83,0.95) | 0.92 (0.84,0.97) |
| GALAD | 7 | 0.80 (0.71,0.87) | 0.78 (0.63,0.88) | 4 | 0.75 (0.57,0.87) | 0.77 (0.53,0.91) |

| Test | (1) All studies | | | (2) Only one-gate studies | | |
|---|---|---|---|---|---|---|
| | No. of studies | Sensitivity (95%CrI) | Specificity (95%CrI) | No. of studies | Sensitivity (95%CrI) | Specificity (95%Cri) |
| HES algorithm | 3 | 0.53 (0.33,0.72) | 0.92 (0.79,0.97) | 3 | 0.44 (0.23,0.66) | 0.93 (0.80,0.98) |
| HES algorithm 2 | 1 | 0.78 (0.35,0.96) | 0.78 (0.33,0.96) | 1 | 0.70 (0.14,0.96) | 0.82 (0.30,0.98) |
| Longitudinal GALAD | 1 | 0.73 (0.28,0.94) | 0.91 (0.62,0.99) | 1 | 0.65 (0.12,0.96) | 0.92 (0.54,0.99) |
| mFB-I | 1 | 0.74 (0.31,0.95) | 0.93 (0.65,0.99) | 1 | 0.60 (0.10,0.95) | 0.93 (0.54,0.99) |
| mFB-J | 1 | 0.74 (0.30,0.95) | 0.93 (0.66,0.99) | 1 | 0.60 (0.10,0.95) | 0.93 (0.55,0.99) |
| Model based on AFP and DCP | 3 | 0.74 (0.56,0.87) | 0.87 (0.68,0.95) | 1 | 0.57 (0.10,0.94) | 0.91 (0.46,0.99) |
| mRNA-199-a | 1 | 0.34 (0.06,0.82) | 1.00 (0.93,1.00) | 1 | 0.35 (0.03,0.90) | 1.00 (0.93,1.00) |
| mRNA-21 | 1 | 1.00 (0.86,1.00) | 0.87 (0.35,0.99) | 1 | 1.00 (0.81,1.00) | 0.90 (0.33,0.99) |
| noncontrast MRI | 2 | 0.92 (0.75,0.97) | 0.98 (0.90,1.00) | 1 | 0.92 (0.43,0.99) | 0.95 (0.64,1.00) |
| PEB algorithm (AFP) | 1 | 0.81 (0.38,0.97) | 0.93 (0.65,0.99) | 1 | 0.69 (0.12,0.97) | 0.93 (0.53,0.99) |
| PEB algorithm (DCP) | 1 | 0.74 (0.29,0.95) | 0.93 (0.66,0.99) | 1 | 0.60 (0.09,0.95) | 0.93 (0.56,0.99) |
| PEB algorithm or AFP | 1 | 0.52 (0.15,0.87) | 0.96 (0.75,1.00) | 1 | 0.40 (0.05,0.90) | 0.98 (0.79,1.00) |
| serial AFP1 | 2 | 0.86 (0.21,0.99) | 0.92 (0.73,0.98) | 2 | 0.79 (0.07,1.00) | 0.93 (0.70,0.99) |
| uFB (AFP) | 1 | 0.89 (0.55,0.98) | 0.93 (0.65,0.99) | 1 | 0.81 (0.24,0.98) | 0.93 (0.54,0.99) |
| uFB (DCP) | 1 | 0.82 (0.40,0.97) | 0.93 (0.65,0.99) | 1 | 0.71 (0.15,0.97) | 0.93 (0.54,0.99) |
| US and CT | 1 | 0.88 (0.49,0.98) | 0.94 (0.66,0.99) | 1 | 0.86 (0.29,0.99) | 0.96 (0.64,1.00) |

**Table S14.2 Ranking estimates from one-gate studies**

| Sensitivity | | | Specificity | | |
|---|---|---|---|---|---|
| Test | Rank | 95%CrI | Test | Rank | 95%CrI |
| CEUS | 2 | (1,10) | CEUS | 1 | (1,10) |
| DCE MRI | 2 | (1,5) | B-mode US | 3 | (1,8) |
| AFP or B-mode US | 3 | (1,6) | CT | 4 | (1,10) |
| CT | 4 | (2,7) | DCP at 7.5 ng/mL | 4 | (1,10) |
| serial AFP 1 | 5 | (1,13) | AFP-L3 at 10% | 6 | (2,10) |
| GALAD at -0.63 | 6 | (3,8) | AFP at 20 ng/mL | 7 | (4,10) |
| B-mode US | 7 | (5,9) | HES algorithm | 7 | (2,12) |
| AFP or AFP-L3 or DCP (ng/ml) | 8 | (4,14) | serial AFP 1 | 7 | (1,13) |
| DCP at 40 mAU/mL | 9 | (6,12) | AFP or B-mode US | 8 | (1,12) |
| AFP at 20 ng/mL | 10 | (8,12) | DCE MRI | 8 | (2,11) |
| AFP-L3 at 10% | 11 | (7,13) | AFP or AFP-L3 or DCP (ng/ml) | 12 | (5,13) |
| HES algorithm | 11 | (7,13) | DCP at 40 mAU/mL | 12 | (8,13) |
| DCP at 7.5 ng/mL | 13 | (8,13) | GALAD at -0.63 | 12 | (9,13) |

### S14.1.2. Independent meta-analyses

*Table S14.3* shows results from single-test meta-analyses restricted to studies at low risk of bias in the reference standard domain of QUADAS-2 (n=17), for tests evaluated in ≥3 such studies.

**Table S14.3 Comparison of pooled estimates of the sensitivity and specificity of each test, with 95% credible intervals (CrIs). Based on (1) all studies, (2) only "one-gate" studies, (3) only studies assessed to be at low risk of bias in the reference standard domain of QUADAS-2. All results shown in this table are based on meta-analysis of each test separately.**

| Test | (1) All studies | | | (2) Only one-gate studies | | | (3) Only studies assessed to be at low risk of bias for reference standard | | |
|---|---|---|---|---|---|---|---|---|---|
| | No. of studies | Sensitivity (95%CrI) | Specificity (95%Cri) | No. of studies | Sensitivity (95%CrI) | Specificity (95%Cri) | No. of studies | Sensitivity (95%CrI) | Specificity (95%Cri) |
| **AFP** 20 ng/mL | 136 | 0.63 (0.60,0.67) | 0.89 (0.86,0.91) | 44 | 0.51 (0.44,0.58) | 0.93 (0.89,0.95) | 31 | 0.60 (0.53,0.65) | 0.88 (0.82,0.93) |
| **AFP-L3** at 10% | 17 | 0.45 (0.30,0.56) | 0.97 (0.88,1.00) | 6 | 0.43 (0.10,0.60) | 0.95 (0.81,0.99) | 5 | 0.46 (0.30,0.63) | 0.98 (0.76,1.00) |
| **B-mode US** | 20 | 0.62 (0.50,0.72) | 0.96 (0.92,0.99) | 16 | 0.61 (0.45,0.74) | 0.97 (0.92,0.99) | 5 | 0.56 (0.22,0.86) | 0.98 (0.90,1.00) |
| **CT** | 8 | 0.88 (0.67,0.98) | 0.92 (0.80,0.98) | 4 | 0.80 (0.64,0.93) | 0.95 (0.82,0.99) | 3 | 0.72 (0.25,0.95) | 0.92 (0.35,1.00) |
| **DCE MRI** | 11 | 0.84 (0.80,0.89) | 0.93 (0.86,0.97) | 8 | 0.87 (0.82,0.91) | 0.94 (0.86,0.98) | 6 | 0.84 (0.71,0.94) | 0.91 (0.67,0.99) |
| **DCP** at 40 mAU/mL | 29 | 0.74 (0.64,0.83) | 0.79 (0.64,0.90) | 10 | 0.57 (0.25,0.85) | 0.80 (0.37,0.98) | 11 | 0.66 (0.33,0.91) | 0.88 (0.55,0.99) |

## S14.2. Predominant aetiology of cirrhosis

### S14.2.1. Threshold-dependent sensitivity and specificity of alpha-fetoprotein in studies with different predominant aetiologies of cirrhosis

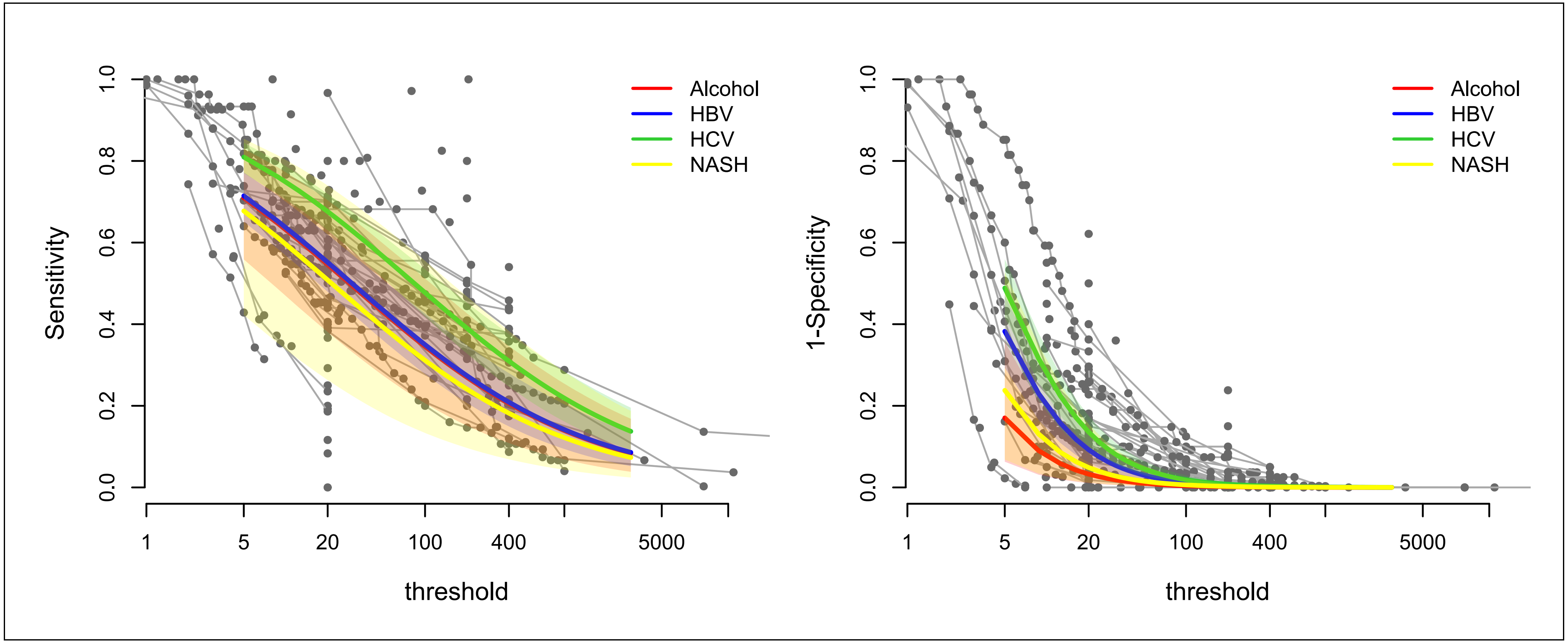


**Figure S14.2 Probabilities of a positive test result for AFP for different predominant aetiology categories**

## S14.2.2. Meta-regression exploring predominant aetiology of cirrhosis

**Table S14.4 Estimated sensitivity, with 95%CrI, of tests by predominant aetiology of cirrhosis. Results are based on meta-regression for each test separately.**

| Test | Alcohol | | HBV | | HCV | | NAFLD | |
|---|---|---|---|---|---|---|---|---|
| | No. studies | Sensitivity | No. studies | Sensitivity | No. studies | Sensitivity | No. studies | Sensitivity |
| AFP 20ng/mL | 6 | 0.55 (0.38,0.70) | 34 | 0.55 (0.48,0.62) | 71 | 0.68 (0.63,0.72) | 3 | 0.51 (0.26,0.74) |
| AFP-L3 10% | 0 | - | 2 | 0.52 (0.37,0.74) | 12 | 0.45 (0.32,0.52) | 2 | 0.15 (0.00,0.45) |
| AFP or B-mode US | 0 | - | 2 | 0.74 (0.07,0.99) | 4 | 0.93 (0.50,0.99) | 0 | - |
| B-mode US | 1 | 0.80 (0.36,0.97) | 6 | 0.39 (0.22,0.60) | 10 | 0.69 (0.54,0.81) | 0 | - |
| Contrast MRI | 0 | - | 5 | 0.82 (0.74,0.89) | 5 | 0.87 (0.81,0.92) | 0 | - |
| Multiphase CT | 1 | 0.79 (0.01,1.00) | 2 | 0.71 (0.04,0.99) | 3 | 0.94 (0.39,1.00) | 0 | - |
| DCP 40 mAU/mL | 0 | - | 12 | 0.73 (0.58,0.86) | 13 | 0.73 (0.58,0.86) | 2 | 0.91 (0.55,1.00) |
| DCP 7.5 ng/ml | 2 | 0.83 (0.38,0.99) | 0 | - | 6 | 0.48 (0.21,0.75) | 1 | 0.21 (0.00,0.78) |
| GALAD at -0.63 | 2 | 0.81 (0.51,1.00) | 0 | - | 4 | 0.58 (0.41,0.80) | 1 | 0.74 (0.39,1.00) |

**Table S14.5 Differences in sensitivity between predominant aetiology categories**

| Test | HBV−Alcohol | HCV−Alcohol | NAFLD−Alcohol | HCV−HBV | NAFLD−HBV | HCV−NAFLD |
|---|---|---|---|---|---|---|
| AFP 20 ng/mL | 0.01 (-0.17,0.18) | 0.13 (-0.03,0.30) | -0.04 (-0.33,0.25) | **0.12 (0.21,0.04)** | -0.04 (-0.30,0.20) | 0.17 (-0.07,0.42) |
| AFP-L3 10% | - | - | - | -0.07 (-0.34,0.08) | **-0.37 (-0.66,-0.05)** | 0.28 (-0.01, 0.47) |
| AFP or B-mode US | - | - | - | 0.17 (-0.30,0.85) | - | - |
| B-mode US | -0.40 (-0.67,0.07) | -0.11 (-0.34,0.35) | - | **0.30 (0.04,0.52)** | - | - |
| Contrast MRI | - | - | - | 0.05 (-0.05,0.15) | - | - |
| Multiphase CT | -0.04 (-0.85,0.86) | 0.11 (-0.39,0.94) | - | 0.19 (-0.37,0.91) | - | - |
| DCP 40 mAU/mL | - | - | - | 0.01 (-0.19,0.20) | 0.17 (-0.20,0.36) | -0.16 (-0.35,0.20) |
| DCP 7.5 ng/mL | - | -0.32 (-0.69,0.16) | -0.57 (-0.95,0.08) | - | - | 0.25 (-0.35,0.64) |
| GALAD at -0.63 | - | -0.21 (-0.50,0.12) | -0.04 (-0.46,0.34) | - | - | -0.15 (-0.49,0.25) |

**Table S14.6 Estimated specificity, with 95%CrI, of tests by predominant aetiology of cirrhosis. Results are based on meta-regression for each test separately.**

| Test | Alcohol | | HBV | | HCV | | NAFLD | |
|---|---|---|---|---|---|---|---|---|
| | No. studies | Specificity | No. studies | Specificity | No. studies | Specificity | No. studies | Specificity |
| AFP 20ng/mL | 6 | 0.97 (0.92,0.99) | 35 | 0.91 (0.87,0.94) | 69 | 0.86 (0.82,0.90) | 4 | 0.95 (0.84,0.99) |
| AFP-L3 10% | 0 | - | 2 | 0.88 (0.15,1.00) | 12 | 0.98 (0.86,1.00) | 2 | 0.97 (0.28,1.00) |
| AFP or B-mode US | 0 | - | 2 | 0.95 (0.17,1.00) | 4 | 0.92 (0.35,1.00) | 0 | - |
| B-mode US | 1 | 0.96 (0.22,1.00) | 6 | 0.99 (0.93,1.00) | 10 | 0.96 (0.86,0.99) | 0 | - |
| Contrast MRI | 1 | 0.88 (0.15,1.00) | 5 | 0.93 (0.70,0.99) | 4 | 0.96 (0.76,1.00) | 0 | - |
| Multiphase CT | 1 | 0.96 (0.08,1.00) | 2 | 0.88 (0.12,1.00) | 3 | 0.97 (0.62,1.00) | 0 | - |
| DCP 40 mAU/mL | 0 | - | 13 | 0.80 (0.56, 0.94) | 12 | 0.77 (0.50,0.92) | 2 | 0.73 (0.13,0.98) |
| DCP 7.5 ng/ml | 2 | 0.95 (0.33,1.00) | 0 | - | 6 | 0.98 (0.82,1.00) | 1 | 0.94 (0.14,1.00) |
| GALAD -0.63 | 2 | 0.94 (0.79,0.99) | 0 | - | 4 | 0.85 (0.62,0.94) | 1 | 0.75 (0.35,0.99) |

**Table S14.7 Differences in specificity between predominant aetiology categories**

| Test | HBV−Alcohol | HCV−Alcohol | NAFLD−Alcohol | HCV−HBV | NAFLD−HBV | HCV−NAFLD |
|---|---|---|---|---|---|---|
| AFP 20 ng/mL | -0.06 (-0.10,0.00) | **-0.10 (-0.15, -0.05)** | -0.02 (-0.12,0.05) | -0.04 (-0.09,0.00) | 0.04 (-0.06,0.10) | -0.09 (-0.14,0.02) |
| AFP-L3 10% | - | - | - | 0.08 (-0.06,0.82) | 0.05 (-0.57,0.79) | 0.00 (-0.10,0.69) |
| AFP or B-mode US | - | - | - | -0.02 (-0.54,0.71) | - | - |
| B-mode US | 0.02 (-0.04,0.76) | 0.00 (-0.11,0.73) | - | -0.02 (-0.13,0.04) | - | - |
| Contrast MRI | 0.04 (-0.20,0.76) | 0.06 (-0.13,0.78) | - | 0.02 (-0.17,0.25) | - | - |
| Multiphase CT | -0.05 (-0.78,0.76) | 0.01 (-0.29,0.87) | - | 0.07 (-0.24,0.82) | - | - |
| DCP 40 mAU/mL | - | - | - | -0.03 (-0.33,0.25) | -0.07 (-0.69,0.30) | 0.04 (-0.35,0.66) |
| DCP 7.5 ng/mL | - | 0.02 (-0.13,0.64) | 0.00 (-0.75,0.54) | - | - | 0.03 (-0.13,0.83) |
| GALAD -0.63 | - | -0.08 (-0.30,0.06) | -0.18 (-0.57,0.08) | - | - | 0.10 (-0.27,0.50) |

## S14.3. Meta-regressions exploring sex, age, and cirrhosis severity

**Table S14.8** Heterogeneity exploration: Results from meta-regressions with study-level covariates. For the imaging tests, producing binary results, coefficient 'b' represents the estimated association of the covariate with logit(sensitivity) or logit(specificity). For continuous biomarkers, coefficient 'b' represents the estimated association of the covariate with the location parameter of the Jones et al (2019) model. Equivalently, 'b' represents a difference in mean continuous test result on the log scale.

| Test | Sex: Proportion of male participants | | | Age: Mean participant age | | | Cirrhosis severity: Proportion of study participants with Child Pugh score A | | |
|---|---|---|---|---|---|---|---|---|---|
| | No. of studies | b (95%CrI) | | No. of studies | b (95%CrI) | | No. of studies | b (95%CrI) | |
| | | Sensitivity | Specificity | | Sensitivity | Specificity | | Sensitivity | Specificity |
| **Continuous biomarkers** | | | | | | | | | |
| AFP | 118 | 0.57 (-1.87,2.98) | -0.15 (-1.27,0.94) | 118 | -0.04 (-0.10,0.02) | **-0.04 (-0.08,-0.01)** | 79 | **-1.56 (-3.07,-0.09)** | -0.67 (-1.54,0.24) |
| AFP-L3 | 15 | 0.96 (-3.80,7.90) | -2.48 (-13.03,7.43) | 15 | 0.01 (-0.08, 0.15) | -0.01 (-0.24,0.19) | 9 | 0.55 (-3.95,7.43) | 2.33 (-7.06,12.54) |
| DCP (mAU/mL) | 26 | 4.52 (-1.24,10.75) | 3.63 (-0.37,7.99) | 25 | -0.03 (-0.15,0.09) | -0.04 (-0.16,0.08) | 17 | -0.09 (-5.35,4.94) | -0.25 (-3.83,4.66) |
| DCP (ng/ml) | 10 | 1.73 (-11.07,14.93) | -5.97 (-17.38,4.05) | 9 | 0.10 (-0.16,0.37) | -0.09 (-0.30,0.13) | - | - | - |
| GALAD | 7 | -1.24 (-10.07,8.84) | 2.07 (-2.37,5.33) | 7 | -0.05 (-0.23,0.18) | -0.01 (-0.19,0.14) | - | - | - |
| **Imaging** | | | | | | | | | |
| B-mode US | 18 | 2.46 (-1.77,6.82) | 0.13 (-7.03,7.34) | 19 | 0.03 (-0.09,0.15) | -0.10 (-0.26,0.07) | 13 | 0.33 (-2.47,3.13) | 1.39 (-3.09,6.22) |
| Contrast MRI | 11 | 0.62 (-2.48,3.53) | 2.64 (-5.37,10.77) | 11 | 0.01 (-0.05,0.08) | 0.09 (-0.10,0.26) | 5 | 0.30 (-3.31,3.09) | 1.80 (-0.55,4.15) |
| Multiphase CT | 8 | 3.83 (-5.35,12.69) | 2.69 (-4.70,10.43) | 8 | **0.19 (0.05,0.31)** | -0.06 (-0.27,0.18) | - | - | - |

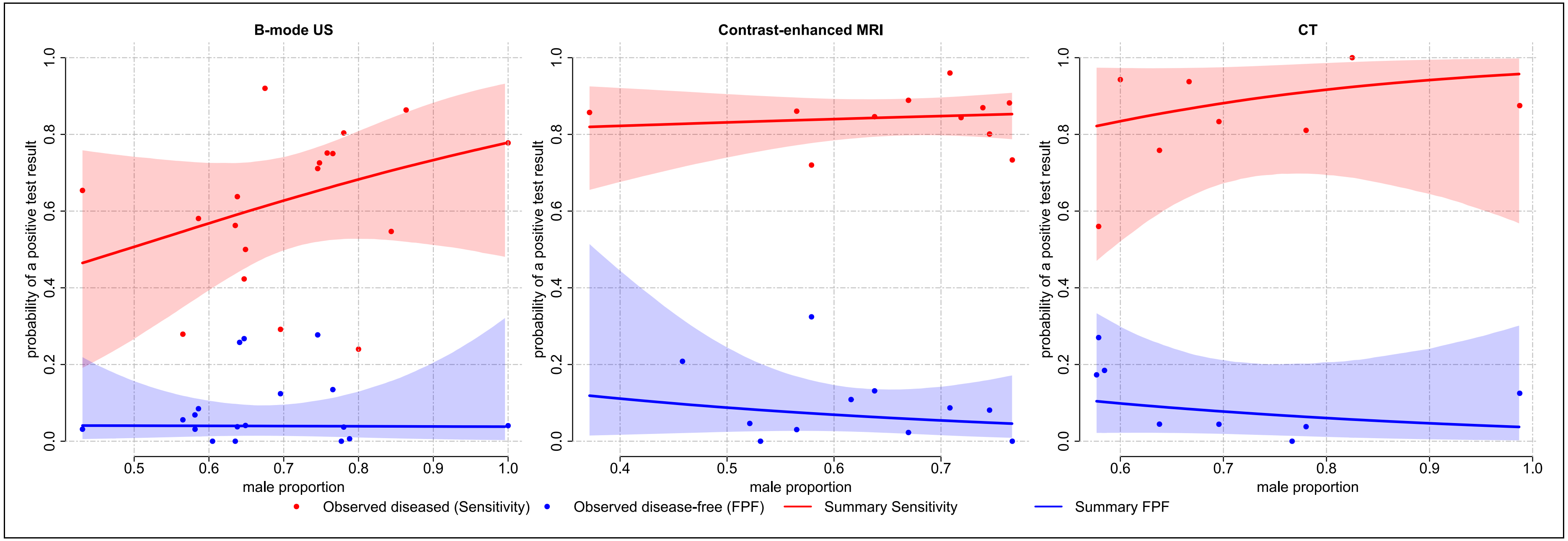


**Figure S14.3 Heterogeneity exploration: sex. Results from meta-regressions with the proportion of male participants as a study-level covariate. Summary estimates of sensitivity (red) and FPF (blue), with 95% CrIs for the imaging tests. Study level estimates are also shown.**

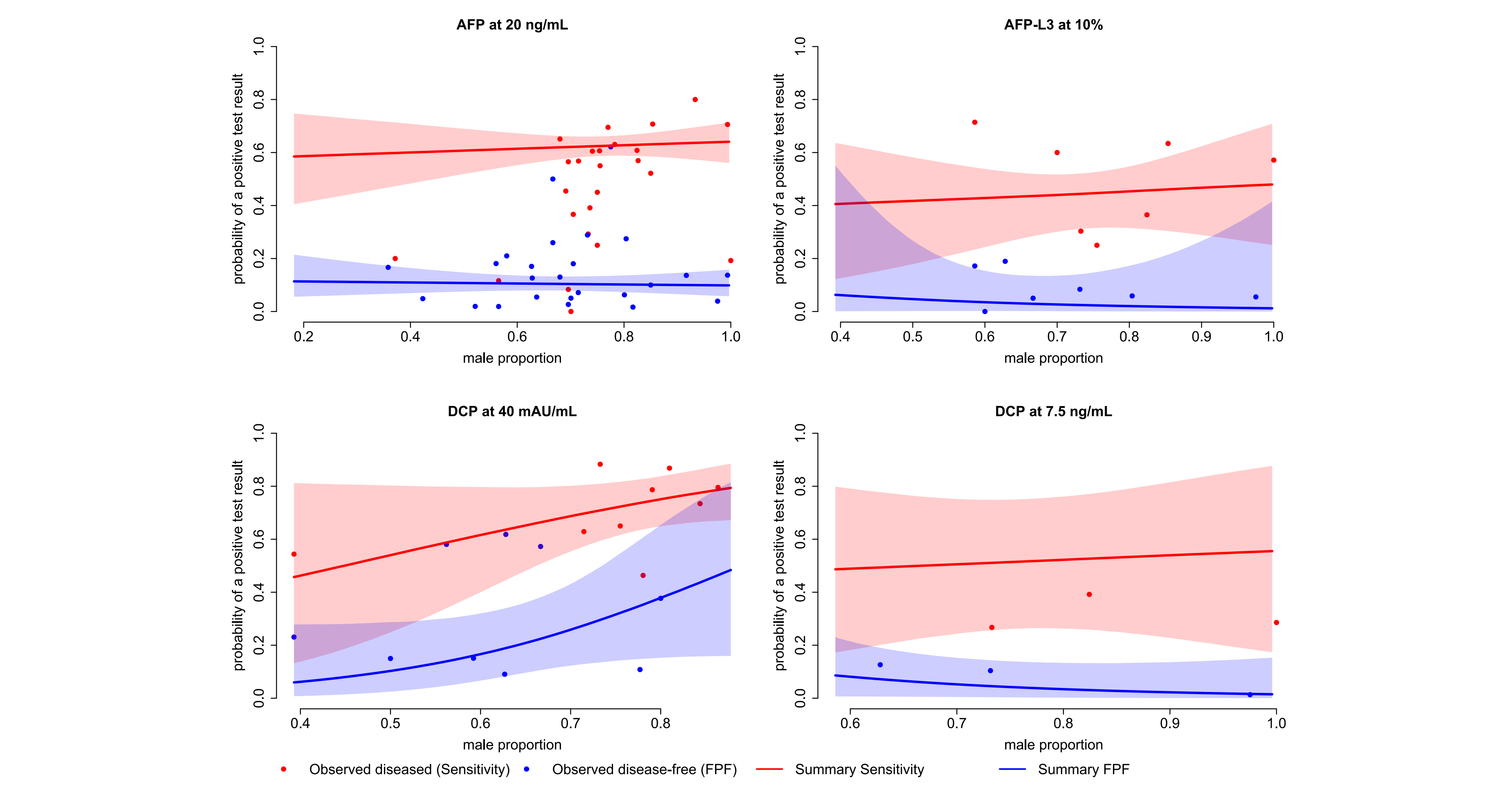


**Figure S14We are unable to share .4 Heterogeneity exploration: sex. Results from meta-regressions with the proportion of male participants as a study-level covariate. Summary estimates of sensitivity (red) and FPF (blue), with 95% CrIs for continuous biomarkers at the most commonly reported threshold. Study level estimates are also shown.**

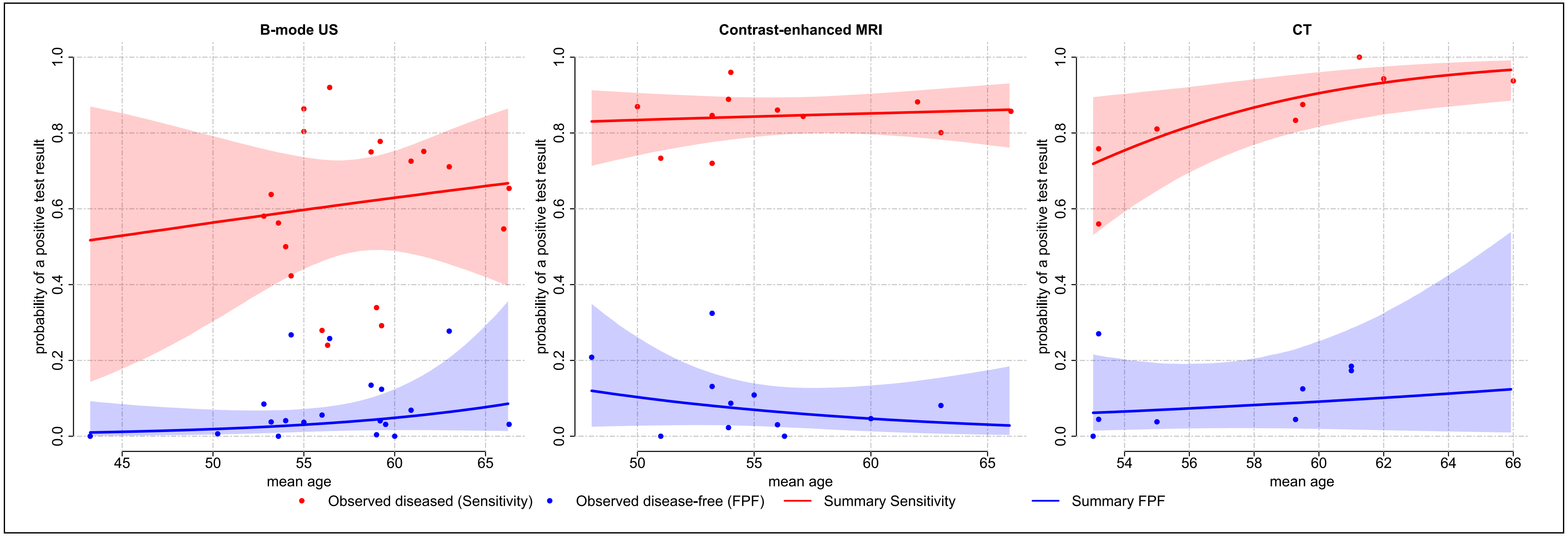


**Figure S14.5** **Heterogeneity exploration: age. Results from meta-regressions with the mean participant age as a study-level covariate. Summary estimates of sensitivity (red) and FPF (blue), with 95% CrIs for the imaging tests. Study level estimates are also shown.**

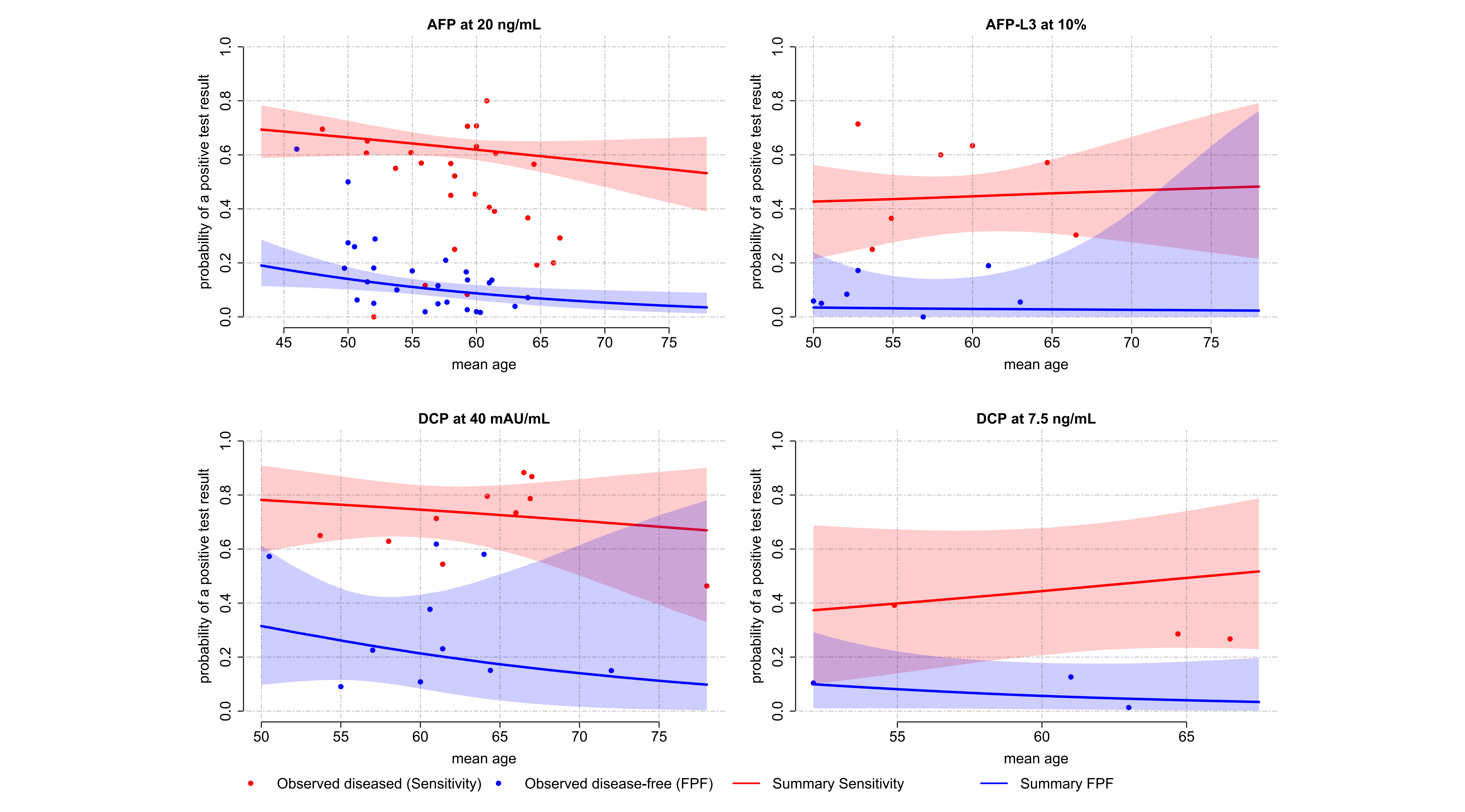


**Figure S14.6 Heterogeneity exploration: age. Results from meta-regressions with the mean participant age as a study-level covariate. Summary estimates of sensitivity (red) and FPF (blue), with 95% CrIs for continuous biomarkers at the most commonly reported threshold. Study level estimates are also shown.**

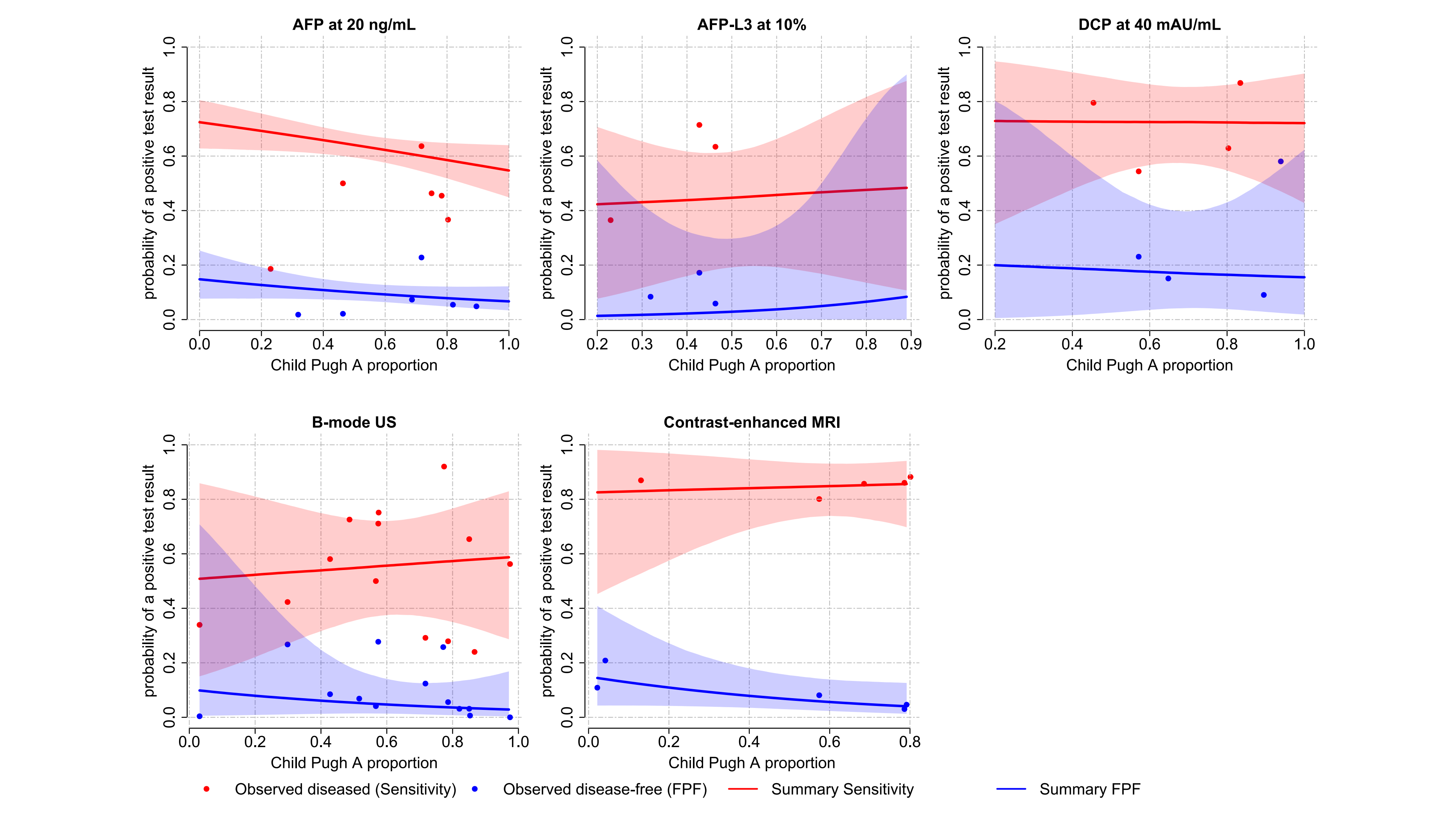


**Figure S14.7** **Heterogeneity exploration: severity of cirrhosis. Results from meta-regressions with the proportion of study participants with Child Pugh score A as a study-level covariate. Summary estimates of sensitivity (red) and FPF (blue), with 95% CrIs. Study level estimates are also shown. For continuous biomarkers, estimates shown correspond to the most commonly reported threshold.**

# Appendix S15 Forest plots of tests reported in 2 or more studies

## S15.1. AFP at threshold 20 ng/mL

| Study | TP | FP | FN | TN | Sens (95% CI) | Spec (95% CI) |
|---|---|---|---|---|---|---|
| Weis et al. (2019) | 5 | 2 | 15 | 18 | 0.25 (0.11-0.47) | 0.90 (0.70-0.97) |
| Hallager et al. (2018) | 56 | 254 | 30 | 1701 | 0.65 (0.54-0.74) | 0.87 (0.85-0.88) |
| Sultanik et al. (2017) | 29 | 21 | 17 | 95 | 0.63 (0.49-0.75) | 0.82 (0.74-0.88) |
| Lin et al. (2016) | 74 | 11 | 48 | 50 | 0.61 (0.52-0.69) | 0.82 (0.71-0.90) |
| Lim et al. (2016) | 205 | 47 | 156 | 229 | 0.57 (0.52-0.62) | 0.83 (0.78-0.87) |
| Sterling et al. (2012) | 11 | 93 | 9 | 265 | 0.55 (0.34-0.74) | 0.74 (0.69-0.78) |
| Sterling et al. (2009) | 45 | 86 | 29 | 212 | 0.61 (0.50-0.71) | 0.71 (0.66-0.76) |
| Zinkin et al. (2008) | 29 | 14 | 12 | 37 | 0.71 (0.56-0.83) | 0.73 (0.60-0.83) |
| Tayob et al. (2022) | 5 | 20 | 21 | 493 | 0.19 (0.08-0.38) | 0.96 (0.94-0.97) |
| Guan et al. (2022) | 26 | 1 | 21 | 56 | 0.55 (0.41-0.68) | 0.98 (0.90-1.00) |
| Dabbish et al. (2022) | 27 | 11 | 42 | 55 | 0.39 (0.28-0.51) | 0.83 (0.72-0.90) |
| Yoon et al. (2020) | 2 | 3 | 22 | 110 | 0.08 (0.02-0.25) | 0.97 (0.92-0.99) |
| Qian et al. (2022) | 0 | 26 | 5 | 490 | 0.00 (0.00-0.43) | 0.95 (0.93-0.97) |
| Malov et al. (2021) | 25 | 3 | 30 | 52 | 0.45 (0.33-0.58) | 0.95 (0.86-0.98) |
| Lewin et al. (2021) | 22 | 5 | 38 | 98 | 0.37 (0.26-0.50) | 0.95 (0.89-0.98) |
| Lu et al. (2020) | 51 | 18 | 42 | 52 | 0.55 (0.45-0.65) | 0.74 (0.63-0.83) |
| Demirtas et al. (2020) | 7 | 5 | 28 | 254 | 0.20 (0.10-0.36) | 0.98 (0.95-0.99) |
| Kim et al. (2017) | 5 | 20 | 38 | 1037 | 0.12 (0.05-0.25) | 0.98 (0.97-0.99) |
| Zhang et al. (2019) | 164 | 18 | 124 | 268 | 0.57 (0.51-0.63) | 0.94 (0.91-0.96) |
| Ma et al. (2017) | 130 | 105 | 57 | 64 | 0.70 (0.63-0.76) | 0.38 (0.31-0.46) |
| Hussein et al. (2012) | 29 | 6 | 1 | 9 | 0.97 (0.84-1.00) | 0.60 (0.36-0.80) |
| Gui et al. (2011) | 24 | 3 | 22 | 19 | 0.52 (0.38-0.66) | 0.86 (0.66-0.95) |
| Pocha et al. (2013) | 12 | 20 | 5 | 126 | 0.71 (0.47-0.87) | 0.86 (0.79-0.91) |
| Abdel Rehem & El-Shikh (2011) | 9 | 30 | 11 | 30 | 0.45 (0.26-0.66) | 0.50 (0.38-0.62) |
| Beudeker et al. (2023) | 81 | 36 | 196 | 249 | 0.29 (0.24-0.35) | 0.87 (0.83-0.90) |
| Chen et al. (2023) | 58 | 20 | 85 | 153 | 0.41 (0.33-0.49) | 0.88 (0.82-0.92) |
| D'Abundo et al. (2024) | 13 | 1 | 10 | 13 | 0.57 (0.37-0.75) | 0.93 (0.69-0.99) |
| Hai et al. (2025) | 78 | 26 | 33 | 30 | 0.70 (0.61-0.78) | 0.54 (0.41-0.66) |
| Hawash et al. (2024) | 49 | 17 | 32 | 64 | 0.60 (0.49-0.70) | 0.79 (0.69-0.86) |
| Khalil et al. (2024) | 48 | 1 | 12 | 59 | 0.80 (0.68-0.88) | 0.98 (0.91-1.00) |
| Ricco et al. (2018) | 99 | 13 | 159 | 117 | 0.38 (0.32-0.44) | 0.90 (0.84-0.94) |
| Chang et al. (2015) | 192 | 83 | 171 | 1151 | 0.53 (0.48-0.58) | 0.93 (0.91-0.94) |
| Gopal et al. (2014) | 317 | 69 | 135 | 607 | 0.70 (0.66-0.74) | 0.90 (0.88-0.92) |
| Ozkan et al. (2011) | 45 | 1 | 30 | 54 | 0.60 (0.49-0.70) | 0.98 (0.90-1.00) |
| Yu et al. (2011) | 78 | 38 | 117 | 285 | 0.40 (0.33-0.47) | 0.88 (0.84-0.91) |
| Marrero et al. (2009) | 247 | 42 | 172 | 375 | 0.59 (0.54-0.64) | 0.90 (0.87-0.93) |
| Liu et al. (2007) | 162 | 29 | 65 | 51 | 0.71 (0.65-0.77) | 0.64 (0.53-0.74) |
| Guo et al. (2022) | 10 | 3 | 8 | 17 | 0.56 (0.34-0.76) | 0.85 (0.64-0.95) |
| Zhu et al. (2020) | 26 | 0 | 11 | 33 | 0.70 (0.54-0.82) | 1.00 (0.90-1.00) |
| Yang et al. (2021) | 56 | 17 | 65 | 128 | 0.46 (0.37-0.55) | 0.88 (0.82-0.92) |
| Brigida et al. (2020) | 21 | 10 | 9 | 20 | 0.70 (0.52-0.83) | 0.67 (0.49-0.81) |
| El-Serag et al. (2025) | 8 | 40 | 35 | 2166 | 0.19 (0.10-0.33) | 0.98 (0.97-0.99) |
| Kim et al. (2019) | 32 | 7 | 32 | 321 | 0.50 (0.38-0.62) | 0.98 (0.96-0.99) |
| Sanai et al. (2010) | 118 | 34 | 88 | 165 | 0.57 (0.50-0.64) | 0.83 (0.77-0.88) |
| Jeng et al. (2014) | 59 | 15 | 31 | 75 | 0.66 (0.56-0.75) | 0.83 (0.74-0.89) |
| Marsh et al. (2025) | 8 | 34 | 26 | 1089 | 0.24 (0.13-0.41) | 0.97 (0.96-0.98) |
| Tsai et al. (2005) | 19 | 3 | 22 | 38 | 0.46 (0.32-0.61) | 0.93 (0.81-0.98) |
| Zhu et al. (2025) | 42 | 18 | 24 | 61 | 0.64 (0.52-0.75) | 0.77 (0.67-0.85) |
| Xing et al. (2023) | 11 | 13 | 25 | 204 | 0.31 (0.18-0.47) | 0.94 (0.90-0.96) |
| Chen et al. (2024) | 194 | 213 | 181 | 1782 | 0.52 (0.47-0.57) | 0.89 (0.88-0.90) |
| Chen et al. (2024) | 33 | 203 | 42 | 2003 | 0.44 (0.33-0.55) | 0.91 (0.90-0.92) |
| Sun et al. (2012) | 17 | 8 | 10 | 19 | 0.63 (0.44-0.78) | 0.70 (0.51-0.84) |
| Summary estimates (NMA-DTA) | | | | | 0.60 (0.57-0.64) | 0.90 (0.88-0.92) |

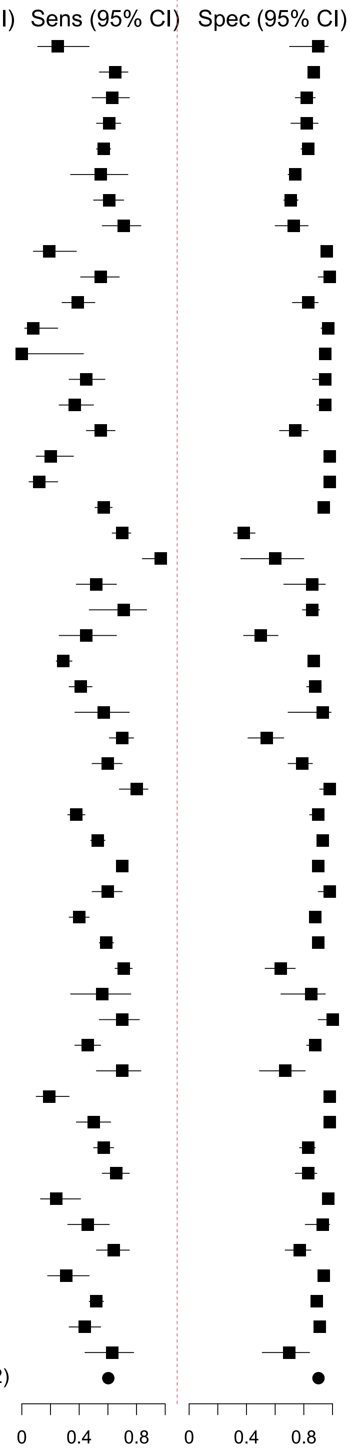


**Figure S15.1 Forest plot: AFP at threshold 20 ng/mL**

## S15.2. AFP-L3 at threshold 10%

| Study | TP | FP | FN | TN | Sens (95% CI) | Spec (95% CI) |
|---|---|---|---|---|---|---|
| Singal et al. (2012) | 30 | 61 | 12 | 294 | 0.71 (0.56-0.82) | 0.83 (0.79-0.87) |
| Sterling et al. (2012) | 5 | 18 | 15 | 340 | 0.25 (0.11-0.47) | 0.95 (0.92-0.97) |
| Sterling et al. (2009) | 27 | 25 | 47 | 273 | 0.36 (0.26-0.47) | 0.92 (0.88-0.95) |
| Zinkin et al. (2008) | 26 | 3 | 15 | 48 | 0.63 (0.48-0.76) | 0.94 (0.84-0.98) |
| Tayob et al. (2022) | 4 | 29 | 3 | 497 | 0.57 (0.25-0.84) | 0.94 (0.92-0.96) |
| Guan et al. (2022) | 11 | 3 | 36 | 54 | 0.23 (0.13-0.37) | 0.95 (0.86-0.98) |
| Brigida et al. (2020) | 18 | 0 | 12 | 30 | 0.60 (0.42-0.75) | 1.00 (0.89-1.00) |
| Beudeker et al. (2023) | 84 | 54 | 193 | 231 | 0.30 (0.25-0.36) | 0.81 (0.76-0.85) |
| Lim et al. (2016) | 170 | 20 | 191 | 256 | 0.47 (0.42-0.52) | 0.93 (0.89-0.95) |
| Marrero et al. (2009) | 176 | 13 | 243 | 404 | 0.42 (0.37-0.47) | 0.97 (0.95-0.98) |
| El-Serag et al. (2025) | 13 | 86 | 30 | 2120 | 0.30 (0.18-0.45) | 0.96 (0.95-0.97) |
| Summary estimates (NMA-DTA) | | | | | 0.52 (0.42-0.62) | 0.94 (0.90-0.96) |

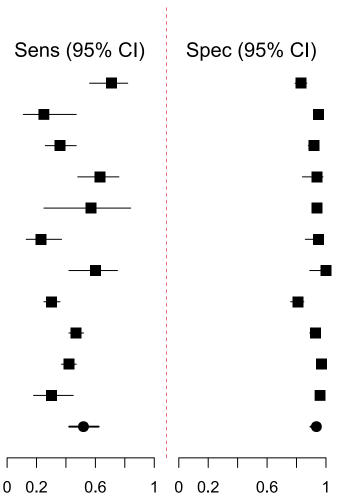


**Figure S15.2 Forest plot: AFP-L3 at threshold 10%**

## S15.3. DCP at threshold 40 mAU/ml

| Study | TP | FP | FN | TN | Sens (95% CI) | Spec (95% CI) |
|---|---|---|---|---|---|---|
| Takaya et al. (2019) | 19 | 3 | 22 | 17 | 0.46 (0.32-0.61) | 0.85 (0.64-0.95) |
| Ricco et al. (2018) | 203 | 49 | 55 | 81 | 0.79 (0.74-0.84) | 0.62 (0.53-0.70) |
| Lim et al. (2016) | 227 | 25 | 134 | 251 | 0.63 (0.58-0.68) | 0.91 (0.87-0.94) |
| Mukozu et al. (2013) | 31 | 6 | 26 | 20 | 0.54 (0.41-0.66) | 0.77 (0.58-0.89) |
| Sterling et al. (2012) | 13 | 205 | 7 | 153 | 0.65 (0.43-0.82) | 0.43 (0.38-0.48) |
| Guan et al. (2022) | 40 | 5 | 7 | 52 | 0.85 (0.72-0.93) | 0.91 (0.81-0.96) |
| Loglio et al. (2020) | 47 | 16 | 17 | 132 | 0.73 (0.61-0.82) | 0.89 (0.83-0.93) |
| Beudeker et al. (2023) | 657 | 361 | 87 | 223 | 0.88 (0.85-0.90) | 0.38 (0.34-0.42) |
| Chen et al. (2023) | 102 | 39 | 41 | 134 | 0.71 (0.63-0.78) | 0.77 (0.70-0.83) |
| Caviglia et al. (2023) | 178 | 570 | 27 | 412 | 0.87 (0.82-0.91) | 0.42 (0.39-0.45) |
| Woo et al. (2025) | 35 | 27 | 9 | 152 | 0.80 (0.66-0.89) | 0.85 (0.79-0.89) |
| Summary estimates (NMA-DTA) | | | | | 0.75 (0.68-0.81) | 0.76 (0.67-0.84) |

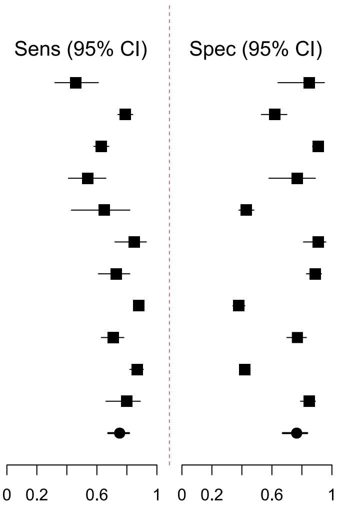


**Figure S15.3 Forest plot: DCP at threshold 40 mAU/ml**

## S15.4. DCP at threshold 7.5 ng/ml

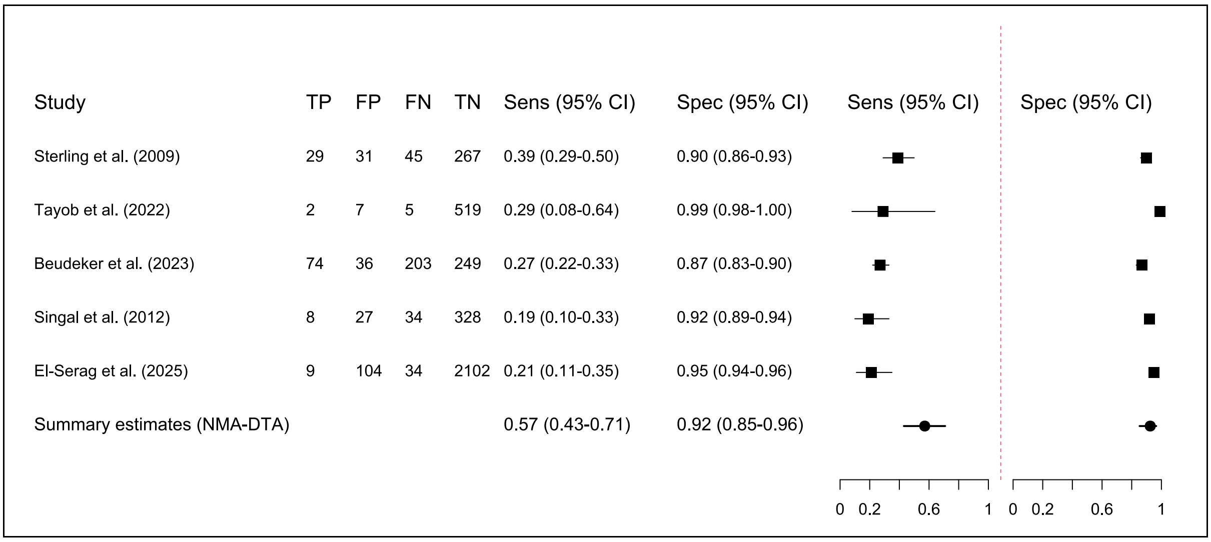

| Study | TP | FP | FN | TN | Sens (95% CI) | Spec (95% CI) |
|---|---|---|---|---|---|---|
| Sterling et al. (2009) | 29 | 31 | 45 | 267 | 0.39 (0.29-0.50) | 0.90 (0.86-0.93) |
| Tayob et al. (2022) | 2 | 7 | 5 | 519 | 0.29 (0.08-0.64) | 0.99 (0.98-1.00) |
| Beudeker et al. (2023) | 74 | 36 | 203 | 249 | 0.27 (0.22-0.33) | 0.87 (0.83-0.90) |
| Singal et al. (2012) | 8 | 27 | 34 | 328 | 0.19 (0.10-0.33) | 0.92 (0.89-0.94) |
| El-Serag et al. (2025) | 9 | 104 | 34 | 2102 | 0.21 (0.11-0.35) | 0.95 (0.94-0.96) |
| Summary estimates (NMA-DTA) | | | | | 0.57 (0.43-0.71) | 0.92 (0.85-0.96) |

**Figure S15.4 Forest plot: DCP at threshold 7.5 ng/ml**

## S15.5. AFP-L3 or DCP (mAU/ml)

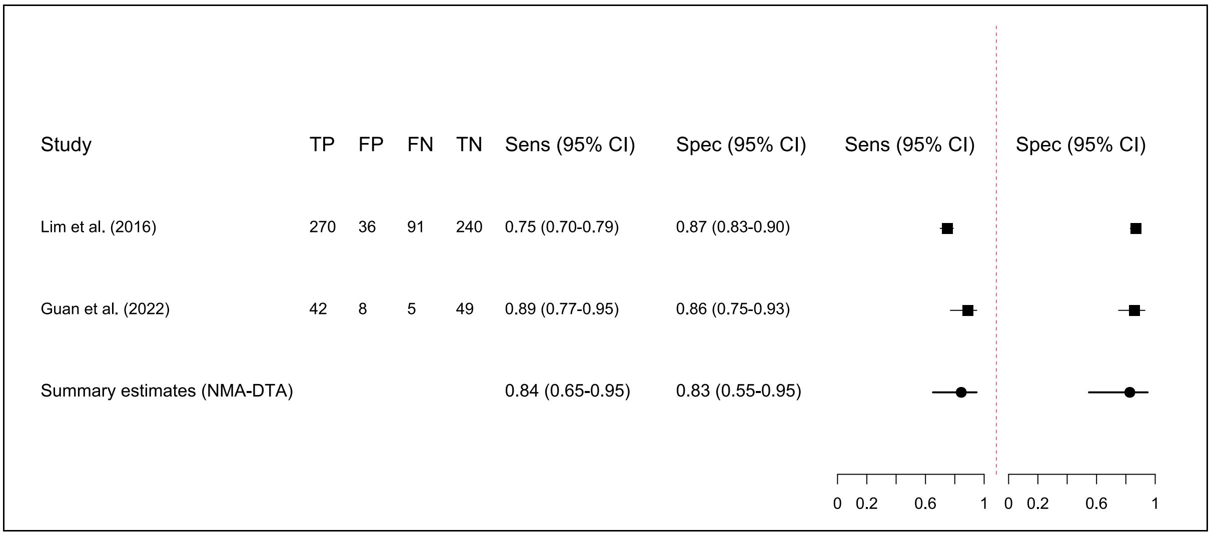

| Study | TP | FP | FN | TN | Sens (95% CI) | Spec (95% CI) |
|---|---|---|---|---|---|---|
| Lim et al. (2016) | 270 | 36 | 91 | 240 | 0.75 (0.70-0.79) | 0.87 (0.83-0.90) |
| Guan et al. (2022) | 42 | 8 | 5 | 49 | 0.89 (0.77-0.95) | 0.86 (0.75-0.93) |
| Summary estimates (NMA-DTA) | | | | | 0.84 (0.65-0.95) | 0.83 (0.55-0.95) |

**Figure S15.5 Forest plot: AFP-L3 or DCP (mAU/ml)**

## S15.6. AFP-L3 or DCP (ng/ml)

| Study | TP | FP | FN | TN | Sens (95% CI) | Spec (95% CI) |
|---|---|---|---|---|---|---|
| Sterling et al. (2009) | 46 | 52 | 28 | 246 | 0.62 (0.51-0.72) | 0.83 (0.78-0.87) |
| Brigida et al. (2020) | 23 | 0 | 7 | 30 | 0.77 (0.59-0.88) | 1.00 (0.89-1.00) |
| Summary estimates (NMA-DTA) | | | | | 0.68 (0.42-0.86) | 0.94 (0.78-0.99) |

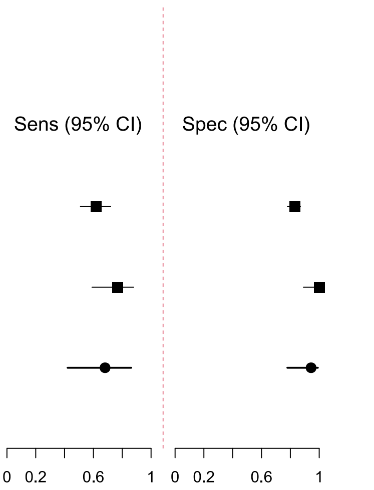


**Figure S15.6 Forest plot: AFP-L3 or DCP (ng/ml)**

## S15.7. AFP and AFP-L3

| Study | TP | FP | FN | TN | Sens (95% CI) | Spec (95% CI) |
|---|---|---|---|---|---|---|
| Sterling et al. (2012) | 5 | 9 | 15 | 349 | 0.25 (0.11-0.47) | 0.97 (0.95-0.98) |
| Zinkin et al. (2008) | 24 | 1 | 17 | 50 | 0.59 (0.44-0.73) | 0.98 (0.90-1.00) |
| Summary estimates (NMA-DTA) | | | | | 0.43 (0.18-0.71) | 0.99 (0.94-1.00) |

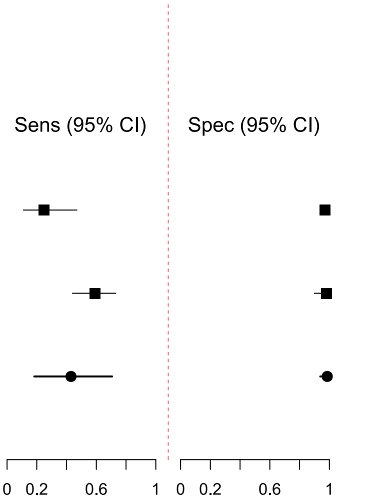


**Figure S15.7 Forest plot: AFP and AFP-L3**

## S15.8. AFP and DCP(mAU/mL)

| Study | TP | FP | FN | TN | Sens (95% CI) | Spec (95% CI) |
|---|---|---|---|---|---|---|
| Sterling et al. (2012) | 6 | 20 | 14 | 338 | 0.30 (0.15-0.52) | 0.94 (0.91-0.96) |
| Song et al. (2020) | 31 | 0 | 24 | 64 | 0.56 (0.43-0.68) | 1.00 (0.94-1.00) |
| Loglio et al. (2020) | 19 | 0 | 45 | 148 | 0.30 (0.20-0.42) | 1.00 (0.97-1.00) |
| Summary estimates (NMA-DTA) | | | | | 0.54 (0.32-0.74) | 0.98 (0.92-1.00) |

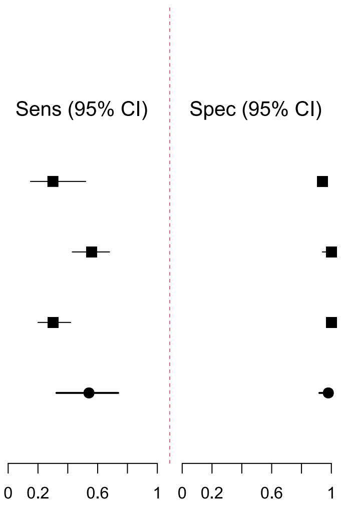


**Figure S15.8 Forest plot: AFP and DCP(mAU/mL)**

## S15.9. AFP or AFP-L3

| Study | TP | FP | FN | TN | Sens (95% CI) | Spec (95% CI) |
|---|---|---|---|---|---|---|
| Sterling et al. (2009) | 51 | 100 | 23 | 198 | 0.69 (0.58-0.78) | 0.66 (0.60-0.71) |
| Guan et al. (2022) | 34 | 13 | 13 | 44 | 0.72 (0.58-0.83) | 0.77 (0.65-0.86) |
| Summary estimates (NMA-DTA) | | | | | 0.73 (0.49-0.89) | 0.73 (0.41-0.91) |

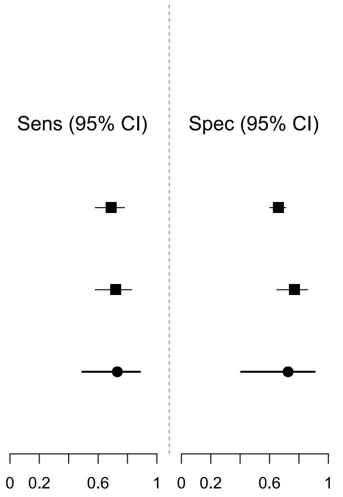


**Figure S15.9 Forest plot: AFP or AFP-L3**

## S15.10. AFP or AFP-L3 or DCP (mAU/ml)

| Study | TP | FP | FN | TN | Sens (95% CI) | Spec (95% CI) |
|---|---|---|---|---|---|---|
| Volk et al. (2007) | 74 | 15 | 10 | 154 | 0.88 (0.79-0.93) | 0.91 (0.86-0.94) |
| Guan et al. (2022) | 42 | 5 | 5 | 52 | 0.89 (0.77-0.95) | 0.91 (0.81-0.96) |
| Summary estimates (NMA-DTA) | | | | | 0.88 (0.70-0.96) | 0.88 (0.64-0.97) |

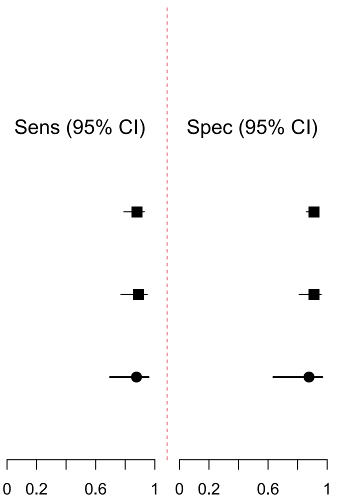


**Figure S15.10 Forest plot: AFP or AFP-L3 or DCP (mAU/ml)**

## S15.11. AFP or AFP-L3 or DCP (ng/ml)

| Study | TP | FP | FN | TN | Sens (95% CI) | Spec (95% CI) |
|---|---|---|---|---|---|---|
| Sterling et al. (2009) | 57 | 121 | 17 | 177 | 0.77 (0.66-0.85) | 0.59 (0.53-0.64) |
| Tayob et al. (2022) | 8 | 48 | 18 | 465 | 0.31 (0.17-0.50) | 0.91 (0.88-0.93) |
| Brigida et al. (2020) | 26 | 10 | 4 | 20 | 0.87 (0.71-0.95) | 0.67 (0.49-0.81) |
| Summary estimates (NMA-DTA) | | | | | 0.76 (0.53-0.89) | 0.75 (0.50-0.90) |

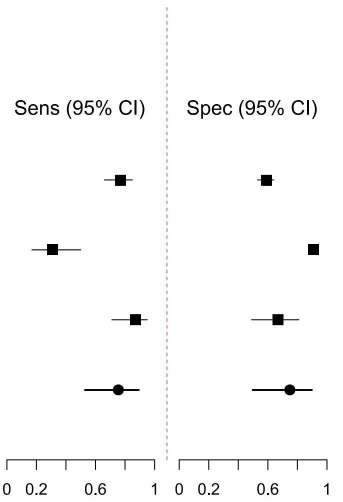


**Figure S15.11 Forest plot: AFP or AFP-L3 or DCP (ng/ml)**

## S15.12. AFP or B-mode US

| Study | TP | FP | FN | TN | Sens (95% CI) | Spec (95% CI) |
|---|---|---|---|---|---|---|
| Kim et al. (2019) | 58 | 7 | 6 | 321 | 0.91 (0.81-0.96) | 0.98 (0.96-0.99) |
| Chang et al. (2015) | 360 | 391 | 3 | 843 | 0.99 (0.97-1.00) | 0.68 (0.65-0.71) |
| Singal et al. (2012) | 24 | 67 | 7 | 334 | 0.77 (0.60-0.88) | 0.83 (0.79-0.86) |
| Kim et al. (2017) | 13 | 36 | 15 | 344 | 0.46 (0.29-0.64) | 0.91 (0.88-0.93) |
| Hernandez et al. (2011) | 20 | 0 | 2 | 167 | 0.91 (0.72-0.98) | 1.00 (0.98-1.00) |
| Patel et al. (2025) | 195 | 48 | 31 | 229 | 0.86 (0.81-0.90) | 0.83 (0.78-0.87) |
| Summary estimates (NMA-DTA) | | | | | 0.90 (0.79-0.96) | 0.89 (0.77-0.95) |

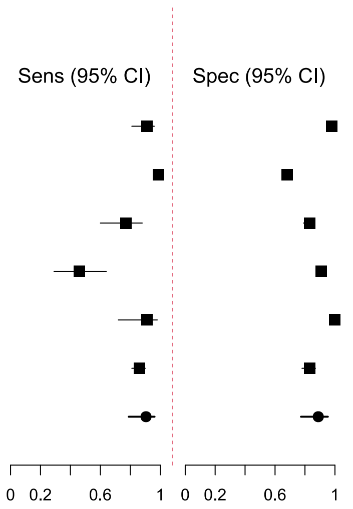


**Figure S15.12 Forest plot: AFP or B-mode US**

## S15.13. AFP or DCP (mAU/ml)

| Study | TP | FP | FN | TN | Sens (95% CI) | Spec (95% CI) |
|---|---|---|---|---|---|---|
| Ricco et al. (2018) | 208 | 43 | 50 | 87 | 0.81 (0.76-0.85) | 0.67 (0.59-0.74) |
| Sultanik et al. (2017) | 40 | 28 | 6 | 88 | 0.87 (0.74-0.94) | 0.76 (0.67-0.83) |
| Lim et al. (2016) | 282 | 62 | 79 | 214 | 0.78 (0.73-0.82) | 0.78 (0.73-0.82) |
| Marrero et al. (2009) | 360 | 154 | 59 | 263 | 0.86 (0.82-0.89) | 0.63 (0.58-0.67) |
| Volk et al. (2007) | 74 | 19 | 10 | 150 | 0.88 (0.79-0.93) | 0.89 (0.83-0.93) |
| Guan et al. (2022) | 45 | 6 | 2 | 51 | 0.96 (0.86-0.99) | 0.89 (0.78-0.95) |
| Summary estimates (NMA-DTA) | | | | | 0.87 (0.80-0.92) | 0.78 (0.62-0.88) |

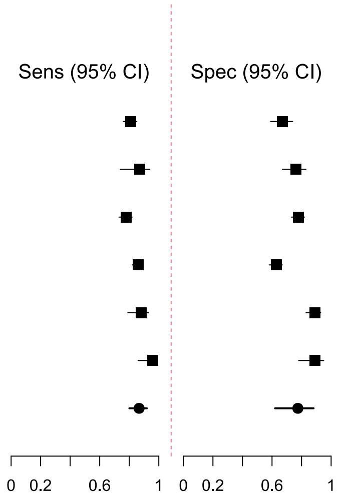


**Figure S15.13 Forest plot: AFP or DCP (mAU/ml)**

## S15.14. AFP or DCP (ng/ml)

| Study | TP | FP | FN | TN | Sens (95% CI) | Spec (95% CI) |
|---|---|---|---|---|---|---|
| Sterling et al. (2009) | 52 | 109 | 22 | 189 | 0.70 (0.59-0.79) | 0.63 (0.57-0.68) |
| Beale et al. (2008) | 47 | 8 | 3 | 33 | 0.94 (0.84-0.98) | 0.80 (0.65-0.89) |
| Brigida et al. (2020) | 26 | 10 | 4 | 20 | 0.87 (0.71-0.95) | 0.67 (0.49-0.81) |
| Summary estimates (NMA-DTA) | | | | | 0.83 (0.65-0.94) | 0.74 (0.47-0.90) |

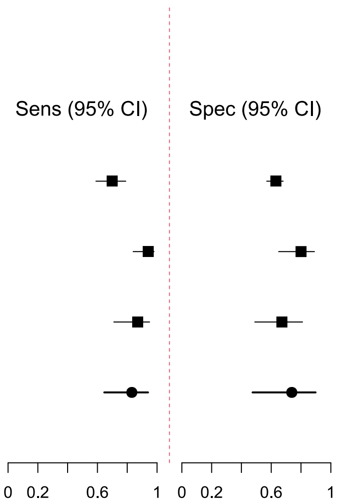


**Figure S15.14 Forest plot: AFP or DCP (ng/ml)**

## S15.15. B-mode US

| Study | TP | FP | FN | TN | Sens (95% CI) | Spec (95% CI) |
|---|---|---|---|---|---|---|
| Kudo et al. (2019) | 17 | 9 | 9 | 278 | 0.65 (0.46-0.80) | 0.97 (0.94-0.98) |
| Kim et al. (2019) | 36 | 0 | 28 | 328 | 0.56 (0.44-0.67) | 1.00 (0.99-1.00) |
| Atiq et al. (2017) | 33 | 119 | 45 | 326 | 0.42 (0.32-0.53) | 0.73 (0.69-0.77) |
| Villacastin Ruiz et al. (2016) | 86 | 6 | 21 | 157 | 0.80 (0.71-0.86) | 0.96 (0.92-0.98) |
| Chang et al. (2015) | 334 | 318 | 29 | 916 | 0.92 (0.89-0.94) | 0.74 (0.71-0.76) |
| Pinero et al. (2015) | 19 | 2 | 37 | 514 | 0.34 (0.23-0.47) | 1.00 (0.99-1.00) |
| Jalli et al. (2015) | 18 | 9 | 12 | 57 | 0.60 (0.42-0.75) | 0.86 (0.76-0.92) |
| Singal et al. (2012) | 18 | 34 | 13 | 367 | 0.58 (0.41-0.74) | 0.92 (0.89-0.94) |
| Yu et al. (2011) | 88 | 11 | 50 | 281 | 0.64 (0.56-0.72) | 0.96 (0.93-0.98) |
| Yoon et al. (2020) | 7 | 14 | 17 | 99 | 0.29 (0.15-0.49) | 0.88 (0.81-0.93) |
| Chong et al. (2022) | 139 | 178 | 46 | 5516 | 0.75 (0.68-0.81) | 0.97 (0.97-0.97) |
| Loglio et al. (2020) | 35 | 0 | 29 | 148 | 0.55 (0.43-0.67) | 1.00 (0.97-1.00) |
| Park et al. (2019) | 6 | 21 | 6 | 491 | 0.50 (0.25-0.75) | 0.96 (0.94-0.97) |
| Kim et al. (2017) | 12 | 59 | 31 | 998 | 0.28 (0.17-0.43) | 0.94 (0.92-0.95) |
| Worland et al. (2018) | 9 | 7 | 3 | 45 | 0.75 (0.47-0.91) | 0.87 (0.75-0.94) |
| Pocha et al. (2013) | 7 | 3 | 2 | 71 | 0.78 (0.45-0.94) | 0.96 (0.89-0.99) |
| Hernandez et al. (2011) | 19 | 0 | 3 | 167 | 0.86 (0.66-0.95) | 1.00 (0.98-1.00) |
| Patel et al. (2025) | 164 | 19 | 62 | 258 | 0.73 (0.67-0.78) | 0.93 (0.89-0.95) |
| Seif El Dahan et al. (2025) | 150 | 117 | 61 | 305 | 0.71 (0.65-0.77) | 0.72 (0.68-0.76) |
| Chen et al. (2024) | 18 | 14 | 57 | 2203 | 0.24 (0.16-0.35) | 0.99 (0.98-0.99) |
| Summary estimates (NMA-DTA) | | | | | 0.70 (0.62-0.78) | 0.94 (0.90-0.97) |

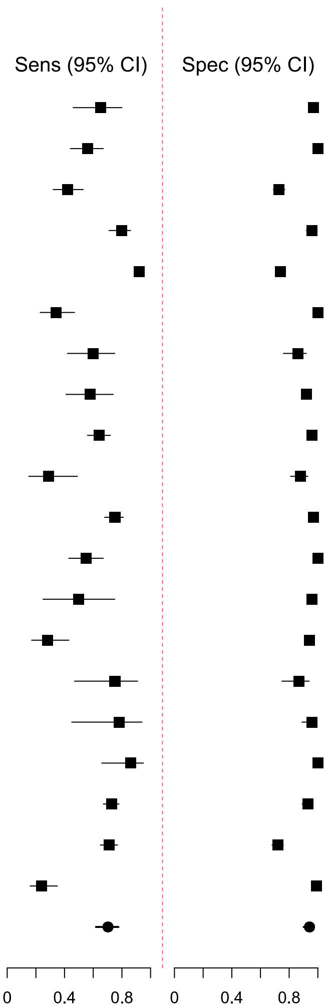


**Figure S15.15 Forest plot: B-mode US**

## S15.16. CEUS

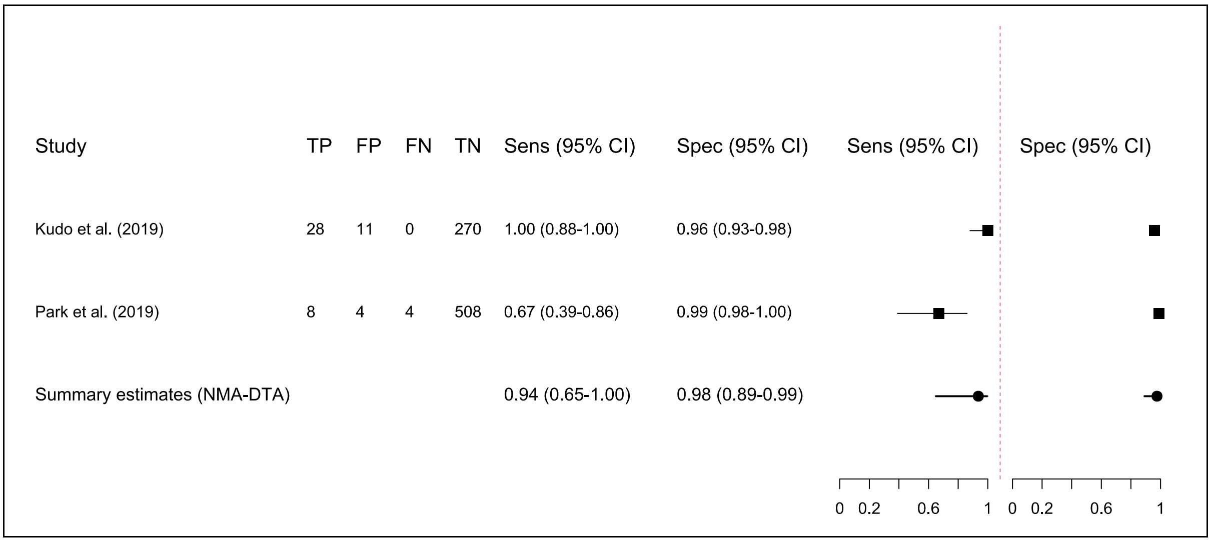


**Figure S15.16 Forest plot: CEUS**

## S15.17. Doylestown algorithm

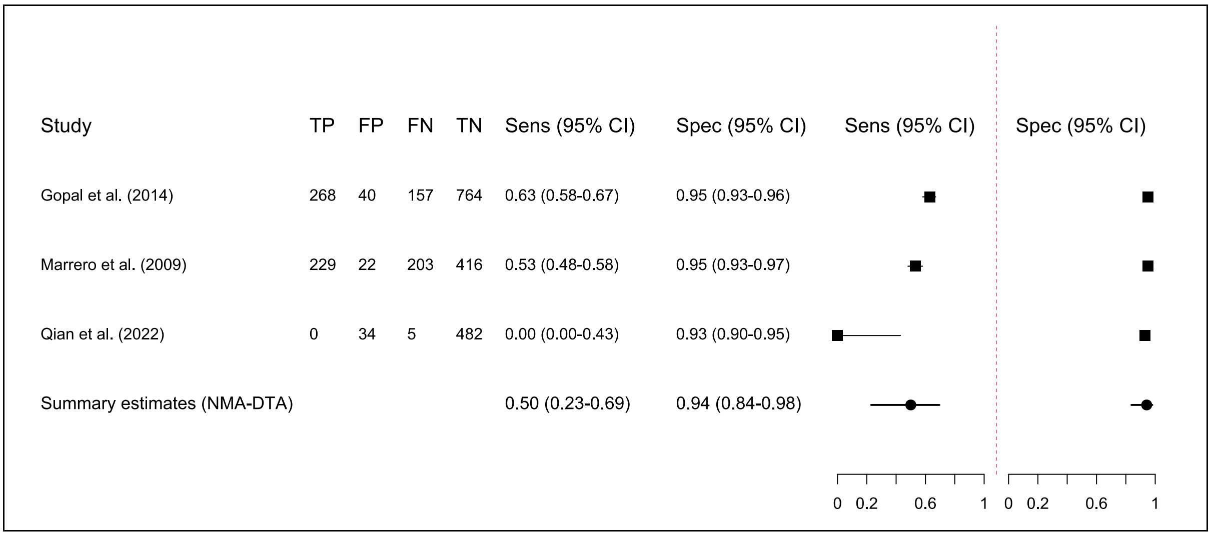


**Figure S15.17 Forest plot: Doylestown algorithm**

## S15.18. GAAD

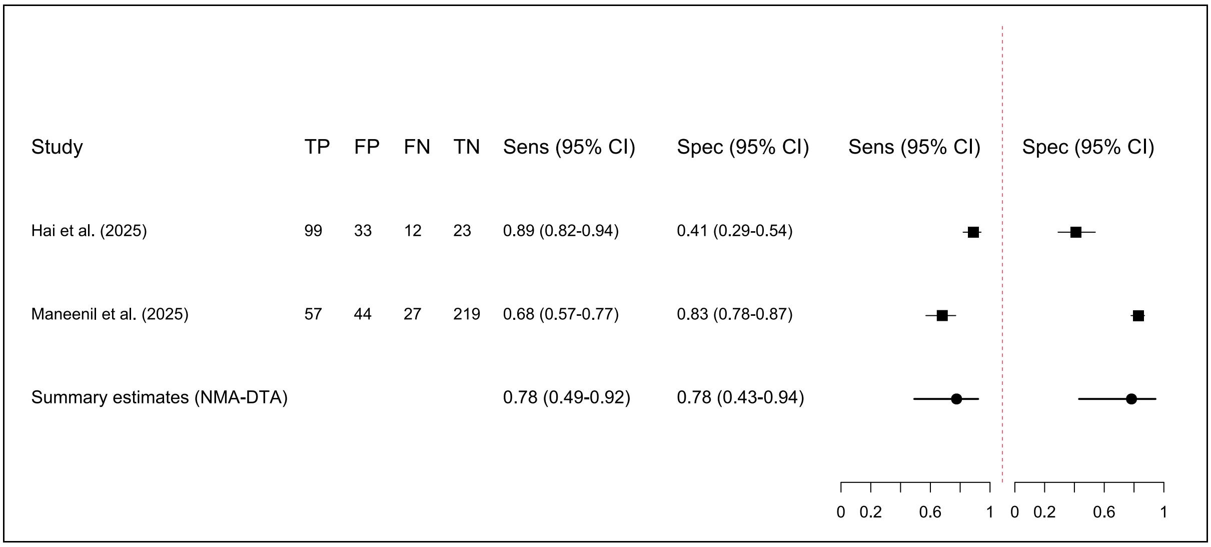

| Study | TP | FP | FN | TN | Sens (95% CI) | Spec (95% CI) |
|---|---|---|---|---|---|---|
| Hai et al. (2025) | 99 | 33 | 12 | 23 | 0.89 (0.82-0.94) | 0.41 (0.29-0.54) |
| Maneenil et al. (2025) | 57 | 44 | 27 | 219 | 0.68 (0.57-0.77) | 0.83 (0.78-0.87) |
| Summary estimates (NMA-DTA) | | | | | 0.78 (0.49-0.92) | 0.78 (0.43-0.94) |



**Figure S15.18 Forest plot: GAAD**

## S15.19. GALAD

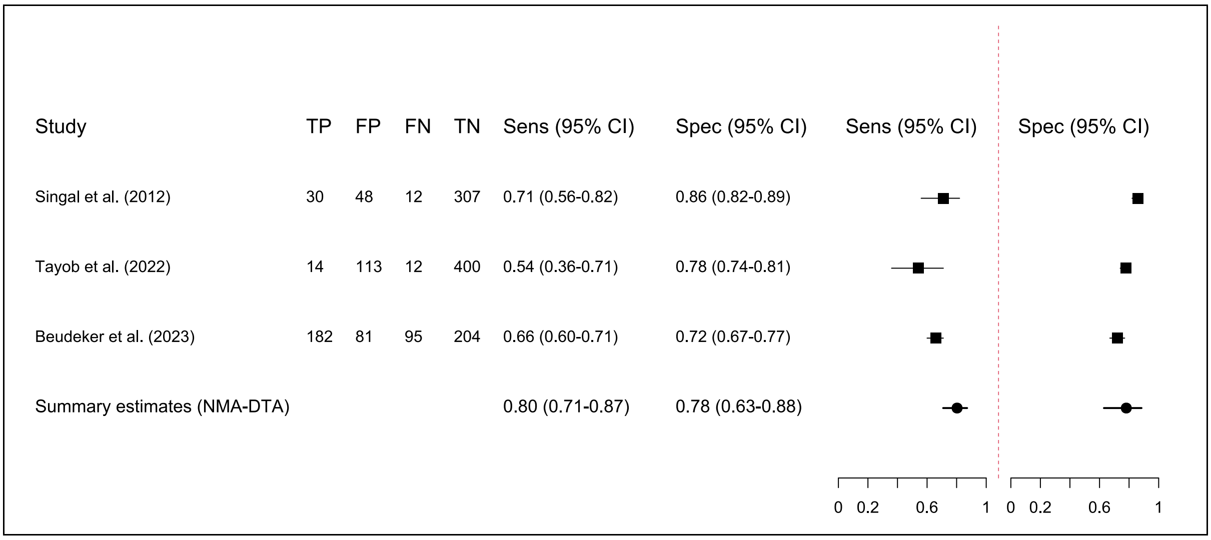

| Study | TP | FP | FN | TN | Sens (95% CI) | Spec (95% CI) |
|---|---|---|---|---|---|---|
| Singal et al. (2012) | 30 | 48 | 12 | 307 | 0.71 (0.56-0.82) | 0.86 (0.82-0.89) |
| Tayob et al. (2022) | 14 | 113 | 12 | 400 | 0.54 (0.36-0.71) | 0.78 (0.74-0.81) |
| Beudeker et al. (2023) | 182 | 81 | 95 | 204 | 0.66 (0.60-0.71) | 0.72 (0.67-0.77) |
| Summary estimates (NMA-DTA) | | | | | 0.80 (0.71-0.87) | 0.78 (0.63-0.88) |



**Figure S15.19 Forest plot: GALAD**

## S15.20. HES algorithm

| Study | TP | FP | FN | TN | Sens (95% CI) | Spec (95% CI) |
|---|---|---|---|---|---|---|
| Singal et al. (2012) | 18 | 34 | 24 | 321 | 0.43 (0.29-0.58) | 0.90 (0.86-0.93) |
| Tayob et al. (2022) | 8 | 31 | 25 | 495 | 0.24 (0.13-0.41) | 0.94 (0.92-0.96) |
| El-Serag et al. (2025) | 13 | 60 | 30 | 2146 | 0.30 (0.18-0.45) | 0.97 (0.96-0.98) |
| Summary estimates (NMA-DTA) | | | | | 0.54 (0.33-0.72) | 0.92 (0.79-0.97) |

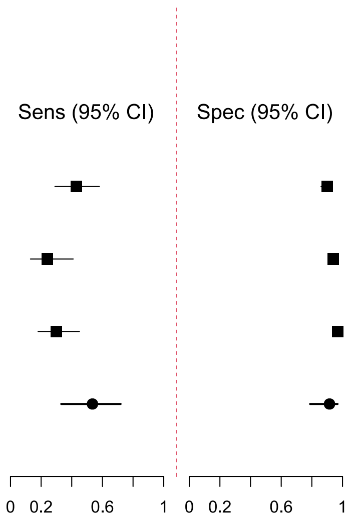


Figure S15.20 Forest plot: HES algorithm

## S15.21. HOTAIR expression

| Study | TP | FP | FN | TN | Sens (95% CI) | Spec (95% CI) |
|---|---|---|---|---|---|---|
| El-Shendidi et al. (2022) | 27 | 3 | 13 | 37 | 0.68 (0.53-0.80) | 0.92 (0.79-0.97) |
| Khalil et al. (2024) | 55 | 2 | 5 | 58 | 0.92 (0.82-0.97) | 0.97 (0.89-0.99) |
| Pan et al. (2024) | 29 | 10 | 8 | 21 | 0.78 (0.62-0.88) | 0.68 (0.50-0.82) |
| Summary estimates (NMA-DTA) | | | | | 0.75 (0.51-0.90) | 0.87 (0.61-0.97) |

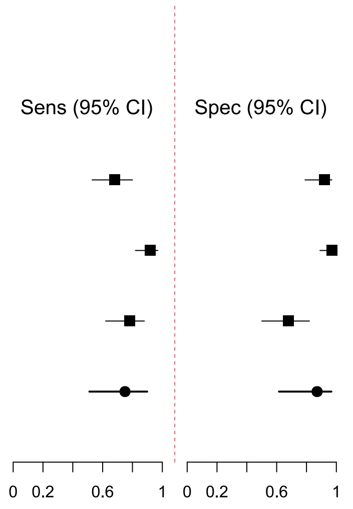


Figure S15.21 Forest plot: HOTAIR expression

## S15.22. hsa-miR-155-5p-fold changes

| Study | TP | FP | FN | TN | Sens (95% CI) | Spec (95% CI) |
|---|---|---|---|---|---|---|
| Eldosoky et al. (2023) | 28 | 21 | 11 | 19 | 0.72 (0.56-0.84) | 0.48 (0.33-0.63) |
| Hammad et al. (2023) | 28 | 21 | 11 | 19 | 0.72 (0.56-0.84) | 0.48 (0.33-0.63) |
| Summary estimates (NMA-DTA) | | | | | 0.68 (0.40-0.88) | 0.34 (0.09-0.71) |

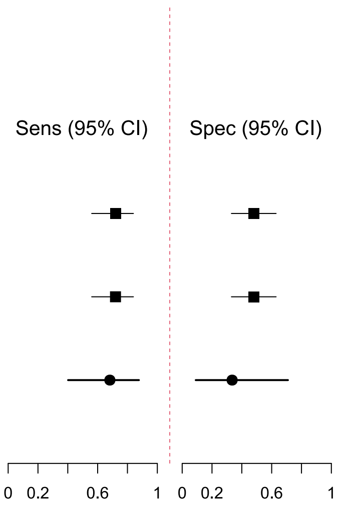


**Figure S15.22 Forest plot: hsa-miR-155-5p-fold changes**

## S15.23. hsa-miR-21-5p-fold changes

| Study | TP | FP | FN | TN | Sens (95% CI) | Spec (95% CI) |
|---|---|---|---|---|---|---|
| Eldosoky et al. (2023) | 39 | 22 | 10 | 18 | 0.80 (0.67-0.89) | 0.45 (0.31-0.60) |
| Hammad et al. (2023) | 29 | 22 | 10 | 18 | 0.74 (0.59-0.85) | 0.45 (0.31-0.60) |
| Summary estimates (NMA-DTA) | | | | | 0.74 (0.46-0.90) | 0.31 (0.08-0.69) |

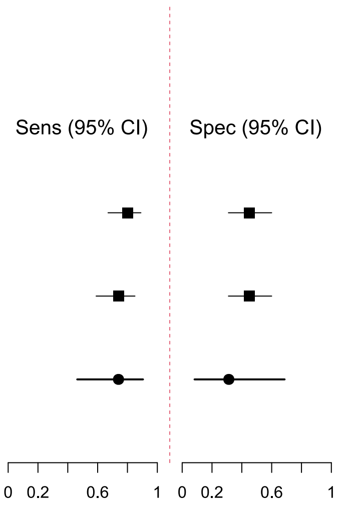


**Figure S15.23 Forest plot: hsa-miR-21-5p-fold changes**

## S15.24. MALAT1 RNA

| Study | TP | FP | FN | TN | Sens (95% CI) | Spec (95% CI) |
|---|---|---|---|---|---|---|
| Toraih et al. (2018) | 18 | 8 | 12 | 12 | 0.60 (0.42-0.75) | 0.60 (0.39-0.78) |
| Khalil et al. (2024) | 58 | 6 | 2 | 54 | 0.97 (0.89-0.99) | 0.90 (0.80-0.95) |
| Summary estimates (NMA-DTA) | | | | | 0.67 (0.12-0.97) | 0.78 (0.40-0.95) |

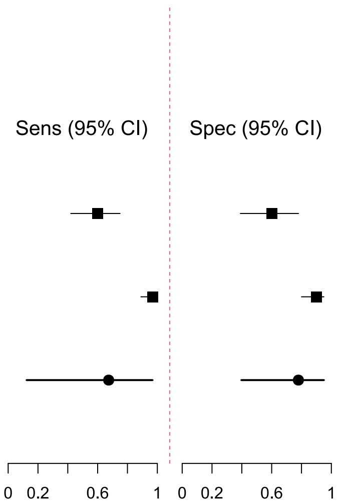


Figure S15.24 Forest plot: MALAT1 RNA

## S15.25. miR-122

| Study | TP | FP | FN | TN | Sens (95% CI) | Spec (95% CI) |
|---|---|---|---|---|---|---|
| Caviglia et al. (2017) | 20 | 7 | 13 | 23 | 0.61 (0.44-0.76) | 0.77 (0.59-0.88) |
| Nafeh et al. (2024) | 10 | 1 | 2 | 21 | 0.83 (0.55-0.95) | 0.95 (0.78-0.99) |
| Summary estimates (NMA-DTA) | | | | | 0.69 (0.35-0.92) | 0.88 (0.57-0.98) |

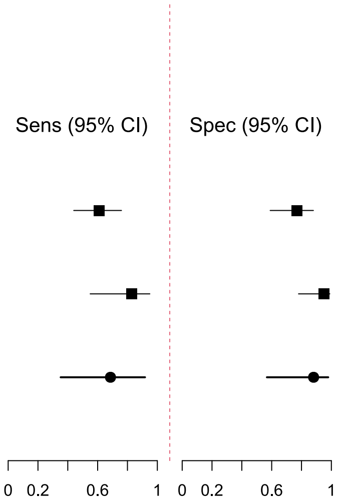


Figure S15.25 Forest plot: miR-122

## S15.26. Model based on AFP and DCP

| Study | TP | FP | FN | TN | Sens (95% CI) | Spec (95% CI) |
|---|---|---|---|---|---|---|
| Li et al. (2019) | 61 | 6 | 13 | 97 | 0.82 (0.72-0.89) | 0.94 (0.88-0.97) |
| Caviglia et al. (2020) | 104 | 32 | 45 | 168 | 0.70 (0.62-0.77) | 0.84 (0.78-0.88) |
| Chen et al. (2023) | 98 | 34 | 45 | 139 | 0.69 (0.61-0.76) | 0.80 (0.73-0.85) |
| Summary estimates (NMA-DTA) | | | | | 0.74 (0.56-0.87) | 0.87 (0.68-0.95) |

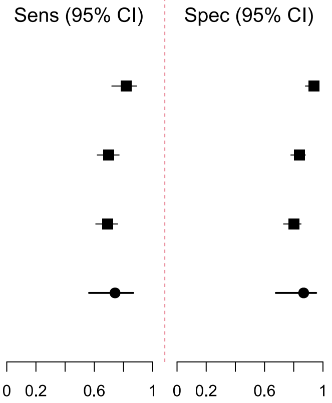


Figure S15.26 Forest plot: Model based on AFP and DCP

## S15.27. MRI (dynamic contrast-enhanced)

| Study | TP | FP | FN | TN | Sens (95% CI) | Spec (95% CI) |
|---|---|---|---|---|---|---|
| Yu et al. (2011) | 99 | 13 | 18 | 86 | 0.85 (0.77-0.90) | 0.87 (0.79-0.92) |
| Demirtas et al. (2020) | 30 | 12 | 5 | 247 | 0.86 (0.71-0.94) | 0.95 (0.92-0.97) |
| Kim et al. (2017) | 37 | 32 | 6 | 1025 | 0.86 (0.73-0.93) | 0.97 (0.96-0.98) |
| Kim et al. (2017) | 54 | 12 | 21 | 25 | 0.72 (0.61-0.81) | 0.68 (0.52-0.81) |
| Baird et al. (2013) | 11 | 0 | 4 | 15 | 0.73 (0.48-0.89) | 1.00 (0.80-1.00) |
| Choi et al. (2008) | 20 | 5 | 3 | 19 | 0.87 (0.68-0.95) | 0.79 (0.59-0.91) |
| Hanna et al. (2008) | 24 | 2 | 1 | 21 | 0.96 (0.80-0.99) | 0.91 (0.73-0.97) |
| Lauenstein et al. (2007) | 24 | 2 | 3 | 86 | 0.89 (0.72-0.96) | 0.98 (0.92-0.99) |
| Zheng et al. (2021) | 27 | 0 | 5 | 32 | 0.84 (0.68-0.93) | 1.00 (0.89-1.00) |
| Yokoo et al. (2023) | 142 | 15 | 19 | 123 | 0.88 (0.82-0.92) | 0.89 (0.83-0.93) |
| Seif El Dahan et al. (2025) | 173 | 35 | 43 | 397 | 0.80 (0.74-0.85) | 0.92 (0.89-0.94) |
| Summary estimates (NMA-DTA) | | | | | 0.90 (0.83-0.94) | 0.92 (0.85-0.96) |

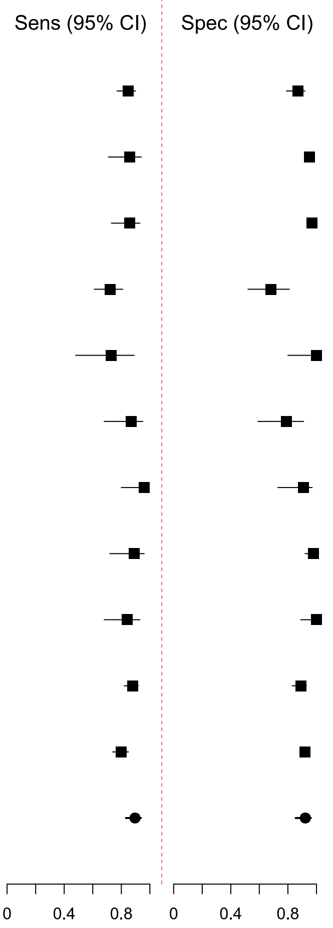


**Figure S15.27 Forest plot: MRI (dynamic contrast-enhanced)**

## S15.28. MRI (noncontrast)

| Study | TP | FP | FN | TN | Sens (95% CI) | Spec (95% CI) |
|---|---|---|---|---|---|---|
| Jalli et al. (2015) | 25 | 0 | 5 | 66 | 0.83 (0.66-0.92) | 1.00 (0.94-1.00) |
| Kim et al. (2017) | 34 | 21 | 9 | 993 | 0.79 (0.65-0.89) | 0.98 (0.97-0.99) |
| Summary estimates (NMA-DTA) | | | | | 0.92 (0.75-0.97) | 0.98 (0.90-1.00) |

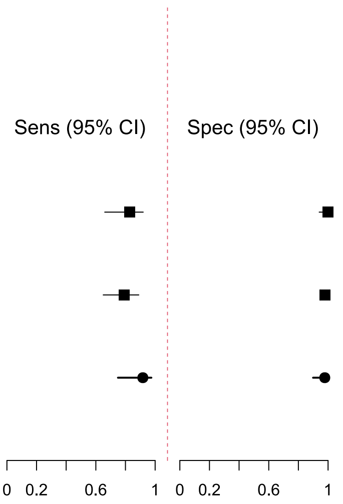


Figure S15.28 Forest plot: MRI (noncontrast)

## S15.29. mSEPT9

| Study | TP | FP | FN | TN | Sens (95% CI) | Spec (95% CI) |
|---|---|---|---|---|---|---|
| Lewin et al. (2021) | 46 | 37 | 14 | 66 | 0.77 (0.65-0.86) | 0.64 (0.54-0.73) |
| Oussalah et al. (2018) | 48 | 21 | 3 | 114 | 0.94 (0.84-0.98) | 0.84 (0.77-0.89) |
| Oussalah et al. (2018) | 40 | 5 | 7 | 51 | 0.85 (0.72-0.93) | 0.91 (0.81-0.96) |
| Summary estimates (NMA-DTA) | | | | | 0.89 (0.75-0.96) | 0.77 (0.45-0.93) |

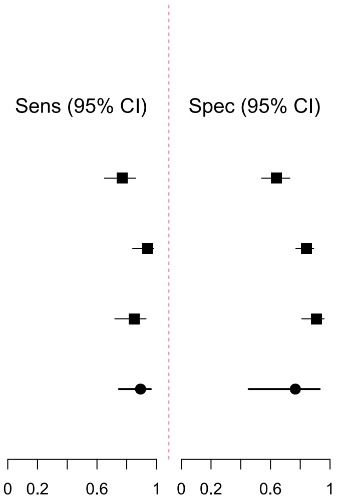


Figure S15.29 Forest plot: mSEPT9

## S15.30. Multiphase CT

| Study | TP | FP | FN | TN | Sens (95% CI) | Spec (95% CI) |
|---|---|---|---|---|---|---|
| Tahon et al. (2019) | 40 | 0 | 0 | 30 | 1.00 (0.91-1.00) | 1.00 (0.89-1.00) |
| Villacastin Ruiz et al. (2016) | 77 | 6 | 18 | 152 | 0.81 (0.72-0.88) | 0.96 (0.92-0.98) |
| Yu et al. (2011) | 113 | 11 | 36 | 236 | 0.76 (0.69-0.82) | 0.96 (0.93-0.98) |
| Yoon et al. (2020) | 20 | 5 | 4 | 108 | 0.83 (0.64-0.93) | 0.96 (0.91-0.98) |
| Kim et al. (2017) | 42 | 10 | 33 | 27 | 0.56 (0.45-0.67) | 0.73 (0.57-0.85) |
| Monzawa et al. (2007) | 45 | 12 | 3 | 53 | 0.94 (0.83-0.98) | 0.82 (0.71-0.89) |
| Jia et al. (2017) | 33 | 9 | 2 | 43 | 0.94 (0.81-0.98) | 0.83 (0.71-0.91) |
| Pocha et al. (2013) | 7 | 9 | 1 | 63 | 0.88 (0.53-0.98) | 0.88 (0.79-0.94) |
| Summary estimates (NMA-DTA) | | | | | 0.89 (0.79-0.95) | 0.93 (0.85-0.96) |

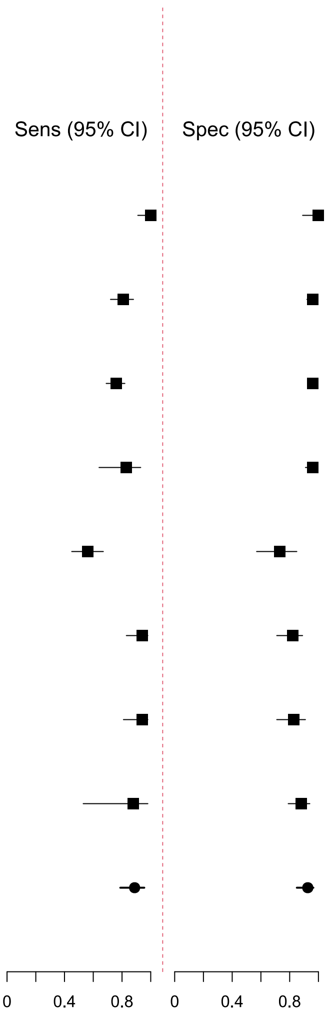


**Figure S15.30 Forest plot: Multiphase CT**

## S15.31. Serial AFP – absolute value AND doubling since last observation

| Study | TP | FP | FN | TN | Sens (95% CI) | Spec (95% CI) |
|---|---|---|---|---|---|---|
| Chang et al. (2015) | 360 | 350 | 3 | 884 | 0.99 (0.97-1.00) | 0.72 (0.69-0.74) |
| Sterling et al. (2012) | 5 | 21 | 15 | 337 | 0.25 (0.11-0.47) | 0.94 (0.91-0.96) |
| Summary estimates (NMA-DTA) | | | | | 0.86 (0.21-0.99) | 0.92 (0.73-0.98) |

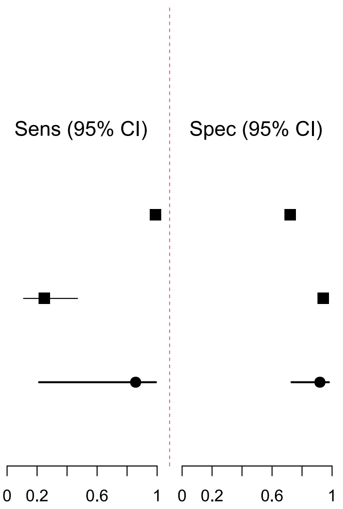


**Figure S15.31 Forest plot: Serial AFP – absolute value AND doubling since last observation**

## Appendix S16 Summary graphic

Our summary builds on an approach that was widely tested among patient representatives, media representatives and health professionals[10]. It uses stage-specific sensitivity data (*Table 2* in main text) and overall specificity estimates (*Figure 2* in main text) to estimate expected outcomes for a cohort of 1,000 people with cirrhosis undergoing a single diagnostic assessment. We estimate the accuracy of $AFP_{\cdot OR \cdot}US$ by combining the results for each test, assuming independence.

To complete the calculations, we need to assume a value for the true prevalence of HCC in such a population, and the distribution of stages among undiagnosed cases. In practice, the exact prevalence of undiagnosed HCC and true stage-distribution of cases in any cross-sectional population of people with cirrhosis will depend on a variety of factors, including the aetiology of cirrhosis, treatment history, how frequently (if at all) members of the population have previously undergone diagnostic tests, and which tests they received.

However, in order to provide comparable estimates for each test in our evidence-base, we must assume the same baseline parameters. Therefore, our illustrative summary uses an indicative prevalence of 10%, which is approximately typical of the studies our review includes that recruited participants in the context of an active surveillance program (e.g. 8.3% in a large US series[11]; 10.7% in a Danish nationwide study[12]).

The true split between very early, early, and late undiagnosed HCC cases is impossible to observe empirically, without being influenced by the accuracy of whatever tools were used to diagnose cases, and how recently and frequently those tests were applied. However, one Canadian study usefully reports stage-distribution of 54 HCCs diagnosed at the **first** screen of an ultrasound surveillance program: 14 (26%) $BCLC_0$, 26 (50%) $BCLC_A$, 11 (20%) $BCLC_{BCD}$ (with 2 [4%] 'missed')[13]. This is a good start, as it is a cross-sectional estimate unaffected by previous surveillance tests, but it does not account for cases the first scans must have missed. However, if we assume that the stage-stratified sensitivity of ultrasound in this program was typical of that observed in our evidence-base (*Table 2* in main text), we can calculate the underlying prevalence that would have led to these results: 14/0.33=42 $BCLC_0$, 26/0.61=44 $BCLC_A$, 11/0.85=13 $BCLC_{BCD}$, implying a distribution of 42%, 44%, and 13% of cases, respectively. Based on this estimate, we use a 40:45:15 split, rounded to the nearest 5% to avoid spurious precision, for our summary graphic.

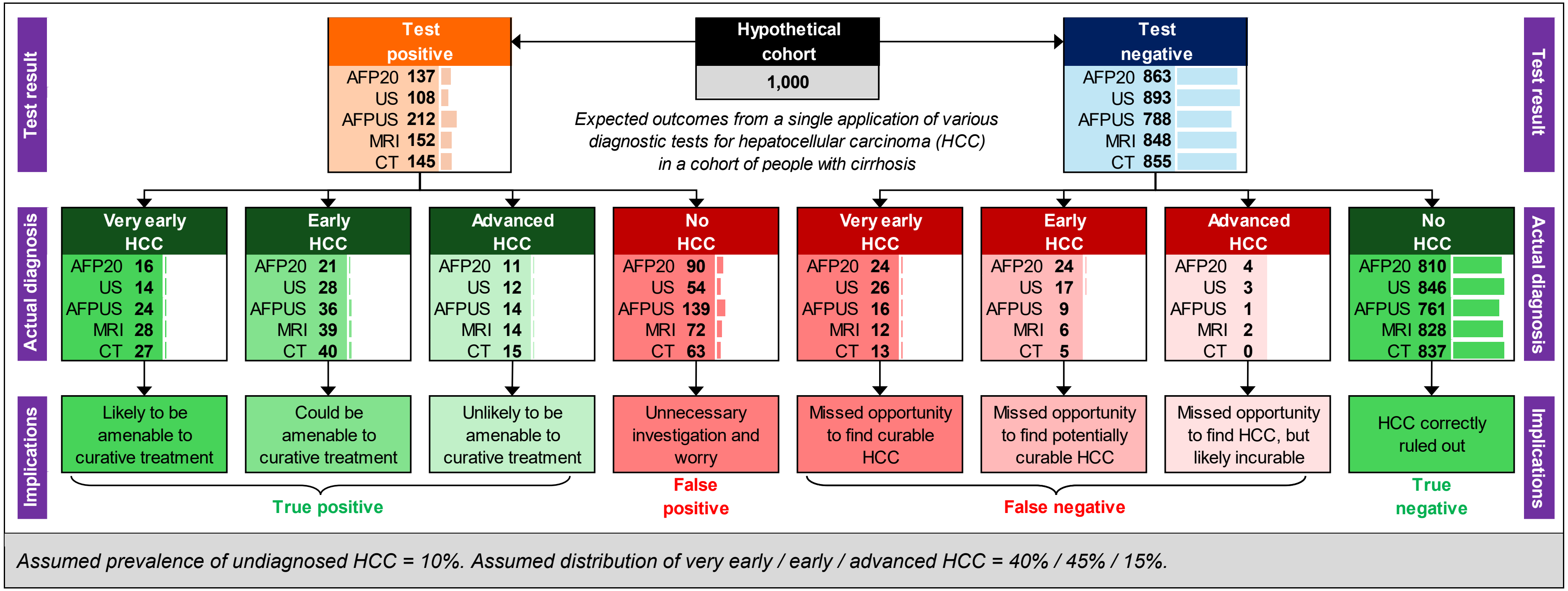


**Figure S16.1 Summary graphic showing expected test outcomes for a cohort of 1,000 people with cirrhosis**

## References for appendices


1. Thompson Coon J, Rogers G, Hewson P, Wright D, Anderson R, Cramp M, et al. Surveillance of cirrhosis for hepatocellular carcinoma: systematic review and economic analysis. Health Technol Assess Winch Engl. 2007 Sep;11(34):1–206. doi:10.3310/hta11340 PubMed PMID: 17767898.

2. Nyaga VN, Aerts M, Arbyn M. ANOVA model for network meta-analysis of diagnostic test accuracy data. Stat Methods Med Res. 2018 Jun 1;27(6):1766–84. doi:10.1177/0962280216669182

3. Jones HE, Gatsonsis CA, Trikalinos TA, Welton NJ, Ades A e. Quantifying how diagnostic test accuracy depends on threshold in a meta-analysis. Stat Med. 2019;38(24):4789–803. doi:10.1002/sim.8301

4. Turner RM, Davey J, Clarke MJ, Thompson SG, Higgins JP. Predicting the extent of heterogeneity in meta-analysis, using empirical data from the Cochrane Database of Systematic Reviews. Int J Epidemiol. 2012 Jun 1;41(3):818–27. doi:10.1093/ije/dys041

5. Derezea E, Rogers G, Welton NJ, Jones HE. Meta-analysis of networks of diagnostic tests with binary and continuous results [Internet]. arXiv; 2026 [cited 2026 Apr 25]. Available from: http://arxiv.org/abs/2604.19996 doi:10.48550/arXiv.2604.19996

6. Chiocchia V, Nikolakopoulou A, Papakonstantinou T, Egger M, Salanti G. Agreement between ranking metrics in network meta-analysis: an empirical study. BMJ Open. 2020 Aug 1;10(8):e037744. doi:10.1136/bmjopen-2020-037744 PubMed PMID: 32819946.

7. Derezea E, Welton NJ, Rogers G, Jones HE. Meta-analysis of diagnostic test accuracy with multiple disease stages: combining stage-specific and merged-stage data [Internet]. arXiv; 2026 [cited 2026 Apr 22]. Available from: http://arxiv.org/abs/2512.12065 doi:10.48550/arXiv.2512.12065

8. Chen L, Wu T, Fan R, Qian YS, Liu JF, Bai J, et al. Cell-free DNA testing for early hepatocellular carcinoma surveillance. EBioMedicine. 2024;100:104962. Located at: Ovid MEDLINE(R) <2024>. doi:10.1016/j.ebiom.2023.104962

9. Oussalah A, Rischer S, Bensenane M, Conroy G, Filhine-Tresarrieu P, Debard R, et al. Plasma mSEPT9: A Novel Circulating Cell-free DNA-Based Epigenetic Biomarker to Diagnose Hepatocellular Carcinoma. EBioMedicine. 2018;30:138–47. doi:10.1016/j.ebiom.2018.03.029

10. Whiting P, Davenport C. Understanding test accuracy research: a test consequence graphic. Diagn Progn Res. 2018 Feb 1;2(1):2. doi:10.1186/s41512-017-0023-0

11. Chong N, Schoenberger H, Yekkaluri S, Fetzer DT, Rich NE, Yokoo T, et al. Association between ultrasound quality and test performance for HCC surveillance in patients with cirrhosis: a retrospective cohort study. Aliment Pharmacol Ther. 2022;Comment in: Aliment Pharmacol Ther. 2022 Mar;55(6):752-753 PMID: 35245956 [https://www.ncbi.nlm.nih.gov/pubmed/35245956]55(6):683–90. doi:10.1111/apt.16779

12. Hallager S, Ladelund S, Kjaer M, Madsen LG, Belard E, Laursen AL, et al. Hepatocellular carcinoma in patients with chronic hepatitis C and cirrhosis in Denmark: A nationwide cohort study. J Viral Hepat. 2018;25(1):47–55. doi:10.1111/jvh.12764

13. Khalili K, Menezes R, Kim TK, Yazdi LK, Jang HJ, Sharma S, et al. The effectiveness of ultrasound surveillance for hepatocellular carcinoma in a Canadian centre and determinants of its success. Can J Gastroenterol Hepatol. 2015;29(5):267–73. doi:10.1155/2015/563893
